# Natural variation and evolutionary dynamics of transposable elements in *Brassica oleracea* based on next-generation sequencing data

**DOI:** 10.1038/s41438-020-00367-0

**Published:** 2020-09-01

**Authors:** Zhen Liu, Miao Fan, Er-Kui Yue, Yu Li, Ruo-Fu Tao, Hai-Ming Xu, Ming-Hua Duan, Jian-Hong Xu

**Affiliations:** 1 Institute of Crop Science, Zhejiang Key Laboratory of Crop Germplasm, Zhejiang University, 310058 Hangzhou, People’s Republic of China; 2 Zhejiang Zhengjingyuan Pharmacy Chain Co., Ltd. & Hangzhou Zhengcaiyuan Pharmaceutical Co., Ltd., 310021 Hangzhou, People’s Republic of China

**Keywords:** Genetic variation, Mobile elements

## Abstract

*Brassica oleracea* comprises various economically important vegetables and presents extremely diverse morphological variations. They provide a rich source of nutrition for human health and have been used as a model system for studying polyploidization. Transposable elements (TEs) account for nearly 40% of the *B. oleracea* genome and contribute greatly to genetic diversity and genome evolution. Although the proliferation of TEs has led to a large expansion of the *B. oleracea* genome, little is known about the population dynamics and evolutionary activity of TEs. A comprehensive mobilome profile of 45,737 TE loci was obtained from resequencing data from 121 diverse accessions across nine *B. oleracea* morphotypes. Approximately 70% (32,195) of the loci showed insertion polymorphisms between or within morphotypes. In particular, up to 1221 loci were differentially fixed among morphotypes. Further analysis revealed that the distribution of the population frequency of TE loci was highly variable across different TE superfamilies and families, implying a diverse expansion history during host genome evolution. These findings provide better insight into the evolutionary dynamics and genetic diversity of *B. oleracea* genomes and will potentially serve as a valuable resource for molecular markers and association studies between TE-based genomic variations and morphotype-specific phenotypic differentiation.

## Introduction

*Brassica oleracea* (2*n* = 18, CC) is one of the most diverse species within Brassiceae, which encompasses a wide range of economically important vegetables such as cabbage, kohlrabi, cauliflower, and broccoli^1^. *B*. *oleracea* presents extremely rich morphological variation, such as leafy heads, enlarged inflorescences and stems, and has been subject to long-term domestication and artificial selection^2^. In addition to its phenotypic diversity and important nutritional value, *B*. *oleracea* is also a model species for studying the evolution of polyploidy because it has experienced multiple rounds of whole-genome duplication (WGD) events^3,4^ and one whole-genome triplication (WGT) event ~15.9 million years ago (mya)^5,6^. Furthermore, it is one of three basic diploid species in the classical ‘U’s triangle’, and two allotetraploid species, *B*. *napus* (AACC) and *B*. *carinata* (BBCC), have recently been formed by hybridization between *B*. *oleracea* and *B*. *rapa* (AA) or *B*. *nigra* (BB), respectively. Therefore, research on *B*. *oleracea* will help us to further understand the speciation and evolution of Brassica and facilitate crop improvements.

Transposable elements (TEs) are widespread in almost all eukaryotes and usually occupy a substantial fraction of the genome^7^. Approximately 40% of the genome is estimated to consist of TEs in *B*. *oleracea*^8,9^. TEs are a powerful driver of genome evolution in Brassica^10–13^. The comparison of nucleotide substitution rates demonstrated the evolutionary asymmetry of the *B*. *oleracea* and *B*. *rapa* genomes and indicated that TEs are likely be responsible for this progress^10^. Moreover, recent studies have revealed that the biased distribution of TEs among the three subgenomes generated through WGT events in *Brassica* contributes greatly to the formation and maintenance of the subgenome dominance phenomenon^11,12^. TEs frequently interfere with gene function and provide a potential source of variants associated with phenotypic changes. In cauliflower, the insertion of a *Harbinger* DNA transposon in the *Pr-D* gene regulatory region increased the expression of the gene responsible for producing the purple phenotype^14^. The presence of a *Helitron* transposon in the promoter of the *BnSP11*-1 gene resulted in the mating system transition from outcrossing to self-fertilizing in *B*. *napus*^15^.

Most TEs have lost mobility because of mutation accumulation or epigenetic modification^16^, though a few still present transposition activity^17,18^. Active TEs produce abundant polymorphic loci, which are probably involved in adaptive evolution and differentiation between populations. For instance, 13 putatively adaptive TEs have been shown to be involved in adaptation to the temperate climate after the migration of *D. melanogaster* out of Africa^19^. In pearl millet, the MITE transposon *Tuareg* located in the 3′ untranslated region (UTR) of *Teosinte branched1* (*PgTb1*), which may be responsible for branching evolution during domestication, was shown to be nearly absent in the wild population, whereas a strong longitudinal frequency cline was observed in the domesticated populations^20^. A variety of sequencing strategies and bioinformatic algorithms have been developed to efficiently identify TE loci based on next-generation sequencing (NGS) technology, and a few comprehensive genome-wide profiles of TE insertion polymorphisms (TIPs) have been constructed in several species^21–26^. These profiles have been successfully applied for the characterization of TE families, population genetic studies, and the discovery of potential selection signatures, giving us a better understanding of the nature of TE families and their contributions to genome evolution at the population level in humans and animals, but they are still unclear in plants.

Previous studies have shown that TEs appear to be more actively amplified in *B*. *oleracea* than in *B*. *rapa*, which diverged from each other by ~4.6 mya^8,27^. They are partially responsible for the expansion of the *B. oleracea* genome relative to the genomes of closely related species^8,27,28^, and have important roles in shaping current genome structure and evolution^10–12^. Nevertheless, little is known about the population dynamics and evolutionary activity of TEs in *B*. *oleracea* at the population level. In this study, we systematically identified TE loci of all types from the *B*. *oleracea* population and performed genotyping at each locus in the individual genome by analyzing the resequencing data of 121 *B*. *oleracea* accessions representing almost all morphotypes. A total of 45,737 unique TE loci were obtained, among which 32,195 showed polymorphic patterns among accessions. The analysis of the population frequency of TEs revealed distinct evolutionary dynamics between different TE superfamilies/families, and a large number of TE insertions with extreme frequency heterogeneity between different morphotypes were identified. These findings not only provide a deeper understanding of the characteristics of TEs and their contribution to genome evolution but also provide valuable resources for studying the evolution, domestication, and breeding of *B*. *oleracea* or related species.

## Results

### Identification and characterization of TEs in *B. oleracea*

A total of 762 Gb of NGS data from 121 diverse *B*. *oleracea* accessions were downloaded from the NCBI SRA^8,9,29^; these accessions represented nine morphotypes with extremely diverse geographical origins (Supplementary Table S1). A library of 3750 nonredundant TE end sequences was constructed from 13,382 TEs representing almost all types of TEs^10^. The NGS reads were first used for searching against the TE end sequence library with Bowtie2 to identify TE-junction reads, which were then mapped to the line 02-12 reference, followed by the removal of redundancy (Supplementary Fig. S1 and “Methods” section). As a result, a total of 62,051 nonredundant unique TE loci were identified from 121 *B*. *oleracea* accessions. Subsequently, the NGS reads of each accession were compared against the flanking sequence library to detect chimeric reads spanning breakpoints; the unmatched portions of these reads were aligned to the corresponding TEs to determine the presence or absence of TE insertions in individual accessions (Supplementary Fig. S1 and “Methods” section). After filtering low-quality and redundant loci, 45,737 TE loci were finally obtained (Supplementary Fig. S2).

**Fig. 1:**
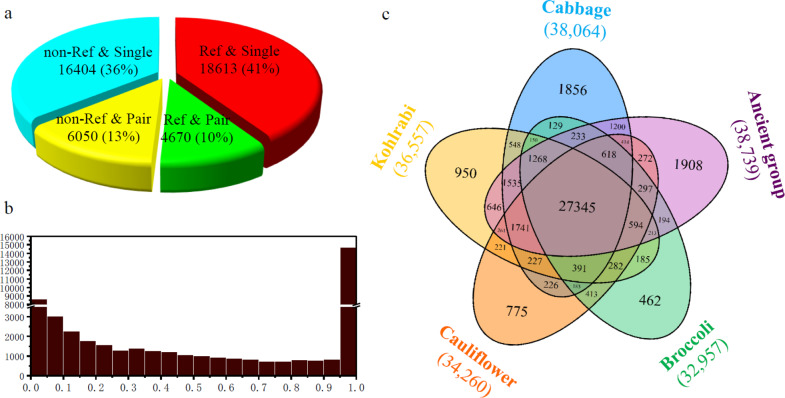
The distribution of TE loci in *B. oleracea* accessions. **a** The number of TE loci present in or absent from the reference genome. Ref: present in the line 02-12 reference genome; non-Ref: absent from the reference genome; Single: insertion was predicted at one end of the TE locus; Pair: insertion was predicted at both ends of the TE locus. **b** The insertion frequencies of 45,737 TE loci in the *B. oleracea* population. **c** Venn diagram showing group-specific and shared TE loci among different morphotypes. The total number of TE loci in each morphotype is indicated in parentheses

Among these loci, DNA transposons (31,363) were approximately twice as abundant as retrotransposons (14,374) (Table 1), which was consistent with the previous analysis of the reference genome assembly^8^. However, half of the TE loci (22,454) were newly identified from resequenced accessions (Fig. 1a). The *Helitron* superfamily exhibited the highest copy number (10,746), followed by LTR-RT/*Copia* (8256), TIR/*Pong* (6897), and TIR/*CACTA* (4850) superfamily, while non-LTR-RTs (SINEs and LINEs) showed relatively low abundance and harbored 2063 and 623 copies, respectively (Table 1). These results showed statistically significant positive correlations with those from the reference genome (*r* = 0.84, *p*-value < 0.001, Pearson).

**Table 1:** The classification of 45,737 TE loci from the *B. oleracea* population

Class	Order	Superfamily	No. of loci
DNA transposon			31,363
	TIR		20,617
		*Pong*	6897
		*CACTA*	4850
		*Tc1/Mariner*	4219
		*hAT*	1719
		*Mutator*	1623
		*PIF/Harbinger*	1309
	Helitron		10,746
		*Helitron*	10,746
Retro transposon			14,374
	LTR		11,688
		*Copia*	8256
		*Gypsy*	2271
		*Unclassified*	1161
	SINE		2063
		*tRNA*	192
		*Unclassified*	1871
	LINE		623
		*L1*	623

The population frequencies of TE loci were further measured (Fig. 1b). Forty percent of these loci exhibited a low frequency (18,575 with a ≤30% population frequency), suggesting that these TEs could have been recently inserted; 30% of the loci were fixed (13,542 with a 100% population frequency), indicating that they were inserted before the divergence of morphotypes from their progenitor; the remaining 30% presented a median or high frequency (population frequency of 31–70% for 8591 or 71–99% for 5029) and showed abundant insertion polymorphisms among accessions. Moreover, a strongly negative correlation was observed between the number of TE loci and the population frequency (*r* = −0.81, *p*-value < 2.2e−16, Pearson).

To determine whether TE insertion potentially affects genes, we analyzed the distribution of TE loci relative to the annotated genes (Table 2). Among the 45,737 TE loci, 542 (1.2%) and 2393 (5.2%) were inserted into the coding regions and introns of genes, respectively, which may disrupt gene function through insertion mutagenesis or altering splicing patterns. A total of 4880 (10.7%) and 3830 (8.4%) loci were found within the 500-bp upstream and downstream sequences adjacent to the annotated genes, respectively. These insertions were likely to change gene expression by providing putative *cis*-regulatory elements or epigenetic regulation. Furthermore, it was observed that as the population frequency of TE insertions increased, the proportion of gene-related TE loci decreased. A total of 30.9% of 18,575 low-frequency loci were located within or near annotated genes, while this proportion decreased to 17.8% for the fixed type (Table 2). This may occur because TE loci with higher frequency, especially those associated with genes, have undergone a longer period of purifying selection and been eliminated from the genome.

**Table 2:** Statistics of TE loci associated with annotated genes

Type	CDS	Intron	Upstream	Downstream	Total
Low (18575)	422 (2.3%)	1247 (6.7%)	2306 (12.4%)	1766 (9.5%)	5741 (30.9%)
Median (8591)	80 (0.9%)	429 (5.0%)	976 (11.4%)	813 (9.5%)	2298 (26.7%)
High (5029)	16 (0.3%)	207 (4.1%)	553 (11.0%)	416 (8.3%)	1192 (23.7%)
Fix (13542)	24 (0.2%)	510 (3.8%)	1045 (7.7%)	835 (6.2%)	2414 (17.8%)
Total	542 (1.2%)	2393 (5.2%)	4880 (10.7%)	3830 (8.4%)	11645 (25.5%)

The numbers in parentheses in the first column represented the total number of TE loci contained within the corresponding frequency type in our data set, whereas the percentages in other columns were calculated as the ratio of the corresponding number to the total number of TE loci contained within the corresponding frequency type

As a result of WGT^8^, the *B*. *oleracea* genome could be divided into three subgenomes: LF (least fractionated), MF1 (medium fractionated), and MF2 (most fractionated). Among 45,737 TE loci, 39,926 (87%) were assigned to the three subgenomes. As expected, the LF subgenome harbored more TE loci (17,402) than MF1 (12,634 loci) and MF2 (9890 loci) (Table 3). Nevertheless, when averaged by subgenome size, there was no significant difference in the density of TE loci among the three subgenomes, ranging from 98 to 101 loci/Mb. For each subgenome, we further analyzed the distribution of TE loci relative to the annotated genes (Table 3). As a result, 6.3–6.6% of all TE loci in the individual subgenomes were found to be inserted into the coding regions or introns of genes, whereas 18.6–20.1% of loci were found within the 500-bp upstream and downstream sequences adjacent to the annotated genes. For each type, the proportion of TE loci showed very small variations among the three subgenomes. Therefore, TEs presented similar distributions among the three subgenomes.

**Table 3:** Distribution of TE loci among the three subgenomes of *B. oleracea*

Type	LF	MF1	MF2
CDS	221 (1.3%)	143 (1.1%)	112 (1.1%)
Intron	924 (5.3%)	693 (5.5%)	518 (5.2%)
Upstream	1923 (11.1%)	1317 (10.4%)	1139 (11.5%)
Downstream	1508 (8.7%)	1034 (8.2%)	849 (8.6%)
Intergenic region	12,826 (73.7%)	9447 (74.8%)	7272 (73.5%)
Total number	17,402	12,634	9890
Genome size (Mb)	178	129	98
TE density (loci/Mb)	98	98	101

The percentage in parentheses was calculated as the ratio of the corresponding number to the total number of TE loci in the subgenome

In addition, the distribution of TE loci was investigated among *B*. *oleracea* morphotypes. To eliminate the interference of small samples, four morphotypes (46 cabbages, 19 kohlrabis, 20 cauliflowers, and 23 broccolis) were considered as four groups, and the remaining five were grouped as a single group (Fig. 1c). In total, 27,345 (59.8%) TE loci were shared by all groups, while 5951 (13.0%) were only present in one group (1856 from cabbages, 950 from kohlrabis, 775 from cauliflowers, 462 from broccolis, and 1908 from others), indicating recent transposition activity of TEs during *B*. *oleracea* domestication and breeding (Fig. 1c). Moreover, 2501 TE loci were unique to the single accession (Table 4). Among the four large morphotypes, the kohlrabi group presented the largest average number of singletons per accession, at 21.6, while the broccoli group presented the lowest, at 3.8 singletons per accession, suggesting lower activity of TEs in broccoli genomes (Table 4).

**Table 4:** The number of singleton TE insertions in each *B. oleracea* morphotype

Morphotype	No. of accession	No. of singleton	Average
Cabbage	46	704	15.3
Kohlrabi	19	411	21.6
Cauliflower	20	367	18.4
Broccoli	23	87	3.8
Others	13	932	71.7
Total	121	2501	20.7

### The evolutionary history of TE activity

**Fig. 2:**
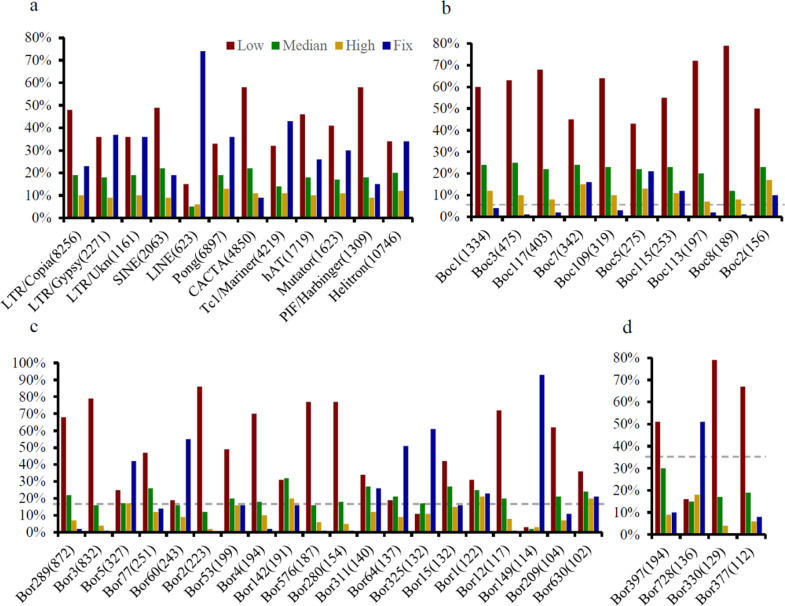
Distribution of the population frequencies of TE loci by superfamily and family. **a** Distribution of the population frequencies of TE loci by superfamily. According to their population frequency, TE loci from the same superfamily were classified into four types: low (0–30%), median (31–70%), high (71–99%), and fixed (100%). **b**–**d** Distribution of the population frequencies of TE loci for families within the *CACTA* (**b**), *Copia* (**c**), and *Gypsy* (**d**) superfamilies. Only highly abundant TE families (>100 copies) are shown. The number of TE loci contained within each superfamily/family is shown in parentheses. The gray dotted lines represent the average proportion of fixed TEs in the corresponding superfamilies

In *B*. *oleracea*, a large number of TE loci showed abundant insertion polymorphisms as described above, suggesting the potential for ongoing transposition activity. Given that recently activated TEs tend to segregate at lower frequencies than older TEs, the population frequency of TE loci may roughly reflect their activity history^30^. Here, TE loci from each superfamily were classified into four types: low (0–30%), median (31–70%), high (71–99%), and fixed (100%) according to their population frequency. The high-frequency type presented the lowest proportion of TE loci, ranging from 6 to 13%, while the low-frequency and fixed TE loci usually accounted for the highest proportion and exhibited a broad range (15–58% for the low-frequency type and 9–74% for the fixed type) (Fig. 2a; Supplementary Table S2). The relative proportions of each frequency type varied considerably across TE superfamilies (*χ*^2^ = 148.93, df = 33, *p*-value < 2.2e−16), reflecting diverse evolutionary histories. In particular, LINEs and *CACTA* exhibited the highest (74%) and lowest (9%) proportions of fixed insertions, respectively, and showed two completely contrasting patterns of transposition activity, where the peak activity of LINEs predated the domestication of *B*. *oleracea*, while *CACTA* arose after domestication. In addition to *CACTA*, in four other superfamilies (*Copia*, SINE, *hAT*, and *PIF/Harbinger*), almost half of the loci were classified into the low-frequency group, implying that they were more actively amplified during recent evolution.

Furthermore, 21 of 34 highly abundant TE families (>100 copies) within three representative superfamilies, *Copia*, *Gypsy*, and *CACTA*, exhibited fewer fixed insertions than were expected and showed similar distribution patterns of the insertion frequency (Fig. 2b–d; Supplementary Table S2), indicating that most TE insertions in these families were derived from recent transposition activity. Conversely, the remaining 13 TE families contained more fixed insertions but fewer polymorphic loci, suggesting distinct expansion patterns to some extent (Fig. 2b–d; Supplementary Table S2). Taken together, these results revealed that the evolutionary dynamics of TEs were highly variable in different TE superfamilies/families, and most highly abundant families appeared to be activated recently.

### Morphotype-differential fixed TE insertions

**Fig. 3:**
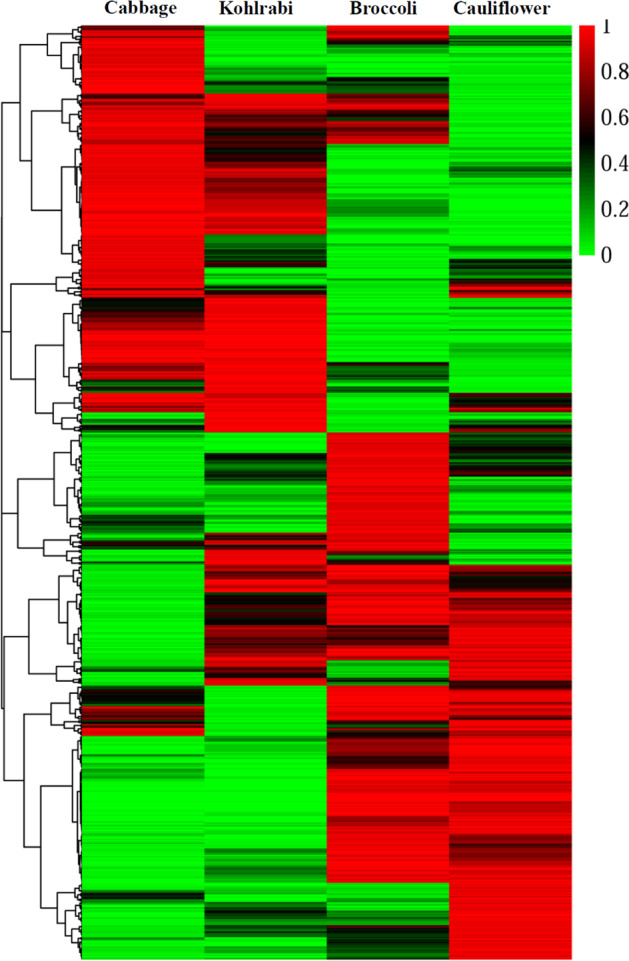
Heatmap of the population frequency of 1221 morphotype-differential fixed TE loci in four large morphotypes. The population frequency was expressed as 0-1, where 1 represented a fixed locus and 0 represented no TE insertion

TE insertions showed substantial variability between populations due to differential proliferation and natural selection, which contribute significantly to speciation and adaptive evolution in species^19,26^. In light of the importance of TE loci showing frequency heterogeneity among different populations, the insertion frequencies of 29,694 polymorphic TE loci were computed for four large morphotypes (Supplementary Fig. S2). Approximately one-third of loci (9127) showed a large difference (>0.5) in allele frequencies between morphotypes (Supplementary Table S3). These loci were further filtered for morphotype-differential fixed insertions, which were defined as being present in at least 90% of accessions of one morphotype but absent from at least 90% of accessions of another. As a result, up to 1221 morphotype-differential fixed TE loci were identified in four morphotypes (Fig. 3; Supplementary Table S3). There were many more differentially fixed loci between cabbage and cauliflower/broccoli than between cabbage and kohlrabi (Table 5). Similarly, many more differential fixed loci were observed between broccoli and cabbage/kohlrabi than between broccoli and cauliflower. There was a strongly positive correlation between the number of differential fixed loci and the level of morphotype differentiation (*F*_ST_) (*r* = 0.97, *p*-value < 0.001, Pearson; Supplementary Fig. S3).

**Table 5:** The number of morphotype-differential fixed TE loci between four different groups

Combination	Morphotype 1	Morphotype 2	*F*_ST_
Cabbage vs Broccoli	Cabbage (228)	Broccoli (277)	0.39
Cabbage vs Cauliflower	Cabbage (238)	Cauliflower (257)	0.40
Cabbage vs Kohlrabi	Cabbage (62)	Kohlrabi (53)	0.27
Broccoli vs Cauliflower	Broccoli (87)	Cauliflower (51)	0.29
Broccoli vs Kohlrabi	Broccoli (188)	Kohlrabi (127)	0.34
Cauliflower vs Kohlrabi	Cauliflower (150)	Kohlrabi (146)	0.36

The number of morphotype-differential fixed TE loci contained within each morphotype is shown in parentheses compared with the other morphotypes

### TE variations reflect evolutionary relationships among morphotypes

**Fig. 4:**
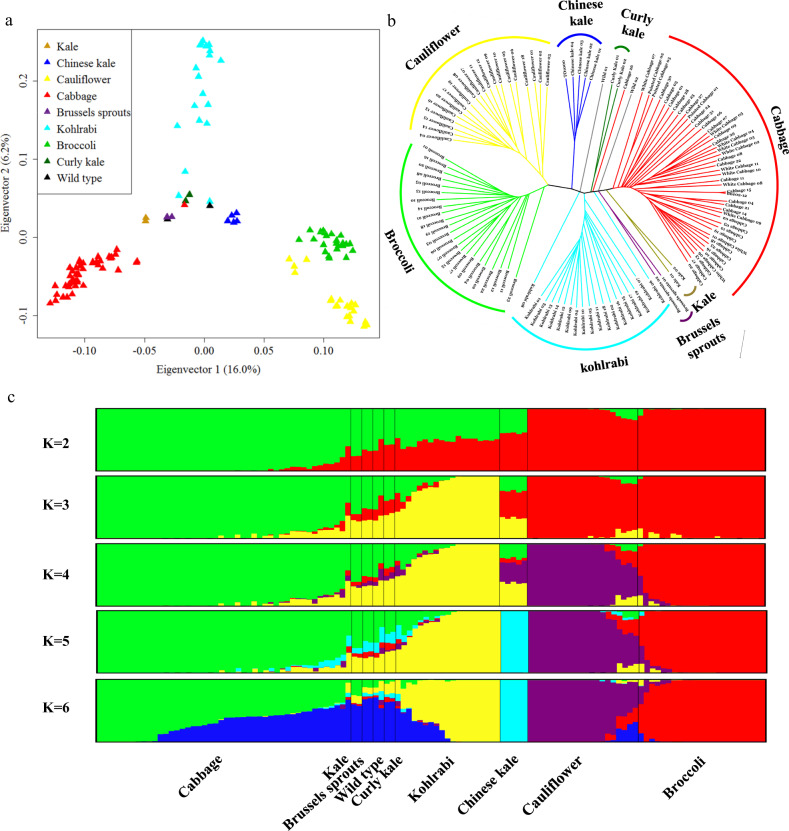
The evolutionary relationships of *B. oleracea* accessions based on insertion polymorphisms at TE loci. **a** The principal component analysis (PCA) of 121 accessions using 13,181 polymorphic TE loci with proper population frequencies (0.2 ≤ TE _Freq_ ≤ 0.8). The plot shows the first two principal components. The phylogenetic tree (**b**) and population structure (**c**) of 121 accessions were inferred from the same set of polymorphic TE loci. The NJ method was used to construct the phylogenetic tree with PAUP* 4.0b10^48^. The population structure was inferred by using STRUCTURE (version 2.3.3)^49^. *K*-values of 2–15 were analyzed, and only *K* = 2–6 are shown. Three replicates were carried out for each *K*-value

The majority of TE loci showed a high level of insertion polymorphism among accessions, which provided a good opportunity for systematically evaluating their application as molecular markers in genetic studies. PCA, phylogenetic analysis, and population structure analysis were performed based on 13,181 polymorphic TE loci with proper population frequencies (0.2 ≤ TE_freq_ ≤ 0.8). The first two principal components of the PCA explained 16.0% and 6.2%, respectively, of the overall genetic variance in the population (Fig. 4a), and clearly separated the vast majority of morphotypes from each other, where cabbage, kohlrabi, cauliflower, and broccoli represented the three poles of *B. oleracea* genomic variation, while the remainder were clustered in the middle, showing more ancient morphotypes. This subdivision was supported by the phylogenetic analysis using the same TE locus data set (Fig. 4b). In the NJ tree, almost all accessions were grouped together according to their respective morphotypes, and the evolutionary relationships between morphotypes were reconstructed properly. Compared with the phylogenetic tree based on single-nucleotide polymorphisms (SNPs)^31^, one obvious difference was that cauliflower and broccoli were clearly separated from each other in this tree, while broccoli formed a subbranch of cauliflower in the SNP-based phylogenetic tree. Nevertheless, the evolutionary relationships among morphotypes showed almost perfect consistency between the two trees overall.

Using the Bayesian model-based clustering method implemented in STRUCTURE, these accessions were again segregated into different groups, reflecting their morphotype distribution, with the number of hypothetical subpopulations (*K*) changing progressively from 2 to 6 (Fig. 4c). The optimal number of subpopulations was *K* = 4 (Supplementary Fig. S4), where four morphotypes (cabbage, kohlrabi, cauliflower, and broccoli) formed distinct clusters. The structure pattern was also highly consistent with the PCA and phylogenetic analysis (Fig. 4a, b). Overall, the genetic variations introduced by TE insertions showed the ability to reliably reveal evolutionary relationships among morphotypes, indicating that these polymorphic TE loci constitute a good source of molecular markers for genetic studies and breeding programs in *B. oleracea* and other *Brassica* species.

## Discussion

### A comprehensive mobilome profile for B. oleracea

TEs account for a large proportion of the *B. oleracea* genome; however, most of the work on this topic has focused on single families or a few families^32–35^ or has been conducted only within reference genomes^8,10,11^. This limits the comprehensive understanding and application of TEs in *B. oleracea*. Here, we systematically identified potential insertions of all TE types in the *B. oleracea* population and performed genotyping at each locus in the individual genome by analyzing NGS reads. A comprehensive mobilome profile was first constructed for *B. oleracea*, which represented the most extensive study of TE variations in the species and allowed us to obtain a deeper understanding of the characteristics of TEs and their contribution to genome evolution.

A considerable difference in the mobilome was observed between *B. oleracea* and its close relative *Arabidopsis thaliana*^36^, indicating that TEs underwent differential amplification between the two species after their divergence from a common ancestor at ~20 mya (Supplementary Fig. S5). *Helitron* transposons represented the most prevalent superfamily, contributing the greatest number of polymorphic loci to the genome in both species, but they accounted for an obviously lower proportion of the loci in *B. oleracea* (22.0%) than in the *Arabidopsis* genome (29.6%) (Supplementary Fig. S5). In addition, the proportions of six TE superfamilies (*Copia*, SINE, *CACTA*, *Tc1/Mariner*, *PIF/Harbinger*, and *Pong*) were higher in *B. oleracea* than in *Arabidopsis*, indicating that they were much more actively amplified in *B. oleracea* after divergence from their progenitor. Four of them (*Copia*, SINE, *CACTA*, and *PIF/Harbinger*) exhibited higher transposition activity during recent evolution, as almost half of the loci showed low-frequency insertions (<30%) in the *B. oleracea* population (Fig. 2). However, four TE superfamilies (*Gypsy*, LINE, *Mutator*, and *hAT*) were less abundant in *B. oleracea* than in *Arabidopsis* (Supplementary Fig. S5). In particular, there was at least a 15% difference between these two species for *Mutator* and *Gypsy* TEs. The mobilization of the *Hi Mutator*-like element in *Arabidopsis* is facilitated by an anti-silencing protein encoded by its genome, which is widespread in non-TIR *MULEs* and has contributed to recent success-related TEs in *Arabidopsis*^37^. In *Arabidopsis*, high-frequency insertions (71–99%) accounted for 40% of the total *Gypsy* loci, whereas they accounted for only 14% of the loci in *B. oleracea* (Supplementary Fig. S6 and Supplementary Table S4), suggesting that the difference was partially attributed to much older transposition in *Arabidopsis*, which occurred soon after the two species split. Overall, high-frequency insertions contributed a significantly larger proportion of the loci in *Arabidopsis* (27.7%) than in *B. oleracea* (15.6%), reflecting earlier activity of TEs in the former (Supplementary Table S3).

### Differential TE abundance among morphotypes

The distribution of TE loci among morphotypes revealed that the total number of loci, group-specific loci and singletons varied extensively across morphotypes (Fig. 1c and Table 4). It is noteworthy that the number of TE loci was underestimated because only unique insertions were identified in the genomes. Cabbage and broccoli possessed the largest and smallest number of TE loci, respectively, for the three types of insertions among the four large morphotypes, which was consistent with a previous study on SNP and indel variations^29^. There were still significant differences in the number of singletons per accession (5 times) and group-specific loci (1.7 times) between cauliflower and broccoli (Fig. 1c and Table 4), even though they were similar to each other. These results implied that a differential TE activity arose after the morphotype split. The differential proliferation of TEs among morphotypes could result from the epigenetic regulation of DNA methylation modifications^37,38^. The annual temperature range has been shown to contribute to the variation in *ATCOPIA78* mobilization in the *Arabidopsis* population^39^. The origination and domestication of diverse morphotypes occurred in different European regions^40^; therefore, environmental factors may also be a potential contributor to differential transposition activity.

### Evolutionary dynamics of TEs in *B. oleracea*

The population frequency analysis showed that TEs were preferentially responsible for rare or fixed insertions (Fig. 1b), similar to a previous study in *Drosophila melanogaster*^22^. The overrepresentation of rare loci mainly resulted from independent proliferation after divergence, while the fixed loci may be due to the accumulation of long-term amplification before domestication. There was a significant difference in the distribution pattern of the population frequency among TE superfamilies (Fig. 2a), suggesting diverse activity histories. In particular, LINE and *CACTA* represented two entirely contrasting patterns of amplification. LINEs have been shown to present very low copy numbers in the *B. oleracea* genome on the basis of BAC library screening and BLASTN analysis and to exhibit a high level of sequence divergence^35^. Moreover, LINEs are intermingled in most lineages (15/18) from *Arabidopsis* and *B. oleracea*^28^. In our data set, LINEs exhibited the lowest copy number (623) but the highest percentage of fixed insertions (74%) in the *B. oleracea* population (Fig. 2a). These results implied that LINEs have been nearly silenced for a long time in this species. In contrast, *CACTA* has been amplified to very high copy numbers and possesses high intrafamily sequence identity^28^. As many as 4850 *CACTA* loci were identified from the *B. oleracea* population, and only 9% were fixed (Fig. 2a), indicating that *CACTA* has experienced bursts of activity during recent domestication.

Even within orders or superfamilies, different TEs displayed distinct activity dynamics. *Copia* was found to harbor many more loci than *Gypsy*^8,35^. However, *Copia* exhibited fewer fixed insertions than *Gypsy* (Fig. 2a). A similar trend was observed for different families within the same superfamily (Fig. 2b–d). These results revealed that different TEs have behaved very differently during *B. oleracea* genome evolution. Nevertheless, the majority of highly abundant families seemed to benefit from recent transposition activity and were partly responsible for *B. oleracea* genome expansion.

### Morphotype-differential fixed insertions may be shaped by domestication or population genetic factors

A large number of TE loci (9127) showed frequency heterogeneity among *B. oleracea* morphotypes. More importantly, 1221 of these loci were morphotype-differential fixed insertions. These TE loci showing frequency heterogeneity may result from directional selection. It has been well documented that purifying selection governs the distribution of TEs in the population. The insertion of an *Accord* element into the 5′-end of the *Cyp6g1* locus was followed by a selective sweep leading to very high frequencies (85–100%) under the strong selection pressure exerted by insecticides in non-African *D. melanogaster* populations^41^. In maize, a *Hopscotch* retrotransposon inserted into the regulatory region of the *Tb1* gene contributes to increased apical dominance by repressing branch outgrowth and has been shown to be almost fixed in the domesticated population but nearly absent in wild progenitors as a result of a selective sweep^42,43^. *B. oleracea* has always been subjected to severe selective pressure, leading to the formation of a variety of highly specialized organs during its long-term domestication and improvement. Therefore, it is reasonable to infer that some TE loci showing frequency heterogeneity, particularly morphotype-differential fixed insertions, can be attributed to selective sweeps when their insertion results in the desired phenotypic variations or contributes to adaptation to the environment. In this case, these loci offer new opportunities to elucidate the genetic basis of morphotype differentiation as ideal candidates, which have profound consequences for the improvement and precision breeding of Brassica.

In addition, population genetic factors such as hitchhiking effects and genetic drift also present strong potential to shape the distribution of TE loci in the population. Although TEs themselves are not the targets of selection in many cases, the insertions linked to the selected variations are affected by selection and hitchhike to a high frequency, leading to sharp departure from the neutral expectation^44^. Therefore, the frequency heterogeneity of TE loci among morphotypes can be considered promising selection signals for future evolutionary studies. In addition, TEs have been shown to be free to drift toward loss or fixation as a consequence of a population bottleneck, which reduces the efficacy of selection against TEs in the genome^45,46^. We inferred the demographic history of four *B. oleracea* morphotypes based on SNP variations identified in a previous study^29^ and found that they all experienced a similar and strong bottleneck in the period from 20,000 to 2500 years ago (Supplementary Fig. S7). Thus, it is likely that the frequency heterogeneity of TE loci is caused by genetic drift during the period. In the future, more work will be carried out to distinguish the transpositional mechanisms involved and determine whether they may be associated with divergent traits.

## Materials and methods

### Data sources

The NGS data of 121 *B. oleracea* accessions (Supplementary Table S1), including two reference accessions (line 02-12 and TO1000DH), were downloaded from the NCBI Sequence Read Archive (SRA) (accession: SRP071086, SRR1212997, SRR585607–SRR585609)^8,9,29^. This collection represents nine morphotypes of *B*. *oleracea* species, including 46 cabbage (var. *capitata*), 23 broccoli (var. *italica*), 20 cauliflower (var. *botrytis*), 19 kohlrabi (var. *gongylodes*), and 5 Chinese kale (var. *alboglabra*) accessions as well as two accessions each of kale (var. *acephala*), Brussels sprouts (var. *gemmifera*), curly kale (var. *sabellica*), and wild *B*. *oleracea*^8,9,29^. A total of 762 Gb of filtered NGS data with an average 10-fold coverage per accession were obtained. Two assembled *B*. *oleracea* genomes, from lines 02-12 (cabbage) and TO1000DH (Chinese kale), were used as references^8,9^, which were downloaded from Bolbase (http://ocri-genomics.org/bolbase/, line 02-12 version 1.0) and Ensembl Plants (ftp://ftp.ensemblgenomes.org/pub/plants/release-37/fasta/brassica_oleracea/dna/, TO1000DH version 2.1), respectively.

A well-curated set of TE sequences was collected from Bolbase (http://ocri-genomics.org/bolbase/), which was composed of 13,382 TEs with clear boundaries from the line 02-12 reference assembly, including 5107 retrotransposons and 8275 DNA transposons^10^. These TEs represented a broad taxonomic range of long terminal repeat retrotransposons (LTR-RTs) (*Copia*, *Gypsy*, and unclassified), non-LTR-RTs (long interspersed nuclear elements-LINEs and short interspersed nuclear elements-SINEs) and nine DNA transposon superfamilies (*Tc1*/*Mariner*, *hAT*, *Mutator*, *PIF*/*Harbinger*, *Pong*, *CACTA*, MITE/*Stowaway*, MITE/*Tourist*, and *Helitron*)^10^.

### Construction of the TE end sequence library

Before alignment, a local library of TE end sequences was constructed to avoid unnecessary computation as previously described^47^. First, 150 bp sequences from both ends of 13,382 TEs were extracted, and redundancy was then removed using the software Blast+ with the following parameters: “-task blastn -outfmt 6 -evalue 1e-20 -max_target_seqs 100000”. The “-evalue 1e-20” setting ensured that the similarity between the sequences was greater than 90% when an alignment of 75 bp in length (the acceptable minimum length) occurred near the end of TE sequences. Provided that the alignment between two sequences met the above criterion, they were considered to be mutually redundant, and only one of them was retained for subsequent analysis. A total of 3750 nonredundant TE end sequences from *B*. *oleracea* were finally retained and used to construct a local TE end sequence library.

### Identification and genotyping of TE insertions

The process of data analysis mainly included the following two stages for obtaining the mobilome profile of the *B. oleracea* population: in the first stage, the NGS reads were used to identify TE loci in the *B*. *oleracea* population using line 02-12 as the reference; in the second stage, presence/absence calling for each identified TE locus was carried out in 121 diverse accessions (Supplementary Fig. S1 and “Methods” section).

### TE locus filtering

To eliminate unreliable TE loci and accurately determine their population frequency, the initial mobilome profile was further filtered according to the following aspects (Supplementary Fig. S2). First, 2474 TE loci with missing calls in more than half of accessions were excluded, since a high level of missing calls would reduce the accuracy of the population frequency estimation. Second, considering that most of the *B*. *oleracea* accessions (except for six GenBank accessions) were homozygous or almost homozygous, TE loci with excessive heterozygosity were unreliable. Therefore, 515 TE loci showing excessive heterozygosity (>0.41; see Supplementary Methods) were discarded. Third, for TEs that were only inserted in a single accession and did not exhibit sufficient supporting reads (<3 reads), the corresponding loci potentially presented high prediction error rates, and 2605 such TE loci were removed, which resulted in 56,457 TE loci. Furthermore, 10,720 TE loci were predicted from both TE ends derived from the same locus. Finally, we obtained a filtered genome-wide mobilome profile consisting of 45,737 TE loci genotyped in 121 diverse accessions across nine *B*. *oleracea* morphotypes.

### Evaluation of data set quality

With an average sequencing depth of 10-fold, the accessions were likely to lack the corresponding chimeric reads spanning the breakpoints at a particular TE locus. Through a survey of the mobilome profile, we observed that ~85% of 45,737 TE loci were detected in at least 108 (90%) accessions, and the overall detection rate of TE loci was up to 93.7% (Supplementary Fig. S8a), which was close to the average mapping rate (92.4%) of the resequencing data. Moreover, the average number of supporting reads was 5.4 for each call, and 84% of the calls exhibited at least three supporting reads (Supplementary Fig. S8b). These results confirmed that our methods showed high sensitivity and stability.

To verify the accuracy of our data set, we adopted the following three strategies. First, because of the availability of the line 02-12 and TO1000DH references, we could determine whether TE insertion actually occurs at particular loci in these two references by scanning their assembled genome sequences and then comparing them with the NGS results. The consistency between the two datasets was 97.4% in line 02-12 and 97.6% in TO1000DH at 1000 randomly selected loci (Supplementary Table S5), indicating the high accuracy of our methods. Second, 10,720 TE loci were independently predicted from both ends of TE insertions, and nearly perfect concordance (99.7%) was obtained by analyzing the presence and absence of these TE loci in the 121 accessions at both ends, which again indicated that the method was reliable. Third, six TE loci, including four polymorphic (morphotype-differential fixed insertions) and two fixed loci (as control), were selected for the validation of their presence or absence in eight accessions (cabbage: JF-1 and ZG-21; kohlrabi: TJQ and LPL-1; cauliflower: FZ-80 and FZ-60; broccoli: ML and LFS) in PCR experiments. The results showed that the four polymorphic loci exhibited abundant insertion polymorphism among these accessions (Supplementary Fig. S9), and their presence/absence in each morphotype was consistent with the results for NGS reads. In comparison, the two fixed loci showed monomorphism and carried TE insertions in all the accessions (Supplementary Fig. S9). Overall, our data set was shown to be very reliable.

### Phylogenetic analysis and population structure

To facilitate the study of genetic relationships among accessions, we first converted the polymorphism data set of TE insertions into a binary matrix in which “1” and “0” indicate the presence and absence of TE insertions, respectively, whereas “?” represents a missing call at the corresponding locus. A subset of 13,181 polymorphic TE loci with proper population frequencies (0.2 ≤ TE_freq_ ≤ 0.8) was then selected for the following analyses.

First, PAUP* 4.0b10 was used to construct the phylogenetic tree for all accessions based on the binary matrix^48^. The mean model (the mean of pairwise character difference), which can adjust for missing calls in the matrix, was employed to calculate the pairwise distance. Then, the neighbor-joining (NJ) method was applied to construct the tree with the least-squares option. Second, STRUCTURE (version 2.3.3) was applied to infer the genetic structure of populations^49^. During this process, the range of *K*-values, which represented the assumed number of populations, was set to 2–15, and three replicates were carried out for each *K*-value. A burn-in period of 100,000 iterations followed by a run length of 100,000 iterations and the admixture model were set. The DISTRUCT program was then used to graphically display the results. Third, principal component analysis (PCA) was performed to investigate the pattern of genetic differentiation among populations and individuals using the R package SNPRelate with default parameters^50^, which conducted eigen-decomposition of the genetic covariance matrix to compute the eigenvalues and eigenvectors. In addition, to estimate the level of genetic differentiation between different *B. oleracea* morphotypes, the *F*_ST_ values between pairwise morphotypes were calculated based on the 13,181 polymorphic TE loci using the snpgdsFst function in SNPRelate with the W&C84 method.

## Supplementary information

Supplementary materials

## Data Availability

The information for the TE loci, their genotyping results in each accession, and the distribution of TE loci relative to annotated genes have been deposited at GitHub for public access (https://github.com/lanceliu2018/HortRes_BoTE). The analysis pipeline used in this study has been configured in the docker container “zhenliuzju/trip” and uploaded to dockerhub (https://hub.docker.com/r/zhenliuzju/trip).
