# Supplementary material for: Natural variation and evolutionary dynamics of transposable elements in Brassica oleracea based on next-generation sequencing data
Source: Hortic Res. 2020 Sep 1;7:145. doi: 10.1038/s41438-020-00367-0 (PMC7459127; doi:10.1038/s41438-020-00367-0)
Supplement: Supplementary file 1 — Supplementary materials [file 41438_2020_367_MOESM1_ESM.pdf]

Supplementary Methods

Protocol for identification and genotyping of TE loci

In stage one: Firstly, the 150 bp split reads of the resequenced accessions were used as queries to search against TEs ends library by Bowtie2 with the following parameters: "--local -D 20 -R 3 -N 1 -L 20 -i S,1,0.5 --score-min G,0,13 -a". Reads with 30 ~ 130 bp match to the ends of TE sequences in the library were considered as TE-junction reads spanning a TE insertion site, which consisted of TE sequences and flanking sequences, and were selected for further analyses via custom Perl scripts. Whereas, the reads aligned to TEs sequences with full length were removed due to lacking adequate information of flanking sequence for proper mapping. Secondly, in order to determine the genomic location of the insertions, the reads retained in the previous step were trimmed off TE portion and then mapped to the line 02-12 reference genome by Bowtie2 with the following parameters: "-a", then the reads uniquely mapped to reference genome were kept. Thirdly, a non-redundant list of TE loci were obtained by clustering the TE insertion sites according to the information of their genomic location. As a result, we identified a total of 62,051 non-redundant TE loci located in unique genomic sequences from B. *oleracea* population composed of 121 diverse varieties.

For a particular locus, the above analysis merely allowed us to acquire a list of accessions in which TE present. However, the remaining accessions still kept uncertain whether the observed absence of a TE insertion is true or is attributable to a lack of sequencing reads. In addition, the sequence divergence between TE at a particular site and the representative one maybe also miss a small number of accessions carrying TE insertions, which will discount the accuracy of the data. Therefore, it is still needed to further analysis with the following procedures to address precisely these kinds of issues.

In stage two, the 150 bp sequences immediately flanking the mapped TE loci were first extracted from reference genome and grouped into a database. Secondly, the NGS reads of all resequenced accessions were used as queries to search the flanking

sequences database extracted in the last step one by one using bowtie2 with the following parameters: “--local -D 20 -R 3 -N 1 -L 20 -i S,1,0.5 --score-min G,0,13 -a”. Reads with full length alignment to flanking sequences were removed, while chimeric reads for which only part belong to flanking sequences were retained. Thirdly, these chimeric reads were trimmed off matching portion, and the remaining sequence of the reads were aligned to the end sequences of corresponding TEs by Blast+, to decide whether these locations probably possess the expected TE insertions. In this way, we could characterize presence or absence of TE insertions at all identified loci in individual genomes and the initial mobilome profile of B. *oleracea* was obtained.

The calculation of heterozygosity threshold

Since the ratio of the observed heterozygosity (Hobs, the proportion of heterozygous calls in all non-missing calls of a locus) to the expected heterozygosity (Hexp = 2pq, where p and q are the two allele frequencies of a locus) was expected to be distributed around 1-F, where F represented the inbreeding coefficient, we calculated the inbreeding coefficient (F) as the median value of 1- Hobs/Hexp at TE loci where Hobs/Hexp < 1. Then a threshold of max observed heterozygotes (Hobs_max) was obtained by the following equation: Hobs_max = 10 (1-F) Hexp, where a TE locus was considered as including excessive heterozygotes, if Hobs_max was ten times more than the most likely value for given frequency and inbreeding coefficient.

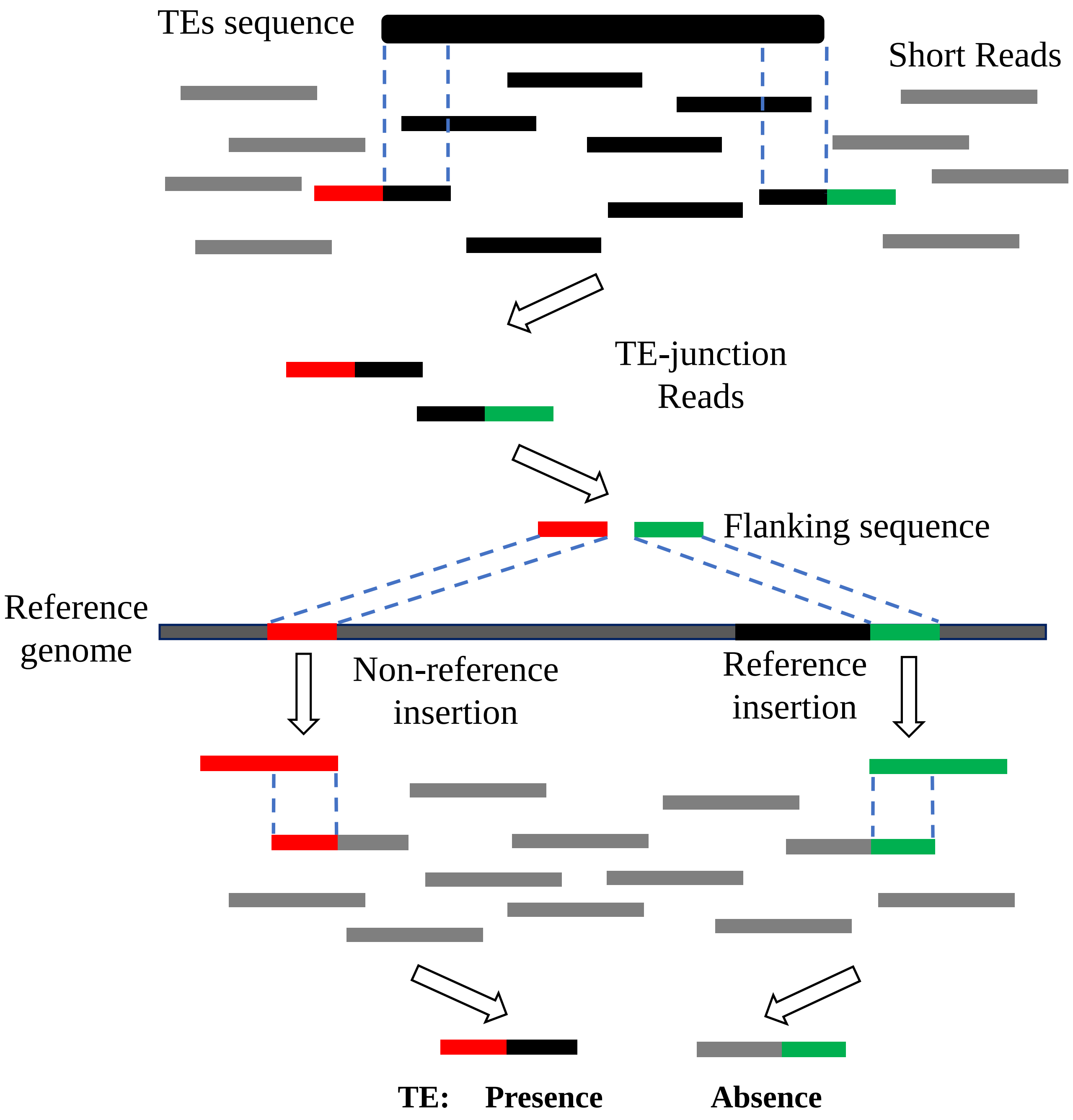

Supplementary Figure 1 The general schematic view of the procedure to identify TE insertion polymorphism.

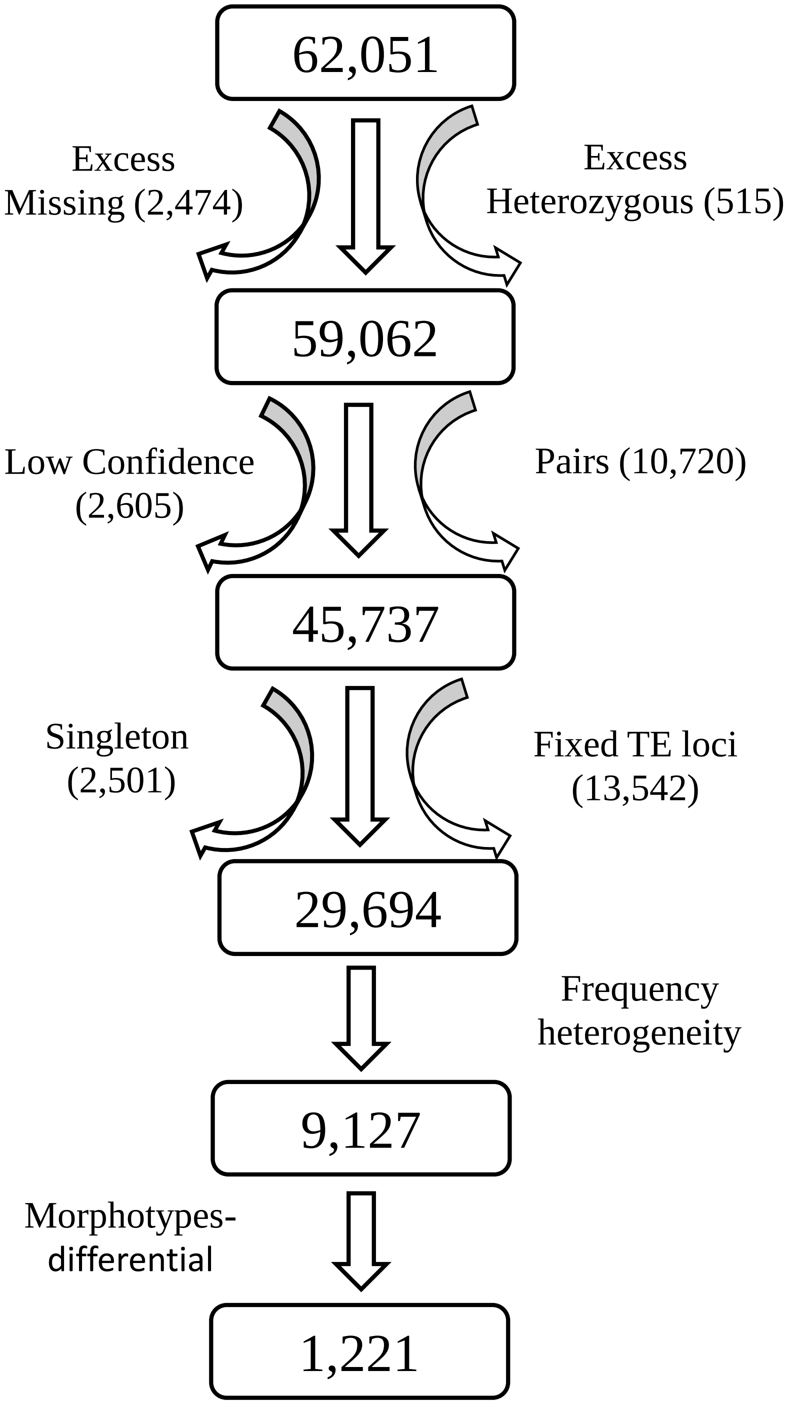

Supplementary Figure 2 The outline of the procedure for the identification of morphotypes-specific insertions.

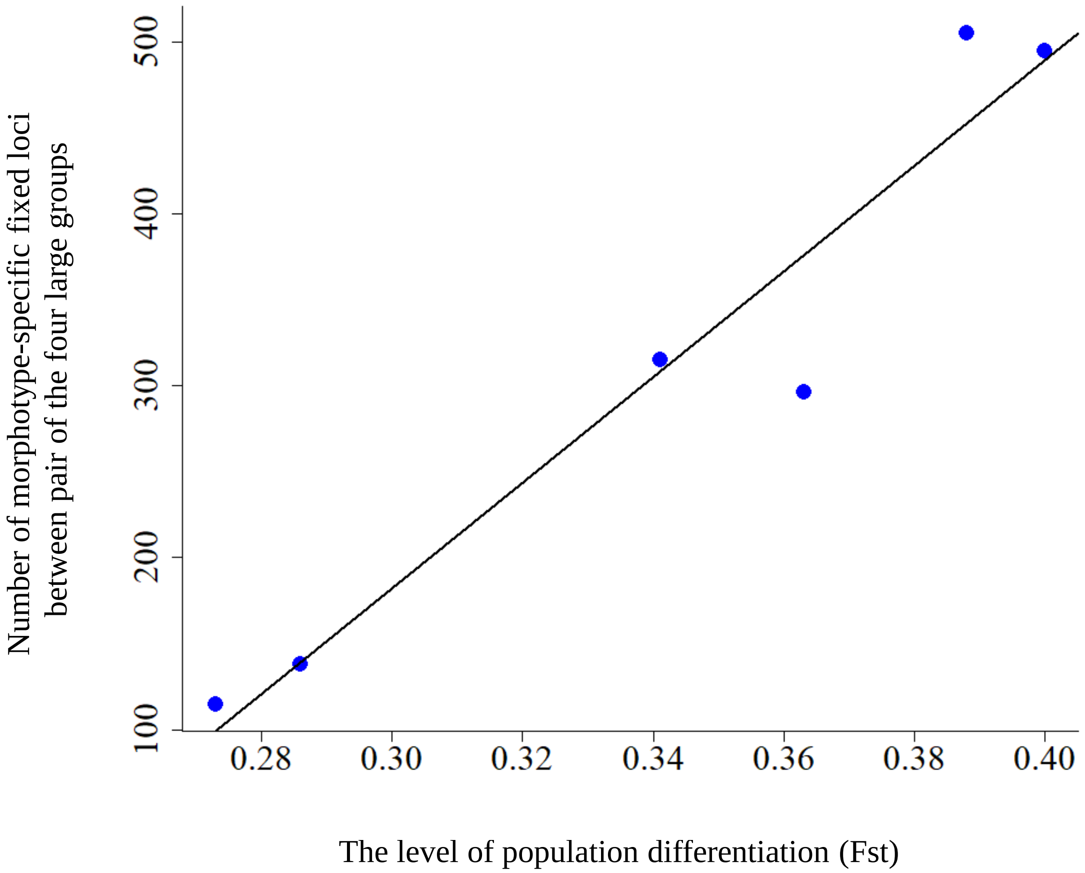

Supplementary Figure 3 The correlation between the number of morphotype-differential fixed loci and the level of population differentiation. The population pairwise estimates of Fst were calculated from the 13,181 polymorphic TE loci in the dataset using R package SNPRelate.

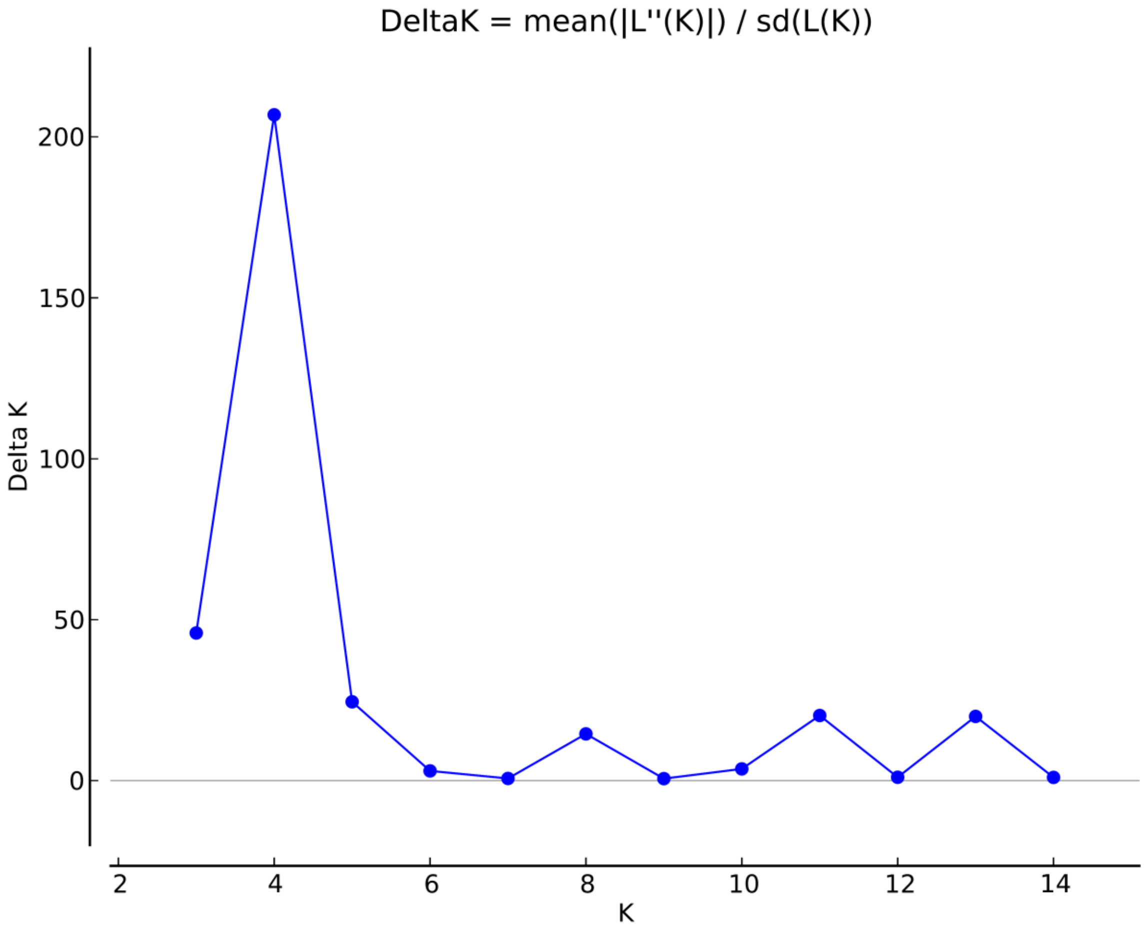

Supplementary Figure 4 The evanno plot of delta-K from STRUCTURE analysis using Structure Harvester. Delta-K was calculated as $\Delta K = m|L''(K)|/s[L(K)]$.

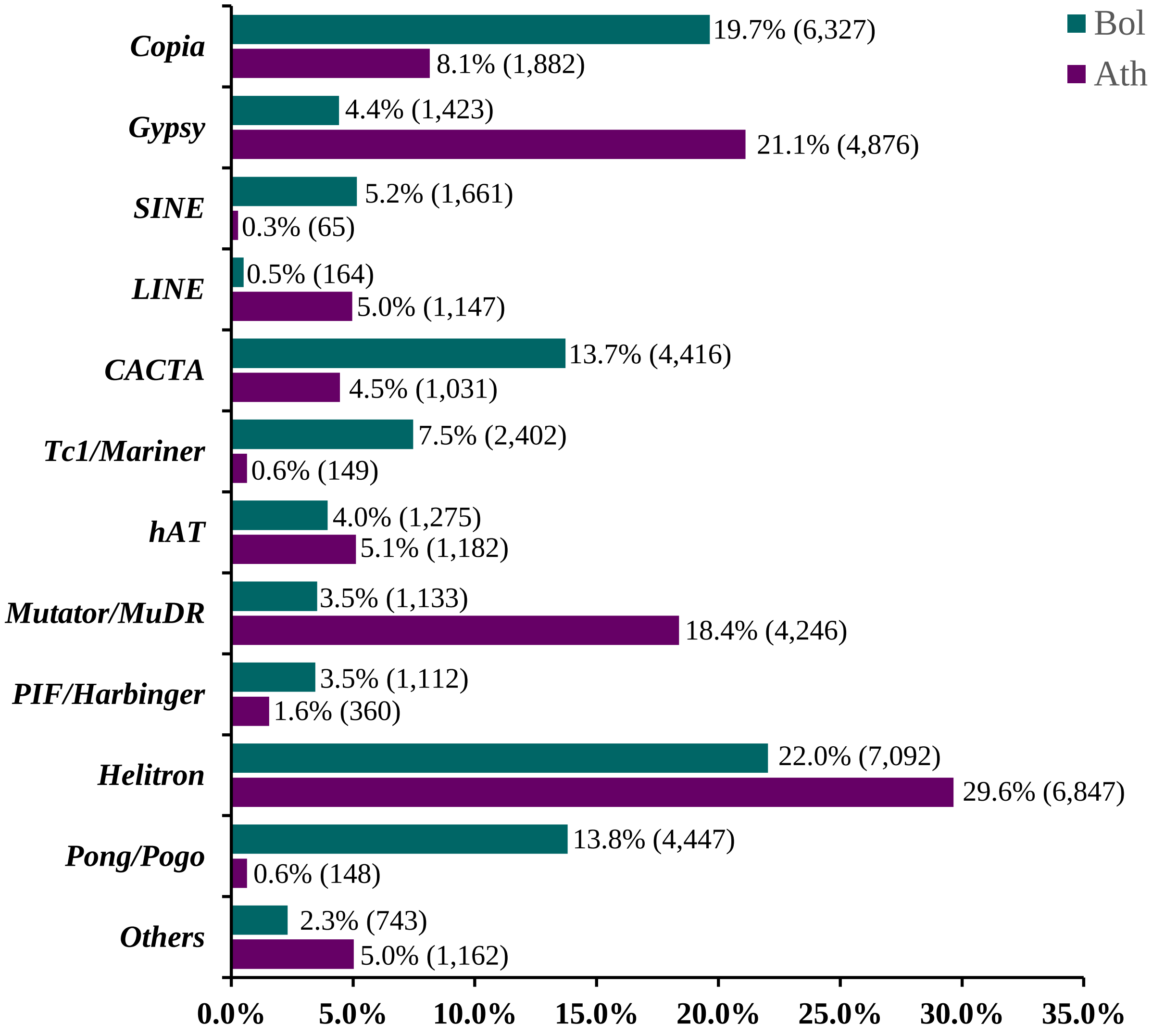

Supplementary Figure 5 The composition of the mobilome profile of *B. oleracea* and Arabidopsis. It is worth noting that this analysis only included 32,195 polymorphic TE loci after excluding fixed insertions from *B. oleracea* and 23,095 polymorphic loci from Arabidopsis.

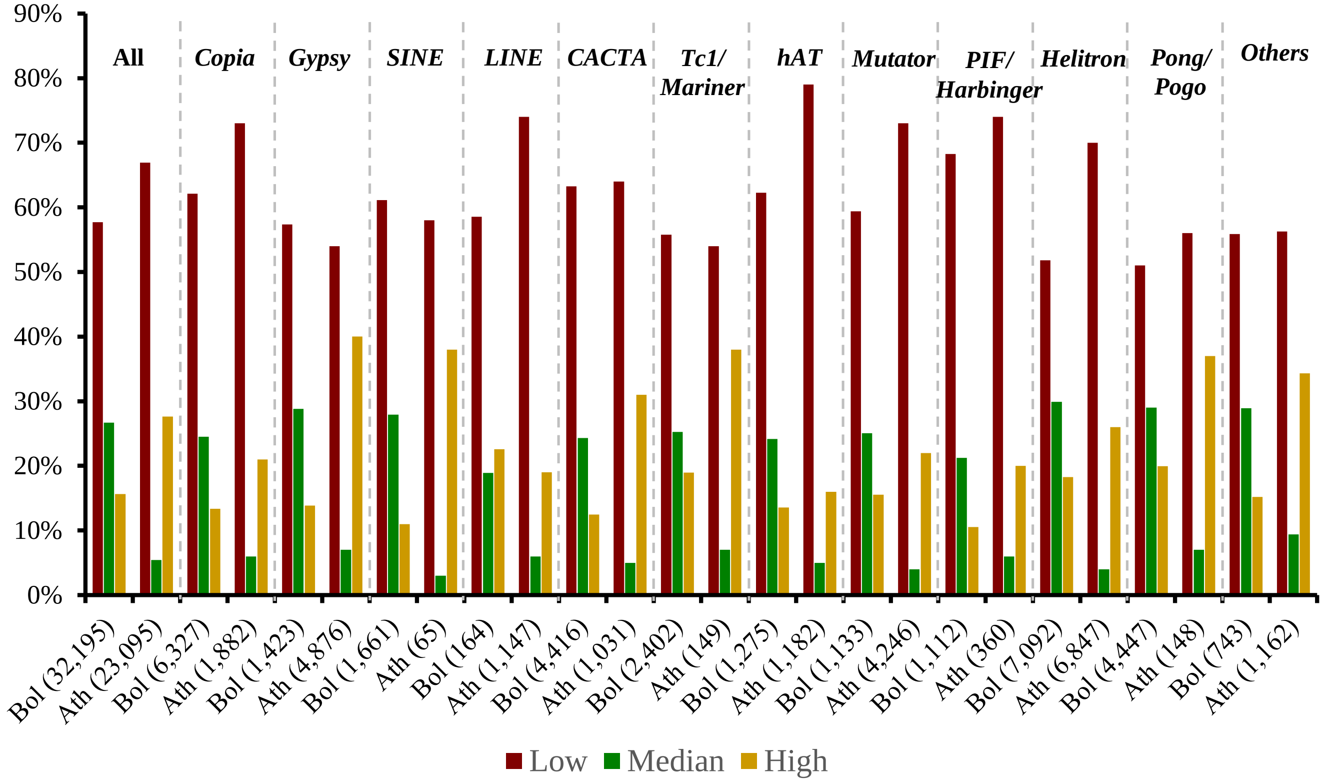

Supplementary Figure 6 The distribution of population frequencies of TE loci by superfamilies in *B. oleracea* and Arabidopsis.

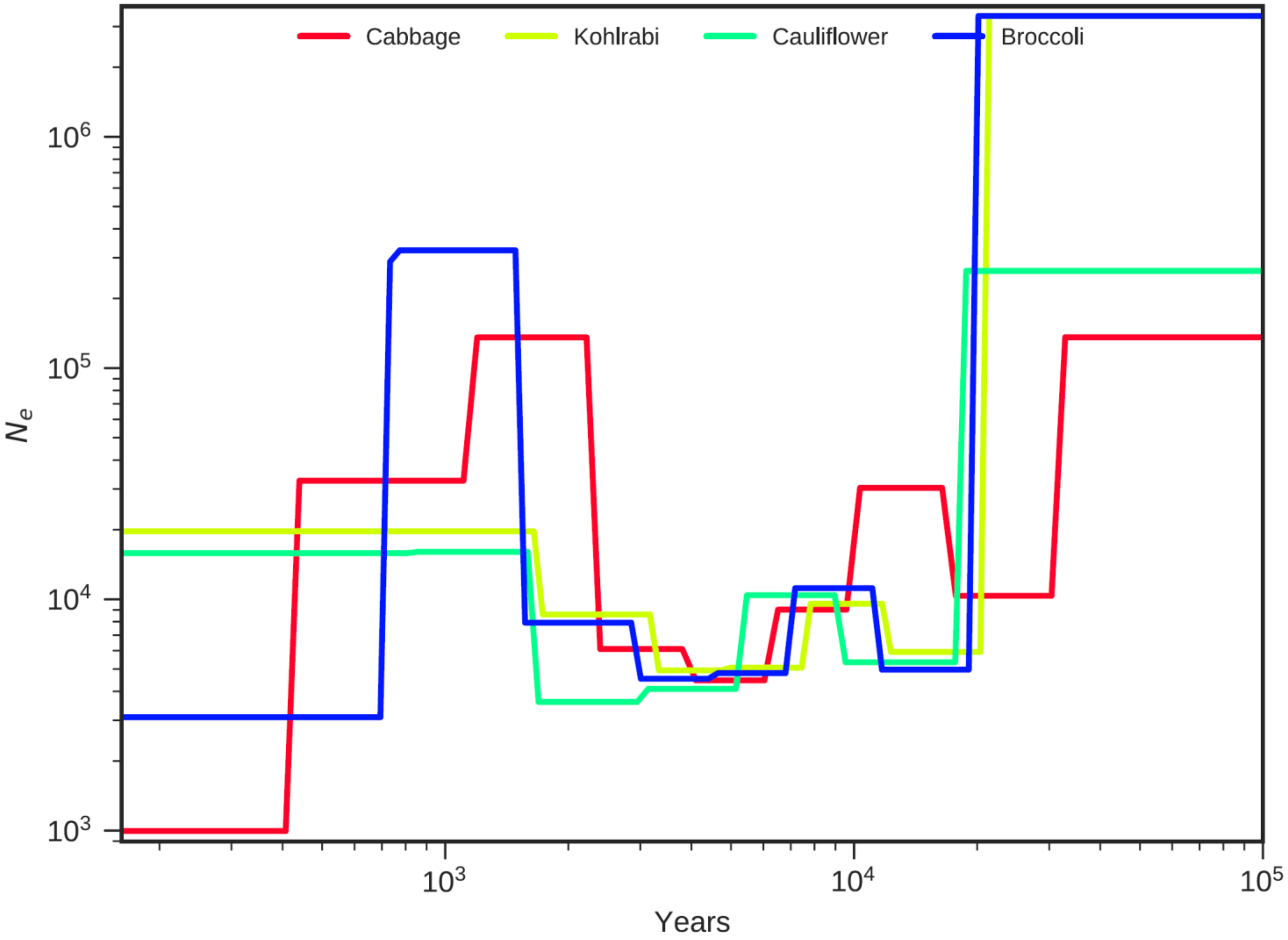

Supplementary Figure 7 The effective population size (*Ne*) histories of the four *B. oleracea* morphotypes inferred from SNP variations using SMC++ (Terhorst *et al*., 2017). The average mutation rate (r) was set to be 1.5×10^{-8} substitutions per site per year (Koch *et al*., 2000), and a generation time of 1 year was used to convert coalescent scaling to calendar time.

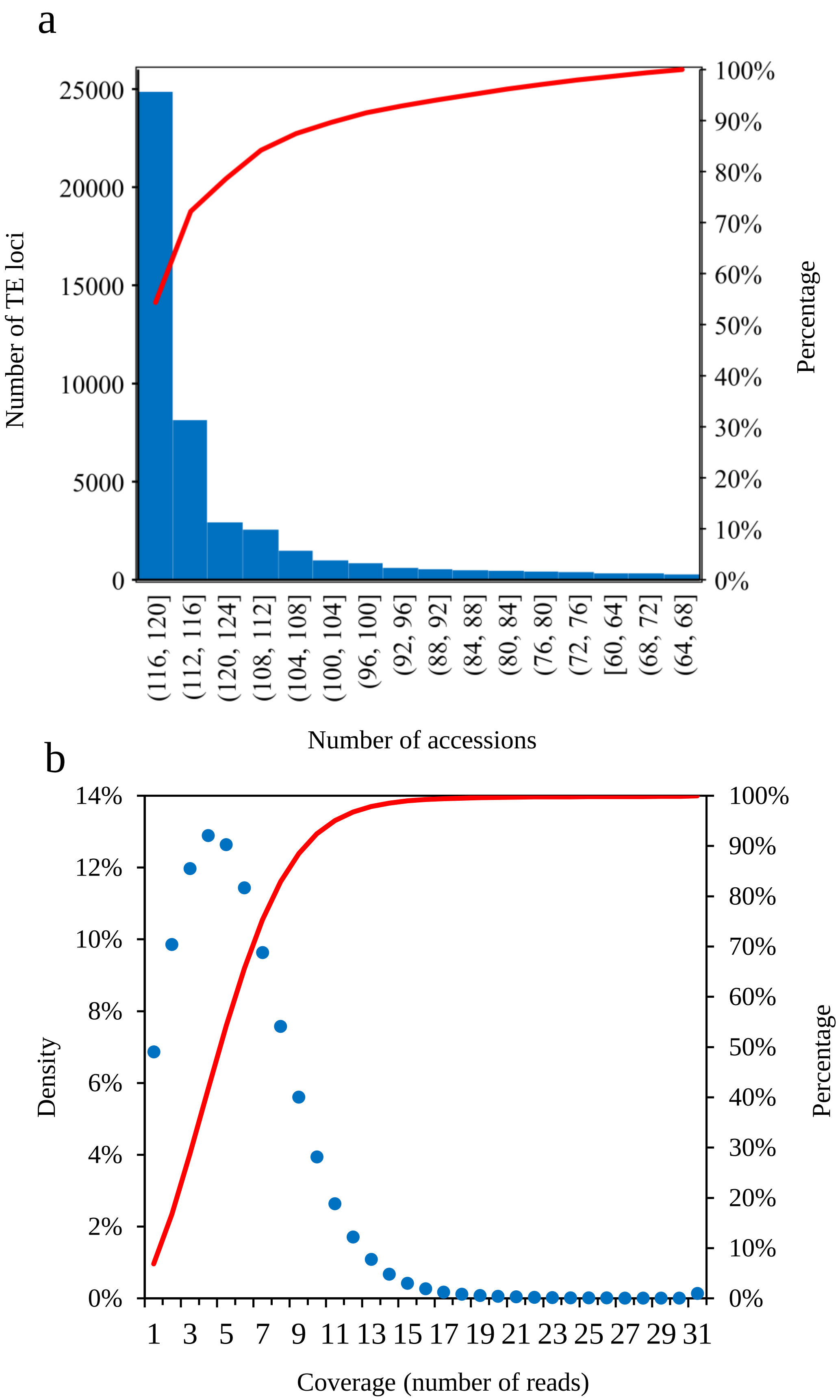

Supplementary Figure 8 The detection rates (a) and reads coverage (b) of 45,737 TE loci in 121 diverse accessions.

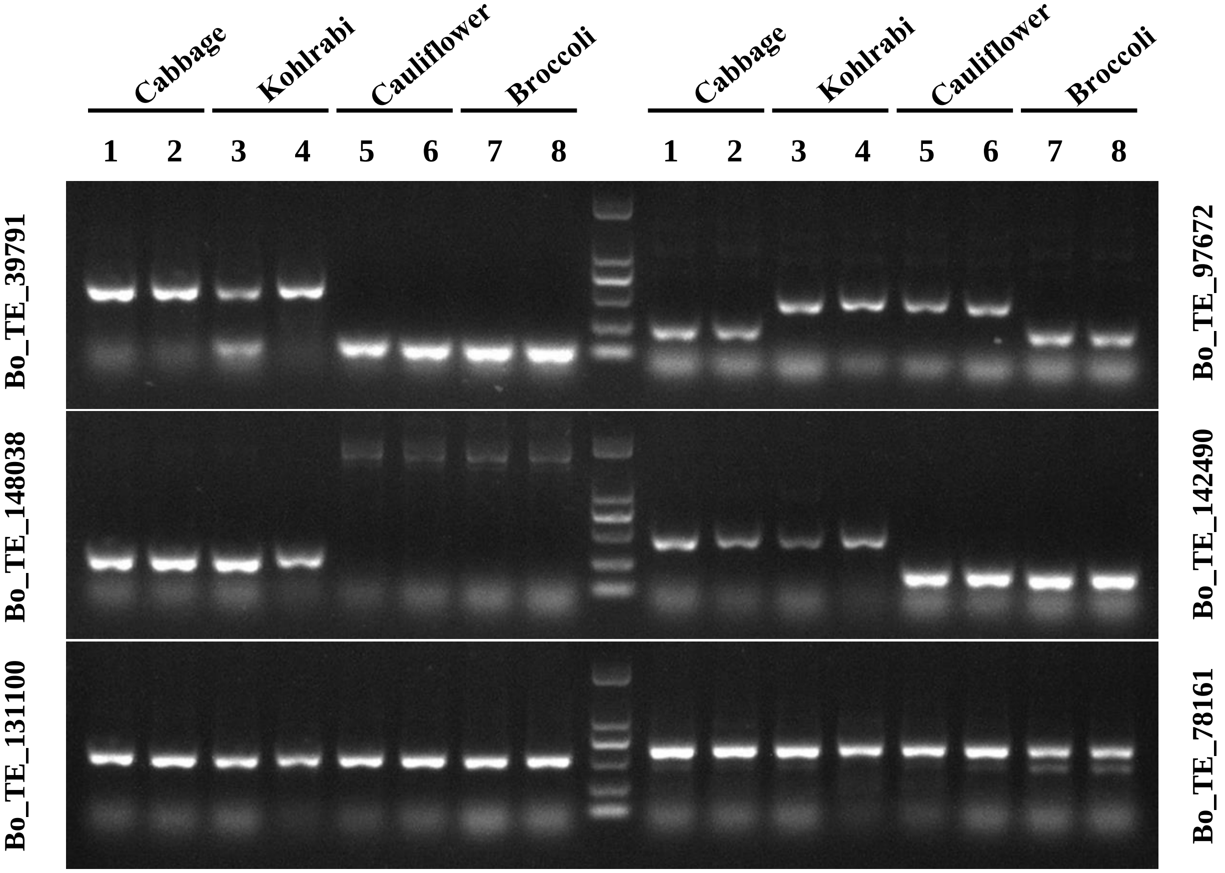

Supplementary Figure 9 PCR detection for the presence or absence of six TE loci in eight *B. oleracea* accessions. The accessions order was as follows: 1, JF-1; 2, ZG-21 ; 3, TJQ; 4, LPL-1; 5, FZ-80; 6, FZ-60; 7, ML; 8, LFS.

Supplementary Table 1 The material information of 121 resequencing B. oleracea accessions.[a]

Accession ID	Morphotypes	Type	Resequencing data(Gb)
Cabbage 01	var. *capitata*	Inbred line	6.87
Cabbage 02	var. *capitata*	Inbred line	7.19
Cabbage 03	var. *capitata*	Inbred line	7.05
Cabbage 04	var. *capitata*	Inbred line	7.16
Cabbage 05	var. *capitata*	Inbred line	6.18
Cabbage 06	var. *capitata*	Inbred line	7.06
Cabbage 07	var. *capitata*	Inbred line	6.95
Cabbage 08	var. *capitata*	Inbred line	6.96
Cabbage 09	var. *capitata*	Inbred line	7.04
Cabbage 10	var. *capitata*	Germplasm	3.77
Cabbage 11	var. *capitata*	Germplasm	4.03
Cabbage 12	var. *capitata*	Germplasm	8.72
Cabbage 13	var. *capitata*	Germplasm	3.73
Cabbage 14	var. *capitata*	Germplasm	5.26
Cabbage 15	var. *capitata*	Germplasm	5.49
Cabbage 16	var. *capitata*	Germplasm	4.30
Cabbage 17	var. *capitata*	Germplasm	7.63
Cabbage 18	var. *capitata*	Germplasm	4.23
Cabbage 19	var. *capitata*	Germplasm	6.26
Cabbage 20	var. *capitata*	Germplasm	4.41
Cabbage 21	var. *capitata*	Germplasm	4.24
Cabbage 22	var. *capitata*	Germplasm	5.17
Cabbage 23	var. *capitata*	Germplasm	5.11
Cabbage 24	var. *capitata*	Germplasm	1.95
Cabbage 25	var. *capitata*	Germplasm	2.99
Cabbage 26	var. *capitata*	Germplasm	3.95
Cabbage 27	var. *capitata*	Germplasm	4.00
Cabbage 28	var. *capitata*	Germplasm	1.92
Cabbage 29	var. *capitata*	Inbred line	6.39
Pointed Cabbage 01	var. *capitata*	Inbred line	7.15
Pointed Cabbage 02	var. *capitata*	Inbred line	8.29
Pointed Cabbage 03	var. *capitata*	Inbred line	7.01
White Cabbage 01	var. *capitata*	Inbred line	7.37
White Cabbage 02	var. *capitata*	Inbred line	7.35
White Cabbage 03	var. *capitata*	Inbred line	7.36
White Cabbage 04	var. *capitata*	Inbred line	7.71
White Cabbage 05	var. *capitata*	Inbred line	7.41
White Cabbage 06	var. *capitata*	Inbred line	6.77
White Cabbage 07	var. *capitata*	Inbred line	7.28
White Cabbage 08	var. *capitata*	Inbred line	7.23
White Cabbage 09	var. *capitata*	Inbred line	7.35
White Cabbage 10	var. *capitata*	Inbred line	5.85
White Cabbage 11	var. *capitata*	Inbred line	5.68
Cabbage 30	var. *capitata*	Germplasm	5.23
Cabbage 31	var. *capitata*	Germplasm	6.04
Kohlrabi 01	var. *gongylodes*	Inbred line	6.91
Kohlrabi 02	var. *gongylodes*	Inbred line	7.56
Kohlrabi 03	var. *gongylodes*	Inbred line	7.21
Kohlrabi 04	var. *gongylodes*	Inbred line	7.49
Kohlrabi 05	var. *gongylodes*	Inbred line	7.48
Kohlrabi 06	var. *gongylodes*	Inbred line	5.14
Kohlrabi 07	var. *gongylodes*	Genebank	6.94

Kohlrabi 08	var. *gongylodes*	Genebank	6.91
Kohlrabi 09	var. *gongylodes*	Inbred line	7.52
Kohlrabi 10	var. *gongylodes*	Inbred line	7.65
Kohlrabi 11	var. *gongylodes*	Inbred line	7.41
Kohlrabi 12	var. *gongylodes*	Inbred line	7.74
Kohlrabi 13	var. *gongylodes*	Inbred line	7.59
Kohlrabi 14	var. *gongylodes*	Inbred line	7.65
Kohlrabi 15	var. *gongylodes*	Inbred line	7.25
Kohlrabi 16	var. *gongylodes*	Germplasm	4.56
Kohlrabi 17	var. *gongylodes*	Germplasm	6.56
Kohlrabi 18	var. *gongylodes*	Germplasm	4.66
Kohlrabi 19	var. *gongylodes*	Germplasm	4.75
Cauliflower 01	var. *botrytis*	Inbred line	7.59
Cauliflower 02	var. *botrytis*	Inbred line	7.63
Cauliflower 03	var. *botrytis*	Inbred line	7.20
Cauliflower 04	var. *botrytis*	Inbred line	7.38
Cauliflower 05	var. *botrytis*	Inbred line	7.24
Cauliflower 06	var. *botrytis*	Inbred line	7.13
Cauliflower 07	var. *botrytis*	Inbred line	7.13
Cauliflower 08	var. *botrytis*	Inbred line	7.31
Cauliflower 09	var. *botrytis*	Inbred line	7.21
Cauliflower 10	var. *botrytis*	Inbred line	6.84
Cauliflower 11	var. *botrytis*	Inbred line	6.86
Cauliflower 12	var. *botrytis*	Inbred line	6.55
Cauliflower 13	var. *botrytis*	Inbred line	7.46
Cauliflower 14	var. *botrytis*	Inbred line	7.54
Cauliflower 15	var. *botrytis*	Inbred line	6.86
Cauliflower 16	var. *botrytis*	Germplasm	4.80
Cauliflower 17	var. *botrytis*	Germplasm	5.03
Cauliflower 18	var. *botrytis*	Germplasm	4.64
Cauliflower 19	var. *botrytis*	Germplasm	4.07
Cauliflower 20	var. *botrytis*	Germplasm	5.32
Broccoli 01	var. *italica*	Inbred line	7.53
Broccoli 02	var. *italica*	Inbred line	7.56
Broccoli 03	var. *italica*	Inbred line	7.23
Broccoli 04	var. *italica*	Inbred line	6.32
Broccoli 05	var. *italica*	Inbred line	6.84
Broccoli 06	var. *italica*	Inbred line	6.92
Broccoli 07	var. *italica*	Inbred line	6.85
Broccoli 08	var. *italica*	Inbred line	7.01
Broccoli 09	var. *italica*	Inbred line	6.91
Broccoli 10	var. *italica*	Inbred line	7.89
Broccoli 11	var. *italica*	Genebank	6.67
Broccoli 12	var. *italica*	Genebank	6.63
Broccoli 13	var. *italica*	Inbred line	6.91
Broccoli 14	var. *italica*	Inbred line	6.91
Broccoli 15	var. *italica*	Inbred line	6.97
Broccoli 16	var. *italica*	Inbred line	6.99
Broccoli 17	var. *italica*	Inbred line	7.17
Broccoli 18	var. *italica*	Germplasm	4.18
Broccoli 19	var. *italica*	Germplasm	4.58
Broccoli 20	var. *italica*	Germplasm	4.13
Broccoli 21	var. *italica*	Germplasm	3.71
Broccoli 22	var. *italica*	Germplasm	4.45
Broccoli 23	var. *italica*	Germplasm	4.67
Chinese kale 01	var. *alboglabra*	Germplasm	5.26

Chinese kale 02	var. *alboglabra*	Germplasm	5.32
Chinese kale 03	var. *alboglabra*	Germplasm	7.28
Chinese kale 04	var. *alboglabra*	Germplasm	3.11
Brussels sprouts 01	var. *gemmifera*	Germplasm	4.64
Brussels sprouts 02	var. *gemmifera*	Germplasm	5.36
Kale 01	var. *acephala*	Germplasm	3.92
Kale 02	var. *acephala*	Germplasm	6.70
Curly Kale 01	var. *sabellica*	Inbred line	7.41
Curly Kale 02	var. *sabellica*	Inbred line	6.85
Wild 01	wild type	Genebank	6.81
Wild 02	wild type	Genebank	6.80

[a] The information invovled in the table was referenced from Cheng *et al* ., 2016, *Scientific Data* .

Supplementary Table 2 The proportion of four frequency type in different superfamilies and three families.

Superfamily/family[a]	Low	Median	High	Fixed
LTR/Copia(8256)	0.48	0.19	0.10	0.23
LTR/Gypsy(2271)	0.36	0.18	0.09	0.37
LTR/Ukn(1161)	0.36	0.19	0.10	0.36
SINE(2063)	0.49	0.22	0.09	0.19
LINE(623)	0.15	0.05	0.06	0.74
Pong(6897)	0.33	0.19	0.13	0.36
CACTA(4850)	0.58	0.22	0.11	0.09
Tc1/Mariner(4219)	0.32	0.14	0.11	0.43
hAT(1719)	0.46	0.18	0.10	0.26
Mutator(1623)	0.41	0.17	0.11	0.30
PIF/Harbinger(1309)	0.58	0.18	0.09	0.15
Helitron(10746)	0.34	0.20	0.12	0.34
**Copia**				
Bor289(872)	0.68	0.22	0.07	0.02
Bor3(832)	0.79	0.16	0.04	0.01
Bor5(327)	0.25	0.17	0.17	0.42
Bor77(251)	0.47	0.26	0.12	0.14
Bor60(243)	0.19	0.16	0.09	0.55
Bor2(223)	0.86	0.12	0.02	0
Bor53(199)	0.49	0.2	0.16	0.16
Bor4(194)	0.7	0.18	0.1	0.02
Bor142(191)	0.31	0.32	0.2	0.16
Bor576(187)	0.77	0.16	0.06	0.01
Bor280(154)	0.77	0.18	0.05	0.01
Bor311(140)	0.34	0.27	0.12	0.26
Bor64(137)	0.19	0.21	0.09	0.51
Bor325(132)	0.11	0.17	0.11	0.61
Bor15(132)	0.42	0.27	0.15	0.16
Bor1(122)	0.31	0.25	0.21	0.23
Bor12(117)	0.72	0.2	0.08	0.01
Bor149(114)	0.03	0.02	0.03	0.93
Bor209(104)	0.62	0.21	0.07	0.11
Bor630(102)	0.36	0.24	0.2	0.21
Bor7(98)	0.35	0.23	0.19	0.22
Bor9(98)	0.49	0.2	0.14	0.16
Bor16(91)	0.14	0.16	0.13	0.56
Bor297(86)	0.16	0.35	0.14	0.35
Bor288(85)	0.6	0.29	0.07	0.04
Bor164(84)	0.04	0.04	0.08	0.85
Bor405(84)	0.12	0.15	0.1	0.63
Bor792(78)	0.22	0.22	0.17	0.4
Bor216(76)	0.63	0.3	0.05	0.01
Bor239(75)	0.79	0.11	0.04	0.07
Bor10(71)	0.52	0.24	0.08	0.15
Bor66(70)	0.16	0.11	0.19	0.54
Bor143(66)	0.09	0.15	0.14	0.62
Bor13(64)	0.13	0.05	0.03	0.8
Bor185(64)	0	0	0	1
Bor136(61)	0.79	0.08	0.05	0.08

Bor569(57)	0.3	0.4	0.18	0.12
Bor559(53)	0.58	0.25	0.09	0.08
Bor238(52)	0.54	0.23	0.17	0.06
Bor614(51)	0.67	0.12	0.08	0.14
Bor14(51)	0.47	0.29	0.12	0.12
Bor6(50)	0.84	0.12	0.04	0
Bor447(49)	0.39	0.27	0.14	0.2
Bor565(47)	0.45	0.26	0.19	0.11
Bor392(46)	0.57	0.22	0.09	0.13
Bor553(46)	0.17	0.22	0.28	0.33
Bor278(45)	0.76	0.13	0.07	0.04
Bor558(43)	0.19	0.09	0.21	0.51
Bor619(42)	0.9	0.07	0	0.02
Bor537(38)	0.79	0.03	0.05	0.13
Bor8(37)	0.11	0.19	0.27	0.43
Bor46(35)	0	0	0	1
Bor63(35)	0.57	0.2	0.11	0.11
Bor366(34)	0.56	0.18	0.12	0.15
Bor628(32)	0.09	0.06	0.28	0.56
Bor497(31)	0.39	0.35	0.23	0.03
Bor300(30)	0.13	0.2	0.17	0.5
Bor197(29)	0.21	0.17	0.14	0.48
Bor227(28)	0.89	0.11	0	0
Bor327(28)	0.07	0.18	0.14	0.61
Bor549(27)	0.93	0	0.04	0.04
Bor201(27)	0.74	0.11	0.11	0.04
Bor87(25)	0.4	0.12	0.2	0.28
Bor52(24)	0.79	0.13	0.08	0
Bor293(24)	0.17	0.17	0.17	0.5
Bor44(24)	0.38	0.08	0.08	0.46
Bor223(23)	0.09	0.13	0.13	0.65
Bor409(22)	0.36	0.18	0	0.45
Bor382(22)	0.59	0.14	0.09	0.18
Bor81(22)	0.18	0.09	0.09	0.64
Bor229(20)	0.15	0.1	0.2	0.55
Bor176(19)	0.42	0.16	0.16	0.26
Bor217(19)	0.26	0.05	0.26	0.42
Bor151(19)	0.89	0.05	0	0.05
Bor96(18)	0.33	0.11	0.06	0.5
Bor294(17)	0.47	0.35	0.18	0
Bor437(17)	0.65	0.29	0	0.06
Bor173(16)	0.25	0.06	0	0.69
Bor567(15)	0.27	0	0.2	0.53
Bor69(15)	0.73	0.2	0	0.07
Bor266(14)	0.36	0.21	0.07	0.36
Bor379(13)	0.31	0.23	0.46	0
Bor441(13)	0.15	0.31	0.08	0.46
Bor49(13)	0.23	0.46	0	0.31
Bor383(13)	0.08	0.15	0.08	0.69
Bor396(12)	0.33	0.25	0.17	0.25
Bor207(12)	0.42	0.25	0.08	0.25
Bor180(11)	0	0.09	0.09	0.82
Bor768(11)	0.45	0.36	0	0.18
Bor599(11)	0.55	0.27	0.09	0.09
Bor40(10)	0.5	0.2	0.2	0.1
Bor490(10)	0.3	0.2	0.1	0.4

Bor255(10)	0.3	0.5	0	0.2
Bor566(10)	0.1	0.1	0.1	0.7
Bor307(9)	0.44	0.22	0.22	0.11
Bor417(9)	0.11	0	0.33	0.56
Bor560(9)	0.22	0	0.11	0.67
Bor530(9)	0.56	0	0.11	0.33
Bor144(9)	0.22	0.44	0.22	0.11
Bor165(8)	0.63	0.25	0	0.13
Bor403(8)	0.88	0	0	0.13
Bor684(8)	0.5	0.25	0.25	0
Bor97(8)	0.13	0.5	0	0.38
Bor279(8)	0.13	0.25	0.13	0.5
Bor206(7)	0.43	0.29	0.29	0
Bor285(7)	0.29	0.14	0.43	0.14
Bor603(7)	0.86	0	0	0.14
Bor274(7)	0.71	0.29	0	0
Bor495(7)	0.71	0.29	0	0
Bor45(7)	0	0.14	0	0.86
Bor488(7)	0.29	0.29	0.14	0.29
Bor11(6)	0	0	0	1
Bor192(6)	0.5	0.33	0	0.17
Bor55(6)	0.17	0	0	0.83
Bor260(6)	0.33	0.17	0.17	0.33
Bor310(6)	0	0	0	1
Bor650(6)	0	0.17	0	0.83
Bor438(6)	0.83	0.17	0	0
Bor494(6)	0.5	0	0.17	0.33
Bor694(6)	0.33	0.5	0	0.17
Bor234(6)	0.33	0.17	0.17	0.33
Bor501(6)	0	0.5	0	0.5
Bor643(5)	0.2	0.2	0.2	0.4
Bor134(5)	0.8	0	0.2	0
Bor263(5)	0.6	0	0.2	0.2
Bor89(5)	0.8	0.2	0	0
Bor570(5)	0.2	0.8	0	0
Bor32(5)	0	0	0	1
Bor93(5)	0.6	0	0	0.4
Bor556(5)	0.4	0.4	0.2	0
Bor126(5)	1	0	0	0
Bor264(5)	0.2	0.2	0.2	0.4
Bor20(5)	0.4	0	0	0.6
Bor638(5)	0.4	0	0.4	0.2
Bor152(5)	1	0	0	0
Bor296(4)	0	0.25	0	0.75
Bor561(4)	0	0.75	0.25	0
Bor25(4)	0.25	0.25	0	0.5
Bor304(4)	0.5	0.25	0.25	0
Bor376(4)	0.25	0	0	0.75
Bor169(4)	0.5	0	0	0.5
Bor39(4)	0.25	0	0	0.75
Bor78(4)	0.25	0.25	0.25	0.25
Bor58(4)	0	0	0.25	0.75
Bor290(4)	0.25	0	0	0.75
Bor456(4)	0.25	0.5	0.25	0
Bor158(4)	0.75	0.25	0	0
Bor413(4)	0.5	0.25	0	0.25

Bor120(4)	0	0.5	0.25	0.25
Bor339(4)	0	0	0	1
Bor85(4)	1	0	0	0
Bor157(4)	0.5	0	0	0.5
Bor408(4)	0.5	0.25	0.25	0
Bor33(4)	0	0	0.25	0.75
Bor373(4)	0.75	0.25	0	0
Bor113(4)	0.75	0	0.25	0
Bor121(4)	0.75	0.25	0	0
Bor114(4)	0.25	0	0.25	0.5
Bor188(4)	0.75	0.25	0	0
Bor679(4)	0.25	0.5	0	0.25
Bor31(3)	0.33	0	0	0.67
Bor189(3)	0	0.33	0.33	0.33
Bor153(3)	0.33	0.33	0	0.33
Bor71(3)	0.33	0.33	0	0.33
Bor552(3)	0	0.33	0	0.67
Bor354(3)	0	0	0	1
Bor523(3)	0	0	0	1
Bor302(3)	0	0	0.67	0.33
Bor526(3)	0	0.67	0	0.33
Bor175(3)	0	0	0.33	0.67
Bor380(3)	0.67	0	0	0.33
Bor334(3)	0.33	0	0.33	0.33
Bor351(3)	0.67	0.33	0	0
Bor663(3)	0.67	0.33	0	0
Bor226(3)	0.33	0	0	0.67
Bor276(3)	0.67	0.33	0	0
Bor618(3)	0.33	0	0	0.67
Bor580(3)	1	0	0	0
Bor258(3)	0.33	0.33	0	0.33
Bor41(3)	0.33	0	0.33	0.33
Bor166(3)	0.33	0.67	0	0
Bor575(3)	0.33	0.33	0.33	0
Bor50(3)	0.33	0	0.33	0.33
Bor669(3)	0.33	0.33	0	0.33
Bor172(3)	0.33	0.33	0.33	0
Bor350(3)	0.67	0.33	0	0
Bor607(3)	0	0	0	1
Bor57(3)	0.67	0.33	0	0
Bor277(3)	0.67	0.33	0	0
Bor147(3)	0.33	0	0.67	0
Bor701(3)	0	0	0	1
Bor259(3)	0	0	0.33	0.67
Bor652(3)	0	0	0	1
Bor80(2)	0	0	0.5	0.5
Bor459(2)	0.5	0	0	0.5
Bor23(2)	0	0.5	0	0.5
Bor448(2)	0	0	1	0
Bor595(2)	0	0	0.5	0.5
Bor372(2)	0.5	0.5	0	0
Bor190(2)	0.5	0	0	0.5
Bor664(2)	0.5	0	0	0.5
Bor511(2)	0.5	0.5	0	0
Bor318(2)	0.5	0	0.5	0
Bor83(2)	0	1	0	0

Bor505(2)	0	0	0	1
Bor268(2)	0.5	0	0.5	0
Bor47(2)	0.5	0	0	0.5
Bor19(2)	0	0	0.5	0.5
Bor326(2)	0	0	0	1
Bor43(2)	0.5	0	0.5	0
Bor34(2)	0	0.5	0.5	0
Bor527(2)	0	1	0	0
Bor291(2)	0	0	0.5	0.5
Bor320(2)	0	0	0	1
Bor432(2)	0.5	0.5	0	0
Bor687(2)	0	0.5	0	0.5
Bor398(2)	0	0	0	1
Bor116(2)	0	1	0	0
Bor195(2)	0	0	0	1
Bor502(2)	0.5	0	0	0.5
Bor666(2)	0	0	0.5	0.5
Bor79(2)	0	0	0	1
Bor106(2)	0	0.5	0	0.5
Bor231(2)	0	0.5	0	0.5
Bor273(2)	0	0	0	1
Bor739(2)	0	0	0	1
Bor515(2)	0.5	0	0	0.5
Bor117(2)	0	0	0	1
Bor564(2)	0	0	0	1
Bor394(2)	0	0	0	1
Bor648(2)	0	0	0	1
Bor582(2)	1	0	0	0
Bor35(2)	0	0.5	0	0.5
Bor135(2)	1	0	0	0
Bor42(2)	0	0.5	0	0.5
Bor369(2)	0	0	0	1
Bor281(2)	0	0.5	0.5	0
Bor212(2)	0	0	0.5	0.5
Bor443(2)	1	0	0	0
Bor29(2)	1	0	0	0
Bor604(2)	0	0	0	1
Bor215(2)	0	0	1	0
Bor642(2)	0.5	0	0	0.5
Bor56(2)	0	0	0	1
Bor61(2)	0.5	0.5	0	0
Bor240(2)	0	0	0	1
Bor540(2)	0	0	0.5	0.5
Bor468(2)	0	0.5	0	0.5
Bor305(2)	0	0	0.5	0.5
Bor84(2)	1	0	0	0
Bor67(2)	0.5	0	0.5	0
Bor546(2)	0	0	0.5	0.5
Bor429(2)	0	1	0	0
Bor348(2)	0.5	0	0.5	0
Bor214(2)	0.5	0.5	0	0
Bor568(1)	1	0	0	0
Bor210(1)	0	0	0	1
Bor184(1)	0	0	0	1
Bor678(1)	0	1	0	0
Bor454(1)	0	0	1	0

Bor414(1)	0	1	0	0
Bor378(1)	0	0	0	1
Bor317(1)	0	0	0	1
Bor194(1)	1	0	0	0
Bor364(1)	1	0	0	0
Bor130(1)	1	0	0	0
Bor59(1)	0	1	0	0
Bor371(1)	0	0	0	1
Bor551(1)	0	0	0	1
Bor637(1)	1	0	0	0
Bor681(1)	0	0	0	1
Bor538(1)	0	0	0	1
Bor453(1)	0	1	0	0
Bor435(1)	0	0	0	1
Bor17(1)	0	0	0	1
Bor660(1)	0	0	1	0
Bor38(1)	0	0	0	1
Bor522(1)	0	0	1	0
Bor163(1)	0	0	1	0
Bor542(1)	0	1	0	0
Bor150(1)	0	0	0	1
Bor125(1)	0	0	1	0
Bor51(1)	0	0	1	0
Bor368(1)	0	0	0	1
Bor86(1)	0	1	0	0
Bor18(1)	1	0	0	0
Bor267(1)	0	0	0	1
Bor696(1)	0	1	0	0
Bor94(1)	0	0	0	1
Bor26(1)	1	0	0	0
Bor360(1)	0	0	1	0
Bor208(1)	0	0	0	1
Bor82(1)	0	0	0	1
Bor118(1)	1	0	0	0
Bor193(1)	0	1	0	0
Bor393(1)	0	0	0	1
Bor275(1)	1	0	0	0
Bor75(1)	0	1	0	0
Bor395(1)	0	0	0	1
Bor621(1)	0	0	0	1
Bor682(1)	0	0	1	0
Bor315(1)	0	0	0	1
Bor70(1)	0	0	0	1
Bor28(1)	0	0	0	1
Bor119(1)	1	0	0	0
Bor27(1)	0	0	0	1
Bor30(1)	1	0	0	0
Bor434(1)	1	0	0	0
Bor145(1)	0	0	0	1
Bor705(1)	1	0	0	0
Bor24(1)	0	0	0	1
Bor62(1)	0	0	0	1
Bor319(1)	0	0	0	1
Bor298(1)	0	0	1	0
Bor198(1)	1	0	0	0
Bor316(1)	0	0	0	1

Bor510(1)	1	0	0	0
Bor65(1)	0	0	0	1
Bor148(1)	0	0	1	0
Bor457(1)	0	0	0	1
Bor375(1)	0	0	0	1
Bor481(1)	0	0	0	1
Bor88(1)	0	0	0	1
Bor602(1)	0	1	0	0
Bor512(1)	0	0	1	0
Bor73(1)	0	0	0	1
Bor352(1)	0	0	0	1
Bor237(1)	0	0	0	1
Bor159(1)	1	0	0	0
Bor324(1)	0	0	0	1
Bor261(1)	0	0	1	0
Bor357(1)	0	0	0	1
**Gypsy**				
Bor397(194)	0.51	0.3	0.09	0.1
Bor728(136)	0.16	0.15	0.18	0.51
Bor330(129)	0.79	0.17	0.04	0
Bor377(112)	0.67	0.19	0.06	0.08
Bor340(97)	0.23	0.14	0.08	0.55
Bor451(73)	0.27	0.32	0.16	0.25
Bor162(58)	0.16	0.03	0.07	0.74
Bor139(46)	0.61	0.17	0.04	0.17
Bor649(46)	0.04	0.04	0.02	0.89
Bor707(42)	0.02	0.05	0.07	0.86
Bor671(40)	0.33	0.47	0.1	0.1
Bor489(39)	0.38	0.23	0.18	0.21
Bor170(36)	0.69	0.17	0.03	0.11
Bor620(35)	0.17	0.23	0.17	0.43
Bor431(35)	0.74	0.2	0.03	0.03
Bor766(34)	0.44	0.29	0.15	0.12
Bor764(34)	0.24	0.21	0.15	0.41
Bor174(32)	0.47	0.13	0.06	0.34
Bor107(32)	0.03	0.13	0.19	0.66
Bor183(31)	0.06	0.06	0.06	0.81
Bor531(31)	0.06	0.06	0.06	0.81
Bor653(30)	0.27	0.23	0.03	0.47
Bor752(29)	0.62	0.24	0.14	0
Bor303(26)	0	0.08	0.08	0.85
Bor218(26)	0.08	0	0.23	0.69
Bor168(24)	0.29	0.25	0.08	0.38
Bor778(23)	0.39	0.17	0	0.43
Bor337(23)	0	0.04	0	0.96
Bor249(22)	0.82	0.05	0.09	0.05
Bor132(22)	0.95	0.05	0	0
Bor475(21)	0.62	0	0.1	0.29
Bor178(21)	0	0	0.05	0.95
Bor478(20)	0.55	0.15	0.15	0.15
Bor532(20)	0.1	0.15	0.15	0.6
Bor244(20)	0.2	0.2	0.05	0.55
Bor110(20)	0.4	0.2	0.1	0.3
Bor574(19)	0.37	0.37	0.05	0.21
Bor462(18)	1	0	0	0

Bor710(17)	0.24	0.24	0.18	0.35
Bor161(17)	0.47	0.29	0.06	0.18
Bor295(15)	0	0.07	0.2	0.73
Bor776(15)	0	0.13	0.07	0.8
Bor725(15)	0.27	0.27	0.13	0.33
Bor385(15)	0.13	0.2	0.2	0.47
Bor154(14)	0.07	0.29	0.07	0.57
Bor783(14)	0.29	0.36	0	0.36
Bor750(12)	0.58	0.33	0	0.08
Bor199(12)	0.33	0.08	0	0.58
Bor446(12)	0.17	0.25	0.17	0.42
Bor727(12)	0.08	0	0	0.92
Bor224(12)	0.58	0.25	0.08	0.08
Bor269(11)	0.82	0.18	0	0
Bor141(11)	0	0	0.09	0.91
Bor665(10)	0.4	0.2	0.1	0.3
Bor467(10)	0	0.3	0.1	0.6
Bor109(9)	0.56	0.22	0	0.22
Bor616(8)	0.38	0.13	0.5	0
Bor245(8)	0.75	0.13	0	0.13
Bor518(8)	0.38	0.38	0.25	0
Bor562(8)	0.13	0.13	0	0.75
Bor718(7)	0	0.14	0	0.86
Bor92(7)	0.29	0.29	0.14	0.29
Bor253(7)	0.86	0	0	0.14
Bor410(6)	0.5	0.17	0.17	0.17
Bor477(6)	0.5	0.17	0	0.33
Bor767(6)	0	0	0	1
Bor124(6)	0.67	0	0	0.33
Bor270(5)	0.2	0	0	0.8
Bor539(5)	0.2	0	0	0.8
Bor115(5)	0.8	0.2	0	0
Bor734(5)	0	0	0	1
Bor112(5)	0.8	0.2	0	0
Bor251(5)	0.4	0.4	0	0.2
Bor228(5)	0.8	0.2	0	0
Bor423(5)	0	0	0	1
Bor358(4)	0.5	0	0.25	0.25
Bor611(4)	0.75	0.25	0	0
Bor155(4)	0	0	0.25	0.75
Bor436(4)	0	1	0	0
Bor133(4)	0.5	0	0	0.5
Bor452(4)	0	0.5	0.25	0.25
Bor550(4)	0.5	0.5	0	0
Bor254(4)	0.25	0.5	0.25	0
Bor606(4)	0	0	0	1
Bor673(4)	0	0	0	1
Bor686(4)	0	0.5	0	0.5
Bor779(4)	0.25	0	0	0.75
Bor464(4)	1	0	0	0
Bor720(3)	0	0	0	1
Bor181(3)	0	0	0	1
Bor103(3)	0.67	0.33	0	0
Bor476(3)	0	0.33	0	0.67
Bor242(3)	0	0	0.33	0.67
Bor177(3)	0.67	0	0	0.33

Bor225(3)	0	0	0	1
Bor782(3)	0.33	0	0	0.67
Bor128(3)	0.67	0.33	0	0
Bor361(3)	0	0.33	0	0.67
Bor661(2)	0	0	0	1
Bor699(2)	0	0	0	1
Bor108(2)	0.5	0.5	0	0
Bor520(2)	0	0	0	1
Bor111(2)	1	0	0	0
Bor99(2)	0.5	0.5	0	0
Bor465(2)	1	0	0	0
Bor753(2)	0	0.5	0.5	0
Bor711(2)	0	0	0	1
Bor374(2)	0	1	0	0
Bor388(2)	0.5	0	0	0.5
Bor504(2)	1	0	0	0
Bor179(2)	0	0	0.5	0.5
Bor250(2)	0	0	0	1
Bor100(2)	0	0.5	0	0.5
Bor612(2)	0	0	0	1
Bor491(2)	1	0	0	0
Bor471(2)	0.5	0	0	0.5
Bor430(2)	0	0	0	1
Bor610(2)	0	0	0	1
Bor401(2)	0	0.5	0	0.5
Bor493(2)	0	0.5	0	0.5
Bor346(2)	0	1	0	0
Bor672(2)	0	0	0	1
Bor248(2)	0	0	0	1
Bor355(2)	0	0	0.5	0.5
Bor460(2)	0	0	0	1
Bor272(2)	1	0	0	0
Bor342(2)	0	0	0.5	0.5
Bor356(2)	0	0	0	1
Bor484(2)	0.5	0	0	0.5
Bor700(2)	0.5	0.5	0	0
Bor444(2)	0	0.5	0	0.5
Bor104(2)	0	0.5	0.5	0
Bor333(2)	0	1	0	0
Bor461(2)	0	0	0	1
Bor656(2)	0.5	0	0	0.5
Bor314(2)	0	0	0	1
Bor585(2)	0.5	0.5	0	0
Bor367(2)	0.5	0	0	0.5
Bor309(2)	0.5	0	0	0.5
Bor271(2)	0	0.5	0	0.5
Bor131(2)	0	0	0	1
Bor122(2)	1	0	0	0
Bor450(1)	0	0	0	1
Bor646(1)	1	0	0	0
Bor127(1)	0	1	0	0
Bor470(1)	0	1	0	0
Bor91(1)	1	0	0	0
Bor323(1)	0	0	0	1
Bor654(1)	0	0	0	1
Bor659(1)	0	0	1	0

Bor600(1)	0	0	0	1
Bor241(1)	0	0	0	1
Bor625(1)	0	0	0	1
Bor202(1)	0	0	0	1
Bor645(1)	1	0	0	0
Bor545(1)	0	0	0	1
Bor365(1)	0	0	0	1
Bor407(1)	0	0	0	1
Bor138(1)	0	1	0	0
Bor692(1)	0	1	0	0
Bor312(1)	0	0	1	0
Bor389(1)	0	1	0	0
Bor695(1)	0	0	0	1
Bor563(1)	0	0	0	1
Bor246(1)	0	0	0	1
Bor370(1)	0	1	0	0
Bor129(1)	0	0	0	1
Bor473(1)	0	0	0	1
Bor479(1)	0	0	0	1
Bor433(1)	1	0	0	0
Bor458(1)	0	1	0	0
Bor101(1)	0	1	0	0
Bor613(1)	0	0	1	0
Bor233(1)	1	0	0	0
Bor483(1)	0	1	0	0
Bor123(1)	1	0	0	0
Bor578(1)	0	0	1	0
Bor222(1)	0	0	0	1
Bor655(1)	0	0	0	1
Bor191(1)	0	0	0	1
Bor200(1)	0	0	0	1
Bor634(1)	0	0	0	1
Bor362(1)	0	0	0	1
Bor617(1)	1	0	0	0
Bor421(1)	0	0	0	1
Bor54(1)	0	0	1	0
Bor662(1)	1	0	0	0
Bor544(1)	0	1	0	0
Bor691(1)	0	0	0	1
Bor102(1)	0	0	0	1
Bor230(1)	0	0	0	1
Bor341(1)	0	0	0	1
Bor232(1)	0	0	0	1
Bor588(1)	0	0	0	1
Bor90(1)	1	0	0	0
Bor422(1)	1	0	0	0
Bor257(1)	0	0	0	1
**CACTA**				
Boc1(1334)	0.6	0.24	0.12	0.04
Boc3(475)	0.63	0.25	0.1	0.01
Boc117(403)	0.68	0.22	0.08	0.02
Boc7(342)	0.45	0.24	0.15	0.16
Boc109(319)	0.64	0.23	0.1	0.03
Boc5(275)	0.43	0.22	0.13	0.21
Boc115(253)	0.55	0.23	0.11	0.12

Boc113(197)	0.72	0.2	0.07	0.02
Boc8(189)	0.79	0.12	0.08	0.01
Boc2(156)	0.5	0.23	0.17	0.1
Boc116(94)	0.52	0.26	0.11	0.12
Boc9(75)	0.79	0.13	0.03	0.05
Boc29(56)	0.2	0.21	0.27	0.32
Boc49(54)	0.31	0.19	0.24	0.26
Boc10(53)	0.83	0.15	0	0.02
Boc73(48)	0.46	0.19	0.15	0.21
Boc12(31)	0.19	0.19	0.1	0.52
Boc22(26)	0.54	0.08	0.23	0.15
Boc14(23)	0.74	0.04	0.04	0.17
Boc50(20)	0.65	0.15	0.1	0.1
Boc31(20)	0.5	0.15	0.05	0.3
Boc6(18)	0.83	0	0.17	0
Boc114(17)	0.24	0.24	0.06	0.47
Boc110(16)	0.56	0.13	0.06	0.25
Boc59(16)	0.25	0.5	0.13	0.13
Boc13(15)	0.53	0.33	0.13	0
Boc11(15)	0.33	0.27	0.07	0.33
Boc92(14)	0.64	0.07	0.14	0.14
Boc78(13)	0.38	0.08	0.15	0.38
Boc66(12)	0.08	0.08	0.08	0.75
Boc32(12)	0.08	0	0.17	0.75
Boc101(12)	0.33	0.25	0.17	0.25
Boc19(12)	0.17	0.33	0.08	0.42
Boc54(10)	0.7	0.3	0	0
Boc67(9)	0.44	0.33	0.11	0.11
Boc85(8)	0.25	0.5	0	0.25
Boc74(8)	0.75	0	0.13	0.13
Boc77(8)	0.25	0.5	0	0.25
Boc16(7)	0.43	0.14	0.29	0.14
Boc18(7)	0.14	0	0	0.86
Boc142(6)	0.33	0.33	0.17	0.17
Boc34(6)	0	0.33	0.17	0.5
Boc126(6)	0.67	0.17	0	0.17
Boc172(6)	0.67	0.33	0	0
Boc97(6)	0.5	0.17	0.33	0
Boc138(6)	0.33	0.17	0.33	0.17
Boc111(5)	0.8	0.2	0	0
Boc100(5)	0.6	0	0.2	0.2
Boc88(5)	0	0.2	0	0.8
Boc95(5)	0	0.4	0.2	0.4
Boc99(5)	0.8	0.2	0	0
Boc61(4)	0	0	0.25	0.75
Boc175(4)	0	0.25	0.75	0
Boc71(4)	1	0	0	0
Boc135(4)	0.5	0.5	0	0
Boc51(4)	0.5	0	0.25	0.25
Boc4(4)	0.5	0.25	0.25	0
Boc15(4)	0.5	0.5	0	0
Boc62(4)	0.75	0	0.25	0
Boc53(4)	1	0	0	0
Boc169(4)	0.75	0.25	0	0
Boc128(4)	0.5	0.5	0	0
Boc56(3)	0	0	0	1

Boc27(3)	0.67	0.33	0	0
Boc83(3)	0.33	0	0.67	0
Boc60(3)	0.33	0.33	0.33	0
Boc17(3)	0.67	0.33	0	0
Boc40(3)	0.33	0	0.33	0.33
Boc58(3)	0	0.33	0.33	0.33
Boc155(3)	1	0	0	0
Boc23(3)	1	0	0	0
Boc121(3)	0.33	0.33	0	0.33
Boc133(2)	0	0	0	1
Boc183(2)	0.5	0.5	0	0
Boc81(2)	0	0	1	0
Boc140(2)	0.5	0.5	0	0
Boc143(2)	1	0	0	0
Boc44(2)	0.5	0	0	0.5
Boc47(2)	0	0.5	0.5	0
Boc180(2)	1	0	0	0
Boc75(2)	0.5	0.5	0	0
Boc152(2)	0	0.5	0	0.5
Boc105(2)	0	0	0	1
Boc170(2)	0	0	0.5	0.5
Boc163(2)	0.5	0.5	0	0
Boc48(2)	0.5	0.5	0	0
Boc38(1)	1	0	0	0
Boc174(1)	0	0	1	0
Boc55(1)	0	1	0	0
Boc120(1)	0	0	0	1
Boc41(1)	0	0	1	0
Boc130(1)	0	0	0	1
Boc108(1)	1	0	0	0
Boc124(1)	0	0	0	1
Boc112(1)	1	0	0	0
Boc134(1)	0	0	0	1
Boc103(1)	0	1	0	0
Boc136(1)	0	0	0	1
Boc96(1)	0	0	0	1
Boc184(1)	1	0	0	0
Boc33(1)	0	0	1	0

[a] The total number of TE loci owned by each famliy was shown in parentheses.

Supplementary Table 3 The population frequencies of 9,127 TE loci with frequency heterogeneity among the four large morphotypes.[a]

	TE loci	Cabbage	Kohlrabi	Broccoli	Cauliflower
Morphotypes-differential fixed	Bo_TE_205363	0.00000(0/40)	0.00000(0/18)	0.90476(19/21)	0.83333(15/18)
	Bo_TE_4393	0.06977(3/43)	0.06250(1/16)	0.90476(19/21)	0.22222(4/18)
	Bo_TE_161101	0.90244(37/41)	0.33333(5/15)	0.00000(0/22)	0.42105(8/19)
	Bo_TE_173795	0.00000(0/46)	0.12500(2/16)	0.60000(12/20)	0.90000(18/20)
	Bo_TE_15547	1.00000(44/44)	0.94737(18/19)	0.04545(1/22)	0.05263(1/19)
	Bo_TE_168448	0.76190(32/42)	1.00000(18/18)	0.30000(6/20)	0.10000(2/20)
	Bo_TE_22363	0.19048(8/42)	0.18750(3/16)	0.90476(19/21)	0.00000(0/20)
	Bo_TE_196464	0.97727(43/44)	0.58824(10/17)	0.00000(0/22)	0.80000(16/20)
	Bo_TE_238173	0.04545(2/44)	0.00000(0/18)	0.95652(22/23)	1.00000(19/19)
	Bo_TE_205246	0.00000(0/44)	0.05556(1/18)	0.42857(9/21)	0.90000(18/20)
	Bo_TE_87607	0.04444(2/45)	0.06250(1/16)	0.90476(19/21)	0.35000(7/20)
	Bo_TE_48056	0.47368(18/38)	1.00000(18/18)	0.00000(0/21)	0.05263(1/19)
	Bo_TE_136753	0.00000(0/46)	0.10526(2/19)	1.00000(21/21)	0.80000(16/20)
	Bo_TE_142451	0.90476(38/42)	0.94444(17/18)	0.09524(2/21)	0.35000(7/20)
	Bo_TE_30536	0.72727(32/44)	0.94118(16/17)	0.04762(1/21)	0.65000(13/20)
	Bo_TE_101106	0.29545(13/44)	0.75000(12/16)	0.09524(2/21)	0.90000(18/20)
	Bo_TE_142490	0.95000(38/40)	0.73333(11/15)	0.00000(0/22)	0.00000(0/19)
	Bo_TE_226500	0.31707(13/41)	0.00000(0/16)	1.00000(23/23)	0.95000(19/20)
	Bo_TE_104951	0.67500(27/40)	0.05882(1/17)	0.95238(20/21)	0.77778(14/18)
	Bo_TE_235698	0.02222(1/45)	0.00000(0/19)	0.95455(21/22)	0.42105(8/19)
	Bo_TE_183168	0.27500(11/40)	1.00000(18/18)	0.00000(0/22)	0.00000(0/20)
	Bo_TE_235825	0.04762(2/42)	0.43750(7/16)	0.90909(20/22)	0.94444(17/18)
	Bo_TE_150833	0.00000(0/45)	0.27778(5/18)	0.90000(18/20)	0.50000(10/20)
	Bo_TE_181961	0.38636(17/44)	0.06250(1/16)	0.90476(19/21)	0.30000(6/20)
	Bo_TE_53737	0.97619(41/42)	0.52632(10/19)	0.09524(2/21)	0.15000(3/20)
	Bo_TE_51177	0.00000(0/45)	0.00000(0/19)	0.95455(21/22)	1.00000(17/17)
	Bo_TE_198495	0.90698(39/43)	0.72222(13/18)	0.63158(12/19)	0.05556(1/18)
	Bo_TE_150906	0.04651(2/43)	0.77778(14/18)	1.00000(22/22)	0.75000(15/20)
	Bo_TE_223631	0.00000(0/46)	0.22222(4/18)	0.25000(4/16)	0.94737(18/19)
	Bo_TE_216177	0.32500(13/40)	0.00000(0/17)	0.90476(19/21)	0.35294(6/17)
	Bo_TE_136269	0.02703(1/37)	0.18750(3/16)	0.94737(18/19)	0.80000(16/20)
	Bo_TE_66491	0.00000(0/43)	0.00000(0/14)	0.90909(20/22)	0.65000(13/20)
	Bo_TE_9937	0.36585(15/41)	0.94118(16/17)	0.27273(6/22)	0.00000(0/19)
	Bo_TE_230405	0.22222(10/45)	0.64706(11/17)	0.90000(18/20)	0.10000(2/20)
	Bo_TE_214651	0.00000(0/45)	0.23529(4/17)	1.00000(21/21)	0.77778(14/18)
	Bo_TE_226899	0.92500(37/40)	0.41176(7/17)	0.00000(0/22)	0.15789(3/19)
	Bo_TE_191342	0.00000(0/44)	0.56250(9/16)	0.14286(3/21)	0.90000(18/20)
	Bo_TE_6011	0.28205(11/39)	0.05882(1/17)	0.28571(6/21)	1.00000(20/20)
	Bo_TE_97274	0.56098(23/41)	0.94118(16/17)	0.00000(0/23)	0.00000(0/20)
	Bo_TE_24535	0.02326(1/43)	0.41176(7/17)	0.95652(22/23)	0.55000(11/20)
	Bo_TE_137087	0.07317(3/41)	0.84211(16/19)	0.47059(8/17)	1.00000(19/19)
	Bo_TE_38988	0.02439(1/41)	0.05882(1/17)	0.04762(1/21)	0.94444(17/18)
	Bo_TE_158691	0.32558(14/43)	0.94118(16/17)	0.36364(8/22)	0.00000(0/18)
	Bo_TE_73515	1.00000(43/43)	0.17647(3/17)	0.00000(0/20)	0.05556(1/18)
	Bo_TE_96348	0.00000(0/39)	0.00000(0/17)	0.38095(8/21)	0.93750(15/16)
	Bo_TE_234231	0.95000(38/40)	0.22222(2/9)	0.00000(0/23)	0.35000(7/20)
	Bo_TE_68625	0.06522(3/46)	0.00000(0/18)	0.95455(21/22)	0.52632(10/19)
	Bo_TE_196603	0.20930(9/43)	0.66667(12/18)	0.09091(2/22)	0.90000(18/20)
	Bo_TE_145060	0.00000(0/44)	0.26316(5/19)	0.00000(0/22)	0.95000(19/20)
	Bo_TE_209222	0.95455(42/44)	1.00000(18/18)	0.57143(12/21)	0.05263(1/19)
	Bo_TE_95777	0.88372(38/43)	1.00000(16/16)	0.33333(6/18)	0.00000(0/20)
	Bo_TE_43225	0.02273(1/44)	0.18750(3/16)	0.90909(20/22)	1.00000(18/18)

Bo_TE_167212	0.02222(1/45)	0.11111(2/18)	0.80952(17/21)	1.00000(19/19)
Bo_TE_9398	0.93023(40/43)	0.05882(1/17)	0.38095(8/21)	0.26316(5/19)
Bo_TE_137187	0.45238(19/42)	0.68750(11/16)	0.95455(21/22)	0.05000(1/20)
Bo_TE_75767	0.97727(43/44)	0.05263(1/19)	0.50000(11/22)	0.26316(5/19)
Bo_TE_117940	0.00000(0/43)	0.00000(0/17)	1.00000(20/20)	0.88889(16/18)
Bo_TE_13652	0.06667(3/45)	1.00000(16/16)	1.00000(19/19)	0.73684(14/19)
Bo_TE_117937	1.00000(44/44)	1.00000(19/19)	0.00000(0/19)	0.10526(2/19)
Bo_TE_92968	0.93182(41/44)	0.93750(15/16)	0.10000(2/20)	0.10000(2/20)
Bo_TE_147129	0.00000(0/45)	0.00000(0/18)	0.91304(21/23)	0.21053(4/19)
Bo_TE_93826	0.95238(40/42)	1.00000(19/19)	0.05000(1/20)	0.10526(2/19)
Bo_TE_65484	0.00000(0/46)	0.22222(4/18)	0.90476(19/21)	0.05263(1/19)
Bo_TE_21728	0.00000(0/44)	0.11111(2/18)	0.28571(6/21)	0.95000(19/20)
Bo_TE_211826	0.91111(41/45)	0.55556(10/18)	0.04545(1/22)	0.60000(12/20)
Bo_TE_226366	0.64865(24/37)	0.00000(0/17)	1.00000(22/22)	0.00000(0/20)
Bo_TE_96122	0.02381(1/42)	0.00000(0/19)	0.40000(8/20)	0.95000(19/20)
Bo_TE_76617	0.06977(3/43)	0.33333(6/18)	1.00000(23/23)	1.00000(20/20)
Bo_TE_148018	0.07143(3/42)	0.00000(0/16)	0.95652(22/23)	0.76471(13/17)
Bo_TE_196606	0.23256(10/43)	0.63158(12/19)	0.09524(2/21)	0.90000(18/20)
Bo_TE_136370	0.09524(4/42)	0.40000(2/5)	0.72222(13/18)	1.00000(19/19)
Bo_TE_205602	0.72973(27/37)	0.05882(1/17)	0.90909(20/22)	0.95000(19/20)
Bo_TE_198971	0.00000(0/46)	0.64286(9/14)	0.80952(17/21)	0.90000(18/20)
Bo_TE_15635	0.15556(7/45)	0.00000(0/17)	1.00000(22/22)	1.00000(20/20)
Bo_TE_53697	0.04348(2/46)	0.50000(9/18)	0.91304(21/23)	0.85000(17/20)
Bo_TE_73646	0.00000(0/45)	0.76471(13/17)	0.63158(12/19)	0.94118(16/17)
Bo_TE_201276	0.72727(32/44)	1.00000(18/18)	0.00000(0/22)	0.00000(0/19)
Bo_TE_220948	0.05000(2/40)	0.94444(17/18)	0.89474(17/19)	0.45000(9/20)
Bo_TE_112322	0.92857(39/42)	0.64706(11/17)	0.10000(2/20)	0.05263(1/19)
Bo_TE_221740	1.00000(42/42)	1.00000(12/12)	0.95238(20/21)	0.05882(1/17)
Bo_TE_39121	0.95349(41/43)	0.93750(15/16)	0.35000(7/20)	0.05000(1/20)
Bo_TE_2980	0.58140(25/43)	1.00000(18/18)	0.04545(1/22)	0.55000(11/20)
Bo_TE_156975	0.31707(13/41)	0.05882(1/17)	0.95455(21/22)	0.20000(4/20)
Bo_TE_113930	0.00000(0/44)	0.00000(0/18)	0.95238(20/21)	0.85000(17/20)
Bo_TE_41872	0.04444(2/45)	0.23529(4/17)	0.90000(18/20)	0.72222(13/18)
Bo_TE_7968	0.92683(38/41)	1.00000(15/15)	0.08696(2/23)	0.61538(8/13)
Bo_TE_215483	0.00000(0/43)	0.00000(0/19)	0.22727(5/22)	0.95000(19/20)
Bo_TE_144271	0.41463(17/41)	0.94118(16/17)	0.15000(3/20)	0.00000(0/19)
Bo_TE_63457	0.02174(1/46)	0.16667(3/18)	0.17391(4/23)	0.94737(18/19)
Bo_TE_18034	0.93333(42/45)	0.82353(14/17)	0.08696(2/23)	0.21053(4/19)
Bo_TE_118467	0.71111(32/45)	0.93750(15/16)	0.00000(0/23)	0.10000(2/20)
Bo_TE_29784	0.97619(41/42)	0.23529(4/17)	0.33333(7/21)	0.05263(1/19)
Bo_TE_76591	0.04651(2/43)	0.31579(6/19)	0.40909(9/22)	0.94737(18/19)
Bo_TE_8193	0.00000(0/44)	0.40000(6/15)	0.25000(5/20)	1.00000(20/20)
Bo_TE_83836	0.02500(1/40)	0.22222(4/18)	0.80000(16/20)	0.94737(18/19)
Bo_TE_13670	0.07895(3/38)	1.00000(17/17)	0.95455(21/22)	0.73684(14/19)
Bo_TE_136699	0.06818(3/44)	0.50000(9/18)	0.95455(21/22)	0.94444(17/18)
Bo_TE_156682	0.96000(24/25)	1.00000(17/17)	0.95455(21/22)	0.00000(0/16)
Bo_TE_215493	0.26190(11/42)	0.00000(0/19)	0.22727(5/22)	0.95000(19/20)
Bo_TE_156962	0.13953(6/43)	0.00000(0/17)	0.95238(20/21)	0.15000(3/20)
Bo_TE_48127	1.00000(45/45)	1.00000(14/14)	0.00000(0/22)	0.15789(3/19)
Bo_TE_209841	0.61364(27/44)	0.06250(1/16)	1.00000(22/22)	1.00000(19/19)
Bo_TE_183530	0.43590(17/39)	0.00000(0/19)	0.00000(0/18)	1.00000(19/19)
Bo_TE_138337	0.18605(8/43)	0.05556(1/18)	0.39130(9/23)	1.00000(19/19)
Bo_TE_230952	0.97778(44/45)	0.05882(1/17)	0.23810(5/21)	0.16667(3/18)
Bo_TE_33205	0.92857(39/42)	0.56250(9/16)	0.66667(14/21)	0.00000(0/20)
Bo_TE_22539	0.92857(39/42)	0.25000(4/16)	0.00000(0/23)	0.00000(0/20)
Bo_TE_122093	0.07895(3/38)	0.00000(0/19)	1.00000(22/22)	0.35000(7/20)

Bo_TE_117958	0.00000(0/44)	0.00000(0/18)	1.00000(19/19)	0.90000(18/20)
Bo_TE_234106	0.26190(11/42)	0.94118(16/17)	0.00000(0/22)	0.85000(17/20)
Bo_TE_201193	0.76190(32/42)	1.00000(18/18)	0.00000(0/22)	0.00000(0/20)
Bo_TE_33407	0.91304(42/46)	0.06250(1/16)	0.80952(17/21)	0.05000(1/20)
Bo_TE_92078	0.04878(2/41)	0.57143(8/14)	1.00000(23/23)	0.44444(8/18)
Bo_TE_179916	0.02273(1/44)	0.05263(1/19)	0.95000(19/20)	0.57895(11/19)
Bo_TE_53703	0.97727(43/44)	0.62500(10/16)	0.09524(2/21)	0.15789(3/19)
Bo_TE_228649	1.00000(45/45)	0.88889(16/18)	0.21053(4/19)	0.00000(0/17)
Bo_TE_94956	0.06667(3/45)	0.71429(10/14)	0.70000(14/20)	1.00000(19/19)
Bo_TE_4127	0.90909(40/44)	0.00000(0/18)	0.00000(0/23)	0.61111(11/18)
Bo_TE_4145	0.00000(0/44)	1.00000(17/17)	0.71429(15/21)	0.05263(1/19)
Bo_TE_47264	0.93478(43/46)	0.05882(1/17)	0.27273(6/22)	0.57895(11/19)
Bo_TE_142436	1.00000(43/43)	0.88235(15/17)	0.10000(2/20)	0.63158(12/19)
Bo_TE_40927	1.00000(46/46)	1.00000(18/18)	0.15000(3/20)	0.00000(0/19)
Bo_TE_23205	0.97674(42/43)	0.11111(2/18)	0.40000(8/20)	0.05556(1/18)
Bo_TE_59515	0.04651(2/43)	0.00000(0/16)	0.68182(15/22)	0.94737(18/19)
Bo_TE_45379	0.04348(2/46)	0.93750(15/16)	0.65000(13/20)	0.89474(17/19)
Bo_TE_67248	0.04348(2/46)	1.00000(18/18)	0.80952(17/21)	0.55000(11/20)
Bo_TE_201539	0.02222(1/45)	0.00000(0/18)	0.09091(2/22)	0.94737(18/19)
Bo_TE_196457	0.02174(1/46)	0.44444(8/18)	1.00000(21/21)	0.20000(4/20)
Bo_TE_183287	0.47500(19/40)	0.00000(0/17)	1.00000(22/22)	0.94737(18/19)
Bo_TE_144312	0.59459(22/37)	0.05263(1/19)	1.00000(23/23)	0.90000(18/20)
Bo_TE_39202	0.51220(21/41)	0.06250(1/16)	0.56522(13/23)	1.00000(17/17)
Bo_TE_197669	0.88095(37/42)	0.23529(4/17)	0.90476(19/21)	0.00000(0/18)
Bo_TE_35974	0.06522(3/46)	0.27778(5/18)	0.85714(18/21)	1.00000(20/20)
Bo_TE_209786	0.43902(18/41)	0.00000(0/16)	0.68182(15/22)	0.95000(19/20)
Bo_TE_144290	0.09524(4/42)	0.23529(4/17)	0.60000(12/20)	0.95000(19/20)
Bo_TE_77668	0.97778(44/45)	0.43750(7/16)	0.38095(8/21)	0.10000(2/20)
Bo_TE_198677	0.07692(3/39)	0.21053(4/19)	0.36842(7/19)	0.95000(19/20)
Bo_TE_52642	0.18605(8/43)	0.11765(2/17)	0.90000(18/20)	0.05000(1/20)
Bo_TE_39057	0.95556(43/45)	0.46667(7/15)	0.10000(2/20)	0.90000(18/20)
Bo_TE_39063	0.04444(2/45)	0.52941(9/17)	0.90476(19/21)	0.10000(2/20)
Bo_TE_164549	0.04878(2/41)	0.88235(15/17)	1.00000(23/23)	0.80000(16/20)
Bo_TE_45760	0.08889(4/45)	0.00000(0/19)	0.90476(19/21)	0.47368(9/19)
Bo_TE_227751	0.83721(36/43)	0.94118(16/17)	0.47619(10/21)	0.05000(1/20)
Bo_TE_15508	0.00000(0/41)	0.05556(1/18)	1.00000(19/19)	0.94118(16/17)
Bo_TE_165589	0.02632(1/38)	0.92857(13/14)	0.81818(9/11)	0.93750(15/16)
Bo_TE_56036	0.80000(32/40)	0.05882(1/17)	1.00000(21/21)	0.90000(18/20)
Bo_TE_196732	0.97727(43/44)	0.43750(7/16)	0.04762(1/21)	0.55556(10/18)
Bo_TE_167161	0.97619(41/42)	0.89474(17/19)	0.20000(4/20)	0.00000(0/19)
Bo_TE_93966	0.97727(43/44)	1.00000(18/18)	0.04762(1/21)	0.11111(2/18)
Bo_TE_59430	0.69767(30/43)	1.00000(16/16)	0.31818(7/22)	0.05263(1/19)
Bo_TE_92077	0.04651(2/43)	0.50000(7/14)	1.00000(22/22)	0.47368(9/19)
Bo_TE_237568	0.22500(9/40)	1.00000(17/17)	0.00000(0/22)	0.40000(8/20)
Bo_TE_214957	1.00000(45/45)	0.93750(15/16)	0.10000(2/20)	0.05263(1/19)
Bo_TE_145093	0.02222(1/45)	0.55556(10/18)	0.90476(19/21)	0.05000(1/20)
Bo_TE_13432	0.07500(3/40)	0.12500(2/16)	0.95455(21/22)	0.73684(14/19)
Bo_TE_222841	0.04444(2/45)	0.06250(1/16)	0.95455(21/22)	0.89474(17/19)
Bo_TE_51231	0.00000(0/41)	0.00000(0/19)	1.00000(21/21)	1.00000(20/20)
Bo_TE_203076	0.92105(35/38)	0.55556(10/18)	0.55000(11/20)	0.05263(1/19)
Bo_TE_113917	0.00000(0/42)	0.00000(0/18)	0.95238(20/21)	0.84211(16/19)
Bo_TE_211124	0.97778(44/45)	0.88889(16/18)	0.04762(1/21)	0.25000(5/20)
Bo_TE_205267	0.97778(44/45)	0.77778(14/18)	0.09524(2/21)	0.05000(1/20)
Bo_TE_73662	0.00000(0/46)	0.11111(2/18)	0.80000(16/20)	1.00000(18/18)
Bo_TE_201055	1.00000(46/46)	1.00000(18/18)	0.00000(0/22)	0.00000(0/20)
Bo_TE_59442	0.72093(31/43)	1.00000(17/17)	0.31818(7/22)	0.05000(1/20)

Bo_TE_203074	0.95455(42/44)	0.64706(11/17)	0.50000(9/18)	0.05000(1/20)
Bo_TE_104749	0.79070(34/43)	0.41176(7/17)	0.10000(2/20)	1.00000(20/20)
Bo_TE_205377	0.97368(37/38)	0.66667(6/9)	0.09091(2/22)	0.00000(0/17)
Bo_TE_87559	1.00000(44/44)	0.22222(4/18)	0.04545(1/22)	0.05263(1/19)
Bo_TE_145201	0.21429(9/42)	0.11111(2/18)	0.95238(20/21)	0.05000(1/20)
Bo_TE_73446	0.00000(0/43)	0.66667(12/18)	0.65000(13/20)	0.95000(19/20)
Bo_TE_228735	1.00000(45/45)	0.88889(16/18)	0.19048(4/21)	0.00000(0/17)
Bo_TE_196408	0.13636(6/44)	0.11765(2/17)	0.95455(21/22)	0.05000(1/20)
Bo_TE_191826	0.06818(3/44)	0.44444(8/18)	1.00000(23/23)	0.78947(15/19)
Bo_TE_177502	0.06667(3/45)	0.05263(1/19)	0.95238(20/21)	0.41176(7/17)
Bo_TE_48125	0.93333(42/45)	1.00000(17/17)	0.00000(0/23)	0.15789(3/19)
Bo_TE_59367	0.72093(31/43)	1.00000(17/17)	0.33333(6/18)	0.05263(1/19)
Bo_TE_211114	0.04444(2/45)	0.44444(8/18)	0.95238(20/21)	0.66667(12/18)
Bo_TE_118462	0.67442(29/43)	1.00000(17/17)	0.15000(3/20)	0.10000(2/20)
Bo_TE_211790	0.07143(3/42)	0.44444(8/18)	0.95000(19/20)	0.40000(8/20)
Bo_TE_235950	0.09756(4/41)	0.31250(5/16)	0.90909(20/22)	0.52632(10/19)
Bo_TE_179868	0.09091(4/44)	0.70588(12/17)	0.90000(18/20)	0.94737(18/19)
Bo_TE_147308	0.00000(0/46)	0.42105(8/19)	0.95652(22/23)	0.25000(5/20)
Bo_TE_148246	0.42500(17/40)	1.00000(17/17)	0.19048(4/21)	0.00000(0/17)
Bo_TE_201118	0.00000(0/44)	0.00000(0/19)	1.00000(22/22)	1.00000(19/19)
Bo_TE_73643	0.02222(1/45)	0.81250(13/16)	0.71429(15/21)	0.94118(16/17)
Bo_TE_130594	0.45455(20/44)	0.05556(1/18)	0.95455(21/22)	1.00000(20/20)
Bo_TE_223470	0.97778(44/45)	0.82353(14/17)	0.10000(2/20)	0.18750(3/16)
Bo_TE_144070	0.42500(17/40)	0.94737(18/19)	0.00000(0/22)	0.15000(3/20)
Bo_TE_15528	0.00000(0/46)	0.05556(1/18)	1.00000(21/21)	0.88889(16/18)
Bo_TE_238177	0.97619(41/42)	0.88235(15/17)	0.00000(0/23)	0.00000(0/20)
Bo_TE_234976	0.00000(0/45)	0.47059(8/17)	1.00000(22/22)	0.36842(7/19)
Bo_TE_45547	0.90698(39/43)	0.00000(0/18)	0.00000(0/21)	0.00000(0/19)
Bo_TE_206069	0.00000(0/43)	0.11111(2/18)	1.00000(23/23)	0.84211(16/19)
Bo_TE_160878	0.02273(1/44)	0.27778(5/18)	0.95238(20/21)	0.90000(18/20)
Bo_TE_154111	0.00000(0/45)	0.00000(0/17)	0.95000(19/20)	0.84211(16/19)
Bo_TE_76837	0.15909(7/44)	0.17647(3/17)	1.00000(23/23)	0.10000(2/20)
Bo_TE_222238	0.80000(32/40)	0.05556(1/18)	1.00000(23/23)	0.29412(5/17)
Bo_TE_53725	0.97674(42/43)	0.55556(10/18)	0.09091(2/22)	0.15000(3/20)
Bo_TE_168543	0.02326(1/43)	0.93750(15/16)	0.28571(6/21)	0.20000(4/20)
Bo_TE_82176	0.93333(42/45)	0.66667(12/18)	0.40000(8/20)	0.05263(1/19)
Bo_TE_30784	0.88636(39/44)	0.94118(16/17)	0.33333(7/21)	0.05263(1/19)
Bo_TE_48008	0.47500(19/40)	1.00000(17/17)	0.00000(0/23)	0.05000(1/20)
Bo_TE_183508	0.25000(10/40)	0.93750(15/16)	0.34783(8/23)	0.00000(0/20)
Bo_TE_118327	0.00000(0/41)	0.00000(0/19)	0.95238(20/21)	0.95000(19/20)
Bo_TE_161035	0.90698(39/43)	0.61111(11/18)	0.04762(1/21)	0.41176(7/17)
Bo_TE_43000	0.83721(36/43)	0.58824(10/17)	0.90476(19/21)	0.05263(1/19)
Bo_TE_224685	0.62500(25/40)	0.93750(15/16)	0.13636(3/22)	0.05000(1/20)
Bo_TE_90731	1.00000(43/43)	0.05882(1/17)	0.04348(1/23)	0.60000(12/20)
Bo_TE_180244	0.00000(0/45)	0.94444(17/18)	0.00000(0/22)	0.00000(0/20)
Bo_TE_228534	1.00000(42/42)	0.89474(17/19)	0.20000(4/20)	0.00000(0/19)
Bo_TE_88515	1.00000(44/44)	0.43750(7/16)	0.00000(0/22)	0.11111(2/18)
Bo_TE_29752	0.02326(1/43)	0.66667(12/18)	0.68182(15/22)	0.95000(19/20)
Bo_TE_222969	0.38636(17/44)	0.25000(4/16)	0.95000(19/20)	0.00000(0/20)
Bo_TE_38995	0.97674(42/43)	0.71429(10/14)	0.90909(20/22)	0.00000(0/20)
Bo_TE_196586	0.84615(33/39)	0.72222(13/18)	0.95652(22/23)	0.05263(1/19)
Bo_TE_196698	0.09091(4/44)	0.33333(6/18)	0.95455(21/22)	0.00000(0/18)
Bo_TE_144938	1.00000(43/43)	0.23529(4/17)	0.00000(0/18)	0.00000(0/17)
Bo_TE_154186	0.00000(0/45)	0.12500(2/16)	0.95652(22/23)	0.94444(17/18)
Bo_TE_237309	0.95455(42/44)	0.84211(16/19)	0.00000(0/23)	0.00000(0/17)
Bo_TE_178376	0.39474(15/38)	0.58824(10/17)	0.90476(19/21)	0.05000(1/20)

Bo_TE_238398	0.00000(0/41)	0.00000(0/18)	0.42857(9/21)	1.00000(17/17)
Bo_TE_46121	0.00000(0/46)	0.50000(9/18)	1.00000(21/21)	0.73684(14/19)
Bo_TE_88623	1.00000(41/41)	0.93333(14/15)	0.00000(0/20)	0.50000(9/18)
Bo_TE_51931	0.02439(1/41)	0.41176(7/17)	1.00000(21/21)	0.90000(18/20)
Bo_TE_167228	0.02222(1/45)	0.10526(2/19)	0.70588(12/17)	0.94444(17/18)
Bo_TE_161734	0.09091(4/44)	0.52941(9/17)	0.90000(18/20)	0.65000(13/20)
Bo_TE_239531	0.97727(43/44)	0.77778(14/18)	0.09524(2/21)	0.90000(18/20)
Bo_TE_88381	0.75610(31/41)	0.00000(0/17)	0.91304(21/23)	0.90000(18/20)
Bo_TE_93962	0.00000(0/43)	0.00000(0/19)	0.95238(20/21)	0.90000(18/20)
Bo_TE_148132	0.14286(6/42)	1.00000(18/18)	0.65000(13/20)	0.00000(0/19)
Bo_TE_48104	0.51282(20/39)	0.00000(0/17)	1.00000(23/23)	0.94737(18/19)
Bo_TE_90740	1.00000(41/41)	0.05556(1/18)	0.04348(1/23)	0.61111(11/18)
Bo_TE_6865	0.09091(4/44)	0.82353(14/17)	0.77273(17/22)	1.00000(20/20)
Bo_TE_180687	0.00000(0/45)	0.00000(0/18)	0.95455(21/22)	0.83333(15/18)
Bo_TE_140152	0.90476(38/42)	0.68750(11/16)	0.36364(8/22)	0.00000(0/19)
Bo_TE_181343	0.21053(8/38)	0.06250(1/16)	1.00000(19/19)	0.45000(9/20)
Bo_TE_15544	1.00000(45/45)	0.94444(17/18)	0.00000(0/21)	0.05000(1/20)
Bo_TE_56102	0.22222(4/18)	0.06250(1/16)	1.00000(22/22)	1.00000(12/12)
Bo_TE_8219	1.00000(42/42)	0.60000(9/15)	0.75000(15/20)	0.00000(0/19)
Bo_TE_154433	0.97674(42/43)	0.06250(1/16)	1.00000(23/23)	1.00000(20/20)
Bo_TE_36768	1.00000(37/37)	0.33333(6/18)	0.19048(4/21)	0.05556(1/18)
Bo_TE_48037	0.52632(20/38)	0.05556(1/18)	1.00000(21/21)	0.94737(18/19)
Bo_TE_222180	0.07500(3/40)	0.72222(13/18)	0.84211(16/19)	0.94737(18/19)
Bo_TE_149537	0.02326(1/43)	0.40000(6/15)	0.23529(4/17)	1.00000(20/20)
Bo_TE_45764	0.93478(43/46)	0.55556(10/18)	0.00000(0/19)	0.27778(5/18)
Bo_TE_181291	0.06667(3/45)	0.81250(13/16)	0.90000(18/20)	0.55000(11/20)
Bo_TE_39161	0.97727(43/44)	1.00000(17/17)	0.00000(0/10)	0.00000(0/17)
Bo_TE_67014	0.02500(1/40)	0.00000(0/16)	1.00000(23/23)	0.88235(15/17)
Bo_TE_62456	0.93333(42/45)	0.66667(10/15)	0.00000(0/23)	0.11111(2/18)
Bo_TE_141729	0.95455(42/44)	0.76471(13/17)	0.00000(0/21)	0.27778(5/18)
Bo_TE_54517	0.04348(2/46)	0.50000(9/18)	0.38095(8/21)	0.94118(16/17)
Bo_TE_201038	0.97826(45/46)	0.23529(4/17)	0.00000(0/20)	0.00000(0/19)
Bo_TE_88390	0.02222(1/45)	0.00000(0/16)	0.90476(19/21)	0.81250(13/16)
Bo_TE_228823	0.02326(1/43)	0.10526(2/19)	0.73684(14/19)	1.00000(16/16)
Bo_TE_216026	0.09302(4/43)	0.37500(6/16)	0.85714(18/21)	0.94737(18/19)
Bo_TE_196483	0.95455(42/44)	0.55556(10/18)	0.00000(0/22)	0.00000(0/20)
Bo_TE_116263	0.97674(42/43)	0.36842(7/19)	0.00000(0/22)	0.40000(8/20)
Bo_TE_63068	0.06522(3/46)	0.62500(10/16)	1.00000(22/22)	1.00000(20/20)
Bo_TE_89632	0.02381(1/42)	0.00000(0/17)	0.61905(13/21)	0.94737(18/19)
Bo_TE_39177	0.31111(14/45)	0.00000(0/9)	0.90909(20/22)	0.00000(0/20)
Bo_TE_127315	0.00000(0/45)	0.11111(2/18)	1.00000(21/21)	0.85000(17/20)
Bo_TE_1642	0.45238(19/42)	0.00000(0/17)	0.26087(6/23)	1.00000(19/19)
Bo_TE_66465	0.51220(21/41)	0.22222(4/18)	0.10000(2/20)	1.00000(19/19)
Bo_TE_67200	0.04348(2/46)	1.00000(19/19)	0.82609(19/23)	0.52632(10/19)
Bo_TE_149358	0.02273(1/44)	0.29412(5/17)	1.00000(21/21)	0.55000(11/20)
Bo_TE_220356	1.00000(42/42)	0.47059(8/17)	0.38095(8/21)	0.10000(2/20)
Bo_TE_4514	0.09375(3/32)	0.00000(0/18)	0.95000(19/20)	0.50000(9/18)
Bo_TE_83637	0.04348(2/46)	0.25000(4/16)	0.95652(22/23)	0.47368(9/19)
Bo_TE_167582	0.97778(44/45)	0.88889(16/18)	0.23529(4/17)	0.00000(0/18)
Bo_TE_22552	0.97368(37/38)	0.25000(4/16)	0.00000(0/22)	0.00000(0/20)
Bo_TE_90726	1.00000(45/45)	0.05882(1/17)	0.04545(1/22)	0.63158(12/19)
Bo_TE_235373	0.08889(4/45)	0.12500(2/16)	1.00000(23/23)	0.57895(11/19)
Bo_TE_178054	0.05000(2/40)	0.88235(15/17)	1.00000(22/22)	0.57895(11/19)
Bo_TE_15191	0.00000(0/40)	0.00000(0/17)	0.90909(20/22)	0.89474(17/19)
Bo_TE_205195	0.95455(42/44)	0.27778(5/18)	0.10526(2/19)	0.05000(1/20)
Bo_TE_70953	0.04348(2/46)	0.05556(1/18)	0.95000(19/20)	0.64706(11/17)

Bo_TE_8139	0.93478(43/46)	0.23529(4/17)	0.00000(0/23)	0.00000(0/20)
Bo_TE_211829	0.93182(41/44)	0.57895(11/19)	0.04762(1/21)	0.60000(12/20)
Bo_TE_194450	0.13636(6/44)	0.00000(0/18)	0.14286(3/21)	0.90000(18/20)
Bo_TE_82732	0.93182(41/44)	0.80000(12/15)	0.04545(1/22)	0.05263(1/19)
Bo_TE_70100	0.92857(39/42)	0.05882(1/17)	0.76190(16/21)	0.35000(7/20)
Bo_TE_173761	0.06977(3/43)	0.00000(0/18)	0.38095(8/21)	0.90000(18/20)
Bo_TE_89646	0.97778(44/45)	1.00000(19/19)	0.38095(8/21)	0.10000(2/20)
Bo_TE_178656	0.37209(16/43)	0.00000(0/18)	0.00000(0/23)	0.90000(18/20)
Bo_TE_149270	0.94595(35/37)	0.84615(11/13)	0.00000(0/19)	0.00000(0/1)
Bo_TE_63456	0.02174(1/46)	0.16667(3/18)	0.17391(4/23)	0.95000(19/20)
Bo_TE_153412	0.00000(0/44)	0.29412(5/17)	1.00000(21/21)	0.94444(17/18)
Bo_TE_148225	0.44186(19/43)	1.00000(17/17)	0.25000(5/20)	0.00000(0/20)
Bo_TE_145661	0.04348(2/46)	0.31250(5/16)	1.00000(22/22)	1.00000(20/20)
Bo_TE_88121	0.92857(39/42)	0.47059(8/17)	0.00000(0/23)	0.00000(0/20)
Bo_TE_223480	0.02222(1/45)	0.18750(3/16)	0.90000(18/20)	0.77778(14/18)
Bo_TE_46616	0.97727(43/44)	0.94118(16/17)	0.00000(0/23)	0.50000(10/20)
Bo_TE_117966	1.00000(45/45)	1.00000(18/18)	0.00000(0/22)	0.10000(2/20)
Bo_TE_158700	0.02500(1/40)	0.94118(16/17)	0.16667(3/18)	0.00000(0/18)
Bo_TE_38983	0.97727(43/44)	0.05882(1/17)	0.90476(19/21)	0.00000(0/20)
Bo_TE_198527	0.04651(2/43)	0.31579(6/19)	0.38095(8/21)	0.95000(19/20)
Bo_TE_101760	0.02222(1/45)	0.58824(10/17)	0.00000(0/21)	1.00000(19/19)
Bo_TE_156679	0.52273(23/44)	0.94444(17/18)	0.95455(21/22)	0.00000(0/20)
Bo_TE_58405	0.90698(39/43)	1.00000(19/19)	0.09091(2/22)	0.80000(16/20)
Bo_TE_67190	0.04545(2/44)	1.00000(17/17)	0.81818(18/22)	0.55000(11/20)
Bo_TE_35976	0.06977(3/43)	0.26316(5/19)	0.80952(17/21)	1.00000(18/18)
Bo_TE_113900	1.00000(46/46)	0.94737(18/19)	0.04545(1/22)	0.15000(3/20)
Bo_TE_13504	0.92683(38/41)	0.86667(13/15)	0.00000(0/22)	0.21053(4/19)
Bo_TE_207062	0.31707(13/41)	0.47059(8/17)	1.00000(22/22)	0.00000(0/19)
Bo_TE_48068	0.46341(19/41)	0.94118(16/17)	0.00000(0/22)	0.05000(1/20)
Bo_TE_30738	0.11364(5/44)	0.00000(0/16)	0.65000(13/20)	0.94737(18/19)
Bo_TE_222729	0.91111(41/45)	0.68421(13/19)	0.00000(0/23)	0.05882(1/17)
Bo_TE_97610	0.09091(4/44)	0.26667(4/15)	1.00000(22/22)	0.11111(2/18)
Bo_TE_160856	0.00000(0/40)	0.35294(6/17)	0.95000(19/20)	0.95000(19/20)
Bo_TE_88635	0.16667(7/42)	0.06250(1/16)	1.00000(20/20)	0.84211(16/19)
Bo_TE_237198	0.90244(37/41)	0.83333(15/18)	0.00000(0/23)	0.00000(0/18)
Bo_TE_4360	0.04545(2/44)	0.31250(5/16)	0.95455(21/22)	0.90000(18/20)
Bo_TE_22598	0.93182(41/44)	0.25000(4/16)	0.00000(0/23)	0.00000(0/20)
Bo_TE_154108	0.00000(0/43)	0.00000(0/18)	0.95455(21/22)	0.83333(15/18)
Bo_TE_48144	1.00000(45/45)	1.00000(19/19)	0.00000(0/23)	0.00000(0/18)
Bo_TE_56066	0.02273(1/44)	0.94444(17/18)	0.00000(0/23)	0.10526(2/19)
Bo_TE_48062	0.54054(20/37)	1.00000(17/17)	0.00000(0/20)	0.06250(1/16)
Bo_TE_118461	0.69048(29/42)	1.00000(18/18)	0.10000(2/20)	0.10000(2/20)
Bo_TE_144284	0.00000(0/42)	0.00000(0/19)	0.60000(12/20)	0.94444(17/18)
Bo_TE_121479	0.62791(27/43)	1.00000(18/18)	0.66667(14/21)	0.10000(2/20)
Bo_TE_145660	0.95455(42/44)	0.75000(12/16)	0.00000(0/21)	0.00000(0/20)
Bo_TE_169499	0.11628(5/43)	0.00000(0/17)	1.00000(22/22)	1.00000(20/20)
Bo_TE_95770	0.26667(12/45)	0.05882(1/17)	0.80000(16/20)	1.00000(20/20)
Bo_TE_136013	1.00000(46/46)	1.00000(18/18)	0.90909(20/22)	0.10000(2/20)
Bo_TE_142469	0.02326(1/43)	0.05263(1/19)	0.90909(20/22)	0.63158(12/19)
Bo_TE_235473	0.02222(1/45)	0.23529(4/17)	1.00000(23/23)	0.60000(12/20)
Bo_TE_107921	0.00000(0/45)	0.93750(15/16)	0.21429(3/14)	0.00000(0/18)
Bo_TE_167515	0.97674(42/43)	0.88235(15/17)	0.20000(4/20)	0.00000(0/18)
Bo_TE_65505	0.04762(2/42)	0.31579(6/19)	0.90909(20/22)	0.00000(0/20)
Bo_TE_180684	0.93182(41/44)	1.00000(17/17)	0.04762(1/21)	0.15000(3/20)
Bo_TE_75783	0.97727(43/44)	0.05882(1/17)	0.54545(12/22)	0.27778(5/18)
Bo_TE_58238	0.34146(14/41)	0.13333(2/15)	0.95238(20/21)	0.05000(1/20)

Bo_TE_230552	0.86047(37/43)	0.94118(16/17)	0.30000(6/20)	0.05556(1/18)
Bo_TE_29740	0.00000(0/46)	0.76471(13/17)	0.65000(13/20)	0.95000(19/20)
Bo_TE_53591	0.02222(1/45)	0.00000(0/18)	0.73684(14/19)	0.90000(18/20)
Bo_TE_237163	0.00000(0/45)	0.21053(4/19)	0.95000(19/20)	0.89474(17/19)
Bo_TE_149328	1.00000(39/39)	0.77778(14/18)	0.80952(17/21)	0.05000(1/20)
Bo_TE_118263	0.97727(43/44)	0.75000(12/16)	0.00000(0/22)	0.00000(0/20)
Bo_TE_160875	0.95349(41/43)	0.80000(12/15)	0.04762(1/21)	0.10000(2/20)
Bo_TE_238386	0.19444(7/36)	0.00000(0/18)	0.90476(19/21)	1.00000(20/20)
Bo_TE_201024	1.00000(46/46)	1.00000(18/18)	0.00000(0/21)	0.00000(0/20)
Bo_TE_90715	0.00000(0/45)	0.94118(16/17)	0.95455(21/22)	0.35000(7/20)
Bo_TE_70056	0.06818(3/44)	0.94444(17/18)	0.20000(4/20)	0.63158(12/19)
Bo_TE_235851	0.06667(3/45)	0.41176(7/17)	0.45455(10/22)	0.95000(19/20)
Bo_TE_30513	0.76316(29/38)	0.94737(18/19)	0.00000(0/19)	0.77778(14/18)
Bo_TE_30545	0.28571(12/42)	0.00000(0/16)	0.95455(21/22)	0.35000(7/20)
Bo_TE_89696	0.97674(42/43)	0.61111(11/18)	0.00000(0/22)	0.05263(1/19)
Bo_TE_102146	0.91111(41/45)	0.94118(16/17)	0.28571(6/21)	0.10000(2/20)
Bo_TE_97320	0.93478(43/46)	0.55556(10/18)	0.08696(2/23)	0.10526(2/19)
Bo_TE_89571	0.55814(24/43)	0.94118(16/17)	0.70000(7/10)	0.00000(0/18)
Bo_TE_177951	0.92857(39/42)	0.25000(4/16)	0.14286(3/21)	0.10000(2/20)
Bo_TE_211517	0.91111(41/45)	0.43750(7/16)	0.80000(16/20)	0.10000(2/20)
Bo_TE_40960	0.00000(0/46)	0.00000(0/17)	0.80000(16/20)	1.00000(19/19)
Bo_TE_167544	0.02326(1/43)	0.05556(1/18)	0.75000(15/20)	0.95000(19/20)
Bo_TE_172139	0.90698(39/43)	0.82353(14/17)	0.05000(1/20)	0.15000(3/20)
Bo_TE_112479	0.10870(5/46)	0.25000(4/16)	0.95238(20/21)	0.05000(1/20)
Bo_TE_39224	0.07143(3/42)	0.00000(0/15)	0.95652(22/23)	0.31579(6/19)
Bo_TE_228533	1.00000(44/44)	0.94737(18/19)	0.20000(4/20)	0.00000(0/19)
Bo_TE_132932	0.92683(38/41)	0.73684(14/19)	0.15789(3/19)	0.10000(2/20)
Bo_TE_180062	0.26190(11/42)	0.00000(0/19)	0.35000(7/20)	1.00000(18/18)
Bo_TE_56071	0.80000(32/40)	0.05556(1/18)	1.00000(22/22)	0.83333(15/18)
Bo_TE_201486	0.07317(3/41)	0.77778(14/18)	1.00000(22/22)	0.77778(14/18)
Bo_TE_101333	1.00000(40/40)	0.26667(4/15)	0.55556(10/18)	0.05882(1/17)
Bo_TE_205476	1.00000(44/44)	0.88235(15/17)	0.04762(1/21)	0.00000(0/20)
Bo_TE_107870	0.90909(40/44)	0.11765(2/17)	0.00000(0/22)	0.05000(1/20)
Bo_TE_230985	0.17073(7/41)	0.05882(1/17)	1.00000(23/23)	0.95000(19/20)
Bo_TE_87395	0.00000(0/45)	0.11111(2/18)	0.95455(21/22)	0.90000(18/20)
Bo_TE_150532	0.04545(2/44)	0.47059(8/17)	0.90909(20/22)	0.45000(9/20)
Bo_TE_236304	0.02273(1/44)	0.05263(1/19)	0.71429(15/21)	0.90000(18/20)
Bo_TE_161893	0.39474(15/38)	0.06250(1/16)	1.00000(21/21)	0.70000(14/20)
Bo_TE_235658	0.18605(8/43)	0.06250(1/16)	0.91304(21/23)	0.35294(6/17)
Bo_TE_41151	0.30000(12/40)	0.16667(3/18)	0.95000(19/20)	0.10000(2/20)
Bo_TE_200363	0.02439(1/41)	0.50000(3/6)	0.91304(21/23)	0.81818(9/11)
Bo_TE_145084	0.97778(44/45)	0.63158(12/19)	0.90476(19/21)	0.10000(2/20)
Bo_TE_155225	0.86364(38/44)	1.00000(17/17)	0.09524(2/21)	0.27778(5/18)
Bo_TE_138142	0.04444(2/45)	0.71429(10/14)	0.95652(22/23)	0.50000(10/20)
Bo_TE_95833	0.69048(29/42)	0.94444(17/18)	0.00000(0/21)	0.00000(0/20)
Bo_TE_107960	0.95349(41/43)	0.73684(14/19)	0.00000(0/21)	0.17647(3/17)
Bo_TE_29833	1.00000(46/46)	0.23529(4/17)	0.31818(7/22)	0.05000(1/20)
Bo_TE_230471	0.55814(24/43)	1.00000(18/18)	0.89474(17/19)	0.05556(1/18)
Bo_TE_224719	0.46341(19/41)	0.94118(16/17)	0.00000(0/23)	0.05000(1/20)
Bo_TE_46127	0.02500(1/40)	0.50000(9/18)	1.00000(22/22)	0.73684(14/19)
Bo_TE_92943	0.88636(39/44)	1.00000(18/18)	0.09524(2/21)	0.00000(0/20)
Bo_TE_21734	0.02564(1/39)	0.23529(4/17)	0.26316(5/19)	1.00000(20/20)
Bo_TE_17684	0.00000(0/45)	0.05882(1/17)	0.77273(17/22)	0.95000(19/20)
Bo_TE_129500	0.86364(38/44)	0.94444(17/18)	0.90476(19/21)	0.00000(0/19)
Bo_TE_60236	0.07500(3/40)	0.42857(6/14)	0.63158(12/19)	1.00000(17/17)
Bo_TE_144893	0.04878(2/41)	0.72727(8/11)	1.00000(22/22)	0.44444(8/18)

Bo_TE_237559	0.08889(4/45)	0.00000(0/16)	0.95238(20/21)	0.78947(15/19)
Bo_TE_235607	0.51220(21/41)	0.00000(0/16)	0.95652(22/23)	0.85000(17/20)
Bo_TE_100352	0.02273(1/44)	0.00000(0/19)	1.00000(22/22)	0.89474(17/19)
Bo_TE_205438	1.00000(43/43)	0.92857(13/14)	0.10000(2/20)	0.00000(0/19)
Bo_TE_31739	0.09091(4/44)	1.00000(16/16)	0.35000(7/20)	0.00000(0/20)
Bo_TE_123288	0.00000(0/43)	0.05556(1/18)	0.95238(20/21)	0.55000(11/20)
Bo_TE_179239	0.50000(20/40)	0.31250(5/16)	0.95238(20/21)	0.00000(0/20)
Bo_TE_144970	1.00000(39/39)	0.85714(12/14)	0.15000(3/20)	0.06250(1/16)
Bo_TE_22541	0.06818(3/44)	0.76471(13/17)	1.00000(22/22)	1.00000(19/19)
Bo_TE_48065	0.47619(20/42)	1.00000(17/17)	0.00000(0/23)	0.05000(1/20)
Bo_TE_221546	0.77143(27/35)	0.94118(16/17)	0.04348(1/23)	0.00000(0/20)
Bo_TE_112470	0.61905(26/42)	1.00000(18/18)	0.09091(2/22)	0.90000(18/20)
Bo_TE_130250	0.82222(37/45)	1.00000(17/17)	0.04545(1/22)	0.21053(4/19)
Bo_TE_149335	1.00000(42/42)	0.78947(15/19)	0.80952(17/21)	0.05000(1/20)
Bo_TE_22574	0.95455(42/44)	0.25000(4/16)	0.00000(0/22)	0.00000(0/20)
Bo_TE_63461	0.90244(37/41)	0.81250(13/16)	0.60000(12/20)	0.05263(1/19)
Bo_TE_101961	0.09524(4/42)	0.76471(13/17)	0.90909(20/22)	0.45000(9/20)
Bo_TE_230949	0.09302(4/43)	0.05882(1/17)	0.95238(20/21)	0.78947(15/19)
Bo_TE_201033	0.00000(0/44)	0.00000(0/19)	1.00000(19/19)	1.00000(20/20)
Bo_TE_45708	0.33333(15/45)	1.00000(18/18)	0.04762(1/21)	0.26316(5/19)
Bo_TE_145033	0.97778(44/45)	0.73333(11/15)	0.90476(19/21)	0.05263(1/19)
Bo_TE_269	0.94737(36/38)	0.53333(8/15)	0.80952(17/21)	0.05000(1/20)
Bo_TE_237570	0.70455(31/44)	0.00000(0/17)	0.95455(21/22)	0.61111(11/18)
Bo_TE_222829	0.95122(39/41)	0.58824(10/17)	0.00000(0/23)	0.42105(8/19)
Bo_TE_85704	0.06667(3/45)	0.77778(14/18)	0.90000(18/20)	0.72222(13/18)
Bo_TE_66223	0.06977(3/43)	1.00000(16/16)	0.45000(9/20)	0.11765(2/17)
Bo_TE_237373	0.02273(1/44)	0.05882(1/17)	0.90000(18/20)	0.94444(17/18)
Bo_TE_205317	0.97826(45/46)	0.82353(14/17)	0.09091(2/22)	0.05000(1/20)
Bo_TE_9856	0.93182(41/44)	0.18750(3/16)	0.00000(0/21)	0.05263(1/19)
Bo_TE_88523	0.00000(0/44)	0.12500(2/16)	1.00000(23/23)	0.84211(16/19)
Bo_TE_6866	0.09091(4/44)	0.81250(13/16)	0.73684(14/19)	1.00000(19/19)
Bo_TE_181248	0.04545(2/44)	0.75000(12/16)	1.00000(22/22)	0.65000(13/20)
Bo_TE_101702	0.82927(34/41)	0.88889(16/18)	0.90476(19/21)	0.00000(0/19)
Bo_TE_89593	0.39024(16/41)	0.93750(15/16)	0.86364(19/22)	0.05263(1/19)
Bo_TE_45238	0.47059(16/34)	0.43750(7/16)	0.90476(19/21)	0.00000(0/17)
Bo_TE_60812	0.97778(44/45)	0.94118(16/17)	0.09091(2/22)	0.31579(6/19)
Bo_TE_92630	0.08108(3/37)	0.93750(15/16)	0.66667(12/18)	0.29412(5/17)
Bo_TE_136697	0.00000(0/42)	0.15789(3/19)	1.00000(21/21)	0.73684(14/19)
Bo_TE_5989	0.71429(30/42)	0.94118(16/17)	0.73684(14/19)	0.00000(0/20)
Bo_TE_180220	1.00000(43/43)	0.05556(1/18)	0.61905(13/21)	0.55000(11/20)
Bo_TE_13641	0.91111(41/45)	0.00000(0/16)	0.00000(0/22)	0.30000(6/20)
Bo_TE_92938	0.00000(0/44)	0.00000(0/18)	0.77778(14/18)	0.94737(18/19)
Bo_TE_46122	0.00000(0/44)	0.47368(9/19)	1.00000(22/22)	0.75000(15/20)
Bo_TE_31745	0.08889(4/45)	1.00000(18/18)	0.26316(5/19)	0.00000(0/19)
Bo_TE_236049	0.97727(43/44)	0.05882(1/17)	0.00000(0/22)	0.00000(0/19)
Bo_TE_167149	0.02222(1/45)	0.11111(2/18)	0.80000(16/20)	1.00000(18/18)
Bo_TE_61386	0.56410(22/39)	0.05556(1/18)	0.90909(20/22)	0.84211(16/19)
Bo_TE_2527	0.40476(17/42)	0.18750(3/16)	0.95652(22/23)	0.00000(0/20)
Bo_TE_30553	0.28261(13/46)	0.05556(1/18)	1.00000(23/23)	0.40000(8/20)
Bo_TE_191398	0.00000(0/46)	0.00000(0/19)	0.27273(6/22)	0.90000(18/20)
Bo_TE_235857	0.00000(0/42)	0.00000(0/8)	1.00000(19/19)	1.00000(19/19)
Bo_TE_196585	0.19512(8/41)	0.29412(5/17)	0.04545(1/22)	0.90000(18/20)
Bo_TE_12766	0.93023(40/43)	0.06250(1/16)	0.80952(17/21)	0.05000(1/20)
Bo_TE_89677	0.00000(0/43)	0.36842(7/19)	0.63636(14/22)	0.94444(17/18)
Bo_TE_173278	0.21429(9/42)	0.41176(7/17)	0.04762(1/21)	0.95000(19/20)
Bo_TE_80033	0.11111(5/45)	0.94118(16/17)	0.00000(0/20)	0.20000(4/20)

Bo_TE_154292	0.07143(3/42)	1.00000(16/16)	0.00000(0/23)	0.00000(0/20)
Bo_TE_67199	0.95455(42/44)	0.00000(0/18)	0.18182(4/22)	0.47368(9/19)
Bo_TE_67926	0.90000(36/40)	0.00000(0/17)	0.10000(2/20)	0.00000(0/19)
Bo_TE_24372	0.02222(1/45)	0.61111(11/18)	0.95455(21/22)	0.80000(16/20)
Bo_TE_57428	0.90698(39/43)	0.05556(1/18)	0.00000(0/22)	0.15789(3/19)
Bo_TE_214955	1.00000(45/45)	0.86667(13/15)	0.14286(3/21)	0.05263(1/19)
Bo_TE_205454	0.00000(0/41)	0.00000(0/18)	0.91304(21/23)	0.90000(18/20)
Bo_TE_91876	1.00000(41/41)	0.94737(18/19)	0.00000(0/20)	0.05000(1/20)
Bo_TE_33392	0.06667(3/45)	0.93750(15/16)	0.20000(4/20)	0.94737(18/19)
Bo_TE_33411	0.97619(41/42)	0.00000(0/14)	0.25000(5/20)	0.05000(1/20)
Bo_TE_198824	0.93182(41/44)	0.11111(2/18)	0.00000(0/20)	0.00000(0/20)
Bo_TE_172578	0.95122(39/41)	0.00000(0/18)	0.00000(0/22)	0.00000(0/19)
Bo_TE_61384	0.47222(17/36)	0.94444(17/18)	0.09524(2/21)	0.15789(3/19)
Bo_TE_77498	0.07692(3/39)	0.66667(10/15)	0.61905(13/21)	1.00000(18/18)
Bo_TE_169717	0.93023(40/43)	0.25000(4/16)	0.04762(1/21)	0.17647(3/17)
Bo_TE_201480	0.47619(20/42)	0.05882(1/17)	0.08696(2/23)	0.90000(18/20)
Bo_TE_95389	0.25581(11/43)	1.00000(17/17)	0.04545(1/22)	0.05000(1/20)
Bo_TE_93958	0.02273(1/44)	0.00000(0/19)	0.90476(19/21)	0.90000(18/20)
Bo_TE_214944	1.00000(45/45)	1.00000(17/17)	0.13636(3/22)	0.05000(1/20)
Bo_TE_117933	1.00000(43/43)	1.00000(18/18)	0.04762(1/21)	0.10526(2/19)
Bo_TE_31781	0.90476(38/42)	0.00000(0/17)	0.66667(14/21)	1.00000(20/20)
Bo_TE_92475	0.04651(2/43)	0.57895(11/19)	0.95000(19/20)	0.84211(16/19)
Bo_TE_215380	0.97500(39/40)	0.29412(5/17)	0.04348(1/23)	0.10000(2/20)
Bo_TE_183974	0.33333(12/36)	0.94118(16/17)	0.17647(3/17)	0.05000(1/20)
Bo_TE_144300	0.00000(0/45)	0.00000(0/18)	1.00000(12/12)	0.95000(19/20)
Bo_TE_237192	0.92857(39/42)	0.94118(16/17)	0.00000(0/21)	0.05000(1/20)
Bo_TE_228736	0.97727(43/44)	0.88889(16/18)	0.20000(4/20)	0.00000(0/19)
Bo_TE_237347	0.00000(0/45)	0.41176(7/17)	1.00000(22/22)	0.63158(12/19)
Bo_TE_215944	0.04651(2/43)	0.64706(11/17)	0.95455(21/22)	0.84211(16/19)
Bo_TE_29826	1.00000(44/44)	0.23529(4/17)	0.33333(7/21)	0.05000(1/20)
Bo_TE_33196	0.06522(3/46)	0.40000(6/15)	0.36364(8/22)	1.00000(20/20)
Bo_TE_9897	0.92857(39/42)	0.12500(2/16)	0.00000(0/17)	0.05882(1/17)
Bo_TE_178027	0.04545(2/44)	0.70588(12/17)	1.00000(23/23)	0.70588(12/17)
Bo_TE_118341	0.00000(0/45)	0.00000(0/19)	0.95238(20/21)	0.94737(18/19)
Bo_TE_67168	0.06667(3/45)	1.00000(18/18)	0.81818(18/22)	0.52632(10/19)
Bo_TE_223351	0.51351(19/37)	0.94118(16/17)	0.05263(1/19)	0.11111(2/18)
Bo_TE_73357	0.00000(0/42)	0.77778(14/18)	0.66667(14/21)	0.95000(19/20)
Bo_TE_145053	0.95556(43/45)	0.00000(0/17)	0.00000(0/23)	0.00000(0/20)
Bo_TE_230942	0.00000(0/40)	1.00000(16/16)	0.09091(1/11)	0.00000(0/14)
Bo_TE_146891	0.90698(39/43)	1.00000(17/17)	0.81818(18/22)	0.00000(0/17)
Bo_TE_205322	0.97727(43/44)	0.75000(12/16)	0.09524(2/21)	0.05263(1/19)
Bo_TE_46614	0.97674(42/43)	1.00000(15/15)	0.00000(0/21)	0.55556(10/18)
Bo_TE_92601	0.04545(2/44)	0.26667(4/15)	1.00000(21/21)	0.80000(16/20)
Bo_TE_224733	0.14634(6/41)	0.00000(0/17)	0.91304(21/23)	0.93750(15/16)
Bo_TE_40929	0.00000(0/46)	0.00000(0/18)	0.80000(16/20)	1.00000(17/17)
Bo_TE_61794	0.00000(0/41)	0.00000(0/18)	0.31818(7/22)	0.94737(18/19)
Bo_TE_12759	0.93333(42/45)	0.06667(1/15)	0.82609(19/23)	0.05000(1/20)
Bo_TE_53701	0.02381(1/42)	0.44444(8/18)	0.90909(20/22)	0.85000(17/20)
Bo_TE_161091	0.09091(4/44)	0.50000(9/18)	1.00000(23/23)	0.57895(11/19)
Bo_TE_39167	0.00000(0/43)	0.00000(0/17)	0.90909(20/22)	0.05000(1/20)
Bo_TE_39028	0.02326(1/43)	0.00000(0/7)	0.08696(2/23)	0.94737(18/19)
Bo_TE_235844	0.06818(3/44)	0.43750(7/16)	0.50000(11/22)	1.00000(19/19)
Bo_TE_238661	0.67500(27/40)	1.00000(16/16)	0.00000(0/22)	0.15000(3/20)
Bo_TE_82926	0.91111(41/45)	0.11765(2/17)	0.00000(0/22)	0.00000(0/20)
Bo_TE_169621	0.05000(2/40)	0.00000(0/18)	0.50000(11/22)	0.95000(19/20)
Bo_TE_16194	0.59524(25/42)	0.94118(16/17)	0.09524(2/21)	0.33333(6/18)

Bo_TE_202779	0.92683(38/41)	0.55556(10/18)	0.00000(0/13)	0.05556(1/18)
Bo_TE_3355	0.00000(0/46)	0.11111(2/18)	0.91304(21/23)	0.11111(2/18)
Bo_TE_53750	0.06818(3/44)	0.13333(2/15)	0.80000(16/20)	1.00000(19/19)
Bo_TE_237652	0.65116(28/43)	0.05882(1/17)	0.91304(21/23)	0.27778(5/18)
Bo_TE_113924	0.09302(4/43)	0.22222(4/18)	1.00000(23/23)	0.95000(19/20)
Bo_TE_13606	0.09091(4/44)	1.00000(18/18)	1.00000(21/21)	0.70588(12/17)
Bo_TE_9802	0.90909(40/44)	0.25000(4/16)	0.04545(1/22)	0.21053(4/19)
Bo_TE_167422	0.02326(1/43)	0.11111(2/18)	0.80000(16/20)	1.00000(18/18)
Bo_TE_30725	0.86364(38/44)	0.94118(16/17)	0.31818(7/22)	0.05556(1/18)
Bo_TE_38977	0.02500(1/40)	0.41176(7/17)	0.10000(2/20)	1.00000(20/20)
Bo_TE_221778	0.00000(0/46)	0.31250(5/16)	0.05000(1/20)	0.90000(18/20)
Bo_TE_97672	0.08696(4/46)	0.82353(14/17)	0.04545(1/22)	0.90000(18/20)
Bo_TE_209781	0.57500(23/40)	0.94444(17/18)	0.31818(7/22)	0.00000(0/20)
Bo_TE_70096	0.93182(41/44)	0.05556(1/18)	0.75000(15/20)	0.36842(7/19)
Bo_TE_92479	0.04545(2/44)	0.58824(10/17)	0.90000(18/20)	0.85000(17/20)
Bo_TE_89400	0.35556(16/45)	0.63158(12/19)	0.10000(2/20)	1.00000(19/19)
Bo_TE_96147	0.07143(3/42)	0.17647(3/17)	0.38095(8/21)	1.00000(19/19)
Bo_TE_223137	0.27273(12/44)	0.11111(2/18)	0.04348(1/23)	0.94444(17/18)
Bo_TE_10039	0.00000(0/42)	0.00000(0/18)	0.95238(20/21)	0.78947(15/19)
Bo_TE_80018	0.08889(4/45)	1.00000(18/18)	0.09091(2/22)	0.16667(3/18)
Bo_TE_126083	1.00000(42/42)	1.00000(17/17)	0.00000(0/22)	0.50000(10/20)
Bo_TE_228306	0.00000(0/45)	0.00000(0/18)	0.80952(17/21)	1.00000(19/19)
Bo_TE_142452	0.95122(39/41)	0.94118(16/17)	0.09091(2/22)	0.36842(7/19)
Bo_TE_226901	0.90698(39/43)	0.41176(7/17)	0.00000(0/23)	0.15000(3/20)
Bo_TE_167206	0.02273(1/44)	0.11111(2/18)	0.89474(17/19)	1.00000(19/19)
Bo_TE_237376	0.02381(1/42)	0.00000(0/16)	0.91304(21/23)	0.72727(8/11)
Bo_TE_148057	0.58537(24/41)	0.93750(15/16)	0.00000(0/22)	0.10000(2/20)
Bo_TE_64254	0.80952(34/42)	0.05882(1/17)	0.38095(8/21)	1.00000(19/19)
Bo_TE_45521	0.06522(3/46)	1.00000(18/18)	1.00000(22/22)	1.00000(20/20)
Bo_TE_89565	0.61364(27/44)	0.94118(16/17)	0.63636(7/11)	0.00000(0/17)
Bo_TE_112388	0.07143(3/42)	0.61111(11/18)	0.95455(21/22)	0.88889(16/18)
Bo_TE_130672	0.00000(0/44)	0.00000(0/18)	0.95455(21/22)	0.94444(17/18)
Bo_TE_150904	0.04651(2/43)	0.68750(11/16)	1.00000(22/22)	0.56250(9/16)
Bo_TE_149462	0.02326(1/43)	0.52941(9/17)	0.25000(5/20)	1.00000(20/20)
Bo_TE_192364	1.00000(46/46)	0.94444(17/18)	0.00000(0/22)	1.00000(20/20)
Bo_TE_88509	0.00000(0/46)	0.12500(2/16)	1.00000(23/23)	0.85000(17/20)
Bo_TE_107597	0.93023(40/43)	0.94444(17/18)	0.04545(1/22)	0.42105(8/19)
Bo_TE_149267	0.02273(1/44)	0.27778(5/18)	0.14286(3/21)	0.95000(19/20)
Bo_TE_149517	0.00000(0/45)	0.38889(7/18)	0.26316(5/19)	1.00000(18/18)
Bo_TE_142497	0.95000(38/40)	0.72222(13/18)	0.00000(0/21)	0.05000(1/20)
Bo_TE_30749	0.11111(5/45)	0.00000(0/16)	0.70000(14/20)	0.95000(19/20)
Bo_TE_19886	0.31707(13/41)	0.93750(15/16)	0.00000(0/19)	0.05556(1/18)
Bo_TE_48020	0.48649(18/37)	0.05263(1/19)	1.00000(22/22)	0.94737(18/19)
Bo_TE_32017	0.06667(3/45)	0.22222(4/18)	0.90476(19/21)	1.00000(20/20)
Bo_TE_107687	0.07692(3/39)	0.05556(1/18)	0.91304(21/23)	0.73684(14/19)
Bo_TE_181666	0.22222(10/45)	0.55556(10/18)	0.95455(21/22)	0.05000(1/20)
Bo_TE_8728	0.09756(4/41)	0.93750(15/16)	0.21739(5/23)	0.52941(9/17)
Bo_TE_114041	0.00000(0/45)	0.47059(8/17)	0.95652(22/23)	0.90000(18/20)
Bo_TE_100335	0.97500(39/40)	1.00000(19/19)	0.00000(0/22)	0.10526(2/19)
Bo_TE_237185	0.57143(4/7)	0.93750(15/16)	0.00000(0/18)	0.00000(0/17)
Bo_TE_151370	0.13953(6/43)	0.94118(16/17)	0.09091(2/22)	0.31579(6/19)
Bo_TE_29750	1.00000(43/43)	0.23529(4/17)	0.36842(7/19)	0.05000(1/20)
Bo_TE_114431	0.45000(18/40)	0.11765(2/17)	0.95238(20/21)	0.00000(0/20)
Bo_TE_172407	0.42857(18/42)	1.00000(17/17)	0.09524(2/21)	0.58333(7/12)
Bo_TE_196687	0.14286(6/42)	0.41176(7/17)	0.95238(20/21)	0.00000(0/20)
Bo_TE_53634	0.04545(2/44)	0.00000(0/18)	0.76190(16/21)	0.90000(18/20)

Bo_TE_172171	0.93023(40/43)	0.72222(13/18)	0.00000(0/22)	0.10000(2/20)
Bo_TE_205564	0.00000(0/43)	0.00000(0/18)	0.90909(20/22)	0.90000(18/20)
Bo_TE_8761	0.93023(40/43)	0.06250(1/16)	1.00000(21/21)	0.45000(9/20)
Bo_TE_4388	0.06818(3/44)	0.11111(2/18)	0.90909(20/22)	0.21053(4/19)
Bo_TE_173018	0.38636(17/44)	0.00000(0/18)	0.90000(18/20)	0.20000(4/20)
Bo_TE_238166	0.97727(43/44)	0.81250(13/16)	0.00000(0/23)	0.00000(0/19)
Bo_TE_206058	0.00000(0/43)	0.11111(2/18)	1.00000(21/21)	0.85000(17/20)
Bo_TE_63422	0.02174(1/46)	0.18750(3/16)	0.17391(4/23)	0.94444(17/18)
Bo_TE_101248	0.95455(42/44)	0.06250(1/16)	0.35000(7/20)	0.36842(7/19)
Bo_TE_203036	0.07143(3/42)	0.33333(6/18)	0.50000(11/22)	0.94444(17/18)
Bo_TE_191532	0.02222(1/45)	0.50000(8/16)	1.00000(20/20)	0.87500(14/16)
Bo_TE_104450	0.26190(11/42)	0.05556(1/18)	0.95455(21/22)	0.16667(3/18)
Bo_TE_174872	0.34146(14/41)	0.70588(12/17)	1.00000(21/21)	0.00000(0/19)
Bo_TE_228380	1.00000(43/43)	1.00000(18/18)	0.21053(4/19)	0.00000(0/19)
Bo_TE_180287	0.97674(42/43)	0.00000(0/16)	0.14286(3/21)	0.38889(7/18)
Bo_TE_11963	0.02273(1/44)	0.00000(0/18)	0.55000(11/20)	0.90000(18/20)
Bo_TE_205510	1.00000(46/46)	0.88889(16/18)	0.13636(3/22)	0.00000(0/19)
Bo_TE_110245	0.97778(44/45)	0.89474(17/19)	0.22222(4/18)	0.00000(0/20)
Bo_TE_9877	0.92857(39/42)	0.11765(2/17)	0.00000(0/23)	0.05263(1/19)
Bo_TE_226871	0.04762(2/42)	0.52941(9/17)	1.00000(22/22)	0.60000(12/20)
Bo_TE_98230	0.55814(24/43)	0.94118(16/17)	0.33333(7/21)	0.10000(2/20)
Bo_TE_100951	0.06818(3/44)	0.68750(11/16)	1.00000(22/22)	0.80000(16/20)
Bo_TE_86015	0.61364(27/44)	0.93750(15/16)	1.00000(10/10)	0.00000(0/18)
Bo_TE_190889	0.65000(26/40)	0.11765(2/17)	0.95000(19/20)	0.00000(0/20)
Bo_TE_235807	0.95000(38/40)	0.56250(9/16)	0.04348(1/23)	0.05263(1/19)
Bo_TE_183533	0.46154(18/39)	1.00000(18/18)	0.63636(14/22)	0.00000(0/20)
Bo_TE_164705	0.09756(4/41)	0.88889(16/18)	1.00000(23/23)	0.78947(15/19)
Bo_TE_22554	0.90909(40/44)	0.23529(4/17)	0.00000(0/23)	0.00000(0/19)
Bo_TE_205127	0.47500(19/40)	0.00000(0/16)	0.95455(21/22)	0.95000(19/20)
Bo_TE_145034	0.95556(43/45)	0.61111(11/18)	0.90476(19/21)	0.05000(1/20)
Bo_TE_163199	0.95238(40/42)	0.06250(1/16)	0.55556(5/9)	0.80000(4/5)
Bo_TE_77992	0.00000(0/42)	0.00000(0/17)	0.63636(14/22)	0.94737(18/19)
Bo_TE_101046	0.95556(43/45)	0.60000(9/15)	0.22222(4/18)	0.05882(1/17)
Bo_TE_77702	0.02222(1/45)	0.81250(13/16)	1.00000(22/22)	0.83333(15/18)
Bo_TE_141065	0.08889(4/45)	0.94118(16/17)	0.18182(4/22)	0.84211(16/19)
Bo_TE_15659	0.86364(38/44)	1.00000(16/16)	0.00000(0/22)	0.00000(0/20)
Bo_TE_76576	0.92500(37/40)	0.37500(3/8)	0.16667(2/12)	0.05556(1/18)
Bo_TE_37081	0.76190(32/42)	0.05556(1/18)	0.95455(21/22)	0.84211(16/19)
Bo_TE_226496	0.68293(28/41)	0.94444(17/18)	0.00000(0/23)	0.05000(1/20)
Bo_TE_144274	0.78571(33/42)	0.94444(17/18)	0.13636(3/22)	0.05000(1/20)
Bo_TE_979	0.90476(38/42)	0.06250(1/16)	0.45455(10/22)	0.75000(15/20)
Bo_TE_183106	0.00000(0/46)	0.00000(0/18)	0.60000(12/20)	0.94444(17/18)
Bo_TE_161743	0.90698(39/43)	0.35294(6/17)	0.09091(2/22)	0.25000(5/20)
Bo_TE_81477	0.20930(9/43)	0.00000(0/18)	0.90909(20/22)	0.05263(1/19)
Bo_TE_4166	0.86667(39/45)	0.94118(16/17)	0.71429(15/21)	0.05263(1/19)
Bo_TE_69956	0.26190(11/42)	0.05556(1/18)	1.00000(21/21)	0.40000(8/20)
Bo_TE_33372	0.91304(42/46)	0.07143(1/14)	0.80952(17/21)	0.05263(1/19)
Bo_TE_234268	1.00000(39/39)	0.55556(10/18)	0.00000(0/22)	0.42105(8/19)
Bo_TE_235841	0.04545(2/44)	0.50000(8/16)	0.86957(20/23)	0.94737(18/19)
Bo_TE_201159	0.24390(10/41)	0.00000(0/18)	1.00000(21/21)	1.00000(20/20)
Bo_TE_190875	0.40000(16/40)	0.78947(15/19)	0.09524(2/21)	1.00000(20/20)
Bo_TE_77701	0.02222(1/45)	0.82353(14/17)	1.00000(23/23)	0.88889(16/18)
Bo_TE_31239	0.44737(17/38)	0.05882(1/17)	0.95000(19/20)	0.73684(14/19)
Bo_TE_214867	1.00000(44/44)	0.93750(15/16)	0.10000(2/20)	0.10526(2/19)
Bo_TE_28035	0.02222(1/45)	0.76471(13/17)	0.52381(11/21)	0.90000(18/20)
Bo_TE_112954	0.00000(0/44)	0.00000(0/19)	0.00000(0/21)	0.94737(18/19)

Bo_TE_24367	0.95556(43/45)	0.38889(7/18)	0.04762(1/21)	0.21053(4/19)
Bo_TE_18703	0.50000(22/44)	0.06250(1/16)	0.95455(21/22)	0.77778(14/18)
Bo_TE_70969	0.02778(1/36)	0.05882(1/17)	1.00000(21/21)	0.88889(16/18)
Bo_TE_235805	0.04444(2/45)	0.62500(10/16)	0.91304(21/23)	0.61111(11/18)
Bo_TE_45573	0.93182(41/44)	0.00000(0/17)	0.19048(4/21)	0.00000(0/20)
Bo_TE_185521	0.02273(1/44)	0.81250(13/16)	0.57143(12/21)	0.90000(18/20)
Bo_TE_18463	0.00000(0/46)	0.18750(3/16)	0.63636(14/22)	0.90000(18/20)
Bo_TE_117957	0.00000(0/43)	0.00000(0/18)	1.00000(21/21)	0.88889(16/18)
Bo_TE_33192	0.91111(41/45)	0.50000(9/18)	0.60000(12/20)	0.00000(0/20)
Bo_TE_202077	1.00000(45/45)	0.05556(1/18)	0.81818(18/22)	0.95000(19/20)
Bo_TE_28786	0.04545(2/44)	0.81250(13/16)	1.00000(21/21)	0.94118(16/17)
Bo_TE_56077	0.79545(35/44)	0.00000(0/18)	1.00000(21/21)	0.55000(11/20)
Bo_TE_151410	0.11628(5/43)	0.94118(16/17)	0.09524(2/21)	0.31579(6/19)
Bo_TE_43047	0.13953(6/43)	0.44444(8/18)	0.09091(2/22)	0.94444(17/18)
Bo_TE_181348	0.97826(45/46)	0.05882(1/17)	0.00000(0/23)	0.36842(7/19)
Bo_TE_76577	0.04545(2/44)	0.27778(5/18)	0.40000(8/20)	0.94737(18/19)
Bo_TE_211504	0.90909(40/44)	0.47059(8/17)	0.80952(17/21)	0.10000(2/20)
Bo_TE_84840	0.00000(0/42)	0.35714(5/14)	0.90476(19/21)	0.55000(11/20)
Bo_TE_10324	0.02381(1/42)	0.18750(3/16)	0.95652(22/23)	0.22222(4/18)
Bo_TE_66974	0.06818(3/44)	0.00000(0/18)	1.00000(18/18)	1.00000(17/17)
Bo_TE_45780	0.13333(6/45)	0.00000(0/17)	0.90476(19/21)	0.38889(7/18)
Bo_TE_113002	0.15909(7/44)	0.83333(15/18)	0.94737(18/19)	0.05000(1/20)
Bo_TE_4372	0.00000(0/45)	0.55556(10/18)	0.95455(21/22)	0.61111(11/18)
Bo_TE_161036	0.90909(40/44)	0.64706(11/17)	0.05000(1/20)	0.38889(7/18)
Bo_TE_188834	0.02326(1/43)	0.05556(1/18)	0.30000(6/20)	1.00000(20/20)
Bo_TE_12760	0.06818(3/44)	0.93750(15/16)	0.19048(4/21)	0.94737(18/19)
Bo_TE_149302	0.95349(41/43)	0.70588(12/17)	0.00000(0/22)	0.00000(0/20)
Bo_TE_173011	0.42857(18/42)	0.00000(0/19)	0.90909(20/22)	0.22222(4/18)
Bo_TE_25072	0.95455(42/44)	0.35294(6/17)	0.00000(0/21)	0.00000(0/19)
Bo_TE_65934	0.92857(39/42)	0.73684(14/19)	0.85714(18/21)	0.00000(0/19)
Bo_TE_33382	0.07143(3/42)	0.88235(15/17)	0.19048(4/21)	1.00000(17/17)
Bo_TE_142471	0.93023(40/43)	0.94118(16/17)	0.09091(2/22)	0.35000(7/20)
Bo_TE_196453	0.92857(39/42)	0.88889(16/18)	0.26087(6/23)	0.05263(1/19)
Bo_TE_117960	1.00000(43/43)	1.00000(18/18)	0.00000(0/21)	0.10000(2/20)
Bo_TE_196470	0.97561(40/41)	0.55556(10/18)	0.04762(1/21)	0.80000(16/20)
Bo_TE_190645	0.93182(41/44)	0.05556(1/18)	0.68182(15/22)	0.10000(2/20)
Bo_TE_97612	0.09091(4/44)	0.31579(6/19)	1.00000(22/22)	0.21053(4/19)
Bo_TE_15040	1.00000(45/45)	0.25000(4/16)	0.33333(7/21)	0.05556(1/18)
Bo_TE_95066	0.09091(4/44)	0.52941(9/17)	0.91304(21/23)	0.00000(0/19)
Bo_TE_231062	0.02500(1/40)	0.94444(17/18)	0.47619(10/21)	0.55000(11/20)
Bo_TE_90011	0.06818(3/44)	0.93750(15/16)	0.76190(16/21)	0.90000(18/20)
Bo_TE_41005	1.00000(43/43)	1.00000(16/16)	0.20000(4/20)	0.00000(0/19)
Bo_TE_173481	1.00000(26/26)	0.93750(15/16)	0.00000(0/23)	0.00000(0/20)
Bo_TE_183685	0.95238(40/42)	0.77778(14/18)	0.00000(0/21)	0.21053(4/19)
Bo_TE_33247	0.93182(41/44)	0.61111(11/18)	0.63636(14/22)	0.00000(0/20)
Bo_TE_205582	1.00000(43/43)	0.94444(17/18)	0.11765(2/17)	0.00000(0/19)
Bo_TE_93395	0.00000(0/45)	0.00000(0/19)	0.95000(19/20)	0.00000(0/18)
Bo_TE_230814	0.02222(1/45)	0.93750(15/16)	0.47619(10/21)	0.83333(15/18)
Bo_TE_234094	0.93333(42/45)	1.00000(19/19)	0.00000(0/20)	1.00000(20/20)
Bo_TE_107956	0.93478(43/46)	0.76471(13/17)	0.00000(0/21)	0.15789(3/19)
Bo_TE_161032	0.09302(4/43)	0.55556(10/18)	1.00000(21/21)	0.70588(12/17)
Bo_TE_78620	0.06977(3/43)	0.52941(9/17)	0.90909(20/22)	0.90000(18/20)
Bo_TE_104711	0.00000(0/40)	0.11765(2/17)	0.90476(19/21)	0.15789(3/19)
Bo_TE_227159	0.58537(24/41)	0.80000(12/15)	0.95000(19/20)	0.10000(2/20)
Bo_TE_114048	0.00000(0/45)	0.41176(7/17)	1.00000(22/22)	0.85000(17/20)
Bo_TE_53838	0.26829(11/41)	0.00000(0/17)	1.00000(22/22)	0.80000(16/20)

Bo_TE_179202	0.25581(11/43)	0.16667(3/18)	0.04348(1/23)	0.90000(18/20)
Bo_TE_73641	0.00000(0/45)	0.64706(11/17)	0.65000(13/20)	0.94444(17/18)
Bo_TE_55929	0.09756(4/41)	0.52941(9/17)	0.15789(3/19)	0.95000(19/20)
Bo_TE_107534	0.55000(22/40)	0.50000(8/16)	0.95652(22/23)	0.05000(1/20)
Bo_TE_91810	0.00000(0/45)	0.05263(1/19)	0.94737(18/19)	0.95000(19/20)
Bo_TE_201054	1.00000(45/45)	1.00000(19/19)	0.00000(0/21)	0.00000(0/20)
Bo_TE_141735	0.92500(37/40)	0.75000(12/16)	0.00000(0/23)	0.30000(6/20)
Bo_TE_44830	0.34211(13/38)	0.94444(17/18)	0.04762(1/21)	0.76471(13/17)
Bo_TE_144566	0.85714(36/42)	0.94118(16/17)	0.00000(0/23)	0.88235(15/17)
Bo_TE_179747	0.35897(14/39)	0.62500(10/16)	0.10000(2/20)	1.00000(19/19)
Bo_TE_137099	0.02326(1/43)	0.88235(15/17)	0.33333(7/21)	0.94444(17/18)
Bo_TE_237828	0.60976(25/41)	0.05556(1/18)	0.95652(22/23)	0.78947(15/19)
Bo_TE_114117	0.09302(4/43)	0.47059(8/17)	0.17391(4/23)	0.95000(19/20)
Bo_TE_87102	0.93333(42/45)	0.89474(17/19)	0.05263(1/19)	0.11765(2/17)
Bo_TE_15584	0.88095(37/42)	1.00000(17/17)	0.61905(13/21)	0.05556(1/18)
Bo_TE_198663	0.06977(3/43)	0.16667(3/18)	0.35000(7/20)	0.94737(18/19)
Bo_TE_103747	0.91111(41/45)	0.52941(9/17)	0.00000(0/21)	0.10526(2/19)
Bo_TE_14154	0.79487(31/39)	0.93750(15/16)	0.77273(17/22)	0.00000(0/19)
Bo_TE_220355	1.00000(44/44)	0.50000(9/18)	0.33333(7/21)	0.10000(2/20)
Bo_TE_30547	0.25581(11/43)	0.05556(1/18)	0.95455(21/22)	0.31579(6/19)
Bo_TE_198581	0.09091(4/44)	0.16667(3/18)	0.40909(9/22)	0.94737(18/19)
Bo_TE_66210	0.04651(2/43)	1.00000(16/16)	0.47619(10/21)	0.15789(3/19)
Bo_TE_110254	0.90476(38/42)	0.88235(15/17)	0.20000(4/20)	0.00000(0/19)
Bo_TE_184068	0.04878(2/41)	0.00000(0/17)	0.75000(15/20)	1.00000(18/18)
Bo_TE_188826	0.02222(1/45)	0.00000(0/19)	0.33333(7/21)	1.00000(20/20)
Bo_TE_196482	0.97674(42/43)	0.58824(10/17)	0.00000(0/22)	0.00000(0/20)
Bo_TE_148038	0.02222(1/45)	0.00000(0/17)	0.95455(21/22)	0.78947(15/19)
Bo_TE_46120	0.00000(0/45)	0.50000(9/18)	1.00000(19/19)	0.75000(15/20)
Bo_TE_188798	0.00000(0/43)	0.00000(0/18)	0.52381(11/21)	0.94737(18/19)
Bo_TE_197736	0.45455(20/44)	0.94118(16/17)	0.09524(2/21)	0.33333(6/18)
Bo_TE_131705	0.32558(14/43)	0.05556(1/18)	0.95238(20/21)	0.57895(11/19)
Bo_TE_205571	1.00000(45/45)	0.88889(16/18)	0.10000(2/20)	0.05263(1/19)
Bo_TE_37112	0.30233(13/43)	0.00000(0/17)	0.95455(21/22)	0.57895(11/19)
Bo_TE_92835	0.70732(29/41)	0.94444(17/18)	0.85000(17/20)	0.10000(2/20)
Bo_TE_200984	0.00000(0/45)	0.16667(3/18)	1.00000(22/22)	1.00000(20/20)
Bo_TE_234058	0.95238(40/42)	0.00000(0/17)	0.00000(0/23)	0.05000(1/20)
Bo_TE_18258	1.00000(42/42)	0.05882(1/17)	0.54545(12/22)	0.26316(5/19)
Bo_TE_70949	0.02174(1/46)	0.00000(0/17)	0.95652(22/23)	0.61111(11/18)
Bo_TE_163704	0.42857(18/42)	0.05263(1/19)	1.00000(20/20)	0.84211(16/19)
Bo_TE_53755	0.08696(4/46)	0.11765(2/17)	0.81818(18/22)	0.94737(18/19)
Bo_TE_40931	1.00000(44/44)	1.00000(18/18)	0.21053(4/19)	0.00000(0/19)
Bo_TE_180257	1.00000(43/43)	0.05556(1/18)	1.00000(23/23)	1.00000(20/20)
Bo_TE_36737	1.00000(41/41)	0.70588(12/17)	0.00000(0/23)	0.13333(2/15)
Bo_TE_149223	1.00000(40/40)	0.90000(9/10)	0.00000(0/19)	0.00000(0/8)
Bo_TE_186013	0.09091(4/44)	0.11111(2/18)	0.30000(6/20)	0.95000(19/20)
Bo_TE_147175	0.00000(0/45)	0.00000(0/19)	0.91304(21/23)	0.26316(5/19)
Bo_TE_222848	0.00000(0/45)	0.00000(0/18)	0.90909(20/22)	0.42105(8/19)
Bo_TE_16901	0.76190(32/42)	0.94118(16/17)	0.00000(0/22)	0.00000(0/20)
Bo_TE_87573	0.02500(1/40)	0.22222(4/18)	0.68750(11/16)	0.94118(16/17)
Bo_TE_69091	0.40476(17/42)	0.70588(12/17)	0.08696(2/23)	0.94444(17/18)
Bo_TE_196399	0.11628(5/43)	0.17647(3/17)	0.95238(20/21)	0.05000(1/20)
Bo_TE_112950	0.76316(29/38)	0.11111(2/18)	1.00000(21/21)	0.00000(0/20)
Bo_TE_45876	0.06818(3/44)	0.64706(11/17)	1.00000(23/23)	0.90000(18/20)
Bo_TE_73616	0.02222(1/45)	0.75000(12/16)	0.68182(15/22)	0.94737(18/19)
Bo_TE_179819	0.02174(1/46)	0.22222(4/18)	0.42857(9/21)	0.95000(19/20)
Bo_TE_159424	0.40000(16/40)	0.94118(16/17)	0.60000(12/20)	0.00000(0/19)

Bo_TE_39068	0.93182(41/44)	0.43750(7/16)	0.00000(0/22)	0.00000(0/19)
Bo_TE_114047	0.00000(0/39)	0.47059(8/17)	1.00000(22/22)	0.89474(17/19)
Bo_TE_78686	0.07143(3/42)	0.27778(5/18)	0.50000(11/22)	0.94737(18/19)
Bo_TE_93166	0.00000(0/44)	0.00000(0/17)	0.90909(20/22)	0.47059(8/17)
Bo_TE_101761	0.00000(0/42)	0.58824(10/17)	0.04348(1/23)	1.00000(19/19)
Bo_TE_181339	0.00000(0/44)	0.00000(0/18)	0.91304(21/23)	0.35000(7/20)
Bo_TE_221692	0.20930(9/43)	0.00000(0/18)	0.47619(10/21)	0.90000(18/20)
Bo_TE_77878	0.82222(37/45)	1.00000(17/17)	0.35000(7/20)	0.00000(0/20)
Bo_TE_192357	0.88372(38/43)	0.76471(13/17)	0.00000(0/22)	0.95000(19/20)
Bo_TE_55993	0.14286(6/42)	0.00000(0/18)	0.90000(18/20)	0.78947(15/19)
Bo_TE_53671	0.97727(43/44)	0.52941(9/17)	0.09524(2/21)	0.10000(2/20)
Bo_TE_164726	0.90698(39/43)	0.11111(2/18)	0.00000(0/22)	0.20000(4/20)
Bo_TE_33373	0.92683(38/41)	0.12500(2/16)	0.86364(19/22)	0.05556(1/18)
Bo_TE_83828	0.75610(31/41)	0.94118(16/17)	0.04545(1/22)	0.57895(11/19)
Bo_TE_34713	0.54762(23/42)	0.94737(18/19)	0.09524(2/21)	0.50000(10/20)
Bo_TE_127350	0.04762(2/42)	0.62500(10/16)	1.00000(22/22)	1.00000(20/20)
Bo_TE_25130	0.02326(1/43)	0.33333(5/15)	0.90000(18/20)	0.20000(3/15)
Bo_TE_48013	0.51220(21/41)	0.05882(1/17)	1.00000(23/23)	1.00000(19/19)
Bo_TE_143124	0.02174(1/46)	0.11111(2/18)	0.90909(20/22)	0.21053(4/19)
Bo_TE_29756	1.00000(44/44)	0.26316(5/19)	0.33333(7/21)	0.05263(1/19)
Bo_TE_167071	0.97778(44/45)	0.86667(13/15)	0.20000(4/20)	0.00000(0/19)
Bo_TE_101694	0.00000(0/45)	0.25000(4/16)	0.09524(2/21)	1.00000(20/20)
Bo_TE_54521	0.95652(44/46)	0.37500(6/16)	0.00000(0/22)	0.05263(1/19)
Bo_TE_51429	0.00000(0/44)	0.00000(0/18)	1.00000(22/22)	1.00000(20/20)
Bo_TE_200993	0.00000(0/45)	0.00000(0/19)	1.00000(21/21)	1.00000(20/20)
Bo_TE_220959	0.02326(1/43)	0.88889(16/18)	0.90000(18/20)	0.26316(5/19)
Bo_TE_196406	0.17073(7/41)	0.18750(3/16)	0.95000(19/20)	0.05263(1/19)
Bo_TE_33060	0.90698(39/43)	0.05882(1/17)	0.39130(9/23)	0.81250(13/16)
Bo_TE_48004	0.46341(19/41)	0.94444(17/18)	0.00000(0/23)	0.05000(1/20)
Bo_TE_149332	0.00000(0/41)	0.22222(4/18)	0.19048(4/21)	0.95000(19/20)
Bo_TE_148577	0.09524(4/42)	0.16667(3/18)	0.95455(21/22)	0.31579(6/19)
Bo_TE_13477	0.92683(38/41)	0.86667(13/15)	0.00000(0/22)	0.20000(4/20)
Bo_TE_91405	0.59524(25/42)	0.00000(0/17)	0.30000(6/20)	0.90000(18/20)
Bo_TE_149327	0.02273(1/44)	0.22222(4/18)	0.19048(4/21)	0.94737(18/19)
Bo_TE_198628	0.04444(2/45)	0.16667(3/18)	0.35000(7/20)	0.95000(19/20)
Bo_TE_66245	0.95556(43/45)	0.00000(0/16)	0.55000(11/20)	0.75000(15/20)
Bo_TE_230427	0.00000(0/45)	0.21053(4/19)	1.00000(21/21)	0.05263(1/19)
Bo_TE_196463	0.95122(39/41)	0.55556(10/18)	0.00000(0/22)	0.00000(0/18)
Bo_TE_48099	0.46341(19/41)	0.00000(0/16)	1.00000(20/20)	0.95000(19/20)
Bo_TE_90091	0.95349(41/43)	0.05882(1/17)	0.20000(4/20)	0.05263(1/19)
Bo_TE_92950	0.06667(3/45)	0.00000(0/19)	0.80000(16/20)	0.90000(18/20)
Bo_TE_76623	0.95000(38/40)	0.72222(13/18)	0.00000(0/22)	0.00000(0/20)
Bo_TE_130667	1.00000(45/45)	1.00000(19/19)	0.05000(1/20)	0.05556(1/18)
Bo_TE_234295	0.02273(1/44)	0.38889(7/18)	1.00000(20/20)	0.66667(12/18)
Bo_TE_13644	0.91111(41/45)	0.00000(0/18)	0.00000(0/23)	0.00000(0/20)
Bo_TE_203047	0.07143(3/42)	0.35294(6/17)	0.47619(10/21)	0.95000(19/20)
Bo_TE_195796	1.00000(45/45)	0.76471(13/17)	0.84211(16/19)	0.10000(2/20)
Bo_TE_114133	1.00000(45/45)	0.57895(11/19)	0.00000(0/5)	0.05000(1/20)
Bo_TE_66211	0.93182(41/44)	0.00000(0/16)	0.52381(11/21)	0.84211(16/19)
Bo_TE_37104	0.04878(2/41)	0.05556(1/18)	0.95238(20/21)	0.83333(15/18)
Bo_TE_205443	0.00000(0/44)	0.05882(1/17)	0.89474(17/19)	0.94737(18/19)
Bo_TE_130453	1.00000(45/45)	0.94118(16/17)	0.00000(0/22)	0.50000(9/18)
Bo_TE_153695	0.91111(41/45)	0.23529(4/17)	0.04348(1/23)	0.31250(5/16)
Bo_TE_15633	0.86047(37/43)	1.00000(17/17)	0.00000(0/22)	0.00000(0/20)
Bo_TE_66090	0.83721(36/43)	0.93750(15/16)	0.04545(1/22)	0.41176(7/17)
Bo_TE_9855	0.95122(39/41)	0.17647(3/17)	0.00000(0/17)	0.05882(1/17)

Bo_TE_92973	0.90698(39/43)	0.94444(17/18)	0.09524(2/21)	0.10000(2/20)
Bo_TE_76640	0.95122(39/41)	0.84615(11/13)	0.00000(0/6)	0.00000(0/16)
Bo_TE_96145	0.09091(4/44)	0.16667(3/18)	0.35000(7/20)	1.00000(18/18)
Bo_TE_97872	0.18182(8/44)	0.05556(1/18)	0.95238(20/21)	0.42105(8/19)
Bo_TE_234208	0.00000(0/43)	0.06250(1/16)	1.00000(22/22)	0.94737(18/19)
Bo_TE_77958	1.00000(43/43)	1.00000(17/17)	0.31818(7/22)	0.05556(1/18)
Bo_TE_144700	0.13953(6/43)	0.83333(15/18)	0.90476(19/21)	0.10000(2/20)
Bo_TE_45584	0.93182(41/44)	0.00000(0/16)	0.18182(4/22)	0.00000(0/20)
Bo_TE_201036	0.00000(0/42)	0.00000(0/19)	1.00000(22/22)	1.00000(20/20)
Bo_TE_89339	0.02174(1/46)	0.16667(3/18)	1.00000(21/21)	0.88889(16/18)
Bo_TE_236045	0.93478(43/46)	0.05882(1/17)	0.00000(0/22)	0.00000(0/20)
Bo_TE_205627	0.73684(28/38)	0.00000(0/16)	0.90909(20/22)	0.95000(19/20)
Bo_TE_93837	0.95349(41/43)	1.00000(19/19)	0.09524(2/21)	0.10526(2/19)
Bo_TE_121466	0.17500(7/40)	0.94118(16/17)	0.25000(5/20)	0.10000(2/20)
Bo_TE_48059	0.48718(19/39)	0.00000(0/16)	1.00000(22/22)	0.95000(19/20)
Bo_TE_226365	0.68421(26/38)	0.00000(0/17)	0.95652(22/23)	0.00000(0/20)
Bo_TE_170738	0.08889(4/45)	0.41176(7/17)	1.00000(21/21)	0.47368(9/19)
Bo_TE_142499	0.95455(42/44)	0.76471(13/17)	0.00000(0/23)	0.00000(0/20)
Bo_TE_205448	1.00000(45/45)	0.82353(14/17)	0.08696(2/23)	0.00000(0/20)
Bo_TE_181347	0.02222(1/45)	0.93750(15/16)	1.00000(23/23)	0.61111(11/18)
Bo_TE_39041	0.05000(2/40)	0.53333(8/15)	0.95238(20/21)	0.66667(2/3)
Bo_TE_48003	0.51282(20/39)	0.94444(17/18)	0.00000(0/23)	0.05000(1/20)
Bo_TE_221621	0.76744(33/43)	0.93750(15/16)	0.28571(6/21)	0.05556(1/18)
Bo_TE_88407	0.20513(8/39)	0.94444(17/18)	0.00000(0/21)	0.11111(2/18)
Bo_TE_95824	0.72727(24/33)	1.00000(18/18)	0.00000(0/1)	0.00000(0/16)
Bo_TE_154423	0.02222(1/45)	0.93750(15/16)	0.00000(0/23)	0.00000(0/20)
Bo_TE_48243	0.02941(1/34)	0.00000(0/16)	0.95455(21/22)	0.40000(8/20)
Bo_TE_95778	0.09756(4/41)	0.00000(0/18)	0.68421(13/19)	1.00000(19/19)
Bo_TE_54891	0.95455(42/44)	0.16667(3/18)	0.08696(2/23)	0.50000(10/20)
Bo_TE_93726	0.05000(2/40)	1.00000(18/18)	0.05000(1/20)	0.00000(0/20)
Bo_TE_113854	0.73333(33/45)	0.61111(11/18)	0.08696(2/23)	0.95000(19/20)
Bo_TE_77861	0.20930(9/43)	0.11111(2/18)	0.08696(2/23)	0.94737(18/19)
Bo_TE_145048	0.00000(0/44)	0.22222(4/18)	0.00000(0/22)	0.95000(19/20)
Bo_TE_14149	0.76923(30/39)	1.00000(16/16)	0.73913(17/23)	0.00000(0/20)
Bo_TE_209290	0.56522(26/46)	0.06250(1/16)	0.95652(22/23)	0.65000(13/20)
Bo_TE_149407	0.10000(4/40)	0.88235(15/17)	1.00000(6/6)	1.00000(19/19)
Bo_TE_53753	0.92857(39/42)	0.94118(16/17)	0.19048(4/21)	0.00000(0/20)
Bo_TE_221573	0.74419(32/43)	0.94444(17/18)	0.52381(11/21)	0.00000(0/19)
Bo_TE_21731	0.97368(37/38)	0.92857(13/14)	0.62500(10/16)	0.00000(0/18)
Bo_TE_87214	0.09091(4/44)	0.11765(2/17)	0.57143(12/21)	0.90000(18/20)
Bo_TE_76832	0.15556(7/45)	0.17647(3/17)	1.00000(23/23)	0.10000(2/20)
Bo_TE_33173	0.97826(45/46)	0.66667(10/15)	0.61905(13/21)	0.05263(1/19)
Bo_TE_164641	0.09524(4/42)	0.88235(15/17)	1.00000(22/22)	0.78947(15/19)
Bo_TE_97869	0.16279(7/43)	0.05556(1/18)	1.00000(21/21)	0.72222(13/18)
Bo_TE_22650	0.00000(0/43)	0.75000(12/16)	1.00000(23/23)	1.00000(20/20)
Bo_TE_37405	0.04762(2/42)	0.81250(13/16)	0.86364(19/22)	0.94737(18/19)
Bo_TE_178276	0.90000(36/40)	0.06250(1/16)	0.00000(0/20)	0.05000(1/20)
Bo_TE_30533	0.75610(31/41)	0.94737(18/19)	0.04545(1/22)	0.65000(13/20)
Bo_TE_154013	0.51163(22/43)	1.00000(19/19)	0.04762(1/21)	0.50000(10/20)
Bo_TE_89977	0.09524(4/42)	0.44444(8/18)	0.76190(16/21)	0.90000(18/20)
Bo_TE_205411	0.00000(0/43)	0.00000(0/19)	0.90909(20/22)	0.88889(16/18)
Bo_TE_234250	0.00000(0/41)	0.38889(7/18)	0.95652(22/23)	0.65000(13/20)
Bo_TE_9592	0.09756(4/41)	0.68750(11/16)	1.00000(22/22)	0.11111(2/18)
Bo_TE_47868	0.93182(41/44)	0.88235(15/17)	0.00000(0/22)	0.21053(4/19)
Bo_TE_159794	0.90698(39/43)	0.17647(3/17)	0.09091(2/22)	0.78947(15/19)
Bo_TE_150815	1.00000(38/38)	0.73684(14/19)	0.05000(1/20)	0.47368(9/19)

Bo_TE_149434	1.00000(43/43)	0.47059(8/17)	0.76190(16/21)	0.00000(0/20)
Bo_TE_142466	0.90000(36/40)	0.18750(3/16)	0.10000(2/20)	0.31579(6/19)
Bo_TE_83796	0.83721(36/43)	0.94118(16/17)	0.04762(1/21)	0.73684(14/19)
Bo_TE_92588	0.04878(2/41)	0.26316(5/19)	1.00000(21/21)	0.80000(16/20)
Bo_TE_198645	0.93182(41/44)	0.83333(15/18)	0.65000(13/20)	0.05263(1/19)
Bo_TE_66216	0.93182(41/44)	0.00000(0/16)	0.52632(10/19)	0.84211(16/19)
Bo_TE_107852	0.00000(0/44)	0.62500(10/16)	0.90476(19/21)	0.80000(16/20)
Bo_TE_70966	0.93750(30/32)	0.94444(17/18)	0.00000(0/23)	0.11111(2/18)
Bo_TE_169502	0.18605(8/43)	0.00000(0/18)	1.00000(22/22)	0.94737(18/19)
Bo_TE_188696	0.39024(16/41)	0.00000(0/18)	0.21739(5/23)	1.00000(19/19)
Bo_TE_63063	0.93182(41/44)	0.37500(6/16)	0.00000(0/20)	0.00000(0/19)
Bo_TE_169760	0.91892(34/37)	0.20000(2/10)	0.00000(0/18)	0.06250(1/16)
Bo_TE_43323	0.00000(0/43)	0.23529(4/17)	0.89474(17/19)	0.90000(18/20)
Bo_TE_39791	0.95349(41/43)	0.93750(15/16)	0.00000(0/21)	0.00000(0/19)
Bo_TE_43919	0.93182(41/44)	0.23529(4/17)	0.00000(0/22)	0.00000(0/20)
Bo_TE_113896	0.00000(0/43)	0.12500(2/16)	0.95238(20/21)	0.90000(18/20)
Bo_TE_7427	0.39535(17/43)	0.06250(1/16)	0.90000(18/20)	0.57895(11/19)
Bo_TE_101721	0.89189(33/37)	0.94444(17/18)	0.95652(22/23)	0.00000(0/17)
Bo_TE_42980	0.84444(38/45)	0.56250(9/16)	0.90000(18/20)	0.00000(0/17)
Bo_TE_13628	0.90698(39/43)	0.00000(0/17)	0.00000(0/18)	0.27778(5/18)
Bo_TE_66086	0.84615(33/39)	1.00000(17/17)	0.00000(0/21)	0.45000(9/20)
Bo_TE_130650	0.00000(0/44)	0.52632(10/19)	0.90909(20/22)	0.94737(18/19)
Bo_TE_230429	0.00000(0/45)	0.21053(4/19)	1.00000(22/22)	0.05000(1/20)
Bo_TE_13649	0.91111(41/45)	0.00000(0/15)	0.00000(0/20)	0.31579(6/19)
Bo_TE_70094	0.06667(3/45)	0.94444(17/18)	0.20000(4/20)	0.63158(12/19)
Bo_TE_148040	0.95455(42/44)	0.87500(14/16)	0.04348(1/23)	0.00000(0/19)
Bo_TE_205409	0.97826(45/46)	0.82353(14/17)	0.09091(2/22)	0.00000(0/20)
Bo_TE_234255	0.90909(40/44)	0.11111(2/18)	0.00000(0/22)	0.36842(7/19)
Bo_TE_144276	0.11905(5/42)	0.05556(1/18)	0.61905(13/21)	0.95000(19/20)
Bo_TE_201026	1.00000(42/42)	1.00000(6/6)	0.00000(0/22)	0.00000(0/19)
Bo_TE_48234	0.00000(0/44)	0.00000(0/19)	0.95652(22/23)	0.40000(8/20)
Bo_TE_172008	0.00000(0/43)	0.10526(2/19)	0.90000(18/20)	0.05000(1/20)
Bo_TE_149464	0.95122(39/41)	0.47059(8/17)	0.70000(14/20)	0.00000(0/20)
Bo_TE_37083	0.73810(31/42)	0.05882(1/17)	0.95455(21/22)	0.85000(17/20)
Bo_TE_4412	0.02222(1/45)	1.00000(17/17)	0.13636(3/22)	0.10526(2/19)
Bo_TE_200359	0.97826(45/46)	0.88889(16/18)	0.10000(2/20)	0.63158(12/19)
Bo_TE_153409	0.00000(0/46)	0.00000(0/17)	0.94444(17/18)	0.94444(17/18)
Bo_TE_48048	0.50000(19/38)	0.05556(1/18)	1.00000(20/20)	0.95000(19/20)
Bo_TE_154143	0.04545(2/44)	0.75000(12/16)	1.00000(20/20)	1.00000(19/19)
Bo_TE_93960	0.97561(40/41)	0.44444(8/18)	0.04762(1/21)	0.10000(2/20)
Bo_TE_9842	0.05128(2/39)	0.83333(15/18)	1.00000(22/22)	0.95000(19/20)
Bo_TE_198682	0.92683(38/41)	0.77778(14/18)	0.66667(14/21)	0.00000(0/18)
Bo_TE_165674	0.63636(28/44)	0.05882(1/17)	0.40909(9/22)	0.90000(18/20)
Bo_TE_141225	0.93023(40/43)	0.05882(1/17)	0.09524(2/21)	0.16667(3/18)
Bo_TE_67929	0.90909(40/44)	0.05556(1/18)	0.13636(3/22)	0.00000(0/19)
Bo_TE_130601	0.55000(22/40)	0.94118(16/17)	0.05000(1/20)	0.00000(0/20)
Bo_TE_88192	0.04651(2/43)	0.22222(4/18)	1.00000(22/22)	0.93333(14/15)
Bo_TE_234966	0.00000(0/43)	0.50000(8/16)	1.00000(21/21)	0.40000(8/20)
Bo_TE_198664	0.07143(3/42)	0.22222(4/18)	0.35000(7/20)	0.94737(18/19)
Bo_TE_132934	0.09524(4/42)	0.29412(5/17)	1.00000(18/18)	0.94444(17/18)
Bo_TE_48046	0.46154(18/39)	0.94444(17/18)	0.00000(0/21)	0.05263(1/19)
Bo_TE_142437	0.92683(38/41)	0.82353(14/17)	0.00000(0/22)	0.31579(6/19)
Bo_TE_221972	0.46341(19/41)	0.06250(1/16)	0.58824(10/17)	0.95000(19/20)
Bo_TE_156227	0.97727(43/44)	1.00000(17/17)	0.00000(0/22)	0.42105(8/19)
Bo_TE_54898	0.97500(39/40)	0.11765(2/17)	0.09091(2/22)	0.52632(10/19)
Bo_TE_156957	0.95455(42/44)	0.33333(6/18)	0.04348(1/23)	0.05000(1/20)

Bo_TE_48086	0.50000(19/38)	0.94118(16/17)	0.00000(0/21)	0.05000(1/20)
Bo_TE_183032	0.59524(25/42)	0.88889(16/18)	0.95455(21/22)	0.10000(2/20)
Bo_TE_15580	0.84091(37/44)	1.00000(16/16)	0.61905(13/21)	0.05000(1/20)
Bo_TE_66461	0.48780(20/41)	0.00000(0/17)	0.14286(3/21)	1.00000(20/20)
Bo_TE_235575	0.00000(0/46)	0.00000(0/18)	0.91304(21/23)	0.20000(4/20)
Bo_TE_126001	0.00000(0/46)	0.00000(0/19)	1.00000(22/22)	0.80000(16/20)
Bo_TE_228573	0.00000(0/44)	0.11111(2/18)	0.80000(16/20)	1.00000(19/19)
Bo_TE_76601	0.04444(2/45)	0.31579(6/19)	0.38095(8/21)	0.95000(19/20)
Bo_TE_29779	1.00000(44/44)	0.27778(5/18)	0.31818(7/22)	0.05263(1/19)
Bo_TE_33493	0.91111(41/45)	0.13333(2/15)	0.09091(2/22)	0.15789(3/19)
Bo_TE_222176	0.09091(4/44)	0.78947(15/19)	1.00000(22/22)	0.85000(17/20)
Bo_TE_201061	1.00000(45/45)	1.00000(18/18)	0.00000(0/21)	0.00000(0/20)
Bo_TE_59366	0.25581(11/43)	0.05556(1/18)	0.63636(14/22)	0.95000(19/20)
Bo_TE_220933	0.00000(0/43)	0.94444(17/18)	0.90476(19/21)	0.47368(9/19)
Bo_TE_150674	0.95652(44/46)	0.27778(5/18)	0.04348(1/23)	0.76471(13/17)
Bo_TE_54890	0.04545(2/44)	0.83333(15/18)	0.91304(21/23)	0.50000(10/20)
Bo_TE_73314	1.00000(39/39)	0.23529(4/17)	0.35000(7/20)	0.05263(1/19)
Bo_TE_63499	0.07143(3/42)	0.11765(2/17)	0.40909(9/22)	0.94737(18/19)
Bo_TE_114279	0.02273(1/44)	0.17647(3/17)	0.90476(19/21)	0.00000(0/19)
Bo_TE_158316	0.90476(38/42)	0.87500(14/16)	0.09524(2/21)	0.21053(4/19)
Bo_TE_68366	0.08889(4/45)	0.05882(1/17)	0.95238(20/21)	0.42105(8/19)
Bo_TE_17779	0.00000(0/44)	0.05556(1/18)	0.40909(9/22)	0.95000(19/20)
Bo_TE_91852	0.97826(45/46)	0.94737(18/19)	0.00000(0/21)	0.05000(1/20)
Bo_TE_196720	0.00000(0/45)	0.33333(6/18)	0.90476(19/21)	0.38889(7/18)
Bo_TE_214936	1.00000(45/45)	1.00000(17/17)	0.16667(3/18)	0.05263(1/19)
Bo_TE_178993	0.00000(0/46)	0.00000(0/17)	0.95000(19/20)	0.05263(1/19)
Bo_TE_63438	0.08696(4/46)	0.23529(4/17)	0.40909(9/22)	0.94444(17/18)
Bo_TE_100313	0.95349(41/43)	1.00000(19/19)	0.00000(0/23)	0.11111(2/18)
Bo_TE_228390	0.00000(0/44)	0.00000(0/17)	0.84211(16/19)	1.00000(19/19)
Bo_TE_93857	0.97500(39/40)	0.89474(17/19)	0.10000(2/20)	0.10526(2/19)
Bo_TE_59540	0.18605(8/43)	0.05882(1/17)	0.66667(14/21)	0.95000(19/20)
Bo_TE_27814	0.97561(40/41)	0.20000(3/15)	0.55556(10/18)	0.05263(1/19)
Bo_TE_91976	0.00000(0/45)	0.05556(1/18)	0.90476(19/21)	0.42105(8/19)
Bo_TE_5342	0.02222(1/45)	0.76471(13/17)	0.52381(11/21)	0.90000(18/20)
Bo_TE_97140	0.50000(20/40)	1.00000(18/18)	0.00000(0/22)	0.47368(9/19)
Bo_TE_39030	0.07143(3/42)	0.56250(9/16)	0.65217(15/23)	1.00000(20/20)
Bo_TE_36494	0.97727(43/44)	0.40000(6/15)	0.47619(10/21)	0.10000(2/20)
Bo_TE_112333	0.97778(44/45)	0.64706(11/17)	0.20000(4/20)	0.05263(1/19)
Bo_TE_33533	0.09302(4/43)	0.58824(10/17)	0.91304(21/23)	0.85000(17/20)
Bo_TE_200357	0.97778(44/45)	0.88235(15/17)	0.09524(2/21)	0.57895(11/19)
Bo_TE_97269	0.00000(0/44)	0.05882(1/17)	0.90476(19/21)	0.75000(15/20)
Bo_TE_121996	0.46154(18/39)	1.00000(17/17)	0.00000(0/22)	0.70000(14/20)
Bo_TE_18936	0.35897(14/39)	0.17647(3/17)	0.95000(19/20)	0.00000(0/20)
Bo_TE_153759	0.00000(0/45)	0.93750(15/16)	0.68182(15/22)	0.15789(3/19)
Bo_TE_169506	0.13953(6/43)	0.00000(0/18)	1.00000(22/22)	1.00000(18/18)
Bo_TE_201065	1.00000(44/44)	1.00000(17/17)	0.00000(0/18)	0.00000(0/16)
Bo_TE_101727	0.00000(0/45)	0.00000(0/17)	0.04545(1/22)	1.00000(18/18)
Bo_TE_48096	0.48837(21/43)	0.00000(0/17)	1.00000(22/22)	0.95000(19/20)
Bo_TE_205569	0.00000(0/41)	0.11111(2/18)	0.90000(18/20)	0.95000(19/20)
Bo_TE_154469	0.97727(43/44)	0.06250(1/16)	0.81818(18/22)	1.00000(20/20)
Bo_TE_174831	0.06977(3/43)	0.43750(7/16)	0.95000(19/20)	0.15000(3/20)
Bo_TE_122281	0.15909(7/44)	0.05263(1/19)	0.90476(19/21)	0.38889(7/18)
Bo_TE_88614	0.90476(38/42)	0.87500(14/16)	0.00000(0/23)	0.38889(7/18)
Bo_TE_23127	1.00000(42/42)	0.11765(2/17)	0.33333(7/21)	0.10000(2/20)
Bo_TE_76222	0.04651(2/43)	0.27778(5/18)	1.00000(22/22)	0.89474(17/19)
Bo_TE_13609	0.09091(4/44)	1.00000(17/17)	1.00000(21/21)	0.70000(14/20)

Bo_TE_113932	1.00000(44/44)	0.94444(17/18)	0.05000(1/20)	0.16667(3/18)
Bo_TE_4557	0.00000(0/45)	0.70588(12/17)	0.95455(21/22)	0.90000(18/20)
Bo_TE_150539	0.04444(2/45)	0.50000(8/16)	0.90476(19/21)	0.45000(9/20)
Bo_TE_92594	0.04878(2/41)	0.29412(5/17)	1.00000(21/21)	0.80000(16/20)
Bo_TE_156909	0.62500(25/40)	1.00000(18/18)	0.45455(10/22)	0.05263(1/19)
Bo_TE_231030	0.02222(1/45)	0.53333(8/15)	0.95000(19/20)	0.11765(2/17)
Bo_TE_205580	0.00000(0/44)	0.10526(2/19)	0.90909(20/22)	0.95000(19/20)
Bo_TE_104778	0.04348(2/46)	0.00000(0/17)	0.85714(18/21)	0.90000(18/20)
Bo_TE_33486	0.90909(40/44)	0.11765(2/17)	0.08696(2/23)	0.15000(3/20)
Bo_TE_141202	0.95000(38/40)	0.05882(1/17)	0.10000(2/20)	0.16667(3/18)
Bo_TE_92978	0.04762(2/42)	0.05556(1/18)	0.90476(19/21)	0.90000(18/20)
Bo_TE_205524	1.00000(43/43)	0.94118(16/17)	0.09524(2/21)	0.00000(0/17)
Bo_TE_225000	0.97727(43/44)	0.93750(15/16)	0.80952(17/21)	0.00000(0/20)
Bo_TE_104244	0.20000(8/40)	0.00000(0/16)	0.90476(19/21)	1.00000(17/17)
Bo_TE_122861	0.97619(41/42)	0.29412(5/17)	0.00000(0/20)	0.26667(4/15)
Bo_TE_12762	0.09302(4/43)	0.93750(15/16)	0.20000(4/20)	0.95000(19/20)
Bo_TE_33409	0.02174(1/46)	0.93333(14/15)	0.77273(17/22)	1.00000(19/19)
Bo_TE_199058	0.00000(0/44)	0.26667(4/15)	0.28571(6/21)	0.90000(18/20)
Bo_TE_196589	0.15000(6/40)	0.29412(5/17)	0.04545(1/22)	0.95000(19/20)
Bo_TE_105040	0.30769(12/39)	0.00000(0/16)	1.00000(22/22)	0.94737(18/19)
Bo_TE_211772	0.06818(3/44)	0.44444(8/18)	0.95000(19/20)	0.36842(7/19)
Bo_TE_194446	0.75676(28/37)	0.94118(16/17)	0.90000(18/20)	0.00000(0/20)
Bo_TE_236923	0.02174(1/46)	0.52941(9/17)	1.00000(22/22)	0.80000(16/20)
Bo_TE_43918	0.93023(40/43)	0.25000(4/16)	0.00000(0/23)	0.00000(0/20)
Bo_TE_191344	0.00000(0/46)	0.52941(9/17)	0.10000(2/20)	0.90000(18/20)
Bo_TE_45574	0.02222(1/45)	1.00000(17/17)	0.78261(18/23)	1.00000(20/20)
Bo_TE_160850	0.04545(2/44)	0.25000(4/16)	0.95455(21/22)	0.94444(17/18)
Bo_TE_51324	0.00000(0/33)	0.00000(0/17)	0.95652(22/23)	1.00000(20/20)
Bo_TE_5640	0.97778(44/45)	0.50000(8/16)	0.04348(1/23)	0.22222(4/18)
Bo_TE_166824	0.97727(43/44)	0.88889(16/18)	0.19048(4/21)	0.00000(0/18)
Bo_TE_113904	1.00000(42/42)	0.72222(13/18)	0.00000(0/23)	0.05263(1/19)
Bo_TE_15565	0.86364(38/44)	1.00000(17/17)	0.36364(8/22)	0.00000(0/18)
Bo_TE_29906	0.00000(0/44)	0.76471(13/17)	0.66667(14/21)	0.95000(19/20)
Bo_TE_211493	0.08696(4/46)	0.70588(12/17)	0.18182(4/22)	0.90000(18/20)
Bo_TE_241979	0.97297(36/37)	0.13333(2/15)	0.45455(10/22)	0.05263(1/19)
Bo_TE_81472	0.22727(10/44)	0.05882(1/17)	0.90909(20/22)	0.05556(1/18)
Bo_TE_190890	0.62791(27/43)	0.16667(3/18)	0.95455(21/22)	0.05263(1/19)
Bo_TE_222962	0.47727(21/44)	0.20000(3/15)	0.95652(22/23)	0.00000(0/20)
Bo_TE_141016	0.09091(4/44)	0.94118(16/17)	0.90000(18/20)	0.84211(16/19)
Bo_TE_20864	0.93333(42/45)	1.00000(16/16)	0.09524(2/21)	0.05263(1/19)
Bo_TE_231673	1.00000(43/43)	0.16667(3/18)	0.50000(11/22)	0.00000(0/20)
Bo_TE_8221	0.97727(43/44)	0.56250(9/16)	0.76190(16/21)	0.00000(0/20)
Bo_TE_196474	0.97727(43/44)	0.55556(10/18)	0.00000(0/19)	0.80000(16/20)
Bo_TE_101705	0.79545(35/44)	0.88235(15/17)	0.95455(21/22)	0.05000(1/20)
Bo_TE_46396	0.07143(3/42)	0.16667(3/18)	0.15000(3/20)	0.95000(19/20)
Bo_TE_229669	0.07692(3/39)	0.58824(10/17)	0.52381(11/21)	0.90000(18/20)
Bo_TE_221694	0.93478(43/46)	1.00000(17/17)	0.47619(10/21)	0.10000(2/20)
Bo_TE_122288	0.97619(41/42)	0.92308(12/13)	0.04762(1/21)	0.72222(13/18)
Bo_TE_143127	0.04348(2/46)	0.33333(6/18)	0.95455(21/22)	0.30000(6/20)
Bo_TE_238334	0.69048(29/42)	0.05882(1/17)	0.90909(20/22)	0.95000(19/20)
Bo_TE_209650	0.97778(44/45)	0.55556(10/18)	0.04348(1/23)	1.00000(20/20)
Bo_TE_33788	0.09091(4/44)	0.11111(2/18)	0.09091(2/22)	0.94444(17/18)
Bo_TE_149542	0.00000(0/43)	0.41176(7/17)	0.20000(4/20)	1.00000(18/18)
Bo_TE_148102	0.56098(23/41)	0.94118(16/17)	0.63158(12/19)	0.00000(0/20)
Bo_TE_116308	0.02273(1/44)	0.61111(11/18)	1.00000(23/23)	0.60000(12/20)
Bo_TE_73455	1.00000(45/45)	0.23529(4/17)	0.35000(7/20)	0.05000(1/20)

Bo_TE_48040	0.55000(22/40)	0.05556(1/18)	1.00000(14/14)	0.93750(15/16)
Bo_TE_203080	0.95122(39/41)	0.66667(12/18)	0.52381(11/21)	0.05263(1/19)
Bo_TE_36992	0.09677(3/31)	0.00000(0/17)	0.90909(20/22)	0.52632(10/19)
Bo_TE_236040	0.00000(0/44)	0.58824(10/17)	0.50000(11/22)	1.00000(18/18)
Bo_TE_52657	0.60714(17/28)	0.20000(2/10)	0.00000(0/21)	0.94444(17/18)
Bo_TE_272	0.00000(0/45)	0.46667(7/15)	0.10000(2/20)	0.95000(19/20)
Bo_TE_224747	0.71111(32/45)	0.93750(15/16)	0.04348(1/23)	0.05263(1/19)
Bo_TE_22463	0.90698(39/43)	0.05263(1/19)	0.08696(2/23)	0.11111(2/18)
Bo_TE_32018	0.91111(41/45)	0.66667(12/18)	0.00000(0/23)	0.00000(0/20)
Bo_TE_113890	0.37209(16/43)	0.00000(0/19)	0.90476(19/21)	0.00000(0/20)
Bo_TE_92987	0.93182(41/44)	0.94118(16/17)	0.09524(2/21)	0.10526(2/19)
Bo_TE_112144	0.35556(16/45)	0.00000(0/18)	0.90476(19/21)	0.60000(12/20)
Bo_TE_93913	0.02273(1/44)	0.05263(1/19)	0.95238(20/21)	0.90000(18/20)
Bo_TE_39008	0.92683(38/41)	0.62500(10/16)	0.90000(18/20)	0.00000(0/20)
Bo_TE_236051	0.97727(43/44)	0.05882(1/17)	0.00000(0/22)	0.00000(0/19)
Bo_TE_157783	0.89130(41/46)	0.94444(17/18)	0.09524(2/21)	0.15789(3/19)
Bo_TE_4554	0.00000(0/45)	0.68750(11/16)	0.95652(22/23)	0.90000(18/20)
Bo_TE_23096	0.97436(38/39)	0.14286(2/14)	0.00000(0/21)	0.00000(0/18)
Bo_TE_148053	0.13636(6/44)	0.00000(0/18)	1.00000(19/19)	0.88889(16/18)
Bo_TE_154353	0.84091(37/44)	0.00000(0/17)	0.85000(17/20)	1.00000(20/20)
Bo_TE_91935	0.97826(45/46)	0.94444(17/18)	0.00000(0/21)	0.05000(1/20)
Bo_TE_123298	1.00000(45/45)	0.89474(17/19)	0.04762(1/21)	0.31579(6/19)
Bo_TE_15553	0.00000(0/24)	0.06250(1/16)	1.00000(20/20)	1.00000(18/18)
Bo_TE_127098	0.04444(2/45)	0.16667(3/18)	0.33333(7/21)	0.93750(15/16)
Bo_TE_129188	0.26829(11/41)	0.94118(16/17)	0.10000(2/20)	0.66667(12/18)
Bo_TE_92606	0.04545(2/44)	0.27778(5/18)	1.00000(21/21)	0.78947(15/19)
Bo_TE_36924	0.95349(41/43)	0.29412(5/17)	0.09091(2/22)	0.66667(2/3)
Bo_TE_139944	0.00000(0/45)	0.16667(3/18)	0.14286(3/21)	0.95000(19/20)
Bo_TE_1246	0.97436(38/39)	0.87500(14/16)	0.10000(2/20)	0.66667(10/15)
Bo_TE_148514	0.06818(3/44)	0.11111(2/18)	0.95652(22/23)	0.21053(4/19)
Bo_TE_73309	0.00000(0/44)	0.75000(12/16)	0.66667(14/21)	0.94444(17/18)
Bo_TE_13490	0.93182(41/44)	0.87500(14/16)	0.00000(0/21)	0.20000(4/20)
Bo_TE_67185	0.04545(2/44)	1.00000(18/18)	0.81818(18/22)	0.55000(11/20)
Bo_TE_15632	0.83721(36/43)	1.00000(16/16)	0.00000(0/22)	0.00000(0/20)
Bo_TE_13631	0.91304(42/46)	0.00000(0/15)	0.00000(0/22)	0.31579(6/19)
Bo_TE_173286	0.18919(7/37)	0.00000(0/13)	0.90476(19/21)	0.00000(0/19)
Bo_TE_70164	0.08889(4/45)	0.94444(17/18)	0.23810(5/21)	0.63158(12/19)
Bo_TE_22594	0.95349(41/43)	0.26667(4/15)	0.00000(0/21)	0.00000(0/18)
Bo_TE_87553	0.00000(0/43)	0.77778(14/18)	0.95652(22/23)	0.95000(19/20)
Bo_TE_84233	0.00000(0/46)	0.43750(7/16)	0.18182(4/22)	0.94737(18/19)
Bo_TE_177791	0.45238(19/42)	0.12500(2/16)	0.04545(1/22)	0.90000(18/20)
Bo_TE_53705	0.02222(1/45)	0.50000(9/18)	0.90909(20/22)	0.78947(15/19)
Bo_TE_73480	0.00000(0/43)	0.77778(14/18)	0.68421(13/19)	0.94444(17/18)
Bo_TE_205616	0.26829(11/41)	0.94118(16/17)	0.09091(2/22)	0.00000(0/20)
Bo_TE_59421	0.25581(11/43)	0.05882(1/17)	0.68182(15/22)	0.94737(18/19)
Bo_TE_222846	0.06667(3/45)	0.00000(0/17)	0.95455(21/22)	0.90000(18/20)
Bo_TE_188781	0.00000(0/42)	0.00000(0/17)	0.47826(11/23)	1.00000(19/19)
Bo_TE_145405	0.30769(12/39)	0.94118(16/17)	0.00000(0/21)	0.00000(0/17)
Bo_TE_80205	0.13333(6/45)	0.05263(1/19)	0.95238(20/21)	0.22222(4/18)
Bo_TE_215385	1.00000(43/43)	0.62500(10/16)	0.05263(1/19)	0.11111(2/18)
Bo_TE_205483	0.00000(0/46)	0.16667(3/18)	0.90909(20/22)	0.95000(19/20)
Bo_TE_235635	0.75610(31/41)	0.06250(1/16)	1.00000(23/23)	0.70000(14/20)
Bo_TE_167209	0.02174(1/46)	0.11111(2/18)	0.80952(17/21)	1.00000(19/19)
Bo_TE_201031	0.00000(0/45)	0.00000(0/19)	1.00000(21/21)	1.00000(20/20)
Bo_TE_92975	0.04545(2/44)	0.05556(1/18)	0.90476(19/21)	0.90000(18/20)
Bo_TE_24380	0.97778(44/45)	0.22222(4/18)	0.04762(1/21)	0.05556(1/18)

Bo_TE_66975	0.91111(41/45)	1.00000(19/19)	0.00000(0/21)	0.00000(0/20)
Bo_TE_205334	0.00000(0/46)	0.23529(4/17)	0.90909(20/22)	0.90000(18/20)
Bo_TE_227657	0.09091(4/44)	0.11765(2/17)	0.82609(19/23)	0.95000(19/20)
Bo_TE_216102	0.84783(39/46)	0.93750(15/16)	0.09524(2/21)	0.73684(14/19)
Bo_TE_161037	0.05000(2/40)	0.38889(7/18)	0.90476(19/21)	0.58824(10/17)
Bo_TE_205213	0.95652(44/46)	0.22222(4/18)	0.08696(2/23)	0.05263(1/19)
Bo_TE_113899	1.00000(45/45)	0.72222(13/18)	0.00000(0/21)	0.05556(1/18)
Bo_TE_148049	0.00000(0/46)	0.00000(0/18)	0.95652(22/23)	0.78947(15/19)
Bo_TE_235849	0.93023(40/43)	0.11111(1/9)	0.50000(9/18)	0.00000(0/16)
Bo_TE_167566	0.97778(44/45)	0.89474(17/19)	0.25000(5/20)	0.00000(0/18)
Bo_TE_43002	0.85366(35/41)	0.62500(10/16)	0.90909(20/22)	0.05556(1/18)
Bo_TE_87557	0.97674(42/43)	0.22222(4/18)	0.04348(1/23)	0.05263(1/19)
Bo_TE_237574	0.30233(13/43)	1.00000(17/17)	0.04348(1/23)	0.40000(8/20)
Bo_TE_67027	0.00000(0/45)	0.00000(0/19)	1.00000(23/23)	0.89474(17/19)
Bo_TE_70081	0.06667(3/45)	0.94118(16/17)	0.28571(6/21)	0.65000(13/20)
Bo_TE_40976	1.00000(45/45)	1.00000(18/18)	0.20000(4/20)	0.00000(0/19)
Bo_TE_90087	0.91304(42/46)	0.05882(1/17)	0.19048(4/21)	0.00000(0/20)
Bo_TE_62712	0.53488(23/43)	0.00000(0/16)	0.52381(11/21)	0.95000(19/20)
Bo_TE_142439	1.00000(45/45)	0.89474(17/19)	0.09524(2/21)	0.63158(12/19)
Bo_TE_196694	0.95122(39/41)	0.47059(8/17)	0.04762(1/21)	0.50000(10/20)
Bo_TE_236589	0.04762(2/42)	0.26316(5/19)	0.90909(20/22)	0.36842(7/19)
Bo_TE_95822	0.24390(10/41)	0.05556(1/18)	1.00000(20/20)	0.20000(4/20)
Bo_TE_22595	0.93478(43/46)	0.31250(5/16)	0.00000(0/22)	0.00000(0/19)
Bo_TE_89625	0.26190(11/42)	1.00000(17/17)	0.31818(7/22)	0.00000(0/19)
Bo_TE_130258	0.00000(0/45)	0.00000(0/19)	0.91304(21/23)	0.70000(14/20)
Bo_TE_228532	1.00000(46/46)	0.88235(15/17)	0.25000(4/16)	0.00000(0/19)
Bo_TE_108165	0.00000(0/44)	0.94737(18/19)	0.71429(15/21)	0.22222(4/18)
Bo_TE_66222	0.92857(39/42)	0.00000(0/16)	0.54545(12/22)	0.84211(16/19)
Bo_TE_67226	0.04348(2/46)	1.00000(18/18)	0.82609(19/23)	0.55000(11/20)
Bo_TE_100312	0.95455(42/44)	1.00000(18/18)	0.00000(0/22)	0.11765(2/17)
Bo_TE_191627	0.02381(1/42)	0.58333(7/12)	0.94737(18/19)	1.00000(14/14)
Bo_TE_230583	0.22727(10/44)	0.82353(14/17)	0.90909(20/22)	0.00000(0/20)
Bo_TE_226343	0.67442(29/43)	0.20000(3/15)	0.95455(21/22)	0.00000(0/20)
Bo_TE_198685	0.93478(43/46)	0.77778(14/18)	0.61905(13/21)	0.05263(1/19)
Bo_TE_208374	0.70000(28/40)	0.25000(4/16)	0.05000(1/20)	0.94737(18/19)
Bo_TE_163720	0.54762(23/42)	0.94737(18/19)	0.00000(0/20)	0.00000(0/20)
Bo_TE_93949	0.02222(1/45)	0.00000(0/19)	0.95000(19/20)	0.90000(18/20)
Bo_TE_92099	0.04444(2/45)	0.26316(5/19)	1.00000(22/22)	0.31579(6/19)
Bo_TE_221751	0.00000(0/45)	0.08333(1/12)	0.04762(1/21)	1.00000(16/16)
Bo_TE_191804	0.00000(0/45)	0.05882(1/17)	1.00000(22/22)	0.22222(4/18)
Bo_TE_45576	0.97561(40/41)	0.00000(0/17)	0.20000(4/20)	0.00000(0/19)
Bo_TE_143772	0.33333(14/42)	0.00000(0/18)	1.00000(23/23)	0.21053(4/19)
Bo_TE_230838	0.06818(3/44)	0.11111(2/18)	0.90476(19/21)	0.94737(18/19)
Bo_TE_169519	0.15909(7/44)	0.05556(1/18)	1.00000(21/21)	0.95000(19/20)
Bo_TE_89674	0.97826(45/46)	0.61111(11/18)	0.40000(8/20)	0.05556(1/18)
Bo_TE_22464	0.90909(40/44)	0.05263(1/19)	0.08696(2/23)	0.10526(2/19)
Bo_TE_220348	0.00000(0/45)	0.50000(8/16)	0.61905(13/21)	0.90000(18/20)
Bo_TE_238170	0.97778(44/45)	0.82353(14/17)	0.00000(0/16)	0.05882(1/17)
Bo_TE_39014	0.04545(2/44)	0.00000(0/18)	0.90476(19/21)	0.00000(0/20)
Bo_TE_144903	0.00000(0/46)	0.80000(12/15)	1.00000(23/23)	0.44444(8/18)
Bo_TE_149264	0.92857(39/42)	0.70588(12/17)	0.81818(18/22)	0.05000(1/20)
Bo_TE_29934	0.00000(0/44)	0.70588(12/17)	0.66667(14/21)	0.94737(18/19)
Bo_TE_129145	0.22222(10/45)	0.00000(0/19)	0.95000(19/20)	0.00000(0/19)
Bo_TE_18886	0.39024(16/41)	0.18750(3/16)	0.95455(21/22)	0.00000(0/19)
Bo_TE_142505	0.97368(37/38)	0.76471(13/17)	0.00000(0/21)	0.00000(0/19)
Bo_TE_140143	0.90909(40/44)	0.70588(12/17)	0.39130(9/23)	0.05263(1/19)

Bo_TE_59517	0.73810(31/42)	1.00000(16/16)	0.28571(6/21)	0.05000(1/20)
Bo_TE_76835	0.15556(7/45)	0.22222(4/18)	1.00000(22/22)	0.05556(1/18)
Bo_TE_117814	0.95455(42/44)	0.44444(8/18)	0.33333(7/21)	0.00000(0/19)
Bo_TE_113914	0.00000(0/43)	0.00000(0/18)	0.95455(21/22)	0.83333(15/18)
Bo_TE_410	0.97778(44/45)	0.88889(16/18)	0.20000(4/20)	0.00000(0/19)
Bo_TE_154106	1.00000(45/45)	1.00000(17/17)	0.09091(2/22)	0.15789(3/19)
Bo_TE_205303	0.00000(0/43)	0.06250(1/16)	0.83333(10/12)	0.94737(18/19)
Bo_TE_11277	0.02564(1/39)	0.58824(10/17)	0.94737(18/19)	0.68421(13/19)
Bo_TE_228684	1.00000(45/45)	0.89474(17/19)	0.20000(4/20)	0.00000(0/19)
Bo_TE_25068	0.97436(38/39)	0.44444(8/18)	0.00000(0/21)	0.00000(0/19)
Bo_TE_161732	0.08696(4/46)	0.55556(10/18)	0.95238(20/21)	0.66667(12/18)
Bo_TE_53718	0.97674(42/43)	0.52632(10/19)	0.10000(2/20)	0.15789(3/19)
Bo_TE_136006	0.00000(0/37)	0.05263(1/19)	0.06667(1/15)	0.90000(18/20)
Bo_TE_113843	0.83721(36/43)	0.82353(14/17)	0.09091(2/22)	0.94737(18/19)
Bo_TE_101706	0.82500(33/40)	0.88889(16/18)	0.95455(21/22)	0.00000(0/17)
Bo_TE_136742	0.00000(0/43)	0.12500(2/16)	1.00000(21/21)	0.78947(15/19)
Bo_TE_191363	0.00000(0/44)	0.63158(12/19)	1.00000(23/23)	1.00000(19/19)
Bo_TE_205575	1.00000(44/44)	0.89474(17/19)	0.09091(2/22)	0.05000(1/20)
Bo_TE_169500	0.84444(38/45)	1.00000(18/18)	0.00000(0/22)	0.05000(1/20)
Bo_TE_43016	0.82500(33/40)	0.58824(10/17)	0.90909(20/22)	0.05882(1/17)
Bo_TE_214641	1.00000(43/43)	0.70588(12/17)	0.00000(0/22)	0.23529(4/17)
Bo_TE_165089	0.93023(40/43)	0.06250(1/16)	0.00000(0/22)	0.20000(4/20)
Bo_TE_6037	0.71111(32/45)	0.06250(1/16)	0.30000(6/20)	1.00000(20/20)
Bo_TE_177413	0.30435(14/46)	0.11111(2/18)	0.04348(1/23)	0.94444(17/18)
Bo_TE_148139	0.83333(35/42)	0.00000(0/19)	0.40000(8/20)	0.94737(18/19)
Bo_TE_51252	0.00000(0/45)	0.00000(0/19)	1.00000(21/21)	1.00000(18/18)
Bo_TE_143949	0.00000(0/43)	0.00000(0/12)	0.85000(17/20)	0.94444(17/18)
Bo_TE_42997	0.12500(5/40)	0.41176(7/17)	0.09091(2/22)	0.94118(16/17)
Bo_TE_29768	0.00000(0/45)	0.77778(14/18)	0.70000(14/20)	0.95000(19/20)
Bo_TE_87105	0.91892(34/37)	0.75000(6/8)	0.05263(1/19)	0.10526(2/19)
Bo_TE_222845	0.06522(3/46)	0.00000(0/19)	0.95455(21/22)	0.94737(18/19)
Bo_TE_134919	0.00000(0/44)	0.05556(1/18)	0.90000(18/20)	0.72222(13/18)
Bo_TE_15571	0.86047(37/43)	1.00000(17/17)	0.30000(6/20)	0.00000(0/20)
Bo_TE_125910	0.06667(3/45)	0.11765(2/17)	0.90476(19/21)	0.94737(18/19)
Bo_TE_141722	0.04444(2/45)	0.23529(4/17)	1.00000(21/21)	0.73684(14/19)
Bo_TE_33211	0.93478(43/46)	0.62500(10/16)	0.68182(15/22)	0.00000(0/19)
Bo_TE_53704	0.97500(39/40)	0.52632(10/19)	0.10000(2/20)	0.15000(3/20)
Bo_TE_137916	0.09302(4/43)	0.56250(9/16)	0.18182(4/22)	0.95000(19/20)
Bo_TE_45707	0.28571(12/42)	1.00000(17/17)	0.04762(1/21)	0.22222(4/18)
Bo_TE_160858	1.00000(44/44)	0.47059(8/17)	0.10526(2/19)	0.10000(2/20)
Bo_TE_167177	0.02222(1/45)	0.11111(2/18)	0.78947(15/19)	1.00000(18/18)
Bo_TE_43753	0.20930(9/43)	0.93750(15/16)	0.55000(11/20)	0.10000(2/20)
Bo_TE_238311	0.65854(27/41)	0.05882(1/17)	0.90909(20/22)	0.95000(19/20)
Bo_TE_60923	0.07143(3/42)	0.11765(2/17)	0.94737(18/19)	0.73684(14/19)
Bo_TE_140159	0.91111(41/45)	0.61111(11/18)	0.38095(8/21)	0.00000(0/19)
Bo_TE_84253	0.90244(37/41)	0.58824(10/17)	0.82609(19/23)	0.05000(1/20)
Bo_TE_205197	0.04348(2/46)	0.76471(13/17)	0.90909(20/22)	0.94444(17/18)
Bo_TE_15044	1.00000(46/46)	0.25000(4/16)	0.30000(6/20)	0.00000(0/20)
Bo_TE_24399	0.97674(42/43)	0.64706(11/17)	0.09091(2/22)	0.40000(4/10)
Bo_TE_93843	0.02326(1/43)	0.00000(0/17)	0.90476(19/21)	0.90000(18/20)
Bo_TE_221574	0.23256(10/43)	0.05556(1/18)	0.47368(9/19)	1.00000(19/19)
Bo_TE_214823	0.97674(42/43)	0.87500(14/16)	0.11111(2/18)	0.10000(2/20)
Bo_TE_234182	0.06667(3/45)	0.16667(3/18)	1.00000(20/20)	0.00000(0/19)
Bo_TE_205456	0.97826(45/46)	0.83333(15/18)	0.09091(2/22)	0.00000(0/20)
Bo_TE_235847	0.06977(3/43)	0.41176(7/17)	0.50000(11/22)	0.95000(19/20)
Bo_TE_6109	0.04545(2/44)	0.00000(0/18)	1.00000(21/21)	0.77778(14/18)

	Bo_TE_45518	0.97727(43/44)	0.00000(0/16)	0.00000(0/21)	0.00000(0/18)
	Bo_TE_228574	0.00000(0/42)	0.10526(2/19)	0.80000(16/20)	1.00000(19/19)
	Bo_TE_167360	0.02381(1/42)	0.05882(1/17)	0.80000(16/20)	1.00000(19/19)
	Bo_TE_30718	0.11364(5/44)	0.05556(1/18)	0.70000(14/20)	0.95000(19/20)
	Bo_TE_29940	0.00000(0/41)	0.77778(14/18)	0.70000(14/20)	0.94737(18/19)
	Bo_TE_73340	1.00000(44/44)	0.22222(4/18)	0.33333(7/21)	0.05000(1/20)
	Bo_TE_180294	0.02439(1/41)	0.94118(16/17)	0.90000(18/20)	0.61111(11/18)
	Bo_TE_76834	0.11628(5/43)	0.12500(2/16)	1.00000(22/22)	0.10000(2/20)
	Bo_TE_214835	1.00000(45/45)	1.00000(17/17)	0.14286(3/21)	0.10000(2/20)
	Bo_TE_201674	0.63415(26/41)	0.94118(16/17)	0.10000(2/20)	0.05263(1/19)
	Bo_TE_39114	0.04545(2/44)	0.06250(1/16)	0.60000(12/20)	0.95000(19/20)
	Bo_TE_230422	0.00000(0/41)	0.23529(4/17)	1.00000(22/22)	0.00000(0/19)
	Bo_TE_33403	0.06977(3/43)	0.93750(15/16)	0.15789(3/19)	0.95000(19/20)
	Bo_TE_154184	0.00000(0/45)	0.12500(2/16)	0.95652(22/23)	0.94444(17/18)
	Bo_TE_73665	0.00000(0/44)	0.11111(2/18)	0.80000(16/20)	1.00000(19/19)
	Bo_TE_43008	0.86667(39/45)	0.58824(10/17)	0.90909(20/22)	0.05263(1/19)
	Bo_TE_7964	0.06667(3/45)	0.00000(0/18)	0.90000(18/20)	0.29412(5/17)
	Bo_TE_13675	0.90909(40/44)	0.00000(0/16)	0.00000(0/20)	0.30000(6/20)
	Bo_TE_93831	0.04651(2/43)	0.00000(0/18)	0.94737(18/19)	0.90000(18/20)
	Bo_TE_191549	0.00000(0/46)	0.62500(10/16)	0.85714(18/21)	0.90000(18/20)
	Bo_TE_183542	0.48718(19/39)	0.00000(0/18)	0.00000(0/22)	1.00000(20/20)
	Bo_TE_130065	0.04762(2/42)	0.00000(0/19)	0.90476(19/21)	0.50000(9/18)
	Bo_TE_56099	0.04444(2/45)	0.05556(1/18)	1.00000(23/23)	0.68750(11/16)
	Bo_TE_182015	0.13636(6/44)	0.00000(0/18)	1.00000(21/21)	0.21053(4/19)
	Bo_TE_11887	0.04545(2/44)	0.35294(6/17)	0.57143(12/21)	0.90000(18/20)
	Bo_TE_147255	0.00000(0/42)	0.42105(8/19)	0.95455(21/22)	0.29412(5/17)
	Bo_TE_93178	0.19048(8/42)	0.05556(1/18)	0.95238(20/21)	0.21053(4/19)
	Bo_TE_102716	0.46341(19/41)	1.00000(16/16)	0.38095(8/21)	0.05000(1/20)
	Bo_TE_190635	0.90909(40/44)	0.00000(0/16)	0.63636(14/22)	0.10526(2/19)
	Bo_TE_239644	0.00000(0/44)	0.52941(9/17)	1.00000(23/23)	0.88235(15/17)
	Bo_TE_154464	0.97826(45/46)	0.06250(1/16)	0.80952(17/21)	1.00000(20/20)
	Bo_TE_33236	0.06667(3/45)	0.40000(6/15)	0.33333(7/21)	1.00000(20/20)
	Bo_TE_97675	0.08889(4/45)	0.87500(14/16)	0.04762(1/21)	0.90000(18/20)
	Bo_TE_54511	0.95652(44/46)	0.38462(5/13)	0.00000(0/21)	0.00000(0/19)
	Bo_TE_195794	1.00000(45/45)	0.81250(13/16)	0.85714(18/21)	0.10000(2/20)
	Bo_TE_169507	0.82222(37/45)	1.00000(18/18)	0.00000(0/22)	0.05000(1/20)
	Bo_TE_211131	0.02222(1/45)	0.05882(1/17)	1.00000(21/21)	0.72222(13/18)
	Bo_TE_183731	0.09302(4/43)	0.22222(4/18)	0.95652(22/23)	0.47059(8/17)
	Bo_TE_38999	0.95349(41/43)	0.62500(10/16)	0.90909(20/22)	0.00000(0/20)
	Bo_TE_121694	0.50000(19/38)	0.06250(1/16)	0.95455(21/22)	0.00000(0/19)
	Bo_TE_221628	0.74419(32/43)	0.94444(17/18)	0.27273(6/22)	0.05000(1/20)
	Bo_TE_77597	0.00000(0/45)	0.94118(16/17)	0.40909(9/22)	0.16667(3/18)
	Bo_TE_154762	0.02222(1/45)	0.62500(10/16)	0.90000(18/20)	0.80000(16/20)
	Bo_TE_76590	0.04878(2/41)	0.31579(6/19)	0.38095(8/21)	0.95000(19/20)
	Bo_TE_83798	0.21951(9/41)	0.05882(1/17)	0.90909(20/22)	0.22222(4/18)
	Bo_TE_228392	0.00000(0/46)	0.00000(0/19)	0.75000(15/20)	1.00000(19/19)
	Bo_TE_170980	0.62791(27/43)	0.94118(16/17)	0.70000(14/20)	0.10000(2/20)
	Bo_TE_226444	0.35897(14/39)	0.00000(0/16)	0.73913(17/23)	0.94737(18/19)
	Bo_TE_90700	0.00000(0/44)	0.93750(15/16)	0.66667(14/21)	0.15000(3/20)
y heterogeneity	Bo_TE_215605	0.71429(30/42)	0.75000(12/16)	0.25000(5/20)	0.21053(4/19)
	Bo_TE_141538	0.83333(35/42)	0.05556(1/18)	0.04762(1/21)	0.22222(4/18)
	Bo_TE_182692	0.50000(20/40)	0.94444(17/18)	1.00000(22/22)	1.00000(18/18)
	Bo_TE_58079	0.39024(16/41)	0.06250(1/16)	0.70000(14/20)	0.22222(4/18)
	Bo_TE_23343	0.00000(0/45)	0.00000(0/19)	0.26316(5/19)	0.70000(14/20)
	Bo_TE_181063	0.32558(14/43)	0.17647(3/17)	0.61905(13/21)	0.00000(0/20)

Frequenc

Bo_TE_65904	0.06977(3/43)	0.12500(2/16)	0.57143(12/21)	0.44444(8/18)
Bo_TE_138412	0.80952(34/42)	1.00000(18/18)	0.85000(17/20)	0.26316(5/19)
Bo_TE_67812	0.88372(38/43)	0.60000(9/15)	0.65217(15/23)	0.22222(4/18)
Bo_TE_213833	0.86047(37/43)	0.94444(17/18)	0.36364(8/22)	0.44444(8/18)
Bo_TE_85472	0.68889(31/45)	0.29412(5/17)	0.47619(10/21)	0.95000(19/20)
Bo_TE_134671	0.84444(38/45)	0.35294(6/17)	0.91304(21/23)	0.94737(18/19)
Bo_TE_230549	0.18605(8/43)	0.00000(0/15)	0.70000(14/20)	0.94444(17/18)
Bo_TE_99683	0.69231(27/39)	0.06250(1/16)	0.00000(0/17)	0.00000(0/14)
Bo_TE_58336	0.22500(9/40)	0.00000(0/19)	0.60000(12/20)	0.15789(3/19)
Bo_TE_232736	0.97826(45/46)	1.00000(19/19)	0.63636(14/22)	0.27778(5/18)
Bo_TE_17966	0.80000(32/40)	0.37500(6/16)	0.28571(6/21)	0.88889(16/18)
Bo_TE_20486	0.58537(24/41)	0.00000(0/17)	0.04545(1/22)	0.05000(1/20)
Bo_TE_144324	0.26829(11/41)	0.47059(8/17)	0.72727(16/22)	0.90000(18/20)
Bo_TE_62780	0.97727(43/44)	0.33333(6/18)	0.54545(12/22)	1.00000(20/20)
Bo_TE_153171	0.02222(1/45)	0.56250(9/16)	0.30435(7/23)	0.72222(13/18)
Bo_TE_102017	0.14634(6/41)	0.58824(10/17)	0.95652(22/23)	1.00000(19/19)
Bo_TE_92450	0.93182(41/44)	0.94444(17/18)	0.21053(4/19)	0.75000(15/20)
Bo_TE_191422	0.00000(0/43)	0.00000(0/19)	0.85714(18/21)	0.73684(14/19)
Bo_TE_142823	0.50000(22/44)	0.05882(1/17)	0.31818(7/22)	0.57895(11/19)
Bo_TE_215623	0.35556(16/45)	0.11765(2/17)	0.77273(17/22)	0.85000(17/20)
Bo_TE_53603	0.02381(1/42)	0.00000(0/19)	0.75000(15/20)	0.89474(17/19)
Bo_TE_41957	0.09091(4/44)	0.11111(2/18)	0.09091(2/22)	0.63158(12/19)
Bo_TE_177486	0.33333(14/42)	0.53333(8/15)	0.81818(18/22)	0.25000(5/20)
Bo_TE_143194	0.00000(0/45)	0.00000(0/19)	0.04545(1/22)	0.88889(16/18)
Bo_TE_25674	0.47619(20/42)	0.68750(11/16)	0.00000(0/19)	0.10526(2/19)
Bo_TE_111853	0.27273(12/44)	0.06250(1/16)	0.76190(16/21)	0.00000(0/18)
Bo_TE_220500	0.54286(19/35)	0.87500(14/16)	0.57143(12/21)	0.20000(4/20)
Bo_TE_97587	0.85714(36/42)	0.82353(14/17)	1.00000(21/21)	0.36842(7/19)
Bo_TE_53129	0.50000(20/40)	0.15789(3/19)	0.00000(0/23)	0.00000(0/20)
Bo_TE_11740	0.58974(23/39)	0.38889(7/18)	0.00000(0/22)	0.00000(0/20)
Bo_TE_96577	0.63415(26/41)	0.05263(1/19)	0.54545(12/22)	0.33333(6/18)
Bo_TE_173247	0.38462(15/39)	0.70588(12/17)	0.72727(16/22)	1.00000(19/19)
Bo_TE_84419	0.95652(44/46)	0.17647(3/17)	0.83333(10/12)	0.43750(7/16)
Bo_TE_68353	0.16279(7/43)	0.44444(8/18)	0.77273(17/22)	0.45000(9/20)
Bo_TE_128391	0.02174(1/46)	0.15789(3/19)	0.55000(11/20)	0.00000(0/20)
Bo_TE_186256	0.21739(10/46)	0.05556(1/18)	0.00000(0/23)	0.88889(16/18)
Bo_TE_93002	0.61364(27/44)	0.23529(4/17)	0.73913(17/23)	1.00000(20/20)
Bo_TE_200450	0.44186(19/43)	0.50000(9/18)	1.00000(22/22)	0.95000(19/20)
Bo_TE_168864	0.02564(1/39)	0.64706(11/17)	0.20000(4/20)	0.55000(11/20)
Bo_TE_80443	0.04545(2/44)	0.00000(0/18)	0.43478(10/23)	0.78947(15/19)
Bo_TE_153880	0.88095(37/42)	0.42105(8/19)	1.00000(23/23)	0.94737(18/19)
Bo_TE_138028	0.82222(37/45)	0.88889(16/18)	0.09524(2/21)	0.00000(0/19)
Bo_TE_70962	0.53659(22/41)	0.55556(10/18)	0.00000(0/23)	0.05000(1/20)
Bo_TE_27411	0.77273(34/44)	0.82353(14/17)	0.30000(6/20)	0.00000(0/20)
Bo_TE_58865	0.00000(0/46)	0.15789(3/19)	0.63158(12/19)	0.00000(0/20)
Bo_TE_189032	0.68293(28/41)	0.18750(3/16)	0.00000(0/23)	0.00000(0/20)
Bo_TE_95302	0.00000(0/46)	0.61111(11/18)	0.04348(1/23)	0.00000(0/20)
Bo_TE_167723	0.09677(3/31)	0.11111(2/18)	0.60000(12/20)	0.06250(1/16)
Bo_TE_113984	0.59459(22/37)	0.66667(10/15)	0.00000(0/19)	0.00000(0/17)
Bo_TE_9580	0.78049(32/41)	0.12500(2/16)	0.40909(9/22)	0.75000(15/20)
Bo_TE_130799	0.00000(0/44)	0.00000(0/18)	0.00000(0/22)	0.52941(9/17)
Bo_TE_136093	0.59091(26/44)	0.17647(3/17)	0.00000(0/22)	0.00000(0/20)
Bo_TE_152270	0.13333(6/45)	0.75000(12/16)	0.50000(11/22)	0.85000(17/20)
Bo_TE_121415	0.00000(0/45)	0.00000(0/18)	0.04545(1/22)	0.73684(14/19)
Bo_TE_211004	0.11111(5/45)	0.43750(7/16)	0.61905(13/21)	0.52632(10/19)
Bo_TE_178535	1.00000(45/45)	1.00000(17/17)	0.52381(11/21)	0.38889(7/18)

Bo_TE_159001	0.17778(8/45)	0.23529(4/17)	0.90000(18/20)	0.73684(14/19)
Bo_TE_148130	0.84091(37/44)	0.11765(2/17)	0.40000(8/20)	0.94737(18/19)
Bo_TE_70166	0.75000(33/44)	0.05882(1/17)	0.76190(16/21)	0.35000(7/20)
Bo_TE_137363	0.44186(19/43)	1.00000(18/18)	0.90476(19/21)	0.57895(11/19)
Bo_TE_180946	0.40909(18/44)	0.82353(14/17)	0.43478(10/23)	0.95000(19/20)
Bo_TE_137509	0.25641(10/39)	0.40000(6/15)	0.10526(2/19)	0.85000(17/20)
Bo_TE_63690	0.00000(0/40)	0.00000(0/14)	0.29412(5/17)	0.55000(11/20)
Bo_TE_131827	0.06818(3/44)	0.22222(4/18)	0.60870(14/23)	0.64706(11/17)
Bo_TE_63574	0.04545(2/44)	0.64706(11/17)	0.23810(5/21)	0.00000(0/20)
Bo_TE_101801	0.04545(2/44)	0.00000(0/16)	0.75000(15/20)	0.83333(15/18)
Bo_TE_92028	0.63415(26/41)	0.05556(1/18)	0.00000(0/23)	0.55556(10/18)
Bo_TE_169192	0.95556(43/45)	1.00000(18/18)	0.42857(9/21)	0.65000(13/20)
Bo_TE_102596	0.34146(14/41)	0.82353(14/17)	0.28571(6/21)	0.47368(9/19)
Bo_TE_66271	1.00000(44/44)	0.12500(2/16)	0.38095(8/21)	0.16667(3/18)
Bo_TE_8588	0.16279(7/43)	0.88889(16/18)	0.85714(18/21)	0.94444(17/18)
Bo_TE_37621	0.37500(15/40)	0.82353(14/17)	0.95238(20/21)	0.75000(15/20)
Bo_TE_156235	0.72500(29/40)	0.05882(1/17)	0.00000(0/23)	0.00000(0/17)
Bo_TE_58323	0.00000(0/35)	0.10526(2/19)	0.93333(14/15)	0.72222(13/18)
Bo_TE_235199	0.30952(13/42)	0.26667(4/15)	0.70000(14/20)	0.83333(15/18)
Bo_TE_177624	0.00000(0/45)	0.00000(0/19)	0.45000(9/20)	0.50000(10/20)
Bo_TE_45638	0.62791(27/43)	0.06250(1/16)	0.22727(5/22)	0.00000(0/20)
Bo_TE_406	0.36842(14/38)	0.76471(13/17)	0.04762(1/21)	0.22222(4/18)
Bo_TE_206517	0.04651(2/43)	0.00000(0/19)	0.04545(1/22)	0.61111(11/18)
Bo_TE_223026	0.72727(32/44)	0.11765(2/17)	0.00000(0/23)	0.00000(0/14)
Bo_TE_28340	0.50000(21/42)	0.76471(13/17)	1.00000(22/22)	0.77778(14/18)
Bo_TE_113498	0.20455(9/44)	0.52632(10/19)	1.00000(19/19)	0.93750(15/16)
Bo_TE_105665	0.04545(2/44)	0.52941(9/17)	0.04545(1/22)	0.00000(0/19)
Bo_TE_17783	0.11364(5/44)	0.17647(3/17)	0.37500(6/16)	0.94737(18/19)
Bo_TE_208531	0.06977(3/43)	0.38462(5/13)	0.59091(13/22)	0.10526(2/19)
Bo_TE_48653	0.48780(20/41)	0.17647(3/17)	0.68182(15/22)	0.94737(18/19)
Bo_TE_232763	0.97778(44/45)	1.00000(19/19)	0.76190(16/21)	0.35000(7/20)
Bo_TE_68485	0.00000(0/45)	0.00000(0/18)	0.65000(13/20)	0.52632(10/19)
Bo_TE_134038	0.35897(14/39)	0.06667(1/15)	0.80000(16/20)	0.00000(0/20)
Bo_TE_111587	0.81395(35/43)	1.00000(19/19)	0.23810(5/21)	1.00000(19/19)
Bo_TE_5669	0.77273(34/44)	0.35294(6/17)	0.04545(1/22)	0.21053(4/19)
Bo_TE_155074	0.12195(5/41)	0.11111(2/18)	0.23810(5/21)	0.70000(14/20)
Bo_TE_79606	0.77273(34/44)	0.42857(6/14)	0.66667(14/21)	0.10000(2/20)
Bo_TE_163621	0.81395(35/43)	0.87500(14/16)	0.81818(18/22)	0.27778(5/18)
Bo_TE_99265	0.15556(7/45)	0.76471(13/17)	0.80952(17/21)	0.60000(12/20)
Bo_TE_221196	0.21429(9/42)	0.87500(14/16)	0.04348(1/23)	0.05000(1/20)
Bo_TE_12224	0.05263(2/38)	0.63636(7/11)	1.00000(13/13)	0.68750(11/16)
Bo_TE_57593	0.54545(24/44)	0.52941(9/17)	0.42857(9/21)	0.00000(0/20)
Bo_TE_216427	0.53659(22/41)	0.05882(1/17)	0.00000(0/23)	0.00000(0/20)
Bo_TE_156159	0.67500(27/40)	0.18750(3/16)	0.00000(0/19)	0.15000(3/20)
Bo_TE_58548	0.67568(25/37)	0.88235(15/17)	0.73684(14/19)	0.00000(0/19)
Bo_TE_235560	0.65909(29/44)	0.00000(0/17)	0.00000(0/22)	0.10000(2/20)
Bo_TE_237292	0.05128(2/39)	0.05882(1/17)	0.57143(12/21)	0.45000(9/20)
Bo_TE_237187	0.83333(35/42)	0.12500(2/16)	0.00000(0/20)	0.00000(0/19)
Bo_TE_216003	1.00000(46/46)	0.72222(13/18)	0.31818(7/22)	0.15789(3/19)
Bo_TE_172879	0.46512(20/43)	0.00000(0/17)	0.45000(9/20)	0.61111(11/18)
Bo_TE_27466	0.72093(31/43)	0.00000(0/18)	0.00000(0/23)	0.00000(0/20)
Bo_TE_109389	0.76923(30/39)	0.31250(5/16)	0.95455(21/22)	1.00000(11/11)
Bo_TE_1036	0.75610(31/41)	0.68750(11/16)	0.66667(14/21)	0.10526(2/19)
Bo_TE_94333	0.02174(1/46)	0.16667(3/18)	0.42857(9/21)	0.72222(13/18)
Bo_TE_81438	0.20513(8/39)	0.00000(0/19)	0.09091(2/22)	0.65000(13/20)
Bo_TE_65608	0.23913(11/46)	0.61111(11/18)	0.95238(20/21)	0.82353(14/17)

Bo_TE_179212	0.35897(14/39)	0.56250(9/16)	0.31818(7/22)	0.95000(19/20)
Bo_TE_79319	0.15000(6/40)	0.00000(0/19)	0.35000(7/20)	0.50000(10/20)
Bo_TE_170628	0.55814(24/43)	0.43750(7/16)	0.00000(0/19)	0.68421(13/19)
Bo_TE_101286	0.04348(2/46)	0.14286(2/14)	0.45000(9/20)	0.70000(14/20)
Bo_TE_215508	0.02174(1/46)	0.47059(8/17)	0.33333(7/21)	0.65000(13/20)
Bo_TE_230128	0.00000(0/45)	0.55556(10/18)	0.09524(2/21)	0.00000(0/20)
Bo_TE_33761	0.54762(23/42)	0.50000(9/18)	0.00000(0/23)	0.05263(1/19)
Bo_TE_88375	0.09756(4/41)	0.56250(9/16)	0.00000(0/23)	0.00000(0/20)
Bo_TE_224571	0.73810(31/42)	0.16667(3/18)	0.72727(16/22)	0.60000(12/20)
Bo_TE_114329	0.30952(13/42)	0.50000(8/16)	0.95455(21/22)	0.63158(12/19)
Bo_TE_221943	0.65909(29/44)	0.22222(4/18)	0.82609(19/23)	1.00000(20/20)
Bo_TE_190944	0.69767(30/43)	0.00000(0/17)	0.00000(0/21)	0.55000(11/20)
Bo_TE_43124	0.55814(24/43)	0.00000(0/15)	0.00000(0/20)	0.30000(6/20)
Bo_TE_230892	0.86667(39/45)	0.88889(16/18)	0.00000(0/21)	0.00000(0/19)
Bo_TE_95635	0.09091(4/44)	0.33333(6/18)	0.19048(4/21)	0.68421(13/19)
Bo_TE_198476	0.11111(5/45)	0.27778(5/18)	0.38095(8/21)	0.94444(17/18)
Bo_TE_41914	0.80000(36/45)	0.35294(6/17)	0.10000(2/20)	0.16667(3/18)
Bo_TE_83751	0.17500(7/40)	0.00000(0/18)	0.73684(14/19)	0.30000(6/20)
Bo_TE_143215	0.52273(23/44)	0.05882(1/17)	0.00000(0/21)	0.00000(0/20)
Bo_TE_141572	0.82609(38/46)	0.05882(1/17)	0.04545(1/22)	0.21053(4/19)
Bo_TE_236121	0.93182(41/44)	0.94118(16/17)	0.25000(5/20)	0.90000(18/20)
Bo_TE_56211	0.41026(16/39)	0.55556(10/18)	0.47619(10/21)	0.00000(0/20)
Bo_TE_144863	0.82609(38/46)	0.05882(1/17)	0.00000(0/23)	0.57895(11/19)
Bo_TE_230320	0.95556(43/45)	0.37500(6/16)	0.86364(19/22)	0.89474(17/19)
Bo_TE_89510	0.38636(17/44)	0.25000(4/16)	0.66667(14/21)	0.94737(18/19)
Bo_TE_108820	0.06667(3/45)	0.50000(8/16)	0.17391(4/23)	0.00000(0/19)
Bo_TE_20003	0.80952(34/42)	0.11111(2/18)	0.47619(10/21)	0.10526(2/19)
Bo_TE_27049	0.97561(40/41)	1.00000(18/18)	0.73684(14/19)	0.50000(9/18)
Bo_TE_220443	0.00000(0/44)	0.10526(2/19)	0.40909(9/22)	0.80000(16/20)
Bo_TE_231035	0.31818(14/44)	0.64286(9/14)	1.00000(23/23)	0.95000(19/20)
Bo_TE_196335	0.11628(5/43)	0.55556(10/18)	0.04762(1/21)	0.25000(5/20)
Bo_TE_2862	0.23810(10/42)	0.05882(1/17)	0.57143(12/21)	0.57895(11/19)
Bo_TE_42290	0.04348(2/46)	0.38889(7/18)	0.78261(18/23)	0.84211(16/19)
Bo_TE_230591	0.17857(5/28)	0.11765(2/17)	0.04348(1/23)	0.58824(10/17)
Bo_TE_23376	0.59091(13/22)	0.83333(15/18)	1.00000(20/20)	0.50000(9/18)
Bo_TE_105321	0.13636(6/44)	0.37500(6/16)	0.00000(0/22)	0.50000(10/20)
Bo_TE_103969	0.00000(0/46)	0.05263(1/19)	0.50000(10/20)	0.52632(10/19)
Bo_TE_120984	0.00000(0/46)	0.00000(0/19)	0.19048(4/21)	0.75000(15/20)
Bo_TE_90115	0.13514(5/37)	0.43750(7/16)	0.50000(7/14)	0.80000(16/20)
Bo_TE_17788	0.11364(5/44)	0.18750(3/16)	0.42857(9/21)	0.95000(19/20)
Bo_TE_120661	0.55814(24/43)	0.87500(14/16)	0.09091(2/22)	0.31579(6/19)
Bo_TE_183897	0.30769(12/39)	0.70588(12/17)	1.00000(23/23)	1.00000(20/20)
Bo_TE_196460	0.02326(1/43)	0.00000(0/19)	0.00000(0/20)	0.77778(14/18)
Bo_TE_235228	0.95455(42/44)	0.17647(3/17)	1.00000(21/21)	0.57895(11/19)
Bo_TE_139078	0.07143(3/42)	0.88889(16/18)	0.68182(15/22)	0.80000(16/20)
Bo_TE_32760	0.92683(38/41)	0.58824(10/17)	0.39130(9/23)	0.61111(11/18)
Bo_TE_158960	0.15909(7/44)	0.52941(9/17)	0.00000(0/22)	0.45000(9/20)
Bo_TE_221998	0.29268(12/41)	0.93750(15/16)	0.65000(13/20)	0.44444(8/18)
Bo_TE_148765	0.33333(12/36)	0.12500(2/16)	1.00000(23/23)	1.00000(20/20)
Bo_TE_154017	0.39535(17/43)	0.68750(11/16)	0.00000(0/22)	0.05263(1/19)
Bo_TE_117910	0.00000(0/46)	0.16667(3/18)	0.57895(11/19)	0.05263(1/19)
Bo_TE_75727	0.00000(0/45)	0.57895(11/19)	0.00000(0/21)	0.00000(0/18)
Bo_TE_156074	0.46667(21/45)	0.83333(15/18)	0.00000(0/21)	0.15000(3/20)
Bo_TE_95693	0.02326(1/43)	0.00000(0/18)	0.00000(0/22)	0.78947(15/19)
Bo_TE_95724	0.53488(23/43)	0.36842(7/19)	0.00000(0/22)	0.05000(1/20)
Bo_TE_160627	0.27907(12/43)	0.42105(8/19)	1.00000(23/23)	0.55000(11/20)

Bo_TE_46268	0.00000(0/42)	0.06250(1/16)	0.71429(15/21)	0.05882(1/17)
Bo_TE_71278	0.11111(5/45)	0.88235(15/17)	0.75000(15/20)	0.52941(9/17)
Bo_TE_101273	0.04444(2/45)	0.18750(3/16)	0.47826(11/23)	0.72222(13/18)
Bo_TE_117376	0.92683(38/41)	0.17647(3/17)	0.30000(6/20)	0.50000(9/18)
Bo_TE_90639	0.08889(4/45)	0.63158(12/19)	0.59091(13/22)	0.15000(3/20)
Bo_TE_227944	0.66667(28/42)	0.07143(1/14)	0.08696(2/23)	0.00000(0/19)
Bo_TE_184077	0.20000(9/45)	0.10526(2/19)	0.57143(12/21)	0.94737(18/19)
Bo_TE_174554	0.38095(16/42)	0.37500(6/16)	0.90909(20/22)	0.26316(5/19)
Bo_TE_53685	0.02326(1/43)	0.42105(8/19)	0.71429(15/21)	0.68421(13/19)
Bo_TE_173284	0.46512(20/43)	0.25000(4/16)	0.85714(18/21)	0.21053(4/19)
Bo_TE_54572	0.00000(0/46)	0.38889(7/18)	0.00000(0/21)	0.60000(12/20)
Bo_TE_183957	0.81395(35/43)	0.72222(13/18)	0.04762(1/21)	0.05556(1/18)
Bo_TE_81656	0.00000(0/45)	0.52632(10/19)	0.00000(0/23)	0.00000(0/20)
Bo_TE_234899	0.97826(45/46)	0.89474(17/19)	0.95652(22/23)	0.47368(9/19)
Bo_TE_53743	0.04651(2/43)	0.47059(8/17)	0.17391(4/23)	0.63158(12/19)
Bo_TE_55460	0.02273(1/44)	0.35294(6/17)	0.71429(15/21)	0.05000(1/20)
Bo_TE_124980	0.60526(23/38)	0.88235(15/17)	0.20000(3/15)	0.15000(3/20)
Bo_TE_76400	0.65116(28/43)	0.43750(7/16)	0.08696(2/23)	0.21053(4/19)
Bo_TE_121175	0.00000(0/35)	0.00000(0/17)	0.80000(8/10)	0.72222(13/18)
Bo_TE_236186	0.61905(26/42)	0.05556(1/18)	0.00000(0/22)	0.00000(0/17)
Bo_TE_122635	0.19512(8/41)	0.16667(3/18)	0.95652(22/23)	0.75000(15/20)
Bo_TE_4335	0.11364(5/44)	0.18750(3/16)	0.09091(2/22)	0.83333(15/18)
Bo_TE_95193	0.30000(12/40)	0.62500(10/16)	0.80000(16/20)	0.07143(1/14)
Bo_TE_102401	0.79545(35/44)	0.73333(11/15)	0.00000(0/22)	0.10000(2/20)
Bo_TE_89964	0.90244(37/41)	0.55556(10/18)	0.25000(5/20)	0.21053(4/19)
Bo_TE_7359	0.85714(36/42)	0.40000(6/15)	0.04762(1/21)	0.42105(8/19)
Bo_TE_90676	0.09091(4/44)	0.64706(11/17)	0.66667(14/21)	0.15000(3/20)
Bo_TE_95011	0.00000(0/45)	0.17647(3/17)	0.54545(12/22)	0.11765(2/17)
Bo_TE_195514	0.35135(13/37)	0.68750(11/16)	0.15000(3/20)	0.33333(6/18)
Bo_TE_196725	0.00000(0/44)	0.00000(0/17)	0.52174(12/23)	0.00000(0/20)
Bo_TE_238517	0.17500(7/40)	0.00000(0/17)	0.71429(15/21)	0.88235(15/17)
Bo_TE_17764	0.58974(23/39)	0.43750(7/16)	1.00000(21/21)	1.00000(20/20)
Bo_TE_108014	1.00000(44/44)	0.35294(6/17)	0.76190(16/21)	0.76471(13/17)
Bo_TE_43042	0.16667(7/42)	0.37500(6/16)	0.09091(2/22)	0.89474(17/19)
Bo_TE_216150	0.02222(1/45)	0.05882(1/17)	0.08696(2/23)	0.57895(11/19)
Bo_TE_58871	0.58140(25/43)	0.00000(0/17)	0.00000(0/23)	0.30000(6/20)
Bo_TE_95612	0.97561(40/41)	0.44444(8/18)	0.85714(18/21)	0.89474(17/19)
Bo_TE_58517	0.00000(0/45)	0.35294(6/17)	0.80000(16/20)	0.00000(0/19)
Bo_TE_171125	0.54545(24/44)	0.29412(5/17)	0.40000(8/20)	0.85000(17/20)
Bo_TE_133287	0.22500(9/40)	0.11765(2/17)	0.90909(20/22)	0.15789(3/19)
Bo_TE_229196	0.00000(0/41)	0.28571(4/14)	0.50000(10/20)	0.50000(10/20)
Bo_TE_126849	0.72500(29/40)	0.47059(8/17)	1.00000(21/21)	0.85000(17/20)
Bo_TE_123404	0.97778(44/45)	0.44444(8/18)	0.19048(4/21)	0.26316(5/19)
Bo_TE_107831	0.00000(0/39)	0.00000(0/15)	0.81250(13/16)	0.81250(13/16)
Bo_TE_215125	1.00000(45/45)	0.47059(8/17)	1.00000(23/23)	1.00000(20/20)
Bo_TE_101362	0.97778(44/45)	0.43750(7/16)	0.68182(15/22)	0.40000(8/20)
Bo_TE_32948	0.43902(18/41)	0.50000(9/18)	1.00000(23/23)	0.95000(19/20)
Bo_TE_72997	0.04651(2/43)	0.61111(11/18)	0.36364(8/22)	0.63158(12/19)
Bo_TE_145914	0.63636(28/44)	0.05556(1/18)	0.00000(0/23)	0.00000(0/19)
Bo_TE_41883	0.18182(8/44)	0.35294(6/17)	0.86364(19/22)	0.83333(15/18)
Bo_TE_99826	0.72500(29/40)	0.64706(11/17)	0.04545(1/22)	0.00000(0/20)
Bo_TE_38919	0.02222(1/45)	0.11111(2/18)	0.17391(4/23)	0.61111(11/18)
Bo_TE_170785	0.48780(20/41)	0.63158(12/19)	0.00000(0/21)	0.52632(10/19)
Bo_TE_121788	0.58974(23/39)	1.00000(18/18)	0.95238(20/21)	0.20000(4/20)
Bo_TE_5064	0.41026(16/39)	1.00000(18/18)	1.00000(23/23)	1.00000(19/19)
Bo_TE_126823	0.56522(26/46)	0.05882(1/17)	0.00000(0/23)	0.31579(6/19)

Bo_TE_135178	0.67500(27/40)	0.23529(4/17)	1.00000(21/21)	0.94737(18/19)
Bo_TE_86025	0.35714(15/42)	0.05263(1/19)	0.00000(0/23)	0.89474(17/19)
Bo_TE_18042	0.61905(26/42)	0.25000(4/16)	0.00000(0/21)	0.05000(1/20)
Bo_TE_77690	0.62222(28/45)	0.00000(0/17)	0.00000(0/22)	0.00000(0/18)
Bo_TE_70255	0.23684(9/38)	0.50000(9/18)	0.19048(4/21)	0.84211(16/19)
Bo_TE_168118	0.30556(11/36)	1.00000(18/18)	0.55000(11/20)	0.50000(9/18)
Bo_TE_130313	0.00000(0/42)	0.88889(16/18)	0.60000(12/20)	0.10000(2/20)
Bo_TE_37398	0.00000(0/42)	0.63158(12/19)	0.00000(0/23)	0.05000(1/20)
Bo_TE_241807	0.77778(35/45)	0.05882(1/17)	0.50000(10/20)	0.10526(2/19)
Bo_TE_34852	0.24390(10/41)	0.00000(0/16)	0.80952(17/21)	0.50000(10/20)
Bo_TE_37117	0.45238(19/42)	0.37500(6/16)	1.00000(22/22)	0.88889(16/18)
Bo_TE_86090	0.58537(24/41)	0.15385(2/13)	0.00000(0/21)	0.14286(2/14)
Bo_TE_153835	0.00000(0/46)	0.50000(9/18)	0.27273(6/22)	0.36842(7/19)
Bo_TE_200299	0.65909(29/44)	0.47059(8/17)	0.09091(2/22)	0.22222(4/18)
Bo_TE_232229	0.30233(13/43)	0.84211(16/19)	0.81818(18/22)	0.90000(18/20)
Bo_TE_105710	0.97778(44/45)	1.00000(19/19)	0.66667(14/21)	0.31579(6/19)
Bo_TE_36203	1.00000(43/43)	1.00000(18/18)	0.22727(5/22)	1.00000(20/20)
Bo_TE_141866	0.00000(0/44)	0.05263(1/19)	0.77273(17/22)	0.15789(3/19)
Bo_TE_180328	0.20930(9/43)	0.22222(4/18)	0.95455(21/22)	0.72222(13/18)
Bo_TE_156939	0.45238(19/42)	0.11111(2/18)	0.76190(16/21)	0.27778(5/18)
Bo_TE_54233	0.64103(25/39)	0.64286(9/14)	0.76190(16/21)	0.22222(4/18)
Bo_TE_101291	0.04348(2/46)	0.13333(2/15)	0.50000(11/22)	0.73684(14/19)
Bo_TE_107445	0.13889(5/36)	0.50000(8/16)	0.00000(0/20)	0.12500(2/16)
Bo_TE_177584	0.00000(0/42)	0.00000(0/19)	0.40000(8/20)	0.52632(10/19)
Bo_TE_133919	0.16279(7/43)	0.38889(7/18)	0.45000(9/20)	0.94737(18/19)
Bo_TE_185090	0.02174(1/46)	0.00000(0/16)	0.42857(9/21)	0.63158(12/19)
Bo_TE_68037	0.41935(13/31)	0.12500(2/16)	0.85000(17/20)	0.25000(5/20)
Bo_TE_66669	0.57143(24/42)	0.12500(2/16)	0.08696(2/23)	0.05000(1/20)
Bo_TE_152174	0.66667(30/45)	0.11111(2/18)	0.13043(3/23)	0.00000(0/19)
Bo_TE_92675	0.00000(0/42)	0.00000(0/19)	0.70000(14/20)	0.22222(4/18)
Bo_TE_156148	0.20000(8/40)	0.77778(14/18)	1.00000(21/21)	0.86667(13/15)
Bo_TE_126946	0.28571(12/42)	0.66667(12/18)	0.35000(7/20)	0.90000(18/20)
Bo_TE_70753	0.94737(36/38)	0.94118(16/17)	0.82609(19/23)	0.42105(8/19)
Bo_TE_238674	0.87500(35/40)	0.55556(10/18)	0.10000(2/20)	0.30000(6/20)
Bo_TE_141188	0.06977(3/43)	0.64706(11/17)	0.17391(4/23)	0.84211(16/19)
Bo_TE_213805	0.86364(38/44)	0.94444(17/18)	0.30000(6/20)	0.50000(9/18)
Bo_TE_108670	0.37500(15/40)	0.00000(0/16)	0.40000(8/20)	0.52632(10/19)
Bo_TE_137605	0.15556(7/45)	0.12500(2/16)	0.19048(4/21)	0.75000(15/20)
Bo_TE_31645	0.62791(27/43)	0.00000(0/16)	0.65000(13/20)	0.85000(17/20)
Bo_TE_61743	0.32558(14/43)	0.31250(5/16)	0.68421(13/19)	0.85000(17/20)
Bo_TE_141533	0.11628(5/43)	0.91667(11/12)	0.93333(14/15)	0.73684(14/19)
Bo_TE_10247	0.02222(1/45)	0.00000(0/17)	0.00000(0/22)	0.55000(11/20)
Bo_TE_123962	0.76923(30/39)	1.00000(17/17)	0.90909(20/22)	0.11111(2/18)
Bo_TE_3737	0.18182(8/44)	0.68750(11/16)	0.50000(11/22)	0.73684(14/19)
Bo_TE_197658	0.93182(41/44)	0.38889(7/18)	0.95000(19/20)	0.17647(3/17)
Bo_TE_145695	0.97826(45/46)	0.18750(3/16)	0.80952(17/21)	0.70000(14/20)
Bo_TE_75612	0.00000(0/45)	0.63158(12/19)	0.00000(0/22)	0.05000(1/20)
Bo_TE_17839	0.22727(10/44)	0.05882(1/17)	0.04545(1/22)	0.63158(12/19)
Bo_TE_70425	0.11628(5/43)	0.05882(1/17)	0.60870(14/23)	0.10526(2/19)
Bo_TE_150443	0.05714(2/35)	0.05882(1/17)	0.19048(4/21)	0.83333(15/18)
Bo_TE_64147	0.52381(22/42)	0.05882(1/17)	0.00000(0/21)	0.10000(2/20)
Bo_TE_191773	0.09091(4/44)	0.55556(10/18)	0.04348(1/23)	0.10000(2/20)
Bo_TE_53862	0.57500(23/40)	0.05882(1/17)	0.19048(4/21)	0.26316(5/19)
Bo_TE_172928	0.00000(0/43)	0.00000(0/18)	0.57143(12/21)	0.73684(14/19)
Bo_TE_98930	0.02174(1/46)	0.00000(0/19)	0.50000(11/22)	0.25000(5/20)
Bo_TE_137733	0.37209(16/43)	0.68750(11/16)	0.04762(1/21)	0.15789(3/19)

Bo_TE_86582	0.00000(0/45)	0.00000(0/18)	0.04545(1/22)	0.60000(12/20)
Bo_TE_42398	0.44444(20/45)	0.68750(11/16)	0.95238(20/21)	0.60000(12/20)
Bo_TE_126215	0.55814(24/43)	0.41176(7/17)	0.90909(20/22)	1.00000(20/20)
Bo_TE_126385	0.09524(4/42)	0.31250(5/16)	0.59091(13/22)	0.73684(14/19)
Bo_TE_86078	0.00000(0/44)	0.00000(0/19)	0.00000(0/23)	0.68421(13/19)
Bo_TE_125169	0.02326(1/43)	0.11765(2/17)	0.00000(0/23)	0.60000(12/20)
Bo_TE_207194	0.41860(18/43)	0.66667(12/18)	0.68421(13/19)	0.10526(2/19)
Bo_TE_78089	0.22727(10/44)	0.77778(14/18)	0.41176(7/17)	0.31579(6/19)
Bo_TE_32664	0.75610(31/41)	0.12500(2/16)	0.00000(0/22)	0.16667(3/18)
Bo_TE_143370	0.63636(28/44)	0.66667(12/18)	0.00000(0/22)	0.00000(0/20)
Bo_TE_69394	0.18421(7/38)	0.55556(10/18)	0.54545(12/22)	1.00000(20/20)
Bo_TE_109452	0.89130(41/46)	0.38462(5/13)	0.09524(2/21)	0.57895(11/19)
Bo_TE_216924	0.57143(24/42)	0.41176(7/17)	0.00000(0/20)	0.15000(3/20)
Bo_TE_216455	0.26316(10/38)	0.05882(1/17)	0.76190(16/21)	0.55556(10/18)
Bo_TE_133988	0.11364(5/44)	0.55556(10/18)	0.42857(9/21)	0.76471(13/17)
Bo_TE_109412	0.66667(28/42)	0.17647(3/17)	0.04762(1/21)	0.15789(3/19)
Bo_TE_213227	0.34884(15/43)	0.82353(14/17)	1.00000(22/22)	0.89474(17/19)
Bo_TE_125774	0.22500(9/40)	0.11111(2/18)	0.15000(3/20)	0.78947(15/19)
Bo_TE_220566	0.61364(27/44)	0.83333(15/18)	0.55000(11/20)	0.25000(5/20)
Bo_TE_36418	0.88095(37/42)	0.00000(0/17)	0.00000(0/21)	0.21053(4/19)
Bo_TE_43068	0.83333(30/36)	0.57895(11/19)	0.88235(15/17)	0.05556(1/18)
Bo_TE_27527	0.17949(7/39)	0.94444(17/18)	1.00000(19/19)	1.00000(16/16)
Bo_TE_26708	0.00000(0/45)	0.00000(0/18)	0.00000(0/22)	0.66667(12/18)
Bo_TE_96815	0.53846(21/39)	0.17647(3/17)	0.80952(17/21)	0.00000(0/19)
Bo_TE_169859	0.39130(18/46)	0.77778(14/18)	1.00000(23/23)	0.90000(18/20)
Bo_TE_228094	1.00000(46/46)	1.00000(18/18)	0.23810(5/21)	0.55000(11/20)
Bo_TE_71774	0.73810(31/42)	0.44444(8/18)	0.14286(3/21)	0.40000(8/20)
Bo_TE_44190	1.00000(45/45)	0.21429(3/14)	0.66667(14/21)	0.25000(5/20)
Bo_TE_69523	0.37209(16/43)	0.66667(12/18)	0.14286(3/21)	0.00000(0/20)
Bo_TE_7961	0.06667(3/45)	0.00000(0/18)	0.66667(14/21)	0.00000(0/17)
Bo_TE_32608	0.69231(27/39)	0.06250(1/16)	0.00000(0/19)	0.25000(4/16)
Bo_TE_103194	0.88095(37/42)	0.29412(5/17)	0.50000(10/20)	0.00000(0/19)
Bo_TE_100230	0.18605(8/43)	0.00000(0/18)	0.61905(13/21)	0.52941(9/17)
Bo_TE_117213	0.00000(0/42)	0.00000(0/16)	0.28571(6/21)	0.50000(8/16)
Bo_TE_54326	0.95455(42/44)	0.41176(7/17)	0.77273(17/22)	0.45000(9/20)
Bo_TE_141409	0.11628(5/43)	0.85714(12/14)	0.95238(20/21)	0.78947(15/19)
Bo_TE_137782	0.31707(13/41)	0.61111(11/18)	0.80952(17/21)	0.05000(1/20)
Bo_TE_131243	0.41860(18/43)	0.94444(17/18)	0.95652(22/23)	0.90000(18/20)
Bo_TE_67629	0.76744(33/43)	0.94118(16/17)	0.25000(5/20)	0.76471(13/17)
Bo_TE_175096	0.72093(31/43)	0.93750(15/16)	0.61905(13/21)	0.11111(2/18)
Bo_TE_96640	0.83721(36/43)	0.36842(7/19)	0.20000(4/20)	0.31579(6/19)
Bo_TE_227509	0.00000(0/43)	0.00000(0/19)	0.27273(6/22)	0.57895(11/19)
Bo_TE_128591	0.27273(12/44)	0.76471(13/17)	0.55000(11/20)	0.00000(0/20)
Bo_TE_117650	0.33333(14/42)	0.64706(11/17)	0.68182(15/22)	0.94737(18/19)
Bo_TE_15847	0.85714(36/42)	0.75000(12/16)	0.60000(12/20)	0.16667(3/18)
Bo_TE_1053	0.02222(1/45)	0.31250(5/16)	0.14286(3/21)	0.89474(17/19)
Bo_TE_230794	0.48780(20/41)	0.50000(8/16)	0.66667(14/21)	1.00000(20/20)
Bo_TE_41128	0.95349(41/43)	1.00000(18/18)	0.95455(21/22)	0.27778(5/18)
Bo_TE_50208	0.54054(20/37)	0.06250(1/16)	0.00000(0/23)	0.27273(3/11)
Bo_TE_178632	0.16667(7/42)	0.25000(2/8)	0.83333(15/18)	0.87500(14/16)
Bo_TE_185114	0.23256(10/43)	0.25000(4/16)	0.61905(13/21)	0.95000(19/20)
Bo_TE_73081	0.09756(4/41)	0.62500(10/16)	0.38889(7/18)	0.60000(12/20)
Bo_TE_205295	0.00000(0/45)	0.00000(0/17)	0.47368(9/19)	0.89474(17/19)
Bo_TE_190913	0.02222(1/45)	0.05263(1/19)	0.00000(0/23)	0.68421(13/19)
Bo_TE_120737	0.71053(27/38)	0.31250(5/16)	0.45000(9/20)	0.84211(16/19)
Bo_TE_196563	0.39535(17/43)	1.00000(17/17)	1.00000(22/22)	1.00000(20/20)

Bo_TE_67389	0.50000(21/42)	0.88235(15/17)	0.25000(5/20)	0.42105(8/19)
Bo_TE_156985	0.42857(18/42)	0.06250(1/16)	0.04348(1/23)	0.70000(14/20)
Bo_TE_182789	0.57143(24/42)	0.11111(2/18)	0.19048(4/21)	0.05263(1/19)
Bo_TE_230007	0.90000(36/40)	0.33333(5/15)	0.52632(10/19)	0.38889(7/18)
Bo_TE_163794	0.90698(39/43)	0.31250(5/16)	0.70000(14/20)	0.20000(4/20)
Bo_TE_165546	0.60465(26/43)	0.05556(1/18)	0.00000(0/23)	0.00000(0/20)
Bo_TE_71421	0.02222(1/45)	0.52941(9/17)	0.00000(0/22)	0.20000(4/20)
Bo_TE_202059	0.78947(30/38)	0.00000(0/17)	0.05000(1/20)	0.16667(3/18)
Bo_TE_235626	0.51163(22/43)	0.00000(0/17)	0.00000(0/22)	0.26316(5/19)
Bo_TE_221284	0.97826(45/46)	0.17647(3/17)	0.95652(22/23)	0.94737(18/19)
Bo_TE_66878	0.86047(37/43)	0.87500(14/16)	0.04545(1/22)	0.10526(2/19)
Bo_TE_76343	0.65116(28/43)	0.06250(1/16)	0.80952(17/21)	0.41176(7/17)
Bo_TE_170829	0.00000(0/44)	0.11111(2/18)	0.50000(10/20)	0.00000(0/20)
Bo_TE_144708	0.85366(35/41)	0.05556(1/18)	0.09091(2/22)	0.68421(13/19)
Bo_TE_223693	0.82927(34/41)	0.93750(15/16)	0.40909(9/22)	1.00000(20/20)
Bo_TE_163190	0.02222(1/45)	0.05556(1/18)	0.57143(12/21)	0.73684(14/19)
Bo_TE_236769	0.15000(6/40)	0.22222(4/18)	0.00000(0/21)	0.55556(10/18)
Bo_TE_160601	0.00000(0/45)	0.20000(3/15)	0.57143(12/21)	0.22222(4/18)
Bo_TE_23151	0.78378(29/37)	0.05556(1/18)	0.00000(0/23)	0.05000(1/20)
Bo_TE_62878	0.00000(0/45)	0.56250(9/16)	0.45000(9/20)	0.00000(0/20)
Bo_TE_28945	0.81395(35/43)	0.06250(1/16)	0.77273(17/22)	0.00000(0/16)
Bo_TE_194257	0.00000(0/43)	0.05882(1/17)	0.33333(7/21)	0.57895(11/19)
Bo_TE_222232	0.90476(38/42)	0.11765(2/17)	1.00000(23/23)	0.30000(6/20)
Bo_TE_67647	0.13636(6/44)	0.06250(1/16)	0.77273(17/22)	0.33333(6/18)
Bo_TE_229872	0.72500(29/40)	0.25000(4/16)	0.63636(14/22)	0.95000(19/20)
Bo_TE_232581	1.00000(44/44)	1.00000(18/18)	0.60000(12/20)	0.45000(9/20)
Bo_TE_121193	0.29268(12/41)	0.27778(5/18)	0.57143(12/21)	0.05000(1/20)
Bo_TE_151957	0.62162(23/37)	0.00000(0/18)	0.42857(9/21)	0.15000(3/20)
Bo_TE_41321	0.86842(33/38)	0.68421(13/19)	0.42105(8/19)	0.21053(4/19)
Bo_TE_229137	0.53846(21/39)	0.29412(5/17)	0.95455(21/22)	1.00000(18/18)
Bo_TE_234959	0.57778(26/45)	0.27778(5/18)	0.00000(0/22)	0.26316(5/19)
Bo_TE_1064	1.00000(45/45)	0.81250(13/16)	0.54545(12/22)	0.50000(9/18)
Bo_TE_219376	0.36364(16/44)	1.00000(18/18)	0.94737(18/19)	0.78947(15/19)
Bo_TE_132946	0.22727(10/44)	0.43750(7/16)	0.80952(17/21)	0.05263(1/19)
Bo_TE_152204	0.72093(31/43)	0.31250(5/16)	1.00000(22/22)	1.00000(17/17)
Bo_TE_203181	0.72500(29/40)	0.77778(14/18)	0.50000(11/22)	0.05000(1/20)
Bo_TE_81868	0.00000(0/43)	0.68750(11/16)	0.26316(5/19)	0.10526(2/19)
Bo_TE_90912	0.35714(15/42)	0.66667(12/18)	0.42105(8/19)	0.05882(1/17)
Bo_TE_88431	0.15909(7/44)	0.94118(16/17)	0.85000(17/20)	0.77778(14/18)
Bo_TE_183427	0.00000(0/43)	0.00000(0/18)	0.18182(4/22)	0.68421(13/19)
Bo_TE_68077	0.33333(8/24)	0.87500(14/16)	0.00000(0/3)	0.21053(4/19)
Bo_TE_112319	0.00000(0/41)	0.22222(4/18)	0.71429(15/21)	0.52632(10/19)
Bo_TE_101599	0.00000(0/43)	0.00000(0/14)	0.59091(13/22)	0.00000(0/19)
Bo_TE_214337	0.11364(5/44)	0.11765(2/17)	0.63636(14/22)	0.42105(8/19)
Bo_TE_82426	0.30769(12/39)	0.50000(8/16)	0.00000(0/20)	0.15789(3/19)
Bo_TE_236211	0.00000(0/45)	0.05882(1/17)	0.68421(13/19)	0.00000(0/20)
Bo_TE_191482	0.22727(10/44)	1.00000(17/17)	1.00000(23/23)	1.00000(19/19)
Bo_TE_175454	0.04762(2/42)	0.05882(1/17)	0.04348(1/23)	0.61111(11/18)
Bo_TE_223325	0.72500(29/40)	0.18750(3/16)	0.91304(21/23)	0.90000(18/20)
Bo_TE_131231	0.15909(7/44)	0.00000(0/17)	0.81818(18/22)	0.38889(7/18)
Bo_TE_111601	0.84091(37/44)	0.83333(15/18)	0.23810(5/21)	0.88889(16/18)
Bo_TE_89737	0.38636(17/44)	0.64706(11/17)	0.00000(0/23)	0.05263(1/19)
Bo_TE_182418	0.06522(3/46)	0.64706(11/17)	0.57143(12/21)	0.50000(9/18)
Bo_TE_180773	0.23810(10/42)	0.66667(12/18)	1.00000(22/22)	0.75000(15/20)
Bo_TE_67803	0.06818(3/44)	0.37500(6/16)	0.28571(6/21)	0.80000(16/20)
Bo_TE_15465	0.77273(34/44)	0.05882(1/17)	0.00000(0/22)	0.52632(10/19)

Bo_TE_174047	0.29545(13/44)	0.22222(4/18)	0.54545(12/22)	0.78947(15/19)
Bo_TE_43522	0.75000(30/40)	0.11111(2/18)	0.00000(0/21)	0.44444(8/18)
Bo_TE_213975	0.65789(25/38)	0.75000(12/16)	0.42857(9/21)	0.10000(2/20)
Bo_TE_31280	0.64286(27/42)	0.23529(4/17)	0.09524(2/21)	0.35000(7/20)
Bo_TE_75722	0.97727(43/44)	0.35294(6/17)	0.80952(17/21)	0.90000(18/20)
Bo_TE_40715	0.79545(35/44)	0.86667(13/15)	0.78947(15/19)	0.25000(5/20)
Bo_TE_122853	0.44737(17/38)	0.00000(0/18)	0.63636(14/22)	0.47059(8/17)
Bo_TE_46929	0.65116(28/43)	0.35294(6/17)	0.85000(17/20)	0.95000(19/20)
Bo_TE_33979	0.69048(29/42)	0.43750(7/16)	0.68421(13/19)	0.15000(3/20)
Bo_TE_237880	0.39474(15/38)	0.58824(10/17)	1.00000(23/23)	1.00000(20/20)
Bo_TE_92199	0.00000(0/43)	0.00000(0/18)	0.00000(0/19)	0.50000(10/20)
Bo_TE_162057	0.53846(21/39)	0.17647(3/17)	0.00000(0/22)	0.00000(0/20)
Bo_TE_240609	0.11628(5/43)	0.77778(14/18)	0.95652(22/23)	0.36842(7/19)
Bo_TE_198576	0.86957(40/46)	0.83333(15/18)	0.65000(13/20)	0.05000(1/20)
Bo_TE_216159	0.06667(3/45)	0.33333(6/18)	0.17391(4/23)	0.60000(12/20)
Bo_TE_112340	0.00000(0/44)	0.22222(4/18)	0.75000(15/20)	0.52632(10/19)
Bo_TE_58061	0.25000(10/40)	0.75000(12/16)	0.45455(10/22)	0.50000(5/10)
Bo_TE_205241	0.02222(1/45)	0.00000(0/19)	0.50000(10/20)	0.05000(1/20)
Bo_TE_10373	0.35897(14/39)	0.87500(14/16)	0.57143(12/21)	0.80000(16/20)
Bo_TE_179598	0.97143(34/35)	0.94118(16/17)	0.52632(10/19)	0.37500(6/16)
Bo_TE_231999	0.00000(0/44)	0.00000(0/17)	0.40000(8/20)	0.52632(10/19)
Bo_TE_104764	0.79070(34/43)	0.35294(6/17)	1.00000(22/22)	1.00000(20/20)
Bo_TE_224702	0.34211(13/38)	0.16667(1/6)	0.86364(19/22)	1.00000(19/19)
Bo_TE_17573	0.60000(24/40)	0.27778(5/18)	0.85000(17/20)	0.00000(0/20)
Bo_TE_70348	0.19444(7/36)	0.56250(9/16)	0.00000(0/20)	0.06667(1/15)
Bo_TE_24477	0.36585(15/41)	0.16667(3/18)	1.00000(23/23)	0.88235(15/17)
Bo_TE_200743	0.82051(32/39)	0.15789(3/19)	0.10000(2/20)	0.00000(0/19)
Bo_TE_14178	0.74286(26/35)	0.88235(15/17)	0.73913(17/23)	0.05000(1/20)
Bo_TE_67636	0.25000(11/44)	0.05882(1/17)	0.73913(17/23)	0.15789(3/19)
Bo_TE_80002	0.02273(1/44)	0.00000(0/17)	0.09091(2/22)	0.60000(12/20)
Bo_TE_33180	0.02222(1/45)	0.44444(8/18)	0.38095(8/21)	0.77778(14/18)
Bo_TE_18337	0.37500(15/40)	0.25000(4/16)	0.85000(17/20)	0.05263(1/19)
Bo_TE_44020	0.14286(6/42)	0.76471(13/17)	0.04762(1/21)	0.10000(2/20)
Bo_TE_170146	0.27500(11/40)	0.68421(13/19)	1.00000(21/21)	0.60000(12/20)
Bo_TE_179859	0.09524(4/42)	0.64706(11/17)	0.61111(11/18)	0.75000(15/20)
Bo_TE_86286	0.09756(4/41)	0.26316(5/19)	0.68182(15/22)	0.00000(0/17)
Bo_TE_58326	1.00000(41/41)	0.84211(16/19)	0.31818(7/22)	0.36842(7/19)
Bo_TE_100792	0.18182(8/44)	0.05882(1/17)	0.09091(2/22)	0.77778(14/18)
Bo_TE_222378	0.02273(1/44)	0.72222(13/18)	0.00000(0/18)	0.00000(0/20)
Bo_TE_123487	1.00000(39/39)	0.93750(15/16)	0.30000(6/20)	0.44444(8/18)
Bo_TE_213054	0.79070(34/43)	0.10526(2/19)	0.36364(8/22)	0.21053(4/19)
Bo_TE_113976	0.00000(0/45)	0.57895(11/19)	0.00000(0/22)	0.00000(0/20)
Bo_TE_113979	0.50000(21/42)	0.35294(6/17)	1.00000(22/22)	1.00000(20/20)
Bo_TE_193878	0.60000(24/40)	0.35294(6/17)	0.18182(4/22)	0.80000(16/20)
Bo_TE_119405	0.95455(42/44)	0.37500(6/16)	0.36842(7/19)	0.90000(18/20)
Bo_TE_37575	0.27500(11/40)	0.76471(13/17)	0.55000(11/20)	0.90000(18/20)
Bo_TE_180999	0.30233(13/43)	0.23529(4/17)	0.75000(15/20)	0.20000(4/20)
Bo_TE_41922	0.75556(34/45)	0.35294(6/17)	0.05263(1/19)	0.15789(3/19)
Bo_TE_89937	0.05000(2/40)	0.44444(8/18)	0.36364(8/22)	0.70588(12/17)
Bo_TE_192204	0.66667(28/42)	0.87500(14/16)	0.45000(9/20)	0.00000(0/20)
Bo_TE_134810	0.85714(36/42)	0.33333(6/18)	0.86364(19/22)	0.94737(18/19)
Bo_TE_119686	0.35714(15/42)	0.88235(15/17)	0.42857(9/21)	1.00000(20/20)
Bo_TE_223418	0.00000(0/45)	0.69231(9/13)	0.60000(12/20)	0.57895(11/19)
Bo_TE_134258	0.53488(23/43)	0.88889(16/18)	0.59091(13/22)	0.15789(3/19)
Bo_TE_749	0.90244(37/41)	0.37500(6/16)	0.21739(5/23)	0.22222(4/18)
Bo_TE_53874	0.09091(4/44)	0.88889(16/18)	0.28571(6/21)	0.61111(11/18)

Bo_TE_91665	0.00000(0/46)	0.10526(2/19)	0.35000(7/20)	0.61111(11/18)
Bo_TE_63989	0.02273(1/44)	0.16667(3/18)	0.57143(12/21)	0.00000(0/19)
Bo_TE_164019	1.00000(45/45)	0.47059(8/17)	0.66667(14/21)	0.52632(10/19)
Bo_TE_10281	0.27907(12/43)	0.94444(17/18)	0.31818(7/22)	0.63158(12/19)
Bo_TE_123852	0.59524(25/42)	0.00000(0/17)	0.33333(7/21)	0.47368(9/19)
Bo_TE_191406	0.02174(1/46)	0.72222(13/18)	0.76190(16/21)	0.05000(1/20)
Bo_TE_97306	0.00000(0/43)	0.38889(7/18)	0.73913(17/23)	0.83333(15/18)
Bo_TE_35765	0.80000(32/40)	0.11765(2/17)	0.13043(3/23)	0.31579(6/19)
Bo_TE_182325	0.07143(3/42)	0.61111(11/18)	0.73684(14/19)	0.44444(8/18)
Bo_TE_195920	0.66667(28/42)	0.88889(16/18)	0.66667(14/21)	0.33333(6/18)
Bo_TE_237817	0.29268(12/41)	0.88235(15/17)	0.00000(0/23)	0.10526(2/19)
Bo_TE_189615	0.35714(15/42)	0.18750(3/16)	0.42857(9/21)	1.00000(20/20)
Bo_TE_224509	0.00000(0/44)	0.00000(0/18)	0.19048(4/21)	0.80000(16/20)
Bo_TE_134381	0.33333(14/42)	0.82353(14/17)	0.94737(18/19)	0.85714(12/14)
Bo_TE_97533	0.65116(28/43)	0.76471(13/17)	0.04348(1/23)	0.00000(0/19)
Bo_TE_108942	0.56098(23/41)	0.88235(15/17)	0.44444(8/18)	0.30000(6/20)
Bo_TE_179890	0.83333(35/42)	0.44444(8/18)	0.43478(10/23)	0.00000(0/18)
Bo_TE_142722	0.40000(16/40)	0.00000(0/19)	0.34783(8/23)	0.60000(12/20)
Bo_TE_236652	0.97826(45/46)	0.52941(9/17)	0.19048(4/21)	1.00000(19/19)
Bo_TE_129227	0.34146(14/41)	0.00000(0/18)	0.00000(0/21)	0.68421(13/19)
Bo_TE_81560	1.00000(46/46)	0.77778(14/18)	0.13636(3/22)	1.00000(20/20)
Bo_TE_88110	0.04444(2/45)	0.64706(11/17)	0.00000(0/20)	0.05000(1/20)
Bo_TE_182835	0.00000(0/42)	0.05263(1/19)	0.63636(14/22)	0.36842(7/19)
Bo_TE_70013	0.61905(26/42)	1.00000(16/16)	0.19048(4/21)	0.47368(9/19)
Bo_TE_172836	0.47500(19/40)	0.31250(5/16)	0.86364(19/22)	0.41176(7/17)
Bo_TE_62764	0.02174(1/46)	0.62500(10/16)	0.45455(10/22)	0.00000(0/20)
Bo_TE_77334	0.80488(33/41)	0.25000(4/16)	0.90909(20/22)	0.55000(11/20)
Bo_TE_236404	0.35294(12/34)	0.87500(14/16)	0.33333(6/18)	0.10526(2/19)
Bo_TE_136810	0.60000(24/40)	0.23529(4/17)	0.04348(1/23)	0.10000(2/20)
Bo_TE_195529	0.37209(16/43)	0.35294(6/17)	0.80952(17/21)	0.05000(1/20)
Bo_TE_65563	0.95000(38/40)	0.35294(6/17)	0.81818(18/22)	0.85000(17/20)
Bo_TE_126121	0.71429(30/42)	0.05263(1/19)	0.00000(0/23)	0.00000(0/20)
Bo_TE_92023	0.56410(22/39)	0.00000(0/17)	0.00000(0/22)	0.52632(10/19)
Bo_TE_158778	0.77778(35/45)	0.06250(1/16)	0.63636(14/22)	0.42105(8/19)
Bo_TE_163347	0.02564(1/39)	0.58824(10/17)	0.00000(0/18)	0.00000(0/20)
Bo_TE_194685	0.16667(7/42)	0.11111(2/18)	0.65000(13/20)	0.36842(7/19)
Bo_TE_182513	0.04444(2/45)	0.00000(0/18)	0.56522(13/23)	0.31579(6/19)
Bo_TE_21458	0.78049(32/41)	0.29412(5/17)	0.04545(1/22)	0.05556(1/18)
Bo_TE_17508	0.92857(39/42)	0.17647(3/17)	0.16667(3/18)	0.26316(5/19)
Bo_TE_41504	0.11111(5/45)	0.52632(10/19)	0.00000(0/19)	0.16667(3/18)
Bo_TE_119544	0.30952(13/42)	0.00000(0/18)	0.76190(16/21)	0.40000(8/20)
Bo_TE_25025	0.04878(2/41)	0.33333(6/18)	0.00000(0/22)	0.85000(17/20)
Bo_TE_20355	0.16667(4/24)	0.83333(15/18)	0.14286(3/21)	0.17647(3/17)
Bo_TE_82202	0.75610(31/41)	0.27778(5/18)	0.38095(8/21)	0.05000(1/20)
Bo_TE_130180	0.32558(14/43)	0.58824(10/17)	1.00000(23/23)	0.94737(18/19)
Bo_TE_184190	0.09524(4/42)	0.70588(12/17)	0.00000(0/21)	0.05000(1/20)
Bo_TE_169196	0.16667(7/42)	0.73684(14/19)	0.14286(3/21)	0.15000(3/20)
Bo_TE_133299	0.35714(10/28)	0.16667(1/6)	0.86364(19/22)	0.29412(5/17)
Bo_TE_101618	0.50000(21/42)	0.33333(5/15)	0.00000(0/19)	0.10000(2/20)
Bo_TE_32208	0.93478(43/46)	0.12500(2/16)	0.85714(6/7)	0.20000(4/20)
Bo_TE_235242	0.06667(3/45)	0.16667(3/18)	0.04545(1/22)	0.80000(16/20)
Bo_TE_53331	0.00000(0/44)	0.43750(7/16)	0.47826(11/23)	0.72222(13/18)
Bo_TE_202037	0.15909(7/44)	0.47368(9/19)	0.66667(14/21)	0.15000(3/20)
Bo_TE_124965	0.58333(21/36)	0.88235(15/17)	0.45000(9/20)	0.21053(4/19)
Bo_TE_50626	0.60976(25/41)	0.88889(16/18)	0.91304(21/23)	0.15789(3/19)
Bo_TE_225354	0.07143(3/42)	0.76471(13/17)	0.00000(0/23)	0.05000(1/20)

Bo_TE_13864	0.40476(17/42)	0.18750(3/16)	1.00000(23/23)	1.00000(20/20)
Bo_TE_223047	0.03571(1/28)	0.13333(2/15)	0.75000(15/20)	0.00000(0/17)
Bo_TE_119484	0.14286(6/42)	0.00000(0/18)	0.59091(13/22)	0.15000(3/20)
Bo_TE_55007	0.93182(41/44)	0.50000(8/16)	0.14286(3/21)	0.52632(10/19)
Bo_TE_221709	0.04545(2/44)	0.00000(0/13)	0.05882(1/17)	0.55556(10/18)
Bo_TE_128423	0.73171(30/41)	0.27778(5/18)	0.33333(7/21)	1.00000(19/19)
Bo_TE_36368	0.00000(0/45)	0.00000(0/19)	0.50000(11/22)	0.10526(2/19)
Bo_TE_177890	0.97619(41/42)	1.00000(19/19)	0.95000(19/20)	0.38889(7/18)
Bo_TE_41169	0.95349(41/43)	0.29412(5/17)	1.00000(23/23)	0.90000(18/20)
Bo_TE_103445	0.02174(1/46)	0.61111(11/18)	0.28571(6/21)	0.22222(4/18)
Bo_TE_208328	0.33333(14/42)	0.61111(11/18)	0.90000(18/20)	0.89474(17/19)
Bo_TE_158613	0.41860(18/43)	0.94118(16/17)	0.13636(3/22)	0.68421(13/19)
Bo_TE_138633	0.61905(26/42)	0.13333(2/15)	0.00000(0/23)	0.21053(4/19)
Bo_TE_210353	0.88636(39/44)	0.58824(10/17)	0.00000(0/22)	0.05000(1/20)
Bo_TE_96436	0.31707(13/41)	0.52941(9/17)	0.00000(0/21)	0.26316(5/19)
Bo_TE_228236	0.00000(0/45)	0.00000(0/19)	0.80000(16/20)	0.44444(8/18)
Bo_TE_151673	0.06818(3/44)	0.11111(2/18)	0.45000(9/20)	0.61111(11/18)
Bo_TE_57660	0.58537(24/41)	0.58824(10/17)	0.42857(9/21)	0.00000(0/20)
Bo_TE_10553	0.35714(15/42)	0.23529(4/17)	0.42105(8/19)	1.00000(20/20)
Bo_TE_12818	0.11905(5/42)	0.00000(0/17)	0.54545(12/22)	0.47368(9/19)
Bo_TE_25890	0.80952(34/42)	0.14286(2/14)	0.52632(10/19)	0.10526(2/19)
Bo_TE_234176	0.83721(36/43)	0.35294(6/17)	0.00000(0/23)	0.15000(3/20)
Bo_TE_124135	0.79545(35/44)	0.26316(5/19)	0.18182(4/22)	0.15000(3/20)
Bo_TE_182205	0.81818(36/44)	0.27778(5/18)	1.00000(22/22)	0.70000(14/20)
Bo_TE_96664	0.17949(7/39)	0.56250(9/16)	0.90909(20/22)	0.47368(9/19)
Bo_TE_205644	0.21951(9/41)	0.64706(11/17)	0.09524(2/21)	0.00000(0/20)
Bo_TE_104611	0.03448(1/29)	0.00000(0/16)	0.63158(12/19)	0.00000(0/4)
Bo_TE_108199	0.00000(0/45)	0.35294(6/17)	0.60000(12/20)	0.33333(6/18)
Bo_TE_185147	0.20455(9/44)	0.12500(2/16)	0.47619(10/21)	0.70000(14/20)
Bo_TE_190897	0.42500(17/40)	0.23529(4/17)	0.04762(1/21)	0.63158(12/19)
Bo_TE_69118	0.88889(40/45)	0.41176(7/17)	0.30435(7/23)	0.94444(17/18)
Bo_TE_206982	0.72093(31/43)	0.62500(10/16)	0.00000(0/22)	0.44444(8/18)
Bo_TE_24338	0.09091(4/44)	0.05882(1/17)	0.00000(0/23)	0.68750(11/16)
Bo_TE_18947	0.87805(36/41)	0.75000(12/16)	0.04545(1/22)	0.15789(3/19)
Bo_TE_135347	0.57500(23/40)	0.29412(5/17)	0.35000(7/20)	0.00000(0/20)
Bo_TE_138667	0.37209(16/43)	0.68750(11/16)	0.85000(17/20)	0.15000(3/20)
Bo_TE_16183	0.00000(0/45)	0.82353(14/17)	0.09524(2/21)	0.40000(8/20)
Bo_TE_8948	0.00000(0/46)	0.00000(0/16)	0.66667(14/21)	0.58824(10/17)
Bo_TE_235631	0.75000(33/44)	0.11765(2/17)	1.00000(22/22)	0.70000(14/20)
Bo_TE_50570	0.64286(27/42)	0.94118(16/17)	0.91304(21/23)	0.15000(3/20)
Bo_TE_159996	0.65854(27/41)	0.18750(3/16)	0.90909(20/22)	0.47059(8/17)
Bo_TE_153754	0.57500(23/40)	0.00000(0/18)	0.00000(0/23)	0.05000(1/20)
Bo_TE_39904	0.56410(22/39)	0.58824(10/17)	0.68421(13/19)	0.00000(0/18)
Bo_TE_44147	0.06818(3/44)	0.00000(0/18)	0.05000(1/20)	0.57895(11/19)
Bo_TE_93350	0.64286(27/42)	0.58824(10/17)	0.00000(0/21)	0.47368(9/19)
Bo_TE_213780	0.84091(37/44)	0.35294(6/17)	0.90909(20/22)	0.80000(16/20)
Bo_TE_186347	0.27907(12/43)	0.05263(1/19)	0.04348(1/23)	0.57895(11/19)
Bo_TE_206726	0.46512(20/43)	0.82353(14/17)	1.00000(22/22)	0.63158(12/19)
Bo_TE_236873	0.48780(20/41)	0.64706(11/17)	0.23810(5/21)	0.10000(2/20)
Bo_TE_198086	0.41860(18/43)	1.00000(17/17)	0.75000(15/20)	1.00000(19/19)
Bo_TE_122478	0.97778(44/45)	1.00000(18/18)	0.19048(4/21)	0.83333(15/18)
Bo_TE_52646	0.51220(21/41)	0.73684(14/19)	0.05556(1/18)	0.05263(1/19)
Bo_TE_85455	0.59524(25/42)	0.10526(2/19)	0.00000(0/22)	0.00000(0/20)
Bo_TE_30865	0.78571(33/42)	0.70588(12/17)	0.20000(4/20)	0.72222(13/18)
Bo_TE_225175	0.35714(15/42)	0.70588(12/17)	0.04348(1/23)	0.44444(8/18)
Bo_TE_211139	0.02222(1/45)	0.00000(0/19)	0.75000(15/20)	0.66667(12/18)

Bo_TE_226728	0.97826(45/46)	0.94444(17/18)	0.27273(6/22)	0.95000(19/20)
Bo_TE_195404	0.83721(36/43)	0.31250(5/16)	1.00000(21/21)	0.47368(9/19)
Bo_TE_212069	0.16279(7/43)	0.07143(1/14)	0.75000(15/20)	0.13333(2/15)
Bo_TE_172331	0.74359(29/39)	1.00000(17/17)	0.19048(4/21)	0.11765(2/17)
Bo_TE_30516	0.00000(0/43)	0.00000(0/19)	0.75000(15/20)	0.00000(0/19)
Bo_TE_84824	0.00000(0/44)	0.05263(1/19)	0.72727(16/22)	0.55000(11/20)
Bo_TE_215559	0.57778(26/45)	0.47368(9/19)	0.42857(9/21)	1.00000(20/20)
Bo_TE_226704	0.33333(14/42)	0.17647(3/17)	0.66667(14/21)	0.10526(2/19)
Bo_TE_67068	0.51282(20/39)	0.00000(0/19)	0.00000(0/23)	0.00000(0/20)
Bo_TE_122841	0.72093(31/43)	0.22222(4/18)	0.72727(16/22)	0.94737(18/19)
Bo_TE_132223	0.30769(12/39)	0.62500(10/16)	1.00000(19/19)	1.00000(19/19)
Bo_TE_236747	0.00000(0/46)	0.17647(3/17)	0.50000(11/22)	0.00000(0/20)
Bo_TE_58538	0.08889(4/45)	0.75000(12/16)	0.19048(4/21)	0.26316(5/19)
Bo_TE_172372	0.61538(24/39)	0.00000(0/15)	0.94737(18/19)	0.37500(6/16)
Bo_TE_94594	0.02222(1/45)	0.11111(2/18)	0.13043(3/23)	0.57895(11/19)
Bo_TE_130157	0.68293(28/41)	0.47059(8/17)	0.04348(1/23)	0.05263(1/19)
Bo_TE_149646	0.79070(34/43)	0.94444(17/18)	0.42105(8/19)	0.47368(9/19)
Bo_TE_153083	0.00000(0/43)	0.82353(14/17)	0.65000(13/20)	0.57895(11/19)
Bo_TE_153404	0.00000(0/45)	0.55556(10/18)	0.00000(0/23)	0.00000(0/19)
Bo_TE_240362	0.79070(34/43)	0.17647(3/17)	0.63636(14/22)	0.50000(10/20)
Bo_TE_111646	0.20455(9/44)	0.33333(5/15)	0.00000(0/23)	0.68421(13/19)
Bo_TE_145539	0.64286(27/42)	0.35294(6/17)	1.00000(23/23)	1.00000(19/19)
Bo_TE_209965	0.54762(23/42)	0.05556(1/18)	0.00000(0/23)	0.00000(0/19)
Bo_TE_98615	0.09524(4/42)	0.33333(6/18)	0.42857(9/21)	0.85000(17/20)
Bo_TE_36400	0.11364(5/44)	0.76471(13/17)	0.75000(15/20)	0.50000(9/18)
Bo_TE_201671	0.66667(26/39)	0.82353(14/17)	0.00000(0/21)	0.05882(1/17)
Bo_TE_163300	0.67442(29/43)	0.18750(3/16)	1.00000(22/22)	1.00000(20/20)
Bo_TE_115792	0.15909(7/44)	0.50000(9/18)	0.81818(18/22)	0.10526(2/19)
Bo_TE_10864	0.68293(28/41)	0.06250(1/16)	0.47619(10/21)	0.00000(0/20)
Bo_TE_136943	0.04651(2/43)	0.00000(0/19)	0.56522(13/23)	0.30000(6/20)
Bo_TE_215803	0.57143(24/42)	0.44444(8/18)	0.00000(0/21)	0.00000(0/20)
Bo_TE_200771	0.00000(0/43)	0.11765(2/17)	0.08696(2/23)	0.57895(11/19)
Bo_TE_54378	1.00000(40/40)	0.33333(3/9)	0.28571(2/7)	0.47059(8/17)
Bo_TE_7436	0.30233(13/43)	0.55556(10/18)	0.00000(0/21)	0.42105(8/19)
Bo_TE_180284	0.00000(0/46)	0.00000(0/19)	0.85714(18/21)	0.41176(7/17)
Bo_TE_148523	0.97826(45/46)	1.00000(17/17)	1.00000(23/23)	0.50000(9/18)
Bo_TE_101252	0.06522(3/46)	0.14286(2/14)	0.09524(2/21)	0.76471(13/17)
Bo_TE_32936	0.51282(20/39)	0.33333(5/15)	0.00000(0/23)	0.05556(1/18)
Bo_TE_207456	0.84091(37/44)	0.88889(16/18)	0.68421(13/19)	0.30000(6/20)
Bo_TE_53271	0.97674(42/43)	0.83333(15/18)	0.30000(6/20)	1.00000(20/20)
Bo_TE_4533	0.68293(28/41)	0.31250(5/16)	0.00000(0/23)	0.25000(5/20)
Bo_TE_8826	0.00000(0/44)	0.05556(1/18)	0.72727(16/22)	0.05000(1/20)
Bo_TE_17910	0.22222(8/36)	0.11765(2/17)	0.04545(1/22)	0.57895(11/19)
Bo_TE_229308	0.52632(20/38)	0.23529(4/17)	0.95238(20/21)	0.95000(19/20)
Bo_TE_85206	0.47500(19/40)	0.77778(14/18)	0.42105(8/19)	0.10000(2/20)
Bo_TE_145583	0.86047(37/43)	0.38889(7/18)	0.61905(13/21)	0.31579(6/19)
Bo_TE_216178	0.43902(18/41)	0.17647(3/17)	0.10526(2/19)	0.72222(13/18)
Bo_TE_181062	0.69048(29/42)	0.77778(14/18)	0.23810(5/21)	0.85000(17/20)
Bo_TE_170805	0.02174(1/46)	0.38889(7/18)	0.00000(0/22)	0.50000(9/18)
Bo_TE_3397	0.64103(25/39)	0.05263(1/19)	0.00000(0/17)	0.00000(0/17)
Bo_TE_221914	0.15909(7/44)	0.16667(3/18)	0.00000(0/22)	0.60000(12/20)
Bo_TE_96612	0.64103(25/39)	0.05263(1/19)	0.57895(11/19)	0.36842(7/19)
Bo_TE_178043	0.00000(0/45)	0.77778(7/9)	0.85714(18/21)	0.47368(9/19)
Bo_TE_134106	0.80952(34/42)	0.00000(0/17)	0.00000(0/23)	0.05263(1/19)
Bo_TE_197243	1.00000(45/45)	0.88889(16/18)	1.00000(23/23)	0.50000(9/18)
Bo_TE_86739	0.95455(42/44)	0.31250(5/16)	0.61905(13/21)	0.73684(14/19)

Bo_TE_67403	0.68293(28/41)	0.00000(0/17)	0.00000(0/23)	0.00000(0/12)
Bo_TE_123789	0.88889(40/45)	0.16667(3/18)	0.63158(12/19)	0.66667(12/18)
Bo_TE_70615	0.00000(0/45)	0.31250(5/16)	0.68182(15/22)	0.20000(4/20)
Bo_TE_32938	0.43902(18/41)	0.47059(8/17)	1.00000(23/23)	0.94444(17/18)
Bo_TE_117831	0.71429(30/42)	0.17647(3/17)	0.54545(12/22)	0.85000(17/20)
Bo_TE_185479	0.77273(34/44)	0.11111(2/18)	0.42105(8/19)	0.10526(2/19)
Bo_TE_111614	0.68293(28/41)	1.00000(18/18)	0.17391(4/23)	0.82353(14/17)
Bo_TE_214466	0.80488(33/41)	0.88235(15/17)	0.61111(11/18)	0.21053(4/19)
Bo_TE_33179	0.02326(1/43)	0.37500(6/16)	0.33333(7/21)	0.78947(15/19)
Bo_TE_85590	0.45455(20/44)	0.40000(6/15)	1.00000(22/22)	0.94737(18/19)
Bo_TE_237342	0.27907(12/43)	0.92857(13/14)	1.00000(23/23)	0.64706(11/17)
Bo_TE_22414	0.72727(32/44)	0.50000(7/14)	0.04762(1/21)	0.63158(12/19)
Bo_TE_232202	0.68182(30/44)	0.05556(1/18)	0.22727(5/22)	0.42105(8/19)
Bo_TE_66838	0.19048(8/42)	0.44444(8/18)	1.00000(23/23)	1.00000(19/19)
Bo_TE_154880	0.29545(13/44)	0.87500(14/16)	0.23810(5/21)	0.05000(1/20)
Bo_TE_215934	0.02273(1/44)	0.21053(4/19)	0.86364(19/22)	0.05263(1/19)
Bo_TE_65358	0.51282(20/39)	0.63158(12/19)	0.15000(3/20)	0.70000(14/20)
Bo_TE_28457	0.21951(9/41)	0.26316(5/19)	1.00000(22/22)	0.70000(14/20)
Bo_TE_169375	0.97674(42/43)	0.41176(7/17)	0.72727(16/22)	0.31579(6/19)
Bo_TE_181778	0.00000(0/43)	0.00000(0/19)	0.50000(10/20)	0.05000(1/20)
Bo_TE_186120	0.80000(36/45)	0.78947(15/19)	0.20000(4/20)	0.15789(3/19)
Bo_TE_115903	0.15909(7/44)	0.55556(10/18)	0.90000(18/20)	0.15789(3/19)
Bo_TE_161763	0.81395(35/43)	0.22222(4/18)	0.09524(2/21)	0.26316(5/19)
Bo_TE_37592	0.30952(13/42)	0.27778(5/18)	0.95455(21/22)	0.60000(12/20)
Bo_TE_141264	0.80952(34/42)	0.05556(1/18)	0.09524(2/21)	0.21053(4/19)
Bo_TE_41245	0.93182(41/44)	0.29412(5/17)	0.63636(14/22)	0.33333(6/18)
Bo_TE_135886	0.14634(6/41)	0.41176(7/17)	1.00000(22/22)	0.85000(17/20)
Bo_TE_180059	0.58537(24/41)	0.00000(0/16)	0.00000(0/21)	0.00000(0/17)
Bo_TE_94558	0.95238(40/42)	0.17647(3/17)	0.89474(17/19)	0.94737(18/19)
Bo_TE_101764	0.00000(0/45)	0.62500(10/16)	0.04545(1/22)	0.89474(17/19)
Bo_TE_179243	0.38095(16/42)	0.29412(5/17)	0.77273(17/22)	0.05263(1/19)
Bo_TE_48955	0.35000(14/40)	0.72222(13/18)	0.09091(2/22)	0.31579(6/19)
Bo_TE_57167	1.00000(46/46)	1.00000(18/18)	0.76190(16/21)	0.44444(8/18)
Bo_TE_75809	0.50000(20/40)	0.11111(2/18)	0.00000(0/21)	0.05263(1/19)
Bo_TE_35979	0.22857(8/35)	0.29412(5/17)	0.85714(18/21)	1.00000(20/20)
Bo_TE_148565	0.00000(0/44)	0.17647(3/17)	0.00000(0/22)	0.52632(10/19)
Bo_TE_137862	0.84444(38/45)	0.05882(1/17)	0.00000(0/23)	0.00000(0/19)
Bo_TE_200127	0.06977(3/43)	0.88889(16/18)	0.80000(16/20)	0.55556(10/18)
Bo_TE_67613	0.83721(36/43)	0.88235(15/17)	0.15789(3/19)	0.72222(13/18)
Bo_TE_108141	0.00000(0/46)	0.00000(0/18)	0.04762(1/21)	0.50000(9/18)
Bo_TE_96851	0.91111(41/45)	0.17647(3/17)	0.23810(5/21)	0.25000(5/20)
Bo_TE_161001	0.11111(5/45)	0.64706(11/17)	0.29412(5/17)	0.43750(7/16)
Bo_TE_95711	0.34091(15/44)	0.44444(8/18)	1.00000(22/22)	0.15000(3/20)
Bo_TE_18652	0.95556(43/45)	0.43750(7/16)	0.00000(0/18)	0.44444(8/18)
Bo_TE_100529	0.10256(4/39)	0.26667(4/15)	0.61905(13/21)	0.84211(16/19)
Bo_TE_234871	0.85366(35/41)	0.17647(3/17)	0.04348(1/23)	0.05263(1/19)
Bo_TE_146936	0.76923(30/39)	0.43750(7/16)	0.00000(0/22)	0.00000(0/20)
Bo_TE_201737	0.27500(11/40)	0.38889(7/18)	0.68182(15/22)	0.16667(3/18)
Bo_TE_55537	0.00000(0/44)	0.70588(12/17)	0.00000(0/23)	0.22222(4/18)
Bo_TE_195003	0.56410(22/39)	0.27778(5/18)	0.68182(15/22)	0.00000(0/19)
Bo_TE_60835	0.18605(8/43)	0.12500(2/16)	0.90000(18/20)	0.50000(10/20)
Bo_TE_191019	0.83333(35/42)	0.66667(12/18)	0.20000(4/20)	0.30000(6/20)
Bo_TE_63095	0.88372(38/43)	0.31579(6/19)	0.40000(8/20)	0.26316(5/19)
Bo_TE_93529	0.95556(43/45)	0.11111(2/18)	1.00000(21/21)	1.00000(20/20)
Bo_TE_69179	0.26087(12/46)	0.25000(4/16)	0.21739(5/23)	0.88889(16/18)
Bo_TE_117724	0.36842(14/38)	0.61111(11/18)	0.71429(15/21)	0.95000(19/20)

Bo_TE_151825	0.50000(21/42)	0.94444(17/18)	1.00000(22/22)	1.00000(19/19)
Bo_TE_177822	1.00000(43/43)	0.88889(16/18)	1.00000(21/21)	0.50000(10/20)
Bo_TE_76804	0.68293(28/41)	0.11765(2/17)	0.65000(13/20)	0.47368(9/19)
Bo_TE_234878	0.00000(0/46)	0.00000(0/19)	0.00000(0/22)	0.52632(10/19)
Bo_TE_32589	0.93333(42/45)	0.11111(2/18)	0.54545(12/22)	0.85000(17/20)
Bo_TE_28417	0.58974(23/39)	0.35294(6/17)	1.00000(22/22)	0.83333(15/18)
Bo_TE_45821	0.06667(3/45)	0.00000(0/19)	0.00000(0/22)	0.65000(13/20)
Bo_TE_222747	0.07317(3/41)	0.22222(4/18)	0.45455(10/22)	0.75000(15/20)
Bo_TE_25420	0.40476(17/42)	0.62500(10/16)	0.86957(20/23)	1.00000(20/20)
Bo_TE_119185	0.87500(35/40)	0.25000(4/16)	0.43478(10/23)	0.10000(2/20)
Bo_TE_235662	0.21429(9/42)	0.10526(2/19)	0.90909(20/22)	0.50000(9/18)
Bo_TE_184820	0.59524(25/42)	0.56250(9/16)	0.65000(13/20)	0.05000(1/20)
Bo_TE_161853	0.73684(28/38)	0.31250(5/16)	0.81818(18/22)	0.65000(13/20)
Bo_TE_226640	0.82051(32/39)	0.11111(2/18)	0.63158(12/19)	0.10000(2/20)
Bo_TE_36752	1.00000(36/36)	0.70588(12/17)	0.00000(0/22)	0.15000(3/20)
Bo_TE_58729	0.97778(44/45)	1.00000(19/19)	0.27273(6/22)	1.00000(20/20)
Bo_TE_66568	0.32558(14/43)	0.50000(9/18)	0.00000(0/23)	0.00000(0/19)
Bo_TE_158321	0.92857(39/42)	0.82353(14/17)	0.15000(3/20)	0.22222(4/18)
Bo_TE_223245	0.55000(22/40)	0.50000(8/16)	0.00000(0/22)	0.00000(0/20)
Bo_TE_197609	1.00000(42/42)	0.89474(17/19)	1.00000(23/23)	0.44444(8/18)
Bo_TE_11762	0.37500(15/40)	0.56250(9/16)	1.00000(21/21)	1.00000(20/20)
Bo_TE_19948	0.59524(25/42)	0.94118(16/17)	0.65000(13/20)	0.17647(3/17)
Bo_TE_137000	0.89744(35/39)	1.00000(17/17)	0.47368(9/19)	0.94118(16/17)
Bo_TE_108952	0.52273(23/44)	0.00000(0/18)	0.04545(1/22)	0.21053(4/19)
Bo_TE_238494	0.58537(24/41)	0.05556(1/18)	0.00000(0/20)	0.00000(0/17)
Bo_TE_76781	0.56757(21/37)	0.06667(1/15)	0.68182(15/22)	0.00000(0/18)
Bo_TE_136832	0.75000(30/40)	0.12500(2/16)	0.40909(9/22)	0.75000(15/20)
Bo_TE_155861	0.69767(30/43)	0.88235(15/17)	0.55000(11/20)	0.20000(4/20)
Bo_TE_164939	1.00000(42/42)	1.00000(18/18)	0.42857(9/21)	0.27778(5/18)
Bo_TE_8727	0.11364(5/44)	0.94444(17/18)	0.22727(5/22)	0.61111(11/18)
Bo_TE_180130	0.72093(31/43)	0.33333(6/18)	0.41176(7/17)	0.00000(0/20)
Bo_TE_69996	0.71429(30/42)	1.00000(18/18)	0.14286(3/21)	0.60000(12/20)
Bo_TE_154426	0.97778(44/45)	0.11111(2/18)	1.00000(18/18)	1.00000(20/20)
Bo_TE_102583	0.44737(17/38)	0.94118(16/17)	0.40000(8/20)	0.84211(16/19)
Bo_TE_119594	0.41860(18/43)	1.00000(17/17)	0.31818(7/22)	0.66667(12/18)
Bo_TE_150843	0.00000(0/44)	0.26316(5/19)	0.50000(11/22)	0.36842(7/19)
Bo_TE_105965	0.65854(27/41)	0.27778(5/18)	0.00000(0/22)	0.05000(1/20)
Bo_TE_96620	0.67442(29/43)	0.05263(1/19)	0.55000(11/20)	0.33333(6/18)
Bo_TE_125732	0.75000(30/40)	0.31250(5/16)	0.13636(3/22)	0.05263(1/19)
Bo_TE_42424	1.00000(46/46)	0.94118(16/17)	0.72222(13/18)	0.50000(10/20)
Bo_TE_156630	0.00000(0/45)	0.70588(12/17)	0.04762(1/21)	0.15000(3/20)
Bo_TE_230687	0.66667(28/42)	0.81250(13/16)	1.00000(21/21)	0.23529(4/17)
Bo_TE_44455	0.13953(6/43)	0.82353(14/17)	0.04545(1/22)	0.21053(4/19)
Bo_TE_50807	0.02222(1/45)	0.05882(1/17)	0.55000(11/20)	0.27778(5/18)
Bo_TE_117853	0.70270(26/37)	0.22222(4/18)	0.00000(0/22)	0.00000(0/18)
Bo_TE_102908	0.46512(20/43)	0.36842(7/19)	1.00000(21/21)	0.30000(6/20)
Bo_TE_61431	0.51282(20/39)	0.11765(2/17)	0.90000(18/20)	0.85000(17/20)
Bo_TE_147730	0.80000(28/35)	0.31250(5/16)	0.50000(10/20)	0.94444(17/18)
Bo_TE_63139	0.97500(39/40)	0.22222(4/18)	0.35000(7/20)	0.21053(4/19)
Bo_TE_58844	0.31111(14/45)	0.17647(3/17)	0.36364(8/22)	0.70000(14/20)
Bo_TE_89392	0.65116(28/43)	0.35294(6/17)	0.10000(2/20)	0.00000(0/19)
Bo_TE_58620	0.28571(12/42)	0.88235(15/17)	0.40000(8/20)	0.36842(7/19)
Bo_TE_213207	0.33333(14/42)	0.82353(14/17)	1.00000(21/21)	0.83333(15/18)
Bo_TE_68220	0.24324(9/37)	0.81250(13/16)	0.00000(0/21)	0.00000(0/19)
Bo_TE_20241	0.40909(18/44)	0.17647(3/17)	0.86364(19/22)	0.73684(14/19)
Bo_TE_130079	0.29268(12/41)	0.81250(13/16)	0.00000(0/22)	0.10000(2/20)

Bo_TE_81671	0.56098(23/41)	0.68750(11/16)	1.00000(22/22)	0.50000(9/18)
Bo_TE_140953	0.13636(6/44)	0.00000(0/19)	0.09091(2/22)	0.68421(13/19)
Bo_TE_229949	0.02174(1/46)	0.00000(0/18)	0.00000(0/23)	0.70000(14/20)
Bo_TE_53639	0.02222(1/45)	0.00000(0/19)	0.66667(14/21)	0.05556(1/18)
Bo_TE_158275	0.08889(4/45)	0.31250(5/16)	0.85000(17/20)	0.57895(11/19)
Bo_TE_81524	0.07143(3/42)	0.68750(11/16)	0.09091(2/22)	0.10526(2/19)
Bo_TE_75866	0.58537(24/41)	0.50000(9/18)	0.04545(1/22)	0.00000(0/20)
Bo_TE_77994	0.16279(7/43)	0.00000(0/18)	0.66667(14/21)	0.89474(17/19)
Bo_TE_223361	0.41860(18/43)	0.00000(0/17)	0.54545(12/22)	0.05263(1/19)
Bo_TE_36823	0.51282(20/39)	0.05263(1/19)	0.00000(0/23)	0.00000(0/20)
Bo_TE_56068	0.46341(19/41)	0.00000(0/18)	0.80952(17/21)	0.44444(8/18)
Bo_TE_41378	0.00000(0/45)	0.00000(0/18)	0.04348(1/23)	0.75000(15/20)
Bo_TE_207888	0.51163(22/43)	0.18750(3/16)	0.81818(18/22)	0.63158(12/19)
Bo_TE_220824	0.00000(0/44)	0.15789(3/19)	0.28571(6/21)	0.63158(12/19)
Bo_TE_140037	0.69048(29/42)	0.29412(5/17)	0.45000(9/20)	0.00000(0/20)
Bo_TE_181668	0.60976(25/41)	0.40000(6/15)	0.00000(0/20)	0.73684(14/19)
Bo_TE_163574	0.77778(35/45)	1.00000(17/17)	0.81818(18/22)	0.35000(7/20)
Bo_TE_153809	0.59524(25/42)	0.00000(0/19)	0.00000(0/22)	0.05000(1/20)
Bo_TE_65786	0.65000(26/40)	0.66667(10/15)	0.80952(17/21)	0.30000(6/20)
Bo_TE_149306	0.04348(1/23)	0.12500(1/8)	0.81818(18/22)	0.05263(1/19)
Bo_TE_196628	0.00000(0/44)	0.38889(7/18)	0.04545(1/22)	0.78947(15/19)
Bo_TE_83632	0.04545(2/44)	0.22222(4/18)	0.66667(14/21)	0.29412(5/17)
Bo_TE_142427	0.82927(34/41)	0.50000(8/16)	0.63636(14/22)	0.25000(5/20)
Bo_TE_96567	0.00000(0/42)	0.00000(0/18)	0.75000(15/20)	0.25000(5/20)
Bo_TE_117235	0.61364(27/44)	0.16667(3/18)	0.00000(0/23)	0.50000(9/18)
Bo_TE_159683	0.63415(26/41)	0.00000(0/18)	0.05000(1/20)	0.11765(2/17)
Bo_TE_130171	0.79487(31/39)	0.41176(7/17)	0.00000(0/23)	0.05263(1/19)
Bo_TE_135840	0.74419(32/43)	0.20000(3/15)	0.72727(16/22)	0.05263(1/19)
Bo_TE_222001	0.69767(30/43)	0.06250(1/16)	0.30000(6/20)	0.57895(11/19)
Bo_TE_136233	0.69767(30/43)	0.72222(13/18)	0.76190(16/21)	0.20000(4/20)
Bo_TE_90039	0.78571(33/42)	0.17647(3/17)	0.08696(2/23)	0.10000(2/20)
Bo_TE_113852	0.81395(35/43)	0.66667(10/15)	0.00000(0/13)	0.00000(0/17)
Bo_TE_81455	0.34211(13/38)	0.66667(6/9)	0.86364(19/22)	0.10526(2/19)
Bo_TE_67800	0.87805(36/41)	0.57143(8/14)	0.69565(16/23)	0.20000(4/20)
Bo_TE_235559	0.65116(28/43)	0.00000(0/18)	0.00000(0/23)	0.10000(2/20)
Bo_TE_173491	0.60000(27/45)	0.64706(11/17)	0.00000(0/22)	0.00000(0/19)
Bo_TE_46185	0.14634(6/41)	0.50000(9/18)	0.00000(0/23)	0.00000(0/19)
Bo_TE_58021	0.56410(22/39)	0.37500(6/16)	0.47619(10/21)	1.00000(20/20)
Bo_TE_132698	0.71053(27/38)	0.83333(15/18)	1.00000(22/22)	0.40000(8/20)
Bo_TE_226194	0.33333(14/42)	0.83333(15/18)	1.00000(23/23)	1.00000(20/20)
Bo_TE_41178	0.00000(0/43)	0.00000(0/19)	0.22727(5/22)	0.52941(9/17)
Bo_TE_119582	0.33333(14/42)	1.00000(17/17)	0.31818(7/22)	0.63158(12/19)
Bo_TE_150418	0.95238(40/42)	0.86667(13/15)	0.75000(15/20)	0.31250(5/16)
Bo_TE_48628	0.97727(43/44)	1.00000(19/19)	0.38095(8/21)	0.38889(7/18)
Bo_TE_59122	0.00000(0/44)	0.57143(8/14)	0.44444(8/18)	0.81250(13/16)
Bo_TE_179172	0.76190(32/42)	0.40000(6/15)	0.14286(3/21)	0.77778(14/18)
Bo_TE_36623	0.24390(10/41)	0.31250(5/16)	1.00000(16/16)	0.94444(17/18)
Bo_TE_122069	0.53488(23/43)	0.58824(10/17)	1.00000(22/22)	0.27778(5/18)
Bo_TE_210346	0.11628(5/43)	0.38889(7/18)	1.00000(23/23)	0.95000(19/20)
Bo_TE_157772	0.00000(0/46)	0.00000(0/19)	0.36842(7/19)	0.70000(14/20)
Bo_TE_226624	0.73171(30/41)	0.50000(9/18)	0.26087(6/23)	0.16667(3/18)
Bo_TE_203179	0.71795(28/39)	0.82353(14/17)	0.50000(11/22)	0.05000(1/20)
Bo_TE_235132	0.00000(0/46)	0.00000(0/19)	0.71429(15/21)	0.00000(0/20)
Bo_TE_124898	0.00000(0/45)	0.00000(0/18)	0.00000(0/23)	0.50000(9/18)
Bo_TE_169419	0.09302(4/43)	0.88889(16/18)	0.86364(19/22)	0.78947(15/19)
Bo_TE_37247	0.15909(7/44)	0.58824(10/17)	0.00000(0/22)	0.05000(1/20)

Bo_TE_179168	0.77778(35/45)	0.40000(6/15)	0.14286(3/21)	0.73684(14/19)
Bo_TE_78609	0.84091(37/44)	0.15789(3/19)	0.10000(2/20)	0.05263(1/19)
Bo_TE_114070	0.59524(25/42)	0.17647(3/17)	0.00000(0/23)	0.05000(1/20)
Bo_TE_163803	0.64444(29/45)	0.15789(3/19)	0.00000(0/23)	0.05556(1/18)
Bo_TE_96725	0.04651(2/43)	0.23529(4/17)	0.57895(11/19)	0.00000(0/20)
Bo_TE_37600	0.33333(13/39)	0.27778(5/18)	1.00000(22/22)	0.63158(12/19)
Bo_TE_221627	0.25000(11/44)	0.00000(0/18)	0.52381(11/21)	0.89474(17/19)
Bo_TE_136385	0.86047(37/43)	0.11765(2/17)	0.38095(8/21)	0.16667(3/18)
Bo_TE_26448	0.17073(7/41)	0.62500(10/16)	0.84211(16/19)	0.89474(17/19)
Bo_TE_198153	0.33333(14/42)	0.52941(9/17)	0.19048(4/21)	1.00000(20/20)
Bo_TE_204390	0.13636(6/44)	0.26316(5/19)	1.00000(21/21)	0.75000(15/20)
Bo_TE_140342	0.26829(11/41)	0.21053(4/19)	0.61905(13/21)	0.00000(0/18)
Bo_TE_48620	0.93478(43/46)	1.00000(19/19)	0.45000(9/20)	0.35294(6/17)
Bo_TE_92178	0.84444(38/45)	0.73333(11/15)	1.00000(21/21)	0.31579(6/19)
Bo_TE_91963	0.16667(7/42)	0.00000(0/18)	0.08696(2/23)	0.57895(11/19)
Bo_TE_111896	0.70732(29/41)	0.12500(2/16)	0.77273(17/22)	0.13333(2/15)
Bo_TE_191225	0.00000(0/45)	0.61111(11/18)	0.00000(0/21)	0.00000(0/20)
Bo_TE_37143	0.72727(32/44)	0.50000(9/18)	1.00000(22/22)	0.95000(19/20)
Bo_TE_92290	0.02500(1/40)	0.06667(1/15)	0.00000(0/21)	0.52941(9/17)
Bo_TE_235125	0.70455(31/44)	0.27778(5/18)	0.19048(4/21)	0.31250(5/16)
Bo_TE_101160	0.57500(23/40)	0.31250(5/16)	0.09524(2/21)	0.61111(11/18)
Bo_TE_109888	0.37209(16/43)	0.22222(4/18)	0.42857(9/21)	1.00000(20/20)
Bo_TE_133500	0.88095(37/42)	0.76471(13/17)	1.00000(23/23)	0.47368(9/19)
Bo_TE_137238	0.83721(36/43)	0.64706(11/17)	0.60000(12/20)	0.00000(0/16)
Bo_TE_224473	0.00000(0/45)	0.16667(3/18)	0.22727(5/22)	0.73684(14/19)
Bo_TE_97663	0.28571(12/42)	0.76471(13/17)	1.00000(23/23)	0.75000(15/20)
Bo_TE_222131	0.00000(0/41)	0.00000(0/17)	0.00000(0/21)	0.70588(12/17)
Bo_TE_55828	0.95122(39/41)	0.86667(13/15)	0.21053(4/19)	0.83333(15/18)
Bo_TE_67138	0.95556(43/45)	0.00000(0/15)	0.18182(4/22)	0.50000(9/18)
Bo_TE_142939	0.00000(0/45)	0.13333(2/15)	0.60000(12/20)	0.29412(5/17)
Bo_TE_5206	0.15789(6/38)	0.64706(11/17)	0.71429(15/21)	0.95000(19/20)
Bo_TE_191570	0.78049(32/41)	0.00000(0/18)	0.00000(0/22)	0.00000(0/20)
Bo_TE_172377	0.33333(14/42)	1.00000(18/18)	0.14286(3/21)	0.25000(5/20)
Bo_TE_96637	0.20000(9/45)	0.61111(11/18)	0.80952(17/21)	0.77778(14/18)
Bo_TE_136479	0.86364(38/44)	0.05556(1/18)	0.68182(15/22)	0.68421(13/19)
Bo_TE_128519	0.27907(12/43)	0.68421(13/19)	0.57143(12/21)	0.00000(0/20)
Bo_TE_103643	0.05000(2/40)	0.60000(9/15)	0.57143(12/21)	0.77778(7/9)
Bo_TE_14794	0.10256(4/39)	0.05556(1/18)	0.10526(2/19)	0.65000(13/20)
Bo_TE_10539	0.02174(1/46)	0.16667(3/18)	0.57143(12/21)	0.00000(0/20)
Bo_TE_39205	0.84615(33/39)	0.12500(2/16)	1.00000(23/23)	1.00000(20/20)
Bo_TE_91640	0.93333(42/45)	0.76471(13/17)	0.38095(8/21)	0.85000(17/20)
Bo_TE_11493	0.62500(25/40)	0.53333(8/15)	0.00000(0/22)	0.15789(3/19)
Bo_TE_4961	0.53333(24/45)	0.27778(5/18)	1.00000(19/19)	0.89474(17/19)
Bo_TE_131261	1.00000(41/41)	1.00000(16/16)	0.95238(20/21)	0.42105(8/19)
Bo_TE_170145	0.26190(11/42)	0.64706(11/17)	1.00000(22/22)	0.57895(11/19)
Bo_TE_25003	0.00000(0/46)	0.05556(1/18)	0.70000(14/20)	0.05000(1/20)
Bo_TE_97427	0.35714(15/42)	0.41176(7/17)	0.95652(22/23)	0.50000(10/20)
Bo_TE_100964	0.00000(0/46)	0.35294(6/17)	0.63636(14/22)	0.50000(9/18)
Bo_TE_18316	0.48780(20/41)	0.25000(4/16)	0.81818(18/22)	0.05000(1/20)
Bo_TE_33628	0.57143(24/42)	1.00000(17/17)	0.70000(14/20)	0.23529(4/17)
Bo_TE_209678	0.36842(14/38)	0.16667(3/18)	0.30000(6/20)	0.75000(15/20)
Bo_TE_216873	0.54054(20/37)	0.00000(0/18)	0.15789(3/19)	0.00000(0/19)
Bo_TE_137015	0.72500(29/40)	0.11765(2/17)	0.00000(0/23)	0.25000(5/20)
Bo_TE_223436	1.00000(45/45)	0.82353(14/17)	0.26316(5/19)	0.47368(9/19)
Bo_TE_58435	0.09302(4/43)	0.00000(0/19)	0.57143(12/21)	0.00000(0/20)
Bo_TE_30111	0.50000(22/44)	0.70588(12/17)	0.33333(7/21)	0.94444(17/18)

Bo_TE_86320	0.65789(25/38)	0.62500(10/16)	0.13043(3/23)	0.31579(6/19)
Bo_TE_180786	1.00000(41/41)	0.50000(9/18)	1.00000(10/10)	0.62500(5/8)
Bo_TE_138447	0.81395(35/43)	0.94737(18/19)	0.80000(16/20)	0.26316(5/19)
Bo_TE_214448	0.68889(31/45)	0.06250(1/16)	0.00000(0/23)	0.05263(1/19)
Bo_TE_83540	0.41860(18/43)	0.58824(10/17)	0.60000(12/20)	1.00000(20/20)
Bo_TE_153911	1.00000(44/44)	1.00000(18/18)	0.57143(12/21)	0.47368(9/19)
Bo_TE_184857	0.53659(22/41)	0.00000(0/16)	0.00000(0/23)	0.00000(0/19)
Bo_TE_59065	0.86364(38/44)	0.29412(5/17)	0.40909(9/22)	0.52632(10/19)
Bo_TE_234202	0.02381(1/42)	0.50000(9/18)	0.00000(0/22)	0.05263(1/19)
Bo_TE_91208	0.76190(32/42)	0.05882(1/17)	0.00000(0/23)	0.22222(4/18)
Bo_TE_67790	0.90698(39/43)	0.66667(10/15)	0.72222(13/18)	0.21053(4/19)
Bo_TE_91065	0.58140(25/43)	0.17647(3/17)	0.72727(16/22)	0.84211(16/19)
Bo_TE_152171	0.31707(13/41)	0.68750(11/16)	0.86364(19/22)	0.88889(16/18)
Bo_TE_201352	0.53659(22/41)	0.33333(6/18)	0.71429(15/21)	0.95000(19/20)
Bo_TE_8223	0.79070(34/43)	0.17647(3/17)	0.00000(0/22)	0.00000(0/19)
Bo_TE_194955	0.31707(13/41)	0.82353(14/17)	0.50000(9/18)	0.61111(11/18)
Bo_TE_235465	0.39535(17/43)	0.64706(11/17)	0.00000(0/23)	0.00000(0/20)
Bo_TE_144849	0.83721(36/43)	0.11765(2/17)	0.00000(0/20)	0.61111(11/18)
Bo_TE_121563	0.20000(8/40)	0.83333(15/18)	0.70000(14/20)	0.73684(14/19)
Bo_TE_144928	0.11111(5/45)	0.11765(2/17)	0.00000(0/22)	0.50000(9/18)
Bo_TE_90103	0.50000(22/44)	0.70588(12/17)	0.90476(19/21)	1.00000(20/20)
Bo_TE_194511	0.10000(4/40)	0.00000(0/5)	0.81818(18/22)	0.27778(5/18)
Bo_TE_221986	0.69767(30/43)	0.05882(1/17)	0.31579(6/19)	0.57895(11/19)
Bo_TE_224691	0.36585(15/41)	0.28571(2/7)	0.85714(18/21)	1.00000(19/19)
Bo_TE_95548	0.83784(31/37)	0.29412(5/17)	0.47059(8/17)	0.68421(13/19)
Bo_TE_31608	0.71795(28/39)	0.00000(0/19)	0.00000(0/22)	0.15000(3/20)
Bo_TE_79105	0.00000(0/45)	0.05882(1/17)	0.63158(12/19)	0.60000(12/20)
Bo_TE_101118	0.02222(1/45)	0.07692(1/13)	0.78947(15/19)	0.22222(4/18)
Bo_TE_140801	0.00000(0/46)	0.00000(0/19)	0.36842(7/19)	0.52632(10/19)
Bo_TE_172580	0.00000(0/45)	0.05263(1/19)	0.47619(10/21)	0.57895(11/19)
Bo_TE_51424	0.36364(16/44)	0.88889(16/18)	1.00000(22/22)	1.00000(20/20)
Bo_TE_148559	0.00000(0/44)	0.17647(3/17)	0.00000(0/23)	0.66667(12/18)
Bo_TE_77033	0.04444(2/45)	0.33333(5/15)	0.22727(5/22)	0.73684(14/19)
Bo_TE_189039	0.70732(29/41)	0.28571(4/14)	0.04545(1/22)	0.00000(0/20)
Bo_TE_105593	0.60000(24/40)	0.83333(15/18)	0.60000(12/20)	0.15000(3/20)
Bo_TE_193865	0.51220(21/41)	0.35294(6/17)	0.17391(4/23)	0.85000(17/20)
Bo_TE_204371	0.68421(26/38)	0.11111(2/18)	0.69565(16/23)	1.00000(20/20)
Bo_TE_108497	0.02174(1/46)	0.64706(11/17)	0.18182(4/22)	0.00000(0/20)
Bo_TE_105929	0.67500(27/40)	0.23529(4/17)	0.00000(0/22)	0.05263(1/19)
Bo_TE_17795	0.50000(21/42)	0.50000(8/16)	0.00000(0/22)	0.00000(0/20)
Bo_TE_156886	0.31111(14/45)	0.00000(0/17)	0.68182(15/22)	0.15000(3/20)
Bo_TE_36023	0.06667(3/45)	0.70588(12/17)	0.47826(11/23)	0.50000(10/20)
Bo_TE_230067	0.15000(6/40)	0.83333(15/18)	0.57143(12/21)	0.80000(16/20)
Bo_TE_37137	0.73810(31/42)	0.47059(8/17)	1.00000(22/22)	0.94737(18/19)
Bo_TE_33985	0.26829(11/41)	0.37500(6/16)	0.18182(4/22)	0.83333(15/18)
Bo_TE_3861	0.18182(8/44)	0.70588(12/17)	0.25000(5/20)	0.30000(6/20)
Bo_TE_114474	0.86047(37/43)	0.58824(10/17)	0.76190(16/21)	0.10000(2/20)
Bo_TE_108427	0.79545(35/44)	0.37500(6/16)	1.00000(22/22)	1.00000(19/19)
Bo_TE_25553	0.70732(29/41)	0.18750(3/16)	0.42857(9/21)	0.47368(9/19)
Bo_TE_119509	0.17500(7/40)	0.00000(0/17)	0.63636(14/22)	0.15000(3/20)
Bo_TE_111878	0.65909(29/44)	0.00000(0/17)	0.76190(16/21)	0.05556(1/18)
Bo_TE_125310	0.79545(35/44)	0.27778(5/18)	0.04545(1/22)	0.68421(13/19)
Bo_TE_28133	0.20930(9/43)	0.88889(16/18)	0.19048(4/21)	0.40000(8/20)
Bo_TE_125962	0.84091(37/44)	0.56250(9/16)	0.00000(0/23)	0.05000(1/20)
Bo_TE_41618	0.76316(29/38)	0.66667(12/18)	0.28571(6/21)	0.22222(4/18)
Bo_TE_38890	0.00000(0/43)	0.11111(2/18)	0.18182(4/22)	0.57895(11/19)

Bo_TE_80384	0.87805(36/41)	0.70588(12/17)	0.54545(12/22)	0.15789(3/19)
Bo_TE_190667	0.88636(39/44)	0.41176(7/17)	0.05000(1/20)	0.20000(4/20)
Bo_TE_81547	0.06667(3/45)	0.62500(10/16)	0.04545(1/22)	0.00000(0/19)
Bo_TE_207004	0.09091(4/44)	0.00000(0/18)	0.50000(10/20)	0.55556(10/18)
Bo_TE_236084	0.00000(0/45)	0.42105(8/19)	0.52632(10/19)	0.00000(0/19)
Bo_TE_202129	0.45238(19/42)	0.94444(17/18)	1.00000(20/20)	0.94737(18/19)
Bo_TE_178196	0.82222(37/45)	1.00000(19/19)	0.38095(8/21)	1.00000(20/20)
Bo_TE_95851	0.52500(21/40)	0.38889(7/18)	0.00000(0/22)	0.00000(0/20)
Bo_TE_134091	0.11364(5/44)	0.64706(11/17)	0.91304(21/23)	0.47059(8/17)
Bo_TE_137049	0.65909(29/44)	0.83333(15/18)	0.27778(5/18)	0.33333(6/18)
Bo_TE_204705	0.57500(23/40)	0.82353(14/17)	0.00000(0/22)	0.52632(10/19)
Bo_TE_37957	0.81818(36/44)	0.11111(2/18)	0.50000(10/20)	0.10526(2/19)
Bo_TE_218899	0.26316(10/38)	0.83333(15/18)	0.14286(3/21)	0.15789(3/19)
Bo_TE_148272	0.50000(21/42)	0.29412(5/17)	0.20000(4/20)	0.00000(0/20)
Bo_TE_103301	0.97727(43/44)	0.83333(15/18)	0.68182(15/22)	0.33333(6/18)
Bo_TE_95566	0.75000(30/40)	0.56250(9/16)	0.04762(1/21)	0.41176(7/17)
Bo_TE_134508	0.45000(18/40)	0.94118(16/17)	1.00000(22/22)	0.78947(15/19)
Bo_TE_222089	0.00000(0/45)	0.81250(13/16)	0.00000(0/23)	0.00000(0/20)
Bo_TE_101536	0.87500(35/40)	0.25000(4/16)	0.47619(10/21)	0.05556(1/18)
Bo_TE_92971	0.02174(1/46)	0.00000(0/19)	0.80952(17/21)	0.88889(16/18)
Bo_TE_203183	0.73684(28/38)	0.78947(15/19)	0.50000(11/22)	0.05263(1/19)
Bo_TE_3833	0.80488(33/41)	0.17647(3/17)	0.57143(12/21)	0.00000(0/20)
Bo_TE_120840	0.41860(18/43)	0.33333(6/18)	0.63636(14/22)	0.00000(0/20)
Bo_TE_212503	0.31707(13/41)	0.66667(12/18)	0.77273(17/22)	0.16667(3/18)
Bo_TE_221466	0.00000(0/44)	0.00000(0/17)	0.00000(0/22)	0.50000(10/20)
Bo_TE_32133	0.92308(36/39)	0.16667(3/18)	0.90476(19/21)	0.95000(19/20)
Bo_TE_115717	0.04762(2/42)	0.11111(2/18)	0.09524(2/21)	0.84211(16/19)
Bo_TE_177434	0.55263(21/38)	0.23529(4/17)	0.86957(20/23)	0.95000(19/20)
Bo_TE_143280	0.51163(22/43)	0.64706(11/17)	0.95652(22/23)	0.11111(2/18)
Bo_TE_55674	1.00000(43/43)	0.33333(6/18)	1.00000(22/22)	1.00000(20/20)
Bo_TE_227177	0.69767(30/43)	0.60000(9/15)	0.95455(21/22)	0.15789(3/19)
Bo_TE_32173	0.04545(2/44)	0.77778(14/18)	0.08696(2/23)	0.05000(1/20)
Bo_TE_58161	0.11905(5/42)	0.94444(17/18)	0.52381(11/21)	0.37500(6/16)
Bo_TE_20465	0.30769(12/39)	0.81250(13/16)	0.72727(16/22)	0.72222(13/18)
Bo_TE_141873	0.97674(42/43)	0.82353(14/17)	0.22727(5/22)	0.84211(16/19)
Bo_TE_67782	0.86364(38/44)	0.66667(10/15)	0.82609(19/23)	0.21053(4/19)
Bo_TE_10163	0.60976(25/41)	0.41176(7/17)	0.38095(8/21)	0.00000(0/20)
Bo_TE_92304	0.25000(11/44)	0.50000(9/18)	0.00000(0/22)	0.00000(0/20)
Bo_TE_51924	0.00000(0/44)	0.00000(0/17)	0.70000(14/20)	0.00000(0/20)
Bo_TE_95631	0.00000(0/38)	0.76471(13/17)	0.47619(10/21)	0.63158(12/19)
Bo_TE_53660	0.00000(0/45)	0.52941(9/17)	0.04762(1/21)	0.11111(2/18)
Bo_TE_103930	0.12195(5/41)	0.70588(12/17)	0.19048(4/21)	0.23529(4/17)
Bo_TE_124031	0.72093(31/43)	0.94118(16/17)	0.30000(6/20)	0.15789(3/19)
Bo_TE_5529	0.78049(32/41)	0.23529(4/17)	0.19048(4/21)	0.15000(3/20)
Bo_TE_97225	0.41860(18/43)	0.75000(12/16)	1.00000(23/23)	0.75000(15/20)
Bo_TE_87860	0.04444(2/45)	0.05263(1/19)	0.54545(12/22)	0.00000(0/20)
Bo_TE_3360	0.27273(12/44)	0.11111(2/18)	0.90909(20/22)	0.15789(3/19)
Bo_TE_87166	0.00000(0/45)	0.00000(0/18)	0.83333(15/18)	0.85000(17/20)
Bo_TE_100732	0.06667(3/45)	0.05556(1/18)	0.09091(2/22)	0.75000(15/20)
Bo_TE_103728	0.93182(41/44)	0.72222(13/18)	0.33333(7/21)	0.43750(7/16)
Bo_TE_211683	0.23810(10/42)	0.56250(9/16)	1.00000(20/20)	0.78947(15/19)
Bo_TE_177279	0.86047(37/43)	0.00000(0/19)	0.08696(2/23)	0.85000(17/20)
Bo_TE_191592	0.60976(25/41)	0.00000(0/18)	0.66667(14/21)	0.26316(5/19)
Bo_TE_80471	0.06977(3/43)	0.22222(4/18)	0.50000(10/20)	0.85000(17/20)
Bo_TE_11438	0.48837(21/43)	0.26667(4/15)	1.00000(21/21)	1.00000(17/17)
Bo_TE_55342	0.84091(37/44)	0.47059(8/17)	0.14286(3/21)	0.21429(3/14)

Bo_TE_209451	0.50000(21/42)	1.00000(16/16)	0.81818(18/22)	0.90000(18/20)
Bo_TE_197495	0.02222(1/45)	0.10526(2/19)	0.17391(4/23)	0.63158(12/19)
Bo_TE_240762	0.84615(33/39)	0.17647(3/17)	0.00000(0/22)	0.05263(1/19)
Bo_TE_149054	0.21429(9/42)	0.42105(8/19)	1.00000(22/22)	1.00000(20/20)
Bo_TE_98528	0.15385(6/39)	0.29412(5/17)	0.36842(7/19)	0.80000(16/20)
Bo_TE_238481	0.60976(25/41)	0.06250(1/16)	0.00000(0/21)	0.00000(0/18)
Bo_TE_22879	0.11111(5/45)	0.55556(10/18)	0.00000(0/21)	0.05000(1/20)
Bo_TE_95527	0.88636(39/44)	0.35294(6/17)	0.90476(19/21)	0.84211(16/19)
Bo_TE_231077	0.04545(2/44)	0.00000(0/17)	0.23810(5/21)	0.50000(10/20)
Bo_TE_21679	0.02222(1/45)	0.11111(2/18)	0.47368(9/19)	0.65000(13/20)
Bo_TE_191824	0.76744(33/43)	0.37500(6/16)	0.00000(0/21)	0.00000(0/20)
Bo_TE_224310	1.00000(44/44)	0.42105(8/19)	1.00000(23/23)	1.00000(19/19)
Bo_TE_234573	0.09091(4/44)	0.33333(6/18)	0.36364(8/22)	0.63158(12/19)
Bo_TE_62444	0.00000(0/46)	0.00000(0/18)	0.00000(0/21)	0.50000(10/20)
Bo_TE_120978	0.66667(28/42)	0.66667(12/18)	0.25000(5/20)	0.10526(2/19)
Bo_TE_185082	0.00000(0/46)	0.00000(0/18)	0.38095(8/21)	0.50000(9/18)
Bo_TE_116588	0.83721(36/43)	0.50000(8/16)	0.33333(7/21)	0.55000(11/20)
Bo_TE_101971	0.55814(24/43)	0.18750(3/16)	0.00000(0/23)	0.52632(10/19)
Bo_TE_179205	0.69231(27/39)	0.41176(7/17)	0.66667(14/21)	0.05000(1/20)
Bo_TE_98869	0.97778(44/45)	0.89474(17/19)	0.43478(10/23)	0.65000(13/20)
Bo_TE_129823	0.31818(14/44)	0.05556(1/18)	0.80952(17/21)	0.00000(0/20)
Bo_TE_141429	0.83721(36/43)	0.05882(1/17)	0.04545(1/22)	0.25000(5/20)
Bo_TE_221301	0.00000(0/46)	0.82353(14/17)	0.00000(0/19)	0.05000(1/20)
Bo_TE_119188	0.13953(6/43)	0.76471(13/17)	0.50000(10/20)	0.90000(18/20)
Bo_TE_11583	0.36364(16/44)	0.25000(4/16)	0.00000(0/22)	0.60000(12/20)
Bo_TE_119748	0.65909(29/44)	0.00000(0/18)	0.00000(0/23)	0.00000(0/19)
Bo_TE_9697	0.39535(17/43)	0.77778(14/18)	0.00000(0/22)	0.22222(4/18)
Bo_TE_225080	0.16279(7/43)	0.52632(10/19)	0.80000(16/20)	0.00000(0/20)
Bo_TE_121863	0.46341(19/41)	1.00000(18/18)	0.66667(12/18)	0.50000(5/10)
Bo_TE_190655	0.88889(40/45)	0.11765(2/17)	0.75000(15/20)	0.10526(2/19)
Bo_TE_149292	0.50000(21/42)	0.64706(11/17)	0.00000(0/21)	0.00000(0/19)
Bo_TE_174305	0.90698(39/43)	0.94444(17/18)	0.82609(19/23)	0.16667(3/18)
Bo_TE_41451	0.79545(35/44)	0.00000(0/18)	0.85714(18/21)	0.12500(2/16)
Bo_TE_50779	0.63415(26/41)	0.64706(11/17)	0.80952(17/21)	0.17647(3/17)
Bo_TE_48989	0.00000(0/43)	0.00000(0/18)	0.55000(11/20)	0.36842(7/19)
Bo_TE_39733	0.56098(23/41)	0.05882(1/17)	0.00000(0/23)	0.00000(0/20)
Bo_TE_4576	0.06977(3/43)	0.50000(8/16)	0.00000(0/21)	0.05263(1/19)
Bo_TE_169347	0.93478(43/46)	0.52941(9/17)	0.61905(13/21)	0.33333(6/18)
Bo_TE_158363	0.65116(28/43)	0.75000(12/16)	0.17391(4/23)	0.23529(4/17)
Bo_TE_56741	0.16667(7/42)	0.42857(6/14)	0.30000(6/20)	0.72222(13/18)
Bo_TE_173008	0.37778(17/45)	0.52941(9/17)	0.00000(0/21)	0.66667(12/18)
Bo_TE_216629	0.00000(0/45)	0.05263(1/19)	0.55000(11/20)	0.30000(6/20)
Bo_TE_179945	0.16279(7/43)	0.38889(7/18)	1.00000(21/21)	0.21053(4/19)
Bo_TE_76275	0.61905(26/42)	0.00000(0/16)	0.66667(14/21)	0.38889(7/18)
Bo_TE_87249	0.64286(27/42)	0.76471(13/17)	0.04762(1/21)	0.10000(2/20)
Bo_TE_221154	0.56818(25/44)	0.50000(8/16)	1.00000(22/22)	0.94737(18/19)
Bo_TE_114445	0.64444(29/45)	0.42105(8/19)	0.95455(21/22)	0.55000(11/20)
Bo_TE_77292	0.17500(7/40)	0.77778(14/18)	1.00000(19/19)	0.88235(15/17)
Bo_TE_81416	0.75000(30/40)	0.94444(17/18)	0.89474(17/19)	0.33333(6/18)
Bo_TE_127531	0.83333(35/42)	0.33333(6/18)	0.14286(3/21)	0.00000(0/20)
Bo_TE_119637	0.00000(0/44)	0.72222(13/18)	0.45455(10/22)	0.20000(4/20)
Bo_TE_86021	0.58140(25/43)	0.94737(18/19)	1.00000(22/22)	0.11111(2/18)
Bo_TE_96615	0.64286(27/42)	0.05263(1/19)	0.52381(11/21)	0.33333(6/18)
Bo_TE_113787	0.38462(15/39)	0.00000(0/17)	0.33333(7/21)	0.80000(16/20)
Bo_TE_25237	0.00000(0/42)	0.05263(1/19)	0.55000(11/20)	0.00000(0/20)
Bo_TE_119533	0.18605(8/43)	0.00000(0/18)	0.61905(13/21)	0.11111(2/18)

Bo_TE_184443	0.22727(10/44)	0.52941(9/17)	0.75000(15/20)	0.60000(12/20)
Bo_TE_72767	0.20930(9/43)	0.66667(12/18)	0.66667(14/21)	0.05556(1/18)
Bo_TE_78937	0.86364(38/44)	0.70588(12/17)	0.36364(8/22)	0.05556(1/18)
Bo_TE_198521	0.11364(5/44)	0.29412(5/17)	0.35000(7/20)	0.94737(18/19)
Bo_TE_184520	0.06452(2/31)	0.42857(6/14)	0.75000(15/20)	0.00000(0/18)
Bo_TE_172595	0.00000(0/45)	0.38462(5/13)	0.04545(1/22)	0.61111(11/18)
Bo_TE_229871	0.04545(2/44)	0.50000(8/16)	0.38095(8/21)	0.00000(0/20)
Bo_TE_170819	0.53488(23/43)	0.68421(13/19)	0.00000(0/22)	0.05263(1/19)
Bo_TE_215822	0.86047(37/43)	0.94737(18/19)	1.00000(21/21)	0.38889(7/18)
Bo_TE_210390	0.25641(10/39)	0.40000(6/15)	0.95455(21/22)	0.73684(14/19)
Bo_TE_17032	0.86364(38/44)	0.00000(0/17)	0.00000(0/22)	0.10526(2/19)
Bo_TE_151745	0.42857(18/42)	0.82353(14/17)	0.65000(13/20)	0.94737(18/19)
Bo_TE_96193	0.35000(14/40)	0.83333(15/18)	0.61905(13/21)	0.05556(1/18)
Bo_TE_122735	0.09302(4/43)	0.00000(0/18)	0.13043(3/23)	0.66667(12/18)
Bo_TE_178078	0.02222(1/45)	0.00000(0/19)	0.00000(0/23)	0.52632(10/19)
Bo_TE_124185	0.76744(33/43)	0.26316(5/19)	0.22222(4/18)	0.15000(3/20)
Bo_TE_22984	0.90244(37/41)	0.94118(16/17)	0.36842(7/19)	0.40000(8/20)
Bo_TE_200836	0.79487(31/39)	0.23529(4/17)	0.14286(3/21)	0.22222(4/18)
Bo_TE_37277	0.00000(0/45)	0.00000(0/19)	0.57143(12/21)	0.00000(0/20)
Bo_TE_122839	0.32609(15/46)	0.83333(15/18)	0.28571(6/21)	0.05263(1/19)
Bo_TE_235051	0.30952(13/42)	0.38889(7/18)	0.61905(13/21)	0.85000(17/20)
Bo_TE_145326	0.25641(10/39)	0.00000(0/17)	0.42857(9/21)	0.63158(12/19)
Bo_TE_41305	0.39024(16/41)	0.06250(1/16)	0.76190(16/21)	0.44444(8/18)
Bo_TE_97974	0.97561(40/41)	0.93750(15/16)	0.47059(8/17)	0.44444(8/18)
Bo_TE_184811	0.58537(24/41)	0.52941(9/17)	0.70000(14/20)	0.05000(1/20)
Bo_TE_195712	0.68182(30/44)	0.88889(16/18)	0.57143(12/21)	0.35000(7/20)
Bo_TE_121163	0.00000(0/44)	0.00000(0/19)	0.17391(4/23)	0.78947(15/19)
Bo_TE_91320	0.00000(0/45)	0.20000(3/15)	0.63158(12/19)	0.61111(11/18)
Bo_TE_16672	0.04762(2/42)	0.29412(5/17)	0.70588(12/17)	0.57895(11/19)
Bo_TE_36450	0.06818(3/44)	0.62500(10/16)	0.59091(13/22)	0.57895(11/19)
Bo_TE_75693	1.00000(46/46)	0.33333(6/18)	0.95238(20/21)	0.85000(17/20)
Bo_TE_170918	0.34091(15/44)	0.11765(2/17)	0.47619(10/21)	0.85000(17/20)
Bo_TE_238352	0.06818(3/44)	0.77778(14/18)	0.09091(2/22)	0.00000(0/20)
Bo_TE_48132	0.35714(15/42)	0.78947(15/19)	0.00000(0/22)	0.00000(0/20)
Bo_TE_1416	0.17778(8/45)	0.87500(14/16)	0.45455(10/22)	0.70000(14/20)
Bo_TE_219360	0.56098(23/41)	0.00000(0/17)	0.21053(4/19)	0.21053(4/19)
Bo_TE_24664	0.06818(3/44)	0.58824(10/17)	0.04348(1/23)	0.10000(2/20)
Bo_TE_117836	0.02222(1/45)	0.52632(10/19)	0.38095(8/21)	0.15000(3/20)
Bo_TE_128311	0.36585(15/41)	0.88235(15/17)	1.00000(22/22)	0.94444(17/18)
Bo_TE_211782	0.73171(30/41)	0.15789(3/19)	0.00000(0/23)	0.00000(0/20)
Bo_TE_216072	0.09091(4/44)	0.05263(1/19)	0.76190(16/21)	0.52632(10/19)
Bo_TE_204581	0.31707(13/41)	0.00000(0/19)	0.40000(8/20)	0.55000(11/20)
Bo_TE_89609	0.02222(1/45)	0.00000(0/19)	0.50000(11/22)	0.00000(0/20)
Bo_TE_135490	0.50000(21/42)	0.10526(2/19)	0.30000(6/20)	0.80000(16/20)
Bo_TE_197992	0.97674(42/43)	0.56250(9/16)	0.19048(4/21)	0.38889(7/18)
Bo_TE_70222	0.77500(31/40)	0.50000(9/18)	0.80952(17/21)	0.16667(3/18)
Bo_TE_46589	0.97674(42/43)	0.88235(15/17)	0.14286(3/21)	0.95000(19/20)
Bo_TE_119825	0.02381(1/42)	0.00000(0/17)	0.04348(1/23)	0.57895(11/19)
Bo_TE_220801	0.00000(0/44)	0.16667(3/18)	0.20000(4/20)	0.63158(12/19)
Bo_TE_226662	0.79070(34/43)	0.50000(8/16)	0.61905(13/21)	0.15000(3/20)
Bo_TE_180379	0.02273(1/44)	0.05556(1/18)	0.60000(12/20)	0.52632(10/19)
Bo_TE_24412	0.16279(7/43)	0.88235(15/17)	0.90909(20/22)	0.95000(19/20)
Bo_TE_130584	0.88636(39/44)	0.33333(6/18)	0.09091(2/22)	0.10526(2/19)
Bo_TE_216291	0.00000(0/46)	0.05556(1/18)	0.45455(10/22)	0.57895(11/19)
Bo_TE_12540	0.18605(8/43)	0.89474(17/19)	0.47368(9/19)	0.90000(18/20)
Bo_TE_163569	0.83333(35/42)	1.00000(16/16)	0.80000(16/20)	0.36842(7/19)

Bo_TE_197603	0.02273(1/44)	0.11111(2/18)	0.00000(0/23)	0.57895(11/19)
Bo_TE_7122	0.09302(4/43)	0.26667(4/15)	0.72727(16/22)	0.73684(14/19)
Bo_TE_184772	0.00000(0/45)	0.00000(0/17)	0.09524(2/21)	0.55000(11/20)
Bo_TE_186111	0.73684(28/38)	0.70588(12/17)	0.21053(4/19)	0.15789(3/19)
Bo_TE_85789	0.00000(0/43)	0.33333(6/18)	0.13636(3/22)	0.50000(8/16)
Bo_TE_115146	0.39024(16/41)	0.23529(4/17)	0.86364(19/22)	0.77778(14/18)
Bo_TE_186158	0.22727(10/44)	0.73333(11/15)	0.45455(10/22)	0.89474(17/19)
Bo_TE_35049	0.53333(24/45)	0.64706(11/17)	0.13636(3/22)	0.10000(2/20)
Bo_TE_169606	0.00000(0/45)	0.55556(10/18)	0.18182(4/22)	0.00000(0/19)
Bo_TE_202657	0.97619(41/42)	0.61111(11/18)	0.77273(17/22)	0.31579(6/19)
Bo_TE_237147	0.26829(11/41)	0.20000(3/15)	1.00000(22/22)	1.00000(18/18)
Bo_TE_241961	0.77273(34/44)	0.10526(2/19)	0.40000(8/20)	0.10000(2/20)
Bo_TE_145627	1.00000(46/46)	0.50000(9/18)	1.00000(23/23)	1.00000(20/20)
Bo_TE_201455	0.00000(0/45)	0.00000(0/19)	0.00000(0/23)	0.57895(11/19)
Bo_TE_230263	0.72500(29/40)	0.37500(6/16)	0.13636(3/22)	0.50000(9/18)
Bo_TE_87172	1.00000(45/45)	0.94118(16/17)	0.19048(4/21)	0.15789(3/19)
Bo_TE_234319	0.02174(1/46)	0.47368(9/19)	0.68182(15/22)	0.68421(13/19)
Bo_TE_171828	0.72500(29/40)	0.27778(5/18)	0.80000(12/15)	0.05263(1/19)
Bo_TE_119212	0.66667(28/42)	0.29412(5/17)	0.23810(5/21)	0.05263(1/19)
Bo_TE_151477	0.11111(5/45)	0.00000(0/18)	0.13636(3/22)	0.52632(10/19)
Bo_TE_128382	0.59524(25/42)	0.05882(1/17)	0.00000(0/21)	0.05263(1/19)
Bo_TE_141935	1.00000(44/44)	0.73333(11/15)	0.27273(6/22)	0.60000(12/20)
Bo_TE_168559	0.33333(14/42)	0.94118(16/17)	0.28571(6/21)	0.20000(4/20)
Bo_TE_188783	0.25581(11/43)	0.66667(12/18)	0.00000(0/21)	0.00000(0/19)
Bo_TE_43613	0.81081(30/37)	0.55556(10/18)	0.00000(0/22)	0.36842(7/19)
Bo_TE_86594	0.69767(30/43)	0.05556(1/18)	0.00000(0/21)	0.00000(0/20)
Bo_TE_157530	0.00000(0/42)	0.00000(0/19)	0.45000(9/20)	0.75000(15/20)
Bo_TE_202666	0.92683(38/41)	0.61111(11/18)	0.80000(16/20)	0.31579(6/19)
Bo_TE_239354	0.24324(9/37)	0.71429(10/14)	0.86364(19/22)	0.52632(10/19)
Bo_TE_211466	0.64286(27/42)	0.23529(4/17)	0.00000(0/20)	0.55556(10/18)
Bo_TE_62776	0.02381(1/42)	0.63158(12/19)	0.50000(10/20)	0.00000(0/20)
Bo_TE_234818	0.88889(40/45)	0.21053(4/19)	0.00000(0/23)	0.10526(2/19)
Bo_TE_108204	0.07692(3/39)	0.00000(0/17)	0.21053(4/19)	0.66667(12/18)
Bo_TE_15482	0.77273(34/44)	0.06250(1/16)	0.00000(0/22)	0.52632(10/19)
Bo_TE_178108	0.02174(1/46)	0.00000(0/17)	0.00000(0/18)	0.68421(13/19)
Bo_TE_162546	0.15556(7/45)	0.05556(1/18)	0.36842(7/19)	0.84211(16/19)
Bo_TE_54500	0.00000(0/44)	0.10000(1/10)	0.61905(13/21)	0.00000(0/9)
Bo_TE_115647	0.28889(13/45)	0.05882(1/17)	0.04348(1/23)	0.72222(13/18)
Bo_TE_55739	0.93182(41/44)	1.00000(18/18)	0.90909(20/22)	0.21053(4/19)
Bo_TE_235300	0.04545(2/44)	0.10526(2/19)	0.04348(1/23)	0.73684(14/19)
Bo_TE_1055	0.73810(31/42)	0.35294(6/17)	0.66667(12/18)	0.10526(2/19)
Bo_TE_134660	0.13333(6/45)	0.62500(10/16)	0.09091(2/22)	0.05000(1/20)
Bo_TE_180202	0.06667(3/45)	0.00000(0/17)	0.73684(14/19)	0.25000(5/20)
Bo_TE_90065	0.51111(23/45)	0.00000(0/19)	0.13636(3/22)	0.00000(0/20)
Bo_TE_231483	0.50000(20/40)	0.27778(5/18)	0.00000(0/23)	0.45000(9/20)
Bo_TE_131186	0.57143(24/42)	0.11111(2/18)	0.04348(1/23)	0.05263(1/19)
Bo_TE_80455	0.93023(40/43)	0.78947(15/19)	0.54545(12/22)	0.15789(3/19)
Bo_TE_111249	0.82500(33/40)	0.58824(10/17)	0.00000(0/19)	0.20000(4/20)
Bo_TE_55014	0.90909(40/44)	0.66667(12/18)	0.30435(7/23)	0.95000(19/20)
Bo_TE_101267	0.97561(40/41)	0.93333(14/15)	0.57143(12/21)	0.40000(8/20)
Bo_TE_85937	0.97778(44/45)	0.47059(8/17)	0.47826(11/23)	0.85000(17/20)
Bo_TE_189041	0.28571(12/42)	0.64286(9/14)	0.95455(21/22)	1.00000(20/20)
Bo_TE_32627	0.02439(1/41)	0.88235(15/17)	0.45000(9/20)	0.15000(3/20)
Bo_TE_137510	0.31579(12/38)	0.37500(6/16)	0.09524(2/21)	0.80000(16/20)
Bo_TE_115750	0.85714(36/42)	0.43750(7/16)	0.17391(4/23)	0.81250(13/16)
Bo_TE_124460	0.86667(39/45)	0.05882(1/17)	0.38095(8/21)	0.00000(0/17)

Bo_TE_183884	0.86047(37/43)	0.31250(5/16)	0.76190(16/21)	0.68421(13/19)
Bo_TE_20070	0.77273(34/44)	0.12500(2/16)	0.47619(10/21)	0.10526(2/19)
Bo_TE_234210	0.62222(28/45)	0.22222(4/18)	0.00000(0/23)	0.00000(0/20)
Bo_TE_165322	0.55814(24/43)	0.00000(0/18)	0.14286(3/21)	0.10000(2/20)
Bo_TE_84575	0.65854(27/41)	0.11765(2/17)	0.66667(14/21)	0.94444(17/18)
Bo_TE_4148	0.75556(34/45)	0.00000(0/18)	0.00000(0/23)	0.00000(0/20)
Bo_TE_151432	0.11111(5/45)	0.87500(14/16)	0.09091(2/22)	0.30000(6/20)
Bo_TE_230931	0.17073(7/41)	0.26667(4/15)	0.42857(9/21)	0.82353(14/17)
Bo_TE_45804	0.60976(25/41)	0.10526(2/19)	0.00000(0/21)	0.15000(3/20)
Bo_TE_105974	0.70455(31/44)	0.77778(14/18)	0.66667(12/18)	0.25000(5/20)
Bo_TE_48969	0.40476(17/42)	0.18750(3/16)	0.90000(18/20)	0.76471(13/17)
Bo_TE_162592	0.34884(15/43)	0.76471(13/17)	0.60000(12/20)	1.00000(20/20)
Bo_TE_106285	0.47500(19/40)	0.68421(13/19)	1.00000(23/23)	0.95000(19/20)
Bo_TE_36932	0.00000(0/44)	0.52941(9/17)	0.30000(6/20)	0.00000(0/20)
Bo_TE_130816	0.60465(26/43)	0.00000(0/16)	0.18182(4/22)	0.21053(4/19)
Bo_TE_8701	0.17778(8/45)	0.68750(11/16)	0.40909(9/22)	0.05263(1/19)
Bo_TE_222762	0.04762(2/42)	0.00000(0/18)	0.52381(11/21)	0.75000(15/20)
Bo_TE_59761	0.82927(34/41)	0.33333(5/15)	0.77273(17/22)	0.20000(4/20)
Bo_TE_10246	0.20000(8/40)	0.06250(1/16)	0.86667(13/15)	0.73684(14/19)
Bo_TE_40620	0.00000(0/45)	0.00000(0/18)	0.63158(12/19)	0.10000(2/20)
Bo_TE_86530	0.00000(0/45)	0.12500(2/16)	0.04545(1/22)	0.58824(10/17)
Bo_TE_56292	0.67442(29/43)	0.47059(8/17)	0.25000(5/20)	0.00000(0/20)
Bo_TE_141631	0.12821(5/39)	0.92308(12/13)	0.88889(16/18)	0.77778(14/18)
Bo_TE_7693	0.81818(36/44)	1.00000(14/14)	0.13636(3/22)	0.00000(0/18)
Bo_TE_28075	0.77273(34/44)	0.11765(2/17)	0.45455(10/22)	0.10000(2/20)
Bo_TE_123927	0.02222(1/45)	0.17647(3/17)	0.15789(3/19)	0.78947(15/19)
Bo_TE_145606	0.06977(3/43)	0.58824(10/17)	0.54545(12/22)	0.68421(13/19)
Bo_TE_209951	0.29268(12/41)	0.38889(7/18)	0.71429(15/21)	1.00000(20/20)
Bo_TE_15435	0.70732(29/41)	0.06250(1/16)	0.04545(1/22)	0.55556(10/18)
Bo_TE_142937	0.00000(0/44)	0.18750(3/16)	0.59091(13/22)	0.26316(5/19)
Bo_TE_2050	0.04444(2/45)	0.38889(7/18)	0.72727(16/22)	0.45000(9/20)
Bo_TE_161234	0.42222(19/45)	0.73684(14/19)	1.00000(23/23)	0.63158(12/19)
Bo_TE_125392	0.00000(0/43)	0.50000(9/18)	0.45455(10/22)	0.25000(5/20)
Bo_TE_208110	0.85000(34/40)	0.27778(5/18)	1.00000(23/23)	1.00000(20/20)
Bo_TE_5499	0.21429(9/42)	0.82353(14/17)	0.80952(17/21)	0.85000(17/20)
Bo_TE_136008	0.60465(26/43)	0.57895(11/19)	0.00000(0/21)	0.00000(0/2)
Bo_TE_209959	0.27500(11/40)	0.26316(5/19)	0.47619(10/21)	0.84211(16/19)
Bo_TE_188373	0.69767(30/43)	0.82353(14/17)	0.31818(7/22)	0.33333(6/18)
Bo_TE_104823	0.76744(33/43)	0.85714(12/14)	0.14286(3/21)	0.20000(4/20)
Bo_TE_194876	0.34211(13/38)	0.29412(5/17)	0.90476(19/21)	0.84211(16/19)
Bo_TE_2786	0.80952(34/42)	0.53333(8/15)	0.00000(0/23)	0.00000(0/20)
Bo_TE_41173	0.88889(40/45)	0.29412(5/17)	0.95238(20/21)	0.30000(6/20)
Bo_TE_182226	0.18182(8/44)	0.70588(12/17)	0.00000(0/21)	0.30000(6/20)
Bo_TE_8001	0.95349(41/43)	1.00000(17/17)	0.44444(8/18)	0.42105(8/19)
Bo_TE_33903	0.00000(0/45)	0.00000(0/18)	0.71429(15/21)	0.00000(0/20)
Bo_TE_149558	0.97826(45/46)	0.11111(2/18)	0.85000(17/20)	0.85000(17/20)
Bo_TE_100042	0.02273(1/44)	0.00000(0/18)	0.65000(13/20)	0.84211(16/19)
Bo_TE_117857	0.79545(35/44)	0.76471(13/17)	0.45000(9/20)	0.00000(0/16)
Bo_TE_20934	0.00000(0/45)	0.00000(0/18)	0.65000(13/20)	0.26316(5/19)
Bo_TE_92824	0.32558(14/43)	0.06250(1/16)	0.18182(4/22)	0.64706(11/17)
Bo_TE_186123	0.17073(7/41)	0.17647(3/17)	0.80952(17/21)	0.21053(4/19)
Bo_TE_220487	0.57143(20/35)	0.88889(16/18)	0.52381(11/21)	0.20000(4/20)
Bo_TE_224582	0.20513(8/39)	0.81250(13/16)	0.11111(2/18)	0.41176(7/17)
Bo_TE_210224	0.06522(3/46)	0.00000(0/19)	0.00000(0/22)	0.52632(10/19)
Bo_TE_150847	0.00000(0/45)	0.05556(1/18)	0.19048(4/21)	0.55556(10/18)
Bo_TE_184859	0.58537(24/41)	0.00000(0/17)	0.00000(0/20)	0.00000(0/20)

Bo_TE_184380	0.76744(33/43)	0.00000(0/11)	0.13636(3/22)	0.22222(4/18)
Bo_TE_54533	0.53659(22/41)	0.43750(7/16)	0.00000(0/23)	0.00000(0/19)
Bo_TE_92434	0.04651(2/43)	0.10526(2/19)	0.75000(15/20)	0.25000(5/20)
Bo_TE_92283	0.56410(22/39)	0.41176(7/17)	0.04545(1/22)	0.15789(3/19)
Bo_TE_179244	0.45238(19/42)	0.55556(10/18)	0.00000(0/21)	0.11765(2/17)
Bo_TE_90386	0.04545(2/44)	0.00000(0/18)	0.66667(14/21)	0.89474(17/19)
Bo_TE_198349	0.52381(22/42)	0.05882(1/17)	0.00000(0/20)	0.00000(0/20)
Bo_TE_86555	0.77273(34/44)	0.50000(8/16)	0.23810(5/21)	0.61111(11/18)
Bo_TE_23134	0.00000(0/43)	0.82353(14/17)	0.47619(10/21)	0.80000(16/20)
Bo_TE_112364	0.68421(26/38)	0.22222(4/18)	0.04348(1/23)	0.05556(1/18)
Bo_TE_173310	0.46341(19/41)	0.76471(13/17)	0.90476(19/21)	0.35000(7/20)
Bo_TE_15373	0.66667(30/45)	0.11765(2/17)	0.00000(0/21)	0.61111(11/18)
Bo_TE_177993	0.00000(0/45)	0.68750(11/16)	0.00000(0/23)	0.00000(0/20)
Bo_TE_165286	0.58140(25/43)	0.00000(0/17)	0.15000(3/20)	0.10000(2/20)
Bo_TE_40723	0.54545(24/44)	0.12500(2/16)	0.75000(15/20)	0.25000(5/20)
Bo_TE_27140	0.08889(4/45)	0.00000(0/17)	0.76190(16/21)	0.52632(10/19)
Bo_TE_31020	0.61538(24/39)	0.47059(8/17)	0.33333(7/21)	0.94444(17/18)
Bo_TE_135725	0.37500(15/40)	0.21053(4/19)	0.73913(17/23)	0.31579(6/19)
Bo_TE_41844	0.04444(2/45)	0.55556(10/18)	0.27273(6/22)	0.57895(11/19)
Bo_TE_70034	0.61905(26/42)	0.93750(15/16)	0.19048(4/21)	0.65000(13/20)
Bo_TE_85601	0.02174(1/46)	0.52941(9/17)	0.04348(1/23)	0.00000(0/20)
Bo_TE_37658	0.37500(15/40)	0.17647(3/17)	0.95455(21/22)	0.65000(13/20)
Bo_TE_139241	0.60465(26/43)	0.23529(4/17)	0.40000(8/20)	0.90000(18/20)
Bo_TE_169458	0.35000(14/40)	0.73684(14/19)	0.95455(21/22)	0.47368(9/19)
Bo_TE_83711	0.23810(10/42)	0.05556(1/18)	0.73684(14/19)	0.33333(6/18)
Bo_TE_101069	0.78049(32/41)	0.38462(5/13)	0.40909(9/22)	0.05263(1/19)
Bo_TE_129037	0.04762(2/42)	0.29412(5/17)	0.00000(0/22)	0.52632(10/19)
Bo_TE_75665	1.00000(46/46)	1.00000(6/6)	1.00000(21/21)	0.42105(8/19)
Bo_TE_49016	0.00000(0/44)	0.00000(0/18)	0.57143(12/21)	0.22222(4/18)
Bo_TE_197619	1.00000(42/42)	0.25000(4/16)	0.91304(21/23)	1.00000(10/10)
Bo_TE_182877	0.69231(27/39)	0.44444(8/18)	1.00000(23/23)	0.95000(19/20)
Bo_TE_82489	0.06818(3/44)	0.73684(14/19)	0.05000(1/20)	0.30000(6/20)
Bo_TE_115423	0.51163(22/43)	0.94118(16/17)	0.86364(19/22)	0.30000(6/20)
Bo_TE_226230	0.36842(14/38)	0.82353(14/17)	0.00000(0/23)	0.05000(1/20)
Bo_TE_129759	0.26316(10/38)	0.87500(14/16)	0.81818(18/22)	0.50000(9/18)
Bo_TE_230843	0.15909(7/44)	0.11111(2/18)	1.00000(22/22)	1.00000(20/20)
Bo_TE_104072	0.50000(20/40)	0.72222(13/18)	0.36364(8/22)	0.20000(4/20)
Bo_TE_237202	0.80952(34/42)	0.05556(1/18)	0.08696(2/23)	0.00000(0/19)
Bo_TE_223967	0.11364(5/44)	0.68750(11/16)	0.23810(5/21)	0.00000(0/20)
Bo_TE_89383	0.33333(15/45)	0.58824(10/17)	0.14286(3/21)	1.00000(19/19)
Bo_TE_16871	0.83333(35/42)	0.83333(15/18)	0.00000(0/23)	0.05000(1/20)
Bo_TE_200037	0.88889(40/45)	0.31579(6/19)	0.23810(5/21)	0.15789(3/19)
Bo_TE_13883	0.53846(21/39)	0.40000(6/15)	0.00000(0/22)	0.00000(0/20)
Bo_TE_170727	0.05405(2/37)	0.12500(2/16)	0.57895(11/19)	0.00000(0/16)
Bo_TE_227308	0.64706(22/34)	1.00000(18/18)	0.26316(5/19)	0.50000(10/20)
Bo_TE_119602	0.62791(27/43)	0.00000(0/17)	0.71429(15/21)	0.36842(7/19)
Bo_TE_235757	0.93478(43/46)	0.77778(14/18)	0.95652(22/23)	0.35000(7/20)
Bo_TE_33697	0.02273(1/44)	0.12500(2/16)	0.86957(20/23)	0.85000(17/20)
Bo_TE_121661	0.51220(21/41)	0.16667(3/18)	0.36364(8/22)	0.84211(16/19)
Bo_TE_26687	0.06250(2/32)	0.62500(10/16)	0.00000(0/23)	0.00000(0/19)
Bo_TE_31216	0.65854(27/41)	0.05882(1/17)	0.76190(16/21)	0.31579(6/19)
Bo_TE_196342	0.15909(7/44)	0.58824(10/17)	0.00000(0/23)	0.42105(8/19)
Bo_TE_75640	1.00000(44/44)	1.00000(17/17)	0.90909(20/22)	0.47059(8/17)
Bo_TE_185522	0.84615(33/39)	0.11765(2/17)	0.45000(9/20)	0.12500(2/16)
Bo_TE_169145	0.17073(7/41)	0.70588(12/17)	1.00000(23/23)	0.94737(18/19)
Bo_TE_163531	0.59524(25/42)	0.41176(7/17)	0.90909(20/22)	0.21053(4/19)

Bo_TE_227078	0.04348(2/46)	0.62500(10/16)	0.80952(17/21)	0.20000(4/20)
Bo_TE_129263	0.00000(0/44)	0.72222(13/18)	0.00000(0/7)	0.00000(0/20)
Bo_TE_146001	0.00000(0/46)	0.64706(11/17)	0.28571(6/21)	0.00000(0/19)
Bo_TE_95712	0.95556(43/45)	1.00000(19/19)	1.00000(23/23)	0.29412(5/17)
Bo_TE_200243	0.00000(0/46)	0.00000(0/18)	0.21739(5/23)	0.70588(12/17)
Bo_TE_104601	0.39024(16/41)	0.11765(2/17)	0.09091(2/22)	0.80000(16/20)
Bo_TE_38891	0.95455(42/44)	0.17647(3/17)	0.65000(13/20)	0.38889(7/18)
Bo_TE_126801	0.85714(36/42)	0.18750(3/16)	0.04348(1/23)	0.31579(6/19)
Bo_TE_65865	1.00000(46/46)	0.94444(17/18)	0.95652(22/23)	0.50000(10/20)
Bo_TE_138732	0.06522(3/46)	0.50000(9/18)	0.00000(0/23)	0.05000(1/20)
Bo_TE_196709	0.78571(33/42)	0.27778(5/18)	0.04762(1/21)	0.15789(3/19)
Bo_TE_168267	0.48889(22/45)	0.37500(6/16)	1.00000(22/22)	0.89474(17/19)
Bo_TE_141568	0.20000(9/45)	0.94118(16/17)	0.95455(21/22)	0.78947(15/19)
Bo_TE_8774	0.86047(37/43)	0.83333(15/18)	0.22727(5/22)	0.88889(16/18)
Bo_TE_117280	0.02273(1/44)	0.82353(14/17)	0.66667(14/21)	0.50000(9/18)
Bo_TE_193858	0.39535(17/43)	0.61111(11/18)	0.86364(19/22)	0.15000(3/20)
Bo_TE_46549	0.34146(14/41)	0.62500(10/16)	0.00000(0/19)	0.65000(13/20)
Bo_TE_52675	0.20930(9/43)	0.05556(1/18)	0.89474(17/19)	0.05263(1/19)
Bo_TE_210573	0.00000(0/45)	0.00000(0/18)	0.59091(13/22)	0.68421(13/19)
Bo_TE_67623	0.17500(7/40)	0.11765(2/17)	0.76190(16/21)	0.20000(4/20)
Bo_TE_224079	0.50000(20/40)	0.00000(0/19)	0.19048(4/21)	0.00000(0/19)
Bo_TE_36641	0.63158(24/38)	0.76471(13/17)	0.00000(0/21)	0.05556(1/18)
Bo_TE_101592	0.93182(41/44)	0.41176(7/17)	0.27273(6/22)	0.70588(12/17)
Bo_TE_204835	0.54348(25/46)	0.35294(6/17)	0.04545(1/22)	0.63158(12/19)
Bo_TE_111709	0.34211(13/38)	0.06667(1/15)	0.76190(16/21)	0.05556(1/18)
Bo_TE_21099	0.20000(9/45)	0.88889(16/18)	0.55000(11/20)	0.89474(17/19)
Bo_TE_234755	0.07317(3/41)	0.33333(6/18)	0.00000(0/22)	0.55000(11/20)
Bo_TE_231036	0.06818(3/44)	0.05556(1/18)	0.57143(12/21)	0.16667(3/18)
Bo_TE_158407	0.80488(33/41)	0.43750(7/16)	1.00000(23/23)	0.94444(17/18)
Bo_TE_165145	0.91111(41/45)	0.37500(6/16)	0.78947(15/19)	1.00000(20/20)
Bo_TE_203602	0.00000(0/43)	0.00000(0/19)	0.13636(3/22)	0.75000(15/20)
Bo_TE_2602	0.23684(9/38)	0.14286(2/14)	1.00000(18/18)	0.87500(14/16)
Bo_TE_159825	0.70000(28/40)	0.05556(1/18)	0.00000(0/22)	0.00000(0/18)
Bo_TE_179541	0.81395(35/43)	0.81250(13/16)	0.00000(0/23)	0.50000(7/14)
Bo_TE_43534	0.23810(10/42)	0.35294(6/17)	0.95000(19/20)	0.47368(9/19)
Bo_TE_216619	0.18605(8/43)	0.93750(15/16)	0.00000(0/10)	0.00000(0/14)
Bo_TE_60924	0.33333(13/39)	0.37500(6/16)	0.95000(19/20)	0.82353(14/17)
Bo_TE_156077	0.53488(23/43)	0.15789(3/19)	1.00000(18/18)	0.84211(16/19)
Bo_TE_201503	0.41026(16/39)	0.88235(15/17)	1.00000(22/22)	1.00000(20/20)
Bo_TE_46755	0.74419(32/43)	0.50000(8/16)	0.15789(3/19)	0.21053(4/19)
Bo_TE_114837	0.18182(8/44)	0.88889(16/18)	0.52381(11/21)	0.90000(18/20)
Bo_TE_141422	0.82222(37/45)	0.11765(2/17)	0.00000(0/18)	0.21053(4/19)
Bo_TE_70272	0.07895(3/38)	0.47059(8/17)	0.09091(2/22)	0.84211(16/19)
Bo_TE_169251	0.20513(8/39)	0.46667(7/15)	0.75000(15/20)	0.42105(8/19)
Bo_TE_119194	0.87805(36/41)	0.21429(3/14)	0.45455(10/22)	0.10000(2/20)
Bo_TE_57276	1.00000(44/44)	1.00000(19/19)	0.77273(17/22)	0.45000(9/20)
Bo_TE_203089	0.04651(2/43)	0.11111(2/18)	0.13636(3/22)	0.75000(15/20)
Bo_TE_198006	0.02222(1/45)	0.43750(7/16)	0.54545(12/22)	0.75000(15/20)
Bo_TE_108577	0.27907(12/43)	0.61111(11/18)	0.36364(8/22)	0.00000(0/20)
Bo_TE_80831	0.55882(19/34)	0.44444(8/18)	0.95652(22/23)	0.15789(3/19)
Bo_TE_194871	0.09302(4/43)	0.00000(0/18)	0.73913(17/23)	0.26316(5/19)
Bo_TE_165653	0.77778(35/45)	0.06250(1/16)	0.08696(2/23)	0.10526(2/19)
Bo_TE_143880	0.13953(6/43)	0.15789(3/19)	0.65000(13/20)	0.80000(16/20)
Bo_TE_48383	0.60465(26/43)	0.44444(8/18)	0.00000(0/22)	0.25000(5/20)
Bo_TE_30530	0.22222(10/45)	0.05556(1/18)	0.77273(17/22)	0.15789(3/19)
Bo_TE_193791	0.42500(17/40)	0.76471(13/17)	0.82609(19/23)	0.15000(3/20)

Bo_TE_64219	0.17073(7/41)	0.64706(11/17)	0.54545(12/22)	0.05556(1/18)
Bo_TE_27067	0.95349(41/43)	0.94118(16/17)	0.15000(3/20)	0.58824(10/17)
Bo_TE_27172	0.90909(40/44)	0.94118(16/17)	0.13636(3/22)	0.20000(4/20)
Bo_TE_95173	0.08889(4/45)	0.68750(11/16)	0.04545(1/22)	0.38889(7/18)
Bo_TE_169750	0.77778(35/45)	0.11765(2/17)	0.00000(0/23)	0.05000(1/20)
Bo_TE_85773	0.00000(0/46)	0.33333(6/18)	0.14286(3/21)	0.52632(10/19)
Bo_TE_183837	0.00000(0/43)	0.00000(0/19)	0.80952(17/21)	0.44444(8/18)
Bo_TE_178264	0.20000(9/45)	0.82353(14/17)	1.00000(21/21)	1.00000(19/19)
Bo_TE_184692	0.84444(38/45)	0.31250(5/16)	0.00000(0/23)	0.15000(3/20)
Bo_TE_153868	0.13636(6/44)	0.56250(9/16)	0.00000(0/22)	0.05000(1/20)
Bo_TE_174532	0.25000(10/40)	0.05882(1/17)	0.00000(0/22)	0.76471(13/17)
Bo_TE_69162	0.26667(12/45)	0.22222(4/18)	0.21739(5/23)	0.88889(16/18)
Bo_TE_96590	0.65116(28/43)	0.05263(1/19)	0.55000(11/20)	0.36842(7/19)
Bo_TE_210884	0.42857(18/42)	0.83333(15/18)	0.25000(5/20)	0.10000(2/20)
Bo_TE_178098	0.33333(14/42)	0.64706(11/17)	1.00000(21/21)	0.26316(5/19)
Bo_TE_109533	0.00000(0/44)	0.56250(9/16)	0.05556(1/18)	0.07143(1/14)
Bo_TE_222391	0.02222(1/45)	0.05556(1/18)	0.18182(4/22)	0.78947(15/19)
Bo_TE_48994	0.86364(38/44)	0.17647(3/17)	0.14286(3/21)	0.30000(6/20)
Bo_TE_199156	0.62162(23/37)	0.50000(8/16)	0.09091(2/22)	0.05263(1/19)
Bo_TE_111048	0.65116(28/43)	0.25000(4/16)	1.00000(23/23)	0.65000(13/20)
Bo_TE_87093	0.00000(0/43)	0.00000(0/18)	0.78947(15/19)	0.84211(16/19)
Bo_TE_224681	0.60976(25/41)	0.16667(3/18)	0.00000(0/21)	0.05263(1/19)
Bo_TE_46348	0.00000(0/38)	0.62500(10/16)	0.85714(18/21)	0.53333(8/15)
Bo_TE_132425	0.45238(19/42)	0.00000(0/19)	0.71429(15/21)	0.05000(1/20)
Bo_TE_112070	0.27273(12/44)	0.00000(0/18)	0.65000(13/20)	0.38889(7/18)
Bo_TE_185129	0.81395(35/43)	0.80000(12/15)	0.40909(9/22)	0.05263(1/19)
Bo_TE_135980	0.00000(0/46)	0.00000(0/19)	0.13636(3/22)	0.63158(12/19)
Bo_TE_130160	0.70455(31/44)	0.60000(9/15)	0.04348(1/23)	0.11111(2/18)
Bo_TE_232038	0.00000(0/39)	0.00000(0/18)	0.30000(6/20)	0.52941(9/17)
Bo_TE_156937	0.54545(24/44)	0.88235(15/17)	0.25000(5/20)	0.68421(13/19)
Bo_TE_33541	0.08696(4/46)	0.64706(11/17)	0.19048(4/21)	0.73684(14/19)
Bo_TE_89652	0.00000(0/46)	0.52632(10/19)	0.00000(0/23)	0.00000(0/20)
Bo_TE_165232	0.52273(23/44)	0.00000(0/17)	0.15000(3/20)	0.05263(1/19)
Bo_TE_236879	0.41860(18/43)	0.11765(2/17)	0.72727(16/22)	0.89474(17/19)
Bo_TE_41882	0.75610(31/41)	0.50000(8/16)	0.05000(1/20)	0.16667(3/18)
Bo_TE_76438	0.51163(22/43)	0.00000(0/18)	0.77273(17/22)	0.52632(10/19)
Bo_TE_123766	0.11364(5/44)	0.88235(15/17)	0.38095(8/21)	0.31579(6/19)
Bo_TE_89913	0.04878(2/41)	0.70588(12/17)	0.55000(11/20)	0.84211(16/19)
Bo_TE_197648	0.16667(7/42)	0.68750(11/16)	0.13636(3/22)	0.77778(14/18)
Bo_TE_90122	0.69231(27/39)	0.87500(14/16)	0.35714(5/14)	0.20000(4/20)
Bo_TE_101113	1.00000(45/45)	0.88889(16/18)	0.43478(10/23)	0.95000(19/20)
Bo_TE_235064	0.65854(27/41)	0.58824(10/17)	0.42857(9/21)	0.15789(3/19)
Bo_TE_128628	0.73810(31/42)	0.26316(5/19)	0.42857(9/21)	1.00000(19/19)
Bo_TE_85318	0.00000(0/43)	0.00000(0/18)	0.60000(12/20)	0.89474(17/19)
Bo_TE_234977	0.46667(21/45)	0.76471(13/17)	1.00000(23/23)	0.72222(13/18)
Bo_TE_183958	0.77778(35/45)	0.47059(8/17)	0.00000(0/23)	0.05556(1/18)
Bo_TE_136403	0.13953(6/43)	0.88235(15/17)	0.63636(14/22)	0.78947(15/19)
Bo_TE_123225	0.17778(8/45)	0.52941(9/17)	0.84211(16/19)	0.44444(8/18)
Bo_TE_91113	0.75610(31/41)	0.00000(0/19)	0.00000(0/22)	0.00000(0/20)
Bo_TE_67037	0.77273(34/44)	0.58824(10/17)	0.00000(0/21)	0.00000(0/20)
Bo_TE_11679	0.41463(17/41)	0.64286(9/14)	0.95652(22/23)	0.57895(11/19)
Bo_TE_139140	0.45714(16/35)	0.88235(15/17)	0.23810(5/21)	0.89474(17/19)
Bo_TE_108418	0.20000(8/40)	0.64706(11/17)	0.00000(0/22)	0.00000(0/19)
Bo_TE_227178	0.90476(38/42)	0.64706(11/17)	0.95455(21/22)	0.21053(4/19)
Bo_TE_222652	0.00000(0/44)	0.26316(5/19)	0.45455(10/22)	0.57895(11/19)
Bo_TE_216716	0.68293(28/41)	0.94444(17/18)	0.50000(11/22)	0.15000(3/20)

Bo_TE_39568	0.06977(3/43)	0.17647(3/17)	0.59091(13/22)	0.55556(10/18)
Bo_TE_21650	0.00000(0/46)	0.00000(0/14)	0.04545(1/22)	0.63158(12/19)
Bo_TE_238418	0.25000(10/40)	0.66667(12/18)	1.00000(22/22)	1.00000(18/18)
Bo_TE_159044	0.97826(45/46)	1.00000(17/17)	0.66667(14/21)	0.42105(8/19)
Bo_TE_163249	0.04651(2/43)	0.06250(1/16)	0.61905(13/21)	0.78947(15/19)
Bo_TE_197650	0.13953(6/43)	0.73684(14/19)	0.14286(3/21)	0.83333(15/18)
Bo_TE_37242	0.18605(8/43)	0.62500(10/16)	0.00000(0/22)	0.05000(1/20)
Bo_TE_204338	0.27273(12/44)	0.77778(14/18)	0.28571(6/21)	0.17647(3/17)
Bo_TE_103649	0.00000(0/46)	0.50000(9/18)	0.00000(0/23)	0.00000(0/20)
Bo_TE_17532	0.52273(23/44)	0.05556(1/18)	0.09091(2/22)	0.00000(0/20)
Bo_TE_81708	0.22727(10/44)	0.76471(13/17)	0.22727(5/22)	0.26316(5/19)
Bo_TE_48314	0.86047(37/43)	0.22222(4/18)	0.54545(12/22)	0.33333(6/18)
Bo_TE_54740	0.55000(22/40)	0.22222(4/18)	0.78947(15/19)	0.55556(10/18)
Bo_TE_199906	0.51351(19/37)	0.31579(6/19)	0.75000(15/20)	0.15789(3/19)
Bo_TE_76127	0.00000(0/46)	0.36842(7/19)	0.85714(18/21)	0.40000(8/20)
Bo_TE_204555	0.28889(13/45)	0.29412(5/17)	0.00000(0/22)	0.75000(15/20)
Bo_TE_70301	0.07500(3/40)	0.38889(7/18)	0.09524(2/21)	0.58824(10/17)
Bo_TE_79849	0.78571(33/42)	1.00000(18/18)	1.00000(18/18)	0.50000(8/16)
Bo_TE_101445	0.79545(35/44)	0.05556(1/18)	0.33333(4/12)	0.00000(0/20)
Bo_TE_150850	0.14634(6/41)	0.58824(10/17)	0.76190(16/21)	0.25000(5/20)
Bo_TE_202119	0.00000(0/44)	0.63158(12/19)	0.17391(4/23)	0.15000(3/20)
Bo_TE_210267	0.00000(0/45)	0.21053(4/19)	0.09091(2/22)	0.52941(9/17)
Bo_TE_52561	0.04762(2/42)	0.05556(1/18)	0.00000(0/22)	0.64706(11/17)
Bo_TE_37702	0.81579(31/38)	0.11111(2/18)	0.40909(9/22)	0.10526(2/19)
Bo_TE_25187	0.51282(20/39)	0.27778(5/18)	0.00000(0/19)	0.15789(3/19)
Bo_TE_223429	1.00000(44/44)	0.82353(14/17)	0.20000(4/20)	0.50000(9/18)
Bo_TE_31555	0.12195(5/41)	0.11765(2/17)	0.00000(0/20)	0.73684(14/19)
Bo_TE_230166	0.06977(3/43)	0.05263(1/19)	0.00000(0/21)	0.58824(10/17)
Bo_TE_29000	0.39535(17/43)	0.72222(13/18)	0.83333(15/18)	0.15789(3/19)
Bo_TE_118374	0.65854(27/41)	0.83333(15/18)	0.04762(1/21)	0.05000(1/20)
Bo_TE_132445	0.88636(39/44)	0.88235(15/17)	0.10000(2/20)	0.47368(9/19)
Bo_TE_31527	0.23810(10/42)	0.87500(14/16)	1.00000(22/22)	0.26316(5/19)
Bo_TE_97422	0.36364(16/44)	1.00000(18/18)	0.95238(20/21)	0.73684(14/19)
Bo_TE_235574	0.81818(36/44)	0.73333(11/15)	0.04545(1/22)	0.42857(6/14)
Bo_TE_91555	0.72727(32/44)	0.55556(10/18)	0.78261(18/23)	0.21053(4/19)
Bo_TE_62457	0.00000(0/43)	0.00000(0/19)	0.08696(2/23)	0.52632(10/19)
Bo_TE_50474	0.95238(40/42)	0.66667(12/18)	0.15789(3/19)	0.18750(3/16)
Bo_TE_235767	0.93333(42/45)	0.80000(12/15)	0.71429(15/21)	0.22222(4/18)
Bo_TE_105718	0.97826(45/46)	1.00000(19/19)	0.66667(14/21)	0.26316(5/19)
Bo_TE_86235	0.36364(16/44)	0.22222(4/18)	0.36364(8/22)	0.73684(14/19)
Bo_TE_13583	0.13636(6/44)	1.00000(16/16)	1.00000(22/22)	0.68421(13/19)
Bo_TE_186118	0.22222(10/45)	0.23529(4/17)	0.81818(18/22)	0.23529(4/17)
Bo_TE_43875	0.24390(10/41)	0.77778(14/18)	0.73913(17/23)	0.83333(15/18)
Bo_TE_148356	0.69231(27/39)	0.50000(9/18)	0.00000(0/23)	0.00000(0/20)
Bo_TE_85992	0.46667(21/45)	0.64706(11/17)	0.13636(3/22)	0.00000(0/19)
Bo_TE_83940	0.11364(5/44)	0.00000(0/18)	0.65000(13/20)	0.77778(14/18)
Bo_TE_154868	0.75000(33/44)	0.18750(3/16)	0.85714(18/21)	0.95000(19/20)
Bo_TE_86635	0.68182(30/44)	0.40000(6/15)	0.00000(0/19)	0.05263(1/19)
Bo_TE_10497	0.25581(11/43)	0.10000(1/10)	0.42857(9/21)	1.00000(20/20)
Bo_TE_31102	0.66667(28/42)	1.00000(17/17)	1.00000(23/23)	0.40000(8/20)
Bo_TE_237143	0.14634(6/41)	0.17647(3/17)	1.00000(20/20)	1.00000(19/19)
Bo_TE_86042	0.09091(4/44)	0.37500(6/16)	0.76190(16/21)	0.00000(0/20)
Bo_TE_123741	0.68293(28/41)	0.05882(1/17)	0.00000(0/22)	0.22222(4/18)
Bo_TE_28057	0.77273(34/44)	0.11111(2/18)	0.47619(10/21)	0.10000(2/20)
Bo_TE_85916	0.08889(4/45)	0.57895(11/19)	0.00000(0/22)	0.31579(6/19)
Bo_TE_175039	0.00000(0/46)	0.00000(0/19)	0.52174(12/23)	0.52941(9/17)

Bo_TE_24910	0.12195(5/41)	0.66667(12/18)	0.91304(21/23)	0.90000(18/20)
Bo_TE_24894	0.50000(20/40)	0.00000(0/17)	0.72727(16/22)	0.15000(3/20)
Bo_TE_237249	0.61905(26/42)	0.05882(1/17)	0.57143(12/21)	0.25000(4/16)
Bo_TE_12828	1.00000(46/46)	1.00000(9/9)	0.47619(10/21)	0.47059(8/17)
Bo_TE_21965	0.62791(27/43)	0.85714(12/14)	0.00000(0/11)	0.00000(0/19)
Bo_TE_227636	0.21053(8/38)	0.12500(2/16)	0.80952(17/21)	0.95000(19/20)
Bo_TE_102188	0.95122(39/41)	1.00000(17/17)	0.60000(12/20)	0.27778(5/18)
Bo_TE_6994	0.07895(3/38)	0.00000(0/14)	0.61111(11/18)	0.72222(13/18)
Bo_TE_10389	0.29545(13/44)	0.87500(14/16)	0.52174(12/23)	0.88889(16/18)
Bo_TE_108568	0.93182(41/44)	0.38889(7/18)	0.55000(11/20)	0.95000(19/20)
Bo_TE_17012	0.00000(0/46)	0.58824(10/17)	0.00000(0/21)	0.00000(0/20)
Bo_TE_226239	0.42105(16/38)	0.77778(14/18)	0.00000(0/23)	0.05263(1/19)
Bo_TE_137600	0.39474(15/38)	0.64286(9/14)	0.95238(20/21)	0.83333(15/18)
Bo_TE_172900	0.90909(40/44)	1.00000(18/18)	0.42857(9/21)	0.36842(7/19)
Bo_TE_32202	0.06667(3/45)	0.88235(15/17)	0.04348(1/23)	0.78947(15/19)
Bo_TE_43700	0.83333(35/42)	0.56250(9/16)	0.00000(0/22)	0.40000(8/20)
Bo_TE_163252	0.62791(27/43)	0.05556(1/18)	0.00000(0/23)	0.00000(0/19)
Bo_TE_49755	0.57895(22/38)	0.16667(3/18)	0.71429(15/21)	0.58824(10/17)
Bo_TE_200182	0.18182(8/44)	0.50000(8/16)	0.73684(14/19)	0.78947(15/19)
Bo_TE_136707	0.58974(23/39)	0.33333(6/18)	0.04762(1/21)	0.06250(1/16)
Bo_TE_119581	0.35556(16/45)	1.00000(17/17)	0.31818(7/22)	0.60000(12/20)
Bo_TE_3816	0.85366(35/41)	0.00000(0/18)	0.61905(13/21)	0.00000(0/20)
Bo_TE_133695	0.46341(19/41)	0.83333(15/18)	0.09091(2/22)	0.21053(4/19)
Bo_TE_198362	0.31707(13/41)	0.27273(3/11)	0.85714(6/7)	1.00000(19/19)
Bo_TE_2196	0.50000(22/44)	0.75000(12/16)	0.08696(2/23)	0.15000(3/20)
Bo_TE_36557	0.65909(29/44)	0.35294(6/17)	0.76190(16/21)	0.15000(3/20)
Bo_TE_183933	0.10870(5/46)	0.31579(6/19)	0.95000(19/20)	0.95000(19/20)
Bo_TE_130511	0.85366(35/41)	0.66667(12/18)	0.22727(5/22)	0.75000(15/20)
Bo_TE_46507	0.58537(24/41)	0.43750(7/16)	0.95238(20/21)	0.26316(5/19)
Bo_TE_34883	0.21951(9/41)	0.00000(0/17)	0.80952(17/21)	0.36842(7/19)
Bo_TE_22989	0.19565(9/46)	0.00000(0/17)	0.22727(5/22)	0.50000(9/18)
Bo_TE_79684	0.30233(13/43)	0.70588(12/17)	0.00000(0/23)	0.00000(0/20)
Bo_TE_43196	0.90909(40/44)	0.31250(5/16)	1.00000(23/23)	1.00000(20/20)
Bo_TE_138556	0.54545(24/44)	0.18750(3/16)	0.95652(22/23)	0.65000(13/20)
Bo_TE_175111	0.69767(30/43)	0.82353(14/17)	0.60870(14/23)	0.10526(2/19)
Bo_TE_92350	0.30952(13/42)	0.58824(10/17)	0.95238(20/21)	0.27778(5/18)
Bo_TE_125494	0.29268(12/41)	0.88889(16/18)	0.35000(7/20)	0.05263(1/19)
Bo_TE_124216	0.25000(11/44)	0.81250(13/16)	0.80952(17/21)	0.85000(17/20)
Bo_TE_182251	0.00000(0/42)	0.52632(10/19)	0.57143(12/21)	0.45000(9/20)
Bo_TE_38955	0.18605(8/43)	0.72222(13/18)	0.14286(3/21)	0.82353(14/17)
Bo_TE_42675	0.84783(39/46)	0.33333(6/18)	0.35000(7/20)	0.20000(4/20)
Bo_TE_3777	0.04651(2/43)	0.23529(4/17)	0.73684(14/19)	0.35000(7/20)
Bo_TE_120881	0.41860(18/43)	0.29412(5/17)	0.63158(12/19)	0.00000(0/19)
Bo_TE_41148	0.06818(3/44)	0.00000(0/19)	0.04545(1/22)	0.73684(14/19)
Bo_TE_148128	0.82500(33/40)	0.11111(2/18)	0.36364(8/22)	0.95000(19/20)
Bo_TE_159642	0.02326(1/43)	0.25000(4/16)	0.09524(2/21)	0.87500(14/16)
Bo_TE_93434	0.30952(13/42)	0.50000(8/16)	1.00000(22/22)	1.00000(19/19)
Bo_TE_101581	0.95556(43/45)	0.46667(7/15)	0.23810(5/21)	0.63158(12/19)
Bo_TE_89500	0.37500(15/40)	0.31250(5/16)	0.70000(14/20)	0.95000(19/20)
Bo_TE_87412	0.50000(20/40)	0.23529(4/17)	0.68182(15/22)	1.00000(20/20)
Bo_TE_109139	0.10256(4/39)	0.50000(8/16)	0.00000(0/20)	0.22222(4/18)
Bo_TE_136363	0.83721(36/43)	0.11765(2/17)	0.35000(7/20)	0.00000(0/19)
Bo_TE_104588	0.95000(38/40)	0.80000(12/15)	0.26316(5/19)	0.23529(4/17)
Bo_TE_5253	0.26190(11/42)	0.72222(13/18)	1.00000(22/22)	1.00000(20/20)
Bo_TE_129190	0.70000(28/40)	0.00000(0/19)	0.81818(18/22)	0.00000(0/18)
Bo_TE_2976	0.00000(0/44)	0.64706(11/17)	0.00000(0/23)	0.00000(0/20)

Bo_TE_97583	0.11111(5/45)	0.10526(2/19)	0.76190(16/21)	0.05000(1/20)
Bo_TE_149074	0.26190(11/42)	0.42105(8/19)	1.00000(23/23)	1.00000(20/20)
Bo_TE_227117	0.97674(42/43)	0.80000(12/15)	0.95238(20/21)	0.15000(3/20)
Bo_TE_17224	0.00000(0/43)	0.46154(6/13)	0.52632(10/19)	0.50000(8/16)
Bo_TE_52773	0.55263(21/38)	0.93333(14/15)	0.00000(0/22)	0.33333(2/6)
Bo_TE_223656	0.87500(35/40)	0.00000(0/16)	0.20000(4/20)	0.05000(1/20)
Bo_TE_22980	0.13636(6/44)	0.68750(11/16)	0.47826(11/23)	0.11111(2/18)
Bo_TE_20857	0.00000(0/45)	0.58824(10/17)	0.00000(0/20)	0.26316(5/19)
Bo_TE_138465	0.23256(10/43)	0.15789(3/19)	0.18182(4/22)	0.73684(14/19)
Bo_TE_129298	0.90698(39/43)	0.94118(16/17)	0.28571(6/21)	0.30000(6/20)
Bo_TE_124154	0.00000(0/42)	0.66667(12/18)	0.08696(2/23)	0.84211(16/19)
Bo_TE_125765	0.25581(11/43)	0.11765(2/17)	0.20000(4/20)	0.80000(16/20)
Bo_TE_10884	0.88636(39/44)	0.75000(12/16)	0.90476(19/21)	0.15789(3/19)
Bo_TE_117520	0.09302(4/43)	0.84211(16/19)	0.52632(10/19)	0.31250(5/16)
Bo_TE_1635	0.06818(3/44)	0.68750(11/16)	0.28571(6/21)	0.00000(0/20)
Bo_TE_184878	0.54762(23/42)	0.64706(11/17)	0.66667(14/21)	0.00000(0/20)
Bo_TE_211575	0.27273(12/44)	0.11765(2/17)	1.00000(21/21)	0.78947(15/19)
Bo_TE_142007	0.97778(44/45)	0.44444(8/18)	0.38095(8/21)	0.55556(10/18)
Bo_TE_49571	0.34211(13/38)	1.00000(16/16)	0.52381(11/21)	0.84211(16/19)
Bo_TE_41460	0.20455(9/44)	1.00000(17/17)	0.13043(3/23)	0.84211(16/19)
Bo_TE_180811	0.56522(26/46)	0.60000(9/15)	0.00000(0/23)	0.16667(3/18)
Bo_TE_204463	0.17500(7/40)	0.27778(5/18)	0.57143(12/21)	0.05000(1/20)
Bo_TE_226315	0.78378(29/37)	0.16667(3/18)	1.00000(23/23)	0.95000(19/20)
Bo_TE_222067	0.13043(6/46)	0.05882(1/17)	0.09091(2/22)	0.73684(14/19)
Bo_TE_154797	0.09302(4/43)	0.76471(13/17)	0.00000(0/22)	0.00000(0/19)
Bo_TE_20321	0.11364(5/44)	0.82353(14/17)	0.13636(3/22)	0.25000(5/20)
Bo_TE_6144	0.25000(11/44)	0.56250(9/16)	0.04762(1/21)	0.40000(8/20)
Bo_TE_88987	0.78571(33/42)	0.43750(7/16)	0.66667(14/21)	0.10526(2/19)
Bo_TE_198758	0.02273(1/44)	0.29412(5/17)	0.57143(12/21)	0.10000(2/20)
Bo_TE_125785	0.21429(9/42)	0.05882(1/17)	0.20000(4/20)	0.60000(12/20)
Bo_TE_148608	0.58974(23/39)	0.44444(8/18)	0.40000(8/20)	0.00000(0/20)
Bo_TE_236860	0.51220(21/41)	0.66667(12/18)	0.30435(7/23)	0.10000(2/20)
Bo_TE_170654	0.43590(17/39)	0.72222(13/18)	1.00000(20/20)	0.31579(6/19)
Bo_TE_165554	0.16279(7/43)	0.68750(11/16)	0.40909(9/22)	0.80000(16/20)
Bo_TE_219048	0.17500(7/40)	0.94444(17/18)	0.70000(14/20)	0.78947(15/19)
Bo_TE_127329	0.15000(6/40)	0.72222(13/18)	1.00000(23/23)	1.00000(20/20)
Bo_TE_124921	0.00000(0/44)	0.00000(0/11)	0.10000(2/20)	0.55556(10/18)
Bo_TE_137801	0.58537(24/41)	0.31250(5/16)	0.22727(5/22)	0.94737(18/19)
Bo_TE_144698	0.84444(38/45)	0.10526(2/19)	0.00000(0/23)	0.57895(11/19)
Bo_TE_183449	0.00000(0/39)	0.83333(15/18)	0.61905(13/21)	0.31579(6/19)
Bo_TE_47030	0.35000(14/40)	0.62500(10/16)	0.11111(2/18)	0.05556(1/18)
Bo_TE_70045	0.70732(29/41)	0.06250(1/16)	0.85000(17/20)	0.35000(7/20)
Bo_TE_103150	0.18605(8/43)	0.38889(7/18)	0.71429(15/21)	0.44444(8/18)
Bo_TE_215436	0.59524(25/42)	0.27778(5/18)	0.19048(4/21)	0.95000(19/20)
Bo_TE_43053	0.83333(35/42)	0.62500(10/16)	0.86364(19/22)	0.05263(1/19)
Bo_TE_156994	0.13043(6/46)	0.00000(0/17)	0.63636(14/22)	0.30000(6/20)
Bo_TE_16192	0.55814(24/43)	0.94118(16/17)	0.14286(3/21)	0.35000(7/20)
Bo_TE_218044	0.27273(12/44)	0.66667(12/18)	0.47619(10/21)	0.15000(3/20)
Bo_TE_72388	0.55814(24/43)	0.58824(10/17)	0.04762(1/21)	0.38889(7/18)
Bo_TE_59056	0.17778(8/45)	0.81250(13/16)	0.59091(13/22)	0.31579(6/19)
Bo_TE_165261	0.00000(0/45)	0.46667(7/15)	0.61905(13/21)	0.73684(14/19)
Bo_TE_168336	0.69048(29/42)	0.47059(8/17)	1.00000(21/21)	1.00000(19/19)
Bo_TE_74460	1.00000(46/46)	0.68750(11/16)	0.45000(9/20)	0.89474(17/19)
Bo_TE_124374	0.88095(37/42)	0.42105(8/19)	1.00000(15/15)	0.94737(18/19)
Bo_TE_181621	0.00000(0/44)	0.00000(0/19)	0.50000(10/20)	0.00000(0/20)
Bo_TE_188648	0.68889(31/45)	0.11111(2/18)	0.04348(1/23)	0.05000(1/20)

Bo_TE_121932	0.50000(21/42)	0.88889(16/18)	0.95652(22/23)	0.31579(6/19)
Bo_TE_27168	0.07143(3/42)	0.00000(0/16)	0.77273(17/22)	0.75000(15/20)
Bo_TE_97177	1.00000(46/46)	1.00000(17/17)	0.19048(4/21)	0.20000(4/20)
Bo_TE_169148	0.22222(8/36)	0.18750(3/16)	1.00000(23/23)	0.75000(15/20)
Bo_TE_91303	0.00000(0/45)	0.20000(3/15)	0.59091(13/22)	0.61111(11/18)
Bo_TE_216170	0.02326(1/43)	0.28571(4/14)	0.09091(2/22)	0.55000(11/20)
Bo_TE_87873	0.90476(38/42)	0.76471(13/17)	0.28571(6/21)	0.83333(15/18)
Bo_TE_96653	0.80952(34/42)	0.43750(7/16)	0.09524(2/21)	0.50000(10/20)
Bo_TE_53609	0.95349(41/43)	1.00000(18/18)	0.26316(5/19)	0.10526(2/19)
Bo_TE_114377	0.64286(27/42)	0.70588(12/17)	0.00000(0/21)	0.05000(1/20)
Bo_TE_33539	0.91111(41/45)	0.31250(5/16)	0.80952(17/21)	0.15789(3/19)
Bo_TE_179424	0.23256(10/43)	0.25000(4/16)	0.90909(20/22)	0.21053(4/19)
Bo_TE_17263	0.95455(42/44)	0.50000(8/16)	0.52381(11/21)	0.20000(4/20)
Bo_TE_128463	0.27273(12/44)	0.72222(13/18)	0.66667(14/21)	0.00000(0/20)
Bo_TE_208729	0.21951(9/41)	0.76471(13/17)	0.13636(3/22)	0.05263(1/19)
Bo_TE_152403	0.83333(35/42)	0.10526(2/19)	0.45000(9/20)	0.10000(2/20)
Bo_TE_121535	0.00000(0/45)	0.00000(0/17)	0.04348(1/23)	0.65000(13/20)
Bo_TE_32602	0.12000(3/25)	0.94118(16/17)	0.52632(10/19)	0.18750(3/16)
Bo_TE_173243	0.54054(20/37)	0.25000(4/16)	0.27273(6/22)	0.00000(0/19)
Bo_TE_28812	0.86667(39/45)	0.06250(1/16)	0.81818(18/22)	0.84211(16/19)
Bo_TE_80644	0.65116(28/43)	0.11111(2/18)	0.45000(9/20)	0.00000(0/20)
Bo_TE_126178	0.35000(14/40)	0.80000(12/15)	0.86364(19/22)	0.05263(1/19)
Bo_TE_215872	0.33333(13/39)	0.58824(10/17)	0.00000(0/23)	0.00000(0/19)
Bo_TE_182721	0.04545(2/44)	0.35294(6/17)	0.57143(12/21)	0.31579(6/19)
Bo_TE_170841	0.79545(35/44)	0.77778(14/18)	0.56522(13/23)	0.05263(1/19)
Bo_TE_76054	0.59524(25/42)	0.00000(0/18)	0.00000(0/22)	0.10000(2/20)
Bo_TE_59629	0.45455(20/44)	0.73333(11/15)	0.95455(21/22)	0.78947(15/19)
Bo_TE_83904	0.90244(37/41)	1.00000(18/18)	0.42857(9/21)	0.21053(4/19)
Bo_TE_81600	0.63636(28/44)	0.00000(0/19)	0.63636(14/22)	0.68421(13/19)
Bo_TE_145923	0.00000(0/42)	0.16667(3/18)	0.52381(11/21)	0.52941(9/17)
Bo_TE_98736	0.88372(38/43)	0.70588(12/17)	0.60000(12/20)	0.15000(3/20)
Bo_TE_13372	0.67500(27/40)	0.00000(0/18)	0.00000(0/22)	0.00000(0/20)
Bo_TE_140212	0.86047(37/43)	0.05882(1/17)	0.40909(9/22)	0.10526(2/19)
Bo_TE_148472	0.97727(43/44)	0.17647(3/17)	0.89474(17/19)	0.64706(11/17)
Bo_TE_120952	0.69048(29/42)	0.70588(12/17)	0.27273(6/22)	0.10000(2/20)
Bo_TE_119213	0.86047(37/43)	0.40000(6/15)	0.76190(16/21)	0.05882(1/17)
Bo_TE_209687	0.15556(7/45)	0.83333(15/18)	0.40909(9/22)	0.05000(1/20)
Bo_TE_197489	0.97727(43/44)	0.89474(17/19)	0.82609(19/23)	0.40000(8/20)
Bo_TE_82133	0.77273(34/44)	0.66667(12/18)	0.42857(9/21)	0.00000(0/20)
Bo_TE_116964	0.10256(4/39)	0.33333(2/6)	0.86957(20/23)	0.11111(2/18)
Bo_TE_152211	0.04348(2/46)	0.58824(10/17)	0.00000(0/21)	0.00000(0/18)
Bo_TE_231488	0.80488(33/41)	0.11765(2/17)	0.19048(4/21)	0.47368(9/19)
Bo_TE_41972	0.24444(11/45)	0.50000(9/18)	0.76190(16/21)	0.72222(13/18)
Bo_TE_85862	0.38462(15/39)	0.11765(2/17)	0.90000(18/20)	0.68421(13/19)
Bo_TE_125451	0.54762(23/42)	1.00000(18/18)	0.59091(13/22)	0.35000(7/20)
Bo_TE_184434	0.21429(9/42)	0.47059(8/17)	0.75000(15/20)	0.60000(12/20)
Bo_TE_19914	0.12195(5/41)	0.10526(2/19)	1.00000(23/23)	0.85000(17/20)
Bo_TE_96339	0.44186(19/43)	0.94444(17/18)	1.00000(16/16)	0.75000(3/4)
Bo_TE_24947	0.11111(5/45)	0.23529(4/17)	0.22727(5/22)	0.75000(15/20)
Bo_TE_165697	0.63636(28/44)	0.06250(1/16)	0.00000(0/23)	0.10000(2/20)
Bo_TE_174344	0.42500(17/40)	0.17647(3/17)	0.73684(14/19)	0.05000(1/20)
Bo_TE_6715	0.89130(41/46)	1.00000(19/19)	0.59091(13/22)	0.11111(2/18)
Bo_TE_202000	0.94872(37/39)	0.94444(17/18)	0.35000(7/20)	0.85000(17/20)
Bo_TE_57432	0.97778(44/45)	1.00000(19/19)	0.57895(11/19)	0.31579(6/19)
Bo_TE_100187	0.18182(8/44)	0.00000(0/18)	0.60000(12/20)	0.50000(9/18)
Bo_TE_58072	0.59524(25/42)	0.93750(15/16)	0.21739(5/23)	0.21053(4/19)

Bo_TE_140011	1.00000(44/44)	0.58824(10/17)	0.36364(8/22)	0.55000(11/20)
Bo_TE_107701	0.66667(30/45)	0.37500(6/16)	0.04545(1/22)	0.23529(4/17)
Bo_TE_151015	0.00000(0/45)	0.56250(9/16)	0.00000(0/20)	0.26316(5/19)
Bo_TE_126473	0.62500(25/40)	0.25000(4/16)	0.00000(0/23)	0.00000(0/20)
Bo_TE_44934	0.29268(12/41)	0.88235(15/17)	0.82609(19/23)	0.57895(11/19)
Bo_TE_19940	0.55000(22/40)	0.00000(0/17)	0.00000(0/22)	0.10526(2/19)
Bo_TE_156611	0.71429(30/42)	0.00000(0/16)	0.18182(4/22)	0.00000(0/20)
Bo_TE_98166	0.21951(9/41)	0.00000(0/17)	0.00000(0/23)	0.55556(10/18)
Bo_TE_213972	0.67442(29/43)	0.78571(11/14)	0.45000(9/20)	0.10000(2/20)
Bo_TE_101379	1.00000(43/43)	0.50000(8/16)	0.70000(14/20)	0.50000(9/18)
Bo_TE_138502	0.32500(13/40)	0.17647(3/17)	0.90909(20/22)	0.68421(13/19)
Bo_TE_209924	0.28571(12/42)	0.27778(5/18)	0.72222(13/18)	1.00000(19/19)
Bo_TE_227341	0.41463(17/41)	0.43750(7/16)	0.68182(15/22)	0.00000(0/20)
Bo_TE_179670	0.31111(14/45)	0.78571(11/14)	0.77273(17/22)	1.00000(19/19)
Bo_TE_134749	0.00000(0/46)	0.64706(11/17)	0.04545(1/22)	0.00000(0/20)
Bo_TE_52365	0.04444(2/45)	0.61111(11/18)	0.08696(2/23)	0.22222(4/18)
Bo_TE_224703	0.65000(26/40)	0.66667(2/3)	0.13636(3/22)	0.00000(0/19)
Bo_TE_52658	0.32558(14/43)	0.16667(3/18)	0.00000(0/23)	0.82353(14/17)
Bo_TE_91477	0.00000(0/45)	0.15789(3/19)	0.76190(16/21)	0.44444(8/18)
Bo_TE_236412	0.18182(8/44)	0.88235(15/17)	0.40000(8/20)	0.11111(2/18)
Bo_TE_119714	0.74419(32/43)	0.88235(15/17)	0.80000(16/20)	0.15000(3/20)
Bo_TE_36552	0.25581(11/43)	1.00000(18/18)	0.66667(14/21)	0.77778(14/18)
Bo_TE_41886	0.73171(30/41)	0.50000(9/18)	0.09524(2/21)	0.15789(3/19)
Bo_TE_44002	0.00000(0/42)	0.73684(14/19)	0.00000(0/21)	0.10526(2/19)
Bo_TE_180602	0.02326(1/43)	0.72222(13/18)	0.04762(1/21)	0.35000(7/20)
Bo_TE_15460	0.30233(13/43)	0.05882(1/17)	0.00000(0/23)	0.55556(10/18)
Bo_TE_54529	0.02174(1/46)	0.50000(8/16)	0.30000(6/20)	0.88889(16/18)
Bo_TE_127050	0.00000(0/41)	0.00000(0/19)	0.63636(14/22)	0.00000(0/20)
Bo_TE_135808	0.13158(5/38)	0.00000(0/7)	0.36364(8/22)	0.94118(16/17)
Bo_TE_201854	0.04444(2/45)	0.22222(4/18)	0.29412(5/17)	0.57895(11/19)
Bo_TE_220196	0.72973(27/37)	0.21053(4/19)	0.45455(10/22)	0.57895(11/19)
Bo_TE_122579	0.10256(4/39)	0.76471(13/17)	0.00000(0/23)	0.10000(2/20)
Bo_TE_114147	0.02273(1/44)	0.00000(0/17)	0.82609(19/23)	0.00000(0/16)
Bo_TE_236078	1.00000(42/42)	0.94118(16/17)	0.45000(9/20)	0.11765(2/17)
Bo_TE_12545	0.80952(34/42)	0.11765(2/17)	0.47619(10/21)	0.10526(2/19)
Bo_TE_28401	0.63415(26/41)	0.16667(3/18)	0.00000(0/23)	0.10000(2/20)
Bo_TE_27183	0.90698(39/43)	1.00000(17/17)	0.40000(6/15)	0.26316(5/19)
Bo_TE_159423	0.38462(15/39)	0.93750(15/16)	0.65000(13/20)	0.00000(0/4)
Bo_TE_140511	0.57500(23/40)	0.23529(4/17)	0.35000(7/20)	0.00000(0/18)
Bo_TE_22968	0.00000(0/45)	0.00000(0/19)	0.36364(8/22)	0.73684(14/19)
Bo_TE_85767	1.00000(45/45)	0.72222(13/18)	0.86957(20/23)	0.50000(10/20)
Bo_TE_46160	0.02222(1/45)	0.52632(10/19)	0.00000(0/23)	0.00000(0/20)
Bo_TE_70263	0.23077(9/39)	0.50000(9/18)	0.20000(4/20)	0.84211(16/19)
Bo_TE_11943	0.00000(0/43)	0.52941(9/17)	0.09091(2/22)	0.05263(1/19)
Bo_TE_118521	0.63415(26/41)	0.11111(2/18)	0.00000(0/20)	0.00000(0/19)
Bo_TE_8821	0.65854(27/41)	0.88235(15/17)	0.28571(6/21)	0.90000(18/20)
Bo_TE_230091	0.40909(18/44)	0.12500(2/16)	0.90000(18/20)	0.78947(15/19)
Bo_TE_61445	0.00000(0/46)	0.00000(0/17)	0.04545(1/22)	0.50000(9/18)
Bo_TE_215746	0.88095(37/42)	0.77778(14/18)	0.45455(10/22)	1.00000(20/20)
Bo_TE_238470	0.00000(0/43)	0.76471(13/17)	0.09091(2/22)	0.00000(0/20)
Bo_TE_141559	0.15000(6/40)	0.88235(15/17)	0.95455(21/22)	0.77778(14/18)
Bo_TE_140441	0.77273(34/44)	0.66667(12/18)	0.45455(10/22)	0.15000(3/20)
Bo_TE_225988	0.73333(33/45)	0.14286(2/14)	0.00000(0/18)	0.00000(0/19)
Bo_TE_231246	1.00000(45/45)	1.00000(14/14)	0.54545(12/22)	0.42105(8/19)
Bo_TE_71218	0.48718(19/39)	0.00000(0/18)	0.71429(15/21)	0.25000(5/20)
Bo_TE_140357	0.69048(29/42)	0.76471(13/17)	0.36364(8/22)	1.00000(20/20)

Bo_TE_180080	0.63636(28/44)	0.18750(3/16)	0.19048(4/21)	0.95000(19/20)
Bo_TE_142048	0.76190(32/42)	0.82353(14/17)	0.69565(16/23)	0.26316(5/19)
Bo_TE_181645	0.54348(25/46)	0.43750(7/16)	0.95455(21/22)	0.15789(3/19)
Bo_TE_30900	0.20000(8/40)	0.33333(6/18)	0.75000(15/20)	0.20000(4/20)
Bo_TE_74422	0.06977(3/43)	0.52941(9/17)	0.17391(4/23)	0.00000(0/20)
Bo_TE_222347	0.15217(7/46)	0.05882(1/17)	0.61905(13/21)	0.15789(3/19)
Bo_TE_65954	0.24390(10/41)	0.94118(16/17)	0.36364(8/22)	0.38889(7/18)
Bo_TE_138294	0.68421(26/38)	0.47368(9/19)	0.80000(16/20)	0.05263(1/19)
Bo_TE_134809	0.83721(36/43)	0.35294(6/17)	0.89474(17/19)	0.95000(19/20)
Bo_TE_2391	0.04348(2/46)	0.61111(11/18)	0.00000(0/23)	0.00000(0/19)
Bo_TE_204500	0.55814(24/43)	0.33333(6/18)	0.00000(0/21)	0.84211(16/19)
Bo_TE_194813	0.50000(6/12)	0.50000(2/4)	0.63636(14/22)	0.00000(0/18)
Bo_TE_177514	0.72093(31/43)	0.88235(15/17)	0.14286(3/21)	0.73684(14/19)
Bo_TE_41931	0.28889(13/45)	0.93750(15/16)	0.90000(18/20)	0.85000(17/20)
Bo_TE_49507	0.02222(1/45)	0.53333(8/15)	0.54545(12/22)	0.75000(15/20)
Bo_TE_86871	0.15556(7/45)	0.76471(13/17)	0.80952(17/21)	0.82353(14/17)
Bo_TE_215723	0.52381(22/42)	0.00000(0/18)	0.00000(0/22)	0.00000(0/20)
Bo_TE_169710	0.06977(3/43)	0.75000(12/16)	0.04545(1/22)	0.47368(9/19)
Bo_TE_37363	0.70732(29/41)	0.17647(3/17)	0.14286(3/21)	0.00000(0/19)
Bo_TE_10764	0.53659(22/41)	0.82353(14/17)	0.00000(0/22)	0.00000(0/20)
Bo_TE_129326	0.07143(1/14)	0.00000(0/10)	0.00000(0/23)	0.76471(13/17)
Bo_TE_155205	0.55814(24/43)	0.82353(14/17)	0.61905(13/21)	0.26316(5/19)
Bo_TE_92621	0.86047(37/43)	0.41176(7/17)	0.00000(0/22)	0.05000(1/20)
Bo_TE_129807	0.47500(19/40)	0.06250(1/16)	0.60000(12/20)	0.15789(3/19)
Bo_TE_77245	0.57500(23/40)	0.05882(1/17)	0.00000(0/23)	0.00000(0/20)
Bo_TE_205997	0.00000(0/46)	0.05556(1/18)	0.85714(18/21)	0.85000(17/20)
Bo_TE_102342	0.88636(39/44)	0.75000(12/16)	0.55000(11/20)	0.15789(3/19)
Bo_TE_150932	0.76923(30/39)	0.29412(5/17)	0.77273(17/22)	0.25000(5/20)
Bo_TE_165243	0.53846(21/39)	0.00000(0/17)	0.13636(3/22)	0.10000(2/20)
Bo_TE_24420	0.13333(6/45)	0.88235(15/17)	0.91304(21/23)	0.95000(19/20)
Bo_TE_41236	0.09091(4/44)	0.68750(11/16)	0.36364(8/22)	0.68421(13/19)
Bo_TE_80611	0.73171(30/41)	0.75000(12/16)	0.52632(10/19)	0.10000(2/20)
Bo_TE_45645	0.35714(15/42)	0.81250(13/16)	0.33333(7/21)	0.21053(4/19)
Bo_TE_13703	0.27500(11/40)	0.62500(10/16)	0.61111(11/18)	0.90000(18/20)
Bo_TE_39117	0.88372(38/43)	0.62500(10/16)	0.00000(0/21)	0.00000(0/19)
Bo_TE_226957	0.47500(19/40)	0.18750(3/16)	1.00000(22/22)	0.85000(17/20)
Bo_TE_29517	1.00000(40/40)	1.00000(18/18)	0.85000(17/20)	0.42105(8/19)
Bo_TE_93675	0.00000(0/42)	0.00000(0/18)	0.80952(17/21)	0.00000(0/20)
Bo_TE_6666	0.64444(29/45)	0.00000(0/17)	0.00000(0/22)	0.23529(4/17)
Bo_TE_231083	0.00000(0/44)	0.72222(13/18)	0.00000(0/21)	0.61111(11/18)
Bo_TE_62882	0.02273(1/44)	0.66667(12/18)	0.45000(9/20)	0.00000(0/20)
Bo_TE_92302	0.68293(28/41)	0.31250(5/16)	1.00000(21/21)	0.36842(7/19)
Bo_TE_186215	0.67500(27/40)	0.11111(2/18)	0.52632(10/19)	0.73684(14/19)
Bo_TE_15452	0.25926(7/27)	0.93333(14/15)	1.00000(23/23)	0.50000(9/18)
Bo_TE_39926	0.95556(43/45)	0.35294(6/17)	0.76190(16/21)	0.30000(6/20)
Bo_TE_191910	0.85714(36/42)	0.33333(6/18)	1.00000(23/23)	0.94737(18/19)
Bo_TE_67817	0.12500(5/40)	0.43750(7/16)	0.36364(8/22)	0.80000(16/20)
Bo_TE_185380	0.02222(1/45)	0.55556(10/18)	0.38095(8/21)	0.15000(3/20)
Bo_TE_89380	0.00000(0/40)	0.00000(0/17)	0.78947(15/19)	0.00000(0/17)
Bo_TE_160727	0.93478(43/46)	0.94444(17/18)	0.31579(6/19)	0.55556(10/18)
Bo_TE_64916	0.33333(14/42)	0.42857(6/14)	0.90476(19/21)	0.61111(11/18)
Bo_TE_17443	0.19512(8/41)	0.05882(1/17)	0.00000(0/12)	0.83333(15/18)
Bo_TE_230188	0.85714(36/42)	0.00000(0/18)	0.38095(8/21)	0.73684(14/19)
Bo_TE_62855	0.97826(45/46)	0.33333(6/18)	0.54545(12/22)	1.00000(20/20)
Bo_TE_147878	0.83333(35/42)	0.55556(10/18)	0.23810(5/21)	0.05556(1/18)
Bo_TE_207596	0.37500(15/40)	0.58824(10/17)	0.00000(0/22)	0.21053(4/19)

Bo_TE_96380	0.64444(29/45)	0.94118(16/17)	0.61111(11/18)	0.11111(2/18)
Bo_TE_25106	0.78571(33/42)	0.31250(5/16)	0.00000(0/23)	0.00000(0/17)
Bo_TE_62846	0.00000(0/45)	0.58824(10/17)	0.43478(10/23)	0.00000(0/20)
Bo_TE_83932	0.13636(6/44)	0.00000(0/16)	0.59091(13/22)	0.78947(15/19)
Bo_TE_204976	0.18519(5/27)	0.12500(2/16)	0.35000(7/20)	0.94444(17/18)
Bo_TE_111019	0.29545(13/44)	0.00000(0/17)	0.00000(0/20)	0.61111(11/18)
Bo_TE_125593	0.34146(14/41)	0.66667(12/18)	0.09091(2/22)	0.00000(0/20)
Bo_TE_182423	0.06667(3/45)	0.21053(4/19)	0.60000(12/20)	0.52632(10/19)
Bo_TE_223511	0.00000(0/46)	0.29412(5/17)	0.71429(15/21)	0.78947(15/19)
Bo_TE_55	0.48889(22/45)	0.72222(13/18)	1.00000(22/22)	0.90000(18/20)
Bo_TE_18912	0.02222(1/45)	0.56250(9/16)	0.04348(1/23)	0.05000(1/20)
Bo_TE_53154	0.27500(11/40)	0.29412(5/17)	0.86957(20/23)	0.05882(1/17)
Bo_TE_178668	0.69231(27/39)	0.86667(13/15)	0.54545(12/22)	0.05000(1/20)
Bo_TE_182503	0.70000(28/40)	0.11765(2/17)	0.00000(0/10)	0.00000(0/14)
Bo_TE_99969	0.67568(25/37)	1.00000(17/17)	0.35000(7/20)	0.20000(4/20)
Bo_TE_238288	0.32500(13/40)	0.75000(12/16)	0.00000(0/23)	0.00000(0/19)
Bo_TE_143172	0.02174(1/46)	0.05556(1/18)	0.31818(7/22)	0.73684(14/19)
Bo_TE_136470	0.00000(0/44)	0.00000(0/18)	0.26316(5/19)	0.63158(12/19)
Bo_TE_178326	0.47500(19/40)	0.33333(6/18)	0.22727(5/22)	0.94737(18/19)
Bo_TE_224281	0.00000(0/43)	0.61111(11/18)	0.19048(4/21)	0.40000(8/20)
Bo_TE_67508	0.97297(36/37)	0.92308(12/13)	1.00000(21/21)	0.50000(8/16)
Bo_TE_124359	0.04651(2/43)	0.62500(10/16)	0.00000(0/22)	0.05000(1/20)
Bo_TE_33868	0.71795(28/39)	0.29412(5/17)	0.95455(21/22)	0.55000(11/20)
Bo_TE_120390	0.85714(36/42)	0.77778(14/18)	0.57143(12/21)	0.31250(5/16)
Bo_TE_130805	0.00000(0/43)	0.05556(1/18)	0.00000(0/23)	0.57895(11/19)
Bo_TE_173798	0.00000(0/44)	0.05882(1/17)	0.56522(13/23)	0.89474(17/19)
Bo_TE_196569	1.00000(41/41)	0.27778(5/18)	0.95238(20/21)	0.25000(4/16)
Bo_TE_80047	0.16279(7/43)	0.75000(9/12)	0.71429(5/7)	0.75000(12/16)
Bo_TE_128688	0.02222(1/45)	0.15789(3/19)	0.57143(12/21)	0.00000(0/19)
Bo_TE_18390	0.43590(17/39)	0.06250(1/16)	0.22727(5/22)	0.85000(17/20)
Bo_TE_134718	0.85714(36/42)	0.33333(6/18)	0.85714(18/21)	0.95000(19/20)
Bo_TE_193823	0.39024(16/41)	0.75000(12/16)	0.85000(17/20)	0.15000(3/20)
Bo_TE_154764	0.02273(1/44)	0.60000(9/15)	0.86364(19/22)	0.87500(14/16)
Bo_TE_173635	0.93478(43/46)	0.94444(17/18)	0.33333(6/18)	0.25000(5/20)
Bo_TE_77560	0.00000(0/46)	0.31250(5/16)	0.00000(0/23)	0.73684(14/19)
Bo_TE_44169	0.77273(34/44)	0.88235(15/17)	0.43750(7/16)	0.15000(3/20)
Bo_TE_159677	0.75000(33/44)	0.00000(0/18)	0.30000(6/20)	0.15789(3/19)
Bo_TE_208687	0.14634(6/41)	0.17647(3/17)	0.22727(5/22)	0.94737(18/19)
Bo_TE_131218	0.15217(7/46)	0.87500(14/16)	0.19048(4/21)	0.58824(10/17)
Bo_TE_179701	0.61905(26/42)	0.29412(5/17)	0.18182(4/22)	0.00000(0/19)
Bo_TE_137536	0.29268(12/41)	0.15789(3/19)	0.86957(20/23)	0.68421(13/19)
Bo_TE_182596	0.95652(44/46)	0.64706(11/17)	0.42857(9/21)	0.68421(13/19)
Bo_TE_44017	0.18605(8/43)	0.76471(13/17)	0.05000(1/20)	0.10000(2/20)
Bo_TE_165568	0.15556(7/45)	0.68750(11/16)	0.47619(10/21)	0.70000(14/20)
Bo_TE_112046	0.02273(1/44)	0.56250(9/16)	0.00000(0/23)	0.25000(5/20)
Bo_TE_41869	0.80000(36/45)	0.62500(10/16)	0.10000(2/20)	0.15789(3/19)
Bo_TE_64223	0.11364(5/44)	0.57895(11/19)	0.00000(0/23)	0.00000(0/20)
Bo_TE_32409	0.78571(33/42)	0.00000(0/17)	0.23810(5/21)	0.76471(13/17)
Bo_TE_125529	0.38095(16/42)	0.87500(14/16)	0.31579(6/19)	0.05000(1/20)
Bo_TE_15378	0.27273(12/44)	0.94118(16/17)	1.00000(23/23)	0.40000(8/20)
Bo_TE_21160	0.86047(37/43)	0.12500(2/16)	0.23810(5/21)	0.00000(0/20)
Bo_TE_170563	0.95455(42/44)	0.50000(9/18)	0.70588(12/17)	1.00000(20/20)
Bo_TE_70460	0.87179(34/39)	0.94444(17/18)	0.18182(4/22)	0.63158(12/19)
Bo_TE_172002	0.63636(28/44)	0.21053(4/19)	0.00000(0/23)	0.00000(0/19)
Bo_TE_19748	0.00000(0/44)	0.81250(13/16)	0.45455(10/22)	0.10526(2/19)
Bo_TE_179982	0.09302(4/43)	0.53333(8/15)	0.86957(20/23)	0.72222(13/18)

Bo_TE_138629	0.04348(2/46)	0.76471(13/17)	0.00000(0/23)	0.22222(4/18)
Bo_TE_37465	0.02632(1/38)	0.62500(10/16)	0.00000(0/23)	0.11111(2/18)
Bo_TE_165237	0.54054(20/37)	0.00000(0/17)	0.14286(3/21)	0.10000(2/20)
Bo_TE_234859	0.87805(36/41)	0.41176(7/17)	0.95238(20/21)	0.85000(17/20)
Bo_TE_220094	0.08696(4/46)	0.56250(9/16)	0.00000(0/23)	0.10526(2/19)
Bo_TE_41258	0.08696(4/46)	0.80000(12/15)	0.30000(6/20)	0.63158(12/19)
Bo_TE_119794	0.31818(14/44)	0.90909(10/11)	1.00000(23/23)	0.68421(13/19)
Bo_TE_52144	1.00000(44/44)	0.87500(14/16)	0.40000(8/20)	0.52632(10/19)
Bo_TE_45890	0.95455(42/44)	0.12500(2/16)	0.40000(8/20)	0.10526(2/19)
Bo_TE_15334	0.16667(7/42)	0.55556(10/18)	0.00000(0/22)	0.30000(6/20)
Bo_TE_165140	0.93333(42/45)	0.41176(7/17)	0.70000(14/20)	1.00000(20/20)
Bo_TE_223405	0.97778(44/45)	0.26667(4/15)	0.47826(11/23)	0.31579(6/19)
Bo_TE_93783	0.95652(44/46)	0.35294(6/17)	0.95000(19/20)	0.90000(18/20)
Bo_TE_210330	0.95349(41/43)	0.75000(12/16)	0.80952(17/21)	0.42105(8/19)
Bo_TE_193760	0.26829(11/41)	0.68750(11/16)	0.40909(9/22)	0.15000(3/20)
Bo_TE_174994	0.95238(40/42)	0.94118(16/17)	0.63636(14/22)	0.26316(5/19)
Bo_TE_83025	0.52273(23/44)	0.31579(6/19)	0.00000(0/22)	0.57895(11/19)
Bo_TE_58067	0.06977(3/43)	0.76471(13/17)	0.13636(3/22)	0.15789(3/19)
Bo_TE_164914	0.00000(0/44)	0.00000(0/18)	0.43478(10/23)	0.73684(14/19)
Bo_TE_209872	0.39535(17/43)	0.89474(17/19)	0.00000(0/23)	0.00000(0/20)
Bo_TE_171209	0.52500(21/40)	0.11765(2/17)	0.14286(3/21)	0.70000(14/20)
Bo_TE_80403	0.66667(26/39)	0.05882(1/17)	0.47619(10/21)	0.00000(0/20)
Bo_TE_207943	0.78125(25/32)	0.12500(2/16)	0.82609(19/23)	0.70588(12/17)
Bo_TE_121644	0.51220(21/41)	0.87500(14/16)	0.63636(14/22)	0.21053(4/19)
Bo_TE_191261	0.18605(8/43)	0.88235(15/17)	0.80952(17/21)	0.05263(1/19)
Bo_TE_172511	0.02174(1/46)	0.00000(0/17)	0.04348(1/23)	0.50000(8/16)
Bo_TE_241157	0.95556(43/45)	1.00000(16/16)	0.26087(6/23)	0.70588(12/17)
Bo_TE_4553	0.79070(34/43)	0.06667(1/15)	0.04762(1/21)	0.05000(1/20)
Bo_TE_177444	0.69048(29/42)	0.71429(10/14)	0.95455(21/22)	0.38889(7/18)
Bo_TE_19125	0.68182(30/44)	0.81250(13/16)	0.28571(6/21)	0.38889(7/18)
Bo_TE_132297	0.24390(10/41)	0.12500(2/16)	1.00000(23/23)	1.00000(20/20)
Bo_TE_151400	0.00000(0/41)	0.00000(0/19)	0.00000(0/22)	0.52632(10/19)
Bo_TE_234800	0.88889(40/45)	0.16667(3/18)	0.00000(0/23)	0.11111(2/18)
Bo_TE_236122	0.02222(1/45)	0.11111(2/18)	0.72727(16/22)	0.00000(0/20)
Bo_TE_205599	0.20000(9/45)	0.57895(11/19)	0.00000(0/21)	0.00000(0/19)
Bo_TE_41576	0.04444(2/45)	0.00000(0/19)	0.52174(12/23)	0.61111(11/18)
Bo_TE_203199	0.00000(0/45)	0.00000(0/19)	0.13636(3/22)	0.73684(14/19)
Bo_TE_86590	0.76190(32/42)	0.06250(1/16)	0.13636(3/22)	0.50000(10/20)
Bo_TE_92895	0.52778(19/36)	0.87500(14/16)	0.00000(0/18)	0.15000(3/20)
Bo_TE_188779	0.76190(32/42)	0.76471(13/17)	0.45455(10/22)	1.00000(18/18)
Bo_TE_103016	0.57143(24/42)	0.47059(8/17)	1.00000(21/21)	0.78947(15/19)
Bo_TE_212511	0.23256(10/43)	0.70588(12/17)	0.40000(8/20)	0.10000(2/20)
Bo_TE_3050	0.00000(0/42)	0.00000(0/16)	0.33333(6/18)	0.61111(11/18)
Bo_TE_27470	0.70000(28/40)	0.00000(0/18)	0.00000(0/11)	0.00000(0/16)
Bo_TE_223937	0.86667(39/45)	0.31250(5/16)	0.70000(14/20)	1.00000(20/20)
Bo_TE_26953	0.74359(29/39)	0.33333(5/15)	0.40909(9/22)	0.10526(2/19)
Bo_TE_51465	0.55814(24/43)	0.16667(3/18)	0.00000(0/22)	0.00000(0/20)
Bo_TE_198184	0.61905(26/42)	0.37500(6/16)	0.72222(13/18)	0.00000(0/20)
Bo_TE_10583	0.67442(29/43)	0.38889(7/18)	0.00000(0/23)	0.00000(0/18)
Bo_TE_122539	0.94872(37/39)	0.64286(9/14)	0.66667(14/21)	0.41176(7/17)
Bo_TE_69135	1.00000(42/42)	1.00000(18/18)	0.40909(9/22)	0.95000(19/20)
Bo_TE_110184	0.02273(1/44)	0.15789(3/19)	0.57143(12/21)	0.00000(0/20)
Bo_TE_174511	0.37209(16/43)	0.05882(1/17)	0.04762(1/21)	0.75000(15/20)
Bo_TE_227995	0.00000(0/45)	0.00000(0/19)	0.80000(16/20)	0.38889(7/18)
Bo_TE_158105	0.80952(34/42)	0.29412(5/17)	0.19048(4/21)	0.26316(5/19)
Bo_TE_234954	0.59091(26/44)	0.27778(5/18)	0.00000(0/21)	0.30000(6/20)

Bo_TE_44642	0.11364(5/44)	0.64706(11/17)	0.90476(19/21)	0.22222(4/18)
Bo_TE_86172	0.02174(1/46)	0.00000(0/18)	0.61905(13/21)	0.80000(16/20)
Bo_TE_7478	0.18605(8/43)	0.58824(10/17)	0.00000(0/21)	0.35000(7/20)
Bo_TE_58850	0.17647(6/34)	0.64706(11/17)	0.00000(0/19)	0.00000(0/9)
Bo_TE_104604	0.39024(16/41)	0.11765(2/17)	0.04762(1/21)	0.80000(16/20)
Bo_TE_237483	0.09091(4/44)	0.00000(0/19)	0.60000(12/20)	0.57895(11/19)
Bo_TE_44997	0.51220(21/41)	0.11111(2/18)	0.59091(13/22)	0.66667(12/18)
Bo_TE_81911	0.02222(1/45)	0.47059(8/17)	0.54545(12/22)	0.85000(17/20)
Bo_TE_7980	0.04651(2/43)	0.00000(0/17)	0.63636(14/22)	0.29412(5/17)
Bo_TE_100198	0.20000(9/45)	0.00000(0/18)	0.61905(13/21)	0.47368(9/19)
Bo_TE_144191	0.04444(2/45)	0.77778(14/18)	0.00000(0/22)	0.10000(2/20)
Bo_TE_6619	0.21429(9/42)	0.06250(1/16)	0.70000(14/20)	0.05263(1/19)
Bo_TE_7993	0.04545(2/44)	0.00000(0/18)	0.52381(11/21)	0.60000(12/20)
Bo_TE_158079	0.04348(2/46)	0.00000(0/18)	0.50000(11/22)	0.15789(3/19)
Bo_TE_226331	0.28571(12/42)	0.78947(15/19)	0.00000(0/22)	0.05556(1/18)
Bo_TE_4796	0.07500(3/40)	0.12500(2/16)	0.80952(17/21)	0.57895(11/19)
Bo_TE_149284	0.00000(0/46)	0.05263(1/19)	0.80000(16/20)	0.05000(1/20)
Bo_TE_18294	0.00000(0/43)	0.05882(1/17)	0.04348(1/23)	0.70000(14/20)
Bo_TE_137856	0.00000(0/45)	0.52941(9/17)	0.08696(2/23)	0.10000(2/20)
Bo_TE_50381	0.02174(1/46)	0.38889(7/18)	0.08696(2/23)	0.52632(10/19)
Bo_TE_21616	0.43902(18/41)	0.82353(14/17)	0.08696(2/23)	0.50000(10/20)
Bo_TE_93413	0.07143(3/42)	0.58824(10/17)	0.00000(0/23)	0.61111(11/18)
Bo_TE_129103	0.63415(26/41)	0.16667(3/18)	0.86364(19/22)	0.80000(16/20)
Bo_TE_199603	0.72222(26/36)	0.82353(14/17)	0.36364(8/22)	0.10526(2/19)
Bo_TE_214538	0.73171(30/41)	0.26667(4/15)	0.22727(5/22)	0.36842(7/19)
Bo_TE_159220	0.54054(20/37)	0.25000(4/16)	0.00000(0/19)	0.00000(0/15)
Bo_TE_57326	0.00000(0/44)	0.00000(0/19)	0.23810(5/21)	0.61111(11/18)
Bo_TE_112729	0.60465(26/43)	0.00000(0/16)	0.00000(0/22)	0.00000(0/20)
Bo_TE_52362	0.95556(43/45)	0.38889(7/18)	0.90909(20/22)	0.77778(14/18)
Bo_TE_27427	0.76190(32/42)	0.87500(14/16)	0.43478(10/23)	0.05000(1/20)
Bo_TE_125960	0.63415(26/41)	0.13333(2/15)	0.00000(0/23)	0.05000(1/20)
Bo_TE_41412	0.00000(0/46)	0.00000(0/19)	0.52381(11/21)	0.00000(0/20)
Bo_TE_111030	0.27907(12/43)	0.27778(5/18)	0.90909(20/22)	0.10526(2/19)
Bo_TE_137614	0.25000(11/44)	0.11765(2/17)	0.19048(4/21)	0.68421(13/19)
Bo_TE_91236	0.82500(33/40)	0.93750(15/16)	0.61905(13/21)	0.26316(5/19)
Bo_TE_12726	0.83721(36/43)	0.05882(1/17)	0.18182(4/22)	0.05556(1/18)
Bo_TE_87422	0.76744(33/43)	0.31579(6/19)	0.25000(5/20)	0.85000(17/20)
Bo_TE_130143	0.06818(3/44)	0.00000(0/16)	0.60870(14/23)	0.89474(17/19)
Bo_TE_208221	0.68889(31/45)	0.53333(8/15)	0.00000(0/22)	0.10000(2/20)
Bo_TE_183014	0.52273(23/44)	0.83333(15/18)	0.36364(8/22)	0.10526(2/19)
Bo_TE_179790	0.25581(11/43)	0.41176(7/17)	0.35000(7/20)	0.78947(15/19)
Bo_TE_58929	0.57143(24/42)	0.11765(2/17)	0.00000(0/21)	0.30000(6/20)
Bo_TE_133170	0.39024(16/41)	0.94444(17/18)	0.75000(15/20)	0.88889(16/18)
Bo_TE_18721	0.26190(11/42)	0.05263(1/19)	0.00000(0/23)	0.57895(11/19)
Bo_TE_54627	0.48718(19/39)	0.41176(7/17)	0.95455(21/22)	0.61111(11/18)
Bo_TE_214317	0.71053(27/38)	0.17647(3/17)	0.30435(7/23)	0.36842(7/19)
Bo_TE_45822	0.86364(38/44)	1.00000(18/18)	1.00000(22/22)	0.35000(7/20)
Bo_TE_68984	0.71111(32/45)	0.41176(7/17)	0.91304(21/23)	0.66667(12/18)
Bo_TE_89821	0.33333(14/42)	0.88235(15/17)	0.42857(9/21)	0.36842(7/19)
Bo_TE_71130	0.57500(23/40)	0.17647(3/17)	0.00000(0/22)	0.57895(11/19)
Bo_TE_155149	0.90698(39/43)	0.83333(15/18)	0.73913(17/23)	0.30000(6/20)
Bo_TE_196571	0.00000(0/44)	0.66667(12/18)	0.04545(1/22)	0.84211(16/19)
Bo_TE_101585	0.93182(41/44)	0.43750(7/16)	0.23810(5/21)	0.66667(12/18)
Bo_TE_219754	0.07317(3/41)	0.52941(9/17)	0.61111(11/18)	0.57895(11/19)
Bo_TE_22835	0.11111(5/45)	0.52941(9/17)	0.00000(0/20)	0.05000(1/20)
Bo_TE_196506	0.21429(9/42)	0.37500(6/16)	1.00000(23/23)	0.94737(18/19)

Bo_TE_42343	0.90698(39/43)	0.47059(8/17)	1.00000(23/23)	0.80000(16/20)
Bo_TE_216622	0.20930(9/43)	0.70588(12/17)	0.00000(0/23)	0.05000(1/20)
Bo_TE_36200	1.00000(45/45)	1.00000(18/18)	0.27273(6/22)	1.00000(20/20)
Bo_TE_37053	0.40541(15/37)	0.17647(3/17)	1.00000(21/21)	0.65000(13/20)
Bo_TE_129094	0.58140(25/43)	0.11765(2/17)	0.10000(2/20)	0.05000(1/20)
Bo_TE_50489	0.90476(38/42)	0.70588(12/17)	0.13043(3/23)	0.10526(2/19)
Bo_TE_121786	0.59091(26/44)	1.00000(18/18)	0.95652(22/23)	0.15000(3/20)
Bo_TE_42141	0.16279(7/43)	0.33333(6/18)	0.33333(7/21)	0.85000(17/20)
Bo_TE_170698	0.46341(19/41)	0.00000(0/17)	0.00000(0/22)	0.70000(14/20)
Bo_TE_227118	0.97727(43/44)	0.75000(12/16)	1.00000(21/21)	0.15789(3/19)
Bo_TE_178888	0.25000(11/44)	0.00000(0/17)	0.31818(7/22)	0.75000(15/20)
Bo_TE_119482	0.83333(35/42)	1.00000(18/18)	0.38095(8/21)	0.85000(17/20)
Bo_TE_132403	0.58140(25/43)	0.18750(3/16)	0.81818(18/22)	0.11111(2/18)
Bo_TE_198544	0.88095(37/42)	0.83333(15/18)	0.65000(13/20)	0.05263(1/19)
Bo_TE_25380	0.41026(16/39)	0.55556(10/18)	0.81818(18/22)	1.00000(17/17)
Bo_TE_128730	0.77500(31/40)	0.29412(5/17)	0.42105(8/19)	0.94737(18/19)
Bo_TE_200345	0.60000(24/40)	0.22222(4/18)	0.00000(0/22)	0.25000(5/20)
Bo_TE_89442	0.60000(24/40)	0.43750(7/16)	0.10000(2/20)	0.00000(0/18)
Bo_TE_59770	0.80000(36/45)	0.27778(5/18)	0.77273(17/22)	0.20000(4/20)
Bo_TE_221279	0.00000(0/46)	0.76471(13/17)	0.00000(0/22)	0.05000(1/20)
Bo_TE_83551	0.62500(25/40)	0.50000(8/16)	0.44444(8/18)	0.00000(0/19)
Bo_TE_222695	0.97727(43/44)	0.70588(12/17)	0.55556(10/18)	0.25000(5/20)
Bo_TE_27031	0.75556(34/45)	0.43750(7/16)	0.47619(10/21)	0.95000(19/20)
Bo_TE_20164	0.18182(8/44)	0.94118(16/17)	0.57895(11/19)	0.89474(17/19)
Bo_TE_6010	0.30769(12/39)	0.66667(12/18)	1.00000(20/20)	1.00000(18/18)
Bo_TE_224712	0.73810(31/42)	0.05556(1/18)	0.00000(0/23)	0.00000(0/19)
Bo_TE_89398	1.00000(42/42)	1.00000(19/19)	0.25000(5/20)	1.00000(19/19)
Bo_TE_97541	0.62162(23/37)	0.77778(14/18)	0.04545(1/22)	0.38889(7/18)
Bo_TE_193772	0.74419(32/43)	0.25000(4/16)	0.52381(11/21)	0.85000(17/20)
Bo_TE_92737	0.12821(5/39)	0.00000(0/18)	0.54545(12/22)	0.00000(0/17)
Bo_TE_121591	0.22727(10/44)	0.88235(15/17)	0.66667(14/21)	0.77778(14/18)
Bo_TE_51145	0.00000(0/45)	0.00000(0/19)	0.27273(6/22)	0.63158(12/19)
Bo_TE_6730	0.10000(4/40)	0.40000(6/15)	0.30435(7/23)	0.83333(15/18)
Bo_TE_104808	0.44444(20/45)	0.29412(5/17)	0.95238(20/21)	0.15789(3/19)
Bo_TE_191758	0.63415(26/41)	0.88235(15/17)	0.00000(0/21)	0.15000(3/20)
Bo_TE_206657	0.61905(26/42)	0.11111(2/18)	0.23810(5/21)	0.35000(7/20)
Bo_TE_9821	0.06667(3/45)	0.56250(9/16)	0.04762(1/21)	0.00000(0/18)
Bo_TE_153986	0.47368(18/38)	0.77778(14/18)	0.04545(1/22)	0.50000(10/20)
Bo_TE_130497	0.95349(41/43)	0.77778(14/18)	0.14286(3/21)	0.31579(6/19)
Bo_TE_204473	0.38095(16/42)	0.56250(9/16)	0.86667(13/15)	0.05556(1/18)
Bo_TE_235312	0.88889(40/45)	0.72222(13/18)	0.31818(7/22)	0.85000(17/20)
Bo_TE_4423	0.08889(4/45)	0.75000(12/16)	0.45455(10/22)	0.20000(4/20)
Bo_TE_220103	0.00000(0/43)	0.10526(2/19)	0.76190(16/21)	0.64706(11/17)
Bo_TE_41710	0.58140(25/43)	0.82353(14/17)	0.30435(7/23)	0.73684(14/19)
Bo_TE_91815	0.00000(0/45)	0.83333(15/18)	1.00000(1/1)	0.00000(0/1)
Bo_TE_133091	0.02174(1/46)	0.00000(0/18)	0.80952(17/21)	0.22222(4/18)
Bo_TE_102824	0.73171(30/41)	0.18750(3/16)	0.00000(0/23)	0.68421(13/19)
Bo_TE_102222	0.93182(41/44)	0.94118(16/17)	0.73684(14/19)	0.22222(4/18)
Bo_TE_154482	0.00000(0/46)	0.82353(14/17)	0.00000(0/23)	0.05000(1/20)
Bo_TE_8741	0.11905(5/42)	0.94737(18/19)	0.20000(4/20)	0.58824(10/17)
Bo_TE_74444	0.00000(0/46)	0.05263(1/19)	0.21739(5/23)	0.61111(11/18)
Bo_TE_136209	0.53846(21/39)	0.18750(3/16)	0.70000(14/20)	0.05000(1/20)
Bo_TE_58053	0.08889(4/45)	0.70588(12/17)	0.40909(9/22)	0.05263(1/19)
Bo_TE_165690	1.00000(42/42)	0.81250(13/16)	0.22727(5/22)	0.27778(5/18)
Bo_TE_2816	0.80952(34/42)	0.60000(9/15)	0.04545(1/22)	0.00000(0/17)
Bo_TE_205032	0.48780(20/41)	0.83333(15/18)	0.61905(13/21)	0.05000(1/20)

Bo_TE_227925	0.83721(36/43)	0.53333(8/15)	0.18182(4/22)	0.66667(12/18)
Bo_TE_15429	0.26667(12/45)	1.00000(12/12)	1.00000(23/23)	0.45000(9/20)
Bo_TE_210728	0.53333(24/45)	0.16667(2/12)	0.00000(0/23)	0.35294(6/17)
Bo_TE_230941	0.86364(38/44)	0.00000(0/17)	0.00000(0/21)	0.22222(4/18)
Bo_TE_56819	0.54762(23/42)	0.50000(9/18)	0.80000(16/20)	0.10000(2/20)
Bo_TE_132343	0.43902(18/41)	0.62500(10/16)	0.47619(10/21)	1.00000(17/17)
Bo_TE_220035	0.93333(42/45)	0.70588(12/17)	0.43478(10/23)	0.10526(2/19)
Bo_TE_55235	0.45455(20/44)	0.81250(13/16)	1.00000(21/21)	0.77778(14/18)
Bo_TE_108514	0.32558(14/43)	0.82353(14/17)	0.04545(1/22)	0.00000(0/19)
Bo_TE_186248	0.64444(29/45)	0.88889(16/18)	0.95652(22/23)	0.11765(2/17)
Bo_TE_117486	0.04545(2/44)	0.81250(13/16)	0.50000(10/20)	0.44444(8/18)
Bo_TE_93378	0.32558(14/43)	0.33333(5/15)	0.00000(0/21)	0.52632(10/19)
Bo_TE_229902	0.51163(22/43)	0.81250(13/16)	0.15000(3/20)	1.00000(16/16)
Bo_TE_100006	0.45238(19/42)	0.76471(13/17)	0.28571(6/21)	0.10526(2/19)
Bo_TE_190866	0.62500(25/40)	0.11765(2/17)	0.04762(1/21)	0.00000(0/19)
Bo_TE_220010	0.02326(1/43)	0.20000(3/15)	0.52632(10/19)	0.10526(2/19)
Bo_TE_19736	0.00000(0/45)	0.81250(13/16)	0.42857(9/21)	0.10000(2/20)
Bo_TE_200114	0.07143(3/42)	0.83333(15/18)	0.80952(17/21)	0.52632(10/19)
Bo_TE_209914	0.37838(14/37)	0.00000(0/17)	0.70000(14/20)	1.00000(5/5)
Bo_TE_207225	0.04545(2/44)	0.00000(0/17)	0.28571(6/21)	0.57895(11/19)
Bo_TE_89553	0.97619(41/42)	1.00000(18/18)	0.47619(10/21)	1.00000(20/20)
Bo_TE_160744	0.02326(1/43)	0.00000(0/19)	0.72727(16/22)	0.47368(9/19)
Bo_TE_43761	0.79070(34/43)	0.05556(1/18)	0.40909(9/22)	0.68421(13/19)
Bo_TE_104770	0.77778(35/45)	0.47059(8/17)	1.00000(22/22)	1.00000(20/20)
Bo_TE_8686	0.30233(13/43)	0.87500(14/16)	0.33333(7/21)	0.05000(1/20)
Bo_TE_97704	0.77273(34/44)	0.05556(1/18)	0.00000(0/23)	0.05263(1/19)
Bo_TE_180214	0.52500(21/40)	0.00000(0/1)	0.00000(0/21)	0.00000(0/16)
Bo_TE_232252	0.00000(0/44)	0.64706(11/17)	0.42857(9/21)	0.52632(10/19)
Bo_TE_201931	0.81818(36/44)	0.38889(7/18)	0.25000(5/20)	0.15000(3/20)
Bo_TE_198459	0.88636(39/44)	0.72222(13/18)	0.61905(13/21)	0.05263(1/19)
Bo_TE_59060	0.86364(38/44)	0.33333(6/18)	0.38095(8/21)	0.50000(10/20)
Bo_TE_46097	0.70732(29/41)	0.53333(8/15)	0.13636(3/22)	0.00000(0/20)
Bo_TE_169451	0.57500(23/40)	0.15789(3/19)	0.04545(1/22)	0.25000(5/20)
Bo_TE_70471	0.90909(40/44)	0.22222(4/18)	0.77273(17/22)	0.66667(12/18)
Bo_TE_119537	0.67442(29/43)	1.00000(17/17)	0.23810(5/21)	0.63158(12/19)
Bo_TE_91542	0.37209(16/43)	0.94118(16/17)	0.95652(22/23)	0.88235(15/17)
Bo_TE_127403	0.30000(12/40)	0.11765(2/17)	1.00000(20/20)	0.68421(13/19)
Bo_TE_123324	0.00000(0/46)	0.00000(0/17)	0.80000(16/20)	0.26316(5/19)
Bo_TE_46724	0.41860(18/43)	0.00000(0/18)	0.60000(12/20)	0.05000(1/20)
Bo_TE_32987	0.76316(29/38)	0.50000(8/16)	0.18182(4/22)	0.61111(11/18)
Bo_TE_204995	0.23810(10/42)	0.12500(2/16)	0.38095(8/21)	0.95000(19/20)
Bo_TE_209206	0.56098(23/41)	0.52941(9/17)	0.36364(8/22)	0.00000(0/20)
Bo_TE_42667	0.13514(5/37)	0.68750(11/16)	0.47619(10/21)	0.27778(5/18)
Bo_TE_121394	0.13514(5/37)	0.81250(13/16)	0.04545(1/22)	0.00000(0/17)
Bo_TE_232184	0.00000(0/46)	0.25000(4/16)	0.36364(8/22)	0.50000(10/20)
Bo_TE_18040	0.95122(39/41)	0.87500(14/16)	0.16667(2/12)	0.22222(4/18)
Bo_TE_169588	0.68182(30/44)	0.23529(4/17)	0.57143(12/21)	0.73684(14/19)
Bo_TE_211438	0.23256(10/43)	0.25000(4/16)	0.50000(10/20)	0.00000(0/20)
Bo_TE_160539	0.88372(38/43)	0.75000(12/16)	0.33333(7/21)	0.85000(17/20)
Bo_TE_2729	0.65854(27/41)	0.00000(0/17)	0.21739(5/23)	0.47368(9/19)
Bo_TE_133727	0.06522(3/46)	0.05263(1/19)	0.19048(4/21)	0.75000(15/20)
Bo_TE_48340	0.84783(39/46)	0.33333(6/18)	0.60000(12/20)	0.38889(7/18)
Bo_TE_78624	0.78571(33/42)	0.15789(3/19)	0.28571(6/21)	0.05000(1/20)
Bo_TE_34429	0.83721(36/43)	0.70588(12/17)	0.86364(19/22)	0.11111(2/18)
Bo_TE_211898	0.02273(1/44)	0.05882(1/17)	0.25000(5/20)	0.68421(13/19)
Bo_TE_42870	0.72727(32/44)	0.47059(8/17)	1.00000(21/21)	1.00000(20/20)

Bo_TE_138824	0.83333(30/36)	0.26316(5/19)	0.33333(7/21)	0.80000(16/20)
Bo_TE_110901	0.05000(2/40)	0.00000(0/16)	0.00000(0/19)	0.56250(9/16)
Bo_TE_170679	0.56098(23/41)	0.11765(2/17)	0.00000(0/21)	0.70000(14/20)
Bo_TE_104602	0.00000(0/45)	0.00000(0/18)	0.04348(1/23)	0.50000(8/16)
Bo_TE_142247	0.73171(30/41)	0.31250(5/16)	0.70000(14/20)	0.84211(16/19)
Bo_TE_91641	0.93182(41/44)	0.76471(13/17)	0.33333(7/21)	0.85000(17/20)
Bo_TE_120793	0.41026(16/39)	0.66667(12/18)	0.90909(20/22)	1.00000(19/19)
Bo_TE_25076	0.00000(0/36)	0.17647(3/17)	0.05263(1/19)	0.85000(17/20)
Bo_TE_175078	0.21951(9/41)	0.62500(10/16)	0.55000(11/20)	0.80000(16/20)
Bo_TE_147907	0.22727(10/44)	0.47059(8/17)	0.95652(22/23)	0.90000(18/20)
Bo_TE_163780	0.92683(38/41)	0.70000(7/10)	0.93750(15/16)	0.21053(4/19)
Bo_TE_224781	0.75000(33/44)	0.15789(3/19)	0.86364(19/22)	0.80000(16/20)
Bo_TE_43675	0.17778(8/45)	0.35294(6/17)	0.95000(19/20)	0.55556(10/18)
Bo_TE_230701	0.11628(5/43)	0.00000(0/16)	0.09524(2/21)	0.77778(14/18)
Bo_TE_42913	0.00000(0/44)	0.50000(9/18)	0.04348(1/23)	0.31579(6/19)
Bo_TE_141802	0.63415(26/41)	0.73684(14/19)	0.00000(0/23)	0.00000(0/20)
Bo_TE_215594	0.04444(2/45)	0.50000(8/16)	0.66667(14/21)	0.55000(11/20)
Bo_TE_212015	0.12195(5/41)	0.00000(0/18)	0.76190(16/21)	0.25000(4/16)
Bo_TE_227259	0.24390(10/41)	0.06667(1/15)	0.66667(14/21)	0.10526(2/19)
Bo_TE_113793	0.59375(19/32)	1.00000(19/19)	0.93750(15/16)	0.25000(4/16)
Bo_TE_156542	0.19565(9/46)	0.50000(9/18)	0.25000(5/20)	0.00000(0/20)
Bo_TE_109893	0.62791(27/43)	0.76471(13/17)	0.55000(11/20)	0.00000(0/20)
Bo_TE_42390	0.44737(17/38)	0.68750(11/16)	0.76190(16/21)	0.22222(4/18)
Bo_TE_161220	0.60976(25/41)	0.58824(10/17)	0.00000(0/22)	0.31579(6/19)
Bo_TE_155556	0.25000(10/40)	0.00000(0/17)	0.76190(16/21)	0.52632(10/19)
Bo_TE_162175	0.61905(26/42)	0.06250(1/16)	0.35000(7/20)	0.85000(17/20)
Bo_TE_24929	0.57895(22/38)	0.22222(4/18)	0.08696(2/23)	0.00000(0/19)
Bo_TE_110826	0.32432(12/37)	0.73684(14/19)	0.84211(16/19)	0.45000(9/20)
Bo_TE_226986	0.47368(18/38)	0.58824(10/17)	1.00000(23/23)	0.85000(17/20)
Bo_TE_136115	0.93023(40/43)	1.00000(18/18)	0.68421(13/19)	0.35000(7/20)
Bo_TE_165708	0.00000(0/44)	0.00000(0/18)	0.00000(0/22)	0.60000(12/20)
Bo_TE_182255	1.00000(44/44)	0.47368(9/19)	0.42857(9/21)	0.55000(11/20)
Bo_TE_106357	0.13043(6/46)	0.00000(0/17)	0.59091(13/22)	0.33333(6/18)
Bo_TE_209972	0.59524(25/42)	0.05556(1/18)	0.00000(0/22)	0.00000(0/20)
Bo_TE_1208	0.42500(17/40)	0.05882(1/17)	0.50000(10/20)	0.00000(0/20)
Bo_TE_121307	0.27500(11/40)	0.15789(3/19)	0.57143(12/21)	0.05556(1/18)
Bo_TE_130798	0.61905(26/42)	0.11111(2/18)	0.26667(4/15)	0.27778(5/18)
Bo_TE_70423	0.80952(34/42)	0.33333(6/18)	0.00000(0/23)	0.00000(0/20)
Bo_TE_6102	0.82500(33/40)	0.82353(14/17)	0.23810(5/21)	0.21053(4/19)
Bo_TE_27449	0.77500(31/40)	0.00000(0/19)	0.00000(0/23)	0.00000(0/20)
Bo_TE_154486	0.95349(41/43)	0.17647(3/17)	0.20000(4/20)	0.15789(3/19)
Bo_TE_216544	0.06977(3/43)	0.00000(0/19)	0.61905(13/21)	0.00000(0/18)
Bo_TE_212521	0.65854(27/41)	0.27778(5/18)	0.19048(4/21)	0.90000(18/20)
Bo_TE_27495	0.00000(0/45)	0.00000(0/19)	0.57143(12/21)	0.00000(0/20)
Bo_TE_149656	0.76923(30/39)	0.94118(16/17)	0.23810(5/21)	0.26316(5/19)
Bo_TE_63300	0.83721(36/43)	0.23529(4/17)	0.45455(10/22)	0.30000(6/20)
Bo_TE_66253	0.63636(28/44)	0.06250(1/16)	0.28571(6/21)	0.41176(7/17)
Bo_TE_222158	0.00000(0/42)	0.63158(12/19)	0.04545(1/22)	0.05000(1/20)
Bo_TE_191759	0.40000(16/40)	0.12500(2/16)	1.00000(22/22)	0.85000(17/20)
Bo_TE_130815	0.61905(26/42)	0.00000(0/16)	0.19048(4/21)	0.21053(4/19)
Bo_TE_107299	0.35000(14/40)	0.82353(14/17)	0.21739(5/23)	0.75000(15/20)
Bo_TE_53359	0.51220(21/41)	0.16667(3/18)	0.91304(21/23)	0.58824(10/17)
Bo_TE_10228	0.58140(25/43)	0.41176(7/17)	0.31818(7/22)	0.00000(0/19)
Bo_TE_235295	0.02381(1/42)	0.00000(0/18)	0.54545(12/22)	0.00000(0/18)
Bo_TE_49773	0.14286(6/42)	0.75000(12/16)	0.15789(3/19)	0.06667(1/15)
Bo_TE_39634	0.29268(12/41)	0.13333(2/15)	0.15000(3/20)	0.88889(16/18)

Bo_TE_31146	0.51163(22/43)	0.00000(0/16)	0.00000(0/21)	0.16667(3/18)
Bo_TE_87418	0.40476(17/42)	0.58824(10/17)	0.04545(1/22)	0.11111(2/18)
Bo_TE_96079	0.47727(21/44)	0.82353(14/17)	0.60000(12/20)	0.00000(0/20)
Bo_TE_11749	0.34211(13/38)	0.62500(10/16)	1.00000(22/22)	1.00000(20/20)
Bo_TE_222558	0.52381(22/42)	0.00000(0/19)	0.04348(1/23)	0.00000(0/19)
Bo_TE_96872	0.00000(0/44)	0.00000(0/18)	0.80000(16/20)	0.44444(8/18)
Bo_TE_73030	0.04762(2/42)	0.61111(11/18)	0.42857(9/21)	0.57895(11/19)
Bo_TE_56220	0.20513(8/39)	0.26667(4/15)	0.76190(16/21)	0.94737(18/19)
Bo_TE_94670	0.79070(34/43)	0.50000(9/18)	0.00000(0/23)	0.73684(14/19)
Bo_TE_39314	0.51163(22/43)	0.20000(3/15)	0.00000(0/22)	0.05263(1/19)
Bo_TE_230812	0.41026(16/39)	0.82353(14/17)	0.70000(14/20)	0.94737(18/19)
Bo_TE_117218	0.61905(26/42)	0.17647(3/17)	0.00000(0/22)	0.52632(10/19)
Bo_TE_3829	0.74419(32/43)	0.06667(1/15)	0.57143(12/21)	0.00000(0/20)
Bo_TE_182824	0.36364(16/44)	0.52941(9/17)	0.81818(18/22)	0.95000(19/20)
Bo_TE_7876	0.90244(37/41)	0.18750(3/16)	0.75000(15/20)	0.47368(9/19)
Bo_TE_212850	0.50000(22/44)	0.00000(0/19)	0.00000(0/23)	0.05263(1/19)
Bo_TE_112350	0.34091(15/44)	0.87500(14/16)	0.14286(3/21)	0.95000(19/20)
Bo_TE_100129	0.14634(6/41)	0.00000(0/17)	0.68182(15/22)	0.52941(9/17)
Bo_TE_27266	0.40000(16/40)	0.55556(10/18)	0.85714(18/21)	0.20000(4/20)
Bo_TE_76073	0.60465(26/43)	0.00000(0/16)	0.00000(0/18)	0.15385(2/13)
Bo_TE_236235	0.04651(2/43)	0.57895(11/19)	0.09091(2/22)	0.15000(3/20)
Bo_TE_113356	0.00000(0/46)	0.16667(3/18)	0.00000(0/23)	0.85000(17/20)
Bo_TE_119601	0.47368(18/38)	1.00000(17/17)	0.41176(7/17)	0.75000(12/16)
Bo_TE_144911	0.72727(32/44)	0.05556(1/18)	0.00000(0/22)	0.00000(0/20)
Bo_TE_167786	0.91176(31/34)	0.88235(15/17)	0.38095(8/21)	1.00000(15/15)
Bo_TE_141763	1.00000(44/44)	1.00000(18/18)	0.19048(4/21)	0.85000(17/20)
Bo_TE_138402	0.86842(33/38)	0.94737(18/19)	0.84211(16/19)	0.27778(5/18)
Bo_TE_190717	0.55000(22/40)	0.35294(6/17)	0.04545(1/22)	0.05556(1/18)
Bo_TE_75356	0.50000(21/42)	0.00000(0/19)	0.00000(0/22)	0.15789(3/19)
Bo_TE_158412	0.81395(35/43)	0.47059(8/17)	1.00000(20/20)	0.94737(18/19)
Bo_TE_155592	0.21429(9/42)	0.52941(9/17)	0.75000(15/20)	0.50000(9/18)
Bo_TE_49994	0.81818(36/44)	0.94444(17/18)	1.00000(21/21)	0.31579(6/19)
Bo_TE_202634	0.95349(41/43)	0.61111(11/18)	0.77273(17/22)	0.31579(6/19)
Bo_TE_23211	0.70732(29/41)	0.12500(2/16)	0.85000(17/20)	0.73684(14/19)
Bo_TE_240641	0.15385(6/39)	0.81250(13/16)	0.95652(22/23)	0.35000(7/20)
Bo_TE_209321	0.38636(17/44)	0.55556(10/18)	0.00000(0/23)	0.00000(0/19)
Bo_TE_209621	0.72093(31/43)	0.17647(3/17)	0.19048(4/21)	0.33333(6/18)
Bo_TE_131743	0.37209(16/43)	0.52941(9/17)	0.00000(0/19)	0.05263(1/19)
Bo_TE_95288	0.78378(29/37)	0.00000(0/14)	0.45000(9/20)	0.16667(3/18)
Bo_TE_143080	0.00000(0/40)	0.31579(6/19)	0.52632(10/19)	0.15000(3/20)
Bo_TE_150986	0.93023(40/43)	0.70588(12/17)	0.18182(4/22)	0.89474(17/19)
Bo_TE_182342	0.02273(1/44)	0.11111(2/18)	0.40000(8/20)	0.52632(10/19)
Bo_TE_183901	0.71795(28/39)	0.29412(5/17)	0.00000(0/22)	0.00000(0/20)
Bo_TE_111380	0.82051(32/39)	0.05263(1/19)	0.00000(0/23)	0.15000(3/20)
Bo_TE_9941	0.39535(17/43)	0.88235(15/17)	0.26087(6/23)	0.00000(0/19)
Bo_TE_50328	0.95455(42/44)	0.62500(10/16)	0.50000(10/20)	0.11111(2/18)
Bo_TE_220855	0.37209(16/43)	0.18750(3/16)	0.57895(11/19)	0.94737(18/19)
Bo_TE_45425	0.59524(25/42)	0.00000(0/17)	0.22727(5/22)	0.00000(0/20)
Bo_TE_54532	0.45238(19/42)	0.43750(7/16)	0.31579(6/19)	0.95000(19/20)
Bo_TE_182340	0.06522(3/46)	0.52941(9/17)	0.59091(13/22)	0.45000(9/20)
Bo_TE_28106	0.65217(30/46)	0.00000(0/18)	0.71429(15/21)	0.50000(9/18)
Bo_TE_201650	0.93333(42/45)	0.94444(17/18)	0.39130(9/23)	0.80000(16/20)
Bo_TE_49599	0.37500(15/40)	0.94444(17/18)	0.18182(4/22)	0.38889(7/18)
Bo_TE_111964	0.45455(20/44)	0.17647(3/17)	0.77273(17/22)	0.05263(1/19)
Bo_TE_177979	0.95238(40/42)	0.66667(4/6)	0.15789(3/19)	0.44444(8/18)
Bo_TE_106080	0.00000(0/46)	0.52632(10/19)	0.00000(0/22)	0.00000(0/20)

Bo_TE_26287	0.04762(2/42)	0.68750(11/16)	0.33333(6/18)	0.21053(4/19)
Bo_TE_149246	1.00000(42/42)	0.52941(9/17)	0.80000(16/20)	0.42105(8/19)
Bo_TE_18159	0.11628(5/43)	0.20000(3/15)	1.00000(22/22)	0.20000(4/20)
Bo_TE_179739	0.60465(26/43)	0.47059(8/17)	0.86364(19/22)	0.00000(0/18)
Bo_TE_1510	0.72093(31/43)	0.05882(1/17)	0.00000(0/22)	0.00000(0/20)
Bo_TE_76722	0.16667(7/42)	0.88235(15/17)	1.00000(21/21)	1.00000(11/11)
Bo_TE_219821	0.43902(18/41)	0.89474(17/19)	0.76190(16/21)	1.00000(19/19)
Bo_TE_97750	0.27907(12/43)	1.00000(19/19)	1.00000(23/23)	0.94737(18/19)
Bo_TE_93845	0.00000(0/44)	0.00000(0/19)	0.04545(1/22)	0.85000(17/20)
Bo_TE_215442	0.28889(13/45)	0.11111(2/18)	0.17391(4/23)	0.84211(16/19)
Bo_TE_236757	0.00000(0/44)	0.00000(0/19)	0.60000(12/20)	0.70588(12/17)
Bo_TE_119534	0.23810(10/42)	0.00000(0/17)	0.76190(16/21)	0.36842(7/19)
Bo_TE_12455	0.85000(34/40)	0.41176(7/17)	0.00000(0/23)	0.00000(0/20)
Bo_TE_33142	0.00000(0/44)	0.35294(6/17)	0.30000(6/20)	0.84211(16/19)
Bo_TE_197950	0.51163(22/43)	0.00000(0/18)	0.68421(13/19)	0.78947(15/19)
Bo_TE_209635	0.12195(5/41)	0.05556(1/18)	0.77273(17/22)	0.85000(17/20)
Bo_TE_179988	0.00000(0/40)	0.00000(0/18)	0.52381(11/21)	0.00000(0/20)
Bo_TE_81888	0.85714(36/42)	0.05882(1/17)	0.13636(3/22)	0.15789(3/19)
Bo_TE_103192	0.87500(35/40)	0.29412(5/17)	0.57143(12/21)	0.00000(0/19)
Bo_TE_25681	0.35000(14/40)	0.05556(1/18)	0.04348(1/23)	0.88889(16/18)
Bo_TE_148561	0.02326(1/43)	0.17647(3/17)	0.00000(0/21)	0.50000(8/16)
Bo_TE_167851	0.34091(15/44)	0.16667(3/18)	1.00000(22/22)	0.55556(10/18)
Bo_TE_83719	0.57500(23/40)	0.88889(16/18)	0.26316(5/19)	0.47368(9/19)
Bo_TE_7039	0.59524(25/42)	0.31250(5/16)	0.14286(3/21)	0.05263(1/19)
Bo_TE_134089	0.54545(24/44)	0.00000(0/17)	0.00000(0/22)	0.18750(3/16)
Bo_TE_36501	0.97674(42/43)	0.44444(8/18)	0.47619(10/21)	0.26316(5/19)
Bo_TE_151516	0.60976(25/41)	0.82353(14/17)	0.26316(5/19)	0.42105(8/19)
Bo_TE_239712	0.21429(9/42)	0.12500(2/16)	0.00000(0/23)	0.88889(16/18)
Bo_TE_49003	0.13636(6/44)	0.73333(11/15)	0.85714(18/21)	0.64706(11/17)
Bo_TE_141785	1.00000(41/41)	1.00000(18/18)	0.18182(4/22)	0.85000(17/20)
Bo_TE_133690	0.34884(15/43)	0.11765(2/17)	0.80952(17/21)	0.70000(14/20)
Bo_TE_181370	0.26087(12/46)	0.56250(9/16)	1.00000(23/23)	0.65000(13/20)
Bo_TE_170994	0.42857(18/42)	0.13333(2/15)	0.31818(7/22)	0.90000(18/20)
Bo_TE_101597	0.69048(29/42)	0.52941(9/17)	0.04348(1/23)	0.63158(12/19)
Bo_TE_130547	0.00000(0/46)	0.61111(11/18)	0.00000(0/23)	0.00000(0/20)
Bo_TE_169326	0.02500(1/40)	0.00000(0/18)	0.04545(1/22)	0.64706(11/17)
Bo_TE_223321	0.25000(9/36)	0.75000(12/16)	0.04762(1/21)	0.00000(0/17)
Bo_TE_80625	0.85366(35/41)	0.70588(12/17)	0.52381(11/21)	0.10526(2/19)
Bo_TE_57669	0.58140(25/43)	0.58824(10/17)	0.42857(9/21)	0.00000(0/20)
Bo_TE_223832	0.69048(29/42)	1.00000(17/17)	0.50000(11/22)	0.84211(16/19)
Bo_TE_3052	0.30952(13/42)	0.52632(10/19)	0.70000(14/20)	0.11765(2/17)
Bo_TE_179200	0.00000(0/44)	0.00000(0/17)	0.57895(11/19)	0.05000(1/20)
Bo_TE_170775	0.04545(2/44)	0.10526(2/19)	0.50000(10/20)	0.00000(0/20)
Bo_TE_137110	0.00000(0/44)	0.05263(1/19)	0.59091(13/22)	0.05000(1/20)
Bo_TE_119204	0.79545(35/44)	0.31250(5/16)	0.20000(4/20)	0.05263(1/19)
Bo_TE_223691	0.02222(1/45)	0.00000(0/17)	0.50000(11/22)	0.00000(0/20)
Bo_TE_39301	0.00000(0/44)	0.00000(0/18)	0.47368(9/19)	0.70000(14/20)
Bo_TE_66448	0.00000(0/46)	0.00000(0/19)	0.13636(3/22)	0.83333(15/18)
Bo_TE_153075	0.00000(0/41)	0.37500(6/16)	0.09091(2/22)	0.55556(10/18)
Bo_TE_147619	0.93478(43/46)	0.11765(2/17)	0.90000(18/20)	1.00000(18/18)
Bo_TE_141697	0.97727(43/44)	0.33333(6/18)	1.00000(22/22)	0.66667(12/18)
Bo_TE_174982	0.00000(0/46)	0.00000(0/19)	0.04348(1/23)	0.58824(10/17)
Bo_TE_79701	0.80000(36/45)	0.50000(9/18)	0.04545(1/22)	0.00000(0/15)
Bo_TE_105094	0.06667(3/45)	0.37500(6/16)	0.77273(17/22)	0.26316(5/19)
Bo_TE_58857	0.42857(18/42)	1.00000(17/17)	1.00000(23/23)	0.68421(13/19)
Bo_TE_210127	0.02222(1/45)	0.84211(16/19)	0.50000(11/22)	0.68421(13/19)

Bo_TE_30860	0.23256(10/43)	0.31250(5/16)	0.80000(16/20)	0.27778(5/18)
Bo_TE_128266	0.37500(15/40)	0.83333(15/18)	1.00000(22/22)	0.94737(18/19)
Bo_TE_8800	0.39474(15/38)	0.06250(1/16)	0.78947(15/19)	0.11111(2/18)
Bo_TE_57636	0.42857(18/42)	0.41176(7/17)	0.31818(7/22)	1.00000(20/20)
Bo_TE_70409	0.19048(8/42)	0.50000(8/16)	0.68182(15/22)	0.10526(2/19)
Bo_TE_169741	0.50000(20/40)	0.05263(1/19)	0.00000(0/21)	0.00000(0/18)
Bo_TE_13375	0.21951(9/41)	1.00000(17/17)	1.00000(20/20)	1.00000(20/20)
Bo_TE_53566	0.54762(23/42)	0.05556(1/18)	0.75000(15/20)	0.05263(1/19)
Bo_TE_121631	0.19565(9/46)	0.00000(0/19)	0.60000(12/20)	0.05000(1/20)
Bo_TE_22837	0.08889(4/45)	0.55556(10/18)	0.00000(0/23)	0.05000(1/20)
Bo_TE_52446	0.54762(23/42)	0.72222(13/18)	0.42857(9/21)	0.15000(3/20)
Bo_TE_94809	0.86364(38/44)	0.84211(16/19)	0.47619(10/21)	0.15789(3/19)
Bo_TE_19127	0.22222(8/36)	0.12500(2/16)	0.70000(14/20)	0.63158(12/19)
Bo_TE_3734	0.79070(34/43)	0.00000(0/16)	0.00000(0/22)	0.00000(0/19)
Bo_TE_188615	0.68889(31/45)	0.11111(2/18)	0.00000(0/22)	0.05000(1/20)
Bo_TE_70127	0.23256(10/43)	0.94118(16/17)	0.23810(5/21)	0.65000(13/20)
Bo_TE_69862	0.48837(21/43)	0.58824(10/17)	0.65000(13/20)	0.05263(1/19)
Bo_TE_200175	0.04545(2/44)	0.05556(1/18)	0.09091(2/22)	0.57895(11/19)
Bo_TE_9286	0.75000(30/40)	0.35294(6/17)	0.80952(17/21)	1.00000(20/20)
Bo_TE_150999	0.47500(19/40)	0.75000(12/16)	0.04545(1/22)	0.00000(0/20)
Bo_TE_148903	0.50000(21/42)	0.76471(13/17)	1.00000(23/23)	1.00000(20/20)
Bo_TE_196561	0.64865(24/37)	0.00000(0/17)	0.00000(0/23)	0.00000(0/18)
Bo_TE_189188	0.73333(33/45)	0.93333(14/15)	0.47619(10/21)	0.22222(4/18)
Bo_TE_201809	0.71053(27/38)	0.33333(5/15)	0.00000(0/19)	0.20000(3/15)
Bo_TE_55267	0.00000(0/46)	0.17647(3/17)	0.00000(0/21)	0.57895(11/19)
Bo_TE_149201	1.00000(41/41)	0.52941(9/17)	0.77273(17/22)	0.40000(8/20)
Bo_TE_67645	0.86047(37/43)	0.94118(16/17)	0.31579(6/19)	0.70588(12/17)
Bo_TE_236112	0.91111(41/45)	0.94737(18/19)	0.25000(5/20)	0.90000(18/20)
Bo_TE_49215	0.86364(38/44)	0.00000(0/17)	0.04545(1/22)	0.63158(12/19)
Bo_TE_5521	0.22727(10/44)	0.82353(14/17)	0.80000(16/20)	0.85000(17/20)
Bo_TE_163256	0.00000(0/44)	0.05556(1/18)	0.60000(12/20)	0.64706(11/17)
Bo_TE_29471	0.15909(7/44)	0.88235(15/17)	0.54545(12/22)	0.89474(17/19)
Bo_TE_140299	0.64865(24/37)	0.78947(15/19)	0.38095(8/21)	1.00000(20/20)
Bo_TE_90210	0.00000(0/43)	0.44444(8/18)	0.33333(7/21)	0.80000(16/20)
Bo_TE_40340	0.23913(11/46)	0.88235(15/17)	1.00000(23/23)	0.85000(17/20)
Bo_TE_8960	0.60000(27/45)	1.00000(17/17)	0.25000(5/20)	0.16667(3/18)
Bo_TE_83661	0.30233(13/43)	0.68421(13/19)	0.90000(18/20)	0.21053(4/19)
Bo_TE_45550	0.88889(40/45)	0.00000(0/18)	0.00000(0/22)	0.00000(0/20)
Bo_TE_141007	0.11111(5/45)	0.93333(14/15)	0.90476(19/21)	0.83333(15/18)
Bo_TE_204886	0.03448(1/29)	0.11111(2/18)	0.34783(8/23)	0.77778(14/18)
Bo_TE_78603	0.76744(33/43)	0.15789(3/19)	0.00000(0/22)	0.05000(1/20)
Bo_TE_227877	0.34146(14/41)	0.64706(11/17)	0.57143(12/21)	0.10000(2/20)
Bo_TE_226551	0.63636(28/44)	0.00000(0/18)	0.30435(7/23)	0.00000(0/20)
Bo_TE_23284	0.36364(16/44)	0.88235(15/17)	0.40000(8/20)	0.25000(5/20)
Bo_TE_121024	0.00000(0/39)	0.00000(0/15)	0.20000(4/20)	0.80000(16/20)
Bo_TE_23664	0.60526(23/38)	0.15789(3/19)	0.52381(11/21)	0.68421(13/19)
Bo_TE_115145	0.47500(19/40)	1.00000(19/19)	1.00000(23/23)	0.95000(19/20)
Bo_TE_76921	0.88095(37/42)	0.82353(14/17)	0.00000(0/21)	0.80000(16/20)
Bo_TE_36578	0.43750(14/32)	0.33333(3/9)	0.31579(6/19)	0.85000(17/20)
Bo_TE_196579	0.02222(1/45)	0.31579(6/19)	0.04545(1/22)	0.88889(16/18)
Bo_TE_179553	0.78571(33/42)	0.82353(14/17)	0.00000(0/23)	0.41176(7/17)
Bo_TE_159081	0.46512(20/43)	0.83333(15/18)	0.90909(20/22)	1.00000(20/20)
Bo_TE_40544	0.27027(10/37)	0.06667(1/15)	0.10000(2/20)	0.78947(15/19)
Bo_TE_211550	0.27907(12/43)	0.11765(2/17)	1.00000(19/19)	0.78947(15/19)
Bo_TE_95352	0.00000(0/45)	0.16667(3/18)	0.70000(14/20)	0.15000(3/20)
Bo_TE_204008	0.65000(26/40)	0.20000(3/15)	0.75000(15/20)	0.15000(3/20)

Bo_TE_225228	0.31707(13/41)	0.47059(8/17)	0.60000(12/20)	0.00000(0/17)
Bo_TE_146596	0.48780(20/41)	1.00000(18/18)	0.76190(16/21)	0.84211(16/19)
Bo_TE_211673	0.76190(32/42)	0.44444(8/18)	0.00000(0/20)	0.21053(4/19)
Bo_TE_191404	0.00000(0/46)	0.11765(2/17)	0.25000(5/20)	0.88889(16/18)
Bo_TE_148849	0.27907(12/43)	0.64706(11/17)	1.00000(22/22)	1.00000(20/20)
Bo_TE_101876	0.83721(36/43)	0.78947(15/19)	0.23810(5/21)	0.10526(2/19)
Bo_TE_212099	0.46341(19/41)	0.50000(9/18)	0.81818(18/22)	0.26316(5/19)
Bo_TE_105859	0.66667(28/42)	0.15789(3/19)	0.00000(0/21)	0.10000(2/20)
Bo_TE_178409	0.84091(37/44)	1.00000(17/17)	0.15000(3/20)	0.94444(17/18)
Bo_TE_86593	0.71429(30/42)	0.05882(1/17)	0.04348(1/23)	0.00000(0/20)
Bo_TE_209712	0.06818(3/44)	0.00000(0/19)	0.50000(11/22)	0.10000(2/20)
Bo_TE_46316	0.00000(0/44)	0.00000(0/19)	0.71429(15/21)	0.36842(7/19)
Bo_TE_154596	1.00000(45/45)	1.00000(18/18)	0.28571(6/21)	0.73684(14/19)
Bo_TE_170852	0.00000(0/39)	0.18750(3/16)	0.50000(11/22)	0.00000(0/20)
Bo_TE_73035	0.00000(0/42)	0.66667(12/18)	0.47368(9/19)	0.63158(12/19)
Bo_TE_144163	0.02273(1/44)	0.58824(10/17)	0.00000(0/22)	0.00000(0/19)
Bo_TE_19657	0.59091(26/44)	0.80000(12/15)	0.04348(1/23)	0.25000(5/20)
Bo_TE_50407	0.00000(0/46)	0.44444(8/18)	0.13636(3/22)	0.80000(16/20)
Bo_TE_184429	0.53846(7/13)	0.26316(5/19)	0.89474(17/19)	0.75000(12/16)
Bo_TE_32653	0.81818(36/44)	0.00000(0/18)	0.47619(10/21)	0.25000(5/20)
Bo_TE_109620	0.50000(20/40)	0.70588(12/17)	0.50000(11/22)	0.00000(0/19)
Bo_TE_220284	0.00000(0/46)	0.10526(2/19)	0.45455(10/22)	0.63158(12/19)
Bo_TE_183749	0.06818(3/44)	0.29412(5/17)	0.61905(13/21)	0.41176(7/17)
Bo_TE_115374	0.48837(21/43)	0.88235(15/17)	0.86364(19/22)	0.30000(6/20)
Bo_TE_179191	1.00000(44/44)	1.00000(16/16)	0.47619(10/21)	0.94737(18/19)
Bo_TE_210981	0.90476(38/42)	0.56250(9/16)	0.40000(8/20)	0.47368(9/19)
Bo_TE_234503	0.13333(6/45)	0.52941(9/17)	0.66667(14/21)	0.50000(10/20)
Bo_TE_221698	0.93023(40/43)	0.94444(17/18)	0.35000(7/20)	0.07143(1/14)
Bo_TE_205050	0.25581(11/43)	0.11111(2/18)	0.33333(7/21)	0.95000(19/20)
Bo_TE_174542	0.09756(4/41)	0.52941(9/17)	0.00000(0/21)	0.00000(0/20)
Bo_TE_80554	0.02222(1/45)	0.00000(0/18)	0.57143(12/21)	0.85000(17/20)
Bo_TE_107532	0.02273(1/44)	0.00000(0/18)	0.04545(1/22)	0.61111(11/18)
Bo_TE_206444	0.77273(34/44)	0.50000(8/16)	0.34783(8/23)	1.00000(19/19)
Bo_TE_222615	0.11364(5/44)	0.38889(7/18)	0.75000(15/20)	0.20000(4/20)
Bo_TE_100231	0.83333(35/42)	1.00000(18/18)	0.40000(8/20)	0.55556(10/18)
Bo_TE_38082	0.63158(24/38)	0.88235(15/17)	0.40909(9/22)	0.16667(3/18)
Bo_TE_107810	0.62222(28/45)	0.47059(8/17)	1.00000(22/22)	1.00000(20/20)
Bo_TE_178097	0.02222(1/45)	0.00000(0/18)	0.00000(0/23)	0.73684(14/19)
Bo_TE_206115	0.72093(31/43)	0.80000(12/15)	0.00000(0/22)	0.05556(1/18)
Bo_TE_16758	0.79070(34/43)	0.16667(3/18)	0.14286(3/21)	0.35000(7/20)
Bo_TE_158324	0.92683(38/41)	0.87500(14/16)	0.14286(3/21)	0.22222(4/18)
Bo_TE_223317	0.32500(13/40)	0.88235(15/17)	0.08696(2/23)	0.10526(2/19)
Bo_TE_5673	0.77273(34/44)	0.35294(6/17)	0.08696(2/23)	0.25000(4/16)
Bo_TE_63416	0.29268(12/41)	0.68421(13/19)	0.22727(5/22)	0.05263(1/19)
Bo_TE_155745	0.56098(23/41)	0.29412(5/17)	0.04545(1/22)	0.10000(2/20)
Bo_TE_53929	0.00000(0/44)	0.26667(4/15)	0.00000(0/21)	0.55556(10/18)
Bo_TE_95881	0.39535(17/43)	0.37500(6/16)	0.71429(15/21)	1.00000(19/19)
Bo_TE_94685	0.73810(31/42)	0.18750(3/16)	0.10000(2/20)	0.68421(13/19)
Bo_TE_39070	0.00000(0/46)	0.50000(8/16)	0.00000(0/22)	0.00000(0/18)
Bo_TE_145216	0.41860(18/43)	0.00000(0/17)	0.85000(17/20)	0.30000(6/20)
Bo_TE_158040	0.02222(1/45)	0.00000(0/18)	0.50000(11/22)	0.11111(2/18)
Bo_TE_91497	0.88372(38/43)	0.58824(10/17)	0.22727(5/22)	0.45000(9/20)
Bo_TE_152167	0.30952(13/42)	0.94444(17/18)	0.81818(18/22)	1.00000(19/19)
Bo_TE_23814	0.11364(5/44)	0.94118(16/17)	0.22727(5/22)	0.31579(6/19)
Bo_TE_50464	0.00000(0/46)	0.00000(0/18)	0.05000(1/20)	0.63158(12/19)
Bo_TE_33182	0.00000(0/44)	0.11765(2/17)	0.04545(1/22)	0.77778(14/18)

Bo_TE_50509	0.92857(39/42)	0.77778(14/18)	0.15000(3/20)	0.11111(2/18)
Bo_TE_66283	0.93478(43/46)	0.58824(10/17)	0.59091(13/22)	0.17647(3/17)
Bo_TE_937	0.46429(13/28)	0.56250(9/16)	0.05263(1/19)	0.10000(2/20)
Bo_TE_191539	0.20000(8/40)	0.68750(11/16)	1.00000(21/21)	1.00000(20/20)
Bo_TE_26455	0.16279(7/43)	0.61111(11/18)	0.82609(19/23)	0.85000(17/20)
Bo_TE_190925	0.46512(20/43)	0.78947(15/19)	0.90909(20/22)	0.20000(4/20)
Bo_TE_48374	0.00000(0/45)	0.52941(9/17)	0.00000(0/22)	0.10000(2/20)
Bo_TE_190206	0.56818(25/44)	0.47368(9/19)	0.00000(0/22)	0.00000(0/20)
Bo_TE_13623	0.00000(0/44)	0.62500(10/16)	0.15000(3/20)	0.10000(2/20)
Bo_TE_148534	0.00000(0/45)	0.00000(0/18)	0.00000(0/22)	0.60000(12/20)
Bo_TE_96547	0.00000(0/45)	0.00000(0/19)	0.86364(19/22)	0.31579(6/19)
Bo_TE_238286	0.68293(28/41)	0.22222(4/18)	0.04545(1/22)	0.00000(0/19)
Bo_TE_148688	0.34884(15/43)	0.52632(10/19)	1.00000(23/23)	1.00000(19/19)
Bo_TE_231282	0.00000(0/44)	0.00000(0/17)	0.20000(4/20)	0.73684(14/19)
Bo_TE_36466	0.11364(5/44)	0.77778(14/18)	0.47619(10/21)	0.94737(18/19)
Bo_TE_47968	0.47500(19/40)	0.87500(14/16)	0.00000(0/21)	0.15789(3/19)
Bo_TE_42193	0.34884(15/43)	0.88889(16/18)	1.00000(21/21)	0.95000(19/20)
Bo_TE_140633	0.45455(20/44)	0.05882(1/17)	0.00000(0/23)	0.63158(12/19)
Bo_TE_215619	0.02273(1/44)	0.05556(1/18)	0.40000(8/20)	0.52632(10/19)
Bo_TE_71845	0.74419(32/43)	0.50000(9/18)	0.19048(4/21)	0.85000(17/20)
Bo_TE_223352	0.57143(24/42)	0.11111(2/18)	0.90909(20/22)	0.90000(18/20)
Bo_TE_181609	0.73333(33/45)	0.22222(4/18)	0.35000(7/20)	0.17647(3/17)
Bo_TE_70893	0.66667(30/45)	0.16667(3/18)	0.57895(11/19)	0.60000(12/20)
Bo_TE_86847	0.92500(37/40)	0.26667(4/15)	0.29412(5/17)	0.18750(3/16)
Bo_TE_67384	0.62857(22/35)	0.86667(13/15)	1.00000(21/21)	0.47059(8/17)
Bo_TE_224632	0.42857(18/42)	0.84211(16/19)	0.35000(7/20)	0.89474(17/19)
Bo_TE_211141	0.02222(1/45)	0.00000(0/19)	0.66667(14/21)	0.64286(9/14)
Bo_TE_214313	0.73171(30/41)	0.18750(3/16)	0.35000(7/20)	0.40000(8/20)
Bo_TE_201534	0.00000(0/43)	0.70588(12/17)	0.76190(16/21)	0.00000(0/20)
Bo_TE_230614	0.00000(0/44)	0.76471(13/17)	0.63158(12/19)	0.00000(0/18)
Bo_TE_33612	0.27907(12/43)	0.00000(0/17)	0.22727(5/22)	0.78947(15/19)
Bo_TE_168984	1.00000(46/46)	0.50000(8/16)	1.00000(22/22)	0.75000(15/20)
Bo_TE_169429	0.69048(29/42)	0.10526(2/19)	0.09091(2/22)	0.21053(4/19)
Bo_TE_177645	1.00000(45/45)	0.23529(4/17)	1.00000(23/23)	1.00000(20/20)
Bo_TE_80245	0.00000(0/38)	0.00000(0/18)	0.78947(15/19)	0.26316(5/19)
Bo_TE_164945	0.00000(0/44)	0.00000(0/18)	0.40909(9/22)	0.75000(15/20)
Bo_TE_169292	0.90476(38/42)	0.11765(2/17)	0.45455(10/22)	0.20000(4/20)
Bo_TE_142118	0.30000(12/40)	0.50000(9/18)	0.00000(0/23)	0.00000(0/19)
Bo_TE_145777	0.77273(34/44)	0.35294(6/17)	0.05000(1/20)	0.31250(5/16)
Bo_TE_53647	0.02222(1/45)	0.00000(0/17)	0.08696(2/23)	0.68421(13/19)
Bo_TE_3046	0.71429(30/42)	0.43750(7/16)	0.00000(0/20)	0.27778(5/18)
Bo_TE_193500	0.34884(15/43)	0.58824(10/17)	0.20000(4/20)	0.05000(1/20)
Bo_TE_43240	1.00000(43/43)	0.50000(9/18)	1.00000(21/21)	1.00000(20/20)
Bo_TE_5375	0.14286(6/42)	0.88235(15/17)	0.57143(12/21)	0.90000(18/20)
Bo_TE_125489	0.50000(21/42)	0.00000(0/18)	0.42857(9/21)	0.55556(10/18)
Bo_TE_216458	0.59091(26/44)	0.52941(9/17)	0.05000(1/20)	0.00000(0/20)
Bo_TE_133632	0.78049(32/41)	0.38889(7/18)	1.00000(23/23)	1.00000(20/20)
Bo_TE_193783	0.60465(26/43)	0.25000(4/16)	0.17391(4/23)	0.85000(17/20)
Bo_TE_61646	0.54545(24/44)	0.29412(5/17)	0.66667(14/21)	0.94444(17/18)
Bo_TE_8204	0.76923(30/39)	0.17647(3/17)	0.00000(0/21)	0.00000(0/20)
Bo_TE_80793	0.50000(21/42)	0.55556(10/18)	0.04348(1/23)	0.78947(15/19)
Bo_TE_95319	0.02273(1/44)	0.64706(11/17)	0.52632(10/19)	0.31579(6/19)
Bo_TE_37147	0.28889(13/45)	0.50000(8/16)	0.00000(0/21)	0.05000(1/20)
Bo_TE_175042	0.00000(0/45)	0.00000(0/19)	0.47619(10/21)	0.50000(8/16)
Bo_TE_194484	0.54054(20/37)	0.82353(14/17)	0.20000(4/20)	0.27778(5/18)
Bo_TE_9483	0.65909(29/44)	0.05882(1/17)	0.04545(1/22)	0.00000(0/20)

Bo_TE_182195	0.21739(10/46)	0.72222(13/18)	0.00000(0/22)	0.31579(6/19)
Bo_TE_213854	0.92857(39/42)	0.94118(16/17)	0.33333(7/21)	0.45000(9/20)
Bo_TE_49676	0.42500(17/40)	0.94118(16/17)	0.31579(6/19)	0.36842(7/19)
Bo_TE_222249	0.97619(41/42)	0.31579(6/19)	1.00000(21/21)	0.94737(18/19)
Bo_TE_105376	0.08889(4/45)	0.29412(5/17)	0.00000(0/23)	0.57895(11/19)
Bo_TE_129061	0.02381(1/42)	0.00000(0/18)	0.00000(0/22)	0.55000(11/20)
Bo_TE_78988	0.00000(0/45)	0.00000(0/17)	0.04545(1/22)	0.88889(16/18)
Bo_TE_184264	0.94872(37/39)	1.00000(15/15)	0.80952(17/21)	0.25000(5/20)
Bo_TE_63658	0.30952(13/42)	0.64706(11/17)	0.00000(0/22)	0.09091(1/11)
Bo_TE_56753	1.00000(43/43)	1.00000(18/18)	0.23810(5/21)	0.10526(2/19)
Bo_TE_103916	0.87805(36/41)	0.46667(7/15)	0.07143(1/14)	0.22222(4/18)
Bo_TE_145010	0.50000(19/38)	0.94444(17/18)	1.00000(20/20)	0.94737(18/19)
Bo_TE_208073	0.90698(39/43)	0.50000(9/18)	1.00000(23/23)	0.68750(11/16)
Bo_TE_130432	0.82927(34/41)	0.84211(16/19)	0.05000(1/20)	0.38889(7/18)
Bo_TE_235860	0.06977(3/43)	0.56250(9/16)	0.13636(3/22)	0.05000(1/20)
Bo_TE_194872	0.56098(23/41)	0.50000(9/18)	0.00000(0/21)	0.57895(11/19)
Bo_TE_85780	1.00000(42/42)	0.68421(13/19)	0.94737(18/19)	0.50000(10/20)
Bo_TE_25365	0.60526(23/38)	0.52632(10/19)	0.17391(4/23)	0.05882(1/17)
Bo_TE_6633	0.18421(7/38)	0.93750(15/16)	0.30435(7/23)	0.06667(1/15)
Bo_TE_222699	0.95122(39/41)	0.68421(13/19)	0.55000(11/20)	0.26316(5/19)
Bo_TE_89225	0.52500(21/40)	0.17647(3/17)	0.86364(19/22)	1.00000(18/18)
Bo_TE_230505	0.31579(12/38)	0.06250(1/16)	0.00000(0/19)	0.62500(10/16)
Bo_TE_86012	0.61538(24/39)	0.77778(14/18)	0.36364(8/22)	0.00000(0/20)
Bo_TE_81579	0.00000(0/46)	0.76471(13/17)	0.54545(12/22)	0.00000(0/20)
Bo_TE_146571	0.51282(20/39)	0.00000(0/18)	0.15789(3/19)	0.42105(8/19)
Bo_TE_28866	0.62500(25/40)	0.00000(0/18)	0.47619(10/21)	0.64706(11/17)
Bo_TE_188953	0.36585(15/41)	0.20000(3/15)	0.90000(18/20)	0.95000(19/20)
Bo_TE_193540	0.00000(0/44)	0.58824(10/17)	0.14286(3/21)	0.00000(0/20)
Bo_TE_133564	0.84091(37/44)	0.70588(12/17)	0.13636(3/22)	0.65000(13/20)
Bo_TE_133260	0.02174(1/46)	0.61111(11/18)	0.04348(1/23)	0.26316(5/19)
Bo_TE_137524	0.00000(0/43)	0.00000(0/16)	0.81818(18/22)	0.00000(0/20)
Bo_TE_111114	0.18605(8/43)	0.23529(4/17)	0.72727(16/22)	0.85000(17/20)
Bo_TE_170947	0.44186(19/43)	0.88235(15/17)	0.57143(12/21)	0.05000(1/20)
Bo_TE_73118	0.89744(35/39)	0.38889(7/18)	0.73684(14/19)	0.38889(7/18)
Bo_TE_212197	0.21739(10/46)	0.50000(8/16)	0.91304(21/23)	0.31579(6/19)
Bo_TE_73047	0.04762(2/42)	0.61111(11/18)	0.50000(11/22)	0.63158(12/19)
Bo_TE_185305	0.97674(42/43)	0.41176(7/17)	0.61905(13/21)	0.85000(17/20)
Bo_TE_234059	0.00000(0/45)	0.11765(2/17)	0.00000(0/22)	0.83333(15/18)
Bo_TE_68843	0.02273(1/44)	0.64706(11/17)	0.04545(1/22)	0.15000(3/20)
Bo_TE_56611	0.86667(39/45)	0.50000(8/16)	0.23810(5/21)	0.05000(1/20)
Bo_TE_88487	0.18421(7/38)	1.00000(16/16)	0.47619(10/21)	0.50000(8/16)
Bo_TE_236102	0.90698(39/43)	0.94118(16/17)	0.25000(5/20)	0.90000(18/20)
Bo_TE_148623	0.29268(12/41)	0.50000(9/18)	0.68182(15/22)	1.00000(18/18)
Bo_TE_10862	0.04545(2/44)	0.23529(4/17)	0.09091(2/22)	0.85000(17/20)
Bo_TE_92886	0.51163(22/43)	0.82353(14/17)	0.05263(1/19)	0.15000(3/20)
Bo_TE_174506	0.04762(2/42)	0.52941(9/17)	0.00000(0/22)	0.00000(0/20)
Bo_TE_33298	0.74419(32/43)	0.11765(2/17)	0.00000(0/23)	0.11111(2/18)
Bo_TE_141504	0.80000(36/45)	0.06250(1/16)	0.04762(1/21)	0.21053(4/19)
Bo_TE_164961	1.00000(46/46)	1.00000(18/18)	0.40000(8/20)	0.26316(5/19)
Bo_TE_200082	0.13636(6/44)	0.18750(3/16)	0.71429(15/21)	0.47368(9/19)
Bo_TE_226364	0.72093(31/43)	0.26316(5/19)	1.00000(22/22)	1.00000(19/19)
Bo_TE_57350	0.97826(45/46)	1.00000(18/18)	0.75000(15/20)	0.36842(7/19)
Bo_TE_205082	0.00000(0/46)	0.00000(0/18)	0.45000(9/20)	0.85000(17/20)
Bo_TE_83740	0.09091(4/44)	0.05556(1/18)	0.73684(14/19)	0.29412(5/17)
Bo_TE_236244	0.00000(0/45)	0.52941(9/17)	0.09091(2/22)	0.16667(3/18)
Bo_TE_196487	0.17500(7/40)	0.50000(9/18)	1.00000(22/22)	1.00000(17/17)

Bo_TE_90461	0.00000(0/44)	0.00000(0/19)	0.33333(7/21)	0.75000(15/20)
Bo_TE_32979	0.07143(3/42)	0.62500(10/16)	0.47619(10/21)	0.21053(4/19)
Bo_TE_14092	0.73810(31/42)	1.00000(15/15)	0.35000(7/20)	0.22222(4/18)
Bo_TE_143662	0.90698(39/43)	0.11111(2/18)	0.42857(9/21)	0.30000(6/20)
Bo_TE_136523	0.29545(13/44)	0.00000(0/17)	0.60870(14/23)	0.50000(10/20)
Bo_TE_75282	0.05263(1/19)	0.18750(3/16)	1.00000(19/19)	0.29412(5/17)
Bo_TE_26645	0.20930(9/43)	0.50000(8/16)	0.00000(0/23)	0.00000(0/20)
Bo_TE_196541	0.25714(9/35)	0.44444(8/18)	1.00000(21/21)	1.00000(17/17)
Bo_TE_143225	0.60976(25/41)	0.05882(1/17)	0.27273(6/22)	0.00000(0/20)
Bo_TE_102700	0.08889(4/45)	0.00000(0/19)	0.42857(9/21)	0.72222(13/18)
Bo_TE_150574	0.13953(6/43)	0.41176(7/17)	0.69565(16/23)	0.88889(16/18)
Bo_TE_45172	0.53333(24/45)	0.37500(6/16)	0.89474(17/19)	0.00000(0/20)
Bo_TE_172507	0.02222(1/45)	0.77778(14/18)	0.85714(18/21)	0.27778(5/18)
Bo_TE_66836	0.83333(35/42)	0.55556(10/18)	0.00000(0/23)	0.00000(0/19)
Bo_TE_133282	0.75000(30/40)	0.11765(2/17)	0.10000(2/20)	0.73684(14/19)
Bo_TE_222548	0.23256(10/43)	0.31250(5/16)	0.04545(1/22)	0.55000(11/20)
Bo_TE_172484	0.02222(1/45)	0.00000(0/19)	0.04762(1/21)	0.52941(9/17)
Bo_TE_91568	0.11111(5/45)	0.33333(5/15)	0.70000(14/20)	0.16667(3/18)
Bo_TE_42556	0.93182(41/44)	0.82353(14/17)	0.35000(7/20)	0.65000(13/20)
Bo_TE_57461	0.65909(29/44)	0.00000(0/18)	0.52632(10/19)	0.10000(2/20)
Bo_TE_145833	0.19512(8/41)	0.88235(15/17)	0.33333(6/18)	1.00000(19/19)
Bo_TE_236871	0.43590(17/39)	0.70588(12/17)	0.27273(6/22)	0.05263(1/19)
Bo_TE_44196	0.00000(0/45)	0.55556(10/18)	0.38095(8/21)	0.75000(15/20)
Bo_TE_71150	1.00000(43/43)	0.88889(16/18)	0.42105(8/19)	1.00000(20/20)
Bo_TE_28320	0.20455(9/44)	0.81250(13/16)	0.04348(1/23)	0.27778(5/18)
Bo_TE_82201	0.73810(31/42)	0.29412(5/17)	0.42857(9/21)	0.00000(0/19)
Bo_TE_9703	0.55263(21/38)	0.23529(4/17)	1.00000(22/22)	0.78947(15/19)
Bo_TE_87251	0.68421(26/38)	0.76471(13/17)	0.05263(1/19)	0.05000(1/20)
Bo_TE_70404	0.00000(0/41)	0.42857(6/14)	0.34783(8/23)	0.88235(15/17)
Bo_TE_183441	0.58974(23/39)	0.93750(15/16)	0.85000(17/20)	0.26316(5/19)
Bo_TE_182210	0.20000(9/45)	0.72222(13/18)	0.00000(0/21)	0.30000(6/20)
Bo_TE_122643	0.02326(1/43)	0.00000(0/19)	0.57143(12/21)	0.00000(0/20)
Bo_TE_21072	0.22222(10/45)	0.88235(15/17)	0.50000(11/22)	0.90000(18/20)
Bo_TE_56706	0.55814(24/43)	0.06250(1/16)	0.00000(0/23)	0.00000(0/19)
Bo_TE_49049	0.93182(41/44)	0.22222(4/18)	0.45000(9/20)	0.68421(13/19)
Bo_TE_11784	0.46341(19/41)	0.64706(11/17)	1.00000(23/23)	1.00000(20/20)
Bo_TE_193605	0.34091(15/44)	0.58824(10/17)	0.25000(5/20)	0.05263(1/19)
Bo_TE_95601	0.50000(21/42)	0.50000(8/16)	0.00000(0/20)	0.05556(1/18)
Bo_TE_198909	0.15556(7/45)	0.70588(12/17)	1.00000(23/23)	1.00000(20/20)
Bo_TE_179065	0.82051(32/39)	0.00000(0/18)	0.28571(6/21)	0.15000(3/20)
Bo_TE_235028	0.65116(28/43)	0.41176(7/17)	0.04545(1/22)	0.30000(6/20)
Bo_TE_2598	0.13333(6/45)	0.18750(3/16)	0.91304(21/23)	0.68421(13/19)
Bo_TE_116631	0.06818(3/44)	0.28571(4/14)	0.57143(12/21)	0.36842(7/19)
Bo_TE_236079	1.00000(42/42)	0.94118(16/17)	0.50000(11/22)	0.10526(2/19)
Bo_TE_113004	0.68293(28/41)	0.05263(1/19)	0.04545(1/22)	0.15789(3/19)
Bo_TE_97171	1.00000(41/41)	1.00000(19/19)	0.19048(4/21)	0.22222(4/18)
Bo_TE_210701	0.62500(25/40)	0.66667(12/18)	0.31579(6/19)	0.16667(3/18)
Bo_TE_213272	0.32558(14/43)	0.94118(16/17)	1.00000(23/23)	0.95000(19/20)
Bo_TE_230899	0.86047(37/43)	0.89474(17/19)	0.00000(0/23)	0.00000(0/20)
Bo_TE_200593	0.56410(22/39)	0.33333(6/18)	0.00000(0/23)	0.00000(0/20)
Bo_TE_60959	0.61905(26/42)	0.58824(10/17)	0.04545(1/22)	0.10526(2/19)
Bo_TE_133722	0.93333(42/45)	0.94118(16/17)	0.81818(18/22)	0.22222(4/18)
Bo_TE_157960	0.74419(32/43)	0.23529(4/17)	0.00000(0/23)	0.05000(1/20)
Bo_TE_92394	0.04545(2/44)	0.56250(9/16)	0.72222(13/18)	0.72222(13/18)
Bo_TE_49832	0.22727(10/44)	0.66667(12/18)	0.57143(12/21)	0.75000(15/20)
Bo_TE_30538	0.63889(23/36)	1.00000(15/15)	0.00000(0/21)	0.58824(10/17)

Bo_TE_217989	0.43590(17/39)	0.64706(11/17)	0.18182(4/22)	0.10526(2/19)
Bo_TE_4273	0.13636(6/44)	0.29412(5/17)	0.08696(2/23)	0.84211(16/19)
Bo_TE_178033	0.02326(1/43)	0.62500(10/16)	0.00000(0/22)	0.21053(4/19)
Bo_TE_102457	0.06667(3/45)	0.84211(16/19)	0.39130(9/23)	0.52632(10/19)
Bo_TE_219285	0.37838(14/37)	1.00000(17/17)	0.71429(15/21)	0.80000(16/20)
Bo_TE_222622	0.80952(34/42)	1.00000(17/17)	0.40909(9/22)	0.73684(14/19)
Bo_TE_215748	0.00000(0/45)	0.00000(0/19)	0.59091(13/22)	0.00000(0/20)
Bo_TE_120363	0.09302(4/43)	0.12500(2/16)	0.06250(1/16)	0.72222(13/18)
Bo_TE_150812	0.00000(0/45)	0.17647(3/17)	0.33333(7/21)	0.55000(11/20)
Bo_TE_129555	0.04545(2/44)	0.50000(8/16)	0.20000(4/20)	0.80000(16/20)
Bo_TE_48925	0.69048(29/42)	0.88235(15/17)	0.34783(8/23)	0.63158(12/19)
Bo_TE_89198	0.42857(18/42)	0.94118(16/17)	0.14286(3/21)	0.27778(5/18)
Bo_TE_121896	0.46341(19/41)	0.88889(16/18)	0.85714(18/21)	0.30000(6/20)
Bo_TE_180716	0.00000(0/43)	0.00000(0/19)	0.57895(11/19)	0.33333(6/18)
Bo_TE_155161	0.09091(4/44)	0.11765(2/17)	0.28571(6/21)	0.68421(13/19)
Bo_TE_83873	0.64706(22/34)	0.22222(4/18)	0.65000(13/20)	0.73684(14/19)
Bo_TE_33119	0.16279(7/43)	0.29412(5/17)	0.31818(7/22)	0.90000(18/20)
Bo_TE_8955	0.02273(1/44)	0.00000(0/17)	0.71429(15/21)	0.65000(13/20)
Bo_TE_88436	0.60526(23/38)	0.50000(8/16)	0.09091(2/22)	0.26316(5/19)
Bo_TE_172711	0.69048(29/42)	0.50000(8/16)	0.57143(12/21)	0.10526(2/19)
Bo_TE_200745	0.83784(31/37)	0.17647(3/17)	0.09091(2/22)	0.00000(0/19)
Bo_TE_52176	0.50000(21/42)	0.11765(2/17)	0.00000(0/23)	0.00000(0/19)
Bo_TE_13416	0.02273(1/44)	0.11765(2/17)	0.68182(15/22)	0.66667(12/18)
Bo_TE_237338	0.75000(33/44)	0.06250(1/16)	0.00000(0/22)	0.26316(5/19)
Bo_TE_80064	0.51282(20/39)	0.11111(2/18)	0.04545(1/22)	0.00000(0/20)
Bo_TE_185983	0.56098(23/41)	0.43750(7/16)	0.42105(8/19)	0.05263(1/19)
Bo_TE_152394	0.81818(36/44)	0.10526(2/19)	0.45000(9/20)	0.10000(2/20)
Bo_TE_101385	0.00000(0/43)	0.61538(8/13)	0.25000(5/20)	0.52632(10/19)
Bo_TE_94897	0.69048(29/42)	0.37500(6/16)	0.00000(0/23)	0.00000(0/20)
Bo_TE_165564	0.15909(7/44)	0.71429(10/14)	0.45000(9/20)	0.68421(13/19)
Bo_TE_119592	0.40000(18/45)	0.94118(16/17)	0.31818(7/22)	0.57895(11/19)
Bo_TE_128722	0.26190(11/42)	0.72222(13/18)	0.55000(11/20)	0.00000(0/20)
Bo_TE_55453	0.97778(44/45)	0.56250(9/16)	0.28571(6/21)	0.95000(19/20)
Bo_TE_52701	0.60465(26/43)	0.11111(2/18)	0.04762(1/21)	0.10526(2/19)
Bo_TE_155089	0.09756(4/41)	0.11111(2/18)	0.27273(6/22)	0.66667(12/18)
Bo_TE_101670	0.25000(10/40)	0.81250(13/16)	0.92308(12/13)	0.57143(4/7)
Bo_TE_151472	0.23077(9/39)	0.73684(14/19)	0.52381(11/21)	1.00000(12/12)
Bo_TE_15721	0.56098(23/41)	0.41176(7/17)	0.36364(8/22)	0.00000(0/20)
Bo_TE_93212	0.38636(17/44)	0.76471(13/17)	0.09524(2/21)	0.21053(4/19)
Bo_TE_218311	0.55814(24/43)	0.00000(0/17)	0.52174(12/23)	0.85000(17/20)
Bo_TE_197260	0.00000(0/45)	0.11111(2/18)	0.00000(0/23)	0.52632(10/19)
Bo_TE_137131	0.50000(22/44)	0.77778(14/18)	1.00000(23/23)	0.63158(12/19)
Bo_TE_60469	0.50000(21/42)	0.68750(11/16)	1.00000(22/22)	1.00000(20/20)
Bo_TE_75360	0.00000(0/45)	0.00000(0/19)	0.14286(3/21)	0.52632(10/19)
Bo_TE_170127	0.53846(21/39)	0.35294(6/17)	0.00000(0/22)	0.36842(7/19)
Bo_TE_121627	0.48837(21/43)	0.83333(15/18)	0.57895(11/19)	0.15789(3/19)
Bo_TE_151021	0.00000(0/41)	0.52941(9/17)	0.00000(0/21)	0.50000(5/10)
Bo_TE_108012	0.00000(0/42)	0.68750(11/16)	0.20000(4/20)	0.17647(3/17)
Bo_TE_169514	0.00000(0/41)	0.00000(0/19)	0.14286(3/21)	0.70000(14/20)
Bo_TE_51436	0.40000(16/40)	0.83333(15/18)	1.00000(20/20)	1.00000(20/20)
Bo_TE_122399	0.62162(23/37)	0.05882(1/17)	0.34783(8/23)	0.57895(11/19)
Bo_TE_211755	0.50000(20/40)	1.00000(14/14)	0.23810(5/21)	0.83333(15/18)
Bo_TE_136555	0.02273(1/44)	0.61111(11/18)	0.00000(0/21)	0.00000(0/20)
Bo_TE_67324	0.75610(31/41)	0.94444(17/18)	0.38095(8/21)	0.43750(7/16)
Bo_TE_77086	0.13333(6/45)	0.25000(4/16)	0.00000(0/23)	0.56250(9/16)
Bo_TE_143453	1.00000(32/32)	1.00000(19/19)	1.00000(17/17)	0.27778(5/18)

Bo_TE_165701	0.65116(28/43)	0.05882(1/17)	0.00000(0/23)	0.10000(2/20)
Bo_TE_91687	0.75000(33/44)	0.50000(9/18)	1.00000(21/21)	0.94737(18/19)
Bo_TE_185666	0.78571(33/42)	0.06250(1/16)	0.45000(9/20)	0.10526(2/19)
Bo_TE_54495	0.68293(28/41)	0.73333(11/15)	0.00000(0/22)	0.44444(8/18)
Bo_TE_95657	0.07143(3/42)	0.38889(7/18)	0.65000(13/20)	0.05263(1/19)
Bo_TE_105441	0.31579(12/38)	0.05882(1/17)	0.60000(12/20)	0.50000(10/20)
Bo_TE_163070	0.00000(0/43)	0.00000(0/17)	0.28571(6/21)	0.55000(11/20)
Bo_TE_73098	0.10000(4/40)	0.58824(10/17)	0.27778(5/18)	0.63158(12/19)
Bo_TE_90240	0.27907(12/43)	0.58824(10/17)	0.00000(0/23)	0.00000(0/20)
Bo_TE_78050	0.47727(21/44)	0.00000(0/18)	0.50000(10/20)	0.70000(14/20)
Bo_TE_27663	0.50000(22/44)	0.27778(5/18)	0.81818(18/22)	0.78947(15/19)
Bo_TE_113756	0.82927(34/41)	0.57895(11/19)	0.04545(1/22)	0.10000(2/20)
Bo_TE_99908	0.75000(33/44)	0.23529(4/17)	0.95455(21/22)	1.00000(19/19)
Bo_TE_223503	1.00000(46/46)	0.43750(7/16)	0.28571(6/21)	0.20000(4/20)
Bo_TE_32124	0.07500(3/40)	0.83333(15/18)	0.10526(2/19)	0.05000(1/20)
Bo_TE_12743	0.82927(34/41)	0.06667(1/15)	0.08696(2/23)	0.05263(1/19)
Bo_TE_232127	0.55556(25/45)	0.47059(8/17)	0.04545(1/22)	0.22222(4/18)
Bo_TE_177633	0.00000(0/44)	0.00000(0/18)	0.45000(9/20)	0.52632(10/19)
Bo_TE_2370	0.93182(41/44)	0.25000(4/16)	0.15000(3/20)	0.10526(2/19)
Bo_TE_122578	0.88095(37/42)	0.11765(2/17)	1.00000(20/20)	0.78947(15/19)
Bo_TE_49529	0.97727(43/44)	0.41176(7/17)	0.45455(10/22)	0.25000(5/20)
Bo_TE_135503	0.45238(19/42)	0.05556(1/18)	0.30435(7/23)	0.78947(15/19)
Bo_TE_24589	0.02222(1/45)	0.47059(8/17)	0.52632(10/19)	0.50000(10/20)
Bo_TE_30940	0.76190(32/42)	0.70588(12/17)	0.19048(4/21)	0.72222(13/18)
Bo_TE_215704	0.26667(12/45)	0.52941(9/17)	0.60000(12/20)	0.94737(18/19)
Bo_TE_204429	0.97436(38/39)	0.31250(5/16)	0.45000(9/20)	0.94118(16/17)
Bo_TE_27339	0.68182(30/44)	0.11765(2/17)	0.71429(15/21)	0.73684(14/19)
Bo_TE_128783	0.02174(1/46)	0.16667(3/18)	0.57143(12/21)	0.00000(0/20)
Bo_TE_231045	0.51163(22/43)	0.00000(0/17)	0.00000(0/23)	0.00000(0/19)
Bo_TE_216661	0.56818(25/44)	0.18750(3/16)	0.14286(3/21)	0.00000(0/19)
Bo_TE_182072	0.34091(15/44)	1.00000(18/18)	1.00000(20/20)	0.57895(11/19)
Bo_TE_177525	0.09091(4/44)	0.00000(0/17)	0.66667(14/21)	0.21053(4/19)
Bo_TE_76136	0.66667(26/39)	0.58824(10/17)	0.13636(3/22)	0.50000(10/20)
Bo_TE_61424	0.11364(5/44)	0.77778(14/18)	0.08696(2/23)	0.10000(2/20)
Bo_TE_234145	0.55814(24/43)	0.05556(1/18)	0.00000(0/22)	0.05263(1/19)
Bo_TE_138188	0.28571(12/42)	0.75000(12/16)	0.82609(19/23)	0.52632(10/19)
Bo_TE_111403	0.53659(22/41)	0.70588(12/17)	0.04762(1/21)	0.25000(5/20)
Bo_TE_33769	0.52381(22/42)	0.47368(9/19)	0.00000(0/22)	0.00000(0/19)
Bo_TE_134281	0.36585(15/41)	0.72222(13/18)	0.13636(3/22)	0.31579(6/19)
Bo_TE_106964	0.56410(22/39)	0.00000(0/16)	0.14286(3/21)	0.00000(0/17)
Bo_TE_74352	0.48718(19/39)	0.40000(6/15)	0.59091(13/22)	0.05263(1/19)
Bo_TE_163745	1.00000(38/38)	0.82353(14/17)	0.57895(11/19)	0.23529(4/17)
Bo_TE_73277	0.00000(0/46)	0.05556(1/18)	0.30435(7/23)	0.50000(9/18)
Bo_TE_168649	0.54545(24/44)	1.00000(17/17)	0.34783(8/23)	0.20000(4/20)
Bo_TE_26931	0.75610(31/41)	0.14286(2/14)	0.54545(12/22)	0.00000(0/19)
Bo_TE_221187	0.39024(16/41)	0.82353(14/17)	1.00000(22/22)	0.90000(18/20)
Bo_TE_207022	0.11111(5/45)	0.00000(0/17)	0.52632(10/19)	0.50000(9/18)
Bo_TE_238601	1.00000(42/42)	0.68750(11/16)	0.22727(5/22)	0.15789(3/19)
Bo_TE_178675	0.74359(29/39)	0.86667(13/15)	0.52381(11/21)	0.05263(1/19)
Bo_TE_236219	0.10870(5/46)	0.81250(13/16)	1.00000(21/21)	0.77778(14/18)
Bo_TE_199094	0.86047(37/43)	0.20000(3/15)	0.27273(6/22)	0.78947(15/19)
Bo_TE_34860	0.76744(33/43)	1.00000(18/18)	0.10526(2/19)	0.50000(10/20)
Bo_TE_111700	0.27907(12/43)	0.00000(0/18)	0.76190(16/21)	0.00000(0/18)
Bo_TE_27034	0.82500(33/40)	0.43750(7/16)	0.50000(11/22)	0.94737(18/19)
Bo_TE_165763	0.00000(0/43)	0.00000(0/19)	0.00000(0/21)	0.60000(12/20)
Bo_TE_162297	0.69048(29/42)	0.23529(4/17)	0.68421(13/19)	0.82353(14/17)

Bo_TE_50605	0.63415(26/41)	0.88889(16/18)	0.90909(20/22)	0.15789(3/19)
Bo_TE_236188	0.65854(27/41)	0.05882(1/17)	0.00000(0/22)	0.00000(0/18)
Bo_TE_109428	0.13636(6/44)	0.75000(12/16)	0.28571(6/21)	0.47059(8/17)
Bo_TE_23843	0.11111(5/45)	0.77778(14/18)	0.13636(3/22)	0.16667(3/18)
Bo_TE_229689	0.04444(2/45)	0.10526(2/19)	0.45000(9/20)	0.75000(15/20)
Bo_TE_20936	0.48718(19/39)	1.00000(17/17)	0.28571(6/21)	0.77778(14/18)
Bo_TE_137308	0.00000(0/45)	0.11111(2/18)	0.30000(6/20)	0.63158(12/19)
Bo_TE_66468	0.57500(23/40)	0.00000(0/16)	0.85714(18/21)	0.83333(15/18)
Bo_TE_16926	0.97674(42/43)	1.00000(17/17)	0.42105(8/19)	1.00000(19/19)
Bo_TE_70408	0.80488(33/41)	0.15789(3/19)	0.31818(7/22)	0.89474(17/19)
Bo_TE_49574	0.35897(14/39)	0.94118(16/17)	0.17391(4/23)	0.42105(8/19)
Bo_TE_36015	0.39024(16/41)	0.00000(0/17)	0.50000(11/22)	0.45000(9/20)
Bo_TE_106178	0.57500(23/40)	0.76471(13/17)	0.00000(0/23)	0.05000(1/20)
Bo_TE_189183	0.25581(11/43)	0.05556(1/18)	0.55556(10/18)	0.80000(16/20)
Bo_TE_212609	0.32558(14/43)	0.33333(6/18)	0.90476(19/21)	0.52632(10/19)
Bo_TE_99184	0.00000(0/44)	0.57895(11/19)	0.09091(2/22)	0.05000(1/20)
Bo_TE_190720	0.83333(35/42)	0.70588(12/17)	0.21053(4/19)	0.47368(9/19)
Bo_TE_45353	0.95455(42/44)	0.00000(0/13)	0.35000(7/20)	0.42857(3/7)
Bo_TE_67709	0.80952(34/42)	1.00000(17/17)	0.28571(6/21)	0.89474(17/19)
Bo_TE_188591	0.18182(8/44)	0.29412(5/17)	0.63636(14/22)	0.05000(1/20)
Bo_TE_165310	0.46341(19/41)	1.00000(17/17)	0.85714(18/21)	0.90000(18/20)
Bo_TE_45901	0.00000(0/45)	0.50000(9/18)	0.00000(0/23)	0.00000(0/20)
Bo_TE_90628	0.08696(4/46)	0.68750(11/16)	0.65000(13/20)	0.15000(3/20)
Bo_TE_128597	0.23810(10/42)	0.66667(12/18)	0.57143(12/21)	0.00000(0/20)
Bo_TE_8706	0.80000(36/45)	0.35294(6/17)	0.66667(14/21)	0.95000(19/20)
Bo_TE_49971	0.18182(8/44)	0.11111(2/18)	0.00000(0/22)	0.65000(13/20)
Bo_TE_148235	0.02222(1/45)	0.00000(0/18)	0.65000(13/20)	0.78947(15/19)
Bo_TE_32654	0.11628(5/43)	0.88889(16/18)	0.34783(8/23)	0.73684(14/19)
Bo_TE_85311	0.02174(1/46)	0.00000(0/18)	0.68421(13/19)	0.89474(17/19)
Bo_TE_180520	0.37209(16/43)	0.55556(10/18)	0.00000(0/23)	0.00000(0/20)
Bo_TE_12295	0.28571(12/42)	0.37500(6/16)	0.35000(7/20)	0.78947(15/19)
Bo_TE_190713	0.78571(33/42)	0.55556(10/18)	0.09091(2/22)	0.50000(9/18)
Bo_TE_33072	0.17500(7/40)	0.60000(9/15)	0.63158(12/19)	0.00000(0/20)
Bo_TE_224929	0.02439(1/41)	0.00000(0/9)	0.21053(4/19)	0.63158(12/19)
Bo_TE_111978	0.46154(18/39)	0.77778(14/18)	0.22727(5/22)	0.85714(12/14)
Bo_TE_15270	0.50000(21/42)	0.00000(0/17)	0.77273(17/22)	0.57895(11/19)
Bo_TE_136376	0.00000(0/45)	0.00000(0/17)	0.33333(7/21)	0.83333(15/18)
Bo_TE_163515	0.45000(18/40)	0.56250(9/16)	0.09091(2/22)	0.78947(15/19)
Bo_TE_103851	1.00000(44/44)	0.70588(12/17)	0.38095(8/21)	0.16667(3/18)
Bo_TE_50284	0.23256(10/43)	0.76471(13/17)	0.57895(11/19)	0.77778(14/18)
Bo_TE_211555	0.75610(31/41)	0.88889(16/18)	0.04762(1/21)	0.20000(4/20)
Bo_TE_106098	0.89130(41/46)	0.16667(3/18)	1.00000(22/22)	1.00000(19/19)
Bo_TE_174799	0.25581(11/43)	0.64706(11/17)	0.45455(10/22)	0.89474(17/19)
Bo_TE_232730	0.02273(1/44)	0.00000(0/19)	0.33333(7/21)	0.63158(12/19)
Bo_TE_3166	0.62791(27/43)	0.35294(6/17)	0.05000(1/20)	0.40000(8/20)
Bo_TE_144222	0.95122(39/41)	0.23529(4/17)	0.86957(20/23)	1.00000(20/20)
Bo_TE_174604	0.09302(4/43)	0.18750(3/16)	0.50000(11/22)	0.00000(0/20)
Bo_TE_239622	0.76923(30/39)	0.33333(5/15)	0.00000(0/21)	0.00000(0/20)
Bo_TE_69102	0.20930(9/43)	0.88889(16/18)	0.40000(8/20)	0.78947(15/19)
Bo_TE_174521	0.04651(2/43)	0.50000(8/16)	0.00000(0/22)	0.00000(0/20)
Bo_TE_235782	1.00000(45/45)	0.76471(13/17)	0.47826(11/23)	0.20000(4/20)
Bo_TE_194587	0.13953(6/43)	0.76471(13/17)	0.00000(0/20)	0.30000(6/20)
Bo_TE_48957	0.38095(16/42)	0.72222(13/18)	0.09091(2/22)	0.27778(5/18)
Bo_TE_155530	0.30769(12/39)	0.31250(5/16)	0.13636(3/22)	0.80000(16/20)
Bo_TE_80688	0.21429(9/42)	0.20000(3/15)	0.47619(10/21)	0.94444(17/18)
Bo_TE_107664	0.60000(27/45)	0.00000(0/18)	0.04348(1/23)	0.25000(5/20)

Bo_TE_57204	1.00000(45/45)	1.00000(19/19)	0.77273(17/22)	0.44444(8/18)
Bo_TE_32946	0.41026(16/39)	0.52941(9/17)	1.00000(22/22)	0.94737(18/19)
Bo_TE_105283	0.46341(19/41)	0.12500(2/16)	1.00000(22/22)	0.50000(10/20)
Bo_TE_215187	0.06667(3/45)	0.57143(8/14)	0.85000(17/20)	0.41176(7/17)
Bo_TE_112325	0.00000(0/46)	0.16667(3/18)	0.75000(15/20)	0.57895(11/19)
Bo_TE_41160	0.25000(10/40)	0.82353(14/17)	0.00000(0/22)	0.15000(3/20)
Bo_TE_198560	0.82927(34/41)	0.12500(2/16)	0.04762(1/21)	0.05000(1/20)
Bo_TE_53606	0.02222(1/45)	0.00000(0/18)	0.76190(16/21)	0.89474(17/19)
Bo_TE_18047	0.69767(30/43)	0.17647(3/17)	0.77778(14/18)	0.15000(3/20)
Bo_TE_179829	0.07143(3/42)	0.56250(9/16)	0.65000(13/20)	0.80000(16/20)
Bo_TE_39169	0.39535(17/43)	0.88235(15/17)	1.00000(23/23)	1.00000(20/20)
Bo_TE_217037	0.59524(25/42)	0.36842(7/19)	0.18182(4/22)	0.90000(18/20)
Bo_TE_204996	0.25581(11/43)	0.11111(2/18)	0.36364(8/22)	0.94737(18/19)
Bo_TE_55426	0.45946(17/37)	0.05882(1/17)	0.04545(1/22)	0.75000(12/16)
Bo_TE_90247	0.17949(7/39)	0.22222(4/18)	0.00000(0/22)	0.78947(15/19)
Bo_TE_40338	0.25581(11/43)	0.58824(10/17)	0.52381(11/21)	0.00000(0/20)
Bo_TE_43376	0.88372(38/43)	0.38889(7/18)	1.00000(23/23)	1.00000(20/20)
Bo_TE_66093	0.30952(13/42)	0.00000(0/16)	0.70000(14/20)	0.42105(8/19)
Bo_TE_82558	0.06818(3/44)	0.23529(4/17)	0.71429(15/21)	0.50000(10/20)
Bo_TE_116187	0.97436(38/39)	0.56250(9/16)	0.52632(10/19)	0.42105(8/19)
Bo_TE_100104	0.07317(3/41)	0.05556(1/18)	0.69565(16/23)	0.47368(9/19)
Bo_TE_88820	0.18182(8/44)	0.72222(13/18)	0.00000(0/23)	0.05000(1/20)
Bo_TE_55072	0.04651(2/43)	0.05263(1/19)	0.68421(13/19)	0.33333(6/18)
Bo_TE_113970	0.52500(21/40)	0.50000(8/16)	0.59091(13/22)	1.00000(19/19)
Bo_TE_239998	0.67500(27/40)	0.33333(6/18)	1.00000(23/23)	0.70000(14/20)
Bo_TE_234617	0.93182(41/44)	0.94444(17/18)	0.38095(8/21)	0.55000(11/20)
Bo_TE_98799	0.16279(7/43)	0.25000(4/16)	0.71429(15/21)	0.31579(6/19)
Bo_TE_197927	0.51282(20/39)	0.00000(0/18)	0.50000(11/22)	0.73684(14/19)
Bo_TE_149401	0.93333(42/45)	0.38889(7/18)	1.00000(23/23)	0.61111(11/18)
Bo_TE_145553	1.00000(28/28)	0.36842(7/19)	1.00000(23/23)	1.00000(20/20)
Bo_TE_144175	0.02273(1/44)	0.11111(2/18)	0.80000(16/20)	0.80000(16/20)
Bo_TE_157432	0.00000(0/46)	0.00000(0/19)	0.40000(8/20)	0.75000(15/20)
Bo_TE_12195	0.00000(0/46)	0.50000(9/18)	0.00000(0/22)	0.00000(0/20)
Bo_TE_216709	0.06522(3/46)	0.00000(0/17)	0.42857(9/21)	0.50000(10/20)
Bo_TE_106932	0.14634(6/41)	0.81250(13/16)	0.76190(16/21)	0.73684(14/19)
Bo_TE_115369	0.52273(23/44)	0.82353(14/17)	0.14286(3/21)	0.65000(13/20)
Bo_TE_114401	0.11905(5/42)	0.22222(4/18)	0.00000(0/23)	0.50000(10/20)
Bo_TE_171443	0.76744(33/43)	0.31250(5/16)	0.77273(17/22)	0.26316(5/19)
Bo_TE_230065	0.78571(33/42)	0.16667(3/18)	0.47368(9/19)	0.20000(4/20)
Bo_TE_178131	0.28947(11/38)	0.64706(11/17)	0.95455(21/22)	0.23529(4/17)
Bo_TE_78761	0.86667(39/45)	1.00000(18/18)	0.90476(19/21)	0.42105(8/19)
Bo_TE_93075	0.53488(23/43)	0.23529(4/17)	0.04762(1/21)	0.00000(0/20)
Bo_TE_156422	0.08889(4/45)	0.00000(0/19)	0.45455(10/22)	0.50000(9/18)
Bo_TE_170790	0.02381(1/42)	0.50000(8/16)	0.00000(0/23)	0.44444(8/18)
Bo_TE_90846	0.53846(21/39)	0.05263(1/19)	0.00000(0/23)	0.00000(0/20)
Bo_TE_121882	0.53659(22/41)	0.05882(1/17)	0.42857(9/21)	0.68421(13/19)
Bo_TE_227199	0.09756(4/41)	0.00000(0/15)	0.04545(1/22)	0.88889(16/18)
Bo_TE_215458	0.11905(5/42)	0.00000(0/19)	0.21739(5/23)	0.57895(11/19)
Bo_TE_44173	0.90909(40/44)	0.88889(16/18)	0.45000(9/20)	0.15000(3/20)
Bo_TE_135806	0.13158(5/38)	0.00000(0/8)	0.36364(8/22)	0.63158(12/19)
Bo_TE_106160	0.88636(39/44)	0.16667(3/18)	1.00000(23/23)	1.00000(20/20)
Bo_TE_158546	0.00000(0/45)	0.10526(2/19)	0.77273(17/22)	0.05000(1/20)
Bo_TE_222243	0.75676(28/37)	0.11765(2/17)	1.00000(23/23)	0.22222(4/18)
Bo_TE_226769	0.06818(3/44)	0.52632(10/19)	0.63158(12/19)	0.00000(0/20)
Bo_TE_236098	0.90698(39/43)	0.82353(14/17)	0.26316(5/19)	0.80000(16/20)
Bo_TE_59724	0.13333(6/45)	0.62500(10/16)	0.22727(5/22)	0.80000(16/20)

Bo_TE_66382	0.17073(7/41)	0.58824(10/17)	0.31579(6/19)	0.00000(0/18)
Bo_TE_46297	0.07692(3/39)	0.70588(12/17)	0.86957(20/23)	0.47059(8/17)
Bo_TE_225363	0.26829(11/41)	0.00000(0/17)	0.54545(12/22)	0.00000(0/20)
Bo_TE_110969	0.41860(18/43)	0.11765(2/17)	0.04545(1/22)	0.63158(12/19)
Bo_TE_88749	0.15789(6/38)	0.35294(6/17)	1.00000(23/23)	1.00000(20/20)
Bo_TE_78812	0.13636(6/44)	0.00000(0/18)	0.14286(3/21)	0.50000(9/18)
Bo_TE_138870	0.07143(3/42)	0.15789(3/19)	0.40000(8/20)	0.73684(14/19)
Bo_TE_129889	0.40000(16/40)	0.05556(1/18)	0.60000(12/20)	0.26316(5/19)
Bo_TE_227258	0.71429(30/42)	0.93750(15/16)	0.33333(7/21)	0.88889(16/18)
Bo_TE_91578	0.13333(6/45)	0.55556(10/18)	0.77273(17/22)	0.15000(3/20)
Bo_TE_87389	0.65789(25/38)	0.77778(14/18)	0.00000(0/23)	0.00000(0/18)
Bo_TE_156760	0.00000(0/44)	0.00000(0/19)	0.04348(1/23)	0.57895(11/19)
Bo_TE_137053	0.33333(14/42)	0.11765(2/17)	0.70000(14/20)	0.66667(12/18)
Bo_TE_205045	0.22727(10/44)	0.12500(2/16)	0.33333(7/21)	0.84211(16/19)
Bo_TE_66035	0.13333(6/45)	0.58824(10/17)	0.04545(1/22)	0.31579(6/19)
Bo_TE_144170	0.02174(1/46)	0.52941(9/17)	0.13636(3/22)	0.00000(0/20)
Bo_TE_170682	0.63415(26/41)	0.12500(2/16)	0.00000(0/23)	0.68421(13/19)
Bo_TE_143582	0.11905(5/42)	0.56250(9/16)	0.23810(5/21)	0.00000(0/20)
Bo_TE_98480	0.46341(19/41)	0.58824(10/17)	0.05263(1/19)	0.11111(2/18)
Bo_TE_194600	1.00000(44/44)	0.47059(8/17)	0.95652(22/23)	0.70000(14/20)
Bo_TE_178831	0.65000(26/40)	0.23529(4/17)	0.50000(11/22)	0.05000(1/20)
Bo_TE_78426	0.00000(0/42)	0.05263(1/19)	0.00000(0/22)	0.57895(11/19)
Bo_TE_130531	0.18605(8/43)	0.05882(1/17)	0.76190(16/21)	0.73684(14/19)
Bo_TE_191516	0.24390(10/41)	0.94444(17/18)	1.00000(21/21)	1.00000(20/20)
Bo_TE_227291	0.39535(17/43)	0.00000(0/17)	0.68182(15/22)	0.22222(4/18)
Bo_TE_203136	0.72973(27/37)	0.81250(13/16)	0.42857(9/21)	0.05556(1/18)
Bo_TE_231084	0.73810(31/42)	0.11765(2/17)	0.00000(0/23)	0.36842(7/19)
Bo_TE_231186	0.73810(31/42)	0.83333(15/18)	0.14286(3/21)	0.33333(5/15)
Bo_TE_190788	0.11905(5/42)	0.05556(1/18)	0.65000(13/20)	0.00000(0/20)
Bo_TE_235669	0.20000(9/45)	0.11111(2/18)	0.86957(20/23)	0.47368(9/19)
Bo_TE_132972	0.21429(9/42)	0.11765(2/17)	0.80000(16/20)	0.00000(0/20)
Bo_TE_74245	0.71111(32/45)	0.40000(6/15)	0.00000(0/20)	0.00000(0/19)
Bo_TE_188856	0.11111(5/45)	0.58824(10/17)	0.42857(9/21)	0.00000(0/20)
Bo_TE_102691	0.23913(11/46)	0.94444(17/18)	0.86364(19/22)	0.77778(14/18)
Bo_TE_165144	0.06667(3/45)	0.56250(9/16)	0.33333(7/21)	0.00000(0/20)
Bo_TE_57602	0.41463(17/41)	0.38889(7/18)	0.31579(6/19)	1.00000(20/20)
Bo_TE_170867	0.00000(0/44)	0.10526(2/19)	0.50000(10/20)	0.00000(0/20)
Bo_TE_198574	0.11111(5/45)	0.16667(3/18)	0.35000(7/20)	0.94737(18/19)
Bo_TE_130298	0.00000(0/45)	0.00000(0/19)	0.60000(12/20)	0.00000(0/20)
Bo_TE_83536	0.40476(17/42)	0.55556(10/18)	0.60000(12/20)	1.00000(19/19)
Bo_TE_136759	0.15909(7/44)	0.50000(9/18)	1.00000(22/22)	0.84211(16/19)
Bo_TE_193192	0.72500(29/40)	0.22222(4/18)	0.00000(0/23)	0.70000(14/20)
Bo_TE_213973	0.31111(14/45)	0.52941(9/17)	0.00000(0/23)	0.00000(0/20)
Bo_TE_178088	0.93182(41/44)	0.88235(15/17)	0.27273(6/22)	0.55556(10/18)
Bo_TE_226184	0.69048(29/42)	0.16667(3/18)	0.00000(0/22)	0.00000(0/20)
Bo_TE_27872	0.22222(10/45)	0.89474(17/19)	0.52381(11/21)	0.89474(17/19)
Bo_TE_195800	0.00000(0/46)	0.00000(0/19)	0.09524(2/21)	0.70000(14/20)
Bo_TE_218908	0.18182(4/22)	0.16667(3/18)	0.82353(14/17)	0.81250(13/16)
Bo_TE_201392	0.00000(0/44)	0.42105(8/19)	0.70000(14/20)	0.05000(1/20)
Bo_TE_232367	0.40476(17/42)	0.52941(9/17)	0.54545(12/22)	0.00000(0/20)
Bo_TE_14658	0.17500(7/40)	0.22222(4/18)	0.80952(17/21)	0.95000(19/20)
Bo_TE_182223	0.80000(36/45)	0.27778(5/18)	1.00000(22/22)	0.70000(14/20)
Bo_TE_133747	0.36364(16/44)	0.11111(2/18)	0.45000(9/20)	0.72222(13/18)
Bo_TE_104429	0.04444(2/45)	0.05882(1/17)	0.19048(4/21)	0.85000(17/20)
Bo_TE_93411	0.20930(9/43)	0.35294(6/17)	1.00000(21/21)	0.33333(6/18)
Bo_TE_128561	0.02174(1/46)	0.15789(3/19)	0.57143(12/21)	0.00000(0/20)

Bo_TE_70958	0.58537(24/41)	0.61111(11/18)	0.00000(0/21)	0.05000(1/20)
Bo_TE_171360	0.46341(19/41)	0.57895(11/19)	0.71429(15/21)	0.17647(3/17)
Bo_TE_46954	0.62791(27/43)	0.35294(6/17)	0.80000(16/20)	0.95000(19/20)
Bo_TE_175046	1.00000(43/43)	1.00000(19/19)	0.84615(11/13)	0.47368(9/19)
Bo_TE_96238	0.26829(11/41)	0.88889(16/18)	1.00000(21/21)	0.88235(15/17)
Bo_TE_179749	0.51163(22/43)	0.29412(5/17)	0.23810(5/21)	0.00000(0/19)
Bo_TE_51075	0.00000(0/38)	0.64706(11/17)	0.00000(0/22)	0.05000(1/20)
Bo_TE_62917	0.55556(25/45)	0.33333(6/18)	0.65000(13/20)	1.00000(20/20)
Bo_TE_162263	0.27273(12/44)	0.66667(12/18)	0.00000(0/23)	0.10000(2/20)
Bo_TE_90842	0.44186(19/43)	0.66667(12/18)	0.39130(9/23)	0.00000(0/20)
Bo_TE_13095	0.97727(43/44)	0.75000(12/16)	0.86364(19/22)	0.40000(8/20)
Bo_TE_105004	0.33333(15/45)	0.68750(11/16)	0.57895(11/19)	0.15000(3/20)
Bo_TE_234957	0.56818(25/44)	0.27778(5/18)	0.00000(0/22)	0.30000(6/20)
Bo_TE_226771	0.11628(5/43)	0.58824(10/17)	0.81818(18/22)	0.80000(16/20)
Bo_TE_145105	0.35000(14/40)	0.83333(15/18)	0.13636(3/22)	0.80000(16/20)
Bo_TE_41885	0.18605(8/43)	0.35294(6/17)	0.90000(18/20)	0.84211(16/19)
Bo_TE_163869	0.06977(3/43)	0.11765(2/17)	0.09524(2/21)	0.85000(17/20)
Bo_TE_44036	0.86047(37/43)	0.21053(4/19)	0.81818(18/22)	0.89474(17/19)
Bo_TE_2183	0.62500(25/40)	0.87500(14/16)	0.34783(8/23)	0.84211(16/19)
Bo_TE_92110	0.20455(9/44)	0.56250(9/16)	0.00000(0/22)	0.00000(0/18)
Bo_TE_132953	0.80488(33/41)	0.60000(9/15)	0.21739(5/23)	0.95000(19/20)
Bo_TE_182199	0.83333(35/42)	0.29412(5/17)	1.00000(22/22)	0.73684(14/19)
Bo_TE_136904	0.97778(44/45)	1.00000(19/19)	0.45455(10/22)	0.68421(13/19)
Bo_TE_57996	0.05263(2/38)	0.64706(11/17)	0.22727(5/22)	0.35000(7/20)
Bo_TE_30875	0.78049(32/41)	0.68750(11/16)	0.21053(4/19)	0.70588(12/17)
Bo_TE_54799	0.02381(1/42)	0.00000(0/15)	0.86364(19/22)	0.61538(8/13)
Bo_TE_88760	0.85000(34/40)	0.62500(10/16)	0.04348(1/23)	0.00000(0/20)
Bo_TE_40705	0.00000(0/45)	0.00000(0/16)	0.65000(13/20)	0.30000(6/20)
Bo_TE_137608	0.21429(9/42)	0.33333(5/15)	0.76190(16/21)	0.10000(2/20)
Bo_TE_96336	0.50000(21/42)	0.05263(1/19)	0.00000(0/23)	0.00000(0/19)
Bo_TE_83834	0.94118(32/34)	0.44444(8/18)	0.80952(17/21)	0.95000(19/20)
Bo_TE_96241	0.65909(29/44)	0.00000(0/19)	0.00000(0/22)	0.05263(1/19)
Bo_TE_169799	0.92105(35/38)	0.88889(16/18)	0.13043(3/23)	0.33333(6/18)
Bo_TE_14156	0.18182(8/44)	0.11765(2/17)	0.26087(6/23)	1.00000(20/20)
Bo_TE_38907	0.00000(0/45)	0.11111(2/18)	0.17391(4/23)	0.55000(11/20)
Bo_TE_50628	0.39024(16/41)	0.11765(2/17)	0.08696(2/23)	0.85000(17/20)
Bo_TE_25657	0.50000(20/40)	0.61111(11/18)	0.00000(0/21)	0.00000(0/18)
Bo_TE_37509	0.02174(1/46)	0.05556(1/18)	0.31818(7/22)	0.68421(13/19)
Bo_TE_16661	0.04545(2/44)	0.37500(6/16)	0.66667(14/21)	0.61111(11/18)
Bo_TE_171126	0.51220(21/41)	0.23529(4/17)	0.40000(8/20)	0.85000(17/20)
Bo_TE_59716	0.11628(5/43)	0.62500(10/16)	0.23810(5/21)	0.80000(16/20)
Bo_TE_94404	0.00000(0/45)	0.10526(2/19)	0.45000(9/20)	0.70000(14/20)
Bo_TE_120560	0.09524(4/42)	0.23529(4/17)	0.15789(3/19)	0.83333(15/18)
Bo_TE_61083	0.48780(20/41)	0.64706(11/17)	0.04762(1/21)	0.47059(8/17)
Bo_TE_2344	0.61538(24/39)	0.37500(6/16)	0.09524(2/21)	0.10000(2/20)
Bo_TE_201466	0.76923(30/39)	0.82353(14/17)	0.30435(7/23)	0.15789(3/19)
Bo_TE_80658	0.16667(7/42)	0.00000(0/19)	0.00000(0/23)	0.77778(14/18)
Bo_TE_50334	0.95556(43/45)	0.62500(10/16)	0.52381(11/21)	0.20000(4/20)
Bo_TE_135949	0.06818(3/44)	0.00000(0/13)	0.52381(11/21)	0.72222(13/18)
Bo_TE_142816	0.34146(14/41)	0.93750(15/16)	0.22727(5/22)	0.15000(3/20)
Bo_TE_25339	0.41026(16/39)	0.64706(11/17)	1.00000(23/23)	0.94444(17/18)
Bo_TE_77076	0.79545(35/44)	0.50000(9/18)	0.68421(13/19)	0.05263(1/19)
Bo_TE_125737	0.00000(0/44)	0.62500(10/16)	0.65000(13/20)	0.11111(2/18)
Bo_TE_171036	0.48780(20/41)	0.81250(13/16)	0.65000(13/20)	0.15000(3/20)
Bo_TE_96438	0.33333(13/39)	0.52941(9/17)	0.00000(0/21)	0.25000(5/20)
Bo_TE_31667	0.44186(19/43)	0.94118(16/17)	0.33333(6/18)	0.15789(3/19)

Bo_TE_173617	1.00000(44/44)	1.00000(18/18)	0.33333(7/21)	0.38889(7/18)
Bo_TE_124159	0.20455(9/44)	0.72222(13/18)	0.80952(17/21)	0.85000(17/20)
Bo_TE_103371	0.86047(37/43)	0.38889(7/18)	1.00000(21/21)	0.78947(15/19)
Bo_TE_186274	0.50000(20/40)	0.88235(15/17)	0.00000(0/22)	0.05263(1/19)
Bo_TE_81493	0.17778(8/45)	0.00000(0/18)	0.78261(18/23)	0.00000(0/20)
Bo_TE_122704	0.40000(16/40)	0.16667(3/18)	0.70000(14/20)	0.63158(12/19)
Bo_TE_178048	0.00000(0/43)	0.26667(4/15)	0.85000(17/20)	0.63158(12/19)
Bo_TE_93379	0.47826(11/23)	0.29412(5/17)	0.95455(21/22)	0.52941(9/17)
Bo_TE_163853	0.02222(1/45)	0.18750(3/16)	0.33333(7/21)	0.84211(16/19)
Bo_TE_24518	0.97727(43/44)	1.00000(18/18)	0.47368(9/19)	0.90000(18/20)
Bo_TE_235313	0.15556(7/45)	0.00000(0/18)	0.30435(7/23)	0.52632(10/19)
Bo_TE_15503	0.82609(38/46)	0.06250(1/16)	0.00000(0/23)	0.00000(0/19)
Bo_TE_73115	0.07692(3/39)	0.64706(11/17)	0.28571(6/21)	0.63158(12/19)
Bo_TE_29199	0.22727(10/44)	0.72222(13/18)	0.84211(16/19)	0.85000(17/20)
Bo_TE_137587	0.21429(9/42)	0.35714(5/14)	0.81818(18/22)	0.21053(4/19)
Bo_TE_235030	0.65116(28/43)	0.37500(6/16)	0.04762(1/21)	0.26316(5/19)
Bo_TE_209915	0.38462(15/39)	0.00000(0/17)	0.63636(14/22)	0.25000(5/20)
Bo_TE_57466	0.36585(15/41)	1.00000(17/17)	0.50000(11/22)	0.89474(17/19)
Bo_TE_171143	0.53488(23/43)	0.27778(5/18)	0.38095(8/21)	0.85000(17/20)
Bo_TE_140110	0.04651(2/43)	0.25000(4/16)	0.65000(13/20)	0.82353(14/17)
Bo_TE_113214	0.42857(18/42)	0.60000(9/15)	1.00000(23/23)	0.90000(18/20)
Bo_TE_163127	0.00000(0/43)	0.73684(14/19)	0.00000(0/23)	0.00000(0/20)
Bo_TE_165240	0.31707(13/41)	0.94444(17/18)	0.86364(19/22)	0.80000(16/20)
Bo_TE_82549	0.06667(3/45)	0.23529(4/17)	0.71429(15/21)	0.50000(9/18)
Bo_TE_109949	0.31707(13/41)	0.17647(3/17)	0.45455(10/22)	1.00000(20/20)
Bo_TE_156980	0.50000(22/44)	0.11765(2/17)	0.04348(1/23)	0.68421(13/19)
Bo_TE_94733	0.76190(32/42)	0.18750(3/16)	0.08696(2/23)	0.72222(13/18)
Bo_TE_47862	0.76190(32/42)	0.00000(0/19)	0.00000(0/23)	0.00000(0/19)
Bo_TE_108723	0.02326(1/43)	0.70588(12/17)	0.35000(7/20)	0.21053(4/19)
Bo_TE_203617	0.34146(14/41)	0.17647(3/17)	0.68421(13/19)	0.89474(17/19)
Bo_TE_163063	0.00000(0/45)	0.10526(2/19)	0.52381(11/21)	0.16667(3/18)
Bo_TE_104933	0.93182(41/44)	1.00000(16/16)	0.38095(8/21)	0.78947(15/19)
Bo_TE_222171	0.88095(37/42)	0.00000(0/19)	0.00000(0/23)	0.21053(4/19)
Bo_TE_202723	0.95349(41/43)	0.55556(10/18)	0.43478(10/23)	0.10526(2/19)
Bo_TE_237230	0.08889(4/45)	0.88889(16/18)	0.00000(0/22)	0.05000(1/20)
Bo_TE_82001	0.00000(0/44)	0.10526(2/19)	0.63636(14/22)	0.44444(8/18)
Bo_TE_211776	0.74359(29/39)	0.16667(3/18)	0.00000(0/21)	0.00000(0/19)
Bo_TE_126599	0.51163(22/43)	0.00000(0/18)	0.00000(0/22)	0.00000(0/20)
Bo_TE_206689	0.60465(26/43)	0.05882(1/17)	0.30000(6/20)	0.31579(6/19)
Bo_TE_230294	0.22500(9/40)	0.40000(6/15)	0.00000(0/23)	0.63158(12/19)
Bo_TE_156643	0.39535(17/43)	0.07143(1/14)	0.00000(0/23)	0.73684(14/19)
Bo_TE_48377	0.00000(0/44)	0.62500(10/16)	0.00000(0/23)	0.00000(0/16)
Bo_TE_73440	0.70000(28/40)	0.17647(3/17)	0.00000(0/23)	0.05000(1/20)
Bo_TE_153703	0.93182(41/44)	0.12500(2/16)	0.47619(10/21)	0.31579(6/19)
Bo_TE_101826	0.65909(29/44)	0.40000(6/15)	0.00000(0/23)	0.00000(0/20)
Bo_TE_41144	0.97619(41/42)	1.00000(18/18)	0.95455(21/22)	0.26316(5/19)
Bo_TE_97532	0.65789(25/38)	0.76471(13/17)	0.04348(1/23)	0.41176(7/17)
Bo_TE_173907	0.25581(11/43)	0.52941(9/17)	0.40909(9/22)	0.00000(0/20)
Bo_TE_84822	0.27907(12/43)	0.88889(16/18)	0.66667(4/6)	0.11111(1/9)
Bo_TE_9125	0.91111(41/45)	0.18750(3/16)	1.00000(22/22)	1.00000(20/20)
Bo_TE_54524	0.00000(0/44)	0.05882(1/17)	0.63636(14/22)	0.05000(1/20)
Bo_TE_119766	0.30233(13/43)	0.88235(15/17)	1.00000(21/21)	0.68421(13/19)
Bo_TE_92764	0.88372(38/43)	0.31250(5/16)	0.80000(16/20)	0.21053(4/19)
Bo_TE_200202	0.20455(9/44)	0.47059(8/17)	0.45455(10/22)	0.73684(14/19)
Bo_TE_62845	0.97778(44/45)	0.33333(6/18)	0.55000(11/20)	1.00000(20/20)
Bo_TE_162578	0.65116(28/43)	0.17647(3/17)	0.00000(0/22)	0.00000(0/20)

Bo_TE_99331	0.11628(5/43)	0.00000(0/19)	0.57143(12/21)	0.33333(6/18)
Bo_TE_109396	0.67442(29/43)	0.23529(4/17)	0.95652(22/23)	0.55000(11/20)
Bo_TE_43286	0.95349(41/43)	0.37500(6/16)	1.00000(23/23)	1.00000(19/19)
Bo_TE_5442	0.82927(34/41)	1.00000(17/17)	0.45000(9/20)	0.78947(15/19)
Bo_TE_237830	0.00000(0/44)	0.05556(1/18)	0.78261(18/23)	0.15000(3/20)
Bo_TE_27531	0.25000(11/44)	0.94737(18/19)	1.00000(11/11)	1.00000(19/19)
Bo_TE_140812	0.41463(17/41)	0.94737(18/19)	0.44444(8/18)	0.42105(8/19)
Bo_TE_234092	0.23810(10/42)	0.00000(0/19)	0.00000(0/22)	0.50000(10/20)
Bo_TE_113792	0.16667(5/30)	0.00000(0/19)	0.05882(1/17)	0.82353(14/17)
Bo_TE_17974	0.06818(3/44)	0.17647(3/17)	0.31579(6/19)	0.73684(14/19)
Bo_TE_54341	0.04545(2/44)	0.58824(10/17)	0.31579(6/19)	0.57895(11/19)
Bo_TE_192064	0.60000(24/40)	0.31250(5/16)	0.00000(0/21)	0.16667(3/18)
Bo_TE_169715	0.81395(35/43)	0.23529(4/17)	0.87500(7/8)	0.36842(7/19)
Bo_TE_179179	0.04651(2/43)	0.00000(0/18)	0.04348(1/23)	0.57895(11/19)
Bo_TE_89883	0.55814(24/43)	0.00000(0/18)	0.00000(0/23)	0.00000(0/20)
Bo_TE_74092	0.09302(4/43)	0.00000(0/16)	0.52381(11/21)	0.26316(5/19)
Bo_TE_13838	0.39024(16/41)	0.55556(10/18)	0.00000(0/22)	0.84211(16/19)
Bo_TE_134570	0.35897(14/39)	0.12500(2/16)	0.85714(18/21)	0.95000(19/20)
Bo_TE_12147	0.47500(19/40)	0.12500(2/16)	1.00000(22/22)	0.15000(3/20)
Bo_TE_226660	0.82222(37/45)	0.53333(8/15)	0.70000(14/20)	0.22222(4/18)
Bo_TE_16884	0.25581(11/43)	0.18750(3/16)	1.00000(23/23)	1.00000(20/20)
Bo_TE_60836	0.16667(7/42)	0.12500(2/16)	0.85714(18/21)	0.50000(10/20)
Bo_TE_111532	0.00000(0/45)	0.11765(2/17)	0.89474(17/19)	0.00000(0/20)
Bo_TE_30023	0.21951(9/41)	0.23529(4/17)	0.13043(3/23)	0.88235(15/17)
Bo_TE_91642	0.00000(0/46)	0.05263(1/19)	0.38095(8/21)	0.70000(14/20)
Bo_TE_222687	0.93478(43/46)	0.68421(13/19)	0.58824(10/17)	0.26316(5/19)
Bo_TE_153327	0.67500(27/40)	0.66667(12/18)	0.90476(19/21)	0.25000(5/20)
Bo_TE_128407	0.59524(25/42)	0.10526(2/19)	0.00000(0/23)	0.05263(1/19)
Bo_TE_134159	0.44444(20/45)	0.55556(10/18)	0.00000(0/23)	0.15789(3/19)
Bo_TE_115376	0.47619(20/42)	0.83333(15/18)	0.85000(17/20)	0.30000(6/20)
Bo_TE_74344	0.57895(22/38)	0.25000(3/12)	0.61905(13/21)	0.11111(2/18)
Bo_TE_72996	0.95000(38/40)	0.38889(7/18)	0.57143(12/21)	0.40000(8/20)
Bo_TE_116983	0.51351(19/37)	0.00000(0/18)	0.09524(2/21)	0.05556(1/18)
Bo_TE_117688	0.57143(24/42)	0.35294(6/17)	0.00000(0/23)	0.05000(1/20)
Bo_TE_191211	0.00000(0/45)	0.55556(10/18)	0.05000(1/20)	0.23077(3/13)
Bo_TE_169225	0.09756(4/41)	0.50000(8/16)	0.00000(0/22)	0.15789(3/19)
Bo_TE_116401	0.97826(45/46)	0.46667(7/15)	0.71429(15/21)	0.15000(3/20)
Bo_TE_53650	0.87500(35/40)	0.94118(16/17)	0.91304(21/23)	0.11111(2/18)
Bo_TE_191103	0.58537(24/41)	0.18750(3/16)	0.05263(1/19)	0.22222(4/18)
Bo_TE_77677	0.02174(1/46)	0.07692(1/13)	0.55556(10/18)	0.89474(17/19)
Bo_TE_48674	0.45238(19/42)	0.62500(10/16)	0.28571(6/21)	0.05000(1/20)
Bo_TE_234808	0.11364(5/44)	0.83333(15/18)	1.00000(22/22)	0.90000(18/20)
Bo_TE_222333	0.86364(38/44)	0.81250(13/16)	0.19048(4/21)	0.40000(8/20)
Bo_TE_224486	0.21429(9/42)	0.05263(1/19)	0.70000(14/20)	0.10526(2/19)
Bo_TE_4370	0.65116(28/43)	0.00000(0/17)	0.00000(0/23)	0.00000(0/20)
Bo_TE_42607	0.69767(30/43)	0.88235(15/17)	0.57143(12/21)	0.36842(7/19)
Bo_TE_83753	0.78571(33/42)	1.00000(18/18)	0.25000(5/20)	0.70000(14/20)
Bo_TE_65577	0.04545(2/44)	0.58824(10/17)	0.18182(4/22)	0.10526(2/19)
Bo_TE_14754	0.14634(6/41)	0.00000(0/18)	0.18182(4/22)	0.65000(13/20)
Bo_TE_61363	0.86364(38/44)	0.50000(8/16)	0.00000(0/22)	0.25000(5/20)
Bo_TE_108385	0.47727(21/44)	0.12500(2/16)	0.95000(19/20)	0.94737(18/19)
Bo_TE_155141	0.91111(41/45)	0.94118(16/17)	0.72727(16/22)	0.30000(6/20)
Bo_TE_151846	0.61364(27/44)	0.11111(2/18)	0.10526(2/19)	0.05263(1/19)
Bo_TE_93051	0.31818(14/44)	0.87500(14/16)	0.95238(20/21)	0.90000(18/20)
Bo_TE_124172	0.77778(35/45)	0.23529(4/17)	0.19048(4/21)	0.15789(3/19)
Bo_TE_36886	0.56098(23/41)	0.56250(9/16)	0.18182(4/22)	0.00000(0/20)

Bo_TE_171482	0.63415(26/41)	0.11111(2/18)	0.00000(0/22)	0.00000(0/13)
Bo_TE_174993	0.00000(0/44)	0.00000(0/19)	0.04348(1/23)	0.58824(10/17)
Bo_TE_136477	0.02273(1/44)	0.82353(14/17)	0.27273(6/22)	0.31579(6/19)
Bo_TE_106119	0.11111(5/45)	0.83333(15/18)	0.00000(0/22)	0.00000(0/20)
Bo_TE_89079	0.02222(1/45)	0.31250(5/16)	0.80952(17/21)	0.42105(8/19)
Bo_TE_150552	0.93182(41/44)	0.43750(7/16)	0.30000(6/20)	0.40000(8/20)
Bo_TE_94497	1.00000(46/46)	0.83333(15/18)	0.54545(12/22)	0.47368(9/19)
Bo_TE_116435	1.00000(45/45)	1.00000(19/19)	0.20000(4/20)	0.52632(10/19)
Bo_TE_10584	0.31707(13/41)	0.62500(10/16)	1.00000(21/21)	1.00000(20/20)
Bo_TE_29113	0.00000(0/44)	0.64706(11/17)	0.09091(2/22)	0.78947(15/19)
Bo_TE_155327	0.35714(15/42)	0.05263(1/19)	0.27273(6/22)	0.57895(11/19)
Bo_TE_119760	0.69767(30/43)	0.06250(1/16)	0.00000(0/23)	0.31579(6/19)
Bo_TE_224242	0.31707(13/41)	0.00000(0/19)	0.04348(1/23)	0.50000(9/18)
Bo_TE_129070	0.02381(1/42)	0.00000(0/18)	0.00000(0/21)	0.66667(12/18)
Bo_TE_204924	0.00000(0/46)	0.63158(12/19)	0.43478(10/23)	0.05263(1/19)
Bo_TE_169386	0.00000(0/45)	0.38889(7/18)	0.04545(1/22)	0.50000(9/18)
Bo_TE_180940	0.43182(19/44)	0.76471(13/17)	0.35000(7/20)	0.95000(19/20)
Bo_TE_198693	0.83333(35/42)	0.12500(2/16)	0.00000(0/21)	0.00000(0/19)
Bo_TE_45249	0.42500(17/40)	0.41176(7/17)	0.90909(20/22)	0.17647(3/17)
Bo_TE_130020	0.00000(0/46)	0.00000(0/19)	0.84211(16/19)	0.41176(7/17)
Bo_TE_65277	0.81081(30/37)	0.26667(4/15)	0.43478(10/23)	0.05882(1/17)
Bo_TE_158852	0.62500(25/40)	0.50000(8/16)	0.36364(8/22)	0.89474(17/19)
Bo_TE_49570	0.60976(25/41)	0.05882(1/17)	0.43478(10/23)	0.15789(3/19)
Bo_TE_34776	0.20000(8/40)	0.00000(0/19)	0.45000(9/20)	0.52632(10/19)
Bo_TE_43132	0.04444(2/45)	0.58824(10/17)	0.00000(0/23)	0.00000(0/18)
Bo_TE_220805	0.00000(0/45)	0.17647(3/17)	0.21739(5/23)	0.68421(13/19)
Bo_TE_108228	0.56818(25/44)	0.47059(8/17)	0.18182(4/22)	0.00000(0/19)
Bo_TE_205155	0.78571(33/42)	0.27778(5/18)	0.42857(9/21)	0.10000(2/20)
Bo_TE_226188	0.87500(14/16)	0.93333(14/15)	0.95000(19/20)	0.25000(5/20)
Bo_TE_175567	0.45238(19/42)	0.00000(0/18)	0.78947(15/19)	0.85000(17/20)
Bo_TE_226430	0.36842(14/38)	0.11765(2/17)	0.73913(17/23)	0.90000(18/20)
Bo_TE_31270	0.00000(0/42)	0.66667(12/18)	0.15000(3/20)	0.11111(2/18)
Bo_TE_223772	0.00000(0/46)	0.11111(2/18)	0.42857(9/21)	0.52632(10/19)
Bo_TE_154506	0.02174(1/46)	0.87500(14/16)	0.78947(15/19)	0.84211(16/19)
Bo_TE_163643	0.14286(6/42)	0.50000(9/18)	0.80000(16/20)	0.20000(4/20)
Bo_TE_46200	0.76923(30/39)	0.41176(7/17)	0.13043(3/23)	0.00000(0/20)
Bo_TE_215873	0.00000(0/44)	0.00000(0/18)	0.09091(2/22)	0.50000(10/20)
Bo_TE_18330	0.00000(0/45)	0.17647(3/17)	0.56522(13/23)	0.00000(0/20)
Bo_TE_41125	0.00000(0/44)	0.00000(0/17)	0.61905(13/21)	0.70588(12/17)
Bo_TE_98541	0.87805(36/41)	0.66667(12/18)	0.60000(12/20)	0.21053(4/19)
Bo_TE_95393	0.32609(15/46)	1.00000(10/10)	0.82353(14/17)	0.88235(15/17)
Bo_TE_105580	0.56818(25/44)	0.38889(7/18)	1.00000(23/23)	0.84211(16/19)
Bo_TE_212825	0.13636(6/44)	0.22222(4/18)	0.90000(18/20)	0.45000(9/20)
Bo_TE_28341	0.27273(12/44)	0.68750(11/16)	1.00000(23/23)	0.78947(15/19)
Bo_TE_159265	0.66667(24/36)	0.61111(11/18)	0.66667(12/18)	0.00000(0/18)
Bo_TE_80038	0.67442(29/43)	0.00000(0/17)	0.52174(12/23)	0.36842(7/19)
Bo_TE_88743	0.82500(33/40)	0.62500(10/16)	0.00000(0/23)	0.00000(0/20)
Bo_TE_206698	0.33333(13/39)	0.87500(14/16)	0.76190(16/21)	0.63158(12/19)
Bo_TE_136830	0.77273(34/44)	0.87500(14/16)	0.54545(12/22)	0.30000(6/20)
Bo_TE_57327	1.00000(44/44)	1.00000(19/19)	0.77273(17/22)	0.44444(8/18)
Bo_TE_141305	0.82222(37/45)	0.06250(1/16)	0.04545(1/22)	0.23529(4/17)
Bo_TE_53537	0.04545(2/44)	0.68750(11/16)	0.14286(3/21)	0.81818(9/11)
Bo_TE_3418	0.00000(0/46)	0.22222(4/18)	0.45455(10/22)	0.84211(16/19)
Bo_TE_241870	0.77500(31/40)	0.10526(2/19)	0.45455(10/22)	0.10000(2/20)
Bo_TE_121594	0.19512(8/41)	0.83333(15/18)	0.66667(14/21)	0.73684(14/19)
Bo_TE_198374	0.41463(17/41)	0.12500(2/16)	0.60000(12/20)	0.00000(0/20)

Bo_TE_191452	0.23684(9/38)	1.00000(17/17)	1.00000(22/22)	1.00000(20/20)
Bo_TE_154724	0.63636(28/44)	1.00000(16/16)	0.05556(1/18)	0.15789(3/19)
Bo_TE_19650	0.60465(26/43)	0.81250(13/16)	0.04762(1/21)	0.25000(5/20)
Bo_TE_179068	0.13953(6/43)	1.00000(14/14)	0.72727(16/22)	0.85000(17/20)
Bo_TE_235136	0.67442(29/43)	0.16667(3/18)	0.90909(20/22)	0.43750(7/16)
Bo_TE_199509	0.82222(37/45)	0.64706(11/17)	0.86957(20/23)	0.10000(2/20)
Bo_TE_148442	0.94872(37/39)	0.35294(6/17)	1.00000(21/21)	0.94118(16/17)
Bo_TE_18798	0.20455(9/44)	0.05556(1/18)	0.00000(0/23)	0.78947(15/19)
Bo_TE_88020	0.86667(39/45)	0.23529(4/17)	0.00000(0/23)	0.00000(0/20)
Bo_TE_17078	0.34146(14/41)	0.76471(13/17)	0.05000(1/20)	0.26316(5/19)
Bo_TE_128514	0.27273(12/44)	0.72222(13/18)	0.57143(12/21)	0.00000(0/20)
Bo_TE_7470	0.58140(25/43)	0.66667(10/15)	0.00000(0/21)	0.31579(6/19)
Bo_TE_145561	0.00000(0/43)	0.68750(11/16)	0.00000(0/19)	0.00000(0/19)
Bo_TE_158334	0.06667(3/45)	0.17647(3/17)	0.86364(19/22)	0.77778(14/18)
Bo_TE_182711	0.04545(2/44)	0.00000(0/19)	0.57143(12/21)	0.27778(5/18)
Bo_TE_13027	0.13953(6/43)	0.87500(14/16)	0.77778(14/18)	0.68421(13/19)
Bo_TE_13941	0.09302(4/43)	0.26667(4/15)	0.56522(13/23)	0.72222(13/18)
Bo_TE_155168	0.81818(36/44)	0.31250(5/16)	0.72727(16/22)	0.05263(1/19)
Bo_TE_113620	0.36585(15/41)	0.11111(2/18)	0.45455(10/22)	0.75000(15/20)
Bo_TE_222023	0.73171(30/41)	0.05556(1/18)	0.55000(11/20)	0.72222(13/18)
Bo_TE_56117	0.88372(38/43)	0.47059(8/17)	0.00000(0/23)	0.27778(5/18)
Bo_TE_216621	0.77273(34/44)	0.25000(4/16)	0.33333(7/21)	0.60000(12/20)
Bo_TE_121773	0.00000(0/35)	0.00000(0/12)	0.05263(1/19)	0.70588(12/17)
Bo_TE_111057	0.23256(10/43)	0.12500(2/16)	0.80952(17/21)	0.15000(3/20)
Bo_TE_99946	0.73171(30/41)	0.23529(4/17)	0.94737(18/19)	1.00000(19/19)
Bo_TE_168706	0.50000(22/44)	1.00000(15/15)	1.00000(21/21)	1.00000(18/18)
Bo_TE_127163	0.97674(42/43)	0.27778(5/18)	0.18182(4/22)	0.40000(8/20)
Bo_TE_240611	0.15385(6/39)	0.82353(14/17)	0.95652(22/23)	0.46667(7/15)
Bo_TE_107660	0.64286(27/42)	0.05556(1/18)	0.05000(1/20)	0.26316(5/19)
Bo_TE_39810	0.92857(39/42)	0.50000(9/18)	1.00000(22/22)	0.94737(18/19)
Bo_TE_81423	0.74359(29/39)	0.94737(18/19)	0.90476(19/21)	0.31579(6/19)
Bo_TE_230362	0.33333(14/42)	0.50000(8/16)	0.90000(18/20)	0.55556(10/18)
Bo_TE_123312	1.00000(44/44)	0.94444(17/18)	0.15000(3/20)	0.30000(6/20)
Bo_TE_5249	0.26190(11/42)	0.76471(13/17)	1.00000(23/23)	1.00000(19/19)
Bo_TE_234869	0.88636(39/44)	0.22222(4/18)	0.05263(1/19)	0.00000(0/17)
Bo_TE_195814	0.00000(0/46)	0.00000(0/18)	0.00000(0/22)	0.73684(14/19)
Bo_TE_122311	0.93478(43/46)	0.33333(6/18)	1.00000(23/23)	0.68421(13/19)
Bo_TE_138791	0.80556(29/36)	0.27778(5/18)	0.30000(6/20)	0.84211(16/19)
Bo_TE_116842	0.26667(12/45)	0.64706(11/17)	0.08696(2/23)	0.10526(2/19)
Bo_TE_238491	0.00000(0/45)	0.05556(1/18)	0.57895(11/19)	0.35294(6/17)
Bo_TE_162214	0.28571(12/42)	0.68750(11/16)	0.00000(0/22)	0.10526(2/19)
Bo_TE_37151	0.90476(38/42)	0.44444(8/18)	0.95455(21/22)	0.95000(19/20)
Bo_TE_1217	0.58537(24/41)	0.76471(13/17)	0.50000(11/22)	1.00000(20/20)
Bo_TE_144842	0.11111(5/45)	0.00000(0/18)	0.00000(0/23)	0.57895(11/19)
Bo_TE_32587	0.30952(13/42)	0.00000(0/18)	0.26316(5/19)	0.57895(11/19)
Bo_TE_93670	1.00000(43/43)	1.00000(3/3)	0.19048(4/21)	1.00000(20/20)
Bo_TE_55410	0.62791(27/43)	0.62500(10/16)	0.95652(22/23)	0.42105(8/19)
Bo_TE_147735	0.74359(29/39)	0.25000(4/16)	0.45455(10/22)	0.94737(18/19)
Bo_TE_57180	0.00000(0/45)	0.00000(0/19)	0.19048(4/21)	0.52632(10/19)
Bo_TE_235212	0.54762(23/42)	0.00000(0/18)	0.00000(0/23)	0.00000(0/19)
Bo_TE_50654	0.02222(1/45)	0.00000(0/18)	0.00000(0/23)	0.85000(17/20)
Bo_TE_48216	0.42857(18/42)	0.64706(11/17)	1.00000(23/23)	1.00000(20/20)
Bo_TE_43845	0.20513(8/39)	0.47059(8/17)	0.50000(10/20)	0.73684(14/19)
Bo_TE_76410	0.66667(28/42)	0.43750(7/16)	0.08696(2/23)	0.20000(4/20)
Bo_TE_141526	0.80952(34/42)	0.06667(1/15)	0.04545(1/22)	0.22222(4/18)
Bo_TE_227285	0.25000(11/44)	0.00000(0/17)	0.63636(14/22)	0.05263(1/19)

Bo_TE_238526	0.02222(1/45)	0.52941(9/17)	0.22727(5/22)	0.10000(2/20)
Bo_TE_165686	0.29268(12/41)	0.76471(13/17)	0.19048(4/21)	0.15789(3/19)
Bo_TE_194605	0.00000(0/45)	0.50000(9/18)	0.00000(0/23)	0.05263(1/19)
Bo_TE_43119	0.59091(26/44)	0.00000(0/16)	0.00000(0/22)	0.30000(6/20)
Bo_TE_132922	0.75000(30/40)	0.18750(3/16)	0.13636(3/22)	0.10526(2/19)
Bo_TE_143470	0.12195(5/41)	0.94118(16/17)	0.55000(11/20)	0.15000(3/20)
Bo_TE_213814	0.13636(6/44)	0.05556(1/18)	0.65217(15/23)	0.52632(10/19)
Bo_TE_145683	0.06667(3/45)	0.76471(13/17)	0.04348(1/23)	0.33333(6/18)
Bo_TE_44170	0.00000(0/46)	0.00000(0/18)	0.13636(3/22)	0.57895(11/19)
Bo_TE_129092	0.56098(23/41)	0.11765(2/17)	0.13043(3/23)	0.05000(1/20)
Bo_TE_77711	0.00000(0/46)	0.52941(9/17)	0.00000(0/22)	0.00000(0/20)
Bo_TE_191156	1.00000(43/43)	1.00000(17/17)	0.71429(15/21)	0.31579(6/19)
Bo_TE_103422	0.93023(40/43)	0.29412(5/17)	0.71429(15/21)	0.78947(15/19)
Bo_TE_169845	0.40476(17/42)	0.88889(16/18)	0.95238(20/21)	0.78947(15/19)
Bo_TE_45182	0.50000(22/44)	0.23529(4/17)	0.75000(15/20)	0.00000(0/18)
Bo_TE_97590	0.11628(5/43)	0.16667(3/18)	0.00000(0/22)	0.61111(11/18)
Bo_TE_113112	0.88372(38/43)	1.00000(19/19)	0.43478(10/23)	0.95000(19/20)
Bo_TE_109952	0.60465(26/43)	0.62500(10/16)	0.00000(0/23)	0.00000(0/20)
Bo_TE_34050	0.69048(29/42)	0.18750(3/16)	0.75000(15/20)	0.15789(3/19)
Bo_TE_120547	0.95238(40/42)	0.77778(14/18)	0.80000(16/20)	0.26316(5/19)
Bo_TE_70235	0.75000(30/40)	0.47059(8/17)	0.80000(16/20)	0.11111(2/18)
Bo_TE_103317	0.00000(0/41)	0.05263(1/19)	0.23529(4/17)	0.68421(13/19)
Bo_TE_17043	0.00000(0/42)	0.37500(6/16)	0.52174(12/23)	0.66667(12/18)
Bo_TE_32722	0.21053(8/38)	0.83333(15/18)	0.00000(0/21)	0.45000(9/20)
Bo_TE_184957	1.00000(46/46)	1.00000(17/17)	0.38095(8/21)	0.90000(18/20)
Bo_TE_16677	0.34146(14/41)	0.68750(11/16)	1.00000(23/23)	1.00000(20/20)
Bo_TE_105282	0.45455(20/44)	0.11765(2/17)	1.00000(23/23)	0.50000(10/20)
Bo_TE_121516	0.16279(7/43)	0.00000(0/19)	0.40000(8/20)	0.75000(15/20)
Bo_TE_125159	0.02273(1/44)	0.11765(2/17)	0.00000(0/23)	0.57895(11/19)
Bo_TE_200241	0.12195(5/41)	0.61111(11/18)	0.63636(14/22)	0.80000(16/20)
Bo_TE_2069	0.06522(3/46)	0.37500(6/16)	0.70000(14/20)	0.47368(9/19)
Bo_TE_58866	0.00000(0/46)	0.57895(11/19)	0.00000(0/23)	0.00000(0/20)
Bo_TE_5255	0.77778(35/45)	0.18750(3/16)	0.00000(0/22)	0.00000(0/20)
Bo_TE_234888	0.75000(33/44)	0.82353(14/17)	0.25000(5/20)	0.43750(7/16)
Bo_TE_20011	0.19512(8/41)	0.88889(16/18)	0.54545(12/22)	0.89474(17/19)
Bo_TE_202227	0.51220(21/41)	0.00000(0/17)	0.10526(2/19)	0.21053(4/19)
Bo_TE_117228	0.60976(25/41)	0.17647(3/17)	0.00000(0/21)	0.50000(9/18)
Bo_TE_98603	0.88095(37/42)	0.66667(12/18)	0.60000(12/20)	0.21053(4/19)
Bo_TE_132387	0.00000(0/46)	0.77778(14/18)	0.17391(4/23)	0.47059(8/17)
Bo_TE_48614	0.97727(43/44)	1.00000(19/19)	0.40000(8/20)	0.47368(9/19)
Bo_TE_54328	0.95556(43/45)	0.43750(7/16)	0.73684(14/19)	0.42105(8/19)
Bo_TE_18397	0.42857(18/42)	0.05556(1/18)	0.15000(3/20)	0.85000(17/20)
Bo_TE_81427	0.23810(10/42)	0.00000(0/18)	0.08696(2/23)	0.72222(13/18)
Bo_TE_189014	0.64286(27/42)	0.29412(5/17)	1.00000(23/23)	1.00000(20/20)
Bo_TE_236099	0.00000(0/45)	0.00000(0/17)	0.76190(16/21)	0.00000(0/20)
Bo_TE_223112	0.04545(2/44)	0.05556(1/18)	0.77273(17/22)	0.00000(0/20)
Bo_TE_24441	0.21951(9/41)	0.94118(16/17)	0.85714(18/21)	0.94737(18/19)
Bo_TE_36570	0.65909(29/44)	0.38889(7/18)	0.75000(15/20)	0.15789(3/19)
Bo_TE_226659	0.90244(37/41)	0.44444(8/18)	0.61905(13/21)	0.15789(3/19)
Bo_TE_54526	0.04348(2/46)	0.50000(9/18)	0.35000(7/20)	0.58824(10/17)
Bo_TE_188777	0.25000(11/44)	0.70588(12/17)	0.00000(0/22)	0.00000(0/19)
Bo_TE_156999	0.56818(25/44)	0.87500(14/16)	0.27273(6/22)	0.70000(14/20)
Bo_TE_137806	0.50000(20/40)	0.18750(3/16)	0.22222(4/18)	0.95000(19/20)
Bo_TE_188866	0.00000(0/46)	0.05556(1/18)	0.00000(0/22)	0.65000(13/20)
Bo_TE_145223	0.37500(15/40)	0.94737(18/19)	0.76190(16/21)	0.78947(15/19)
Bo_TE_197954	0.00000(0/46)	0.00000(0/17)	0.09524(2/21)	0.50000(9/18)

Bo_TE_205118	0.76744(33/43)	0.29412(5/17)	0.00000(0/23)	0.05263(1/19)
Bo_TE_138805	0.82222(37/45)	0.58333(7/12)	0.33333(7/21)	1.00000(16/16)
Bo_TE_95318	0.69767(30/43)	0.21053(4/19)	0.19048(4/21)	0.15000(3/20)
Bo_TE_188452	0.04651(2/43)	0.00000(0/19)	0.00000(0/22)	0.65000(13/20)
Bo_TE_32139	0.04878(2/41)	0.81250(13/16)	0.04545(1/22)	0.05000(1/20)
Bo_TE_135959	0.09302(4/43)	0.00000(0/18)	0.50000(10/20)	0.68421(13/19)
Bo_TE_34589	0.90698(39/43)	0.58824(10/17)	0.42857(9/21)	0.22222(4/18)
Bo_TE_9234	0.20930(9/43)	0.62500(10/16)	0.19048(4/21)	0.00000(0/18)
Bo_TE_30458	0.95238(40/42)	1.00000(18/18)	0.23810(5/21)	0.95000(19/20)
Bo_TE_144186	0.75000(33/44)	0.05556(1/18)	0.45000(9/20)	0.52941(9/17)
Bo_TE_120520	0.07692(3/39)	0.12500(2/16)	0.10526(2/19)	0.68750(11/16)
Bo_TE_77484	0.74359(29/39)	0.17647(3/17)	0.52381(11/21)	0.10000(2/20)
Bo_TE_57427	0.22500(9/40)	0.41176(7/17)	0.95455(21/22)	0.65000(13/20)
Bo_TE_32647	0.17949(7/39)	0.94444(17/18)	0.45000(9/20)	0.20000(4/20)
Bo_TE_5683	0.75556(34/45)	0.35294(6/17)	0.08696(2/23)	0.16667(3/18)
Bo_TE_24549	0.04545(2/44)	0.58824(10/17)	0.42105(8/19)	0.45000(9/20)
Bo_TE_150860	0.14286(6/42)	0.44444(8/18)	0.71429(15/21)	0.25000(5/20)
Bo_TE_137033	0.00000(0/45)	0.00000(0/19)	0.71429(15/21)	0.35000(7/20)
Bo_TE_153570	0.26667(12/45)	0.90000(9/10)	0.47619(10/21)	1.00000(18/18)
Bo_TE_9597	0.87879(29/33)	0.15385(2/13)	0.00000(0/23)	0.88235(15/17)
Bo_TE_20746	0.53659(22/41)	0.81250(13/16)	0.00000(0/22)	0.10000(2/20)
Bo_TE_86280	0.12195(5/41)	0.70588(12/17)	0.71429(15/21)	0.10000(2/20)
Bo_TE_86168	0.16279(7/43)	0.56250(9/16)	0.19048(4/21)	0.00000(0/19)
Bo_TE_52000	0.60976(25/41)	0.29412(5/17)	0.18182(4/22)	0.00000(0/20)
Bo_TE_67943	0.00000(0/45)	0.13333(2/15)	0.59091(13/22)	0.10000(2/20)
Bo_TE_231588	0.06818(3/44)	0.52941(9/17)	0.22727(5/22)	0.00000(0/20)
Bo_TE_54030	0.60976(25/41)	0.05882(1/17)	0.00000(0/23)	0.10526(2/19)
Bo_TE_179841	0.09091(4/44)	0.62500(10/16)	0.71429(15/21)	0.80000(16/20)
Bo_TE_182211	0.00000(0/43)	0.00000(0/19)	0.57143(12/21)	0.33333(6/18)
Bo_TE_104516	0.47727(21/44)	0.75000(12/16)	0.00000(0/23)	0.65000(13/20)
Bo_TE_143003	0.23077(9/39)	0.84615(11/13)	0.78947(15/19)	0.45000(9/20)
Bo_TE_194694	0.64286(27/42)	0.18750(3/16)	0.14286(3/21)	0.05263(1/19)
Bo_TE_228230	0.00000(0/44)	0.00000(0/18)	0.84211(16/19)	0.43750(7/16)
Bo_TE_125789	0.00000(0/44)	0.58824(10/17)	0.66667(12/18)	0.15789(3/19)
Bo_TE_194674	1.00000(40/40)	1.00000(18/18)	1.00000(20/20)	0.33333(6/18)
Bo_TE_144705	0.86667(39/45)	0.11111(2/18)	0.10000(2/20)	0.70000(14/20)
Bo_TE_189695	0.02222(1/45)	0.15789(3/19)	0.54545(12/22)	0.00000(0/20)
Bo_TE_188941	0.68293(28/41)	0.81250(13/16)	0.13636(3/22)	0.05000(1/20)
Bo_TE_66955	0.02326(1/43)	0.68750(11/16)	0.47826(11/23)	0.78947(15/19)
Bo_TE_132261	0.60976(25/41)	0.70588(12/17)	0.18182(4/22)	0.00000(0/20)
Bo_TE_180536	0.02222(1/45)	0.00000(0/18)	0.38095(8/21)	0.65000(13/20)
Bo_TE_41640	0.65116(28/43)	0.82353(14/17)	0.27273(6/22)	0.16667(3/18)
Bo_TE_93003	0.61364(27/44)	0.23529(4/17)	0.80000(16/20)	1.00000(20/20)
Bo_TE_190985	0.02326(1/43)	0.58824(10/17)	0.45000(9/20)	0.22222(4/18)
Bo_TE_96837	0.56818(25/44)	0.23529(4/17)	0.77273(17/22)	0.00000(0/20)
Bo_TE_22890	0.11364(5/44)	0.58824(10/17)	0.00000(0/21)	0.05882(1/17)
Bo_TE_194578	0.61905(26/42)	0.41176(7/17)	0.86364(19/22)	0.21053(4/19)
Bo_TE_630	0.00000(0/44)	0.00000(0/18)	0.00000(0/21)	0.60000(12/20)
Bo_TE_101786	0.06977(3/43)	0.73684(14/19)	0.31818(7/22)	0.88889(16/18)
Bo_TE_178418	0.78571(33/42)	0.33333(6/18)	0.05263(1/19)	0.22222(4/18)
Bo_TE_149281	0.00000(0/45)	0.05556(1/18)	0.80952(17/21)	0.05000(1/20)
Bo_TE_105817	0.95349(41/43)	0.37500(6/16)	0.56522(13/23)	0.26316(5/19)
Bo_TE_129898	0.39024(16/41)	0.50000(9/18)	0.10000(2/20)	0.72222(13/18)
Bo_TE_116439	0.00000(0/41)	0.00000(0/18)	0.84211(16/19)	0.47368(9/19)
Bo_TE_9668	0.08889(4/45)	0.50000(9/18)	0.00000(0/23)	0.00000(0/20)
Bo_TE_107032	0.15556(7/45)	0.29412(5/17)	0.77273(17/22)	0.89474(17/19)

Bo_TE_58884	0.00000(0/43)	0.17647(3/17)	0.70000(14/20)	0.00000(0/19)
Bo_TE_49738	0.57895(22/38)	0.17647(3/17)	0.80952(17/21)	0.94444(17/18)
Bo_TE_5204	0.67500(27/40)	0.17647(3/17)	0.00000(0/23)	0.05263(1/19)
Bo_TE_63848	0.46154(18/39)	0.70588(12/17)	0.78947(15/19)	0.05556(1/18)
Bo_TE_52677	0.15385(6/39)	0.05556(1/18)	0.81818(18/22)	0.00000(0/18)
Bo_TE_17252	0.06522(3/46)	0.50000(8/16)	0.00000(0/22)	0.15789(3/19)
Bo_TE_91651	0.07143(3/42)	0.27778(5/18)	0.59091(13/22)	0.15789(3/19)
Bo_TE_31563	0.20000(8/40)	0.58824(10/17)	0.72727(16/22)	0.33333(6/18)
Bo_TE_154719	0.29268(12/41)	0.00000(0/18)	0.80952(17/21)	0.63158(12/19)
Bo_TE_97581	0.06977(3/43)	0.00000(0/18)	0.00000(0/23)	0.50000(9/18)
Bo_TE_182120	0.45000(18/40)	0.50000(9/18)	0.95238(20/21)	0.78947(15/19)
Bo_TE_137643	0.02174(1/46)	0.00000(0/18)	0.63158(12/19)	0.05000(1/20)
Bo_TE_222252	0.97674(42/43)	0.31579(6/19)	1.00000(23/23)	0.95000(19/20)
Bo_TE_197592	1.00000(45/45)	0.89474(17/19)	0.82609(19/23)	0.31579(6/19)
Bo_TE_83942	0.69048(29/42)	0.33333(5/15)	0.00000(0/21)	0.00000(0/20)
Bo_TE_227696	0.02326(1/43)	0.05556(1/18)	0.36364(8/22)	0.52632(10/19)
Bo_TE_21174	0.62791(27/43)	0.00000(0/14)	0.00000(0/23)	0.00000(0/20)
Bo_TE_216459	0.26829(11/41)	0.06250(1/16)	0.68182(15/22)	0.27778(5/18)
Bo_TE_49228	0.11628(5/43)	0.88889(16/18)	0.23810(5/21)	0.33333(6/18)
Bo_TE_148719	0.50000(21/42)	0.56250(9/16)	0.00000(0/23)	0.00000(0/20)
Bo_TE_222576	0.21951(9/41)	0.05263(1/19)	0.71429(15/21)	0.15000(3/20)
Bo_TE_205872	0.81818(36/44)	0.73333(11/15)	0.00000(0/22)	0.05263(1/19)
Bo_TE_62864	0.97826(45/46)	0.27778(5/18)	0.54545(12/22)	1.00000(20/20)
Bo_TE_191012	0.58974(23/39)	0.23529(4/17)	0.76190(16/21)	0.50000(10/20)
Bo_TE_81961	0.69767(30/43)	0.52941(9/17)	0.23810(5/21)	0.16667(3/18)
Bo_TE_33678	0.52500(21/40)	0.61538(8/13)	0.04348(1/23)	0.00000(0/20)
Bo_TE_174980	0.04762(2/42)	0.31250(5/16)	0.13636(3/22)	0.62500(10/16)
Bo_TE_8662	0.13953(6/43)	0.23529(4/17)	0.61905(13/21)	0.77778(14/18)
Bo_TE_137547	0.24390(10/41)	0.16667(3/18)	0.86364(19/22)	0.68421(13/19)
Bo_TE_169837	0.18182(8/44)	0.37500(6/16)	0.81818(18/22)	0.10000(2/20)
Bo_TE_80083	0.79070(34/43)	0.23529(4/17)	1.00000(21/21)	1.00000(18/18)
Bo_TE_206594	0.04762(2/42)	0.18750(3/16)	0.04762(1/21)	0.73684(14/19)
Bo_TE_45640	0.39535(17/43)	0.94118(16/17)	0.80952(17/21)	1.00000(20/20)
Bo_TE_237247	0.00000(0/44)	0.00000(0/19)	0.45455(10/22)	0.63158(12/19)
Bo_TE_141416	0.11905(5/42)	0.85714(12/14)	0.95238(20/21)	0.78947(15/19)
Bo_TE_143727	0.38095(16/42)	0.05556(1/18)	0.45000(9/20)	0.57895(11/19)
Bo_TE_117341	0.97727(43/44)	0.18750(3/16)	0.31818(7/22)	0.44444(8/18)
Bo_TE_214819	0.82927(34/41)	0.06667(1/15)	0.00000(0/22)	0.05000(1/20)
Bo_TE_210882	0.53659(22/41)	0.22222(4/18)	0.71429(15/21)	0.90000(18/20)
Bo_TE_227469	0.20930(9/43)	0.16667(3/18)	0.15000(3/20)	0.70000(14/20)
Bo_TE_201788	0.20000(8/40)	0.55556(10/18)	0.72727(16/22)	0.20000(4/20)
Bo_TE_201702	0.60976(25/41)	0.26316(5/19)	0.61111(11/18)	0.10000(2/20)
Bo_TE_84595	1.00000(26/26)	1.00000(17/17)	0.00000(0/17)	0.11765(2/17)
Bo_TE_55280	0.04348(2/46)	0.50000(8/16)	0.00000(0/22)	0.05000(1/20)
Bo_TE_40936	0.00000(0/44)	0.00000(0/18)	0.00000(0/23)	0.52941(9/17)
Bo_TE_126925	0.13636(6/44)	0.70588(12/17)	0.00000(0/23)	0.00000(0/19)
Bo_TE_121685	0.33333(14/42)	0.11765(2/17)	0.95455(21/22)	0.15789(3/19)
Bo_TE_201125	0.00000(0/44)	0.76471(13/17)	0.00000(0/2)	?(?/?)
Bo_TE_123791	0.00000(0/46)	0.68421(13/19)	0.00000(0/22)	0.05000(1/20)
Bo_TE_134568	0.68293(28/41)	0.82353(14/17)	0.10526(2/19)	0.05556(1/18)
Bo_TE_136432	0.02222(1/45)	0.00000(0/18)	0.35000(7/20)	0.52632(10/19)
Bo_TE_231070	0.76744(33/43)	0.00000(0/16)	0.16667(3/18)	0.05263(1/19)
Bo_TE_200548	0.26829(11/41)	0.52941(9/17)	0.90909(20/22)	0.80000(16/20)
Bo_TE_216561	0.53659(22/41)	0.55556(10/18)	0.15789(3/19)	0.72222(13/18)
Bo_TE_121717	0.86667(39/45)	1.00000(18/18)	0.95455(21/22)	0.10526(2/19)
Bo_TE_153828	1.00000(29/29)	1.00000(19/19)	0.65000(13/20)	0.35000(7/20)

Bo_TE_153981	0.52500(21/40)	0.16667(3/18)	0.76190(16/21)	0.50000(10/20)
Bo_TE_5670	0.79070(34/43)	0.35294(6/17)	0.08696(2/23)	0.23529(4/17)
Bo_TE_27504	0.22727(10/44)	0.94737(18/19)	0.75000(9/12)	1.00000(20/20)
Bo_TE_129750	0.27500(11/40)	0.87500(14/16)	0.90000(18/20)	0.35000(7/20)
Bo_TE_234982	0.53333(24/45)	0.29412(5/17)	0.00000(0/22)	0.35000(7/20)
Bo_TE_71286	0.11111(5/45)	0.88889(16/18)	0.75000(15/20)	0.47059(8/17)
Bo_TE_133582	0.15789(6/38)	0.20000(3/15)	0.66667(14/21)	0.50000(10/20)
Bo_TE_78870	1.00000(41/41)	1.00000(18/18)	0.95000(19/20)	0.26316(5/19)
Bo_TE_17192	0.04545(2/44)	0.41176(7/17)	0.59091(13/22)	0.47368(9/19)
Bo_TE_163788	0.02273(1/44)	0.50000(9/18)	0.23810(5/21)	0.00000(0/19)
Bo_TE_18467	0.00000(0/45)	0.00000(0/17)	0.63636(14/22)	0.89474(17/19)
Bo_TE_45253	0.00000(0/45)	0.10526(2/19)	0.61905(13/21)	0.00000(0/19)
Bo_TE_119455	0.56098(23/41)	0.12500(2/16)	0.00000(0/22)	0.50000(9/18)
Bo_TE_226236	0.41463(17/41)	0.77778(14/18)	0.00000(0/23)	0.05000(1/20)
Bo_TE_93711	0.95455(42/44)	0.11765(2/17)	1.00000(21/21)	1.00000(20/20)
Bo_TE_149187	0.97500(39/40)	0.88889(16/18)	0.20000(4/20)	0.52941(9/17)
Bo_TE_10569	0.88095(37/42)	0.31250(5/16)	0.00000(0/20)	0.05556(1/18)
Bo_TE_220950	0.76744(33/43)	0.00000(0/19)	0.00000(0/23)	0.15789(3/19)
Bo_TE_225964	0.88636(39/44)	0.38889(7/18)	1.00000(23/23)	1.00000(19/19)
Bo_TE_108878	0.00000(0/45)	0.33333(6/18)	0.52381(11/21)	0.10000(2/20)
Bo_TE_165776	0.59091(26/44)	0.94118(16/17)	0.18182(4/22)	0.68421(13/19)
Bo_TE_226566	0.95556(43/45)	0.27778(5/18)	0.72727(16/22)	0.95000(19/20)
Bo_TE_205858	0.18421(7/38)	0.25000(4/16)	1.00000(22/22)	0.94737(18/19)
Bo_TE_15798	0.88372(38/43)	0.70588(12/17)	0.60000(12/20)	0.15789(3/19)
Bo_TE_215092	0.00000(0/43)	0.52941(9/17)	0.00000(0/22)	0.00000(0/20)
Bo_TE_133348	0.52500(21/40)	0.64706(11/17)	0.00000(0/23)	0.42105(8/19)
Bo_TE_74592	1.00000(42/42)	0.62500(10/16)	0.36364(8/22)	0.77778(14/18)
Bo_TE_94385	0.00000(0/46)	0.11111(2/18)	0.42857(9/21)	0.73684(14/19)
Bo_TE_91241	0.79545(35/44)	0.94118(16/17)	0.60000(12/20)	0.30000(6/20)
Bo_TE_108274	0.21951(9/41)	0.66667(12/18)	0.76190(16/21)	0.63158(12/19)
Bo_TE_104971	0.34146(14/41)	1.00000(16/16)	0.70000(14/20)	0.40000(8/20)
Bo_TE_240243	0.48780(20/41)	0.31250(5/16)	0.57143(12/21)	0.90000(18/20)
Bo_TE_67748	0.85366(35/41)	0.83333(15/18)	0.77273(17/22)	0.15000(3/20)
Bo_TE_228154	0.00000(0/46)	0.00000(0/18)	0.80000(16/20)	0.47368(9/19)
Bo_TE_59804	0.00000(0/46)	0.27778(5/18)	0.00000(0/22)	0.65000(13/20)
Bo_TE_234923	0.02174(1/46)	0.05556(1/18)	0.04545(1/22)	0.52632(10/19)
Bo_TE_148834	0.68293(28/41)	0.33333(6/18)	0.00000(0/21)	0.00000(0/19)
Bo_TE_27523	0.00000(0/40)	0.00000(0/19)	0.57143(12/21)	0.00000(0/16)
Bo_TE_201935	0.83333(35/42)	0.35294(6/17)	0.00000(0/23)	0.00000(0/20)
Bo_TE_215875	0.35556(16/45)	0.61111(11/18)	0.00000(0/22)	0.00000(0/20)
Bo_TE_20242	0.38095(16/42)	0.17647(3/17)	0.86364(19/22)	0.82353(14/17)
Bo_TE_131786	0.04444(2/45)	0.05556(1/18)	0.63636(14/22)	0.63158(12/19)
Bo_TE_167720	0.91176(31/34)	0.94444(17/18)	0.38095(8/21)	0.94737(18/19)
Bo_TE_189685	0.02174(1/46)	0.15789(3/19)	0.55000(11/20)	0.00000(0/19)
Bo_TE_39896	0.30769(12/39)	0.47059(8/17)	0.85714(18/21)	0.83333(15/18)
Bo_TE_120563	0.44737(17/38)	0.22222(4/18)	0.73684(14/19)	0.00000(0/20)
Bo_TE_124060	0.61364(27/44)	0.73684(14/19)	0.90909(20/22)	0.27778(5/18)
Bo_TE_103217	0.59524(25/42)	0.61111(11/18)	0.36842(7/19)	0.05556(1/18)
Bo_TE_162939	0.29545(13/44)	0.00000(0/17)	0.38095(8/21)	0.80000(16/20)
Bo_TE_43461	0.88372(38/43)	0.58824(10/17)	0.00000(0/21)	0.47368(9/19)
Bo_TE_165139	0.93333(42/45)	0.35294(6/17)	0.73684(14/19)	1.00000(20/20)
Bo_TE_169363	0.77778(35/45)	0.26316(5/19)	0.54545(12/22)	0.36842(7/19)
Bo_TE_80703	0.34091(15/44)	0.18750(3/16)	0.90476(19/21)	0.94737(18/19)
Bo_TE_34436	0.19048(8/42)	0.26667(4/15)	0.13636(3/22)	0.88235(15/17)
Bo_TE_27228	0.07500(3/40)	0.16667(3/18)	0.38095(8/21)	0.78947(15/19)
Bo_TE_143618	0.69767(30/43)	0.37500(6/16)	0.00000(0/23)	0.05000(1/20)

Bo_TE_21059	0.19565(9/46)	0.88235(15/17)	0.54545(12/22)	0.90000(18/20)
Bo_TE_89502	0.43590(17/39)	0.43750(7/16)	0.76190(16/21)	1.00000(18/18)
Bo_TE_145830	0.56818(25/44)	0.05556(1/18)	0.52632(10/19)	0.00000(0/20)
Bo_TE_7124	0.88095(37/42)	0.68750(11/16)	0.30000(6/20)	0.20000(3/15)
Bo_TE_24034	0.44444(20/45)	0.70588(12/17)	0.20000(4/20)	0.10000(2/20)
Bo_TE_74295	0.81395(35/43)	0.18750(3/16)	0.34783(8/23)	0.30000(6/20)
Bo_TE_154425	0.97778(44/45)	0.11765(2/17)	1.00000(22/22)	1.00000(20/20)
Bo_TE_235642	0.72727(32/44)	0.11111(2/18)	1.00000(23/23)	0.65000(13/20)
Bo_TE_104634	0.19512(8/41)	0.22222(4/18)	0.72727(16/22)	0.00000(0/19)
Bo_TE_36550	0.22500(9/40)	1.00000(17/17)	0.58824(10/17)	0.73684(14/19)
Bo_TE_131730	0.34884(15/43)	0.52941(9/17)	0.00000(0/19)	0.05556(1/18)
Bo_TE_128657	0.06818(3/44)	0.55556(10/18)	0.00000(0/23)	0.00000(0/20)
Bo_TE_163583	0.79070(34/43)	1.00000(16/16)	0.82609(19/23)	0.35000(7/20)
Bo_TE_8468	0.44444(20/45)	0.11765(2/17)	0.35000(7/20)	0.68421(13/19)
Bo_TE_61068	0.21951(9/41)	0.10526(2/19)	0.31579(6/19)	0.63158(12/19)
Bo_TE_223932	0.87805(36/41)	0.35294(6/17)	0.68182(15/22)	1.00000(20/20)
Bo_TE_127394	0.73171(30/41)	0.27778(5/18)	0.00000(0/22)	0.05000(1/20)
Bo_TE_174310	1.00000(44/44)	1.00000(18/18)	0.80952(17/21)	0.21053(4/19)
Bo_TE_235664	0.79545(35/44)	0.89474(17/19)	0.08696(2/23)	0.52632(10/19)
Bo_TE_180492	0.54762(23/42)	0.61111(11/18)	0.31818(7/22)	0.00000(0/19)
Bo_TE_134154	0.51163(22/43)	0.75000(12/16)	0.00000(0/23)	0.57895(11/19)
Bo_TE_43139	0.04545(2/44)	0.58824(10/17)	0.00000(0/23)	0.00000(0/20)
Bo_TE_158986	0.60000(27/45)	0.47059(8/17)	0.95652(22/23)	0.25000(5/20)
Bo_TE_141403	0.17778(8/45)	0.94118(16/17)	0.95000(19/20)	0.77778(14/18)
Bo_TE_208799	0.87500(35/40)	1.00000(14/14)	0.72222(13/18)	0.05263(1/19)
Bo_TE_150856	0.85714(36/42)	0.35294(6/17)	0.00000(0/22)	0.22222(4/18)
Bo_TE_205649	0.76744(33/43)	0.35294(6/17)	0.90476(19/21)	1.00000(20/20)
Bo_TE_143683	0.97727(43/44)	0.44444(8/18)	1.00000(22/22)	0.30000(6/20)
Bo_TE_191967	0.67500(27/40)	0.42857(6/14)	0.80000(16/20)	0.11765(2/17)
Bo_TE_101089	0.72093(31/43)	0.26667(4/15)	0.85714(18/21)	0.10526(2/19)
Bo_TE_239898	0.25641(10/39)	0.11765(2/17)	0.90476(19/21)	0.21053(4/19)
Bo_TE_148297	0.59091(26/44)	0.76471(13/17)	0.04545(1/22)	0.00000(0/20)
Bo_TE_55642	0.56410(22/39)	0.78947(15/19)	0.65000(13/20)	0.27778(5/18)
Bo_TE_41212	0.92857(39/42)	0.17647(3/17)	0.42857(9/21)	0.38889(7/18)
Bo_TE_59926	0.97826(45/46)	0.94444(17/18)	0.68182(15/22)	0.35000(7/20)
Bo_TE_126176	0.35714(15/42)	0.80000(12/15)	0.86364(19/22)	0.05263(1/19)
Bo_TE_120509	0.65789(25/38)	0.76471(13/17)	0.76190(16/21)	0.26316(5/19)
Bo_TE_230369	0.00000(0/46)	0.52941(9/17)	0.09091(2/22)	0.58824(10/17)
Bo_TE_112886	0.26190(11/42)	0.55556(10/18)	0.00000(0/23)	0.00000(0/20)
Bo_TE_129404	0.80952(34/42)	0.06250(1/16)	0.73684(14/19)	0.31579(6/19)
Bo_TE_108174	0.88636(39/44)	0.00000(0/18)	0.05556(1/18)	0.52941(9/17)
Bo_TE_148567	0.02326(1/43)	0.17647(3/17)	0.00000(0/23)	0.57895(11/19)
Bo_TE_200845	0.30952(13/42)	0.72222(13/18)	1.00000(22/22)	0.68421(13/19)
Bo_TE_219412	0.02222(1/45)	0.10526(2/19)	0.61905(13/21)	0.57895(11/19)
Bo_TE_57154	0.00000(0/46)	0.00000(0/19)	0.21739(5/23)	0.58824(10/17)
Bo_TE_179949	0.79545(35/44)	0.64286(9/14)	0.00000(0/23)	0.05000(1/20)
Bo_TE_202661	0.97674(42/43)	0.55556(10/18)	0.72727(16/22)	0.33333(6/18)
Bo_TE_67912	0.67442(29/43)	0.06250(1/16)	0.04545(1/22)	0.29412(5/17)
Bo_TE_99199	0.00000(0/44)	0.55556(10/18)	0.04348(1/23)	0.00000(0/19)
Bo_TE_102058	0.17500(7/40)	0.58824(10/17)	0.69565(16/23)	0.65000(13/20)
Bo_TE_58418	0.07143(3/42)	0.58824(10/17)	0.23810(5/21)	0.75000(15/20)
Bo_TE_130135	0.91111(41/45)	0.61111(11/18)	0.33333(7/21)	0.22222(4/18)
Bo_TE_213314	0.67500(27/40)	0.00000(0/18)	0.21739(5/23)	0.05263(1/19)
Bo_TE_155374	0.42857(18/42)	0.35294(6/17)	0.25000(5/20)	0.75000(15/20)
Bo_TE_97803	0.75610(31/41)	0.11111(2/18)	0.95455(21/22)	0.82353(14/17)
Bo_TE_234088	0.25000(11/44)	0.00000(0/19)	0.00000(0/21)	0.50000(10/20)

Bo_TE_164881	0.20000(8/40)	0.52941(9/17)	0.58824(10/17)	0.72222(13/18)
Bo_TE_178285	0.10000(4/40)	0.52941(9/17)	0.80952(17/21)	0.75000(15/20)
Bo_TE_155527	0.31818(14/44)	0.27778(5/18)	0.15000(3/20)	0.80000(16/20)
Bo_TE_205652	0.76744(33/43)	0.35294(6/17)	0.90909(20/22)	1.00000(20/20)
Bo_TE_149249	0.02500(1/40)	0.38889(7/18)	0.17647(3/17)	0.52632(10/19)
Bo_TE_132447	0.09524(4/42)	0.00000(0/17)	0.77273(17/22)	0.52941(9/17)
Bo_TE_131096	0.54762(23/42)	0.00000(0/15)	0.04348(1/23)	0.21053(4/19)
Bo_TE_105904	0.35000(14/40)	0.82353(14/17)	1.00000(23/23)	0.90000(18/20)
Bo_TE_236033	0.79487(31/39)	0.23529(4/17)	0.00000(0/23)	0.00000(0/17)
Bo_TE_54619	0.55814(24/43)	0.47368(9/19)	0.00000(0/23)	0.38889(7/18)
Bo_TE_169487	0.13953(6/43)	0.00000(0/17)	0.81818(18/22)	0.31579(6/19)
Bo_TE_148435	0.04444(2/45)	0.57895(11/19)	0.00000(0/22)	0.00000(0/19)
Bo_TE_120808	0.51163(22/43)	0.31579(6/19)	0.09091(2/22)	0.00000(0/20)
Bo_TE_103122	0.11111(5/45)	0.11111(2/18)	0.76190(16/21)	0.63158(12/19)
Bo_TE_155879	0.28261(13/46)	0.11765(2/17)	0.45000(9/20)	0.84211(16/19)
Bo_TE_182728	0.34884(15/43)	0.55556(10/18)	1.00000(21/21)	0.95000(19/20)
Bo_TE_238816	0.65000(26/40)	0.11765(2/17)	0.42857(9/21)	0.20000(4/20)
Bo_TE_157903	0.00000(0/44)	0.77778(14/18)	0.45000(9/20)	0.50000(10/20)
Bo_TE_197737	0.46512(20/43)	0.77778(14/18)	0.09091(2/22)	0.31579(6/19)
Bo_TE_95429	0.04545(2/44)	0.00000(0/18)	0.57895(11/19)	0.73684(14/19)
Bo_TE_216227	1.00000(45/45)	0.88889(16/18)	0.47826(11/23)	0.80000(16/20)
Bo_TE_116154	0.02273(1/44)	0.41176(7/17)	0.45455(10/22)	0.55000(11/20)
Bo_TE_211348	0.72093(31/43)	0.07143(1/14)	0.00000(0/20)	0.85000(17/20)
Bo_TE_131992	0.29545(13/44)	0.82353(14/17)	0.38095(8/21)	0.00000(0/19)
Bo_TE_232368	1.00000(45/45)	0.72222(13/18)	0.60870(14/23)	0.50000(10/20)
Bo_TE_11949	0.00000(0/42)	0.00000(0/18)	0.57143(12/21)	0.89474(17/19)
Bo_TE_119560	0.36364(16/44)	1.00000(16/16)	0.31818(7/22)	0.60000(12/20)
Bo_TE_26697	0.60000(24/40)	0.37500(6/16)	0.95652(22/23)	1.00000(20/20)
Bo_TE_8046	0.95652(44/46)	1.00000(17/17)	0.47368(9/19)	0.50000(10/20)
Bo_TE_121550	0.04444(2/45)	0.70588(12/17)	0.00000(0/23)	0.05000(1/20)
Bo_TE_236658	0.02174(1/46)	0.46667(7/15)	0.80000(16/20)	0.05000(1/20)
Bo_TE_229883	0.32558(14/43)	0.25000(4/16)	0.57143(12/21)	0.78947(15/19)
Bo_TE_96327	0.00000(0/45)	0.00000(0/17)	0.36842(7/19)	0.83333(15/18)
Bo_TE_80647	0.35714(15/42)	0.94118(16/17)	0.50000(11/22)	1.00000(20/20)
Bo_TE_216148	0.97778(44/45)	0.94444(17/18)	0.90476(19/21)	0.47368(9/19)
Bo_TE_151663	0.06522(3/46)	0.05882(1/17)	0.52381(11/21)	0.58824(10/17)
Bo_TE_169442	0.71111(32/45)	0.10526(2/19)	0.09091(2/22)	0.21053(4/19)
Bo_TE_129160	0.97297(36/37)	0.57143(4/7)	0.25000(5/20)	1.00000(18/18)
Bo_TE_192000	0.83721(36/43)	0.05882(1/17)	0.57143(12/21)	0.00000(0/18)
Bo_TE_68860	0.68889(31/45)	0.11765(2/17)	0.00000(0/23)	0.27778(5/18)
Bo_TE_171285	0.53488(23/43)	0.15789(3/19)	0.10526(2/19)	0.73684(14/19)
Bo_TE_220778	1.00000(39/39)	0.82353(14/17)	0.83333(15/18)	0.18750(3/16)
Bo_TE_61079	0.74419(32/43)	0.88235(15/17)	0.68182(15/22)	0.35000(7/20)
Bo_TE_43733	0.85366(35/41)	0.88889(16/18)	0.00000(0/21)	0.60000(12/20)
Bo_TE_80161	0.00000(0/45)	0.10526(2/19)	0.45455(10/22)	0.58824(10/17)
Bo_TE_112827	0.25641(10/39)	0.50000(9/18)	0.00000(0/22)	0.05000(1/20)
Bo_TE_193666	0.65116(28/43)	0.38889(7/18)	0.76190(16/21)	0.95000(19/20)
Bo_TE_21227	0.06667(3/45)	0.50000(8/16)	0.30000(6/20)	0.63158(12/19)
Bo_TE_204068	0.67442(29/43)	0.20000(3/15)	0.76190(16/21)	0.15000(3/20)
Bo_TE_234776	0.13333(6/45)	0.78947(15/19)	0.95652(22/23)	0.90000(18/20)
Bo_TE_12920	0.09091(4/44)	0.82353(14/17)	0.82609(19/23)	0.61111(11/18)
Bo_TE_216249	0.28889(13/45)	0.05882(1/17)	0.23810(5/21)	0.88889(16/18)
Bo_TE_103899	0.18182(8/44)	0.33333(6/18)	0.95455(21/22)	0.77778(14/18)
Bo_TE_227512	0.67568(25/37)	0.17647(3/17)	0.40000(8/20)	0.84211(16/19)
Bo_TE_45179	0.52273(23/44)	0.35294(6/17)	0.85714(18/21)	0.00000(0/20)
Bo_TE_35839	0.00000(0/46)	0.05882(1/17)	0.55000(11/20)	0.05000(1/20)

Bo_TE_177825	0.02222(1/45)	0.05556(1/18)	0.63636(14/22)	0.20000(4/20)
Bo_TE_46341	0.09091(4/44)	0.70588(12/17)	0.86957(20/23)	0.57895(11/19)
Bo_TE_9241	0.80952(34/42)	0.37500(6/16)	0.81818(18/22)	1.00000(20/20)
Bo_TE_98243	0.00000(0/45)	0.00000(0/19)	0.57143(12/21)	0.05000(1/20)
Bo_TE_225196	0.37500(15/40)	0.66667(12/18)	0.05000(1/20)	0.35000(7/20)
Bo_TE_177795	0.80488(33/41)	0.06250(1/16)	0.78947(15/19)	0.33333(6/18)
Bo_TE_45320	0.57895(22/38)	0.43750(7/16)	0.85714(18/21)	0.00000(0/19)
Bo_TE_43518	0.23684(9/38)	0.42105(8/19)	1.00000(21/21)	0.52632(10/19)
Bo_TE_212226	0.17391(8/46)	0.68750(11/16)	0.54545(12/22)	0.00000(0/20)
Bo_TE_70214	0.25000(10/40)	0.50000(9/18)	0.19048(4/21)	0.77778(14/18)
Bo_TE_227939	0.07500(3/40)	0.00000(0/16)	0.71429(15/21)	0.70588(12/17)
Bo_TE_131217	0.15556(7/45)	0.82353(14/17)	0.20000(4/20)	0.58824(10/17)
Bo_TE_188841	0.11111(5/45)	0.68750(11/16)	0.40000(8/20)	0.00000(0/20)
Bo_TE_169402	0.00000(0/45)	0.73684(14/19)	0.73684(14/19)	0.42105(8/19)
Bo_TE_193190	0.73171(30/41)	0.22222(4/18)	0.00000(0/23)	0.70000(14/20)
Bo_TE_133018	0.14634(6/41)	0.87500(14/16)	0.95455(21/22)	0.50000(10/20)
Bo_TE_210010	0.04545(2/44)	0.56250(9/16)	0.00000(0/23)	0.00000(0/20)
Bo_TE_224313	0.69048(29/42)	0.43750(7/16)	1.00000(22/22)	0.94737(18/19)
Bo_TE_83934	0.11905(5/42)	0.00000(0/17)	0.61905(13/21)	0.80000(16/20)
Bo_TE_7424	0.42500(17/40)	0.06250(1/16)	0.85000(17/20)	0.57895(11/19)
Bo_TE_147774	0.04762(2/42)	0.05556(1/18)	0.60000(12/20)	0.15000(3/20)
Bo_TE_33975	0.33333(14/42)	0.56250(9/16)	0.28571(6/21)	0.84211(16/19)
Bo_TE_155844	0.62791(27/43)	0.35294(6/17)	0.00000(0/23)	0.26316(5/19)
Bo_TE_70752	0.09524(4/42)	0.05263(1/19)	0.17391(4/23)	0.57895(11/19)
Bo_TE_153626	0.91304(42/46)	0.44444(8/18)	0.54545(12/22)	0.17647(3/17)
Bo_TE_110246	0.00000(0/44)	0.11111(2/18)	0.22727(5/22)	0.83333(15/18)
Bo_TE_140905	0.23256(10/43)	0.88889(16/18)	0.90909(20/22)	0.84211(16/19)
Bo_TE_154164	0.02174(1/46)	0.00000(0/18)	0.77273(17/22)	0.57895(11/19)
Bo_TE_68564	0.50000(21/42)	0.78947(15/19)	1.00000(21/21)	0.95000(19/20)
Bo_TE_90323	0.04444(2/45)	0.05263(1/19)	0.81818(18/22)	0.70000(14/20)
Bo_TE_129218	0.34146(14/41)	0.00000(0/17)	0.63636(14/22)	0.00000(0/20)
Bo_TE_119763	0.32558(14/43)	0.93750(15/16)	1.00000(22/22)	0.66667(12/18)
Bo_TE_92612	0.86364(38/44)	0.41176(7/17)	0.00000(0/22)	0.05000(1/20)
Bo_TE_52143	0.00000(0/46)	0.00000(0/18)	0.50000(10/20)	0.47368(9/19)
Bo_TE_35066	0.90698(39/43)	0.92308(12/13)	0.15789(3/19)	0.14286(2/14)
Bo_TE_162573	0.54762(23/42)	0.06250(1/16)	0.00000(0/22)	0.00000(0/20)
Bo_TE_80209	1.00000(45/45)	1.00000(18/18)	0.95652(22/23)	0.27778(5/18)
Bo_TE_22944	1.00000(36/36)	1.00000(18/18)	0.63158(12/19)	0.29412(5/17)
Bo_TE_232214	0.68293(28/41)	0.15789(3/19)	0.19048(4/21)	0.15000(3/20)
Bo_TE_148680	0.57500(23/40)	0.44444(8/18)	0.00000(0/22)	0.00000(0/19)
Bo_TE_117159	0.71429(30/42)	0.16667(3/18)	0.00000(0/23)	0.52941(9/17)
Bo_TE_49513	0.02222(1/45)	0.60000(9/15)	0.54545(12/22)	0.75000(15/20)
Bo_TE_181845	0.78049(32/41)	1.00000(18/18)	0.45455(10/22)	0.81250(13/16)
Bo_TE_66074	1.00000(36/36)	1.00000(15/15)	0.10526(2/19)	0.70588(12/17)
Bo_TE_34017	0.68293(28/41)	0.40000(6/15)	0.00000(0/7)	0.10526(2/19)
Bo_TE_218982	0.72973(27/37)	0.17647(3/17)	0.85000(17/20)	0.85000(17/20)
Bo_TE_154878	0.71429(30/42)	0.12500(2/16)	0.77273(17/22)	0.94737(18/19)
Bo_TE_112232	0.50000(22/44)	0.00000(0/19)	0.00000(0/23)	0.00000(0/20)
Bo_TE_100106	0.11111(5/45)	0.05263(1/19)	0.68182(15/22)	0.47368(9/19)
Bo_TE_17757	0.31818(14/44)	0.62500(10/16)	0.00000(0/22)	0.00000(0/19)
Bo_TE_136106	0.45238(19/42)	0.83333(15/18)	1.00000(22/22)	1.00000(20/20)
Bo_TE_122834	0.76190(32/42)	0.18750(3/16)	0.76190(16/21)	0.89474(17/19)
Bo_TE_201688	0.40000(16/40)	0.50000(9/18)	0.00000(0/22)	0.00000(0/20)
Bo_TE_91216	0.87500(35/40)	0.05556(1/18)	0.00000(0/19)	0.25000(4/16)
Bo_TE_18710	0.02222(1/45)	0.52941(9/17)	0.04545(1/22)	0.00000(0/18)
Bo_TE_24215	0.86667(39/45)	1.00000(17/17)	0.47826(11/23)	0.27778(5/18)

Bo_TE_63600	0.06977(3/43)	0.70588(12/17)	0.13043(3/23)	0.00000(0/20)
Bo_TE_199935	0.02381(1/42)	0.11111(2/18)	0.50000(11/22)	0.00000(0/20)
Bo_TE_60179	0.34211(13/38)	0.40000(4/10)	0.47619(10/21)	1.00000(19/19)
Bo_TE_190912	0.35897(14/39)	0.81250(13/16)	0.00000(0/22)	0.66667(10/15)
Bo_TE_63664	0.34146(14/41)	0.76471(13/17)	0.00000(0/22)	0.05263(1/19)
Bo_TE_117980	0.00000(0/45)	0.00000(0/18)	0.09091(2/22)	0.63158(12/19)
Bo_TE_30808	0.66667(26/39)	0.33333(6/18)	0.73684(14/19)	1.00000(20/20)
Bo_TE_207024	0.09091(4/44)	0.00000(0/19)	0.47368(9/19)	0.50000(9/18)
Bo_TE_228009	1.00000(46/46)	1.00000(18/18)	0.21053(4/19)	0.55556(10/18)
Bo_TE_49232	0.97727(43/44)	0.94118(16/17)	0.31818(7/22)	1.00000(19/19)
Bo_TE_144234	0.95122(39/41)	0.17647(3/17)	0.86957(20/23)	1.00000(20/20)
Bo_TE_198485	0.10870(5/46)	0.31579(6/19)	0.38095(8/21)	0.95000(19/20)
Bo_TE_156080	0.16667(7/42)	0.77778(14/18)	0.04545(1/22)	0.10526(2/19)
Bo_TE_79523	0.04444(2/45)	0.83333(15/18)	0.85714(18/21)	0.55000(11/20)
Bo_TE_221321	1.00000(44/44)	0.17647(3/17)	0.70000(14/20)	0.90000(18/20)
Bo_TE_237200	0.06818(3/44)	0.77778(14/18)	0.00000(0/23)	0.00000(0/20)
Bo_TE_151676	0.93478(43/46)	0.82353(14/17)	0.47619(10/21)	0.35000(7/20)
Bo_TE_81688	0.60526(23/38)	0.68421(13/19)	1.00000(23/23)	0.47368(9/19)
Bo_TE_196661	0.78947(30/38)	0.50000(8/16)	0.57143(12/21)	1.00000(19/19)
Bo_TE_241179	1.00000(38/38)	1.00000(17/17)	0.19048(4/21)	1.00000(13/13)
Bo_TE_186087	0.16279(7/43)	1.00000(16/16)	0.80952(17/21)	0.68421(13/19)
Bo_TE_211135	0.00000(0/44)	0.00000(0/19)	0.50000(10/20)	0.61111(11/18)
Bo_TE_101823	0.67500(27/40)	0.36842(7/19)	0.00000(0/23)	0.00000(0/20)
Bo_TE_136936	0.32558(14/43)	0.88235(15/17)	1.00000(22/22)	0.72222(13/18)
Bo_TE_143897	0.00000(0/43)	0.00000(0/17)	0.45455(10/22)	0.65000(13/20)
Bo_TE_98118	0.09302(4/43)	0.00000(0/19)	0.59091(13/22)	0.27778(5/18)
Bo_TE_174592	0.52381(22/42)	0.00000(0/17)	0.00000(0/22)	0.00000(0/20)
Bo_TE_14058	0.95556(43/45)	1.00000(16/16)	0.63636(14/22)	0.27778(5/18)
Bo_TE_42168	0.13953(6/43)	0.18750(3/16)	1.00000(21/21)	1.00000(20/20)
Bo_TE_129785	0.24390(10/41)	0.11111(2/18)	0.95652(22/23)	0.89474(17/19)
Bo_TE_234051	0.00000(0/45)	0.63158(12/19)	0.00000(0/21)	0.00000(0/20)
Bo_TE_40512	0.85366(35/41)	0.00000(0/18)	0.00000(0/22)	0.00000(0/20)
Bo_TE_112868	0.54054(20/37)	0.00000(0/19)	0.63636(14/22)	0.15789(3/19)
Bo_TE_163216	0.97778(44/45)	0.94737(18/19)	0.45000(9/20)	0.31250(5/16)
Bo_TE_119475	0.55814(24/43)	0.43750(7/16)	0.00000(0/23)	0.40000(8/20)
Bo_TE_238600	0.00000(0/46)	0.23529(4/17)	0.52381(11/21)	0.63158(12/19)
Bo_TE_155130	0.08889(4/45)	0.11765(2/17)	0.27273(6/22)	0.68421(13/19)
Bo_TE_122580	0.00000(0/45)	0.00000(0/18)	0.04545(1/22)	0.52941(9/17)
Bo_TE_214150	0.00000(0/46)	0.56250(9/16)	0.22727(5/22)	0.00000(0/20)
Bo_TE_41392	0.00000(0/43)	0.00000(0/17)	0.04348(1/23)	0.75000(15/20)
Bo_TE_107949	0.04762(2/42)	0.05882(1/17)	0.50000(10/20)	0.65000(13/20)
Bo_TE_116799	0.00000(0/46)	0.00000(0/19)	0.59091(13/22)	0.10526(2/19)
Bo_TE_124201	0.19048(8/42)	0.72222(13/18)	0.80952(17/21)	0.85000(17/20)
Bo_TE_18983	0.33333(14/42)	0.61111(11/18)	0.55000(11/20)	0.05263(1/19)
Bo_TE_191380	0.00000(0/46)	0.05556(1/18)	0.27273(6/22)	0.89474(17/19)
Bo_TE_112993	0.57143(24/42)	0.11765(2/17)	0.00000(0/21)	0.00000(0/19)
Bo_TE_100978	0.00000(0/44)	0.50000(9/18)	0.61905(13/21)	0.00000(0/17)
Bo_TE_17912	0.15909(7/44)	0.05882(1/17)	0.04545(1/22)	0.60000(12/20)
Bo_TE_35073	0.09302(4/43)	0.00000(0/13)	0.80952(17/21)	0.61111(11/18)
Bo_TE_199720	0.15556(7/45)	0.31250(5/16)	0.66667(14/21)	0.87500(14/16)
Bo_TE_214308	0.77500(31/40)	0.12500(2/16)	0.28571(6/21)	0.40000(8/20)
Bo_TE_99327	0.91111(41/45)	1.00000(18/18)	0.40000(8/20)	0.72222(13/18)
Bo_TE_169849	0.60000(27/45)	0.11111(2/18)	0.00000(0/22)	0.10000(2/20)
Bo_TE_165336	0.47619(20/42)	1.00000(17/17)	0.81818(18/22)	0.90000(18/20)
Bo_TE_33162	0.00000(0/45)	0.00000(0/17)	0.35000(7/20)	0.89474(17/19)
Bo_TE_38796	0.73171(30/41)	0.75000(12/16)	0.31579(6/19)	0.05263(1/19)

Bo_TE_89223	0.44737(17/38)	0.88235(15/17)	0.10000(2/20)	0.00000(0/16)
Bo_TE_65690	0.15909(7/44)	0.00000(0/18)	0.71429(15/21)	0.00000(0/20)
Bo_TE_45711	0.02222(1/45)	0.58824(10/17)	0.04545(1/22)	0.10000(2/20)
Bo_TE_89465	0.44186(19/43)	0.60000(9/15)	0.90000(18/20)	1.00000(20/20)
Bo_TE_17484	0.55814(24/43)	0.05882(1/17)	0.09524(2/21)	0.00000(0/19)
Bo_TE_53534	0.72500(29/40)	0.23529(4/17)	0.08696(2/23)	0.10000(2/20)
Bo_TE_10232	0.88095(37/42)	0.70588(12/17)	0.00000(0/18)	0.15000(3/20)
Bo_TE_145355	0.60000(24/40)	0.00000(0/17)	0.33333(7/21)	0.31579(6/19)
Bo_TE_87605	0.84444(38/45)	0.25000(4/16)	0.08696(2/23)	0.05263(1/19)
Bo_TE_65679	0.97619(41/42)	0.80000(12/15)	0.42105(8/19)	0.61111(11/18)
Bo_TE_209324	0.40000(16/40)	0.58824(10/17)	0.00000(0/21)	0.00000(0/19)
Bo_TE_159079	0.26667(12/45)	0.11765(2/17)	0.95455(21/22)	1.00000(20/20)
Bo_TE_151784	0.00000(0/45)	0.00000(0/18)	0.19048(4/21)	0.64706(11/17)
Bo_TE_70608	0.00000(0/45)	0.56250(9/16)	0.70000(14/20)	0.20000(4/20)
Bo_TE_53993	0.43902(18/41)	0.83333(15/18)	0.47619(10/21)	0.11111(2/18)
Bo_TE_75874	0.60465(26/43)	0.50000(9/18)	0.04348(1/23)	0.00000(0/18)
Bo_TE_143889	0.50000(21/42)	0.88235(15/17)	0.66667(14/21)	0.35000(7/20)
Bo_TE_104508	0.72093(31/43)	0.75000(12/16)	0.09091(2/22)	0.70000(14/20)
Bo_TE_14671	0.84091(37/44)	0.75000(12/16)	0.19048(4/21)	0.05263(1/19)
Bo_TE_91978	0.80952(34/42)	1.00000(19/19)	0.90476(19/21)	0.40000(8/20)
Bo_TE_31441	0.87805(36/41)	0.50000(9/18)	0.65000(13/20)	1.00000(20/20)
Bo_TE_238478	0.57500(23/40)	0.06250(1/16)	0.00000(0/22)	0.00000(0/18)
Bo_TE_181125	0.68889(31/45)	1.00000(18/18)	0.35000(7/20)	0.68421(13/19)
Bo_TE_163114	0.00000(0/46)	0.00000(0/19)	0.45455(10/22)	0.52632(10/19)
Bo_TE_132705	0.26190(11/42)	0.15789(3/19)	0.00000(0/23)	0.52941(9/17)
Bo_TE_198972	0.15909(7/44)	0.68750(11/16)	1.00000(23/23)	1.00000(20/20)
Bo_TE_63135	0.02381(1/42)	0.76471(13/17)	0.61905(13/21)	0.77778(14/18)
Bo_TE_237014	0.00000(0/44)	0.00000(0/19)	0.72727(16/22)	0.42105(8/19)
Bo_TE_89188	0.48780(20/41)	0.94444(17/18)	0.13636(3/22)	0.26316(5/19)
Bo_TE_121564	0.20455(9/44)	0.83333(15/18)	0.66667(14/21)	0.73684(14/19)
Bo_TE_54033	0.58140(25/43)	0.05556(1/18)	0.00000(0/23)	0.10000(2/20)
Bo_TE_9702	0.61905(26/42)	0.23529(4/17)	1.00000(22/22)	0.78947(15/19)
Bo_TE_182736	0.58140(25/43)	0.05556(1/18)	0.00000(0/22)	0.05000(1/20)
Bo_TE_230745	0.00000(0/44)	0.00000(0/18)	0.05263(1/19)	0.68421(13/19)
Bo_TE_214467	0.83333(35/42)	0.53333(8/15)	0.00000(0/22)	0.05556(1/18)
Bo_TE_9251	0.80000(36/45)	0.37500(6/16)	0.80952(17/21)	1.00000(20/20)
Bo_TE_43802	0.20455(9/44)	0.46667(7/15)	0.72727(16/22)	0.57895(11/19)
Bo_TE_67632	0.21951(9/41)	0.11111(2/18)	0.76190(16/21)	0.15789(3/19)
Bo_TE_37395	0.42105(16/38)	0.18750(3/16)	0.86364(19/22)	0.88889(16/18)
Bo_TE_56011	0.19444(7/36)	0.57895(11/19)	0.00000(0/19)	0.12500(2/16)
Bo_TE_69262	0.02174(1/46)	0.00000(0/17)	0.61905(13/21)	0.44444(8/18)
Bo_TE_12959	0.86364(38/44)	0.06667(1/15)	0.18182(4/22)	0.36842(7/19)
Bo_TE_138276	0.20455(9/44)	0.52632(10/19)	0.19048(4/21)	0.88889(16/18)
Bo_TE_167797	0.24324(9/37)	0.66667(12/18)	0.00000(0/21)	0.15000(3/20)
Bo_TE_129318	0.73171(30/41)	0.76471(13/17)	0.00000(0/23)	0.10526(2/19)
Bo_TE_105924	0.65854(27/41)	0.35294(6/17)	0.00000(0/23)	0.05263(1/19)
Bo_TE_31678	0.54762(23/42)	0.11111(2/18)	0.70000(14/20)	0.84211(16/19)
Bo_TE_45197	0.59091(26/44)	0.68750(11/16)	0.13636(3/22)	0.73684(14/19)
Bo_TE_27472	0.75000(33/44)	0.00000(0/18)	0.00000(0/11)	0.00000(0/16)
Bo_TE_124178	0.74419(32/43)	0.23529(4/17)	0.20000(4/20)	0.15000(3/20)
Bo_TE_6936	0.31707(13/41)	1.00000(17/17)	1.00000(22/22)	0.75000(15/20)
Bo_TE_43584	0.82927(34/41)	0.61111(11/18)	0.00000(0/21)	0.36842(7/19)
Bo_TE_167885	0.83721(36/43)	0.83333(15/18)	0.00000(0/21)	0.52632(10/19)
Bo_TE_86080	0.52273(23/44)	0.31250(5/16)	0.13636(3/22)	0.00000(0/20)
Bo_TE_142420	0.02564(1/39)	0.16667(3/18)	0.34783(8/23)	0.70000(14/20)
Bo_TE_164967	1.00000(44/44)	1.00000(17/17)	0.52632(10/19)	0.25000(5/20)

Bo_TE_91592	0.11628(5/43)	0.55556(10/18)	0.77273(17/22)	0.15000(3/20)
Bo_TE_89165	0.02273(1/44)	0.00000(0/19)	0.85714(18/21)	0.63158(12/19)
Bo_TE_165507	0.34884(15/43)	0.00000(0/17)	0.36364(8/22)	0.78947(15/19)
Bo_TE_3742	0.06522(3/46)	0.64706(11/17)	0.45455(10/22)	0.70000(14/20)
Bo_TE_41438	0.61538(24/39)	0.62500(10/16)	0.19048(4/21)	0.00000(0/20)
Bo_TE_30446	0.00000(0/46)	0.10526(2/19)	0.70000(14/20)	0.15789(3/19)
Bo_TE_154593	0.00000(0/43)	0.00000(0/18)	0.71429(10/14)	0.56250(9/16)
Bo_TE_19317	0.54762(23/42)	0.44444(8/18)	0.04762(1/21)	0.22222(4/18)
Bo_TE_52726	0.00000(0/46)	0.00000(0/19)	0.78947(15/19)	0.20000(4/20)
Bo_TE_80476	0.88372(38/43)	0.43750(7/16)	0.00000(0/22)	0.06250(1/16)
Bo_TE_88418	0.56098(23/41)	0.94444(17/18)	0.26316(5/19)	0.36842(7/19)
Bo_TE_138730	0.06818(3/44)	0.50000(9/18)	0.00000(0/21)	0.05882(1/17)
Bo_TE_78833	0.13953(6/43)	0.52941(9/17)	0.13636(3/22)	0.00000(0/20)
Bo_TE_128797	0.21429(9/42)	0.55556(10/18)	0.00000(0/22)	0.00000(0/20)
Bo_TE_209319	0.37778(17/45)	0.56250(9/16)	0.00000(0/22)	0.00000(0/20)
Bo_TE_115724	0.92857(39/42)	0.89474(17/19)	0.90476(19/21)	0.15000(3/20)
Bo_TE_156113	0.00000(0/45)	0.50000(8/16)	0.20000(4/20)	0.26316(5/19)
Bo_TE_108676	0.93182(41/44)	0.17647(3/17)	0.65000(13/20)	0.75000(15/20)
Bo_TE_97660	0.61364(27/44)	0.17647(3/17)	0.00000(0/22)	0.11111(2/18)
Bo_TE_35870	0.57143(24/42)	0.21053(4/19)	0.00000(0/22)	0.00000(0/18)
Bo_TE_123794	0.11628(5/43)	0.87500(14/16)	0.36842(7/19)	0.33333(6/18)
Bo_TE_162803	0.76190(32/42)	0.29412(5/17)	1.00000(23/23)	1.00000(20/20)
Bo_TE_123699	0.28889(13/45)	1.00000(17/17)	1.00000(23/23)	0.70000(14/20)
Bo_TE_60300	0.53488(23/43)	0.18750(3/16)	0.60000(12/20)	0.90000(18/20)
Bo_TE_172779	0.00000(0/42)	0.27778(5/18)	0.52381(11/21)	0.52632(10/19)
Bo_TE_57004	0.50000(21/42)	0.94737(18/19)	0.90909(20/22)	1.00000(20/20)
Bo_TE_70256	0.71795(28/39)	0.50000(9/18)	0.80952(17/21)	0.15789(3/19)
Bo_TE_26848	0.25581(11/43)	0.88235(15/17)	0.27273(6/22)	0.00000(0/20)
Bo_TE_141905	0.00000(0/46)	0.63158(12/19)	0.00000(0/23)	0.00000(0/20)
Bo_TE_111421	0.26190(11/42)	0.61111(11/18)	0.20000(4/20)	0.73684(14/19)
Bo_TE_30917	0.76190(32/42)	0.70588(12/17)	0.23810(5/21)	0.80000(16/20)
Bo_TE_232163	0.59459(22/37)	0.06250(1/16)	0.00000(0/10)	0.31250(5/16)
Bo_TE_31641	0.65116(28/43)	0.11111(2/18)	0.66667(14/21)	0.85000(17/20)
Bo_TE_20913	0.75000(21/28)	0.35294(6/17)	1.00000(16/16)	0.89474(17/19)
Bo_TE_110940	0.11628(5/43)	0.25000(4/16)	0.90000(18/20)	0.61111(11/18)
Bo_TE_121823	0.00000(0/46)	0.82353(14/17)	0.00000(0/23)	0.00000(0/20)
Bo_TE_144702	0.13636(6/44)	0.88235(15/17)	0.85714(18/21)	0.10000(2/20)
Bo_TE_179257	0.36585(15/41)	0.31250(5/16)	0.71429(15/21)	0.00000(0/20)
Bo_TE_68071	0.64286(27/42)	0.27778(5/18)	0.95652(22/23)	0.75000(15/20)
Bo_TE_68015	0.51220(21/41)	0.11765(2/17)	0.00000(0/23)	0.00000(0/20)
Bo_TE_118518	0.59524(25/42)	0.31579(6/19)	0.04348(1/23)	0.05263(1/19)
Bo_TE_230056	0.82222(37/45)	0.12500(2/16)	0.45000(9/20)	0.15000(3/20)
Bo_TE_44916	0.79487(31/39)	0.22222(4/18)	0.35000(7/20)	0.78947(15/19)
Bo_TE_111251	0.12195(5/41)	0.27778(5/18)	1.00000(19/19)	0.83333(15/18)
Bo_TE_153857	0.09091(4/44)	0.11765(2/17)	0.55000(11/20)	0.80000(16/20)
Bo_TE_66960	0.00000(0/42)	0.61111(11/18)	0.45000(9/20)	0.75000(15/20)
Bo_TE_173227	0.43590(17/39)	0.60000(9/15)	0.66667(14/21)	1.00000(20/20)
Bo_TE_210028	0.92500(37/40)	0.35294(6/17)	1.00000(23/23)	1.00000(18/18)
Bo_TE_225973	0.69767(30/43)	0.33333(6/18)	0.22727(5/22)	0.00000(0/20)
Bo_TE_123385	0.90698(39/43)	0.47059(8/17)	0.20000(4/20)	0.30000(6/20)
Bo_TE_77308	0.22727(10/44)	0.64706(11/17)	1.00000(22/22)	0.90000(18/20)
Bo_TE_236010	0.91111(41/45)	0.37500(6/16)	0.50000(11/22)	0.26316(5/19)
Bo_TE_46412	0.23810(10/42)	0.35294(6/17)	0.33333(6/18)	0.94118(16/17)
Bo_TE_151500	0.24444(11/45)	0.50000(8/16)	0.00000(0/22)	0.33333(6/18)
Bo_TE_141096	1.00000(41/41)	0.71429(10/14)	0.26316(5/19)	1.00000(20/20)
Bo_TE_232079	0.55814(24/43)	0.47059(8/17)	0.04348(1/23)	0.26316(5/19)

Bo_TE_126172	0.67442(29/43)	0.33333(6/18)	0.16667(3/18)	0.90000(18/20)
Bo_TE_154627	0.27907(12/43)	0.33333(5/15)	0.84211(16/19)	0.25000(4/16)
Bo_TE_231135	0.71053(27/38)	0.23529(4/17)	0.90476(19/21)	1.00000(20/20)
Bo_TE_87813	0.08889(4/45)	0.05556(1/18)	0.76190(16/21)	0.16667(3/18)
Bo_TE_100262	0.00000(0/45)	0.00000(0/19)	0.65000(13/20)	0.88889(16/18)
Bo_TE_43474	0.88372(38/43)	0.55556(10/18)	0.05263(1/19)	0.50000(9/18)
Bo_TE_70650	0.00000(0/44)	0.72222(13/18)	0.50000(11/22)	0.25000(5/20)
Bo_TE_190289	0.97619(41/42)	0.83333(15/18)	0.50000(10/20)	1.00000(20/20)
Bo_TE_168635	0.61905(26/42)	1.00000(18/18)	0.31579(6/19)	0.21053(4/19)
Bo_TE_101596	0.69767(30/43)	0.52941(9/17)	0.04545(1/22)	0.61111(11/18)
Bo_TE_148863	0.19048(8/42)	0.42105(8/19)	1.00000(22/22)	1.00000(20/20)
Bo_TE_105659	0.05263(2/38)	0.58824(10/17)	0.06250(1/16)	0.16667(1/6)
Bo_TE_213982	0.35000(14/40)	0.26667(4/15)	0.55000(11/20)	0.88889(16/18)
Bo_TE_34466	0.15385(6/39)	0.41176(7/17)	0.09524(2/21)	0.80000(16/20)
Bo_TE_193602	0.63636(28/44)	0.41176(7/17)	0.78947(15/19)	0.94737(18/19)
Bo_TE_182116	0.79487(31/39)	0.52941(9/17)	0.60000(12/20)	0.05556(1/18)
Bo_TE_191219	0.64286(27/42)	0.82353(14/17)	0.89474(17/19)	0.00000(0/20)
Bo_TE_85940	0.97778(44/45)	0.47368(9/19)	0.50000(11/22)	0.85000(17/20)
Bo_TE_172524	0.56818(25/44)	0.00000(0/18)	0.04348(1/23)	0.00000(0/20)
Bo_TE_237906	0.52632(20/38)	0.05882(1/17)	0.00000(0/23)	0.00000(0/20)
Bo_TE_145998	0.02222(1/45)	0.66667(12/18)	0.30435(7/23)	0.05263(1/19)
Bo_TE_69705	0.45000(18/40)	0.00000(0/18)	0.23810(5/21)	0.50000(10/20)
Bo_TE_174802	0.30233(13/43)	0.58824(10/17)	0.54545(12/22)	0.90000(18/20)
Bo_TE_129907	0.69767(30/43)	0.41176(7/17)	0.08696(2/23)	0.00000(0/19)
Bo_TE_16996	0.67442(29/43)	0.00000(0/16)	0.00000(0/22)	0.00000(0/20)
Bo_TE_18000	0.04545(2/44)	0.17647(3/17)	0.36842(7/19)	0.77778(14/18)
Bo_TE_32928	0.54762(23/42)	0.47368(9/19)	0.00000(0/23)	0.05000(1/20)
Bo_TE_208807	0.00000(0/42)	0.00000(0/17)	0.22727(5/22)	0.89474(17/19)
Bo_TE_18412	0.45000(18/40)	0.05882(1/17)	0.16667(3/18)	0.85000(17/20)
Bo_TE_58013	0.02381(1/42)	0.11111(2/18)	0.00000(0/22)	0.57895(11/19)
Bo_TE_183463	0.42500(17/40)	0.23529(4/17)	0.61111(11/18)	0.89474(17/19)
Bo_TE_189957	0.36364(16/44)	0.11765(2/17)	0.40000(8/20)	1.00000(20/20)
Bo_TE_95333	0.02326(1/43)	0.64706(11/17)	0.47368(9/19)	0.20000(4/20)
Bo_TE_55502	0.51111(23/45)	0.00000(0/18)	0.09524(2/21)	0.05263(1/19)
Bo_TE_135937	0.15385(6/39)	0.62500(10/16)	1.00000(20/20)	0.89474(17/19)
Bo_TE_136892	0.00000(0/45)	0.62500(10/16)	0.00000(0/23)	0.00000(0/19)
Bo_TE_132786	0.65909(29/44)	0.23529(4/17)	0.14286(3/21)	0.22222(4/18)
Bo_TE_159956	0.80000(36/45)	0.44444(8/18)	0.95238(20/21)	0.52632(10/19)
Bo_TE_221025	0.97674(42/43)	1.00000(18/18)	0.72727(16/22)	0.47368(9/19)
Bo_TE_36549	0.69767(30/43)	0.00000(0/17)	0.36364(8/22)	0.25000(5/20)
Bo_TE_164991	0.00000(0/45)	0.00000(0/17)	0.33333(7/21)	0.68421(13/19)
Bo_TE_132418	0.63415(26/41)	0.17647(3/17)	0.82609(19/23)	0.20000(4/20)
Bo_TE_206522	0.04545(2/44)	0.00000(0/18)	0.04545(1/22)	0.65000(13/20)
Bo_TE_122756	0.38462(15/39)	0.35294(6/17)	0.15789(3/19)	0.70588(12/17)
Bo_TE_9222	0.20000(9/45)	0.64706(11/17)	0.19048(4/21)	0.00000(0/20)
Bo_TE_49852	0.84615(33/39)	0.47059(8/17)	0.14286(3/21)	0.25000(3/12)
Bo_TE_193513	0.65000(26/40)	0.41176(7/17)	0.78947(15/19)	0.94737(18/19)
Bo_TE_128556	0.27273(12/44)	0.72222(13/18)	0.55000(11/20)	0.00000(0/19)
Bo_TE_53378	0.55000(22/40)	0.52941(9/17)	0.33333(7/21)	0.90000(18/20)
Bo_TE_134092	0.13953(6/43)	0.66667(12/18)	0.90000(18/20)	0.47368(9/19)
Bo_TE_71384	0.00000(0/46)	0.00000(0/18)	0.23810(5/21)	0.57895(11/19)
Bo_TE_149184	0.93023(40/43)	0.88889(16/18)	0.19048(4/21)	0.55000(11/20)
Bo_TE_148558	0.00000(0/45)	0.16667(3/18)	0.00000(0/23)	0.60000(12/20)
Bo_TE_73310	0.76316(29/38)	0.17647(3/17)	0.00000(0/23)	0.05882(1/17)
Bo_TE_75645	0.00000(0/44)	0.63158(12/19)	0.00000(0/22)	0.06250(1/16)
Bo_TE_18949	0.11628(5/43)	0.25000(4/16)	0.35000(7/20)	0.89474(17/19)

Bo_TE_66840	0.84615(33/39)	0.53333(8/15)	0.00000(0/22)	0.00000(0/19)
Bo_TE_80700	0.36364(16/44)	0.16667(3/18)	0.90476(19/21)	0.95000(19/20)
Bo_TE_68610	0.52381(22/42)	0.22222(4/18)	0.00000(0/22)	0.00000(0/19)
Bo_TE_81448	0.34884(15/43)	0.10526(2/19)	0.09091(2/22)	0.65000(13/20)
Bo_TE_38888	0.02174(1/46)	0.11111(2/18)	0.18182(4/22)	0.57895(11/19)
Bo_TE_46684	0.09091(4/44)	0.33333(6/18)	0.60000(12/20)	0.75000(15/20)
Bo_TE_112861	0.19048(8/42)	0.11111(2/18)	0.52381(11/21)	0.00000(0/20)
Bo_TE_38765	0.93182(41/44)	0.44444(8/18)	0.91304(21/23)	0.95000(19/20)
Bo_TE_171801	0.04762(2/42)	0.66667(12/18)	0.13636(3/22)	0.89474(17/19)
Bo_TE_2814	0.80000(32/40)	0.60000(9/15)	0.00000(0/23)	0.00000(0/20)
Bo_TE_140239	0.97619(41/42)	0.70588(12/17)	0.65217(15/23)	0.30000(6/20)
Bo_TE_122703	0.41026(16/39)	0.12500(2/16)	0.70000(14/20)	0.63158(12/19)
Bo_TE_117821	0.00000(0/44)	0.50000(9/18)	0.42857(9/21)	0.15000(3/20)
Bo_TE_138539	0.53488(23/43)	0.18750(3/16)	0.90909(20/22)	0.65000(13/20)
Bo_TE_58628	0.75000(33/44)	0.00000(0/16)	0.55000(11/20)	0.05000(1/20)
Bo_TE_197634	0.76190(32/42)	0.00000(0/18)	0.85714(18/21)	0.00000(0/20)
Bo_TE_178898	0.00000(0/46)	0.00000(0/18)	0.04762(1/21)	0.57895(11/19)
Bo_TE_184186	0.86364(38/44)	0.35294(6/17)	0.37500(6/16)	1.00000(20/20)
Bo_TE_223806	0.00000(0/45)	0.11111(2/18)	0.40909(9/22)	0.55000(11/20)
Bo_TE_125107	0.37500(15/40)	0.17647(3/17)	0.45000(9/20)	0.83333(15/18)
Bo_TE_177428	0.44186(19/43)	0.76471(13/17)	0.13043(3/23)	0.05000(1/20)
Bo_TE_125742	0.00000(0/46)	0.58824(10/17)	0.66667(14/21)	0.11111(2/18)
Bo_TE_204420	0.38636(17/44)	0.88235(15/17)	0.56522(13/23)	0.05263(1/19)
Bo_TE_57943	0.06667(3/45)	0.60000(9/15)	0.40000(8/20)	0.63158(12/19)
Bo_TE_15626	0.40476(17/42)	0.70588(12/17)	0.00000(0/22)	0.00000(0/20)
Bo_TE_24826	0.63636(28/44)	0.43750(7/16)	0.95000(19/20)	0.87500(14/16)
Bo_TE_85423	0.69565(32/46)	0.50000(7/14)	0.42857(9/21)	0.11765(2/17)
Bo_TE_162066	0.00000(0/44)	0.00000(0/19)	0.50000(10/20)	0.05000(1/20)
Bo_TE_62909	0.04762(2/42)	0.66667(12/18)	0.45000(9/20)	0.00000(0/20)
Bo_TE_37055	0.47368(18/38)	0.71429(5/7)	1.00000(23/23)	0.61111(11/18)
Bo_TE_114150	1.00000(43/43)	0.55556(10/18)	0.95238(20/21)	0.47368(9/19)
Bo_TE_209696	0.82222(37/45)	0.11111(2/18)	0.59091(13/22)	0.89474(17/19)
Bo_TE_226243	0.41463(17/41)	0.77778(14/18)	0.00000(0/23)	0.05000(1/20)
Bo_TE_93639	0.95652(44/46)	0.11765(2/17)	1.00000(22/22)	1.00000(20/20)
Bo_TE_178807	0.67442(29/43)	0.25000(4/16)	0.50000(10/20)	0.05000(1/20)
Bo_TE_73036	0.95000(38/40)	0.20000(3/15)	0.57143(12/21)	0.42105(8/19)
Bo_TE_213641	0.36842(14/38)	0.42857(6/14)	0.90476(19/21)	0.94737(18/19)
Bo_TE_76582	0.95349(41/43)	0.50000(9/18)	0.50000(10/20)	1.00000(20/20)
Bo_TE_230479	0.48837(21/43)	0.00000(0/15)	0.08696(2/23)	0.83333(15/18)
Bo_TE_221925	0.36585(15/41)	0.76471(13/17)	0.18182(4/22)	0.00000(0/20)
Bo_TE_239609	0.27500(11/40)	0.52941(9/17)	1.00000(19/19)	0.57895(11/19)
Bo_TE_101666	0.23810(10/42)	0.75000(12/16)	0.92308(12/13)	0.57143(4/7)
Bo_TE_44194	1.00000(41/41)	0.35294(6/17)	0.66667(14/21)	0.17647(3/17)
Bo_TE_130168	0.16667(7/42)	0.52941(9/17)	0.86364(19/22)	0.89474(17/19)
Bo_TE_107641	0.50000(23/46)	0.83333(15/18)	0.00000(0/22)	0.00000(0/20)
Bo_TE_141460	0.15789(6/38)	0.94118(16/17)	0.95238(20/21)	0.78947(15/19)
Bo_TE_105484	0.47727(21/44)	0.93750(15/16)	0.38095(8/21)	0.42105(8/19)
Bo_TE_70619	0.00000(0/46)	0.00000(0/19)	0.68182(15/22)	0.00000(0/20)
Bo_TE_18238	0.86047(37/43)	0.81250(13/16)	0.00000(0/19)	0.80000(16/20)
Bo_TE_133706	0.95455(42/44)	1.00000(14/14)	0.21053(4/19)	0.21053(4/19)
Bo_TE_57772	0.64286(27/42)	0.05556(1/18)	0.00000(0/22)	0.00000(0/20)
Bo_TE_78746	0.60465(26/43)	0.27778(5/18)	0.04348(1/23)	0.00000(0/20)
Bo_TE_221270	0.00000(0/44)	0.81250(13/16)	0.04762(1/21)	0.05263(1/19)
Bo_TE_195014	0.61905(26/42)	0.29412(5/17)	0.70000(14/20)	0.00000(0/19)
Bo_TE_201652	0.91111(41/45)	0.33333(6/18)	0.18182(4/22)	0.57895(11/19)
Bo_TE_69529	0.06667(3/45)	0.00000(0/18)	0.00000(0/23)	0.72222(13/18)

Bo_TE_44044	0.15385(6/39)	0.75000(12/16)	0.10526(2/19)	0.10000(2/20)
Bo_TE_30051	0.78947(30/38)	0.23529(4/17)	0.31579(6/19)	0.05263(1/19)
Bo_TE_104802	0.97674(42/43)	1.00000(17/17)	0.66667(14/21)	0.47368(9/19)
Bo_TE_195664	1.00000(46/46)	0.35294(6/17)	0.19048(4/21)	0.78947(15/19)
Bo_TE_222123	0.37209(16/43)	0.12500(2/16)	0.90000(18/20)	0.77778(14/18)
Bo_TE_117512	0.95455(42/44)	0.11765(2/17)	0.50000(9/18)	0.58824(10/17)
Bo_TE_55718	0.93478(43/46)	1.00000(19/19)	0.91304(21/23)	0.11111(2/18)
Bo_TE_70384	0.60465(26/43)	0.06250(1/16)	0.18182(4/22)	0.31579(6/19)
Bo_TE_104956	0.65909(29/44)	0.00000(0/16)	0.36364(8/22)	0.57895(11/19)
Bo_TE_129698	0.14706(5/34)	0.88889(16/18)	0.04545(1/22)	0.58824(10/17)
Bo_TE_112377	0.97297(36/37)	0.25000(4/16)	1.00000(20/20)	1.00000(10/10)
Bo_TE_179758	0.00000(0/46)	0.11111(2/18)	0.61905(13/21)	0.00000(0/20)
Bo_TE_227712	0.92683(38/41)	1.00000(17/17)	0.20000(4/20)	0.17647(3/17)
Bo_TE_118352	0.30769(12/39)	0.21053(4/19)	0.95455(21/22)	0.94737(18/19)
Bo_TE_118473	0.00000(0/45)	0.00000(0/18)	0.55000(11/20)	0.85000(17/20)
Bo_TE_62808	0.02174(1/46)	0.66667(12/18)	0.47619(10/21)	0.00000(0/19)
Bo_TE_58402	0.97674(42/43)	0.42105(8/19)	0.61905(13/21)	0.26316(5/19)
Bo_TE_68406	0.45000(18/40)	0.05882(1/17)	0.93750(15/16)	0.64706(11/17)
Bo_TE_143987	0.00000(0/45)	0.10526(2/19)	0.78261(18/23)	0.89474(17/19)
Bo_TE_99507	0.23256(10/43)	0.53333(8/15)	0.95455(21/22)	1.00000(20/20)
Bo_TE_136679	0.34146(14/41)	0.58824(10/17)	1.00000(21/21)	0.94737(18/19)
Bo_TE_190996	0.58974(23/39)	0.11765(2/17)	0.00000(0/22)	0.21053(4/19)
Bo_TE_17969	0.80488(33/41)	0.41176(7/17)	0.28571(6/21)	0.90000(18/20)
Bo_TE_80301	0.88372(38/43)	0.73684(14/19)	0.52381(11/21)	0.15789(3/19)
Bo_TE_236223	0.10870(5/46)	0.76471(13/17)	1.00000(22/22)	1.00000(20/20)
Bo_TE_214406	0.76190(32/42)	0.54545(6/11)	0.13043(3/23)	0.50000(9/18)
Bo_TE_120795	0.42857(18/42)	0.70588(12/17)	0.91304(21/23)	1.00000(19/19)
Bo_TE_126744	0.02273(1/44)	0.11111(2/18)	0.30435(7/23)	0.70588(12/17)
Bo_TE_37115	0.36842(14/38)	0.33333(6/18)	1.00000(22/22)	0.88889(16/18)
Bo_TE_2854	0.55000(22/40)	0.94118(16/17)	0.37500(3/8)	0.25000(4/16)
Bo_TE_150792	0.88372(38/43)	0.22222(4/18)	0.18182(4/22)	0.66667(12/18)
Bo_TE_111970	0.54348(25/46)	0.87500(14/16)	0.23810(5/21)	0.89474(17/19)
Bo_TE_167767	0.06667(3/45)	0.16667(3/18)	0.60000(12/20)	0.00000(0/19)
Bo_TE_69613	0.04545(2/44)	0.72222(13/18)	0.00000(0/22)	0.00000(0/20)
Bo_TE_80805	0.43182(19/44)	0.41176(7/17)	0.95455(21/22)	0.26316(5/19)
Bo_TE_92891	0.53659(22/41)	0.82353(14/17)	0.00000(0/16)	0.17647(3/17)
Bo_TE_28499	0.17949(7/39)	0.62500(10/16)	1.00000(22/22)	0.89474(17/19)
Bo_TE_172032	0.90909(40/44)	0.73684(14/19)	0.19048(4/21)	0.94444(17/18)
Bo_TE_104661	0.32558(14/43)	0.72222(13/18)	1.00000(22/22)	0.57895(11/19)
Bo_TE_125098	0.64103(25/39)	0.88235(15/17)	0.57143(12/21)	0.15000(3/20)
Bo_TE_17753	0.08889(4/45)	0.00000(0/18)	0.80000(16/20)	0.36842(7/19)
Bo_TE_173219	0.58974(23/39)	0.29412(5/17)	0.27273(6/22)	0.00000(0/20)
Bo_TE_147844	0.55000(22/40)	0.47059(8/17)	0.00000(0/21)	0.00000(0/19)
Bo_TE_207041	0.11364(5/44)	0.00000(0/18)	0.00000(0/19)	0.55556(10/18)
Bo_TE_104839	0.10256(4/39)	0.43750(7/16)	0.61905(13/21)	0.30000(6/20)
Bo_TE_173212	0.56410(22/39)	0.25000(4/16)	0.27273(6/22)	0.00000(0/19)
Bo_TE_140921	0.73810(31/42)	0.05882(1/17)	0.09524(2/21)	0.16667(3/18)
Bo_TE_25705	0.23810(10/42)	0.66667(10/15)	0.00000(0/23)	0.60000(12/20)
Bo_TE_230907	0.86667(39/45)	0.88889(16/18)	0.00000(0/23)	0.00000(0/19)
Bo_TE_50229	0.19048(8/42)	0.55556(10/18)	0.65000(13/20)	0.80000(16/20)
Bo_TE_62810	0.02273(1/44)	0.66667(12/18)	0.47619(10/21)	0.00000(0/20)
Bo_TE_144900	0.72093(31/43)	0.12500(2/16)	0.00000(0/22)	0.00000(0/18)
Bo_TE_175090	0.25581(11/43)	0.60000(9/15)	0.13043(3/23)	0.89474(17/19)
Bo_TE_79474	0.75000(30/40)	0.55556(10/18)	0.60000(12/20)	0.25000(5/20)
Bo_TE_84187	0.02326(1/43)	0.80000(12/15)	0.60000(12/20)	0.55556(10/18)
Bo_TE_126814	0.35897(14/39)	0.94118(16/17)	1.00000(23/23)	0.68421(13/19)

Bo_TE_170557	0.04444(2/45)	0.00000(0/17)	0.52381(11/21)	0.00000(0/20)
Bo_TE_41138	0.95349(41/43)	1.00000(18/18)	0.95455(21/22)	0.21053(4/19)
Bo_TE_83961	0.86364(38/44)	1.00000(18/18)	0.38095(8/21)	0.20000(4/20)
Bo_TE_33388	0.84091(37/44)	0.06667(1/15)	0.14286(3/21)	0.05000(1/20)
Bo_TE_139288	0.00000(0/45)	0.05263(1/19)	0.00000(0/23)	0.52632(10/19)
Bo_TE_205083	0.16667(7/42)	0.73333(11/15)	0.55000(11/20)	0.90000(18/20)
Bo_TE_45273	0.65000(26/40)	0.62500(10/16)	0.14286(3/21)	1.00000(20/20)
Bo_TE_216910	0.41860(18/43)	0.52941(9/17)	1.00000(23/23)	0.80000(16/20)
Bo_TE_199735	0.40909(18/44)	0.38889(7/18)	0.19048(4/21)	0.82353(14/17)
Bo_TE_156627	0.42222(19/45)	0.05263(1/19)	0.00000(0/23)	0.75000(15/20)
Bo_TE_91117	0.06667(3/45)	0.55556(10/18)	0.25000(5/20)	0.00000(0/20)
Bo_TE_25637	0.51282(20/39)	0.23529(4/17)	0.08696(2/23)	0.73684(14/19)
Bo_TE_89133	0.97826(45/46)	0.64706(11/17)	0.13636(3/22)	0.72222(13/18)
Bo_TE_238396	0.80000(32/40)	1.00000(15/15)	0.09524(2/21)	0.00000(0/20)
Bo_TE_215393	0.79070(34/43)	0.41176(7/17)	0.35000(7/20)	0.90000(18/20)
Bo_TE_152103	0.45238(19/42)	0.88889(16/18)	0.05000(1/20)	0.33333(6/18)
Bo_TE_43198	0.06977(3/43)	0.64706(11/17)	0.00000(0/23)	0.00000(0/20)
Bo_TE_75728	0.97778(44/45)	0.36842(7/19)	0.80000(16/20)	1.00000(20/20)
Bo_TE_216568	0.69048(29/42)	0.87500(14/16)	0.71429(15/21)	0.15000(3/20)
Bo_TE_12538	0.80000(36/45)	0.11111(2/18)	0.47619(10/21)	0.10000(2/20)
Bo_TE_133004	0.15909(7/44)	0.55556(10/18)	0.95652(22/23)	0.40000(8/20)
Bo_TE_239703	0.76744(33/43)	0.41176(7/17)	0.04762(1/21)	0.05882(1/17)
Bo_TE_74385	0.06667(3/45)	0.61111(11/18)	0.00000(0/18)	0.10000(2/20)
Bo_TE_9995	0.63636(28/44)	0.17647(3/17)	0.68421(13/19)	1.00000(17/17)
Bo_TE_148562	0.02174(1/46)	0.17647(3/17)	0.00000(0/23)	0.55556(10/18)
Bo_TE_171877	0.13636(6/44)	0.00000(0/17)	0.57143(12/21)	0.10000(2/20)
Bo_TE_80385	0.87500(35/40)	0.70588(12/17)	0.55000(11/20)	0.15789(3/19)
Bo_TE_191268	0.14286(6/42)	0.10526(2/19)	0.81818(18/22)	0.05000(1/20)
Bo_TE_181722	0.84444(38/45)	0.44444(8/18)	1.00000(20/20)	0.68421(13/19)
Bo_TE_200316	0.79070(34/43)	0.52941(9/17)	0.00000(0/21)	0.40000(8/20)
Bo_TE_158892	0.73810(31/42)	0.00000(0/10)	0.00000(0/21)	0.70000(14/20)
Bo_TE_45608	0.41463(17/41)	0.93750(15/16)	0.73684(14/19)	1.00000(20/20)
Bo_TE_232063	0.13953(6/43)	0.05556(1/18)	0.57143(12/21)	0.20000(4/20)
Bo_TE_128769	0.02222(1/45)	0.11111(2/18)	0.57143(12/21)	0.00000(0/20)
Bo_TE_238881	0.40000(16/40)	0.87500(14/16)	1.00000(23/23)	1.00000(20/20)
Bo_TE_50377	0.97778(44/45)	0.58824(10/17)	0.86364(19/22)	0.20000(4/20)
Bo_TE_154643	0.72727(32/44)	0.66667(10/15)	0.22727(5/22)	0.68421(13/19)
Bo_TE_38940	0.00000(0/43)	0.11111(2/18)	0.17391(4/23)	0.57895(11/19)
Bo_TE_9155	0.08696(4/46)	0.75000(12/16)	0.00000(0/23)	0.00000(0/20)
Bo_TE_88632	0.81395(35/43)	0.81250(13/16)	0.00000(0/23)	0.10000(2/20)
Bo_TE_184862	0.41026(16/39)	1.00000(17/17)	1.00000(21/21)	1.00000(19/19)
Bo_TE_193883	0.00000(0/44)	0.10526(2/19)	0.77273(17/22)	0.10000(2/20)
Bo_TE_215352	0.95652(44/46)	0.36842(7/19)	0.65217(15/23)	0.23529(4/17)
Bo_TE_91124	0.06977(3/43)	0.52941(9/17)	0.28571(6/21)	0.00000(0/20)
Bo_TE_124956	0.41860(18/43)	0.05882(1/17)	0.55000(11/20)	0.77778(14/18)
Bo_TE_114092	0.00000(0/45)	0.52941(9/17)	0.57143(12/21)	0.50000(10/20)
Bo_TE_70163	0.60976(25/41)	0.05556(1/18)	0.72727(16/22)	0.00000(0/19)
Bo_TE_15726	0.40476(17/42)	0.55556(10/18)	0.68182(15/22)	1.00000(19/19)
Bo_TE_37217	0.70455(31/44)	0.38889(7/18)	1.00000(22/22)	0.95000(19/20)
Bo_TE_141393	0.18182(8/44)	0.94118(16/17)	0.95000(19/20)	0.72222(13/18)
Bo_TE_53107	1.00000(45/45)	0.31579(6/19)	1.00000(23/23)	1.00000(19/19)
Bo_TE_137379	0.59459(22/37)	0.77778(7/9)	0.95000(19/20)	0.33333(6/18)
Bo_TE_105942	0.62500(25/40)	0.27778(5/18)	0.00000(0/22)	0.05000(1/20)
Bo_TE_97574	0.13333(6/45)	0.10526(2/19)	0.77273(17/22)	0.23529(4/17)
Bo_TE_170995	0.40476(17/42)	0.11765(2/17)	0.35000(7/20)	0.90000(18/20)
Bo_TE_173606	0.00000(0/45)	0.00000(0/19)	0.70000(14/20)	0.68421(13/19)

Bo_TE_65094	0.02326(1/43)	0.00000(0/19)	0.57143(12/21)	0.00000(0/20)
Bo_TE_63594	0.04444(2/45)	0.64706(11/17)	0.09091(2/22)	0.00000(0/20)
Bo_TE_107910	0.02174(1/46)	0.81250(13/16)	0.50000(10/20)	0.10000(2/20)
Bo_TE_30856	0.75000(30/40)	0.68750(11/16)	0.15789(3/19)	0.72222(13/18)
Bo_TE_69555	0.06667(3/45)	0.11111(2/18)	0.00000(0/23)	0.80000(16/20)
Bo_TE_3849	0.17647(6/34)	0.85714(12/14)	0.36842(7/19)	1.00000(19/19)
Bo_TE_36864	0.97778(44/45)	0.50000(8/16)	0.81818(18/22)	1.00000(20/20)
Bo_TE_96526	1.00000(46/46)	1.00000(19/19)	0.15000(3/20)	0.23529(4/17)
Bo_TE_31637	0.64286(27/42)	0.05882(1/17)	0.65000(13/20)	0.82353(14/17)
Bo_TE_128820	0.26829(11/41)	0.55556(10/18)	0.00000(0/23)	0.00000(0/20)
Bo_TE_159837	0.58537(24/41)	0.05556(1/18)	0.71429(15/21)	0.00000(0/19)
Bo_TE_112889	0.16216(6/37)	0.33333(5/15)	0.42857(9/21)	0.78947(15/19)
Bo_TE_230714	0.02273(1/44)	0.53333(8/15)	0.14286(3/21)	0.70588(12/17)
Bo_TE_239039	0.93023(40/43)	0.94118(16/17)	0.50000(10/20)	1.00000(20/20)
Bo_TE_148841	0.30000(12/40)	0.66667(12/18)	1.00000(22/22)	1.00000(20/20)
Bo_TE_113488	0.83333(35/42)	0.64706(11/17)	0.00000(0/17)	0.05882(1/17)
Bo_TE_40788	1.00000(44/44)	0.52941(9/17)	0.47368(9/19)	0.57895(11/19)
Bo_TE_112352	0.41026(16/39)	0.29412(5/17)	0.15000(3/20)	0.95000(19/20)
Bo_TE_236498	0.46512(20/43)	0.83333(15/18)	0.50000(11/22)	0.10000(2/20)
Bo_TE_151934	0.47500(19/40)	0.11765(2/17)	0.61905(13/21)	0.15789(3/19)
Bo_TE_138720	0.70732(29/41)	0.50000(8/16)	1.00000(22/22)	1.00000(18/18)
Bo_TE_42099	0.53333(24/45)	0.56250(9/16)	0.09524(2/21)	0.05556(1/18)
Bo_TE_134195	0.11111(5/45)	0.61111(11/18)	0.08696(2/23)	0.00000(0/20)
Bo_TE_82104	0.97674(42/43)	0.93750(15/16)	1.00000(20/20)	0.45000(9/20)
Bo_TE_147968	0.86667(39/45)	0.80000(12/15)	0.22727(5/22)	0.00000(0/20)
Bo_TE_4434	0.06818(3/44)	0.68750(11/16)	0.43478(10/23)	0.25000(5/20)
Bo_TE_87494	0.81395(35/43)	0.21053(4/19)	0.47368(9/19)	0.75000(15/20)
Bo_TE_138949	0.18182(8/44)	0.52941(9/17)	0.65000(13/20)	0.75000(15/20)
Bo_TE_107967	0.02222(1/45)	0.70588(12/17)	0.31579(6/19)	0.52632(10/19)
Bo_TE_222256	0.00000(0/46)	0.76471(13/17)	0.00000(0/23)	0.05000(1/20)
Bo_TE_155086	0.84091(37/44)	0.84211(16/19)	0.72727(16/22)	0.31579(6/19)
Bo_TE_182748	0.60465(26/43)	0.00000(0/17)	0.00000(0/21)	0.10000(2/20)
Bo_TE_236941	0.50000(22/44)	0.05882(1/17)	0.68182(15/22)	0.35000(7/20)
Bo_TE_70818	0.31818(14/44)	0.82353(14/17)	0.81818(18/22)	0.36842(7/19)
Bo_TE_103302	0.00000(0/42)	0.00000(0/18)	0.31818(7/22)	0.66667(12/18)
Bo_TE_8582	0.84615(33/39)	0.11765(2/17)	0.13636(3/22)	0.05000(1/20)
Bo_TE_37268	0.83333(35/42)	0.41176(7/17)	1.00000(21/21)	0.94444(17/18)
Bo_TE_234895	0.97778(44/45)	0.94444(17/18)	0.95455(21/22)	0.47368(9/19)
Bo_TE_60281	0.57500(23/40)	0.83333(15/18)	0.27273(6/22)	0.80000(16/20)
Bo_TE_143999	0.00000(0/43)	0.00000(0/19)	0.71429(15/21)	0.80000(16/20)
Bo_TE_236507	0.02273(1/44)	0.00000(0/19)	0.15000(3/20)	0.61111(11/18)
Bo_TE_23414	0.25581(11/43)	0.66667(10/15)	0.17391(4/23)	0.68421(13/19)
Bo_TE_20835	0.85185(23/27)	0.37500(6/16)	0.95238(20/21)	0.50000(8/16)
Bo_TE_163724	0.63636(28/44)	0.18750(3/16)	1.00000(22/22)	1.00000(20/20)
Bo_TE_47420	0.84211(32/38)	0.66667(12/18)	0.13333(2/15)	0.27778(5/18)
Bo_TE_219993	0.00000(0/40)	0.00000(0/11)	0.57143(12/21)	0.10526(2/19)
Bo_TE_223004	0.69767(30/43)	0.88235(15/17)	0.85714(18/21)	0.00000(0/20)
Bo_TE_179727	0.00000(0/45)	0.11111(2/18)	0.61905(13/21)	0.00000(0/20)
Bo_TE_70372	0.56818(25/44)	0.88235(15/17)	0.80952(17/21)	0.27778(5/18)
Bo_TE_86514	0.00000(0/43)	0.31250(5/16)	0.47619(10/21)	0.75000(15/20)
Bo_TE_39734	0.39024(16/41)	0.61111(11/18)	0.09524(2/21)	0.10526(2/19)
Bo_TE_159620	0.02381(1/42)	0.25000(4/16)	0.09091(2/22)	0.83333(15/18)
Bo_TE_163324	0.97778(44/45)	0.50000(9/18)	1.00000(23/23)	1.00000(20/20)
Bo_TE_163208	0.04545(2/44)	0.05556(1/18)	0.60000(12/20)	0.75000(15/20)
Bo_TE_64074	0.02273(1/44)	0.11765(2/17)	0.55000(11/20)	0.00000(0/20)
Bo_TE_178214	0.11905(5/42)	0.38889(7/18)	0.65000(13/20)	0.00000(0/20)

Bo_TE_12660	0.86047(37/43)	0.06250(1/16)	0.08696(2/23)	0.06250(1/16)
Bo_TE_126792	0.13636(6/44)	0.81250(13/16)	0.95455(21/22)	0.72222(13/18)
Bo_TE_100799	0.81818(36/44)	1.00000(16/16)	0.90476(19/21)	0.15000(3/20)
Bo_TE_49998	0.19048(8/42)	0.05556(1/18)	0.00000(0/21)	0.63158(12/19)
Bo_TE_89996	0.09302(4/43)	0.00000(0/18)	0.23810(5/21)	0.68421(13/19)
Bo_TE_143559	0.69048(29/42)	0.66667(12/18)	1.00000(21/21)	0.38889(7/18)
Bo_TE_30058	0.06522(3/46)	0.58824(10/17)	0.55556(5/9)	0.66667(10/15)
Bo_TE_177643	0.00000(0/45)	0.75000(12/16)	0.00000(0/23)	0.00000(0/19)
Bo_TE_145919	0.64286(27/42)	0.05263(1/19)	0.00000(0/23)	0.00000(0/20)
Bo_TE_186015	0.72727(32/44)	0.35294(6/17)	0.09091(2/22)	0.00000(0/20)
Bo_TE_93248	0.39535(17/43)	0.81250(13/16)	0.04348(1/23)	0.10000(2/20)
Bo_TE_129038	0.00000(0/44)	0.50000(9/18)	0.00000(0/23)	0.00000(0/20)
Bo_TE_197486	0.97727(43/44)	0.89474(17/19)	0.82609(19/23)	0.35294(6/17)
Bo_TE_54333	0.95349(41/43)	0.43750(7/16)	0.73684(14/19)	0.50000(10/20)
Bo_TE_213979	0.37209(16/43)	0.25000(4/16)	0.57143(12/21)	0.89474(17/19)
Bo_TE_116191	0.80952(34/42)	0.55556(10/18)	0.19048(4/21)	0.40000(8/20)
Bo_TE_34770	0.75000(33/44)	0.77778(14/18)	0.31818(7/22)	0.00000(0/19)
Bo_TE_41164	0.04545(2/44)	0.00000(0/19)	0.04348(1/23)	0.73684(14/19)
Bo_TE_67716	0.81395(35/43)	1.00000(15/15)	0.25000(5/20)	0.73684(14/19)
Bo_TE_162023	0.42857(18/42)	0.76471(13/17)	1.00000(23/23)	0.95000(19/20)
Bo_TE_131561	0.47727(21/44)	0.11111(2/18)	0.66667(14/21)	0.88889(16/18)
Bo_TE_54054	0.02564(1/39)	0.52941(9/17)	0.45000(9/20)	0.22222(4/18)
Bo_TE_41234	0.92857(39/42)	0.31579(6/19)	0.61905(13/21)	0.30000(6/20)
Bo_TE_23109	0.00000(0/45)	0.76471(13/17)	0.15000(3/20)	0.00000(0/20)
Bo_TE_194897	0.19048(8/42)	0.23529(4/17)	0.15000(3/20)	0.76471(13/17)
Bo_TE_97538	0.61364(27/44)	0.76471(13/17)	0.00000(0/20)	0.35294(6/17)
Bo_TE_89957	0.09756(4/41)	0.44444(8/18)	0.80000(16/20)	0.73684(14/19)
Bo_TE_92883	0.64444(29/45)	0.77778(14/18)	0.73684(14/19)	0.11111(2/18)
Bo_TE_97567	0.76190(32/42)	0.70588(12/17)	0.21739(5/23)	0.26316(5/19)
Bo_TE_89382	0.00000(0/45)	0.00000(0/19)	0.76190(16/21)	0.00000(0/19)
Bo_TE_143905	1.00000(44/44)	0.94444(17/18)	0.40000(8/20)	0.35000(7/20)
Bo_TE_89226	0.53488(23/43)	0.12500(2/16)	0.86364(19/22)	1.00000(18/18)
Bo_TE_237238	0.61364(27/44)	0.11111(2/18)	0.86667(13/15)	0.38889(7/18)
Bo_TE_50636	0.16279(7/43)	0.83333(15/18)	0.66667(14/21)	0.10000(2/20)
Bo_TE_92440	0.04545(2/44)	0.10526(2/19)	0.75000(15/20)	0.20000(4/20)
Bo_TE_183877	0.11628(5/43)	0.64706(11/17)	0.26316(5/19)	0.31579(6/19)
Bo_TE_241128	0.00000(0/45)	0.00000(0/19)	0.55000(11/20)	0.00000(0/20)
Bo_TE_181338	0.97826(45/46)	0.46667(7/15)	0.13636(3/22)	0.70000(14/20)
Bo_TE_158210	0.88636(39/44)	0.68750(11/16)	0.15000(3/20)	0.33333(6/18)
Bo_TE_111788	0.25581(11/43)	0.00000(0/16)	0.75000(15/20)	0.10000(2/20)
Bo_TE_208	0.18605(8/43)	0.89474(17/19)	0.54545(12/22)	0.90000(18/20)
Bo_TE_70438	0.68889(31/45)	0.00000(0/19)	0.00000(0/23)	0.00000(0/20)
Bo_TE_212130	0.58974(23/39)	0.50000(8/16)	0.04545(1/22)	0.38889(7/18)
Bo_TE_144724	0.13636(6/44)	0.83333(15/18)	0.81818(18/22)	0.05000(1/20)
Bo_TE_231152	0.88095(37/42)	0.18750(3/16)	0.90476(19/21)	0.93333(14/15)
Bo_TE_95189	0.13158(5/38)	0.47059(8/17)	0.70000(14/20)	0.05000(1/20)
Bo_TE_193541	0.66667(28/42)	0.37500(6/16)	0.85000(17/20)	0.95000(19/20)
Bo_TE_148547	0.97727(43/44)	0.16667(3/18)	1.00000(23/23)	0.15789(3/19)
Bo_TE_136487	0.08889(4/45)	0.05556(1/18)	0.60000(12/20)	0.60000(12/20)
Bo_TE_90646	0.10000(4/40)	0.85714(12/14)	0.61905(13/21)	0.15000(3/20)
Bo_TE_54430	0.02174(1/46)	0.00000(0/18)	0.66667(14/21)	0.25000(5/20)
Bo_TE_59505	0.28571(12/42)	0.00000(0/15)	0.68182(15/22)	0.94737(18/19)
Bo_TE_156920	0.51111(23/45)	0.16667(3/18)	0.00000(0/23)	0.05000(1/20)
Bo_TE_45796	0.42857(18/42)	0.17647(3/17)	1.00000(21/21)	0.80000(16/20)
Bo_TE_96868	0.97674(42/43)	0.93750(15/16)	0.95455(21/22)	0.47059(8/17)
Bo_TE_54931	0.91892(34/37)	0.11765(2/17)	0.23810(5/21)	0.55000(11/20)

Bo_TE_25308	0.76744(33/43)	0.31250(5/16)	0.28571(6/21)	0.10526(2/19)
Bo_TE_161194	0.13953(6/43)	0.23529(4/17)	0.04762(1/21)	0.63158(12/19)
Bo_TE_91864	0.41860(18/43)	0.93750(15/16)	1.00000(1/1)	0.00000(0/2)
Bo_TE_11629	0.41026(16/39)	0.66667(12/18)	0.95652(22/23)	0.30000(6/20)
Bo_TE_93625	0.04444(2/45)	0.88235(15/17)	0.00000(0/23)	0.00000(0/19)
Bo_TE_63111	0.02326(1/43)	0.64706(11/17)	0.00000(0/23)	0.20000(4/20)
Bo_TE_106928	0.02273(1/44)	0.41176(7/17)	0.00000(0/22)	0.50000(10/20)
Bo_TE_121330	0.69048(29/42)	0.84211(16/19)	0.35000(7/20)	0.15789(3/19)
Bo_TE_235133	0.67442(29/43)	0.11765(2/17)	0.90909(20/22)	0.50000(9/18)
Bo_TE_220610	0.71053(27/38)	0.44444(8/18)	0.50000(10/20)	0.00000(0/20)
Bo_TE_214305	0.20930(9/43)	0.11765(2/17)	0.63636(14/22)	0.52632(10/19)
Bo_TE_222186	0.80952(34/42)	0.05882(1/17)	0.00000(0/22)	0.05263(1/19)
Bo_TE_168188	0.46341(19/41)	0.05556(1/18)	0.10000(2/20)	0.78947(15/19)
Bo_TE_184386	0.08889(4/45)	0.17647(3/17)	0.78947(15/19)	0.50000(10/20)
Bo_TE_222246	0.00000(0/44)	0.70588(12/17)	0.00000(0/23)	0.05000(1/20)
Bo_TE_183135	0.60000(24/40)	0.55556(10/18)	0.38095(8/21)	0.00000(0/20)
Bo_TE_95263	0.17143(6/35)	0.58824(10/17)	0.25000(5/20)	0.05882(1/17)
Bo_TE_104655	0.53488(23/43)	0.47059(8/17)	0.80000(16/20)	0.30000(6/20)
Bo_TE_147891	0.19048(8/42)	0.44444(8/18)	0.40000(8/20)	0.89474(17/19)
Bo_TE_182228	0.22222(10/45)	0.72222(13/18)	0.00000(0/21)	0.26316(5/19)
Bo_TE_67667	0.76744(33/43)	0.93750(15/16)	0.23810(5/21)	0.65000(13/20)
Bo_TE_116016	0.00000(0/43)	0.33333(4/12)	0.52381(11/21)	0.23077(3/13)
Bo_TE_153407	0.00000(0/44)	0.00000(0/18)	0.75000(15/20)	0.89474(17/19)
Bo_TE_158900	0.19512(8/41)	0.87500(14/16)	0.57143(12/21)	0.05000(1/20)
Bo_TE_178324	0.45455(20/44)	0.33333(6/18)	0.15000(3/20)	0.94737(18/19)
Bo_TE_108473	0.02222(1/45)	0.58824(10/17)	0.18182(4/22)	0.05000(1/20)
Bo_TE_71276	0.11111(5/45)	0.87500(14/16)	0.75000(15/20)	0.55000(11/20)
Bo_TE_204670	0.95455(42/44)	0.94118(16/17)	0.45455(10/22)	0.80000(16/20)
Bo_TE_86638	0.68182(30/44)	0.40000(6/15)	0.04545(1/22)	0.05263(1/19)
Bo_TE_70488	0.83333(35/42)	0.94444(17/18)	0.38095(8/21)	0.33333(6/18)
Bo_TE_27980	0.79545(35/44)	0.11765(2/17)	0.45000(9/20)	0.10000(2/20)
Bo_TE_197944	0.50000(22/44)	0.00000(0/17)	0.75000(15/20)	0.75000(15/20)
Bo_TE_5480	0.20455(9/44)	0.87500(14/16)	0.78947(15/19)	0.85000(17/20)
Bo_TE_44510	1.00000(39/39)	0.41176(7/17)	0.45000(9/20)	0.84211(16/19)
Bo_TE_111585	0.81395(35/43)	1.00000(19/19)	0.25000(5/20)	1.00000(18/18)
Bo_TE_43401	0.90909(40/44)	0.38889(7/18)	1.00000(23/23)	1.00000(20/20)
Bo_TE_24206	1.00000(44/44)	1.00000(19/19)	0.70000(14/20)	0.36842(7/19)
Bo_TE_184367	0.07143(3/42)	0.47368(9/19)	0.68421(13/19)	0.00000(0/20)
Bo_TE_69708	0.53488(23/43)	0.00000(0/18)	0.22727(5/22)	0.50000(10/20)
Bo_TE_193210	0.34884(15/43)	0.58824(10/17)	0.16667(3/18)	0.05000(1/20)
Bo_TE_42041	0.19048(8/42)	0.80000(12/15)	0.70000(14/20)	0.73684(14/19)
Bo_TE_15672	0.58537(24/41)	0.43750(7/16)	0.35000(7/20)	0.00000(0/20)
Bo_TE_208831	0.00000(0/37)	0.00000(0/16)	0.23529(4/17)	0.66667(12/18)
Bo_TE_213648	0.62791(27/43)	0.68750(11/16)	0.09091(2/22)	0.05000(1/20)
Bo_TE_68598	0.00000(0/44)	0.50000(9/18)	0.00000(0/21)	0.15000(3/20)
Bo_TE_30047	0.02174(1/46)	0.23529(4/17)	0.57143(12/21)	0.75000(15/20)
Bo_TE_65614	0.23810(10/42)	0.33333(6/18)	0.04545(1/22)	0.63158(12/19)
Bo_TE_8009	0.97727(43/44)	1.00000(16/16)	0.50000(9/18)	0.44444(8/18)
Bo_TE_106381	0.13953(6/43)	0.00000(0/16)	0.52174(12/23)	0.10000(2/20)
Bo_TE_99833	0.70270(26/37)	0.46154(6/13)	0.00000(0/21)	0.00000(0/20)
Bo_TE_239841	0.90476(38/42)	0.33333(6/18)	0.95455(21/22)	0.90000(18/20)
Bo_TE_38904	0.00000(0/44)	0.11111(2/18)	0.17391(4/23)	0.57895(11/19)
Bo_TE_135054	0.13636(6/44)	0.64706(11/17)	0.00000(0/22)	0.00000(0/20)
Bo_TE_10813	0.87805(36/41)	0.81250(13/16)	0.81818(18/22)	0.10526(2/19)
Bo_TE_108043	0.00000(0/46)	0.88889(16/18)	0.00000(0/23)	0.00000(0/20)
Bo_TE_153329	0.69767(30/43)	0.66667(12/18)	0.85714(18/21)	0.25000(5/20)

Bo_TE_191029	0.73684(28/38)	0.10526(2/19)	0.00000(0/22)	0.10526(2/19)
Bo_TE_96028	0.72093(31/43)	0.47059(8/17)	0.00000(0/21)	0.00000(0/20)
Bo_TE_43147	0.06667(3/45)	0.56250(9/16)	0.00000(0/22)	0.00000(0/20)
Bo_TE_24957	0.80000(32/40)	0.00000(0/17)	0.00000(0/23)	0.00000(0/20)
Bo_TE_165004	0.83333(35/42)	0.43750(7/16)	0.23810(5/21)	0.22222(4/18)
Bo_TE_66488	0.46512(20/43)	0.31579(6/19)	0.85714(18/21)	0.57895(11/19)
Bo_TE_163626	0.20455(9/44)	0.12500(2/16)	0.19048(4/21)	0.70000(14/20)
Bo_TE_7643	0.00000(0/41)	0.37500(6/16)	0.68421(13/19)	0.41176(7/17)
Bo_TE_124908	0.71111(32/45)	0.46667(7/15)	0.91304(21/23)	0.15789(3/19)
Bo_TE_10878	0.11364(5/44)	0.22222(4/18)	0.04545(1/22)	0.84211(16/19)
Bo_TE_142557	0.69767(30/43)	0.82353(14/17)	0.86364(19/22)	0.35294(6/17)
Bo_TE_2480	0.59524(25/42)	0.11765(2/17)	0.00000(0/23)	0.00000(0/20)
Bo_TE_127157	0.00000(0/44)	0.09091(1/11)	0.80000(16/20)	0.60000(12/20)
Bo_TE_21974	0.97778(44/45)	0.84211(16/19)	0.47368(9/19)	1.00000(20/20)
Bo_TE_232177	0.00000(0/46)	0.31250(5/16)	0.33333(7/21)	0.52632(10/19)
Bo_TE_92286	0.54545(24/44)	0.27778(5/18)	0.04545(1/22)	0.16667(3/18)
Bo_TE_11131	0.11111(5/45)	0.82353(14/17)	0.26087(6/23)	0.57895(11/19)
Bo_TE_120396	0.88889(40/45)	0.77778(14/18)	0.52381(11/21)	0.26316(5/19)
Bo_TE_144331	0.04545(2/44)	0.05263(1/19)	0.57143(12/21)	0.50000(10/20)
Bo_TE_24008	0.64286(27/42)	0.35294(6/17)	0.73684(14/19)	0.90000(18/20)
Bo_TE_226515	0.00000(0/43)	0.58824(10/17)	0.00000(0/23)	0.00000(0/19)
Bo_TE_211258	0.00000(0/46)	0.05263(1/19)	0.00000(0/22)	0.56250(9/16)
Bo_TE_53319	1.00000(44/44)	0.64706(11/17)	0.50000(10/20)	0.72222(13/18)
Bo_TE_57450	0.35556(16/45)	1.00000(18/18)	0.45000(9/20)	0.90000(18/20)
Bo_TE_96660	0.81395(35/43)	0.29412(5/17)	0.09091(2/22)	0.50000(10/20)
Bo_TE_96281	0.59091(26/44)	0.05263(1/19)	0.00000(0/23)	0.05000(1/20)
Bo_TE_235116	0.00000(0/44)	0.52632(10/19)	0.04348(1/23)	0.38889(7/18)
Bo_TE_56050	0.51282(20/39)	0.00000(0/17)	0.81818(18/22)	0.52632(10/19)
Bo_TE_10687	0.06818(3/44)	0.66667(10/15)	0.78947(15/19)	0.05556(1/18)
Bo_TE_153287	0.72727(32/44)	0.00000(0/19)	0.04348(1/23)	0.47059(8/17)
Bo_TE_6047	0.09302(4/43)	0.00000(0/14)	0.71429(15/21)	0.84211(16/19)
Bo_TE_6432	0.28571(12/42)	0.56250(9/16)	0.91304(21/23)	1.00000(20/20)
Bo_TE_7371	0.76316(29/38)	0.47059(8/17)	0.11111(2/18)	0.11111(2/18)
Bo_TE_106995	0.25714(9/35)	0.61111(11/18)	0.00000(0/20)	0.61111(11/18)
Bo_TE_221681	0.22222(10/45)	0.00000(0/18)	0.50000(11/22)	0.89474(17/19)
Bo_TE_181143	0.28571(12/42)	0.00000(0/18)	0.61905(13/21)	0.05000(1/20)
Bo_TE_73185	0.53846(21/39)	0.11765(2/17)	0.59091(13/22)	0.85000(17/20)
Bo_TE_42294	0.06667(3/45)	0.36842(7/19)	0.71429(15/21)	0.50000(10/20)
Bo_TE_119447	0.31111(14/45)	0.77778(14/18)	0.42857(9/21)	0.10526(2/19)
Bo_TE_181366	0.72727(32/44)	0.06250(1/16)	0.00000(0/23)	0.40000(8/20)
Bo_TE_105738	0.04545(2/44)	0.50000(8/16)	0.04348(1/23)	0.00000(0/20)
Bo_TE_19373	0.00000(0/44)	0.05882(1/17)	0.66667(14/21)	0.31579(6/19)
Bo_TE_141525	0.14286(6/42)	0.92857(13/14)	0.94118(16/17)	0.76471(13/17)
Bo_TE_178039	0.00000(0/43)	0.50000(8/16)	0.00000(0/23)	0.00000(0/19)
Bo_TE_146763	0.85366(35/41)	1.00000(19/19)	0.50000(10/20)	0.85000(17/20)
Bo_TE_70484	0.11905(5/42)	0.05556(1/18)	0.59091(13/22)	0.73684(14/19)
Bo_TE_89881	0.59524(25/42)	0.11765(2/17)	0.61905(13/21)	0.84211(16/19)
Bo_TE_93354	0.30000(12/40)	0.35294(6/17)	1.00000(21/21)	0.52632(10/19)
Bo_TE_108055	0.00000(0/44)	0.88889(16/18)	0.00000(0/22)	0.00000(0/20)
Bo_TE_140946	0.19512(8/41)	0.94118(16/17)	0.89474(17/19)	0.78947(15/19)
Bo_TE_123984	0.20000(9/45)	0.00000(0/18)	0.08696(2/23)	0.83333(15/18)
Bo_TE_143591	0.83721(36/43)	0.25000(4/16)	0.45000(9/20)	0.75000(15/20)
Bo_TE_152126	0.57143(24/42)	0.00000(0/16)	0.19048(4/21)	0.05000(1/20)
Bo_TE_122845	0.00000(0/44)	0.66667(12/18)	0.00000(0/20)	0.05556(1/18)
Bo_TE_91499	0.88372(38/43)	0.52941(9/17)	0.21739(5/23)	0.47368(9/19)
Bo_TE_138493	0.27907(12/43)	0.16667(3/18)	0.91304(21/23)	0.63158(12/19)

Bo_TE_156904	0.06818(3/44)	0.00000(0/16)	0.14286(3/21)	0.84211(16/19)
Bo_TE_122142	0.44737(17/38)	0.64706(11/17)	1.00000(20/20)	0.50000(7/14)
Bo_TE_38378	0.27500(11/40)	0.11111(2/18)	0.04348(1/23)	0.70000(14/20)
Bo_TE_100041	0.43902(18/41)	0.05556(1/18)	0.61905(13/21)	0.82353(14/17)
Bo_TE_148536	0.97674(42/43)	0.11111(2/18)	1.00000(22/22)	0.21053(4/19)
Bo_TE_218910	0.67500(27/40)	0.11765(2/17)	0.82353(14/17)	0.84211(16/19)
Bo_TE_138279	0.22222(10/45)	0.52632(10/19)	0.18182(4/22)	0.88889(16/18)
Bo_TE_70036	0.32609(15/46)	0.94118(16/17)	0.19048(4/21)	0.65000(13/20)
Bo_TE_199064	0.15909(7/44)	0.60000(9/15)	0.72727(16/22)	0.00000(0/20)
Bo_TE_212017	0.90000(36/40)	0.94444(17/18)	0.25000(5/20)	0.78947(15/19)
Bo_TE_132687	0.71795(28/39)	0.68750(11/16)	0.42857(9/21)	0.17647(3/17)
Bo_TE_132437	0.90476(38/42)	0.29412(5/17)	0.10526(2/19)	0.35294(6/17)
Bo_TE_108136	0.00000(0/45)	0.00000(0/17)	0.22727(5/22)	0.52632(10/19)
Bo_TE_20259	0.87805(36/41)	0.23529(4/17)	0.86364(19/22)	0.83333(15/18)
Bo_TE_42561	0.93333(42/45)	0.82353(14/17)	0.35000(7/20)	0.63158(12/19)
Bo_TE_186181	0.06667(3/45)	0.29412(5/17)	0.65000(13/20)	0.05263(1/19)
Bo_TE_57527	0.48837(21/43)	0.76471(13/17)	0.45455(10/22)	0.05000(1/20)
Bo_TE_173626	0.00000(0/46)	0.00000(0/18)	0.65000(13/20)	0.65000(13/20)
Bo_TE_3798	0.11628(5/43)	0.70588(12/17)	0.28571(6/21)	0.70000(14/20)
Bo_TE_204568	0.02222(1/45)	0.00000(0/18)	0.00000(0/23)	0.50000(9/18)
Bo_TE_39270	0.30233(13/43)	0.37500(6/16)	0.86957(20/23)	1.00000(17/17)
Bo_TE_47920	0.50000(19/38)	0.88889(16/18)	0.00000(0/22)	0.20000(4/20)
Bo_TE_142591	0.61905(26/42)	0.17647(3/17)	0.00000(0/22)	0.28571(4/14)
Bo_TE_26862	0.07143(3/42)	0.11111(2/18)	0.18750(3/16)	0.63158(12/19)
Bo_TE_239662	0.14286(6/42)	0.56250(9/16)	1.00000(23/23)	1.00000(18/18)
Bo_TE_148406	0.90909(40/44)	0.87500(14/16)	0.81818(18/22)	0.35000(7/20)
Bo_TE_20347	0.11111(5/45)	0.81250(13/16)	0.13636(3/22)	0.20000(4/20)
Bo_TE_36847	0.00000(0/44)	0.50000(8/16)	0.19048(4/21)	0.00000(0/20)
Bo_TE_224708	0.79070(34/43)	0.88235(15/17)	0.09524(2/21)	0.05000(1/20)
Bo_TE_67869	0.14634(6/41)	0.36842(7/19)	0.45455(10/22)	0.77778(14/18)
Bo_TE_191918	0.81395(35/43)	0.33333(6/18)	0.33333(6/18)	1.00000(20/20)
Bo_TE_159013	0.00000(0/43)	0.23529(4/17)	0.84211(16/19)	0.11765(2/17)
Bo_TE_85479	0.79545(35/44)	0.00000(0/17)	0.00000(0/17)	0.66667(6/9)
Bo_TE_14627	0.33333(11/33)	0.35294(6/17)	0.72222(13/18)	0.94737(18/19)
Bo_TE_93237	0.50000(21/42)	0.82353(14/17)	0.82609(19/23)	0.31579(6/19)
Bo_TE_169848	0.00000(0/43)	0.68421(13/19)	0.00000(0/22)	0.05000(1/20)
Bo_TE_119394	0.91111(41/45)	0.37500(6/16)	0.45455(10/22)	0.95000(19/20)
Bo_TE_93693	0.04444(2/45)	0.87500(14/16)	0.00000(0/22)	0.00000(0/20)
Bo_TE_205950	0.00000(0/41)	0.05263(1/19)	0.89474(17/19)	0.85000(17/20)
Bo_TE_177613	0.93478(43/46)	0.94118(16/17)	0.50000(10/20)	0.30000(6/20)
Bo_TE_217790	0.76190(32/42)	0.70588(12/17)	0.55000(11/20)	0.10526(2/19)
Bo_TE_162223	0.66667(26/39)	0.10526(2/19)	0.42857(9/21)	0.89474(17/19)
Bo_TE_153796	0.26190(11/42)	0.88889(16/18)	0.04545(1/22)	0.11111(2/18)
Bo_TE_109570	0.13514(5/37)	0.25000(4/16)	0.47619(10/21)	1.00000(18/18)
Bo_TE_234614	0.87500(35/40)	0.25000(4/16)	0.33333(7/21)	0.78571(11/14)
Bo_TE_71001	0.92308(36/39)	0.38889(7/18)	1.00000(22/22)	0.88889(16/18)
Bo_TE_179538	0.83721(36/43)	0.82353(14/17)	0.00000(0/23)	0.75000(12/16)
Bo_TE_5201	0.71429(30/42)	0.17647(3/17)	0.00000(0/19)	0.05882(1/17)
Bo_TE_27634	0.95556(43/45)	0.94737(18/19)	0.13636(3/22)	0.20000(4/20)
Bo_TE_108918	0.52500(21/40)	0.00000(0/17)	0.00000(0/23)	0.05263(1/19)
Bo_TE_41240	0.06667(3/45)	0.70588(12/17)	0.31818(7/22)	0.65000(13/20)
Bo_TE_69289	0.25000(11/44)	0.41176(7/17)	0.95652(22/23)	1.00000(19/19)
Bo_TE_81700	0.33333(14/42)	0.72222(13/18)	0.26087(6/23)	0.20000(4/20)
Bo_TE_213960	0.30952(13/42)	0.25000(4/16)	0.65000(13/20)	0.94737(18/19)
Bo_TE_172560	0.12500(5/40)	0.93750(15/16)	0.10526(2/19)	0.77778(14/18)
Bo_TE_169241	0.83333(35/42)	0.05882(1/17)	0.22727(5/22)	0.31579(6/19)

Bo_TE_30109	0.54545(24/44)	0.27778(5/18)	0.63158(12/19)	0.05556(1/18)
Bo_TE_11620	0.52381(22/42)	0.33333(6/18)	0.04545(1/22)	0.70000(14/20)
Bo_TE_141030	0.89130(41/46)	0.05556(1/18)	0.09524(2/21)	0.15789(3/19)
Bo_TE_238754	0.50000(20/40)	0.06667(1/15)	0.00000(0/13)	0.00000(0/18)
Bo_TE_1152	0.55814(24/43)	0.31250(5/16)	0.31818(7/22)	0.95000(19/20)
Bo_TE_34997	0.19512(8/41)	0.00000(0/19)	0.68421(13/19)	0.75000(15/20)
Bo_TE_130879	0.39394(13/33)	0.77778(14/18)	0.47368(9/19)	0.15789(3/19)
Bo_TE_138436	0.16279(7/43)	0.05263(1/19)	0.15000(3/20)	0.73684(14/19)
Bo_TE_215577	0.22727(5/22)	0.88235(15/17)	0.81818(18/22)	0.05000(1/20)
Bo_TE_159594	0.53488(23/43)	0.23529(4/17)	0.73684(14/19)	0.88235(15/17)
Bo_TE_232352	0.00000(0/46)	0.33333(5/15)	0.42857(9/21)	0.50000(10/20)
Bo_TE_83543	0.55263(21/38)	0.47059(8/17)	0.40000(8/20)	0.00000(0/20)
Bo_TE_196485	0.84783(39/46)	0.50000(9/18)	0.00000(0/23)	0.00000(0/20)
Bo_TE_42630	0.04651(2/43)	0.05882(1/17)	0.57895(11/19)	0.47368(9/19)
Bo_TE_137107	0.06667(3/45)	0.64706(11/17)	0.33333(7/21)	0.22222(4/18)
Bo_TE_148438	0.93333(42/45)	0.35294(6/17)	1.00000(23/23)	0.90000(18/20)
Bo_TE_230109	0.00000(0/46)	0.64706(11/17)	0.14286(3/21)	0.00000(0/20)
Bo_TE_170816	0.97619(41/42)	0.89474(17/19)	0.45455(10/22)	1.00000(19/19)
Bo_TE_50291	0.79070(34/43)	0.18750(3/16)	0.36842(7/19)	0.17647(3/17)
Bo_TE_58650	0.39024(16/41)	0.76471(13/17)	0.13636(3/22)	0.70000(14/20)
Bo_TE_27418	0.00000(0/38)	0.00000(0/16)	0.50000(10/20)	0.23077(3/13)
Bo_TE_225164	0.58974(23/39)	0.05556(1/18)	0.00000(0/23)	0.00000(0/18)
Bo_TE_226716	0.60976(25/41)	0.83333(15/18)	0.33333(7/21)	0.90000(18/20)
Bo_TE_81573	0.56098(23/41)	1.00000(17/17)	0.63636(14/22)	0.45000(9/20)
Bo_TE_77096	0.81395(35/43)	0.52941(9/17)	0.38095(8/21)	0.15000(3/20)
Bo_TE_177667	0.00000(0/44)	0.73684(14/19)	0.04348(1/23)	0.10526(2/19)
Bo_TE_169797	0.21429(9/42)	0.11111(2/18)	0.85000(17/20)	0.61111(11/18)
Bo_TE_111835	0.69767(30/43)	0.94118(16/17)	0.23810(5/21)	1.00000(17/17)
Bo_TE_178741	0.04545(2/44)	0.72222(13/18)	0.00000(0/16)	0.10526(2/19)
Bo_TE_182779	0.35000(14/40)	0.83333(10/12)	0.81818(18/22)	0.94737(18/19)
Bo_TE_72782	0.28571(12/42)	0.52632(10/19)	0.00000(0/23)	0.00000(0/19)
Bo_TE_237486	0.46341(19/41)	0.50000(8/16)	1.00000(23/23)	1.00000(19/19)
Bo_TE_189186	0.72093(31/43)	0.81250(13/16)	0.29412(5/17)	0.20000(4/20)
Bo_TE_169785	0.06977(3/43)	0.77778(14/18)	0.36364(8/22)	0.41176(7/17)
Bo_TE_149896	0.95556(43/45)	0.72222(13/18)	0.90000(18/20)	0.33333(6/18)
Bo_TE_80469	0.93333(42/45)	0.81250(13/16)	0.55556(10/18)	0.15789(3/19)
Bo_TE_119106	0.08889(4/45)	0.68750(11/16)	0.55000(11/20)	0.63158(12/19)
Bo_TE_183842	0.75556(34/45)	0.06250(1/16)	0.00000(0/21)	0.21053(4/19)
Bo_TE_178239	0.34146(14/41)	0.94444(17/18)	1.00000(23/23)	1.00000(20/20)
Bo_TE_77074	0.79070(34/43)	0.52941(9/17)	0.68421(13/19)	0.10526(2/19)
Bo_TE_61740	0.65854(27/41)	0.70588(12/17)	0.33333(7/21)	0.16667(3/18)
Bo_TE_67749	0.76744(33/43)	0.77778(14/18)	0.78947(15/19)	0.15000(3/20)
Bo_TE_224478	0.69767(30/43)	0.10526(2/19)	0.71429(15/21)	0.16667(3/18)
Bo_TE_39818	0.59524(25/42)	0.47059(8/17)	0.22727(5/22)	0.05263(1/19)
Bo_TE_100138	0.86364(38/44)	0.94737(18/19)	0.36364(8/22)	0.52632(10/19)
Bo_TE_145493	0.51220(21/41)	0.05556(1/18)	0.68182(15/22)	0.61111(11/18)
Bo_TE_97678	0.00000(0/45)	0.76471(13/17)	0.09091(2/22)	0.63158(12/19)
Bo_TE_137667	0.54545(24/44)	0.11111(2/18)	0.61905(13/21)	0.10000(2/20)
Bo_TE_237158	0.89474(34/38)	0.82353(14/17)	0.00000(0/21)	0.00000(0/17)
Bo_TE_154374	0.16279(7/43)	0.76471(13/17)	0.13636(3/22)	0.00000(0/20)
Bo_TE_224675	0.41463(17/41)	0.83333(15/18)	0.33333(7/21)	0.89474(17/19)
Bo_TE_104635	0.18605(8/43)	0.22222(4/18)	0.72727(16/22)	0.00000(0/20)
Bo_TE_234583	0.07500(3/40)	0.14286(2/14)	0.29412(5/17)	0.58824(10/17)
Bo_TE_237899	0.36842(14/38)	0.30000(3/10)	1.00000(12/12)	1.00000(18/18)
Bo_TE_111712	0.34211(13/38)	0.05556(1/18)	0.77273(17/22)	0.00000(0/17)
Bo_TE_69275	0.04545(2/44)	0.57895(11/19)	0.00000(0/23)	0.16667(3/18)

Bo_TE_151352	0.02222(1/45)	0.10526(2/19)	0.38095(8/21)	0.60000(12/20)
Bo_TE_70288	0.71053(27/38)	0.62500(10/16)	0.15000(3/20)	0.27778(5/18)
Bo_TE_37340	0.60000(24/40)	0.25000(4/16)	1.00000(21/21)	0.95000(19/20)
Bo_TE_97726	0.00000(0/44)	0.75000(12/16)	0.66667(12/18)	0.66667(12/18)
Bo_TE_208466	0.75000(33/44)	0.35294(6/17)	0.85714(18/21)	0.90000(18/20)
Bo_TE_123801	0.42500(17/40)	0.11111(2/18)	0.68421(13/19)	0.37500(6/16)
Bo_TE_69189	0.86667(39/45)	0.72222(13/18)	0.45455(10/22)	0.10526(2/19)
Bo_TE_219192	0.04444(2/45)	0.12500(2/16)	0.52381(11/21)	0.84211(16/19)
Bo_TE_49615	0.60526(23/38)	0.05882(1/17)	0.80952(17/21)	0.61111(11/18)
Bo_TE_215793	0.11628(5/43)	0.31250(5/16)	0.55000(11/20)	0.65000(13/20)
Bo_TE_37238	0.86667(39/45)	0.43750(7/16)	1.00000(22/22)	0.95000(19/20)
Bo_TE_203191	0.34146(14/41)	0.17647(3/17)	0.47619(10/21)	0.94737(18/19)
Bo_TE_69175	0.60976(25/41)	0.47059(8/17)	0.52381(11/21)	0.00000(0/18)
Bo_TE_97695	0.00000(0/44)	0.72222(13/18)	0.76190(16/21)	0.80000(16/20)
Bo_TE_98178	0.02273(1/44)	0.00000(0/17)	0.09524(2/21)	0.50000(9/18)
Bo_TE_86264	0.73333(33/45)	0.22222(4/18)	0.26087(6/23)	0.90000(18/20)
Bo_TE_112284	0.71795(28/39)	0.05263(1/19)	0.00000(0/23)	0.00000(0/20)
Bo_TE_102499	0.09091(4/44)	0.84211(16/19)	0.40909(9/22)	0.52632(10/19)
Bo_TE_215821	0.40000(18/45)	0.33333(6/18)	1.00000(23/23)	0.44444(8/18)
Bo_TE_238498	0.06977(3/43)	0.50000(9/18)	0.00000(0/22)	0.00000(0/18)
Bo_TE_66135	0.11628(5/43)	0.22222(4/18)	0.75000(15/20)	0.38889(7/18)
Bo_TE_56831	0.50000(21/42)	0.61111(11/18)	1.00000(23/23)	0.90000(18/20)
Bo_TE_193607	0.65909(29/44)	0.41176(7/17)	0.84211(16/19)	0.95000(19/20)
Bo_TE_154278	0.93023(40/43)	0.18750(3/16)	1.00000(22/22)	1.00000(20/20)
Bo_TE_224138	0.57143(20/35)	0.29412(5/17)	0.77273(17/22)	1.00000(18/18)
Bo_TE_100867	0.00000(0/43)	0.13333(2/15)	0.73684(14/19)	0.63158(12/19)
Bo_TE_163590	0.08889(4/45)	0.00000(0/18)	0.14286(3/21)	0.65000(13/20)
Bo_TE_4085	0.55556(25/45)	0.00000(0/18)	0.00000(0/23)	0.10000(2/20)
Bo_TE_184733	0.00000(0/44)	0.66667(10/15)	0.50000(11/22)	0.41176(7/17)
Bo_TE_197981	0.97778(44/45)	0.52941(9/17)	0.14286(3/21)	0.35294(6/17)
Bo_TE_91701	0.72500(29/40)	0.50000(8/16)	1.00000(20/20)	0.89474(17/19)
Bo_TE_206722	0.43182(19/44)	0.82353(14/17)	1.00000(23/23)	0.72222(13/18)
Bo_TE_155059	0.13333(6/45)	0.00000(0/18)	0.27273(6/22)	0.70000(14/20)
Bo_TE_10254	0.87179(34/39)	0.05882(1/17)	0.50000(11/22)	0.11765(2/17)
Bo_TE_68299	0.79412(27/34)	0.70588(12/17)	0.18182(4/22)	0.13333(2/15)
Bo_TE_10267	0.00000(0/41)	0.06250(1/16)	0.57143(12/21)	0.63158(12/19)
Bo_TE_138765	0.62791(27/43)	0.21053(4/19)	0.35000(7/20)	0.83333(15/18)
Bo_TE_143606	0.80435(37/46)	0.25000(4/16)	0.50000(10/20)	0.58824(10/17)
Bo_TE_201344	0.00000(0/46)	0.52941(9/17)	0.00000(0/23)	0.05263(1/19)
Bo_TE_114444	0.33333(14/42)	0.58824(10/17)	0.04762(1/21)	0.44444(8/18)
Bo_TE_129106	0.72093(31/43)	0.17647(3/17)	0.86364(19/22)	0.95000(19/20)
Bo_TE_3161	0.60870(28/46)	0.37500(6/16)	0.04348(1/23)	0.44444(8/18)
Bo_TE_42434	0.32558(14/43)	0.27778(5/18)	0.04545(1/22)	0.80000(16/20)
Bo_TE_69828	0.55000(22/40)	0.44444(8/18)	0.33333(7/21)	0.90000(18/20)
Bo_TE_191630	0.76744(33/43)	0.16667(3/18)	0.04348(1/23)	0.00000(0/18)
Bo_TE_237279	0.02174(1/46)	0.84211(16/19)	0.45000(9/20)	0.50000(9/18)
Bo_TE_177796	1.00000(45/45)	0.87500(14/16)	0.22727(5/22)	0.38889(7/18)
Bo_TE_117501	0.95455(42/44)	0.17647(3/17)	0.50000(10/20)	0.55556(10/18)
Bo_TE_211346	0.62791(27/43)	0.05263(1/19)	0.00000(0/20)	0.84211(16/19)
Bo_TE_55638	0.53846(21/39)	0.88235(15/17)	0.65000(13/20)	0.05556(1/18)
Bo_TE_102665	0.48837(21/43)	1.00000(19/19)	1.00000(20/20)	0.84211(16/19)
Bo_TE_203715	0.65116(28/43)	0.25000(4/16)	0.84211(16/19)	0.85000(17/20)
Bo_TE_8406	0.68421(26/38)	0.83333(15/18)	0.43750(7/16)	0.31250(5/16)
Bo_TE_80039	0.69767(30/43)	0.00000(0/17)	0.55000(11/20)	0.36842(7/19)
Bo_TE_124188	0.22222(10/45)	0.76471(13/17)	0.80952(17/21)	0.85000(17/20)
Bo_TE_87599	0.04348(2/46)	0.05882(1/17)	0.55000(11/20)	0.26316(5/19)

Bo_TE_213932	0.30233(13/43)	0.50000(8/16)	0.00000(0/23)	0.00000(0/20)
Bo_TE_150554	0.08696(4/46)	0.56250(9/16)	0.63636(14/22)	0.60000(12/20)
Bo_TE_68044	0.35714(15/42)	0.82353(14/17)	0.19048(4/21)	0.73684(14/19)
Bo_TE_61387	0.59524(25/42)	0.10526(2/19)	0.90476(19/21)	0.85000(17/20)
Bo_TE_236867	0.45238(19/42)	0.64706(11/17)	0.27273(6/22)	0.10000(2/20)
Bo_TE_231458	0.02326(1/43)	0.00000(0/18)	0.19048(4/21)	0.52941(9/17)
Bo_TE_154	0.02381(1/42)	0.56250(9/16)	0.22727(5/22)	0.55000(11/20)
Bo_TE_123924	0.28261(13/46)	0.94737(18/19)	0.80000(16/20)	0.95000(19/20)
Bo_TE_189675	0.97826(45/46)	0.88889(16/18)	0.45455(10/22)	1.00000(20/20)
Bo_TE_156998	0.56818(25/44)	0.87500(14/16)	0.27273(6/22)	0.70000(14/20)
Bo_TE_211614	0.20930(9/43)	0.62500(10/16)	0.00000(0/23)	0.45000(9/20)
Bo_TE_16248	0.41860(18/43)	0.31250(5/16)	0.26316(5/19)	0.81250(13/16)
Bo_TE_211757	0.13333(6/45)	0.47368(9/19)	0.22727(5/22)	0.65000(13/20)
Bo_TE_112807	0.32558(14/43)	0.64706(11/17)	0.04348(1/23)	0.40000(8/20)
Bo_TE_40377	0.24444(11/45)	0.83333(15/18)	1.00000(22/22)	0.85000(17/20)
Bo_TE_185402	1.00000(43/43)	0.43750(7/16)	0.68750(11/16)	0.82353(14/17)
Bo_TE_42333	0.61538(24/39)	0.88889(16/18)	0.04545(1/22)	0.05000(1/20)
Bo_TE_16751	0.00000(0/44)	0.16667(3/18)	0.68421(13/19)	0.57895(11/19)
Bo_TE_82138	0.78571(33/42)	0.70588(12/17)	0.42857(9/21)	0.00000(0/20)
Bo_TE_178109	0.32558(14/43)	0.61111(11/18)	1.00000(22/22)	0.30000(6/20)
Bo_TE_229614	0.00000(0/44)	0.10526(2/19)	0.68182(15/22)	0.68421(13/19)
Bo_TE_33737	0.73171(30/41)	0.27778(5/18)	0.09091(2/22)	0.00000(0/19)
Bo_TE_21223	0.95556(43/45)	0.43750(7/16)	0.70000(14/20)	0.33333(6/18)
Bo_TE_225104	0.13043(6/46)	0.36842(7/19)	0.80952(17/21)	0.00000(0/19)
Bo_TE_81142	0.00000(0/45)	0.00000(0/19)	0.70000(14/20)	0.00000(0/20)
Bo_TE_197289	1.00000(44/44)	0.89474(17/19)	1.00000(23/23)	0.50000(9/18)
Bo_TE_212814	0.63415(26/41)	0.05263(1/19)	0.04348(1/23)	0.05000(1/20)
Bo_TE_211465	0.64286(27/42)	0.22222(4/18)	0.00000(0/23)	0.58824(10/17)
Bo_TE_158549	0.20455(9/44)	0.46667(7/15)	1.00000(14/14)	0.84211(16/19)
Bo_TE_81435	0.24324(9/37)	0.05263(1/19)	0.09091(2/22)	0.63158(12/19)
Bo_TE_37598	0.66667(28/42)	0.76471(13/17)	0.04762(1/21)	0.36842(7/19)
Bo_TE_174999	0.00000(0/44)	0.00000(0/18)	0.04762(1/21)	0.62500(10/16)
Bo_TE_60744	0.35000(14/40)	0.75000(12/16)	0.90909(20/22)	0.52632(10/19)
Bo_TE_19213	0.04444(2/45)	0.05263(1/19)	0.13636(3/22)	0.61111(11/18)
Bo_TE_171872	0.00000(0/46)	0.00000(0/18)	0.13043(3/23)	0.73684(14/19)
Bo_TE_115329	0.05000(2/40)	0.82353(14/17)	0.13636(3/22)	0.16667(3/18)
Bo_TE_117508	0.04878(2/41)	0.77778(14/18)	0.55556(10/18)	0.47368(9/19)
Bo_TE_155299	0.52778(19/36)	0.82353(14/17)	0.25000(5/20)	0.62500(10/16)
Bo_TE_224848	0.60976(25/41)	0.11765(2/17)	1.00000(20/20)	1.00000(20/20)
Bo_TE_21123	0.15909(7/44)	0.88889(16/18)	0.52381(11/21)	0.89474(17/19)
Bo_TE_148337	0.02273(1/44)	0.06250(1/16)	0.75000(15/20)	0.88889(16/18)
Bo_TE_163631	0.80952(34/42)	0.82353(14/17)	0.80952(17/21)	0.31579(6/19)
Bo_TE_226890	0.11905(5/42)	0.58824(10/17)	1.00000(22/22)	0.85000(17/20)
Bo_TE_25875	0.80488(33/41)	0.11111(2/18)	0.45455(10/22)	0.10526(2/19)
Bo_TE_43614	0.79070(34/43)	0.61111(11/18)	0.00000(0/21)	0.40000(8/20)
Bo_TE_26804	0.30233(13/43)	0.11765(2/17)	0.23810(5/21)	0.63158(12/19)
Bo_TE_170633	0.00000(0/46)	0.05263(1/19)	0.52381(11/21)	0.00000(0/20)
Bo_TE_210397	0.67568(25/37)	0.56250(9/16)	0.04348(1/23)	0.25000(5/20)
Bo_TE_211651	0.04545(2/44)	0.00000(0/19)	0.68421(13/19)	0.00000(0/20)
Bo_TE_169734	0.93333(42/45)	0.31250(5/16)	0.25000(5/20)	0.36842(7/19)
Bo_TE_170410	0.58537(24/41)	0.47368(9/19)	0.36364(8/22)	0.00000(0/20)
Bo_TE_112788	0.71111(32/45)	0.23529(4/17)	0.25000(5/20)	0.00000(0/20)
Bo_TE_5274	0.76190(32/42)	0.18750(3/16)	0.61905(13/21)	0.27778(5/18)
Bo_TE_126871	0.00000(0/44)	0.10526(2/19)	0.59091(13/22)	0.00000(0/20)
Bo_TE_210268	0.00000(0/44)	0.16667(3/18)	0.08696(2/23)	0.50000(9/18)
Bo_TE_63114	0.02174(1/46)	0.66667(12/18)	0.57143(12/21)	0.76471(13/17)

Bo_TE_28275	0.80000(24/30)	0.23529(4/17)	0.85000(17/20)	0.50000(9/18)
Bo_TE_224036	0.50000(21/42)	1.00000(19/19)	0.78261(18/23)	1.00000(20/20)
Bo_TE_224096	0.65854(27/41)	1.00000(19/19)	0.57143(12/21)	0.35000(7/20)
Bo_TE_195927	0.97619(41/42)	0.88235(15/17)	0.66667(14/21)	0.29412(5/17)
Bo_TE_127542	0.20930(9/43)	0.61111(11/18)	0.85000(17/20)	1.00000(19/19)
Bo_TE_22945	0.80000(36/45)	0.75000(12/16)	0.25000(5/20)	0.15789(3/19)
Bo_TE_145924	0.97826(45/46)	0.70588(12/17)	0.50000(10/20)	0.41176(7/17)
Bo_TE_191518	0.76744(33/43)	0.05556(1/18)	0.00000(0/22)	0.00000(0/19)
Bo_TE_214112	0.51282(20/39)	0.18750(3/16)	0.77273(17/22)	0.95000(19/20)
Bo_TE_69252	0.02273(1/44)	0.00000(0/17)	0.77273(17/22)	0.45000(9/20)
Bo_TE_67706	0.85714(36/42)	1.00000(17/17)	0.28571(6/21)	0.89474(17/19)
Bo_TE_149658	0.20000(9/45)	0.05556(1/18)	0.59091(13/22)	0.47368(9/19)
Bo_TE_24220	0.00000(0/43)	0.00000(0/19)	0.54545(12/22)	0.63158(12/19)
Bo_TE_107577	0.72222(26/36)	1.00000(16/16)	0.00000(0/16)	0.41176(7/17)
Bo_TE_27030	0.77778(35/45)	0.43750(7/16)	0.47619(10/21)	0.95000(19/20)
Bo_TE_124586	0.07143(3/42)	0.55556(10/18)	0.70588(12/17)	0.05000(1/20)
Bo_TE_45226	0.34146(14/41)	0.21053(4/19)	0.66667(14/21)	0.00000(0/19)
Bo_TE_45889	0.00000(0/43)	0.61111(11/18)	0.04545(1/22)	0.10526(2/19)
Bo_TE_137191	0.43902(18/41)	1.00000(13/13)	1.00000(22/22)	0.75000(6/8)
Bo_TE_81554	0.65854(27/41)	0.11111(2/18)	0.00000(0/20)	0.63158(12/19)
Bo_TE_227711	0.02174(1/46)	0.05556(1/18)	0.42105(8/19)	0.57895(11/19)
Bo_TE_145407	0.68293(28/41)	0.11111(2/18)	0.60000(12/20)	1.00000(20/20)
Bo_TE_209982	0.00000(0/44)	0.61111(11/18)	0.04545(1/22)	0.05000(1/20)
Bo_TE_207048	0.70732(29/41)	0.52941(9/17)	0.00000(0/23)	0.43750(7/16)
Bo_TE_145328	0.18182(8/44)	0.00000(0/18)	0.40000(8/20)	0.63158(12/19)
Bo_TE_219077	0.24390(10/41)	0.94118(16/17)	0.72727(16/22)	0.80000(16/20)
Bo_TE_159324	0.00000(0/38)	0.00000(0/18)	0.10000(2/20)	0.64706(11/17)
Bo_TE_229944	0.27907(12/43)	0.33333(6/18)	1.00000(23/23)	0.77778(14/18)
Bo_TE_201150	0.00000(0/46)	0.61111(11/18)	0.00000(0/23)	0.00000(0/20)
Bo_TE_165525	0.53659(22/41)	0.00000(0/16)	0.31818(7/22)	0.63158(12/19)
Bo_TE_21457	0.60526(23/38)	0.22222(4/18)	0.04545(1/22)	0.05263(1/19)
Bo_TE_207856	0.51111(23/45)	0.17647(3/17)	0.81818(18/22)	0.61111(11/18)
Bo_TE_133217	0.50000(21/42)	0.27778(5/18)	0.00000(0/22)	0.15789(3/19)
Bo_TE_50528	0.93182(41/44)	0.82353(14/17)	0.66667(14/21)	0.15789(3/19)
Bo_TE_142361	0.79070(34/43)	0.27778(5/18)	0.21739(5/23)	0.00000(0/20)
Bo_TE_104258	0.65116(28/43)	0.06250(1/16)	0.86364(19/22)	0.83333(15/18)
Bo_TE_144227	0.88095(37/42)	0.25000(4/16)	0.86957(20/23)	1.00000(20/20)
Bo_TE_126016	0.54545(24/44)	0.66667(12/18)	0.00000(0/23)	0.05263(1/19)
Bo_TE_154559	0.58537(24/41)	0.20000(3/15)	0.00000(0/23)	0.00000(0/20)
Bo_TE_141515	0.82222(37/45)	0.06250(1/16)	0.04545(1/22)	0.25000(5/20)
Bo_TE_188950	0.09756(4/41)	0.50000(8/16)	0.00000(0/23)	0.00000(0/20)
Bo_TE_111892	0.67442(29/43)	0.00000(0/16)	0.80952(17/21)	0.11765(2/17)
Bo_TE_33823	0.73333(33/45)	0.73684(14/19)	0.31818(7/22)	0.94444(17/18)
Bo_TE_62298	0.26190(11/42)	0.55556(10/18)	0.90909(20/22)	0.15000(3/20)
Bo_TE_138329	0.09524(4/42)	0.00000(0/17)	0.54545(12/22)	0.00000(0/19)
Bo_TE_213812	0.11628(5/43)	0.05556(1/18)	0.65000(13/20)	0.58824(10/17)
Bo_TE_133923	0.57143(24/42)	0.00000(0/12)	0.33333(6/18)	0.05263(1/19)
Bo_TE_153998	0.45238(19/42)	0.16667(3/18)	0.90909(20/22)	0.47368(9/19)
Bo_TE_201898	1.00000(44/44)	0.94118(16/17)	0.45455(10/22)	0.52632(10/19)
Bo_TE_114110	0.52500(21/40)	0.16667(3/18)	0.00000(0/22)	0.10000(2/20)
Bo_TE_170935	0.47727(21/44)	0.70588(12/17)	0.61905(13/21)	0.05000(1/20)
Bo_TE_204146	0.64103(25/39)	0.50000(8/16)	0.13636(3/22)	0.52941(9/17)
Bo_TE_45627	0.61905(26/42)	0.06250(1/16)	0.19048(4/21)	0.00000(0/20)
Bo_TE_224682	0.39024(16/41)	0.81250(13/16)	1.00000(23/23)	0.94444(17/18)
Bo_TE_108040	0.00000(0/45)	0.88889(16/18)	0.00000(0/22)	0.00000(0/20)
Bo_TE_13921	0.16667(7/42)	0.93750(15/16)	1.00000(23/23)	1.00000(20/20)

Bo_TE_30190	0.93023(40/43)	0.26316(5/19)	0.15789(3/19)	0.47368(9/19)
Bo_TE_48958	0.02273(1/44)	0.00000(0/18)	0.63636(14/22)	0.52632(10/19)
Bo_TE_42117	0.00000(0/45)	0.00000(0/19)	0.59091(13/22)	0.10000(2/20)
Bo_TE_145332	0.80952(34/42)	0.52941(9/17)	0.21053(4/19)	0.05263(1/19)
Bo_TE_167765	0.06522(3/46)	0.05556(1/18)	0.61905(13/21)	0.05263(1/19)
Bo_TE_216585	0.60000(24/40)	0.25000(4/16)	0.09091(2/22)	0.42105(8/19)
Bo_TE_153982	0.52632(20/38)	0.18750(3/16)	0.73684(14/19)	0.50000(10/20)
Bo_TE_206436	0.54348(25/46)	0.72222(13/18)	0.04348(1/23)	0.70588(12/17)
Bo_TE_47958	0.50000(20/40)	0.88235(15/17)	0.00000(0/21)	0.20000(4/20)
Bo_TE_41876	0.20455(9/44)	0.35294(6/17)	0.90476(19/21)	0.78947(15/19)
Bo_TE_206512	0.12121(4/33)	0.00000(0/17)	0.07143(1/14)	0.76471(13/17)
Bo_TE_9541	0.45238(19/42)	0.64286(9/14)	1.00000(21/21)	0.85000(17/20)
Bo_TE_62474	1.00000(46/46)	1.00000(18/18)	0.19048(4/21)	0.85000(17/20)
Bo_TE_66916	0.00000(0/44)	0.55556(10/18)	0.47619(10/21)	0.73684(14/19)
Bo_TE_28933	0.73684(28/38)	1.00000(18/18)	0.94444(17/18)	0.31579(6/19)
Bo_TE_169411	0.02174(1/46)	0.61111(11/18)	0.78261(18/23)	0.75000(15/20)
Bo_TE_68410	0.50000(22/44)	0.88889(16/18)	0.04762(1/21)	0.31579(6/19)
Bo_TE_215942	0.76744(33/43)	0.33333(6/18)	0.00000(0/22)	0.05556(1/18)
Bo_TE_78176	0.34091(15/44)	0.26316(5/19)	0.50000(11/22)	0.00000(0/20)
Bo_TE_63116	0.02222(1/45)	0.64706(11/17)	0.00000(0/21)	0.20000(4/20)
Bo_TE_129086	0.09524(4/42)	0.05556(1/18)	0.80000(16/20)	0.05000(1/20)
Bo_TE_213307	0.84091(37/44)	0.35294(6/17)	0.43478(10/23)	0.16667(3/18)
Bo_TE_204452	0.83721(36/43)	0.23529(4/17)	0.61905(13/21)	0.11765(2/17)
Bo_TE_15796	0.12195(5/41)	0.25000(4/16)	0.38095(8/21)	0.84211(16/19)
Bo_TE_41209	0.06667(3/45)	0.82353(14/17)	0.61905(13/21)	0.63158(12/19)
Bo_TE_101343	1.00000(43/43)	0.26667(4/15)	0.68421(13/19)	0.11765(2/17)
Bo_TE_153817	1.00000(45/45)	1.00000(19/19)	0.72727(16/22)	0.36842(7/19)
Bo_TE_92313	0.21429(9/42)	0.50000(9/18)	0.00000(0/22)	0.00000(0/20)
Bo_TE_178116	0.70000(28/40)	0.33333(6/18)	0.00000(0/23)	0.05000(1/20)
Bo_TE_199563	0.12500(5/40)	0.06667(1/15)	1.00000(21/21)	0.55556(10/18)
Bo_TE_58146	0.00000(0/38)	0.00000(0/8)	0.18182(4/22)	0.52632(10/19)
Bo_TE_126772	0.88636(39/44)	0.82353(14/17)	0.00000(0/23)	0.10526(2/19)
Bo_TE_37403	0.58974(23/39)	0.05882(1/17)	0.00000(0/23)	0.00000(0/18)
Bo_TE_30128	0.09524(4/42)	0.50000(9/18)	0.00000(0/22)	0.15789(3/19)
Bo_TE_136935	0.66667(28/42)	0.06250(1/16)	0.00000(0/23)	0.22222(4/18)
Bo_TE_104970	0.66667(28/42)	0.00000(0/16)	0.26316(5/19)	0.57895(11/19)
Bo_TE_38769	0.95238(40/42)	0.47059(8/17)	0.95455(21/22)	1.00000(19/19)
Bo_TE_165769	0.59091(26/44)	0.94118(16/17)	1.00000(20/20)	0.31579(6/19)
Bo_TE_111402	0.45000(18/40)	0.29412(5/17)	0.95000(19/20)	0.75000(15/20)
Bo_TE_163570	0.20930(9/43)	0.00000(0/19)	0.19048(4/21)	0.61111(11/18)
Bo_TE_64823	0.02439(1/41)	0.05263(1/19)	0.66667(14/21)	0.47368(9/19)
Bo_TE_56351	0.34091(15/44)	0.53333(8/15)	0.38095(8/21)	0.85000(17/20)
Bo_TE_234961	0.41304(19/46)	0.73684(14/19)	1.00000(22/22)	0.70000(14/20)
Bo_TE_198442	0.11628(5/43)	0.27778(5/18)	0.35000(7/20)	0.95000(19/20)
Bo_TE_129274	0.04762(2/42)	0.00000(0/18)	0.00000(0/7)	0.73684(14/19)
Bo_TE_117347	0.97778(44/45)	0.23529(4/17)	0.35000(7/20)	0.52941(9/17)
Bo_TE_112739	0.23256(10/43)	0.54545(6/11)	1.00000(17/17)	0.94118(16/17)
Bo_TE_90035	0.75610(31/41)	0.21053(4/19)	0.08696(2/23)	0.05263(1/19)
Bo_TE_77548	0.97727(43/44)	0.23529(4/17)	1.00000(22/22)	0.26316(5/19)
Bo_TE_120383	0.09524(4/42)	0.23529(4/17)	0.36364(8/22)	0.78947(15/19)
Bo_TE_56719	0.13636(6/44)	0.58333(7/12)	0.37500(6/16)	0.94737(18/19)
Bo_TE_201367	0.39474(15/38)	0.50000(9/18)	0.22727(5/22)	0.00000(0/20)
Bo_TE_169159	0.59091(26/44)	0.27778(5/18)	0.00000(0/22)	0.00000(0/20)
Bo_TE_32205	0.00000(0/45)	0.07143(1/14)	0.71429(15/21)	0.05263(1/19)
Bo_TE_112448	0.42857(18/42)	0.81250(13/16)	0.95652(22/23)	0.64706(11/17)
Bo_TE_112550	0.41463(17/41)	0.41176(7/17)	0.47619(10/21)	1.00000(20/20)

Bo_TE_216528	0.58537(24/41)	0.11111(2/18)	0.63636(14/22)	0.11111(2/18)
Bo_TE_44025	0.15909(7/44)	0.72222(13/18)	0.04545(1/22)	0.10526(2/19)
Bo_TE_25825	0.78571(33/42)	0.11111(2/18)	0.45000(9/20)	0.11111(2/18)
Bo_TE_226233	0.60526(23/38)	0.22222(4/18)	0.66667(14/21)	0.95000(19/20)
Bo_TE_17567	0.90909(40/44)	0.13333(2/15)	0.11111(2/18)	0.16667(3/18)
Bo_TE_168108	0.18605(8/43)	1.00000(17/17)	0.47619(10/21)	0.36842(7/19)
Bo_TE_89425	0.37209(16/43)	0.66667(12/18)	0.95238(20/21)	1.00000(19/19)
Bo_TE_93081	0.32558(14/43)	0.77778(14/18)	0.95238(20/21)	0.90000(18/20)
Bo_TE_38871	0.04651(2/43)	0.27778(5/18)	0.45455(10/22)	0.72222(13/18)
Bo_TE_230829	0.04348(2/46)	0.38889(7/18)	0.45455(10/22)	0.70000(14/20)
Bo_TE_60968	0.38095(16/42)	0.35294(6/17)	0.90476(19/21)	0.26316(5/19)
Bo_TE_111874	0.21739(10/46)	0.94737(18/19)	0.25000(5/20)	0.78947(15/19)
Bo_TE_46415	0.77500(31/40)	0.13333(2/15)	0.70000(14/20)	0.00000(0/18)
Bo_TE_117495	0.95455(42/44)	0.16667(3/18)	0.50000(10/20)	0.55556(10/18)
Bo_TE_17687	0.28205(11/39)	0.50000(7/14)	0.77273(17/22)	0.95000(19/20)
Bo_TE_108583	0.23077(9/39)	0.16667(3/18)	0.65000(13/20)	0.10000(2/20)
Bo_TE_60972	0.63158(24/38)	0.64706(11/17)	0.13636(3/22)	0.73684(14/19)
Bo_TE_134504	0.25581(11/43)	0.94118(16/17)	1.00000(22/22)	0.78947(15/19)
Bo_TE_154742	0.63415(26/41)	0.64706(11/17)	0.00000(0/22)	0.00000(0/18)
Bo_TE_9498	0.70455(31/44)	0.12500(2/16)	0.05882(1/17)	0.00000(0/17)
Bo_TE_182357	0.06667(3/45)	0.21053(4/19)	0.57143(12/21)	0.45000(9/20)
Bo_TE_92992	0.47619(20/42)	0.82353(14/17)	0.04348(1/23)	0.11111(2/18)
Bo_TE_210970	0.02273(1/44)	0.38889(7/18)	0.60000(12/20)	0.50000(9/18)
Bo_TE_97080	0.44186(19/43)	0.00000(0/17)	0.80952(17/21)	0.42105(8/19)
Bo_TE_225372	0.30000(12/40)	0.00000(0/18)	0.55000(11/20)	0.00000(0/20)
Bo_TE_121885	0.51220(21/41)	0.11765(2/17)	0.45455(10/22)	0.73684(14/19)
Bo_TE_144744	0.86667(39/45)	0.11765(2/17)	0.18182(4/22)	1.00000(18/18)
Bo_TE_70769	0.12500(5/40)	0.05263(1/19)	0.18182(4/22)	0.57895(11/19)
Bo_TE_82100	0.97674(42/43)	1.00000(18/18)	1.00000(22/22)	0.36842(7/19)
Bo_TE_86903	0.86667(39/45)	0.00000(0/16)	0.00000(0/23)	0.05263(1/19)
Bo_TE_97980	0.97674(42/43)	0.88235(15/17)	0.38095(8/21)	0.21053(4/19)
Bo_TE_98	0.95556(43/45)	0.70588(12/17)	0.85714(18/21)	0.35294(6/17)
Bo_TE_42345	0.93023(40/43)	0.88235(15/17)	0.20000(4/20)	0.50000(9/18)
Bo_TE_22379	1.00000(42/42)	0.52941(9/17)	0.22727(5/22)	0.78947(15/19)
Bo_TE_240654	0.76190(32/42)	0.16667(3/18)	0.04545(1/22)	0.47368(9/19)
Bo_TE_87456	0.93182(41/44)	0.64706(11/17)	0.13043(3/23)	0.77778(14/18)
Bo_TE_213049	0.74359(29/39)	0.11111(2/18)	0.33333(7/21)	0.20000(4/20)
Bo_TE_95722	0.50000(21/42)	0.36842(7/19)	0.00000(0/23)	0.05000(1/20)
Bo_TE_174064	0.69048(29/42)	0.66667(12/18)	0.47619(10/21)	0.16667(3/18)
Bo_TE_200844	0.28571(12/42)	0.70588(12/17)	1.00000(23/23)	0.76471(13/17)
Bo_TE_168639	0.58140(25/43)	1.00000(18/18)	0.31579(6/19)	0.20000(4/20)
Bo_TE_181708	0.73333(33/45)	0.17647(3/17)	0.55000(11/20)	0.10526(2/19)
Bo_TE_46922	0.35000(14/40)	0.64706(11/17)	0.20000(4/20)	0.05000(1/20)
Bo_TE_56704	0.13636(6/44)	0.40000(6/15)	0.75000(15/20)	0.95000(19/20)
Bo_TE_184844	0.84444(38/45)	0.41176(7/17)	0.36364(8/22)	1.00000(19/19)
Bo_TE_210711	0.34884(15/43)	0.12500(2/16)	0.70000(14/20)	0.78947(15/19)
Bo_TE_192036	0.17073(7/41)	0.37500(6/16)	0.28571(6/21)	0.77778(14/18)
Bo_TE_237500	0.36842(14/38)	0.50000(8/16)	0.91304(21/23)	0.95000(19/20)
Bo_TE_216858	0.41463(17/41)	1.00000(17/17)	0.80952(17/21)	0.95000(19/20)
Bo_TE_128545	0.69048(29/42)	0.26316(5/19)	0.38095(8/21)	1.00000(20/20)
Bo_TE_137291	0.02273(1/44)	0.42105(8/19)	0.15000(3/20)	0.55000(11/20)
Bo_TE_76263	0.59524(25/42)	0.05556(1/18)	0.66667(14/21)	0.52632(10/19)
Bo_TE_235632	0.25581(11/43)	0.88235(15/17)	0.00000(0/22)	0.30000(6/20)
Bo_TE_125997	0.44186(19/43)	0.29412(5/17)	1.00000(23/23)	0.90000(18/20)
Bo_TE_120896	0.55814(24/43)	0.66667(12/18)	0.14286(3/21)	0.20000(4/20)
Bo_TE_14790	0.14286(6/42)	0.00000(0/17)	0.15000(3/20)	0.65000(13/20)

Bo_TE_113628	0.84211(32/38)	0.83333(15/18)	0.60000(12/20)	0.30000(6/20)
Bo_TE_110905	0.90698(39/43)	0.86667(13/15)	0.84211(16/19)	0.33333(6/18)
Bo_TE_224609	0.60976(25/41)	0.11111(2/18)	0.00000(0/23)	0.05263(1/19)
Bo_TE_45546	0.88636(39/44)	0.00000(0/18)	0.00000(0/20)	0.00000(0/20)
Bo_TE_90493	0.00000(0/45)	0.00000(0/19)	0.63636(14/22)	0.10526(2/19)
Bo_TE_138151	0.71429(30/42)	0.11765(2/17)	0.00000(0/23)	0.00000(0/19)
Bo_TE_129258	0.82051(32/39)	0.76471(13/17)	1.00000(7/7)	0.26316(5/19)
Bo_TE_226591	0.87500(35/40)	0.64706(11/17)	0.75000(15/20)	0.35000(7/20)
Bo_TE_137513	0.67500(27/40)	0.55556(10/18)	0.85714(18/21)	0.20000(4/20)
Bo_TE_89962	0.88372(38/43)	0.52941(9/17)	0.23810(5/21)	0.21053(4/19)
Bo_TE_118525	0.59524(25/42)	0.40000(6/15)	0.13636(3/22)	0.05000(1/20)
Bo_TE_135184	0.65116(28/43)	0.27778(5/18)	1.00000(23/23)	1.00000(19/19)
Bo_TE_58257	0.00000(0/45)	0.00000(0/17)	0.40909(9/22)	0.65000(13/20)
Bo_TE_55444	0.69767(30/43)	0.50000(8/16)	0.28571(6/21)	0.84211(16/19)
Bo_TE_40812	0.06667(3/45)	0.88235(15/17)	0.15789(3/19)	0.36842(7/19)
Bo_TE_179054	0.55000(22/40)	0.87500(14/16)	0.04545(1/22)	0.65000(13/20)
Bo_TE_158036	0.02439(1/41)	0.00000(0/18)	0.50000(11/22)	0.06250(1/16)
Bo_TE_139500	0.58537(24/41)	0.00000(0/16)	0.29412(5/17)	0.47059(8/17)
Bo_TE_59944	1.00000(44/44)	0.94118(16/17)	0.71429(15/21)	0.35000(7/20)
Bo_TE_15705	0.41860(18/43)	0.58824(10/17)	0.65000(13/20)	1.00000(19/19)
Bo_TE_216877	0.22500(9/40)	0.64706(11/17)	0.04545(1/22)	0.00000(0/15)
Bo_TE_103656	0.92683(38/41)	0.38889(7/18)	0.86364(19/22)	0.66667(12/18)
Bo_TE_140062	0.70455(31/44)	0.94444(17/18)	0.47368(9/19)	0.10526(2/19)
Bo_TE_30189	0.93182(41/44)	0.29412(5/17)	0.20000(4/20)	0.47368(9/19)
Bo_TE_196364	0.00000(0/44)	0.52632(10/19)	0.04762(1/21)	0.25000(5/20)
Bo_TE_87379	0.62791(27/43)	0.76471(13/17)	0.00000(0/23)	0.00000(0/20)
Bo_TE_21642	1.00000(44/44)	0.66667(12/18)	0.90909(20/22)	0.10526(2/19)
Bo_TE_39642	0.02439(1/41)	0.50000(7/14)	0.60000(12/20)	0.10526(2/19)
Bo_TE_50405	0.97778(44/45)	0.55556(10/18)	0.86364(19/22)	0.20000(4/20)
Bo_TE_177280	0.86364(38/44)	0.00000(0/18)	0.08696(2/23)	0.84211(16/19)
Bo_TE_62208	0.65854(27/41)	0.78571(11/14)	0.00000(0/23)	0.05000(1/20)
Bo_TE_80599	0.15556(7/45)	0.00000(0/18)	0.00000(0/23)	0.83333(15/18)
Bo_TE_144845	0.15217(7/46)	0.88235(15/17)	1.00000(22/22)	0.38889(7/18)
Bo_TE_184424	0.24390(10/41)	0.55556(10/18)	0.76190(16/21)	0.60000(12/20)
Bo_TE_81805	0.00000(0/46)	0.00000(0/19)	0.52381(11/21)	0.00000(0/20)
Bo_TE_76691	0.16279(7/43)	0.38889(7/18)	1.00000(23/23)	1.00000(19/19)
Bo_TE_92202	0.85000(34/40)	0.73333(11/15)	1.00000(19/19)	0.36842(7/19)
Bo_TE_237352	0.76190(32/42)	0.00000(0/17)	0.00000(0/21)	0.00000(0/18)
Bo_TE_74389	0.93023(40/43)	0.35294(6/17)	0.85000(17/20)	0.89474(17/19)
Bo_TE_95731	0.51515(17/33)	0.53333(8/15)	1.00000(22/22)	0.15000(3/20)
Bo_TE_195087	0.33333(14/42)	0.88889(16/18)	1.00000(22/22)	0.62500(10/16)
Bo_TE_81838	0.43902(18/41)	0.00000(0/19)	0.42105(8/19)	0.52632(10/19)
Bo_TE_15495	0.17391(8/46)	0.93750(15/16)	1.00000(22/22)	1.00000(20/20)
Bo_TE_34825	0.48780(20/41)	0.94737(18/19)	0.35000(7/20)	0.20000(4/20)
Bo_TE_15315	0.45000(18/40)	1.00000(17/17)	0.21739(5/23)	0.40000(8/20)
Bo_TE_200757	0.84615(33/39)	0.93333(14/15)	0.50000(2/4)	0.22222(4/18)
Bo_TE_111885	0.30952(13/42)	0.94737(18/19)	0.23810(5/21)	1.00000(18/18)
Bo_TE_191807	0.35000(14/40)	0.62500(10/16)	0.95652(22/23)	0.38889(7/18)
Bo_TE_36999	0.63415(26/41)	0.20000(3/15)	0.04545(1/22)	0.42105(8/19)
Bo_TE_200326	0.41860(18/43)	0.37500(6/16)	0.90909(20/22)	0.78947(15/19)
Bo_TE_165099	0.00000(0/44)	0.29412(5/17)	0.85714(18/21)	0.80000(16/20)
Bo_TE_79149	0.54762(23/42)	0.77778(14/18)	0.33333(7/21)	0.26316(5/19)
Bo_TE_55292	0.10870(5/46)	0.57895(11/19)	0.95238(20/21)	0.84211(16/19)
Bo_TE_102680	0.26190(11/42)	0.05556(1/18)	0.36364(8/22)	0.73684(14/19)
Bo_TE_215977	0.73171(30/41)	0.62500(10/16)	0.60000(12/20)	0.15000(3/20)
Bo_TE_89914	0.04651(2/43)	0.70588(12/17)	0.55000(11/20)	0.84211(16/19)

Bo_TE_163814	0.90476(38/42)	0.35294(6/17)	0.63158(12/19)	0.21053(4/19)
Bo_TE_113491	0.15385(6/39)	0.11765(2/17)	0.61905(13/21)	0.10000(2/20)
Bo_TE_11878	1.00000(39/39)	0.64706(11/17)	0.53333(8/15)	0.11765(2/17)
Bo_TE_37496	0.97778(44/45)	0.94737(18/19)	0.39130(9/23)	0.15789(3/19)
Bo_TE_199078	0.85714(36/42)	0.23529(4/17)	0.22727(5/22)	0.70588(12/17)
Bo_TE_169831	0.35714(15/42)	0.93750(15/16)	1.00000(22/22)	0.90000(18/20)
Bo_TE_230837	0.41026(16/39)	0.37500(6/16)	1.00000(23/23)	1.00000(20/20)
Bo_TE_194282	0.00000(0/46)	0.05556(1/18)	0.33333(7/21)	0.60000(12/20)
Bo_TE_156698	0.48780(20/41)	0.38889(7/18)	0.95652(22/23)	0.80000(16/20)
Bo_TE_50385	0.02222(1/45)	0.42105(8/19)	0.14286(3/21)	0.80000(16/20)
Bo_TE_69131	1.00000(45/45)	1.00000(18/18)	0.42857(9/21)	0.95000(19/20)
Bo_TE_141958	0.00000(0/44)	0.33333(6/18)	0.57143(12/21)	0.50000(10/20)
Bo_TE_34643	1.00000(43/43)	0.72222(13/18)	0.40000(8/20)	0.16667(3/18)
Bo_TE_165039	0.02174(1/46)	0.05882(1/17)	0.36364(8/22)	0.55000(11/20)
Bo_TE_65089	1.00000(45/45)	1.00000(19/19)	0.40000(8/20)	1.00000(20/20)
Bo_TE_90672	0.88889(40/45)	0.05556(1/18)	0.00000(0/22)	0.00000(0/20)
Bo_TE_103715	0.76923(30/39)	0.17647(3/17)	0.76190(16/21)	0.35294(6/17)
Bo_TE_236437	0.36585(15/41)	0.70588(12/17)	0.42105(8/19)	0.00000(0/20)
Bo_TE_220078	0.21053(8/38)	0.78947(15/19)	0.15000(3/20)	0.29412(5/17)
Bo_TE_58533	0.90698(39/43)	0.23529(4/17)	0.80952(17/21)	0.75000(15/20)
Bo_TE_117794	0.56410(22/39)	0.75000(12/16)	0.00000(0/23)	0.05000(1/20)
Bo_TE_18772	0.41860(18/43)	0.22222(4/18)	0.05000(1/20)	0.60000(12/20)
Bo_TE_78641	0.85714(36/42)	0.83333(15/18)	0.39130(9/23)	0.10000(2/20)
Bo_TE_178794	0.53488(23/43)	0.83333(15/18)	0.34783(8/23)	0.05556(1/18)
Bo_TE_122867	0.00000(0/45)	0.55556(10/18)	0.00000(0/22)	0.10000(2/20)
Bo_TE_110956	0.56098(23/41)	0.83333(15/18)	0.33333(7/21)	0.70000(14/20)
Bo_TE_9216	0.78571(33/42)	0.35294(6/17)	0.80952(17/21)	1.00000(20/20)
Bo_TE_136458	0.02174(1/46)	0.83333(15/18)	0.66667(14/21)	0.36842(7/19)
Bo_TE_230793	0.50000(21/42)	0.50000(9/18)	0.33333(7/21)	0.00000(0/20)
Bo_TE_134869	0.11364(5/44)	0.62500(10/16)	0.09524(2/21)	0.05000(1/20)
Bo_TE_73156	0.13953(6/43)	0.68750(11/16)	0.30000(6/20)	0.60000(12/20)
Bo_TE_116417	0.00000(0/45)	0.00000(0/19)	0.76190(16/21)	0.47368(9/19)
Bo_TE_136200	0.12195(5/41)	0.18750(3/16)	0.04762(1/21)	0.73684(14/19)
Bo_TE_224852	0.52381(22/42)	0.11765(2/17)	0.20000(4/20)	0.85000(17/20)
Bo_TE_234135	0.22727(10/44)	0.50000(8/16)	0.00000(0/21)	0.85000(17/20)
Bo_TE_141390	0.82222(37/45)	0.06250(1/16)	0.04762(1/21)	0.17647(3/17)
Bo_TE_43435	0.34884(15/43)	0.41176(7/17)	1.00000(21/21)	0.55556(10/18)
Bo_TE_198691	0.93939(31/33)	0.75000(12/16)	0.52632(10/19)	0.06250(1/16)
Bo_TE_143477	0.00000(0/42)	0.00000(0/17)	0.59091(13/22)	0.00000(0/20)
Bo_TE_62927	0.04444(2/45)	0.66667(12/18)	0.42857(9/21)	0.00000(0/20)
Bo_TE_70039	0.64286(27/42)	0.94444(17/18)	0.19048(4/21)	0.65000(13/20)
Bo_TE_10266	0.65909(29/44)	0.00000(0/17)	0.00000(0/21)	0.17647(3/17)
Bo_TE_235153	0.00000(0/46)	0.00000(0/17)	0.52381(11/21)	0.00000(0/20)
Bo_TE_125277	0.00000(0/44)	0.50000(9/18)	0.42857(9/21)	0.05000(1/20)
Bo_TE_63105	0.00000(0/45)	0.00000(0/18)	0.55556(10/18)	0.52632(10/19)
Bo_TE_20945	0.11905(5/42)	0.16667(3/18)	1.00000(19/19)	0.94444(17/18)
Bo_TE_185338	0.02222(1/45)	0.55556(10/18)	0.38095(8/21)	0.15000(3/20)
Bo_TE_81646	0.02174(1/46)	0.88235(15/17)	0.00000(0/22)	0.00000(0/20)
Bo_TE_140007	0.00000(0/45)	0.11765(2/17)	0.50000(10/20)	0.05000(1/20)
Bo_TE_195793	0.79487(31/39)	0.20000(3/15)	0.04348(1/23)	0.10526(2/19)
Bo_TE_26350	0.80000(32/40)	0.16667(3/18)	0.44444(8/18)	0.89474(17/19)
Bo_TE_240158	0.02174(1/46)	0.52632(10/19)	0.04348(1/23)	0.05000(1/20)
Bo_TE_130475	0.33333(13/39)	0.50000(9/18)	0.00000(0/23)	0.00000(0/20)
Bo_TE_159691	0.44737(17/38)	1.00000(18/18)	0.95455(21/22)	0.93750(15/16)
Bo_TE_92880	0.40000(18/45)	0.55556(10/18)	0.00000(0/19)	0.10000(2/20)
Bo_TE_58422	0.06977(3/43)	0.00000(0/19)	0.54545(12/22)	0.00000(0/20)

Bo_TE_112461	0.70000(28/40)	0.75000(12/16)	0.10526(2/19)	0.90000(18/20)
Bo_TE_134249	0.43590(17/39)	0.94737(18/19)	0.55000(11/20)	0.25000(5/20)
Bo_TE_168159	0.32558(14/43)	0.10526(2/19)	0.47368(9/19)	0.63158(12/19)
Bo_TE_84203	0.17073(7/41)	0.22222(4/18)	0.63636(14/22)	1.00000(20/20)
Bo_TE_145870	0.82222(37/45)	0.72222(13/18)	0.09091(2/22)	0.61111(11/18)
Bo_TE_105918	0.41463(17/41)	0.83333(15/18)	1.00000(23/23)	0.90000(18/20)
Bo_TE_76798	0.65854(27/41)	0.11111(2/18)	0.71429(15/21)	0.47368(9/19)
Bo_TE_18281	1.00000(44/44)	0.93750(15/16)	0.95455(21/22)	0.11111(2/18)
Bo_TE_105006	0.66667(30/45)	0.33333(6/18)	0.40000(8/20)	0.85000(17/20)
Bo_TE_43063	0.84091(37/44)	0.61111(11/18)	0.85714(18/21)	0.05556(1/18)
Bo_TE_71219	0.42857(18/42)	0.88889(16/18)	0.28571(6/21)	0.57895(11/19)
Bo_TE_39629	0.02326(1/43)	0.00000(0/17)	0.55000(11/20)	0.45000(9/20)
Bo_TE_140477	0.44186(19/43)	0.76471(13/17)	0.35000(7/20)	1.00000(19/19)
Bo_TE_102657	0.21429(9/42)	0.05882(1/17)	0.36364(8/22)	0.68421(13/19)
Bo_TE_123895	0.08696(4/46)	0.88235(15/17)	0.36842(7/19)	0.73333(11/15)
Bo_TE_208467	0.76744(33/43)	0.35294(6/17)	0.86364(19/22)	0.90000(18/20)
Bo_TE_112741	0.79070(34/43)	0.17647(3/17)	0.00000(0/22)	0.00000(0/20)
Bo_TE_210887	0.41463(17/41)	0.83333(15/18)	0.30000(6/20)	0.10526(2/19)
Bo_TE_49148	0.02273(1/44)	0.00000(0/18)	0.70000(14/20)	0.00000(0/19)
Bo_TE_208032	0.93023(40/43)	0.47059(8/17)	1.00000(22/22)	1.00000(18/18)
Bo_TE_226434	0.37838(14/37)	0.00000(0/17)	0.36364(8/22)	0.52632(10/19)
Bo_TE_71375	0.11628(5/43)	0.94444(17/18)	0.75000(15/20)	0.57895(11/19)
Bo_TE_8760	0.90909(40/44)	0.00000(0/15)	1.00000(23/23)	0.47368(9/19)
Bo_TE_96385	0.00000(0/45)	0.00000(0/18)	0.30000(6/20)	0.84211(16/19)
Bo_TE_155491	0.70732(29/41)	0.41176(7/17)	0.15000(3/20)	0.73333(11/15)
Bo_TE_196385	0.95455(42/44)	0.47059(8/17)	1.00000(23/23)	1.00000(19/19)
Bo_TE_14107	0.44186(19/43)	0.00000(0/13)	0.95652(22/23)	0.22222(4/18)
Bo_TE_66950	0.02273(1/44)	0.62500(10/16)	0.45455(10/22)	0.73684(14/19)
Bo_TE_179641	0.71111(32/45)	0.26667(4/15)	0.26316(5/19)	0.00000(0/20)
Bo_TE_39501	0.68182(30/44)	0.16667(3/18)	0.28571(6/21)	0.00000(0/19)
Bo_TE_125060	0.33333(14/42)	0.06250(1/16)	0.47368(9/19)	0.78947(15/19)
Bo_TE_122848	0.52500(21/40)	0.66667(12/18)	0.04762(1/21)	0.05000(1/20)
Bo_TE_236758	0.97674(42/43)	0.82353(14/17)	0.27273(6/22)	0.20000(4/20)
Bo_TE_17598	0.53659(22/41)	0.06250(1/16)	0.00000(0/20)	0.00000(0/16)
Bo_TE_18470	0.72727(32/44)	0.22222(4/18)	0.19048(4/21)	0.00000(0/20)
Bo_TE_177620	1.00000(44/44)	1.00000(18/18)	0.57143(12/21)	0.50000(9/18)
Bo_TE_184447	0.54054(20/37)	0.00000(0/19)	0.09524(2/21)	0.00000(0/16)
Bo_TE_4735	0.00000(0/44)	0.68750(11/16)	0.42857(9/21)	0.05000(1/20)
Bo_TE_21179	0.63415(26/41)	0.35294(6/17)	0.00000(0/21)	0.33333(6/18)
Bo_TE_51036	0.42857(18/42)	0.47059(8/17)	1.00000(21/21)	1.00000(19/19)
Bo_TE_64913	0.38095(16/42)	0.52941(9/17)	0.95455(21/22)	0.61111(11/18)
Bo_TE_133072	0.65116(28/43)	0.77778(14/18)	0.19048(4/21)	0.50000(10/20)
Bo_TE_110863	0.93182(41/44)	0.93750(15/16)	0.82609(19/23)	0.31579(6/19)
Bo_TE_25264	0.00000(0/44)	0.55556(10/18)	0.47619(10/21)	0.70000(14/20)
Bo_TE_145008	0.00000(0/40)	0.80000(4/5)	1.00000(18/18)	0.78947(15/19)
Bo_TE_8141	0.00000(0/43)	0.29412(5/17)	0.54545(12/22)	0.85000(17/20)
Bo_TE_108008	0.97674(42/43)	0.41176(7/17)	0.76190(16/21)	0.77778(14/18)
Bo_TE_174203	0.38636(17/44)	0.75000(12/16)	0.71429(15/21)	1.00000(20/20)
Bo_TE_198491	0.88372(38/43)	0.64706(11/17)	0.63636(14/22)	0.00000(0/17)
Bo_TE_5848	0.70455(31/44)	0.35294(6/17)	0.21739(5/23)	0.00000(0/18)
Bo_TE_61959	0.67500(27/40)	0.56250(9/16)	0.47619(10/21)	1.00000(20/20)
Bo_TE_69137	1.00000(46/46)	1.00000(18/18)	0.42857(9/21)	0.95000(19/20)
Bo_TE_45630	0.40476(17/42)	0.93333(14/15)	0.78947(15/19)	1.00000(16/16)
Bo_TE_61980	0.63636(28/44)	0.56250(9/16)	0.47619(10/21)	1.00000(20/20)
Bo_TE_133278	0.79070(34/43)	0.17647(3/17)	0.04545(1/22)	0.78947(15/19)
Bo_TE_34778	0.23256(10/43)	0.00000(0/19)	0.42857(9/21)	0.50000(9/18)

Bo_TE_65876	0.73333(33/45)	0.94737(18/19)	1.00000(22/22)	0.47368(9/19)
Bo_TE_180086	0.42500(17/40)	0.53333(8/15)	0.80952(17/21)	0.00000(0/19)
Bo_TE_10782	0.93333(42/45)	0.31250(5/16)	1.00000(23/23)	0.75000(15/20)
Bo_TE_122444	0.18605(8/43)	0.05556(1/18)	0.72727(16/22)	0.36842(7/19)
Bo_TE_121484	0.35714(15/42)	0.00000(0/18)	0.33333(7/21)	0.89474(17/19)
Bo_TE_137234	0.16279(7/43)	0.37500(6/16)	0.47619(10/21)	1.00000(19/19)
Bo_TE_70482	0.11905(5/42)	0.05882(1/17)	0.59091(13/22)	0.73684(14/19)
Bo_TE_212059	0.13636(6/44)	0.00000(0/18)	0.75000(15/20)	0.05000(1/20)
Bo_TE_88887	0.00000(0/45)	0.00000(0/18)	0.18182(4/22)	0.70000(14/20)
Bo_TE_101028	0.20930(9/43)	0.69231(9/13)	0.65000(13/20)	0.94444(17/18)
Bo_TE_207272	0.36364(16/44)	0.66667(12/18)	0.63158(12/19)	0.15000(3/20)
Bo_TE_37109	0.35714(15/42)	0.87500(14/16)	0.04762(1/21)	0.05000(1/20)
Bo_TE_90448	0.73810(31/42)	0.64706(11/17)	0.04545(1/22)	0.21053(4/19)
Bo_TE_160796	0.31111(14/45)	0.47368(9/19)	0.72727(16/22)	0.05263(1/19)
Bo_TE_158128	0.14634(6/41)	0.64706(11/17)	0.57143(12/21)	0.77778(14/18)
Bo_TE_111980	0.46341(19/41)	0.16667(3/18)	0.72727(16/22)	0.16667(3/18)
Bo_TE_93454	0.18605(8/43)	0.11111(2/18)	1.00000(22/22)	0.95000(19/20)
Bo_TE_3266	0.00000(0/44)	0.00000(0/17)	0.35000(7/20)	0.62500(10/16)
Bo_TE_158716	0.22727(10/44)	0.06250(1/16)	0.57895(11/19)	0.10526(2/19)
Bo_TE_239513	0.72500(29/40)	0.93333(14/15)	0.90476(19/21)	0.36842(7/19)
Bo_TE_128603	0.59091(26/44)	0.05556(1/18)	0.00000(0/23)	0.05556(1/18)
Bo_TE_12825	1.00000(42/42)	0.52941(9/17)	0.45000(9/20)	0.42105(8/19)
Bo_TE_13250	0.34211(13/38)	0.22222(4/18)	0.47059(8/17)	1.00000(20/20)
Bo_TE_207011	0.62500(25/40)	0.43750(7/16)	0.00000(0/23)	0.50000(9/18)
Bo_TE_225959	0.88636(39/44)	0.38889(7/18)	1.00000(21/21)	1.00000(20/20)
Bo_TE_145219	0.17949(7/39)	0.58824(10/17)	0.00000(0/21)	0.55000(11/20)
Bo_TE_111061	0.63636(28/44)	0.29412(5/17)	1.00000(22/22)	0.65000(13/20)
Bo_TE_101131	0.00000(0/46)	0.15789(3/19)	0.59091(13/22)	0.05000(1/20)
Bo_TE_29175	0.78571(33/42)	0.26316(5/19)	0.19048(4/21)	0.15000(3/20)
Bo_TE_230121	0.30435(14/46)	0.18750(3/16)	0.80952(17/21)	0.84211(16/19)
Bo_TE_37422	0.35000(14/40)	0.86667(13/15)	0.85000(17/20)	1.00000(19/19)
Bo_TE_211754	0.03704(1/27)	0.00000(0/18)	0.73684(14/19)	0.15000(3/20)
Bo_TE_136880	0.00000(0/42)	0.68750(11/16)	0.00000(0/23)	0.00000(0/20)
Bo_TE_154831	0.73810(31/42)	0.17647(3/17)	0.77273(17/22)	0.95000(19/20)
Bo_TE_53398	0.42500(17/40)	0.64706(11/17)	0.66667(14/21)	1.00000(19/19)
Bo_TE_207154	0.96296(26/27)	0.50000(8/16)	0.87500(14/16)	1.00000(17/17)
Bo_TE_32944	0.43902(18/41)	0.50000(9/18)	1.00000(23/23)	0.95000(19/20)
Bo_TE_11862	0.97561(40/41)	0.64706(11/17)	0.42857(9/21)	0.15789(3/19)
Bo_TE_2313	0.04545(2/44)	0.20000(3/15)	0.35000(7/20)	0.75000(15/20)
Bo_TE_155115	0.08696(4/46)	0.58824(10/17)	0.00000(0/23)	0.26316(5/19)
Bo_TE_90762	0.84444(38/45)	1.00000(18/18)	0.61905(13/21)	0.15789(3/19)
Bo_TE_126286	0.13333(6/45)	0.76471(13/17)	0.23810(5/21)	0.05000(1/20)
Bo_TE_80693	0.15556(7/45)	0.21053(4/19)	0.35000(7/20)	0.89474(17/19)
Bo_TE_88101	0.06667(3/45)	0.55556(10/18)	0.00000(0/23)	0.05000(1/20)
Bo_TE_175279	0.43902(18/41)	0.94444(17/18)	0.95000(19/20)	0.80000(16/20)
Bo_TE_97917	0.32500(13/40)	0.94444(17/18)	0.31818(7/22)	0.66667(12/18)
Bo_TE_88986	0.18182(8/44)	0.58824(10/17)	0.33333(7/21)	0.89474(17/19)
Bo_TE_104409	0.02222(1/45)	0.00000(0/18)	0.21739(5/23)	0.52632(10/19)
Bo_TE_32656	0.71429(30/42)	0.00000(0/18)	0.00000(0/22)	0.27778(5/18)
Bo_TE_55899	0.02703(1/37)	0.50000(8/16)	0.85714(18/21)	0.15000(3/20)
Bo_TE_145420	0.40476(17/42)	0.70588(12/17)	0.60000(12/20)	1.00000(18/18)
Bo_TE_84305	0.17949(7/39)	0.05882(1/17)	0.47826(11/23)	0.61111(11/18)
Bo_TE_156623	0.00000(0/44)	0.88235(15/17)	0.04762(1/21)	0.00000(0/17)
Bo_TE_41690	0.59091(26/44)	0.82353(14/17)	0.40909(9/22)	0.25000(5/20)
Bo_TE_129355	0.20000(8/40)	0.14286(2/14)	1.00000(23/23)	0.15789(3/19)
Bo_TE_235479	0.44186(19/43)	0.40000(6/15)	1.00000(23/23)	0.63158(12/19)

Bo_TE_146463	0.78571(33/42)	0.58824(10/17)	0.28571(6/21)	0.63158(12/19)
Bo_TE_104132	0.00000(0/46)	0.56250(9/16)	0.04348(1/23)	0.00000(0/19)
Bo_TE_116448	0.59091(26/44)	0.06250(1/16)	0.28571(6/21)	0.70000(14/20)
Bo_TE_25045	0.07143(3/42)	0.35294(6/17)	0.08696(2/23)	0.85000(17/20)
Bo_TE_80720	0.34091(15/44)	0.21053(4/19)	0.91304(21/23)	0.94444(17/18)
Bo_TE_62954	0.19512(8/41)	0.62500(10/16)	0.45000(9/20)	0.00000(0/20)
Bo_TE_8017	0.95349(41/43)	1.00000(17/17)	0.50000(9/18)	0.42105(8/19)
Bo_TE_58429	0.04545(2/44)	0.55556(10/18)	0.27273(6/22)	0.73684(14/19)
Bo_TE_232160	0.30435(14/46)	0.56250(9/16)	0.05000(1/20)	0.00000(0/20)
Bo_TE_55717	0.04348(2/46)	0.00000(0/18)	0.08696(2/23)	0.88889(16/18)
Bo_TE_66990	0.71429(30/42)	0.64706(11/17)	0.00000(0/22)	0.05263(1/19)
Bo_TE_6038	0.74419(32/43)	0.05556(1/18)	0.26316(5/19)	0.44444(8/18)
Bo_TE_132453	0.09302(4/43)	0.00000(0/17)	0.77273(17/22)	0.50000(9/18)
Bo_TE_83985	0.11364(5/44)	0.00000(0/18)	0.57143(12/21)	0.80000(16/20)
Bo_TE_190881	0.63415(26/41)	0.00000(0/17)	0.04545(1/22)	0.00000(0/20)
Bo_TE_226738	0.97826(45/46)	1.00000(17/17)	0.23810(5/21)	0.95000(19/20)
Bo_TE_227655	0.88636(39/44)	0.88235(15/17)	0.15000(3/20)	0.05000(1/20)
Bo_TE_126160	0.40000(16/40)	0.94444(17/18)	0.35000(7/20)	0.10526(2/19)
Bo_TE_76620	0.00000(0/44)	0.00000(0/17)	0.52381(11/21)	0.00000(0/20)
Bo_TE_195589	0.09302(4/43)	0.23529(4/17)	0.26316(5/19)	0.66667(12/18)
Bo_TE_123785	0.79310(23/29)	0.12500(2/16)	0.65000(13/20)	0.70588(12/17)
Bo_TE_24916	0.65789(25/38)	0.00000(0/18)	0.00000(0/21)	0.00000(0/20)
Bo_TE_178153	0.48485(16/33)	0.17647(3/17)	1.00000(19/19)	0.33333(2/6)
Bo_TE_40779	0.73171(30/41)	0.73333(11/15)	0.21053(4/19)	0.55556(10/18)
Bo_TE_162875	0.02273(1/44)	0.11111(2/18)	0.33333(7/21)	0.65000(13/20)
Bo_TE_186389	0.06667(3/45)	0.72222(13/18)	0.56250(9/16)	0.05556(1/18)
Bo_TE_220491	0.02632(1/38)	0.82353(14/17)	0.52632(10/19)	0.15000(3/20)
Bo_TE_119250	0.14286(6/42)	0.05882(1/17)	0.50000(10/20)	0.00000(0/20)
Bo_TE_153613	0.02326(1/43)	0.50000(9/18)	0.45000(9/20)	0.82353(14/17)
Bo_TE_141089	0.00000(0/45)	0.22222(4/18)	0.75000(15/20)	0.00000(0/20)
Bo_TE_168552	0.93939(31/33)	0.05882(1/17)	0.71429(15/21)	0.80000(16/20)
Bo_TE_25639	0.51220(21/41)	0.17647(3/17)	0.09091(2/22)	0.75000(15/20)
Bo_TE_95278	0.19512(8/41)	0.00000(0/18)	0.50000(11/22)	0.05556(1/18)
Bo_TE_78467	0.41026(16/39)	0.17647(3/17)	0.68182(15/22)	0.72222(13/18)
Bo_TE_84453	0.02174(1/46)	0.77778(14/18)	0.04348(1/23)	0.26316(5/19)
Bo_TE_203580	0.30000(12/40)	0.12500(2/16)	0.60000(12/20)	0.90000(18/20)
Bo_TE_224276	0.29545(13/44)	0.05556(1/18)	0.60000(12/20)	0.05556(1/18)
Bo_TE_16749	0.88636(39/44)	0.52941(9/17)	0.20000(4/20)	0.20000(4/20)
Bo_TE_163610	0.06522(3/46)	0.11765(2/17)	0.18182(4/22)	0.70588(12/17)
Bo_TE_137571	0.50000(20/40)	0.93750(15/16)	0.95652(22/23)	1.00000(20/20)
Bo_TE_148473	0.00000(0/46)	0.82353(14/17)	0.00000(0/23)	0.31579(6/19)
Bo_TE_239643	0.16279(7/43)	0.58824(10/17)	1.00000(22/22)	1.00000(16/16)
Bo_TE_61936	0.93478(43/46)	0.84211(16/19)	0.47619(10/21)	1.00000(20/20)
Bo_TE_50052	1.00000(45/45)	0.76471(13/17)	1.00000(23/23)	0.35294(6/17)
Bo_TE_239042	0.02381(1/42)	0.05556(1/18)	0.50000(10/20)	0.00000(0/20)
Bo_TE_132230	0.27500(11/40)	0.62500(10/16)	0.95455(21/22)	1.00000(20/20)
Bo_TE_24180	0.58537(24/41)	0.27778(5/18)	0.36842(7/19)	0.90000(18/20)
Bo_TE_85716	0.04762(2/42)	0.52632(10/19)	0.20000(4/20)	0.55556(10/18)
Bo_TE_205223	0.00000(0/45)	0.64706(11/17)	0.00000(0/23)	0.00000(0/20)
Bo_TE_159555	0.53846(21/39)	0.83333(15/18)	0.70000(7/10)	0.27778(5/18)
Bo_TE_62875	0.97778(44/45)	0.33333(6/18)	0.52381(11/21)	1.00000(19/19)
Bo_TE_107969	0.95652(44/46)	0.18750(3/16)	0.40000(8/20)	0.21053(4/19)
Bo_TE_152413	0.77778(35/45)	0.05263(1/19)	0.00000(0/22)	0.05000(1/20)
Bo_TE_69270	0.82500(33/40)	0.18750(3/16)	0.36364(8/22)	0.68421(13/19)
Bo_TE_4783	0.76190(32/42)	0.50000(9/18)	0.90000(18/20)	0.25000(4/16)
Bo_TE_128355	0.33333(13/39)	0.66667(12/18)	0.33333(6/18)	1.00000(18/18)

Bo_TE_236508	0.00000(0/45)	0.00000(0/19)	0.19048(4/21)	0.61111(11/18)
Bo_TE_3841	0.16279(7/43)	0.75000(12/16)	0.40000(8/20)	1.00000(20/20)
Bo_TE_102575	0.54545(24/44)	0.05882(1/17)	0.59091(13/22)	0.16667(3/18)
Bo_TE_133839	0.93333(42/45)	0.50000(9/18)	0.81818(18/22)	0.31579(6/19)
Bo_TE_85431	0.27273(12/44)	0.52941(9/17)	1.00000(21/21)	0.77778(7/9)
Bo_TE_109562	0.18919(7/37)	0.11765(2/17)	0.65000(13/20)	0.72222(13/18)
Bo_TE_8049	1.00000(44/44)	0.94444(17/18)	0.45000(9/20)	0.55556(10/18)
Bo_TE_104636	0.18182(8/44)	0.23529(4/17)	0.80952(17/21)	0.00000(0/20)
Bo_TE_224488	0.06667(3/45)	0.68421(13/19)	0.00000(0/21)	0.00000(0/20)
Bo_TE_10319	0.42857(15/35)	0.80000(12/15)	0.04545(1/22)	0.68421(13/19)
Bo_TE_184939	0.00000(0/42)	0.52941(9/17)	0.21739(5/23)	0.05000(1/20)
Bo_TE_22734	0.88889(40/45)	0.33333(6/18)	1.00000(22/22)	0.95000(19/20)
Bo_TE_128774	0.24390(10/41)	0.70588(12/17)	0.59091(13/22)	0.00000(0/20)
Bo_TE_43592	0.82927(34/41)	0.61111(11/18)	0.00000(0/21)	0.44444(8/18)
Bo_TE_219992	0.00000(0/38)	0.00000(0/10)	0.61905(13/21)	0.26316(5/19)
Bo_TE_16948	0.73810(31/42)	0.00000(0/16)	0.04545(1/22)	0.31579(6/19)
Bo_TE_52343	0.02222(1/45)	0.72222(13/18)	0.28571(6/21)	0.23529(4/17)
Bo_TE_102724	0.46512(20/43)	0.05556(1/18)	0.00000(0/23)	0.66667(12/18)
Bo_TE_33614	0.29268(12/41)	0.00000(0/17)	0.27273(6/22)	0.77778(14/18)
Bo_TE_162162	0.57500(23/40)	0.11765(2/17)	0.33333(6/18)	0.82353(14/17)
Bo_TE_186369	0.06667(3/45)	0.77778(14/18)	0.63158(12/19)	0.15789(3/19)
Bo_TE_169465	0.64286(27/42)	0.22222(4/18)	0.00000(0/23)	0.00000(0/19)
Bo_TE_15342	0.60465(26/43)	0.17647(3/17)	0.75000(15/20)	0.55556(10/18)
Bo_TE_156610	0.27273(12/44)	1.00000(18/18)	0.90000(18/20)	0.89474(17/19)
Bo_TE_99650	0.72093(31/43)	0.05556(1/18)	0.00000(0/23)	0.00000(0/20)
Bo_TE_111607	0.82609(38/46)	0.77778(14/18)	0.28571(6/21)	0.89474(17/19)
Bo_TE_58012	0.35897(14/39)	0.50000(8/16)	0.00000(0/22)	0.00000(0/20)
Bo_TE_142639	0.09756(4/41)	0.00000(0/18)	0.57143(12/21)	0.26316(5/19)
Bo_TE_70367	0.44444(20/45)	0.11765(2/17)	0.18182(4/22)	0.73684(14/19)
Bo_TE_205146	0.00000(0/45)	0.00000(0/18)	0.42857(9/21)	0.85000(17/20)
Bo_TE_181780	0.00000(0/46)	0.00000(0/19)	0.50000(10/20)	0.05000(1/20)
Bo_TE_147943	0.24390(10/41)	0.35294(6/17)	0.95455(21/22)	0.70000(14/20)
Bo_TE_101122	0.00000(0/43)	0.00000(0/18)	0.09091(2/22)	0.50000(9/18)
Bo_TE_178677	0.32500(13/40)	0.14286(2/14)	0.42857(9/21)	0.94737(18/19)
Bo_TE_214884	1.00000(46/46)	0.88889(16/18)	0.15000(3/20)	0.10526(2/19)
Bo_TE_131810	0.93478(43/46)	0.77778(14/18)	0.36364(8/22)	0.42105(8/19)
Bo_TE_122048	0.57500(23/40)	0.00000(0/2)	1.00000(23/23)	0.35294(6/17)
Bo_TE_28090	0.19048(8/42)	0.88889(16/18)	0.54545(12/22)	0.90000(18/20)
Bo_TE_26701	0.00000(0/45)	0.00000(0/19)	0.00000(0/22)	0.68421(13/19)
Bo_TE_84212	0.40000(16/40)	0.58824(10/17)	0.61905(13/21)	1.00000(20/20)
Bo_TE_91674	0.02174(1/46)	0.50000(8/16)	0.30000(6/20)	0.80000(16/20)
Bo_TE_58504	0.65909(29/44)	0.35294(6/17)	0.00000(0/21)	0.05556(1/18)
Bo_TE_80708	0.64103(25/39)	0.75000(12/16)	0.05263(1/19)	0.05556(1/18)
Bo_TE_136208	0.54762(23/42)	0.21053(4/19)	0.63158(12/19)	0.05000(1/20)
Bo_TE_113594	0.61905(26/42)	0.43750(7/16)	1.00000(22/22)	1.00000(18/18)
Bo_TE_209295	0.63415(26/41)	0.16667(3/18)	1.00000(23/23)	0.90000(18/20)
Bo_TE_113618	0.37838(14/37)	0.11111(2/18)	0.45455(10/22)	0.75000(15/20)
Bo_TE_82158	0.72500(29/40)	0.60000(9/15)	0.38095(8/21)	0.00000(0/20)
Bo_TE_75992	0.00000(0/45)	0.05556(1/18)	0.85000(17/20)	0.47368(9/19)
Bo_TE_119666	0.00000(0/44)	0.00000(0/17)	0.57143(12/21)	0.00000(0/19)
Bo_TE_27400	0.89474(34/38)	0.80000(12/15)	0.35714(5/14)	0.05556(1/18)
Bo_TE_68229	0.00000(0/45)	0.00000(0/19)	0.54545(12/22)	0.75000(15/20)
Bo_TE_70454	0.83721(36/43)	0.83333(15/18)	0.04762(1/21)	0.22222(4/18)
Bo_TE_223228	0.36364(16/44)	0.37500(6/16)	0.34783(8/23)	0.85000(17/20)
Bo_TE_146413	0.11905(5/42)	0.35294(6/17)	0.63636(14/22)	0.33333(6/18)
Bo_TE_15714	0.42857(18/42)	0.58824(10/17)	0.66667(14/21)	1.00000(20/20)

Bo_TE_38969	0.32558(14/43)	0.00000(0/17)	0.56522(13/23)	0.00000(0/20)
Bo_TE_211458	0.77273(34/44)	0.88235(15/17)	0.00000(0/22)	0.77778(14/18)
Bo_TE_53096	0.86364(38/44)	0.21429(3/14)	0.15000(3/20)	0.22222(4/18)
Bo_TE_199001	0.86364(38/44)	0.33333(6/18)	0.00000(0/22)	0.00000(0/19)
Bo_TE_23845	0.11364(5/44)	0.76471(13/17)	0.13636(3/22)	0.21053(4/19)
Bo_TE_93259	0.47619(20/42)	0.76471(13/17)	0.18182(4/22)	0.88889(16/18)
Bo_TE_193756	0.40000(14/35)	0.61111(11/18)	0.20000(4/20)	0.05882(1/17)
Bo_TE_36139	0.11364(5/44)	0.06250(1/16)	0.85714(18/21)	0.31579(6/19)
Bo_TE_171341	0.51282(20/39)	0.12500(2/16)	0.15000(3/20)	0.70000(14/20)
Bo_TE_124049	0.00000(0/45)	0.00000(0/19)	0.04348(1/23)	0.55000(11/20)
Bo_TE_224580	0.67647(23/34)	0.11111(2/18)	0.71429(15/21)	0.61111(11/18)
Bo_TE_221205	0.18605(8/43)	0.87500(14/16)	0.00000(0/22)	0.05263(1/19)
Bo_TE_68308	0.51111(23/45)	0.23529(4/17)	0.00000(0/21)	0.33333(6/18)
Bo_TE_45611	0.60976(25/41)	0.05556(1/18)	0.22727(5/22)	0.00000(0/20)
Bo_TE_142899	0.28889(13/45)	0.00000(0/19)	0.30000(6/20)	0.65000(13/20)
Bo_TE_20322	0.88372(38/43)	0.23529(4/17)	0.86364(19/22)	0.88235(15/17)
Bo_TE_103088	0.36364(16/44)	0.94118(16/17)	0.55000(11/20)	0.36842(7/19)
Bo_TE_150848	0.05405(2/37)	0.53333(8/15)	0.71429(15/21)	0.25000(4/16)
Bo_TE_88792	0.02439(1/41)	0.00000(0/17)	0.33333(6/18)	0.84211(16/19)
Bo_TE_132342	0.02174(1/46)	0.00000(0/18)	0.55000(11/20)	0.00000(0/20)
Bo_TE_121764	0.86486(32/37)	1.00000(12/12)	0.94444(17/18)	0.20000(4/20)
Bo_TE_124912	0.73333(33/45)	0.50000(8/16)	0.90909(20/22)	0.11765(2/17)
Bo_TE_215392	0.19512(8/41)	0.16667(3/18)	0.26316(5/19)	0.77778(14/18)
Bo_TE_124321	0.17073(7/41)	0.73684(14/19)	0.73913(17/23)	0.82353(14/17)
Bo_TE_169297	0.52381(22/42)	0.00000(0/19)	0.00000(0/22)	0.00000(0/20)
Bo_TE_80235	0.65116(28/43)	0.77778(14/18)	0.08696(2/23)	0.77778(14/18)
Bo_TE_118567	0.90909(40/44)	0.94118(16/17)	1.00000(23/23)	0.45000(9/20)
Bo_TE_61426	0.36585(15/41)	0.87500(14/16)	0.04762(1/21)	0.10526(2/19)
Bo_TE_220034	0.06667(3/45)	0.29412(5/17)	0.61905(13/21)	0.89474(17/19)
Bo_TE_36107	0.87500(35/40)	0.94118(16/17)	0.22727(5/22)	0.68421(13/19)
Bo_TE_37062	0.42500(17/40)	0.25000(2/8)	0.95652(22/23)	0.83333(15/18)
Bo_TE_151376	0.12500(5/40)	0.88889(16/18)	0.15789(3/19)	0.44444(8/18)
Bo_TE_208239	0.27907(12/43)	0.37500(6/16)	0.80952(17/21)	0.90000(18/20)
Bo_TE_25252	0.00000(0/44)	0.05263(1/19)	0.50000(10/20)	0.00000(0/20)
Bo_TE_184257	0.94737(36/38)	1.00000(17/17)	0.78947(15/19)	0.29412(5/17)
Bo_TE_210054	0.93182(41/44)	1.00000(19/19)	0.50000(11/22)	0.95000(19/20)
Bo_TE_210013	0.95238(40/42)	0.41176(7/17)	1.00000(23/23)	1.00000(20/20)
Bo_TE_204461	0.17778(8/45)	0.52941(9/17)	0.42857(9/21)	0.95000(19/20)
Bo_TE_29188	0.20000(8/40)	0.05556(1/18)	0.71429(15/21)	0.05882(1/17)
Bo_TE_149244	0.04545(2/44)	0.47059(8/17)	0.20000(4/20)	0.64706(11/17)
Bo_TE_46140	0.75610(31/41)	0.41176(7/17)	0.00000(0/22)	0.05000(1/20)
Bo_TE_94721	0.73810(31/42)	0.18750(3/16)	0.08696(2/23)	0.66667(12/18)
Bo_TE_33517	0.83721(36/43)	0.00000(0/16)	0.04545(1/22)	0.16667(3/18)
Bo_TE_169870	0.63415(26/41)	0.27778(5/18)	0.00000(0/23)	0.05882(1/17)
Bo_TE_38911	0.97778(44/45)	0.88889(16/18)	0.81818(18/22)	0.42105(8/19)
Bo_TE_48794	0.80000(36/45)	0.77778(14/18)	0.45455(10/22)	0.30000(6/20)
Bo_TE_167768	0.03125(1/32)	0.15789(3/19)	0.65000(13/20)	0.00000(0/20)
Bo_TE_128607	0.65000(26/40)	0.00000(0/17)	0.00000(0/22)	0.05000(1/20)
Bo_TE_204780	0.00000(0/45)	0.23529(4/17)	0.52381(11/21)	0.11111(2/18)
Bo_TE_4639	0.00000(0/45)	0.78571(11/14)	0.66667(14/21)	0.50000(9/18)
Bo_TE_40321	0.25581(11/43)	0.57895(11/19)	0.55000(11/20)	0.00000(0/18)
Bo_TE_194589	0.90244(37/41)	0.11765(2/17)	0.95652(22/23)	0.70000(14/20)
Bo_TE_11047	0.02439(1/41)	0.58824(10/17)	0.13043(3/23)	0.72222(13/18)
Bo_TE_57734	0.05000(2/40)	0.55556(10/18)	0.04348(1/23)	0.10526(2/19)
Bo_TE_50389	0.02222(1/45)	0.47059(8/17)	0.14286(3/21)	0.77778(14/18)
Bo_TE_99906	0.19512(8/41)	0.70588(12/17)	0.04545(1/22)	0.00000(0/19)

Bo_TE_4308	0.88372(38/43)	0.73333(11/15)	0.90909(20/22)	0.15000(3/20)
Bo_TE_101884	0.69767(30/43)	0.11765(2/17)	0.00000(0/23)	0.00000(0/19)
Bo_TE_127324	0.16279(7/43)	0.55556(10/18)	0.00000(0/21)	0.10000(2/20)
Bo_TE_159143	0.54762(23/42)	0.00000(0/16)	0.00000(0/16)	0.00000(0/20)
Bo_TE_197938	0.44444(20/45)	1.00000(17/17)	0.33333(7/21)	0.22222(4/18)
Bo_TE_28774	0.70732(29/41)	0.11765(2/17)	0.00000(0/22)	0.05000(1/20)
Bo_TE_98611	0.12195(5/41)	0.36842(7/19)	0.44444(8/18)	0.84211(16/19)
Bo_TE_189621	0.97561(40/41)	0.88889(16/18)	0.42857(9/21)	1.00000(20/20)
Bo_TE_61712	0.31707(13/41)	0.31250(5/16)	0.66667(14/21)	0.85000(17/20)
Bo_TE_112943	0.71429(30/42)	0.00000(0/18)	0.85714(18/21)	0.00000(0/20)
Bo_TE_109207	0.11905(5/42)	0.00000(0/16)	0.44444(8/18)	0.77778(14/18)
Bo_TE_36874	0.55000(22/40)	0.58824(10/17)	0.18182(4/22)	0.00000(0/20)
Bo_TE_50046	1.00000(44/44)	0.66667(12/18)	0.95652(22/23)	0.42105(8/19)
Bo_TE_169212	0.06977(3/43)	0.82353(14/17)	0.30000(6/20)	0.16667(3/18)
Bo_TE_212014	0.64865(24/37)	0.05556(1/18)	0.12500(1/8)	0.06250(1/16)
Bo_TE_5142	0.72093(31/43)	0.17647(3/17)	0.09524(2/21)	0.11765(2/17)
Bo_TE_216155	0.02273(1/44)	0.29412(5/17)	0.17391(4/23)	0.55556(10/18)
Bo_TE_81495	0.28205(11/39)	0.05556(1/18)	0.04545(1/22)	0.55000(11/20)
Bo_TE_149608	0.76190(32/42)	0.94444(17/18)	0.42857(9/21)	0.47368(9/19)
Bo_TE_18333	0.59524(25/42)	0.66667(10/15)	0.18182(4/22)	0.85000(17/20)
Bo_TE_177997	0.04545(2/44)	0.75000(12/16)	0.80952(17/21)	0.89474(17/19)
Bo_TE_94470	1.00000(45/45)	0.82353(14/17)	0.57895(11/19)	0.47059(8/17)
Bo_TE_126158	0.82500(33/40)	0.44444(8/18)	0.75000(15/20)	1.00000(16/16)
Bo_TE_115547	0.72727(32/44)	0.94118(16/17)	0.86364(19/22)	0.33333(6/18)
Bo_TE_155128	0.06818(3/44)	0.11111(2/18)	0.23810(5/21)	0.70000(14/20)
Bo_TE_235476	0.80488(33/41)	0.16667(3/18)	0.00000(0/23)	0.00000(0/13)
Bo_TE_165325	0.42857(18/42)	1.00000(17/17)	0.85714(18/21)	0.94444(17/18)
Bo_TE_16877	0.32432(12/37)	0.55556(10/18)	0.00000(0/22)	0.00000(0/19)
Bo_TE_201990	0.87500(35/40)	0.55556(10/18)	0.91304(21/23)	0.36842(7/19)
Bo_TE_28710	0.27778(10/36)	0.81250(13/16)	0.28571(6/21)	0.41176(7/17)
Bo_TE_55804	0.04878(2/41)	0.13333(2/15)	0.81818(18/22)	0.15000(3/20)
Bo_TE_214241	0.00000(0/46)	0.64706(11/17)	0.04762(1/21)	0.05000(1/20)
Bo_TE_144224	0.95122(39/41)	0.23529(4/17)	0.86957(20/23)	1.00000(20/20)
Bo_TE_58667	0.71111(32/45)	0.86667(13/15)	0.13043(3/23)	0.05263(1/19)
Bo_TE_197344	0.00000(0/45)	0.11111(2/18)	0.00000(0/23)	0.55556(10/18)
Bo_TE_22435	0.37209(16/43)	0.93750(15/16)	1.00000(21/21)	0.61111(11/18)
Bo_TE_151713	0.61364(27/44)	0.94118(16/17)	0.66667(14/21)	0.38889(7/18)
Bo_TE_158939	0.42857(18/42)	0.46667(7/15)	1.00000(21/21)	0.94737(18/19)
Bo_TE_223707	0.86842(33/38)	0.93750(15/16)	0.38095(8/21)	1.00000(19/19)
Bo_TE_11781	0.59524(25/42)	0.38889(7/18)	0.00000(0/21)	0.00000(0/19)
Bo_TE_60878	0.68293(28/41)	0.28571(4/14)	0.05263(1/19)	0.46667(7/15)
Bo_TE_123325	0.00000(0/44)	0.05556(1/18)	0.80000(16/20)	0.27778(5/18)
Bo_TE_191567	0.78571(33/42)	0.00000(0/18)	0.00000(0/23)	0.00000(0/19)
Bo_TE_12358	0.34146(14/41)	0.38889(7/18)	1.00000(22/22)	0.15000(3/20)
Bo_TE_129320	0.00000(0/45)	0.00000(0/19)	0.22727(5/22)	0.68421(13/19)
Bo_TE_236399	0.48837(21/43)	0.11111(2/18)	0.29412(5/17)	0.85000(17/20)
Bo_TE_182502	0.95455(42/44)	1.00000(19/19)	0.45000(9/20)	0.70588(12/17)
Bo_TE_24709	0.06522(3/46)	0.88235(15/17)	0.27273(6/22)	0.60000(12/20)
Bo_TE_9661	0.00000(0/34)	0.57143(4/7)	0.52632(10/19)	0.00000(0/16)
Bo_TE_214326	0.00000(0/44)	0.58824(10/17)	0.04545(1/22)	0.05882(1/17)
Bo_TE_49579	0.39474(15/38)	0.94118(16/17)	0.50000(10/20)	0.85000(17/20)
Bo_TE_128691	0.74419(32/43)	0.23529(4/17)	0.42857(9/21)	1.00000(20/20)
Bo_TE_127156	0.97500(39/40)	0.27778(5/18)	0.19048(4/21)	0.40000(8/20)
Bo_TE_130164	0.71429(30/42)	0.44444(8/18)	0.04762(1/21)	0.05263(1/19)
Bo_TE_92637	0.92857(39/42)	0.76471(13/17)	0.33333(7/21)	0.70588(12/17)
Bo_TE_183017	0.06977(3/43)	0.05882(1/17)	0.68421(13/19)	0.00000(0/20)

Bo_TE_167750	0.71429(30/42)	1.00000(17/17)	0.00000(0/14)	0.38889(7/18)
Bo_TE_35960	0.54762(23/42)	0.62500(10/16)	0.38095(8/21)	0.90000(18/20)
Bo_TE_96182	0.50000(21/42)	0.00000(0/19)	0.00000(0/23)	0.00000(0/20)
Bo_TE_149251	0.95000(38/40)	0.53333(8/15)	0.80952(17/21)	0.42105(8/19)
Bo_TE_133251	0.02174(1/46)	0.58824(10/17)	0.47619(10/21)	0.22222(4/18)
Bo_TE_12800	0.12195(5/41)	0.00000(0/17)	0.57143(12/21)	0.44444(8/18)
Bo_TE_4515	0.55263(21/38)	0.33333(5/15)	0.00000(0/22)	0.16667(3/18)
Bo_TE_77048	0.02439(1/41)	0.35294(6/17)	0.63158(12/19)	0.81250(13/16)
Bo_TE_193427	0.00000(0/44)	0.64706(11/17)	0.05263(1/19)	0.00000(0/18)
Bo_TE_92222	0.95455(42/44)	0.70588(12/17)	0.90476(19/21)	0.25000(4/16)
Bo_TE_44175	0.00000(0/44)	0.00000(0/18)	0.13043(3/23)	0.57895(11/19)
Bo_TE_59815	0.00000(0/46)	0.00000(0/19)	0.00000(0/22)	0.55000(11/20)
Bo_TE_53578	0.04444(2/45)	0.00000(0/17)	0.08696(2/23)	0.85000(17/20)
Bo_TE_4366	0.11628(5/43)	0.46667(7/15)	0.80952(17/21)	0.00000(0/20)
Bo_TE_226181	0.70455(31/44)	0.15789(3/19)	0.00000(0/23)	0.00000(0/17)
Bo_TE_47456	0.59524(25/42)	0.00000(0/17)	0.35000(7/20)	0.30000(6/20)
Bo_TE_17834	0.76190(32/42)	0.94118(16/17)	0.85000(17/20)	0.30000(6/20)
Bo_TE_151027	0.82927(34/41)	0.00000(0/19)	0.00000(0/20)	0.10000(1/10)
Bo_TE_216069	0.68182(30/44)	0.66667(10/15)	0.04545(1/22)	0.47368(9/19)
Bo_TE_34529	0.81395(35/43)	0.61111(11/18)	0.42857(9/21)	0.16667(3/18)
Bo_TE_15688	0.57143(24/42)	0.41176(7/17)	0.38095(8/21)	0.00000(0/20)
Bo_TE_21935	0.60465(26/43)	0.66667(12/18)	0.00000(0/23)	0.00000(0/20)
Bo_TE_132770	0.15000(6/40)	0.00000(0/17)	0.71429(15/21)	0.55556(10/18)
Bo_TE_6883	0.14634(6/41)	0.81250(13/16)	0.76190(16/21)	1.00000(20/20)
Bo_TE_147053	0.14634(6/41)	0.50000(9/18)	0.09524(2/21)	0.00000(0/19)
Bo_TE_102176	0.04762(2/42)	0.00000(0/18)	0.31818(7/22)	0.64706(11/17)
Bo_TE_120796	0.58537(24/41)	0.29412(5/17)	0.10000(2/20)	0.05000(1/20)
Bo_TE_124267	0.22222(10/45)	0.76471(13/17)	0.81818(18/22)	0.84211(16/19)
Bo_TE_75632	0.00000(0/43)	0.58824(10/17)	0.00000(0/21)	0.05000(1/20)
Bo_TE_45423	0.00000(0/38)	0.76471(13/17)	0.57895(11/19)	1.00000(14/14)
Bo_TE_57581	0.48780(20/41)	0.82353(14/17)	0.40000(8/20)	0.00000(0/19)
Bo_TE_119702	0.22727(10/44)	0.00000(0/16)	0.04348(1/23)	0.50000(10/20)
Bo_TE_12830	1.00000(44/44)	0.52941(9/17)	0.47619(10/21)	0.40000(8/20)
Bo_TE_213243	0.63415(26/41)	0.17647(3/17)	0.00000(0/22)	0.10526(2/19)
Bo_TE_132671	0.09756(4/41)	0.41176(7/17)	0.73684(14/19)	0.78947(15/19)
Bo_TE_134441	0.00000(0/42)	0.81250(13/16)	0.00000(0/23)	0.05000(1/20)
Bo_TE_75966	0.58140(25/43)	0.00000(0/18)	0.65000(13/20)	0.11765(2/17)
Bo_TE_77476	0.13953(6/43)	0.37500(6/16)	0.50000(11/22)	0.88235(15/17)
Bo_TE_61168	0.20000(8/40)	0.61111(11/18)	0.85714(18/21)	0.55000(11/20)
Bo_TE_31889	0.66667(26/39)	0.11111(2/18)	1.00000(22/22)	0.89474(17/19)
Bo_TE_57924	0.97727(43/44)	1.00000(16/16)	0.63158(12/19)	0.15789(3/19)
Bo_TE_230917	0.09091(3/33)	0.94444(17/18)	0.14286(3/21)	0.38889(7/18)
Bo_TE_114056	0.02222(1/45)	0.64706(11/17)	0.33333(7/21)	0.78947(15/19)
Bo_TE_106930	0.09756(4/41)	0.82353(14/17)	0.78261(18/23)	0.75000(15/20)
Bo_TE_86106	0.05000(2/40)	0.35714(5/14)	0.65217(15/23)	0.41176(7/17)
Bo_TE_134077	0.30233(13/43)	0.00000(0/17)	0.59091(13/22)	0.75000(15/20)
Bo_TE_61732	0.30952(13/42)	0.33333(5/15)	0.68182(15/22)	0.84211(16/19)
Bo_TE_112888	0.24390(10/41)	0.58824(10/17)	0.00000(0/23)	0.00000(0/20)
Bo_TE_103992	0.94872(37/39)	0.56250(9/16)	0.40909(9/22)	0.10526(2/19)
Bo_TE_141563	0.82222(37/45)	0.16667(3/18)	0.04545(1/22)	0.20000(4/20)
Bo_TE_60532	0.06818(3/44)	0.56250(9/16)	0.70000(14/20)	0.00000(0/19)
Bo_TE_169618	0.86667(39/45)	0.33333(6/18)	0.13043(3/23)	0.00000(0/20)
Bo_TE_54021	0.67500(27/40)	0.05882(1/17)	0.08333(1/12)	0.73333(11/15)
Bo_TE_131113	0.58537(24/41)	0.00000(0/17)	0.57143(12/21)	0.45000(9/20)
Bo_TE_200167	0.74419(32/43)	0.05882(1/17)	0.00000(0/23)	0.10526(2/19)
Bo_TE_158879	0.75556(34/45)	0.13333(2/15)	0.36842(7/19)	0.94737(18/19)

Bo_TE_219881	0.61364(27/44)	0.10526(2/19)	0.22727(5/22)	0.00000(0/20)
Bo_TE_70281	0.05000(2/40)	0.44444(8/18)	0.08696(2/23)	0.72222(13/18)
Bo_TE_56650	0.13636(6/44)	0.23529(4/17)	0.77273(17/22)	0.95000(19/20)
Bo_TE_197976	0.97561(40/41)	0.56250(9/16)	0.21053(4/19)	0.35294(6/17)
Bo_TE_44932	0.34146(14/41)	0.36842(7/19)	0.84211(16/19)	0.68750(11/16)
Bo_TE_32789	0.64103(25/39)	0.58824(10/17)	0.38095(8/21)	0.05000(1/20)
Bo_TE_113625	0.20455(9/44)	0.15789(3/19)	0.04545(1/22)	0.65000(13/20)
Bo_TE_197924	0.47619(20/42)	0.00000(0/18)	0.44444(8/18)	0.66667(12/18)
Bo_TE_83608	0.30233(13/43)	0.84211(16/19)	0.56522(13/23)	0.58824(10/17)
Bo_TE_104105	0.16667(7/42)	0.00000(0/19)	0.60000(12/20)	0.52632(10/19)
Bo_TE_237399	0.81395(35/43)	0.00000(0/16)	0.04545(1/22)	0.25000(5/20)
Bo_TE_220086	0.78378(29/37)	0.05556(1/18)	0.04762(1/21)	0.22222(4/18)
Bo_TE_191007	0.60976(25/41)	0.11765(2/17)	0.00000(0/21)	0.25000(5/20)
Bo_TE_17600	0.43902(18/41)	0.70588(12/17)	0.85714(18/21)	1.00000(20/20)
Bo_TE_236131	0.58140(25/43)	0.00000(0/17)	0.00000(0/23)	0.00000(0/18)
Bo_TE_104224	0.18182(8/44)	0.50000(8/16)	0.95238(20/21)	0.77778(14/18)
Bo_TE_8504	0.30233(13/43)	0.50000(8/16)	0.00000(0/20)	0.58824(10/17)
Bo_TE_48838	0.91111(41/45)	0.70588(12/17)	0.86364(19/22)	0.40000(8/20)
Bo_TE_129779	0.02326(1/43)	0.00000(0/18)	0.68421(13/19)	0.58824(10/17)
Bo_TE_209238	0.46667(21/45)	0.68750(11/16)	0.90476(19/21)	0.10526(2/19)
Bo_TE_205215	0.00000(0/44)	0.61111(11/18)	0.00000(0/23)	0.00000(0/20)
Bo_TE_26359	0.85714(36/42)	0.16667(3/18)	0.50000(10/20)	0.90000(18/20)
Bo_TE_216134	0.97727(43/44)	0.94444(17/18)	0.91304(21/23)	0.47368(9/19)
Bo_TE_225937	0.09091(4/44)	0.58824(10/17)	0.00000(0/21)	0.00000(0/20)
Bo_TE_85241	0.16279(7/43)	0.57895(11/19)	0.00000(0/23)	0.00000(0/20)
Bo_TE_3435	0.78378(29/37)	0.60000(9/15)	0.00000(0/23)	0.00000(0/19)
Bo_TE_165251	0.54762(23/42)	0.00000(0/17)	0.15000(3/20)	0.10000(2/20)
Bo_TE_21264	0.27273(12/44)	0.00000(0/18)	0.55000(11/20)	0.52941(9/17)
Bo_TE_155790	0.00000(0/45)	0.00000(0/19)	0.50000(11/22)	0.00000(0/20)
Bo_TE_235798	1.00000(46/46)	0.77778(14/18)	0.47826(11/23)	0.15000(3/20)
Bo_TE_121321	0.37778(17/45)	0.17647(3/17)	0.65217(15/23)	0.84211(16/19)
Bo_TE_180010	0.15909(7/44)	0.50000(8/16)	0.00000(0/22)	0.38889(7/18)
Bo_TE_70665	0.63636(28/44)	0.05882(1/17)	0.31818(7/22)	0.38889(7/18)
Bo_TE_189887	0.61905(26/42)	0.64706(11/17)	0.00000(0/23)	0.00000(0/20)
Bo_TE_155148	0.07317(3/41)	0.11765(2/17)	0.27273(6/22)	0.70000(14/20)
Bo_TE_156877	0.23684(9/38)	0.00000(0/17)	0.75000(15/20)	0.10526(2/19)
Bo_TE_225417	0.34884(15/43)	0.70588(12/17)	0.95652(22/23)	0.52632(10/19)
Bo_TE_75259	0.50000(21/42)	1.00000(19/19)	0.15000(3/20)	0.73684(14/19)
Bo_TE_26474	0.14286(6/42)	0.58824(10/17)	0.80000(16/20)	0.85000(17/20)
Bo_TE_37110	0.52381(22/42)	0.00000(0/18)	0.00000(0/23)	0.05263(1/19)
Bo_TE_156064	0.82222(37/45)	0.83333(15/18)	0.26087(6/23)	0.72222(13/18)
Bo_TE_10612	0.83721(36/43)	0.29412(5/17)	0.00000(0/22)	0.60000(12/20)
Bo_TE_195393	0.18605(8/43)	0.70588(12/17)	0.00000(0/22)	0.55556(10/18)
Bo_TE_201513	0.37209(16/43)	0.72222(13/18)	0.89474(17/19)	0.89474(17/19)
Bo_TE_154615	0.30952(13/42)	0.25000(4/16)	0.76190(16/21)	0.26316(5/19)
Bo_TE_12930	0.86364(38/44)	0.11765(2/17)	0.17391(4/23)	0.35000(7/20)
Bo_TE_76050	0.08696(4/46)	0.00000(0/18)	0.61905(13/21)	0.15789(3/19)
Bo_TE_18821	0.37778(17/45)	0.66667(12/18)	0.04348(1/23)	0.15000(3/20)
Bo_TE_170602	0.00000(0/40)	0.05263(1/19)	0.55000(11/20)	0.00000(0/20)
Bo_TE_57156	0.00000(0/45)	0.00000(0/19)	0.21739(5/23)	0.57895(11/19)
Bo_TE_173612	0.27907(12/43)	0.58824(10/17)	0.00000(0/23)	0.00000(0/19)
Bo_TE_172800	0.11111(5/45)	0.88889(16/18)	1.00000(23/23)	0.94737(18/19)
Bo_TE_25576	0.90476(38/42)	0.44444(8/18)	0.70000(14/20)	0.23529(4/17)
Bo_TE_223025	0.04545(2/44)	0.05556(1/18)	0.00000(0/22)	0.68421(13/19)
Bo_TE_108220	0.02273(1/44)	0.31250(5/16)	0.70000(14/20)	0.33333(6/18)
Bo_TE_12863	0.22222(8/36)	0.12500(2/16)	0.63158(12/19)	0.62500(10/16)

Bo_TE_89469	0.70732(29/41)	0.68750(11/16)	0.09091(2/22)	0.05263(1/19)
Bo_TE_25038	0.16279(7/43)	0.31250(5/16)	0.08696(2/23)	0.85000(17/20)
Bo_TE_7977	0.00000(0/46)	0.00000(0/18)	0.61905(13/21)	0.16667(3/18)
Bo_TE_163598	0.11111(5/45)	0.00000(0/18)	0.18182(4/22)	0.65000(13/20)
Bo_TE_129346	0.83333(10/12)	0.66667(2/3)	1.00000(23/23)	0.16667(3/18)
Bo_TE_62228	0.59524(25/42)	0.78947(15/19)	0.00000(0/22)	0.05000(1/20)
Bo_TE_3587	0.06522(3/46)	0.70588(12/17)	0.00000(0/21)	0.15789(3/19)
Bo_TE_216566	0.11905(5/42)	0.05556(1/18)	0.27273(6/22)	0.60000(12/20)
Bo_TE_28635	0.65116(28/43)	0.83333(15/18)	0.50000(8/16)	0.25000(4/16)
Bo_TE_17983	0.04651(2/43)	0.18750(3/16)	0.40000(8/20)	0.75000(15/20)
Bo_TE_132205	0.02222(1/45)	0.00000(0/19)	0.50000(10/20)	0.00000(0/19)
Bo_TE_133250	0.86667(39/45)	0.68750(11/16)	0.09091(2/22)	0.78947(15/19)
Bo_TE_4531	0.13636(6/44)	0.64706(11/17)	0.04762(1/21)	0.29412(5/17)
Bo_TE_223006	0.35897(14/39)	0.05556(1/18)	0.00000(0/23)	0.68421(13/19)
Bo_TE_235619	0.28889(13/45)	0.88889(16/18)	0.00000(0/22)	0.30000(6/20)
Bo_TE_92245	0.32500(13/40)	0.50000(9/18)	0.95455(21/22)	0.20000(4/20)
Bo_TE_71476	0.00000(0/45)	0.00000(0/18)	0.52174(12/23)	0.30000(6/20)
Bo_TE_238648	0.61538(24/39)	1.00000(11/11)	0.00000(0/7)	0.00000(0/19)
Bo_TE_230438	0.34884(15/43)	1.00000(18/18)	0.90476(19/21)	0.15000(3/20)
Bo_TE_196533	0.72093(31/43)	0.50000(9/18)	0.00000(0/21)	0.00000(0/20)
Bo_TE_90243	0.13158(5/38)	0.40000(4/10)	0.00000(0/22)	0.80000(16/20)
Bo_TE_65997	0.52381(22/42)	0.87500(14/16)	0.08696(2/23)	0.82353(14/17)
Bo_TE_177594	1.00000(45/45)	1.00000(19/19)	0.60000(12/20)	0.50000(10/20)
Bo_TE_180808	0.58140(25/43)	0.58824(10/17)	0.00000(0/22)	0.00000(0/19)
Bo_TE_136726	0.59524(25/42)	0.35294(6/17)	0.00000(0/21)	0.15789(3/19)
Bo_TE_24900	0.08889(4/45)	0.72222(13/18)	0.21739(5/23)	0.20000(4/20)
Bo_TE_53538	0.02222(1/45)	0.00000(0/18)	0.04545(1/22)	0.84211(16/19)
Bo_TE_41839	0.95349(41/43)	0.44444(8/18)	0.72727(16/22)	0.44444(8/18)
Bo_TE_109856	0.77778(28/36)	0.62500(10/16)	0.00000(0/22)	0.00000(0/18)
Bo_TE_215963	0.62222(28/45)	0.73684(14/19)	0.04348(1/23)	0.05000(1/20)
Bo_TE_154844	0.76190(32/42)	0.25000(4/16)	0.85714(18/21)	0.95000(19/20)
Bo_TE_121893	0.53659(22/41)	0.16667(3/18)	0.14286(3/21)	0.75000(15/20)
Bo_TE_170911	0.38462(15/39)	0.11765(2/17)	0.47619(10/21)	0.85000(17/20)
Bo_TE_51507	0.77273(34/44)	0.37500(6/16)	1.00000(22/22)	0.89474(17/19)
Bo_TE_94431	0.00000(0/45)	0.05556(1/18)	0.45000(9/20)	0.60000(12/20)
Bo_TE_197261	0.00000(0/43)	0.10526(2/19)	0.00000(0/22)	0.50000(9/18)
Bo_TE_93181	0.37209(16/43)	0.89474(17/19)	0.00000(0/23)	0.47368(9/19)
Bo_TE_224285	0.36111(13/36)	0.05882(1/17)	0.63158(12/19)	0.07692(1/13)
Bo_TE_230828	0.04651(2/43)	0.35294(6/17)	0.47619(10/21)	0.73684(14/19)
Bo_TE_237214	0.79487(31/39)	0.05556(1/18)	0.00000(0/12)	0.10526(2/19)
Bo_TE_220814	0.00000(0/45)	0.72222(13/18)	0.00000(0/21)	0.00000(0/20)
Bo_TE_163038	1.00000(45/45)	1.00000(17/17)	0.23810(5/21)	0.77778(14/18)
Bo_TE_88839	0.13636(6/44)	0.16667(3/18)	0.90909(20/22)	0.57895(11/19)
Bo_TE_234778	0.85714(36/42)	0.28571(4/14)	0.04348(1/23)	0.11111(2/18)
Bo_TE_101608	0.65116(28/43)	0.52941(9/17)	0.00000(0/23)	0.33333(6/18)
Bo_TE_122148	0.45238(19/42)	0.66667(12/18)	0.95455(21/22)	0.63158(12/19)
Bo_TE_22300	0.00000(0/45)	0.16667(3/18)	0.61905(13/21)	0.00000(0/20)
Bo_TE_20429	0.00000(0/45)	0.33333(6/18)	0.80000(16/20)	0.36842(7/19)
Bo_TE_172042	0.02273(1/44)	0.00000(0/16)	0.78947(15/19)	0.00000(0/19)
Bo_TE_2336	0.04545(2/44)	0.43750(7/16)	0.27273(6/22)	0.75000(15/20)
Bo_TE_236509	0.36111(13/36)	0.00000(0/15)	0.19048(4/21)	0.84211(16/19)
Bo_TE_100018	0.46341(19/41)	0.76471(13/17)	0.21053(4/19)	0.10526(2/19)
Bo_TE_236654	0.97778(44/45)	0.52941(9/17)	0.22727(5/22)	0.95000(19/20)
Bo_TE_129684	0.46667(21/45)	0.82353(14/17)	1.00000(22/22)	0.47368(9/19)
Bo_TE_52619	0.34146(14/41)	0.11111(2/18)	0.28571(6/21)	0.63158(12/19)
Bo_TE_105513	0.47500(19/40)	0.11765(2/17)	0.65000(13/20)	0.52941(9/17)

Bo_TE_70019	0.41026(16/39)	0.05882(1/17)	0.80952(17/21)	0.50000(10/20)
Bo_TE_203702	0.00000(0/46)	0.68750(11/16)	0.15000(3/20)	0.00000(0/20)
Bo_TE_60400	0.14286(6/42)	0.66667(12/18)	0.90000(18/20)	0.85000(17/20)
Bo_TE_198185	0.64103(25/39)	0.37500(6/16)	0.77778(14/18)	0.00000(0/18)
Bo_TE_201736	0.27500(11/40)	0.37500(6/16)	0.68182(15/22)	0.15000(3/20)
Bo_TE_21454	0.12821(5/39)	0.55556(10/18)	0.95455(21/22)	0.95000(19/20)
Bo_TE_223067	0.02273(1/44)	0.00000(0/18)	0.55000(11/20)	0.10000(2/20)
Bo_TE_93549	0.95652(44/46)	0.12500(2/16)	1.00000(22/22)	1.00000(20/20)
Bo_TE_137797	0.32500(13/40)	0.60000(9/15)	0.75000(15/20)	0.05000(1/20)
Bo_TE_128326	0.28571(12/42)	0.68750(11/16)	0.33333(7/21)	0.95000(19/20)
Bo_TE_185375	0.02222(1/45)	0.55556(10/18)	0.35000(7/20)	0.15789(3/19)
Bo_TE_41313	0.38095(16/42)	0.11111(2/18)	0.86364(19/22)	0.45000(9/20)
Bo_TE_145393	0.17500(7/40)	0.82353(14/17)	0.00000(0/23)	0.05000(1/20)
Bo_TE_141684	0.00000(0/45)	0.00000(0/19)	0.77273(17/22)	0.33333(6/18)
Bo_TE_28314	0.00000(0/45)	0.00000(0/19)	0.65000(13/20)	0.00000(0/20)
Bo_TE_126392	0.38095(16/42)	0.66667(12/18)	0.90909(20/22)	0.82353(14/17)
Bo_TE_61359	0.02273(1/44)	0.27778(5/18)	0.86364(19/22)	0.52632(10/19)
Bo_TE_12901	0.38095(16/42)	0.94118(16/17)	0.77778(14/18)	0.65000(13/20)
Bo_TE_80214	0.00000(0/46)	0.66667(12/18)	0.00000(0/23)	0.00000(0/20)
Bo_TE_143843	0.18182(8/44)	0.00000(0/17)	0.05556(1/18)	0.50000(10/20)
Bo_TE_213845	0.07317(3/41)	0.11765(2/17)	0.76190(16/21)	0.55556(10/18)
Bo_TE_52784	0.27907(12/43)	0.00000(0/15)	0.89474(17/19)	0.31579(6/19)
Bo_TE_1147	0.73529(25/34)	0.29412(5/17)	0.33333(7/21)	0.95000(19/20)
Bo_TE_230954	0.13953(6/43)	0.05556(1/18)	0.66667(14/21)	0.05556(1/18)
Bo_TE_125351	0.43902(18/41)	0.38889(7/18)	0.04545(1/22)	0.78947(15/19)
Bo_TE_121436	1.00000(45/45)	1.00000(18/18)	0.95455(21/22)	0.22222(4/18)
Bo_TE_145112	0.84091(37/44)	0.75000(12/16)	0.90000(18/20)	0.30000(6/20)
Bo_TE_186059	0.00000(0/45)	0.58824(10/17)	0.28571(6/21)	0.10526(2/19)
Bo_TE_238452	0.64286(27/42)	0.76471(13/17)	0.00000(0/23)	0.00000(0/20)
Bo_TE_235850	0.86047(37/43)	0.05556(1/18)	0.39130(9/23)	0.00000(0/20)
Bo_TE_109443	0.37209(16/43)	0.62500(10/16)	0.04348(1/23)	0.15789(3/19)
Bo_TE_143986	0.00000(0/45)	0.00000(0/19)	0.81818(18/22)	0.89474(17/19)
Bo_TE_236775	0.11628(5/43)	0.47059(8/17)	0.78261(18/23)	0.90000(18/20)
Bo_TE_169489	0.86667(39/45)	1.00000(17/17)	0.20000(4/20)	0.68421(13/19)
Bo_TE_126221	0.00000(0/44)	0.61111(11/18)	0.09091(2/22)	0.00000(0/20)
Bo_TE_164632	0.88372(38/43)	0.11765(2/17)	0.00000(0/22)	0.21053(4/19)
Bo_TE_38020	0.86667(39/45)	0.82353(14/17)	1.00000(21/21)	0.50000(10/20)
Bo_TE_107832	0.76087(35/46)	0.26316(5/19)	0.00000(0/21)	0.00000(0/19)
Bo_TE_150573	0.58537(24/41)	0.41176(7/17)	0.04545(1/22)	0.20000(4/20)
Bo_TE_202730	0.92500(37/40)	0.42105(8/19)	0.42857(9/21)	0.10526(2/19)
Bo_TE_110187	0.02222(1/45)	0.16667(3/18)	0.57143(12/21)	0.00000(0/20)
Bo_TE_151000	0.51111(23/45)	0.72222(13/18)	0.04348(1/23)	0.00000(0/19)
Bo_TE_79980	0.52273(23/44)	0.00000(0/16)	0.60870(14/23)	0.15000(3/20)
Bo_TE_224171	0.60465(26/43)	0.76471(13/17)	0.86364(19/22)	0.29412(5/17)
Bo_TE_61164	0.00000(0/46)	0.00000(0/18)	0.15789(3/19)	0.50000(9/18)
Bo_TE_120542	0.06977(3/43)	0.16667(3/18)	0.10000(2/20)	0.73684(14/19)
Bo_TE_66472	0.07143(3/42)	0.27778(5/18)	0.77273(17/22)	0.70000(14/20)
Bo_TE_162410	0.59091(26/44)	0.72222(13/18)	0.04348(1/23)	0.15000(3/20)
Bo_TE_215597	0.97727(43/44)	1.00000(19/19)	0.52381(11/21)	0.45000(9/20)
Bo_TE_169191	0.00000(0/46)	0.00000(0/18)	0.59091(13/22)	0.25000(5/20)
Bo_TE_109006	0.00000(0/46)	0.25000(4/16)	0.25000(5/20)	0.70000(14/20)
Bo_TE_182247	0.22727(10/44)	0.16667(3/18)	0.77273(17/22)	0.26316(5/19)
Bo_TE_9879	0.00000(0/44)	0.05556(1/18)	0.85714(18/21)	0.00000(0/20)
Bo_TE_232197	0.28571(12/42)	0.88889(16/18)	0.42857(9/21)	0.50000(10/20)
Bo_TE_142596	0.59091(26/44)	0.22222(4/18)	0.04545(1/22)	0.52632(10/19)
Bo_TE_21969	0.37209(16/43)	0.17647(3/17)	0.40000(8/20)	1.00000(19/19)

Bo_TE_61755	0.24390(10/41)	0.29412(5/17)	0.70000(14/20)	0.84211(16/19)
Bo_TE_115587	0.67647(23/34)	0.93750(15/16)	0.86364(19/22)	0.23529(4/17)
Bo_TE_175033	1.00000(44/44)	1.00000(18/18)	0.59091(13/22)	0.44444(8/18)
Bo_TE_75770	0.97727(43/44)	0.75000(12/16)	0.95455(21/22)	0.29412(5/17)
Bo_TE_201882	0.06667(3/45)	0.05882(1/17)	0.47368(9/19)	0.78947(15/19)
Bo_TE_156913	0.17949(7/39)	0.81250(13/16)	0.37500(6/16)	0.12500(1/8)
Bo_TE_227339	0.55000(22/40)	0.50000(8/16)	0.20000(4/20)	1.00000(20/20)
Bo_TE_124370	0.88636(39/44)	0.38889(7/18)	1.00000(23/23)	0.95000(19/20)
Bo_TE_56360	0.23256(10/43)	0.77778(14/18)	0.82609(19/23)	0.83333(15/18)
Bo_TE_231509	0.00000(0/45)	0.06250(1/16)	0.54545(12/22)	0.52632(10/19)
Bo_TE_119577	0.33333(15/45)	1.00000(18/18)	0.31818(7/22)	0.66667(12/18)
Bo_TE_195408	0.20455(9/44)	0.70588(12/17)	0.04348(1/23)	0.50000(10/20)
Bo_TE_42395	0.25000(10/40)	0.23529(4/17)	0.20000(4/20)	0.73684(14/19)
Bo_TE_129369	0.73171(30/41)	0.00000(0/16)	0.17391(4/23)	0.00000(0/18)
Bo_TE_212228	0.77778(35/45)	0.31250(5/16)	0.47368(9/19)	1.00000(18/18)
Bo_TE_54569	0.52381(22/42)	0.44444(8/18)	0.95238(20/21)	0.10526(2/19)
Bo_TE_153605	0.34884(15/43)	0.94118(16/17)	0.50000(11/22)	1.00000(19/19)
Bo_TE_72376	0.84444(38/45)	0.60000(9/15)	0.05263(1/19)	0.05263(1/19)
Bo_TE_201712	0.02273(1/44)	0.00000(0/17)	0.71429(15/21)	0.05000(1/20)
Bo_TE_224598	0.46875(15/32)	0.81250(13/16)	0.19048(4/21)	0.78571(11/14)
Bo_TE_85471	0.75610(31/41)	0.31579(6/19)	0.50000(9/18)	0.94737(18/19)
Bo_TE_211137	0.77273(34/44)	0.41176(7/17)	0.04545(1/22)	0.00000(0/20)
Bo_TE_159313	0.09302(4/43)	0.36364(4/11)	0.35000(7/20)	0.77778(14/18)
Bo_TE_56768	0.12821(5/39)	0.43750(7/16)	0.73684(14/19)	0.89474(17/19)
Bo_TE_127456	0.27907(12/43)	0.87500(14/16)	0.05000(1/20)	0.50000(10/20)
Bo_TE_159952	0.59524(25/42)	0.50000(9/18)	0.04348(1/23)	0.20000(4/20)
Bo_TE_220508	0.66667(26/39)	0.82353(14/17)	0.57143(12/21)	0.20000(4/20)
Bo_TE_32637	0.17949(7/39)	0.93750(15/16)	0.41176(7/17)	0.27778(5/18)
Bo_TE_68185	0.30233(13/43)	0.81250(13/16)	0.08696(2/23)	0.10526(2/19)
Bo_TE_45690	0.79070(34/43)	0.00000(0/17)	0.28571(6/21)	0.10000(2/20)
Bo_TE_133275	0.80000(32/40)	0.35294(6/17)	0.09091(2/22)	0.78947(15/19)
Bo_TE_31510	0.65789(25/38)	0.00000(0/17)	0.00000(0/18)	0.00000(0/18)
Bo_TE_117869	0.09091(4/44)	0.70588(12/17)	0.23810(5/21)	0.00000(0/20)
Bo_TE_76750	0.57143(24/42)	0.05882(1/17)	0.57143(12/21)	0.00000(0/20)
Bo_TE_70674	1.00000(45/45)	0.60000(9/15)	0.30435(7/23)	0.52941(9/17)
Bo_TE_80442	0.04878(2/41)	0.00000(0/18)	0.47619(10/21)	0.80000(16/20)
Bo_TE_240722	0.75610(31/41)	0.15789(3/19)	0.00000(0/23)	0.05000(1/20)
Bo_TE_138974	0.04348(2/46)	0.88235(15/17)	0.77273(17/22)	0.68421(13/19)
Bo_TE_44032	0.66667(28/42)	0.18750(3/16)	0.81818(18/22)	0.90000(18/20)
Bo_TE_129441	0.15385(6/39)	0.88889(16/18)	0.31579(6/19)	1.00000(19/19)
Bo_TE_40696	0.00000(0/46)	0.00000(0/16)	0.68421(13/19)	0.31579(6/19)
Bo_TE_19149	1.00000(40/40)	1.00000(18/18)	0.68182(15/22)	0.50000(9/18)
Bo_TE_234152	0.67568(25/37)	0.09091(1/11)	0.00000(0/23)	0.33333(1/3)
Bo_TE_31296	0.77778(35/45)	0.00000(0/17)	0.00000(0/23)	0.00000(0/20)
Bo_TE_70238	0.09302(4/43)	0.47368(9/19)	0.09524(2/21)	0.88235(15/17)
Bo_TE_143731	0.93478(43/46)	0.64706(11/17)	0.84211(16/19)	0.23529(4/17)
Bo_TE_239342	0.08889(4/45)	0.56250(9/16)	0.80952(17/21)	0.11111(2/18)
Bo_TE_85441	0.00000(0/44)	0.36364(4/11)	0.70000(14/20)	0.38462(5/13)
Bo_TE_53801	0.29545(13/44)	0.76471(13/17)	0.00000(0/21)	0.00000(0/20)
Bo_TE_234082	0.00000(0/44)	0.66667(12/18)	0.00000(0/21)	0.00000(0/20)
Bo_TE_61989	0.60000(27/45)	0.50000(8/16)	0.50000(10/20)	1.00000(20/20)
Bo_TE_50135	0.70270(26/37)	0.77778(14/18)	1.00000(23/23)	0.47368(9/19)
Bo_TE_94738	0.30952(13/42)	0.88235(15/17)	0.95455(21/22)	0.50000(10/20)
Bo_TE_6148	0.69767(30/43)	0.37500(6/16)	0.95000(19/20)	0.52941(9/17)
Bo_TE_107541	0.44186(19/43)	0.41176(7/17)	0.77273(17/22)	0.05263(1/19)
Bo_TE_43774	0.18182(8/44)	0.75000(12/16)	0.68182(15/22)	0.33333(6/18)

Bo_TE_7963	0.92857(39/42)	1.00000(18/18)	0.14286(3/21)	0.66667(12/18)
Bo_TE_78567	0.13953(6/43)	0.25000(4/16)	0.20000(4/20)	0.94737(18/19)
Bo_TE_69653	0.93182(41/44)	0.70588(12/17)	0.22222(4/18)	0.12500(2/16)
Bo_TE_112254	0.00000(0/46)	0.57895(11/19)	0.00000(0/23)	0.10000(2/20)
Bo_TE_224445	0.28571(12/42)	0.05263(1/19)	0.63636(14/22)	0.11111(2/18)
Bo_TE_127264	0.23256(10/43)	0.82353(14/17)	1.00000(23/23)	0.75000(15/20)
Bo_TE_81930	0.89744(35/39)	1.00000(15/15)	0.95000(19/20)	0.12500(2/16)
Bo_TE_162955	0.20000(9/45)	0.70588(12/17)	0.36364(8/22)	0.20000(4/20)
Bo_TE_22821	0.92857(39/42)	0.37500(6/16)	1.00000(23/23)	1.00000(18/18)
Bo_TE_179861	0.95000(38/40)	0.42857(6/14)	0.36842(7/19)	0.13333(2/15)
Bo_TE_61237	0.53659(22/41)	0.70588(12/17)	0.47619(10/21)	0.00000(0/20)
Bo_TE_70495	0.47619(20/42)	0.88235(15/17)	1.00000(21/21)	0.90000(18/20)
Bo_TE_97548	0.23256(10/43)	0.22222(4/18)	0.78261(18/23)	0.53333(8/15)
Bo_TE_180620	0.80952(34/42)	0.18750(3/16)	1.00000(23/23)	1.00000(20/20)
Bo_TE_52132	1.00000(45/45)	0.87500(14/16)	0.95455(21/22)	0.50000(10/20)
Bo_TE_237290	0.51163(22/43)	0.88889(16/18)	0.00000(0/22)	0.00000(0/18)
Bo_TE_100225	0.15909(7/44)	0.00000(0/17)	0.59091(13/22)	0.47368(9/19)
Bo_TE_143410	0.51220(21/41)	0.35294(6/17)	0.13636(3/22)	0.68421(13/19)
Bo_TE_115302	0.60000(24/40)	0.82353(14/17)	0.15000(3/20)	0.26316(5/19)
Bo_TE_180901	0.56818(25/44)	0.23529(4/17)	0.59091(13/22)	0.05000(1/20)
Bo_TE_35050	0.90909(40/44)	0.64706(11/17)	0.13043(3/23)	0.13333(2/15)
Bo_TE_49991	0.65854(27/41)	0.94444(17/18)	1.00000(23/23)	0.30000(6/20)
Bo_TE_194507	0.06977(3/43)	0.05556(1/18)	0.81818(18/22)	0.22222(4/18)
Bo_TE_153761	0.29268(12/41)	0.05882(1/17)	0.35000(7/20)	0.70588(12/17)
Bo_TE_177912	0.00000(0/45)	0.00000(0/18)	0.09091(2/22)	0.63158(12/19)
Bo_TE_55798	0.03030(1/33)	0.29412(5/17)	0.82609(19/23)	0.38889(7/18)
Bo_TE_180034	0.40476(17/42)	0.50000(8/16)	0.00000(0/20)	0.15000(3/20)
Bo_TE_56718	0.13333(6/45)	0.63636(7/11)	0.28571(4/14)	0.95000(19/20)
Bo_TE_213840	0.06522(3/46)	0.11111(2/18)	0.71429(15/21)	0.57895(11/19)
Bo_TE_134494	0.35714(15/42)	0.93750(15/16)	1.00000(22/22)	0.95000(19/20)
Bo_TE_127408	0.28571(12/42)	0.11111(2/18)	1.00000(23/23)	0.68421(13/19)
Bo_TE_212926	0.34884(15/43)	0.05556(1/18)	0.57143(12/21)	0.42105(8/19)
Bo_TE_68192	0.28571(12/42)	0.81250(13/16)	0.09091(2/22)	0.10000(2/20)
Bo_TE_63575	0.82927(34/41)	0.11765(2/17)	0.54545(12/22)	1.00000(20/20)
Bo_TE_100183	0.20930(9/43)	0.00000(0/18)	0.57895(11/19)	0.50000(9/18)
Bo_TE_111050	0.64286(27/42)	0.29412(5/17)	1.00000(23/23)	0.63158(12/19)
Bo_TE_134102	0.13636(6/44)	0.64706(11/17)	0.91304(21/23)	0.50000(8/16)
Bo_TE_195565	0.16667(7/42)	0.41176(7/17)	0.52174(12/23)	0.78947(15/19)
Bo_TE_114290	0.23256(10/43)	0.20000(3/15)	0.04348(1/23)	0.57895(11/19)
Bo_TE_143472	0.02273(1/44)	0.00000(0/19)	0.09524(2/21)	0.73684(14/19)
Bo_TE_170629	0.57143(24/42)	0.44444(8/18)	0.00000(0/22)	0.70000(14/20)
Bo_TE_114204	0.82927(34/41)	0.35294(6/17)	1.00000(21/21)	0.70000(14/20)
Bo_TE_143632	0.28571(12/42)	0.41176(7/17)	1.00000(22/22)	0.75000(6/8)
Bo_TE_93405	0.66667(28/42)	0.05556(1/18)	0.00000(0/23)	0.00000(0/20)
Bo_TE_90832	0.75000(33/44)	0.68750(11/16)	0.42857(9/21)	0.00000(0/19)
Bo_TE_141213	0.81395(35/43)	0.05882(1/17)	0.04762(1/21)	0.10526(2/19)
Bo_TE_143991	0.87500(35/40)	0.41176(7/17)	0.09091(2/22)	0.00000(0/20)
Bo_TE_164042	1.00000(45/45)	0.43750(7/16)	0.40000(8/20)	0.50000(10/20)
Bo_TE_7581	0.72093(31/43)	0.06250(1/16)	0.36364(8/22)	0.00000(0/20)
Bo_TE_173204	0.60976(25/41)	0.26667(4/15)	0.19048(4/21)	0.10526(2/19)
Bo_TE_65020	0.45238(19/42)	1.00000(19/19)	0.38095(8/21)	0.52941(9/17)
Bo_TE_76873	0.51220(21/41)	0.23529(4/17)	0.00000(0/20)	0.00000(0/20)
Bo_TE_129401	0.20000(8/40)	0.94118(16/17)	0.28571(6/21)	0.70000(14/20)
Bo_TE_82210	0.21429(9/42)	0.70588(12/17)	0.61905(13/21)	0.95000(19/20)
Bo_TE_80673	0.00000(0/20)	0.00000(0/13)	0.00000(0/23)	0.72222(13/18)
Bo_TE_133222	0.13333(6/45)	0.00000(0/19)	0.71429(15/21)	0.61111(11/18)

Bo_TE_156811	0.73913(34/46)	0.88889(16/18)	0.50000(11/22)	0.26316(5/19)
Bo_TE_93044	0.00000(0/44)	0.00000(0/19)	0.65000(13/20)	0.31579(6/19)
Bo_TE_95957	0.34884(15/43)	0.35294(6/17)	1.00000(23/23)	0.95000(19/20)
Bo_TE_89221	0.51220(21/41)	0.05882(1/17)	0.50000(3/6)	0.88889(16/18)
Bo_TE_92027	0.32500(13/40)	1.00000(18/18)	1.00000(22/22)	0.42105(8/19)
Bo_TE_60820	0.17073(7/41)	0.14286(2/14)	0.90909(20/22)	0.57895(11/19)
Bo_TE_215877	0.34211(13/38)	0.58824(10/17)	0.00000(0/23)	0.00000(0/20)
Bo_TE_240613	0.86364(38/44)	0.15789(3/19)	0.04545(1/22)	0.47368(9/19)
Bo_TE_138156	0.25000(10/40)	0.05882(1/17)	0.85714(18/21)	0.52632(10/19)
Bo_TE_222375	0.02222(1/45)	0.68421(13/19)	0.00000(0/23)	0.00000(0/20)
Bo_TE_226571	0.35135(13/37)	0.11765(2/17)	0.52632(10/19)	0.68421(13/19)
Bo_TE_109709	0.22500(9/40)	0.23529(4/17)	0.45455(10/22)	1.00000(16/16)
Bo_TE_149198	0.97727(43/44)	0.89474(17/19)	0.20000(4/20)	0.52632(10/19)
Bo_TE_220865	0.13636(3/22)	0.76471(13/17)	0.20000(3/15)	0.05263(1/19)
Bo_TE_226335	0.68421(26/38)	0.16667(3/18)	1.00000(23/23)	0.95000(19/20)
Bo_TE_165149	0.09091(4/44)	0.64706(11/17)	0.30000(6/20)	0.00000(0/18)
Bo_TE_14540	0.75000(30/40)	0.33333(6/18)	0.80952(17/21)	1.00000(19/19)
Bo_TE_107982	0.00000(0/43)	0.68750(11/16)	0.00000(0/23)	0.00000(0/20)
Bo_TE_15521	0.00000(0/42)	0.06667(1/15)	1.00000(17/17)	0.87500(14/16)
Bo_TE_191244	0.69767(30/43)	0.06250(1/16)	0.00000(0/21)	0.10000(2/20)
Bo_TE_45874	0.00000(0/43)	0.55556(10/18)	0.09091(2/22)	0.25000(5/20)
Bo_TE_38261	0.11628(5/43)	0.18750(3/16)	0.86957(20/23)	0.25000(5/20)
Bo_TE_236080	0.00000(0/45)	0.00000(0/16)	0.54545(12/22)	0.89474(17/19)
Bo_TE_124198	0.22222(10/45)	0.72222(13/18)	0.80000(16/20)	0.85000(17/20)
Bo_TE_58854	0.44444(20/45)	1.00000(17/17)	1.00000(23/23)	0.70000(14/20)
Bo_TE_97408	0.58140(25/43)	0.00000(0/18)	0.00000(0/22)	0.21053(4/19)
Bo_TE_138406	0.85294(29/34)	0.94444(17/18)	0.90476(19/21)	0.35294(6/17)
Bo_TE_195935	0.45455(20/44)	0.77778(14/18)	0.63158(12/19)	0.15000(3/20)
Bo_TE_60826	0.25641(10/39)	0.18750(3/16)	0.95455(21/22)	0.75000(15/20)
Bo_TE_100868	0.93182(41/44)	1.00000(15/15)	0.75000(15/20)	0.11765(2/17)
Bo_TE_44838	0.36842(14/38)	0.88235(15/17)	0.89474(17/19)	0.77778(14/18)
Bo_TE_137090	0.67500(27/40)	0.12500(2/16)	0.00000(0/22)	0.00000(0/17)
Bo_TE_135249	0.15909(7/44)	0.58824(10/17)	0.47619(10/21)	0.00000(0/20)
Bo_TE_148756	0.35000(14/40)	0.11111(2/18)	0.95455(21/22)	1.00000(19/19)
Bo_TE_92914	0.31579(12/38)	0.22222(4/18)	0.86667(13/15)	0.83333(15/18)
Bo_TE_100761	0.81395(35/43)	0.88889(16/18)	0.90909(20/22)	0.15789(3/19)
Bo_TE_163317	0.03030(1/33)	0.52632(10/19)	0.00000(0/18)	0.00000(0/17)
Bo_TE_239036	0.93023(40/43)	0.94444(17/18)	0.47619(10/21)	1.00000(20/20)
Bo_TE_161026	0.11628(5/43)	0.35294(6/17)	0.95000(19/20)	0.84615(11/13)
Bo_TE_60977	0.33333(14/42)	0.35294(6/17)	0.85714(18/21)	0.26316(5/19)
Bo_TE_62399	1.00000(45/45)	1.00000(17/17)	0.95238(20/21)	0.35294(6/17)
Bo_TE_152163	0.29545(13/44)	0.94444(17/18)	0.80952(17/21)	1.00000(19/19)
Bo_TE_153317	0.69767(30/43)	0.73684(14/19)	0.90476(19/21)	0.25000(5/20)
Bo_TE_179882	0.67500(27/40)	0.05263(1/19)	0.27273(6/22)	0.00000(0/20)
Bo_TE_119656	0.67442(29/43)	0.00000(0/16)	0.54545(12/22)	0.00000(0/18)
Bo_TE_101604	0.66667(28/42)	0.52941(9/17)	0.00000(0/20)	0.30000(6/20)
Bo_TE_144906	0.11364(5/44)	0.11111(2/18)	0.00000(0/23)	0.55000(11/20)
Bo_TE_147818	0.87179(34/39)	0.76471(13/17)	0.27778(5/18)	0.33333(6/18)
Bo_TE_201693	0.58537(24/41)	0.12500(2/16)	0.61905(13/21)	0.10526(2/19)
Bo_TE_203649	0.16279(7/43)	0.50000(8/16)	0.00000(0/21)	0.35294(6/17)
Bo_TE_143605	0.13953(6/43)	0.70588(12/17)	0.20000(4/20)	0.00000(0/20)
Bo_TE_101168	0.48780(20/41)	0.50000(8/16)	0.10000(2/20)	0.88235(15/17)
Bo_TE_77471	0.20000(8/40)	0.37500(6/16)	0.47826(11/23)	0.88889(16/18)
Bo_TE_205144	0.82500(33/40)	0.27778(5/18)	0.50000(10/20)	0.11765(2/17)
Bo_TE_215717	0.38095(16/42)	0.75000(12/16)	0.00000(0/22)	0.05000(1/20)
Bo_TE_225273	0.58140(25/43)	0.47059(8/17)	0.70000(14/20)	0.00000(0/20)

Bo_TE_103971	0.00000(0/45)	0.50000(9/18)	0.52381(11/21)	0.61111(11/18)
Bo_TE_34037	0.02381(1/42)	0.44444(8/18)	0.20000(4/20)	0.83333(15/18)
Bo_TE_70022	0.60000(24/40)	0.94118(16/17)	0.25000(5/20)	0.65000(13/20)
Bo_TE_132821	0.35556(16/45)	0.11765(2/17)	0.71429(15/21)	0.73684(14/19)
Bo_TE_58441	0.86047(37/43)	0.94737(18/19)	0.38095(8/21)	0.90000(18/20)
Bo_TE_12850	0.72500(29/40)	0.37500(6/16)	0.04348(1/23)	0.17647(3/17)
Bo_TE_129833	0.00000(0/45)	0.82353(14/17)	0.04348(1/23)	0.15789(3/19)
Bo_TE_20422	0.16667(7/42)	0.31250(5/16)	0.90909(20/22)	0.65000(13/20)
Bo_TE_219357	0.40000(16/40)	1.00000(18/18)	0.80000(16/20)	0.84211(16/19)
Bo_TE_111045	0.20930(9/43)	0.16667(3/18)	0.85714(18/21)	0.00000(0/19)
Bo_TE_44008	0.48837(21/43)	0.17647(3/17)	0.57143(12/21)	0.90000(18/20)
Bo_TE_163654	0.75610(31/41)	0.36842(7/19)	1.00000(23/23)	0.88889(16/18)
Bo_TE_137299	0.89130(41/46)	0.76471(13/17)	0.00000(0/22)	0.30000(6/20)
Bo_TE_18725	0.72093(31/43)	0.80000(12/15)	1.00000(21/21)	0.33333(6/18)
Bo_TE_169847	0.38636(17/44)	0.88235(15/17)	0.95455(21/22)	0.82353(14/17)
Bo_TE_224741	0.13953(6/43)	0.00000(0/18)	0.28571(6/21)	0.84211(16/19)
Bo_TE_168668	0.69231(27/39)	1.00000(17/17)	0.31579(6/19)	0.20000(4/20)
Bo_TE_49726	0.00000(0/44)	0.12500(2/16)	0.71429(15/21)	0.47059(8/17)
Bo_TE_10230	0.58537(24/41)	0.50000(9/18)	0.36364(8/22)	0.00000(0/20)
Bo_TE_86142	0.15909(7/44)	0.00000(0/17)	0.28571(6/21)	0.78947(15/19)
Bo_TE_57918	0.57500(23/40)	0.75000(12/16)	0.52941(9/17)	0.05000(1/20)
Bo_TE_86710	0.95455(42/44)	0.29412(5/17)	0.63636(14/22)	0.75000(15/20)
Bo_TE_157953	0.00000(0/46)	0.00000(0/16)	0.45000(9/20)	0.84211(16/19)
Bo_TE_216520	0.11628(5/43)	0.22222(2/9)	0.00000(0/17)	0.68750(11/16)
Bo_TE_191483	0.67442(29/43)	0.00000(0/18)	0.00000(0/22)	0.00000(0/19)
Bo_TE_238371	0.51163(22/43)	0.23529(4/17)	0.00000(0/23)	0.00000(0/20)
Bo_TE_8469	0.44444(20/45)	0.11765(2/17)	0.36842(7/19)	0.68421(13/19)
Bo_TE_193434	0.65854(27/41)	0.41176(7/17)	0.80000(16/20)	0.94737(18/19)
Bo_TE_37469	0.71429(30/42)	0.29412(5/17)	0.40909(9/22)	0.00000(0/20)
Bo_TE_179858	0.07317(3/41)	0.66667(12/18)	0.68182(15/22)	0.75000(15/20)
Bo_TE_10798	0.88372(38/43)	0.75000(12/16)	0.81818(18/22)	0.05000(1/20)
Bo_TE_121207	0.70732(29/41)	0.55556(10/18)	0.08696(2/23)	0.25000(5/20)
Bo_TE_32800	0.52632(20/38)	0.52941(9/17)	0.00000(0/23)	0.05000(1/20)
Bo_TE_222493	0.86364(38/44)	0.43750(7/16)	0.00000(0/23)	0.50000(9/18)
Bo_TE_77680	0.00000(0/46)	0.50000(8/16)	0.08696(2/23)	0.00000(0/20)
Bo_TE_34676	0.02273(1/44)	0.31250(5/16)	0.60000(12/20)	0.83333(15/18)
Bo_TE_102060	0.15385(6/39)	0.53333(8/15)	0.76190(16/21)	0.65000(13/20)
Bo_TE_121243	0.00000(0/45)	0.00000(0/17)	0.28571(6/21)	0.66667(12/18)
Bo_TE_199220	0.21429(9/42)	0.47059(8/17)	0.95455(21/22)	1.00000(17/17)
Bo_TE_232773	0.97826(45/46)	1.00000(19/19)	0.75000(15/20)	0.31579(6/19)
Bo_TE_125879	0.72727(32/44)	0.82353(14/17)	0.09524(2/21)	0.05000(1/20)
Bo_TE_227287	0.77273(34/44)	1.00000(17/17)	0.33333(7/21)	0.95000(19/20)
Bo_TE_222619	0.02326(1/43)	0.00000(0/18)	0.00000(0/22)	0.65000(13/20)
Bo_TE_88484	0.19512(8/41)	0.88889(16/18)	0.45000(9/20)	0.50000(8/16)
Bo_TE_103748	0.93333(42/45)	0.70588(12/17)	0.33333(7/21)	0.50000(10/20)
Bo_TE_92849	0.15556(7/45)	0.22222(4/18)	0.10526(2/19)	0.63158(12/19)
Bo_TE_15432	0.74419(32/43)	0.06250(1/16)	0.00000(0/23)	0.55556(10/18)
Bo_TE_4773	0.60465(26/43)	0.31250(5/16)	0.10000(2/20)	0.11765(2/17)
Bo_TE_111990	0.09756(4/41)	0.00000(0/18)	0.76190(16/21)	0.38889(7/18)
Bo_TE_131108	0.39535(17/43)	0.94118(16/17)	0.45455(10/22)	0.50000(9/18)
Bo_TE_182784	0.65854(27/41)	0.15385(2/13)	0.13636(3/22)	0.05263(1/19)
Bo_TE_230693	0.68293(28/41)	1.00000(13/13)	1.00000(23/23)	0.22222(4/18)
Bo_TE_10588	0.09302(4/43)	0.56250(9/16)	0.00000(0/20)	0.50000(10/20)
Bo_TE_17497	0.00000(0/43)	0.77778(14/18)	0.86364(19/22)	0.61111(11/18)
Bo_TE_169400	0.88889(40/45)	0.11765(2/17)	0.00000(0/23)	0.10000(2/20)
Bo_TE_95048	0.79545(35/44)	0.81250(13/16)	0.13043(3/23)	0.00000(0/20)

Bo_TE_232714	0.97727(43/44)	1.00000(19/19)	0.65217(15/23)	0.23529(4/17)
Bo_TE_59152	1.00000(46/46)	0.33333(5/15)	0.38095(8/21)	0.10526(2/19)
Bo_TE_87744	0.82222(37/45)	0.11765(2/17)	0.15000(3/20)	0.23529(4/17)
Bo_TE_96803	0.32500(13/40)	0.47059(8/17)	0.19048(4/21)	1.00000(18/18)
Bo_TE_9671	0.56818(25/44)	0.27778(5/18)	0.27273(6/22)	0.95000(19/20)
Bo_TE_105048	0.70000(28/40)	1.00000(14/14)	0.00000(0/21)	0.10000(2/20)
Bo_TE_149463	0.09302(4/43)	0.35294(6/17)	0.75000(15/20)	0.00000(0/20)
Bo_TE_49928	0.04545(2/44)	0.81250(13/16)	0.86957(20/23)	0.50000(10/20)
Bo_TE_191062	0.67442(29/43)	0.36842(7/19)	0.15000(3/20)	0.26316(5/19)
Bo_TE_156375	0.11628(5/43)	0.00000(0/18)	0.61905(13/21)	0.65000(13/20)
Bo_TE_173609	0.80952(34/42)	0.44444(8/18)	0.31818(7/22)	0.18750(3/16)
Bo_TE_49979	0.31818(14/44)	0.05556(1/18)	0.00000(0/22)	0.73684(14/19)
Bo_TE_150862	0.86047(37/43)	0.42105(8/19)	0.23810(5/21)	0.42105(8/19)
Bo_TE_34946	0.77273(34/44)	1.00000(18/18)	0.66667(14/21)	0.50000(9/18)
Bo_TE_94734	0.70732(29/41)	0.27778(5/18)	0.05263(1/19)	0.66667(10/15)
Bo_TE_171927	0.81818(36/44)	0.23529(4/17)	0.09091(2/22)	0.42105(8/19)
Bo_TE_210568	0.11628(5/43)	0.05882(1/17)	0.52381(11/21)	0.70000(14/20)
Bo_TE_223095	0.48889(22/45)	0.41176(7/17)	0.21739(5/23)	0.90000(18/20)
Bo_TE_180792	0.00000(0/45)	0.55556(10/18)	0.00000(0/21)	0.18750(3/16)
Bo_TE_234537	0.79070(34/43)	0.31250(5/16)	0.00000(0/23)	0.00000(0/19)
Bo_TE_156401	0.25000(10/40)	0.94118(16/17)	1.00000(23/23)	0.84211(16/19)
Bo_TE_143820	0.14815(4/27)	0.08333(1/12)	1.00000(21/21)	0.31579(6/19)
Bo_TE_80085	0.23913(11/46)	0.77778(14/18)	0.00000(0/21)	0.00000(0/20)
Bo_TE_43652	0.16279(7/43)	0.35294(6/17)	1.00000(21/21)	0.84211(16/19)
Bo_TE_1003	0.52273(23/44)	0.64286(9/14)	0.23810(5/21)	0.75000(15/20)
Bo_TE_181006	0.30233(13/43)	0.23529(4/17)	0.65000(13/20)	0.00000(0/20)
Bo_TE_45860	0.43182(19/44)	0.77778(7/9)	1.00000(19/19)	1.00000(14/14)
Bo_TE_66069	0.14286(6/42)	0.05882(1/17)	0.86364(19/22)	0.27778(5/18)
Bo_TE_135735	0.06667(3/45)	0.50000(8/16)	0.00000(0/23)	0.20000(4/20)
Bo_TE_182493	0.95455(42/44)	0.64706(11/17)	0.42857(9/21)	0.68421(13/19)
Bo_TE_172875	0.50000(21/42)	0.00000(0/19)	0.50000(10/20)	0.61111(11/18)
Bo_TE_95999	0.22500(9/40)	0.40000(6/15)	1.00000(15/15)	1.00000(19/19)
Bo_TE_153825	0.80000(24/30)	0.88889(16/18)	0.35000(7/20)	0.05882(1/17)
Bo_TE_12995	0.86364(38/44)	0.05882(1/17)	0.19048(4/21)	0.36842(7/19)
Bo_TE_221930	0.34884(15/43)	0.73684(14/19)	0.18182(4/22)	0.00000(0/20)
Bo_TE_120885	0.41026(16/39)	0.35294(6/17)	0.68421(13/19)	0.00000(0/20)
Bo_TE_154251	0.82609(38/46)	0.62500(10/16)	0.00000(0/23)	0.11111(2/18)
Bo_TE_224479	0.00000(0/45)	0.16667(3/18)	0.14286(3/21)	0.73684(14/19)
Bo_TE_170855	0.23256(10/43)	0.29412(5/17)	1.00000(23/23)	0.45000(9/20)
Bo_TE_215522	0.79545(35/44)	0.50000(9/18)	0.50000(10/20)	0.00000(0/20)
Bo_TE_7840	0.59459(22/37)	0.12500(2/16)	0.64286(9/14)	0.05000(1/20)
Bo_TE_55809	0.50000(21/42)	0.29412(5/17)	0.81818(18/22)	0.29412(5/17)
Bo_TE_31242	0.00000(0/46)	0.55556(10/18)	0.00000(0/22)	0.25000(5/20)
Bo_TE_136897	0.51429(18/35)	0.87500(14/16)	0.31579(6/19)	0.33333(6/18)
Bo_TE_88849	0.80000(32/40)	0.35294(6/17)	1.00000(21/21)	0.55556(10/18)
Bo_TE_226887	0.88372(38/43)	0.43750(7/16)	0.00000(0/23)	0.15000(3/20)
Bo_TE_37392	0.02326(1/43)	0.76471(13/17)	0.14286(3/21)	0.11765(2/17)
Bo_TE_32559	0.27273(12/44)	0.05882(1/17)	0.52381(11/21)	0.75000(15/20)
Bo_TE_121779	0.13333(6/45)	0.00000(0/18)	0.04348(1/23)	0.85000(17/20)
Bo_TE_143184	0.91304(42/46)	0.84211(16/19)	0.23810(5/21)	0.15000(3/20)
Bo_TE_99917	0.73333(33/45)	0.23529(4/17)	0.95455(21/22)	1.00000(19/19)
Bo_TE_205115	0.20000(9/45)	0.70588(12/17)	0.21429(3/14)	0.33333(1/3)
Bo_TE_25109	0.78049(32/41)	0.29412(5/17)	0.00000(0/22)	0.00000(0/20)
Bo_TE_163465	0.25000(11/44)	0.05263(1/19)	0.71429(15/21)	0.00000(0/19)
Bo_TE_124873	0.00000(0/45)	0.00000(0/17)	0.00000(0/23)	0.55556(10/18)
Bo_TE_154247	0.88372(38/43)	0.73333(11/15)	0.00000(0/23)	0.10526(2/19)

Bo_TE_111898	0.32558(14/43)	0.85714(12/14)	0.20000(4/20)	0.89474(17/19)
Bo_TE_77526	0.92857(39/42)	0.20000(3/15)	0.59091(13/22)	0.10526(2/19)
Bo_TE_190651	0.88889(40/45)	0.11765(2/17)	0.68182(15/22)	0.10526(2/19)
Bo_TE_154729	0.00000(0/45)	0.00000(0/18)	0.75000(15/20)	0.61111(11/18)
Bo_TE_224620	0.64865(24/37)	0.05556(1/18)	0.00000(0/22)	0.05263(1/19)
Bo_TE_179816	0.04348(2/46)	0.52941(9/17)	0.00000(0/23)	0.00000(0/19)
Bo_TE_13533	0.84091(37/44)	0.00000(0/18)	0.00000(0/22)	0.00000(0/18)
Bo_TE_206533	0.70732(29/41)	0.05556(1/18)	0.50000(10/20)	0.12500(1/8)
Bo_TE_204869	0.00000(0/46)	0.15789(3/19)	0.34783(8/23)	0.84211(16/19)
Bo_TE_216394	0.00000(0/44)	0.17647(3/17)	0.61905(13/21)	0.38889(7/18)
Bo_TE_36686	0.11628(5/43)	0.72222(13/18)	0.71429(15/21)	0.42105(8/19)
Bo_TE_207182	0.39024(16/41)	0.64706(11/17)	0.63158(12/19)	0.10526(2/19)
Bo_TE_35777	0.88372(38/43)	0.44444(8/18)	0.95238(20/21)	0.77778(14/18)
Bo_TE_145610	0.00000(0/46)	0.61111(11/18)	0.00000(0/23)	0.00000(0/20)
Bo_TE_40498	0.40476(17/42)	0.93750(15/16)	0.95652(22/23)	1.00000(20/20)
Bo_TE_148100	0.35714(15/42)	0.10526(2/19)	0.36364(8/22)	1.00000(20/20)
Bo_TE_41961	0.16279(7/43)	0.31250(5/16)	0.61905(13/21)	0.00000(0/20)
Bo_TE_151125	0.86364(38/44)	0.33333(6/18)	0.14286(3/21)	0.73684(14/19)
Bo_TE_107912	0.02222(1/45)	0.82353(14/17)	0.47368(9/19)	0.05263(1/19)
Bo_TE_97982	0.97619(41/42)	0.88235(15/17)	0.38095(8/21)	0.21053(4/19)
Bo_TE_17734	0.57895(22/38)	0.64706(11/17)	0.00000(0/22)	0.57895(11/19)
Bo_TE_165565	0.86047(37/43)	0.31250(5/16)	0.50000(11/22)	0.35000(7/20)
Bo_TE_203216	0.32500(13/40)	0.17647(3/17)	0.63158(12/19)	0.90000(18/20)
Bo_TE_177864	0.72500(29/40)	0.23529(4/17)	0.75000(15/20)	0.47368(9/19)
Bo_TE_25315	0.76190(32/42)	0.31250(5/16)	0.30000(6/20)	0.10526(2/19)
Bo_TE_235872	0.15909(7/44)	0.11111(2/18)	0.90909(20/22)	0.94737(18/19)
Bo_TE_114359	0.23256(10/43)	0.26316(5/19)	0.90476(19/21)	0.72727(8/11)
Bo_TE_6965	0.32558(14/43)	0.00000(0/16)	0.04348(1/23)	0.75000(15/20)
Bo_TE_179724	0.55814(24/43)	0.26667(4/15)	0.18182(4/22)	0.00000(0/20)
Bo_TE_181608	0.00000(0/44)	0.00000(0/19)	0.50000(10/20)	0.00000(0/20)
Bo_TE_69412	0.97778(44/45)	1.00000(18/18)	0.80952(17/21)	0.31579(6/19)
Bo_TE_97618	0.73333(33/45)	0.00000(0/16)	0.00000(0/22)	0.05882(1/17)
Bo_TE_235861	0.93333(42/45)	0.44444(8/18)	0.86957(20/23)	0.94737(18/19)
Bo_TE_89246	0.68293(28/41)	0.23529(4/17)	0.78947(15/19)	0.22222(4/18)
Bo_TE_16852	0.82500(33/40)	0.56250(9/16)	0.23810(5/21)	0.44444(8/18)
Bo_TE_51078	0.00000(0/45)	0.72222(13/18)	0.00000(0/23)	0.05000(1/20)
Bo_TE_51152	0.00000(0/45)	0.00000(0/19)	0.28571(6/21)	0.61111(11/18)
Bo_TE_169123	0.00000(0/46)	0.00000(0/19)	0.00000(0/22)	0.64706(11/17)
Bo_TE_141758	0.02273(1/44)	0.00000(0/19)	0.76190(16/21)	0.15000(3/20)
Bo_TE_140422	0.76923(30/39)	0.52632(10/19)	0.38095(8/21)	0.00000(0/20)
Bo_TE_55748	0.97727(43/44)	1.00000(18/18)	0.90909(20/22)	0.20000(4/20)
Bo_TE_91645	0.04651(2/43)	0.23529(4/17)	0.65000(13/20)	0.15000(3/20)
Bo_TE_159189	0.59091(26/44)	0.06667(1/15)	0.00000(0/21)	0.00000(0/19)
Bo_TE_44804	0.06667(3/45)	0.72222(13/18)	0.85000(17/20)	0.00000(0/20)
Bo_TE_239789	0.40476(17/42)	0.62500(10/16)	0.86364(19/22)	0.00000(0/18)
Bo_TE_218371	0.21739(10/46)	0.05882(1/17)	0.38095(8/21)	0.80000(16/20)
Bo_TE_143827	0.92857(39/42)	0.68750(11/16)	0.36842(7/19)	0.31579(6/19)
Bo_TE_145853	1.00000(43/43)	0.83333(15/18)	0.71429(15/21)	0.36842(7/19)
Bo_TE_144649	0.00000(0/46)	0.52941(9/17)	0.23810(5/21)	0.20000(4/20)
Bo_TE_158207	0.04545(2/44)	0.11765(2/17)	0.54545(12/22)	0.36842(7/19)
Bo_TE_220404	0.00000(0/45)	0.10526(2/19)	0.45455(10/22)	0.78947(15/19)
Bo_TE_27028	0.76744(33/43)	0.43750(7/16)	0.47619(10/21)	0.95000(19/20)
Bo_TE_194551	0.88372(38/43)	0.23529(4/17)	0.00000(0/22)	0.47368(9/19)
Bo_TE_111600	0.78571(33/42)	0.76471(13/17)	0.23810(5/21)	0.88889(16/18)
Bo_TE_191455	0.25641(10/39)	1.00000(17/17)	1.00000(22/22)	1.00000(20/20)
Bo_TE_85595	0.02222(1/45)	0.26316(5/19)	0.77273(17/22)	0.60000(12/20)

Bo_TE_21464	0.19512(8/41)	0.72222(13/18)	0.95455(21/22)	0.95000(19/20)
Bo_TE_13517	0.76744(33/43)	0.00000(0/19)	0.00000(0/22)	0.00000(0/19)
Bo_TE_41239	0.08696(4/46)	0.70588(12/17)	0.35000(7/20)	0.68421(13/19)
Bo_TE_36496	0.02222(1/45)	0.58824(10/17)	0.47059(8/17)	0.72222(13/18)
Bo_TE_131169	0.70000(28/40)	1.00000(17/17)	0.18182(4/22)	0.57895(11/19)
Bo_TE_124934	0.61538(24/39)	0.29412(5/17)	0.42105(8/19)	0.11111(2/18)
Bo_TE_73252	0.51282(20/39)	0.16667(3/18)	0.80000(16/20)	1.00000(17/17)
Bo_TE_219093	0.28205(11/39)	0.94118(16/17)	0.72727(16/22)	0.78947(15/19)
Bo_TE_156399	0.28205(11/39)	0.93333(14/15)	1.00000(21/21)	0.81250(13/16)
Bo_TE_230335	0.46512(20/43)	0.00000(0/15)	0.00000(0/23)	0.50000(9/18)
Bo_TE_157760	0.00000(0/46)	0.00000(0/19)	0.45000(9/20)	0.70000(14/20)
Bo_TE_4174	0.42857(18/42)	0.87500(14/16)	0.19048(4/21)	0.00000(0/19)
Bo_TE_231243	0.52500(21/40)	0.68750(11/16)	0.14286(3/21)	0.21053(4/19)
Bo_TE_148775	0.70732(29/41)	0.88235(15/17)	0.00000(0/21)	0.00000(0/17)
Bo_TE_152273	0.20930(9/43)	0.88889(16/18)	0.52632(10/19)	0.89474(17/19)
Bo_TE_191749	0.28571(12/42)	0.33333(6/18)	0.95455(21/22)	0.85000(17/20)
Bo_TE_143566	0.67568(25/37)	0.66667(12/18)	1.00000(23/23)	0.35000(7/20)
Bo_TE_70660	0.00000(0/45)	0.66667(12/18)	0.00000(0/23)	0.00000(0/20)
Bo_TE_214332	0.11905(5/42)	0.11765(2/17)	0.61905(13/21)	0.44444(8/18)
Bo_TE_225361	0.73810(31/42)	1.00000(19/19)	0.47826(11/23)	1.00000(20/20)
Bo_TE_227457	0.68293(28/41)	0.76471(13/17)	0.65000(13/20)	0.21053(4/19)
Bo_TE_141466	0.11364(5/44)	0.68750(11/16)	0.95238(20/21)	0.78947(15/19)
Bo_TE_152282	0.83721(36/43)	0.05556(1/18)	0.47619(10/21)	0.10000(2/20)
Bo_TE_50582	0.34884(15/43)	0.00000(0/17)	0.08696(2/23)	0.85000(17/20)
Bo_TE_5009	0.33333(13/39)	0.86667(13/15)	0.42857(9/21)	0.94444(17/18)
Bo_TE_183728	0.07143(3/42)	0.16667(3/18)	0.55000(11/20)	0.80000(16/20)
Bo_TE_95637	0.09091(4/44)	0.33333(6/18)	0.19048(4/21)	0.68421(13/19)
Bo_TE_53592	1.00000(44/44)	1.00000(19/19)	0.26316(5/19)	0.18182(2/11)
Bo_TE_168570	0.36364(16/44)	0.93750(15/16)	0.28571(6/21)	0.20000(4/20)
Bo_TE_53604	0.95122(39/41)	1.00000(16/16)	0.25000(5/20)	0.10526(2/19)
Bo_TE_101332	0.97619(41/42)	0.88235(15/17)	0.75000(15/20)	0.21053(4/19)
Bo_TE_58312	0.38095(16/42)	0.88235(15/17)	0.59091(13/22)	0.75000(15/20)
Bo_TE_217954	0.55814(24/43)	0.05882(1/17)	0.69565(16/23)	0.85000(17/20)
Bo_TE_69580	0.38462(15/39)	0.66667(12/18)	0.15789(3/19)	0.00000(0/19)
Bo_TE_105261	0.00000(0/45)	0.00000(0/18)	0.80000(16/20)	0.21053(4/19)
Bo_TE_154253	0.92857(39/42)	0.18750(3/16)	1.00000(23/23)	1.00000(19/19)
Bo_TE_205294	0.00000(0/45)	0.50000(9/18)	0.00000(0/14)	0.00000(0/2)
Bo_TE_136061	0.11364(5/44)	0.77778(14/18)	0.95455(21/22)	0.77778(14/18)
Bo_TE_161130	1.00000(43/43)	0.83333(15/18)	0.55000(11/20)	0.44444(8/18)
Bo_TE_182633	0.04545(2/44)	0.38889(7/18)	0.59091(13/22)	0.30000(6/20)
Bo_TE_191723	0.52381(22/42)	0.33333(6/18)	0.00000(0/23)	0.00000(0/20)
Bo_TE_13835	0.37209(16/43)	0.58824(10/17)	0.00000(0/22)	0.84211(16/19)
Bo_TE_228082	0.00000(0/44)	0.00000(0/19)	0.80000(16/20)	0.50000(10/20)
Bo_TE_91458	0.23256(10/43)	0.42105(8/19)	0.84211(16/19)	0.42105(8/19)
Bo_TE_92878	0.34091(15/44)	0.11765(2/17)	0.33333(6/18)	0.85000(17/20)
Bo_TE_149189	0.95455(42/44)	0.88889(16/18)	0.21053(4/19)	0.52632(10/19)
Bo_TE_57488	0.17949(7/39)	0.82353(14/17)	0.86364(19/22)	0.10526(2/19)
Bo_TE_209773	0.15385(6/39)	0.46667(7/15)	0.15000(3/20)	0.72222(13/18)
Bo_TE_82577	0.30233(13/43)	0.52941(9/17)	0.00000(0/23)	0.00000(0/20)
Bo_TE_201704	0.37500(15/40)	0.76471(13/17)	0.36842(7/19)	0.94737(18/19)
Bo_TE_62255	0.61905(26/42)	0.88235(15/17)	0.00000(0/20)	0.05000(1/20)
Bo_TE_55750	0.04878(2/41)	0.00000(0/17)	0.09091(2/22)	0.80000(16/20)
Bo_TE_112251	0.64103(25/39)	0.42105(8/19)	1.00000(23/23)	0.90000(18/20)
Bo_TE_214473	0.81395(35/43)	0.87500(14/16)	0.47619(10/21)	0.15000(3/20)
Bo_TE_176894	0.97826(45/46)	1.00000(18/18)	0.28571(6/21)	1.00000(19/19)
Bo_TE_10218	0.61905(26/42)	0.41176(7/17)	0.00000(0/14)	0.00000(0/19)

Bo_TE_172880	0.48780(20/41)	1.00000(18/18)	0.57895(11/19)	0.36842(7/19)
Bo_TE_198974	0.15909(7/44)	0.73333(11/15)	1.00000(22/22)	1.00000(18/18)
Bo_TE_198337	0.62791(27/43)	0.33333(6/18)	0.61905(13/21)	0.00000(0/19)
Bo_TE_129121	0.06977(3/43)	0.73684(14/19)	0.13636(3/22)	0.00000(0/20)
Bo_TE_80163	0.00000(0/45)	0.58824(10/17)	0.84211(16/19)	0.57895(11/19)
Bo_TE_78423	0.02273(1/44)	0.05263(1/19)	0.00000(0/22)	0.55000(11/20)
Bo_TE_236279	0.02326(1/43)	0.00000(0/18)	0.52381(11/21)	0.89474(17/19)
Bo_TE_211572	0.29545(13/44)	0.12500(2/16)	1.00000(21/21)	0.80000(16/20)
Bo_TE_43970	0.14286(6/42)	0.27778(5/18)	0.95652(22/23)	0.88889(16/18)
Bo_TE_53970	0.68293(28/41)	0.94118(16/17)	0.47619(10/21)	0.11765(2/17)
Bo_TE_82898	0.20000(9/45)	0.18750(3/16)	1.00000(21/21)	0.89474(17/19)
Bo_TE_91699	0.12821(5/39)	0.31250(5/16)	0.66667(14/21)	0.00000(0/20)
Bo_TE_193447	0.33333(13/39)	0.58824(10/17)	0.19048(4/21)	0.05000(1/20)
Bo_TE_90943	0.40909(18/44)	0.72222(13/18)	0.38095(8/21)	0.00000(0/18)
Bo_TE_27161	0.10870(5/46)	0.00000(0/18)	0.73913(17/23)	0.44444(8/18)
Bo_TE_119754	0.65116(28/43)	0.00000(0/18)	0.00000(0/22)	0.00000(0/19)
Bo_TE_201793	0.17949(7/39)	0.47059(8/17)	0.75000(15/20)	0.15789(3/19)
Bo_TE_33599	0.22727(10/44)	0.62500(10/16)	0.23810(5/21)	0.77778(14/18)
Bo_TE_241105	0.00000(0/45)	0.00000(0/19)	0.52381(11/21)	0.00000(0/20)
Bo_TE_101792	0.81818(36/44)	0.50000(9/18)	0.57143(12/21)	1.00000(20/20)
Bo_TE_181071	0.63158(24/38)	0.82353(14/17)	0.27273(6/22)	0.65000(13/20)
Bo_TE_71279	0.90476(38/42)	0.13333(2/15)	0.23810(5/21)	0.47059(8/17)
Bo_TE_234516	0.02222(1/45)	0.61111(11/18)	0.60000(12/20)	0.50000(9/18)
Bo_TE_8022	0.06977(3/43)	0.00000(0/17)	0.55556(10/18)	0.60000(12/20)
Bo_TE_130768	0.55000(22/40)	0.38889(7/18)	0.09091(2/22)	0.68421(13/19)
Bo_TE_16844	0.41026(16/39)	0.23529(4/17)	0.84211(16/19)	0.33333(6/18)
Bo_TE_111107	0.17778(8/45)	0.18750(3/16)	0.69565(16/23)	0.85000(17/20)
Bo_TE_235647	0.28571(12/42)	0.52941(9/17)	0.00000(0/23)	0.10000(2/20)
Bo_TE_203699	0.37500(15/40)	0.68750(11/16)	0.16667(3/18)	0.05000(1/20)
Bo_TE_94683	0.68293(28/41)	0.18750(3/16)	0.09091(2/22)	0.72222(13/18)
Bo_TE_232548	1.00000(46/46)	1.00000(18/18)	0.63636(14/22)	0.44444(8/18)
Bo_TE_100250	0.17073(7/41)	0.00000(0/17)	0.77273(17/22)	0.50000(9/18)
Bo_TE_142831	0.44444(20/45)	0.94118(16/17)	0.86364(19/22)	0.30000(6/20)
Bo_TE_211462	0.18182(8/44)	0.10526(2/19)	0.81818(18/22)	0.11765(2/17)
Bo_TE_188651	0.08696(4/46)	0.50000(9/18)	0.04545(1/22)	0.70000(14/20)
Bo_TE_142844	0.42857(18/42)	0.88235(15/17)	0.90476(19/21)	0.15000(3/20)
Bo_TE_165046	0.50000(21/42)	0.06667(1/15)	0.00000(0/22)	0.00000(0/20)
Bo_TE_56745	0.84091(37/44)	0.56250(9/16)	0.20000(4/20)	0.05882(1/17)
Bo_TE_105301	0.00000(0/44)	0.27778(5/18)	0.00000(0/23)	0.60000(12/20)
Bo_TE_22462	0.02326(1/43)	0.47059(8/17)	0.50000(10/20)	0.57895(11/19)
Bo_TE_39994	0.42857(18/42)	0.33333(6/18)	0.61905(13/21)	0.89474(17/19)
Bo_TE_10788	0.85366(35/41)	0.26667(4/15)	0.04348(1/23)	0.05263(1/19)
Bo_TE_199667	0.09091(4/44)	0.05263(1/19)	0.00000(0/22)	0.72222(13/18)
Bo_TE_117203	0.02273(1/44)	0.63158(12/19)	0.66667(14/21)	0.47368(9/19)
Bo_TE_12532	0.17778(8/45)	0.88889(16/18)	0.52381(11/21)	0.89474(17/19)
Bo_TE_134526	0.86047(37/43)	0.82353(14/17)	0.40000(8/20)	0.16667(3/18)
Bo_TE_80728	0.65116(28/43)	0.88235(15/17)	0.09524(2/21)	0.05263(1/19)
Bo_TE_226730	0.97778(44/45)	1.00000(19/19)	0.23810(5/21)	0.95000(19/20)
Bo_TE_192139	0.42222(19/45)	0.23529(4/17)	0.27273(6/22)	0.90000(18/20)
Bo_TE_106161	0.90909(40/44)	0.16667(3/18)	1.00000(23/23)	1.00000(20/20)
Bo_TE_151008	0.25581(11/43)	0.07143(1/14)	0.76190(16/21)	0.00000(0/20)
Bo_TE_101387	1.00000(44/44)	0.35294(6/17)	0.75000(15/20)	0.36842(7/19)
Bo_TE_76844	0.14634(6/41)	0.17647(3/17)	1.00000(23/23)	0.10526(2/19)
Bo_TE_85480	0.84615(33/39)	0.00000(0/17)	0.00000(0/21)	0.46154(6/13)
Bo_TE_119521	0.18182(8/44)	0.00000(0/17)	0.61905(13/21)	0.15000(3/20)
Bo_TE_116841	0.16667(7/42)	0.05556(1/18)	0.04762(1/21)	0.55556(10/18)

Bo_TE_190622	0.10870(5/46)	0.66667(4/6)	0.33333(7/21)	0.89474(17/19)
Bo_TE_67864	0.10000(4/40)	0.35294(6/17)	0.42857(9/21)	0.80000(16/20)
Bo_TE_37385	0.65000(26/40)	0.05263(1/19)	0.00000(0/23)	0.00000(0/20)
Bo_TE_210017	0.95238(40/42)	0.35294(6/17)	1.00000(23/23)	1.00000(18/18)
Bo_TE_151237	1.00000(44/44)	0.33333(6/18)	0.42105(8/19)	0.52632(10/19)
Bo_TE_82310	0.71429(30/42)	0.38889(7/18)	0.13043(3/23)	0.45000(9/20)
Bo_TE_54803	0.02500(1/40)	0.00000(0/15)	0.86364(19/22)	0.50000(6/12)
Bo_TE_194850	0.51282(20/39)	0.23529(4/17)	0.18182(4/22)	0.00000(0/19)
Bo_TE_236478	0.28889(13/45)	0.11765(2/17)	0.54545(12/22)	0.00000(0/19)
Bo_TE_169776	0.24390(10/41)	0.88235(15/17)	1.00000(21/21)	1.00000(19/19)
Bo_TE_117780	0.00000(0/45)	0.00000(0/18)	0.28571(6/21)	0.83333(15/18)
Bo_TE_141513	0.81818(36/44)	0.05882(1/17)	0.05000(1/20)	0.21053(4/19)
Bo_TE_60973	0.61538(24/39)	0.64706(11/17)	0.13636(3/22)	0.72222(13/18)
Bo_TE_220072	0.13953(6/43)	0.77778(14/18)	0.15000(3/20)	0.25000(4/16)
Bo_TE_42332	0.63415(26/41)	0.88889(16/18)	0.05000(1/20)	0.05263(1/19)
Bo_TE_39278	0.75610(31/41)	0.64706(11/17)	0.11111(2/18)	0.00000(0/16)
Bo_TE_55877	0.00000(0/44)	0.47059(8/17)	0.85714(18/21)	0.35000(7/20)
Bo_TE_226451	0.31707(13/41)	0.00000(0/18)	0.30000(6/20)	0.55000(11/20)
Bo_TE_141260	0.13953(6/43)	0.70588(12/17)	0.85000(17/20)	0.33333(6/18)
Bo_TE_86882	0.16279(7/43)	0.41176(7/17)	0.73913(17/23)	0.60000(12/20)
Bo_TE_239665	0.79545(35/44)	0.46667(7/15)	0.00000(0/20)	0.05263(1/19)
Bo_TE_100887	0.11364(5/44)	0.38889(7/18)	0.68182(15/22)	0.00000(0/20)
Bo_TE_130340	0.34884(15/43)	0.05556(1/18)	0.08696(2/23)	0.60000(12/20)
Bo_TE_144570	0.87805(36/41)	0.88235(15/17)	0.00000(0/23)	0.83333(15/18)
Bo_TE_158989	0.09091(4/44)	0.33333(6/18)	0.00000(0/22)	0.63158(12/19)
Bo_TE_37211	0.84444(38/45)	0.35294(6/17)	1.00000(19/19)	0.95000(19/20)
Bo_TE_159634	0.02326(1/43)	0.29412(5/17)	0.10526(2/19)	0.88235(15/17)
Bo_TE_7697	0.06977(3/43)	0.00000(0/16)	0.36364(8/22)	0.52632(10/19)
Bo_TE_80190	0.00000(0/45)	0.47059(8/17)	0.83333(15/18)	0.56250(9/16)
Bo_TE_208596	0.39535(17/43)	0.62500(10/16)	0.54545(12/22)	1.00000(20/20)
Bo_TE_129245	0.17500(7/40)	0.22222(4/18)	0.70000(14/20)	0.73684(14/19)
Bo_TE_72369	0.86667(39/45)	0.83333(15/18)	0.10000(2/20)	0.05263(1/19)
Bo_TE_135217	0.15556(7/45)	0.55556(10/18)	0.45000(9/20)	0.00000(0/20)
Bo_TE_228008	1.00000(46/46)	1.00000(18/18)	0.20000(4/20)	0.52632(10/19)
Bo_TE_138331	1.00000(44/44)	0.42105(8/19)	1.00000(23/23)	0.47368(9/19)
Bo_TE_88817	0.62791(27/43)	0.56250(9/16)	0.27273(6/22)	0.95000(19/20)
Bo_TE_226386	0.42105(16/38)	0.21053(4/19)	0.38095(8/21)	0.95000(19/20)
Bo_TE_24969	0.67500(27/40)	0.00000(0/17)	0.00000(0/23)	0.78947(15/19)
Bo_TE_111160	0.15385(6/39)	0.25000(4/16)	0.80952(17/21)	0.72222(13/18)
Bo_TE_1544	0.65000(26/40)	0.37500(6/16)	0.04545(1/22)	0.15000(3/20)
Bo_TE_137799	0.58974(23/39)	0.35714(5/14)	0.30435(7/23)	0.95000(19/20)
Bo_TE_52876	0.82927(34/41)	0.15789(3/19)	1.00000(22/22)	0.90000(18/20)
Bo_TE_126135	0.11628(5/43)	0.56250(9/16)	0.00000(0/22)	0.00000(0/20)
Bo_TE_226079	0.04545(2/44)	0.66667(12/18)	0.33333(7/21)	0.00000(0/20)
Bo_TE_58559	0.34146(14/41)	0.94444(17/18)	0.91304(21/23)	0.65000(13/20)
Bo_TE_54613	0.55000(22/40)	0.33333(5/15)	0.00000(0/22)	0.38889(7/18)
Bo_TE_132317	0.24444(11/45)	0.05556(1/18)	0.54545(12/22)	0.75000(15/20)
Bo_TE_148711	0.39474(15/38)	0.52941(9/17)	1.00000(23/23)	1.00000(18/18)
Bo_TE_126514	0.47500(19/40)	0.68750(11/16)	0.69565(16/23)	1.00000(18/18)
Bo_TE_154267	0.93023(40/43)	0.23529(4/17)	1.00000(23/23)	1.00000(20/20)
Bo_TE_141261	0.15556(7/45)	0.88235(15/17)	0.90000(18/20)	0.78947(15/19)
Bo_TE_136122	0.00000(0/44)	0.00000(0/19)	0.22727(5/22)	0.60000(12/20)
Bo_TE_90276	0.51613(16/31)	0.17647(3/17)	1.00000(21/21)	1.00000(6/6)
Bo_TE_119117	0.70000(28/40)	0.18750(3/16)	0.09091(2/22)	0.10526(2/19)
Bo_TE_46706	0.07317(3/41)	0.81250(13/16)	0.00000(0/21)	0.33333(6/18)
Bo_TE_46948	0.35714(15/42)	0.64706(11/17)	0.20000(4/20)	0.05000(1/20)

Bo_TE_155752	0.60000(24/40)	0.29412(5/17)	0.04762(1/21)	0.10000(2/20)
Bo_TE_92994	0.47727(21/44)	0.17647(3/17)	0.95455(21/22)	0.89474(17/19)
Bo_TE_43269	0.02174(1/46)	0.50000(8/16)	0.00000(0/22)	0.00000(0/20)
Bo_TE_95651	0.00000(0/44)	0.66667(12/18)	0.04545(1/22)	0.16667(3/18)
Bo_TE_43349	0.95238(40/42)	0.44444(8/18)	1.00000(20/20)	1.00000(18/18)
Bo_TE_81607	0.62222(28/45)	0.00000(0/17)	0.63636(14/22)	0.70000(14/20)
Bo_TE_127130	0.08889(4/45)	0.26667(4/15)	0.60000(12/20)	0.05263(1/19)
Bo_TE_236943	0.51111(23/45)	0.05882(1/17)	0.00000(0/21)	0.20000(4/20)
Bo_TE_55271	0.00000(0/45)	0.21053(4/19)	0.00000(0/23)	0.66667(12/18)
Bo_TE_96599	0.71795(28/39)	0.05263(1/19)	0.57895(11/19)	0.29412(5/17)
Bo_TE_212127	0.40000(16/40)	0.50000(9/18)	0.78947(15/19)	0.25000(5/20)
Bo_TE_35797	0.46341(19/41)	0.05882(1/17)	0.61905(13/21)	0.57895(11/19)
Bo_TE_134064	0.73810(31/42)	1.00000(16/16)	0.40000(8/20)	0.25000(5/20)
Bo_TE_140631	0.50000(21/42)	0.05556(1/18)	0.00000(0/22)	0.46667(7/15)
Bo_TE_205021	0.00000(0/45)	0.66667(12/18)	0.40909(9/22)	0.05000(1/20)
Bo_TE_135095	0.00000(0/46)	0.00000(0/19)	0.52632(10/19)	0.63158(12/19)
Bo_TE_174050	0.70000(28/40)	0.77778(14/18)	0.42857(9/21)	0.21053(4/19)
Bo_TE_116703	0.02326(1/43)	0.05556(1/18)	0.80952(17/21)	0.64706(11/17)
Bo_TE_132411	0.93182(41/44)	0.12500(2/16)	0.82609(19/23)	0.16667(3/18)
Bo_TE_230161	0.95455(42/44)	1.00000(16/16)	1.00000(23/23)	0.42105(8/19)
Bo_TE_103304	0.00000(0/44)	0.00000(0/17)	0.31818(7/22)	0.70000(14/20)
Bo_TE_70014	0.33333(14/42)	0.06250(1/16)	0.78947(15/19)	0.30000(6/20)
Bo_TE_45044	0.72727(32/44)	0.66667(12/18)	0.22727(5/22)	0.89474(17/19)
Bo_TE_148096	0.59524(25/42)	0.88889(16/18)	0.59091(13/22)	0.00000(0/20)
Bo_TE_236667	1.00000(45/45)	0.53846(7/13)	0.22727(5/22)	1.00000(19/19)
Bo_TE_76882	0.88095(37/42)	0.77778(14/18)	0.00000(0/20)	0.80000(16/20)
Bo_TE_238483	0.57500(23/40)	0.11765(2/17)	0.61905(13/21)	0.90000(18/20)
Bo_TE_66296	0.48780(20/41)	0.75000(12/16)	0.13636(3/22)	0.17647(3/17)
Bo_TE_156746	0.44186(19/43)	0.87500(14/16)	0.17391(4/23)	0.44444(8/18)
Bo_TE_163609	0.80952(34/42)	0.83333(10/12)	0.42857(3/7)	0.27778(5/18)
Bo_TE_62746	0.38095(16/42)	0.82353(14/17)	0.00000(0/22)	0.05000(1/20)
Bo_TE_142370	0.22500(9/40)	0.70588(12/17)	0.78261(18/23)	1.00000(20/20)
Bo_TE_6951	0.26190(11/42)	0.68750(11/16)	0.20000(4/20)	0.00000(0/20)
Bo_TE_33325	0.95122(39/41)	0.50000(7/14)	0.52381(11/21)	0.44444(8/18)
Bo_TE_84209	0.48649(18/37)	0.53333(8/15)	0.68182(15/22)	1.00000(20/20)
Bo_TE_76053	0.17949(7/39)	0.50000(9/18)	0.00000(0/23)	0.00000(0/20)
Bo_TE_150412	0.04651(2/43)	0.22222(4/18)	0.38095(8/21)	0.57895(11/19)
Bo_TE_123170	0.47727(21/44)	0.64706(11/17)	0.25000(5/20)	0.05000(1/20)
Bo_TE_152141	0.73810(31/42)	0.26316(5/19)	0.19048(4/21)	0.00000(0/18)
Bo_TE_11485	0.28571(12/42)	0.73333(11/15)	1.00000(22/22)	0.83333(15/18)
Bo_TE_201459	0.08696(4/46)	0.72222(13/18)	0.31818(7/22)	0.10000(2/20)
Bo_TE_105622	0.65789(25/38)	0.87500(14/16)	0.66667(14/21)	0.16667(3/18)
Bo_TE_63602	0.04878(2/41)	0.70588(12/17)	0.10000(2/20)	0.00000(0/19)
Bo_TE_54102	0.34091(15/44)	0.05263(1/19)	0.75000(15/20)	0.45000(9/20)
Bo_TE_18406	0.53659(22/41)	0.94444(17/18)	0.80952(17/21)	0.15789(3/19)
Bo_TE_14473	0.00000(0/45)	0.00000(0/18)	0.52174(12/23)	0.00000(0/20)
Bo_TE_124240	0.79545(35/44)	0.27778(5/18)	0.20000(4/20)	0.15789(3/19)
Bo_TE_107944	0.04651(2/43)	0.17647(3/17)	0.50000(10/20)	0.63158(12/19)
Bo_TE_158107	0.81395(35/43)	0.31250(5/16)	0.20000(4/20)	0.26316(5/19)
Bo_TE_70030	0.31707(13/41)	0.05556(1/18)	0.80952(17/21)	0.21053(4/19)
Bo_TE_102142	1.00000(44/44)	0.94444(17/18)	0.36842(7/19)	0.52632(10/19)
Bo_TE_172014	0.25000(11/44)	0.00000(0/9)	0.68182(15/22)	0.05263(1/19)
Bo_TE_68846	0.84091(37/44)	0.33333(6/18)	0.36364(8/22)	0.47368(9/19)
Bo_TE_91656	0.06667(3/45)	0.27778(5/18)	0.65000(13/20)	0.15000(3/20)
Bo_TE_92602	0.86047(37/43)	0.41176(7/17)	0.00000(0/21)	0.05263(1/19)
Bo_TE_30266	0.64286(9/14)	0.60000(6/10)	0.13636(3/22)	1.00000(20/20)

Bo_TE_58393	0.97674(42/43)	0.47059(8/17)	0.63636(14/22)	0.25000(5/20)
Bo_TE_95992	0.23810(10/42)	0.33333(6/18)	0.72222(13/18)	0.95000(19/20)
Bo_TE_44268	0.13636(6/44)	0.64706(11/17)	0.18182(4/22)	0.05000(1/20)
Bo_TE_178920	0.00000(0/44)	0.00000(0/18)	0.50000(11/22)	0.26316(5/19)
Bo_TE_62479	0.00000(0/46)	0.00000(0/18)	0.80000(16/20)	0.00000(0/20)
Bo_TE_159262	0.53846(21/39)	0.68750(11/16)	0.59091(13/22)	0.06250(1/16)
Bo_TE_211589	0.62500(25/40)	0.17647(3/17)	0.30000(6/20)	0.70000(14/20)
Bo_TE_197693	0.60465(26/43)	0.11111(2/18)	0.00000(0/22)	0.00000(0/20)
Bo_TE_201342	0.45238(19/42)	0.41176(7/17)	0.77273(17/22)	0.94737(18/19)
Bo_TE_10708	0.95652(44/46)	0.70588(12/17)	0.23810(5/21)	0.68421(13/19)
Bo_TE_14376	0.08696(4/46)	0.11111(2/18)	0.54545(12/22)	0.00000(0/20)
Bo_TE_239797	0.52273(23/44)	0.21053(4/19)	0.09524(2/21)	0.73684(14/19)
Bo_TE_199482	0.64444(29/45)	0.35294(6/17)	0.85714(18/21)	0.44444(8/18)
Bo_TE_133298	0.32432(12/37)	0.10000(1/10)	0.90476(19/21)	0.33333(6/18)
Bo_TE_55661	0.32500(13/40)	0.33333(6/18)	0.78947(15/19)	0.00000(0/20)
Bo_TE_178922	0.02273(1/44)	0.00000(0/17)	0.50000(11/22)	0.21053(4/19)
Bo_TE_204937	0.21951(9/41)	0.12500(2/16)	0.34783(8/23)	0.85000(17/20)
Bo_TE_85772	0.00000(0/46)	0.27778(5/18)	0.13043(3/23)	0.50000(10/20)
Bo_TE_159231	0.43590(17/39)	0.75000(12/16)	1.00000(22/22)	1.00000(20/20)
Bo_TE_172970	0.21951(9/41)	0.56250(9/16)	0.00000(0/22)	0.66667(12/18)
Bo_TE_8288	0.26829(11/41)	0.47059(8/17)	0.86364(19/22)	0.47368(9/19)
Bo_TE_140245	0.17778(8/45)	0.50000(9/18)	0.00000(0/22)	0.00000(0/20)
Bo_TE_125452	0.47727(21/44)	0.88889(16/18)	0.35000(7/20)	0.20000(4/20)
Bo_TE_163358	0.86047(37/43)	0.27778(5/18)	1.00000(20/20)	1.00000(20/20)
Bo_TE_229890	0.31818(14/44)	0.25000(4/16)	0.57143(12/21)	0.84211(16/19)
Bo_TE_211530	1.00000(43/43)	1.00000(17/17)	0.18182(4/22)	1.00000(20/20)
Bo_TE_178209	0.31818(14/44)	0.27778(5/18)	1.00000(21/21)	0.94444(17/18)
Bo_TE_108953	0.51163(22/43)	0.00000(0/18)	0.04762(1/21)	0.21053(4/19)
Bo_TE_216096	0.25000(10/40)	0.12500(2/16)	0.15789(3/19)	0.68421(13/19)
Bo_TE_212057	0.79545(35/44)	0.84211(16/19)	0.20000(4/20)	0.68421(13/19)
Bo_TE_152127	0.40909(18/44)	1.00000(17/17)	0.82609(19/23)	0.95000(19/20)
Bo_TE_234522	0.00000(0/45)	0.36842(7/19)	0.71429(15/21)	0.10526(2/19)
Bo_TE_74275	0.11905(5/42)	0.00000(0/16)	0.60870(14/23)	0.68421(13/19)
Bo_TE_173936	0.28571(12/42)	0.35294(6/17)	0.75000(15/20)	0.84211(16/19)
Bo_TE_143734	0.81395(35/43)	0.66667(12/18)	0.80952(17/21)	0.27778(5/18)
Bo_TE_61375	0.17073(7/41)	0.22222(4/18)	0.90000(18/20)	0.52632(10/19)
Bo_TE_65314	0.11905(5/42)	0.58824(10/17)	0.00000(0/20)	0.10000(2/20)
Bo_TE_111491	1.00000(42/42)	0.43750(7/16)	1.00000(23/23)	0.66667(12/18)
Bo_TE_45300	0.39535(17/43)	0.62500(10/16)	0.14286(3/21)	1.00000(20/20)
Bo_TE_178259	0.75556(34/45)	0.05556(1/18)	0.00000(0/20)	0.00000(0/19)
Bo_TE_194921	0.79545(35/44)	0.94118(16/17)	0.36364(8/22)	1.00000(19/19)
Bo_TE_223712	0.00000(0/46)	0.11111(2/18)	0.17391(4/23)	0.58824(10/17)
Bo_TE_168342	0.72727(32/44)	0.44444(8/18)	1.00000(23/23)	1.00000(18/18)
Bo_TE_145538	0.64103(25/39)	0.36842(7/19)	1.00000(23/23)	1.00000(20/20)
Bo_TE_19572	0.86957(40/46)	0.31250(5/16)	1.00000(21/21)	0.95000(19/20)
Bo_TE_89259	0.62791(27/43)	0.68421(13/19)	0.85000(17/20)	0.26316(5/19)
Bo_TE_101263	0.02174(1/46)	0.06667(1/15)	0.47619(10/21)	0.63158(12/19)
Bo_TE_203162	0.31707(13/41)	0.22222(4/18)	0.52381(11/21)	0.94737(18/19)
Bo_TE_54631	0.64286(27/42)	0.75000(12/16)	0.04348(1/23)	0.44444(8/18)
Bo_TE_64117	0.48889(22/45)	0.88235(15/17)	1.00000(20/20)	0.88235(15/17)
Bo_TE_86034	0.09756(4/41)	0.35294(6/17)	0.82609(19/23)	0.00000(0/19)
Bo_TE_59774	0.81818(36/44)	0.31250(5/16)	0.77273(17/22)	0.20000(4/20)
Bo_TE_207919	0.51111(23/45)	0.11765(2/17)	0.84211(16/19)	0.64706(11/17)
Bo_TE_5059	0.23810(10/42)	0.11765(2/17)	0.56522(13/23)	0.05263(1/19)
Bo_TE_29169	0.78571(33/42)	0.23529(4/17)	0.23810(5/21)	0.20000(3/15)
Bo_TE_146047	0.90476(38/42)	1.00000(16/16)	0.19048(4/21)	0.38889(7/18)

Bo_TE_180217	0.51282(20/39)	0.00000(0/17)	0.65000(13/20)	0.36842(7/19)
Bo_TE_84579	0.27907(12/43)	0.82353(14/17)	0.30435(7/23)	0.10000(2/20)
Bo_TE_97265	0.02222(1/45)	0.00000(0/19)	0.61111(11/18)	0.63158(12/19)
Bo_TE_31266	0.15385(6/39)	0.64706(11/17)	0.38095(8/21)	0.00000(0/20)
Bo_TE_23091	1.00000(43/43)	1.00000(17/17)	0.15000(3/20)	0.78947(15/19)
Bo_TE_191497	0.28571(12/42)	1.00000(18/18)	1.00000(22/22)	1.00000(20/20)
Bo_TE_177996	0.00000(0/45)	0.58824(10/17)	0.00000(0/23)	0.00000(0/20)
Bo_TE_82343	0.45455(20/44)	0.68750(11/16)	0.25000(5/20)	0.15000(3/20)
Bo_TE_182244	0.04444(2/45)	0.64706(11/17)	0.00000(0/22)	0.21053(4/19)
Bo_TE_48389	0.39024(16/41)	0.50000(8/16)	0.90909(20/22)	0.55000(11/20)
Bo_TE_2371	0.07692(3/39)	0.75000(12/16)	0.85000(17/20)	0.88889(16/18)
Bo_TE_83861	0.02439(1/41)	0.50000(8/16)	0.42857(9/21)	0.00000(0/20)
Bo_TE_200229	0.11905(5/42)	0.38889(7/18)	0.59091(13/22)	0.80000(16/20)
Bo_TE_58675	0.29545(13/44)	0.05263(1/19)	0.00000(0/23)	0.61111(11/18)
Bo_TE_123690	0.57778(26/45)	0.00000(0/17)	0.00000(0/22)	0.30000(6/20)
Bo_TE_49515	0.02222(1/45)	0.56250(9/16)	0.52381(11/21)	0.75000(15/20)
Bo_TE_213856	0.06522(3/46)	0.11111(2/18)	0.75000(15/20)	0.55000(11/20)
Bo_TE_102240	0.68293(28/41)	0.58824(10/17)	0.47619(10/21)	0.15000(3/20)
Bo_TE_117263	0.02273(1/44)	0.81250(13/16)	0.65217(15/23)	0.40000(6/15)
Bo_TE_177415	0.73171(30/41)	0.18750(3/16)	0.86957(20/23)	0.94737(18/19)
Bo_TE_51126	0.00000(0/44)	0.00000(0/19)	0.27273(6/22)	0.61111(11/18)
Bo_TE_158931	0.55814(24/43)	0.75000(12/16)	0.14286(3/21)	1.00000(19/19)
Bo_TE_19869	0.82500(33/40)	0.66667(12/18)	0.22727(5/22)	0.52941(9/17)
Bo_TE_216839	0.51220(21/41)	0.00000(0/17)	0.00000(0/21)	0.05000(1/20)
Bo_TE_113867	0.06818(3/44)	0.11111(2/18)	0.60000(12/20)	0.85000(17/20)
Bo_TE_69723	0.50000(21/42)	1.00000(18/18)	0.76190(16/21)	0.45000(9/20)
Bo_TE_32937	0.53659(22/41)	0.47368(9/19)	0.00000(0/23)	0.05000(1/20)
Bo_TE_138524	0.34091(15/44)	0.16667(3/18)	0.91304(21/23)	0.63158(12/19)
Bo_TE_55792	0.04444(2/45)	0.26316(5/19)	0.80952(17/21)	0.38889(7/18)
Bo_TE_143714	0.11364(5/44)	0.00000(0/18)	0.50000(10/20)	0.55000(11/20)
Bo_TE_35875	0.76923(30/39)	0.17647(3/17)	0.55000(11/20)	0.05000(1/20)
Bo_TE_201900	0.80952(34/42)	0.70588(12/17)	0.23810(5/21)	0.52632(10/19)
Bo_TE_53821	0.27907(12/43)	0.52941(9/17)	0.00000(0/22)	0.11111(2/18)
Bo_TE_174568	0.68293(28/41)	0.23529(4/17)	0.00000(0/22)	0.00000(0/19)
Bo_TE_105928	0.09091(4/44)	0.41176(7/17)	0.40909(9/22)	0.75000(15/20)
Bo_TE_195403	0.18605(8/43)	0.68750(11/16)	0.00000(0/22)	0.52941(9/17)
Bo_TE_139117	0.30769(12/39)	0.82353(14/17)	0.55556(10/18)	0.45000(9/20)
Bo_TE_234564	0.00000(0/39)	0.33333(5/15)	0.35000(7/20)	0.72222(13/18)
Bo_TE_129839	0.14286(6/42)	0.00000(0/16)	0.04545(1/22)	0.55556(10/18)
Bo_TE_27104	0.04545(2/44)	0.00000(0/16)	0.76471(13/17)	0.52941(9/17)
Bo_TE_155501	0.11905(5/42)	0.44444(8/18)	0.81818(18/22)	0.25000(5/20)
Bo_TE_168833	0.47619(20/42)	0.76471(13/17)	0.38095(8/21)	0.00000(0/19)
Bo_TE_107530	0.43182(19/44)	0.33333(6/18)	0.81818(18/22)	0.05000(1/20)
Bo_TE_119508	0.80000(36/45)	1.00000(18/18)	0.42105(8/19)	0.84211(16/19)
Bo_TE_144188	0.82051(32/39)	0.06250(1/16)	0.45000(9/20)	0.55556(10/18)
Bo_TE_11926	1.00000(46/46)	1.00000(17/17)	0.45455(10/22)	0.11111(2/18)
Bo_TE_184807	0.61111(22/36)	0.60000(9/15)	0.70000(14/20)	0.05882(1/17)
Bo_TE_38933	0.97727(43/44)	0.89474(17/19)	0.82609(19/23)	0.44444(8/18)
Bo_TE_118315	0.48837(21/43)	0.75000(12/16)	1.00000(21/21)	1.00000(19/19)
Bo_TE_55341	0.85714(36/42)	0.47059(8/17)	0.18182(4/22)	0.18750(3/16)
Bo_TE_216586	0.59524(25/42)	0.21429(3/14)	0.09524(2/21)	0.45000(9/20)
Bo_TE_4595	0.42857(9/21)	0.00000(0/14)	0.68182(15/22)	0.16667(3/18)
Bo_TE_2597	0.63043(29/46)	0.64706(11/17)	0.00000(0/22)	0.00000(0/19)
Bo_TE_108573	0.97619(41/42)	0.38889(7/18)	0.65217(15/23)	1.00000(20/20)
Bo_TE_117493	0.04651(2/43)	0.82353(14/17)	0.42857(9/21)	0.41176(7/17)
Bo_TE_108426	0.16279(7/43)	0.56250(9/16)	0.00000(0/23)	0.00000(0/20)

Bo_TE_50989	0.04348(2/46)	0.00000(0/17)	0.50000(10/20)	0.68421(13/19)
Bo_TE_227632	0.17073(7/41)	0.12500(2/16)	0.85714(18/21)	0.94737(18/19)
Bo_TE_217788	0.24390(10/41)	0.27778(5/18)	0.47619(10/21)	0.85000(17/20)
Bo_TE_136234	0.09375(3/32)	0.05882(1/17)	0.00000(0/18)	0.88889(16/18)
Bo_TE_57892	0.00000(0/46)	0.11111(2/18)	0.04348(1/23)	0.58824(10/17)
Bo_TE_8018	0.91111(41/45)	1.00000(18/18)	0.52941(9/17)	0.40000(8/20)
Bo_TE_131148	0.23810(10/42)	0.00000(0/17)	0.82609(19/23)	0.41176(7/17)
Bo_TE_179101	0.21429(9/42)	0.68750(11/16)	0.22727(5/22)	0.00000(0/20)
Bo_TE_34695	0.97727(43/44)	0.64706(11/17)	0.39130(9/23)	0.15789(3/19)
Bo_TE_225962	0.88372(38/43)	0.41176(7/17)	1.00000(21/21)	1.00000(18/18)
Bo_TE_201454	0.00000(0/45)	0.72222(13/18)	0.26087(6/23)	0.10000(2/20)
Bo_TE_108016	0.02273(1/44)	0.56250(9/16)	0.14286(3/21)	0.22222(4/18)
Bo_TE_63661	0.06667(3/45)	0.61111(11/18)	0.00000(0/23)	0.09091(1/11)
Bo_TE_179811	0.77778(35/45)	0.05882(1/17)	0.00000(0/22)	0.00000(0/20)
Bo_TE_199147	0.70000(28/40)	0.58824(10/17)	0.19048(4/21)	0.05556(1/18)
Bo_TE_86533	0.09302(4/43)	0.33333(6/18)	0.50000(10/20)	0.00000(0/18)
Bo_TE_189674	0.02273(1/44)	0.15789(3/19)	0.57143(12/21)	0.00000(0/18)
Bo_TE_188664	0.04444(2/45)	0.41176(7/17)	0.00000(0/22)	0.70000(14/20)
Bo_TE_224555	0.19512(8/41)	0.11111(2/18)	0.69565(16/23)	0.05556(1/18)
Bo_TE_87231	0.11628(5/43)	0.11111(2/18)	0.63158(12/19)	0.94118(16/17)
Bo_TE_1071	0.00000(0/37)	0.07143(1/14)	0.43478(10/23)	0.50000(9/18)
Bo_TE_178132	0.02222(1/45)	0.00000(0/19)	0.04545(1/22)	0.78947(15/19)
Bo_TE_39510	0.68182(30/44)	0.11765(2/17)	0.27273(6/22)	0.05263(1/19)
Bo_TE_165541	0.00000(0/44)	0.00000(0/18)	0.31818(7/22)	0.63158(12/19)
Bo_TE_191043	0.23256(10/43)	0.63158(12/19)	0.95238(20/21)	0.66667(12/18)
Bo_TE_88323	0.28125(9/32)	0.82353(14/17)	0.04545(1/22)	0.05556(1/18)
Bo_TE_225375	0.71429(30/42)	1.00000(17/17)	0.45000(9/20)	1.00000(20/20)
Bo_TE_141940	0.00000(0/44)	0.33333(6/18)	0.72727(16/22)	0.40000(8/20)
Bo_TE_97592	0.68182(30/44)	0.76471(13/17)	0.23810(5/21)	0.31579(6/19)
Bo_TE_194650	1.00000(40/40)	0.41176(7/17)	0.86364(19/22)	1.00000(19/19)
Bo_TE_184633	0.11111(5/45)	0.00000(0/18)	0.73684(14/19)	0.00000(0/18)
Bo_TE_101117	0.82222(37/45)	0.52941(9/17)	0.19048(4/21)	0.05000(1/20)
Bo_TE_163334	0.97826(45/46)	0.47059(8/17)	1.00000(23/23)	1.00000(20/20)
Bo_TE_58903	0.88636(39/44)	0.28571(4/14)	0.05000(1/20)	0.46667(7/15)
Bo_TE_54575	0.36585(15/41)	0.38889(7/18)	0.95455(21/22)	0.15789(3/19)
Bo_TE_214438	0.64286(27/42)	0.05882(1/17)	0.00000(0/22)	0.00000(0/17)
Bo_TE_104893	0.78261(36/46)	0.73333(11/15)	0.23810(5/21)	0.73684(14/19)
Bo_TE_119603	0.37209(16/43)	1.00000(16/16)	0.33333(7/21)	0.78947(15/19)
Bo_TE_215455	0.39535(17/43)	0.55556(10/18)	0.33333(7/21)	0.00000(0/20)
Bo_TE_67184	0.95238(40/42)	0.00000(0/15)	0.18182(4/22)	0.52941(9/17)
Bo_TE_90227	0.97778(44/45)	0.90909(10/11)	1.00000(23/23)	0.35294(6/17)
Bo_TE_179737	0.57143(24/42)	0.28571(4/14)	0.84211(16/19)	0.00000(0/20)
Bo_TE_182348	0.63158(24/38)	0.15385(2/13)	0.05556(1/18)	0.00000(0/17)
Bo_TE_142562	0.33333(14/42)	0.88889(16/18)	0.95652(22/23)	0.47368(9/19)
Bo_TE_158333	0.90909(40/44)	0.82353(14/17)	0.14286(3/21)	0.29412(5/17)
Bo_TE_82405	0.02222(1/45)	0.11111(2/18)	0.54545(12/22)	0.80000(16/20)
Bo_TE_95605	0.97500(39/40)	0.47368(9/19)	0.90476(19/21)	0.88889(16/18)
Bo_TE_70332	0.80952(34/42)	0.62500(10/16)	0.14286(3/21)	0.42105(8/19)
Bo_TE_130813	0.33333(14/42)	0.87500(14/16)	0.45000(9/20)	0.15000(3/20)
Bo_TE_129201	0.21429(9/42)	0.88235(15/17)	0.09524(2/21)	0.15000(3/20)
Bo_TE_122441	0.80435(37/46)	1.00000(17/17)	0.26316(5/19)	0.60000(12/20)
Bo_TE_230858	0.54762(23/42)	0.05556(1/18)	0.00000(0/21)	0.00000(0/20)
Bo_TE_83537	0.40909(18/44)	0.52632(10/19)	0.63636(14/22)	1.00000(19/19)
Bo_TE_88771	0.50000(21/42)	0.62500(10/16)	0.60000(12/20)	0.10000(2/20)
Bo_TE_13441	0.11628(5/43)	0.11765(2/17)	1.00000(20/20)	0.78947(15/19)
Bo_TE_62490	0.00000(0/42)	0.00000(0/13)	0.16667(3/18)	0.52941(9/17)

Bo_TE_165518	0.00000(0/44)	0.00000(0/18)	0.33333(7/21)	0.60000(12/20)
Bo_TE_235656	0.64444(29/45)	0.94444(17/18)	0.13636(3/22)	0.40000(8/20)
Bo_TE_122758	0.00000(0/43)	0.00000(0/19)	0.52381(11/21)	0.22222(4/18)
Bo_TE_185122	0.76744(33/43)	0.75000(12/16)	0.14286(3/21)	0.00000(0/20)
Bo_TE_34640	0.00000(0/45)	0.00000(0/18)	0.57143(12/21)	0.83333(15/18)
Bo_TE_16726	0.11905(5/42)	0.43750(7/16)	0.85000(17/20)	0.78947(15/19)
Bo_TE_55528	0.26190(11/42)	0.56250(9/16)	0.00000(0/22)	0.05263(1/19)
Bo_TE_107210	0.04444(2/45)	0.17647(3/17)	0.50000(10/20)	0.60000(12/20)
Bo_TE_9220	0.78571(33/42)	0.37500(6/16)	0.80952(17/21)	1.00000(20/20)
Bo_TE_138906	0.48718(19/39)	0.87500(14/16)	0.47619(10/21)	0.15000(3/20)
Bo_TE_60920	0.34884(15/43)	0.37500(6/16)	0.95238(20/21)	0.88889(16/18)
Bo_TE_52679	0.27273(9/33)	0.25000(4/16)	0.00000(0/2)	0.94118(16/17)
Bo_TE_156704	0.48780(20/41)	0.31250(5/16)	0.95455(21/22)	0.77778(14/18)
Bo_TE_138834	0.19048(8/42)	0.17647(3/17)	0.47619(10/21)	0.75000(15/20)
Bo_TE_52847	0.19048(8/42)	0.88889(16/18)	0.85000(17/20)	0.84211(16/19)
Bo_TE_3057	0.75676(28/37)	0.56250(9/16)	0.33333(7/21)	0.89474(17/19)
Bo_TE_11653	0.42500(17/40)	0.23529(4/17)	0.04545(1/22)	0.61111(11/18)
Bo_TE_5011	0.57500(23/40)	0.00000(0/18)	0.00000(0/23)	0.00000(0/20)
Bo_TE_11272	0.00000(0/46)	0.17647(3/17)	0.75000(15/20)	0.00000(0/20)
Bo_TE_179039	0.88372(38/43)	0.05882(1/17)	0.00000(0/23)	0.10000(2/20)
Bo_TE_34545	0.91304(42/46)	0.57895(11/19)	0.42857(9/21)	0.17647(3/17)
Bo_TE_30543	0.24390(10/41)	0.05263(1/19)	0.68421(13/19)	0.15000(3/20)
Bo_TE_222638	0.88372(38/43)	0.64706(11/17)	0.09091(2/22)	0.20000(4/20)
Bo_TE_206587	0.06818(3/44)	0.18750(3/16)	0.09524(2/21)	0.63158(12/19)
Bo_TE_230085	0.68421(26/38)	0.87500(14/16)	0.05882(1/17)	0.26316(5/19)
Bo_TE_54056	0.30000(12/40)	0.87500(14/16)	0.50000(10/20)	0.26316(5/19)
Bo_TE_80660	0.19048(8/42)	0.23529(4/17)	0.45455(10/22)	0.85000(17/20)
Bo_TE_90980	0.74419(32/43)	0.75000(12/16)	0.55556(10/18)	0.15000(3/20)
Bo_TE_197496	0.00000(0/42)	0.10526(2/19)	0.18182(4/22)	0.61111(11/18)
Bo_TE_94587	0.48889(22/45)	0.58824(10/17)	0.26087(6/23)	0.00000(0/19)
Bo_TE_133361	0.44737(17/38)	0.88889(16/18)	0.27273(6/22)	0.55556(10/18)
Bo_TE_227992	0.00000(0/45)	0.00000(0/19)	0.80000(16/20)	0.44444(8/18)
Bo_TE_26846	0.56098(23/41)	1.00000(17/17)	1.00000(21/21)	0.42105(8/19)
Bo_TE_37397	0.54054(20/37)	0.05556(1/18)	0.00000(0/23)	0.00000(0/20)
Bo_TE_96405	0.68182(30/44)	0.94444(17/18)	0.54545(12/22)	0.25000(5/20)
Bo_TE_177167	0.00000(0/44)	0.61111(11/18)	0.00000(0/22)	0.00000(0/20)
Bo_TE_5020	0.25641(10/39)	0.11765(2/17)	0.57143(12/21)	0.05000(1/20)
Bo_TE_39989	0.00000(0/44)	0.00000(0/13)	0.57143(12/21)	0.05263(1/19)
Bo_TE_207035	0.09524(4/42)	0.00000(0/18)	0.04762(1/21)	0.56250(9/16)
Bo_TE_168518	0.30952(13/42)	0.94118(16/17)	0.28571(6/21)	0.15789(3/19)
Bo_TE_198	0.88372(38/43)	0.93750(15/16)	0.95455(21/22)	0.42105(8/19)
Bo_TE_85703	0.56818(25/44)	0.25000(4/16)	0.00000(0/22)	0.33333(6/18)
Bo_TE_155094	0.13636(6/44)	0.11111(2/18)	0.27273(6/22)	0.70000(14/20)
Bo_TE_76070	0.08696(4/46)	0.00000(0/18)	0.60870(14/23)	0.15789(3/19)
Bo_TE_50661	0.58140(25/43)	0.84211(16/19)	1.00000(15/15)	0.10526(2/19)
Bo_TE_145945	0.11111(5/45)	0.72222(13/18)	0.42857(9/21)	0.72222(13/18)
Bo_TE_54802	0.70732(29/41)	0.00000(0/14)	0.09091(2/22)	0.00000(0/14)
Bo_TE_44009	0.51220(21/41)	0.77778(14/18)	0.38095(8/21)	0.11111(2/18)
Bo_TE_207833	0.52273(23/44)	0.16667(3/18)	0.82609(19/23)	0.63158(12/19)
Bo_TE_237767	1.00000(46/46)	1.00000(19/19)	0.50000(10/20)	0.72222(13/18)
Bo_TE_143593	0.16279(7/43)	0.75000(12/16)	0.52381(11/21)	0.45000(9/20)
Bo_TE_105907	0.63415(26/41)	0.13333(2/15)	0.00000(0/23)	0.05263(1/19)
Bo_TE_213796	0.11905(5/42)	0.20000(3/15)	0.15000(3/20)	0.72222(13/18)
Bo_TE_189682	0.02222(1/45)	0.16667(3/18)	0.52381(11/21)	0.00000(0/20)
Bo_TE_36916	0.06818(3/44)	0.00000(0/19)	0.00000(0/23)	0.70588(12/17)
Bo_TE_130517	0.83721(36/43)	0.70588(12/17)	0.23810(5/21)	0.78947(15/19)

Bo_TE_121654	0.09091(4/44)	0.76471(13/17)	0.00000(0/23)	0.10526(2/19)
Bo_TE_34790	0.00000(0/46)	0.72222(13/18)	0.00000(0/22)	0.00000(0/20)
Bo_TE_83925	0.13333(6/45)	0.00000(0/18)	0.59091(13/22)	0.83333(15/18)
Bo_TE_131803	0.89189(33/37)	0.71429(10/14)	0.33333(7/21)	0.21429(3/14)
Bo_TE_10914	0.88095(37/42)	0.76471(13/17)	0.95238(20/21)	0.17647(3/17)
Bo_TE_43605	0.20000(8/40)	0.87500(7/8)	1.00000(23/23)	0.63158(12/19)
Bo_TE_145962	0.11364(5/44)	0.76471(13/17)	0.40000(8/20)	0.70588(12/17)
Bo_TE_66062	0.15152(5/33)	0.00000(0/16)	0.86364(19/22)	0.23529(4/17)
Bo_TE_50815	0.47500(19/40)	0.83333(15/18)	0.10000(2/20)	0.55000(11/20)
Bo_TE_37464	1.00000(37/37)	0.41176(7/17)	1.00000(22/22)	0.94118(16/17)
Bo_TE_111555	0.13953(6/43)	0.00000(0/19)	0.76190(16/21)	0.00000(0/20)
Bo_TE_197381	1.00000(44/44)	0.87500(14/16)	1.00000(23/23)	0.42105(8/19)
Bo_TE_197472	0.02273(1/44)	0.10526(2/19)	0.17391(4/23)	0.61111(11/18)
Bo_TE_3267	0.67500(27/40)	0.44444(8/18)	0.08696(2/23)	0.26316(5/19)
Bo_TE_111936	0.38636(17/44)	0.64706(11/17)	0.23810(5/21)	0.88889(16/18)
Bo_TE_138296	0.27907(12/43)	0.52632(10/19)	0.19048(4/21)	0.88235(15/17)
Bo_TE_180352	0.73333(33/45)	0.68750(11/16)	0.36364(8/22)	0.15000(3/20)
Bo_TE_23040	0.80435(37/46)	0.11765(2/17)	0.18182(4/22)	0.10526(2/19)
Bo_TE_149970	0.73810(31/42)	0.55556(10/18)	0.23810(5/21)	0.10000(2/20)
Bo_TE_75981	0.94444(17/18)	0.94737(18/19)	0.14286(3/21)	0.50000(9/18)
Bo_TE_57565	0.57143(24/42)	0.87500(14/16)	0.42857(9/21)	0.00000(0/20)
Bo_TE_141449	0.20000(9/45)	0.94118(16/17)	0.95455(21/22)	0.78947(15/19)
Bo_TE_212234	0.80488(33/41)	0.35294(6/17)	0.55000(11/20)	0.94737(18/19)
Bo_TE_15002	1.00000(45/45)	0.25000(4/16)	0.33333(7/21)	0.11111(2/18)
Bo_TE_129756	0.50000(18/36)	0.00000(0/16)	0.13636(3/22)	0.50000(8/16)
Bo_TE_101870	0.00000(0/39)	0.00000(0/7)	0.76190(16/21)	0.00000(0/12)
Bo_TE_226173	0.27500(11/40)	0.77778(14/18)	1.00000(22/22)	0.83333(15/18)
Bo_TE_27525	0.00000(0/37)	0.00000(0/18)	0.61905(13/21)	0.00000(0/14)
Bo_TE_97662	0.27273(12/44)	0.73333(11/15)	1.00000(21/21)	0.78947(15/19)
Bo_TE_57960	1.00000(43/43)	1.00000(16/16)	0.80952(17/21)	0.25000(5/20)
Bo_TE_147971	0.86364(38/44)	0.70588(12/17)	0.10526(2/19)	0.00000(0/20)
Bo_TE_126189	0.44186(19/43)	0.00000(0/17)	0.50000(10/20)	0.00000(0/20)
Bo_TE_5824	0.00000(0/45)	0.00000(0/18)	0.14286(3/21)	0.78947(15/19)
Bo_TE_230962	0.00000(0/46)	0.68750(11/16)	0.00000(0/23)	0.10526(2/19)
Bo_TE_65291	0.65116(28/43)	0.13333(2/15)	0.47368(9/19)	0.05882(1/17)
Bo_TE_36416	0.11364(5/44)	1.00000(18/18)	1.00000(23/23)	0.83333(15/18)
Bo_TE_163448	0.19048(8/42)	0.00000(0/17)	0.71429(15/21)	0.05000(1/20)
Bo_TE_150422	0.00000(0/45)	0.10526(2/19)	0.27273(6/22)	0.73684(14/19)
Bo_TE_194729	0.17949(7/39)	0.16667(3/18)	0.68421(13/19)	0.31250(5/16)
Bo_TE_54348	0.95122(39/41)	0.41176(7/17)	0.66667(14/21)	0.42105(8/19)
Bo_TE_77270	0.00000(0/42)	0.00000(0/17)	0.00000(0/23)	0.60000(12/20)
Bo_TE_43331	0.97674(42/43)	0.47059(8/17)	1.00000(22/22)	1.00000(20/20)
Bo_TE_57406	0.73810(31/42)	0.05882(1/17)	0.61905(13/21)	0.66667(12/18)
Bo_TE_184323	0.40000(16/40)	0.56250(9/16)	0.76471(13/17)	1.00000(18/18)
Bo_TE_191154	1.00000(42/42)	1.00000(17/17)	0.71429(15/21)	0.22222(4/18)
Bo_TE_45114	0.43182(19/44)	0.16667(3/18)	0.63636(14/22)	0.00000(0/20)
Bo_TE_95340	0.02222(1/45)	0.05556(1/18)	0.04545(1/22)	0.60000(12/20)
Bo_TE_24762	0.36585(15/41)	0.66667(12/18)	0.14286(3/21)	0.30000(6/20)
Bo_TE_101103	0.24390(10/41)	0.70588(12/17)	0.13636(3/22)	0.89474(17/19)
Bo_TE_60539	0.25581(11/43)	0.73333(11/15)	1.00000(21/21)	0.88889(16/18)
Bo_TE_43112	0.45455(20/44)	1.00000(17/17)	1.00000(22/22)	0.70000(14/20)
Bo_TE_229882	0.33333(13/39)	0.25000(4/16)	0.52381(11/21)	0.80000(16/20)
Bo_TE_144811	0.81818(36/44)	0.11111(2/18)	0.00000(0/22)	0.57895(11/19)
Bo_TE_45591	0.59524(25/42)	0.00000(0/16)	0.23810(5/21)	0.00000(0/18)
Bo_TE_27667	0.50000(22/44)	0.23529(4/17)	0.80000(16/20)	0.80000(16/20)
Bo_TE_128878	0.02222(1/45)	0.15789(3/19)	0.52381(11/21)	0.00000(0/20)

Bo_TE_99956	0.45455(20/44)	0.64706(11/17)	0.00000(0/21)	0.00000(0/20)
Bo_TE_148620	0.41463(17/41)	0.52632(10/19)	0.63636(14/22)	1.00000(20/20)
Bo_TE_174781	0.25000(11/44)	0.50000(9/18)	0.52381(11/21)	0.85000(17/20)
Bo_TE_72881	0.58537(24/41)	0.44444(8/18)	0.91304(21/23)	1.00000(19/19)
Bo_TE_89163	0.02222(1/45)	0.00000(0/18)	0.86364(19/22)	0.63158(12/19)
Bo_TE_48115	0.75000(30/40)	0.05263(1/19)	0.00000(0/23)	0.05000(1/20)
Bo_TE_133080	0.32558(14/43)	1.00000(17/17)	0.28571(6/21)	0.68421(13/19)
Bo_TE_205887	0.19512(8/41)	0.29412(5/17)	1.00000(21/21)	0.94737(18/19)
Bo_TE_15624	0.41463(17/41)	0.68750(11/16)	0.00000(0/22)	0.00000(0/19)
Bo_TE_14646	0.73171(30/41)	0.64706(11/17)	0.19048(4/21)	0.05263(1/19)
Bo_TE_58622	0.71429(30/42)	0.06250(1/16)	0.55556(10/18)	0.57895(11/19)
Bo_TE_121366	0.29268(12/41)	0.11765(2/17)	0.61905(13/21)	0.10000(1/10)
Bo_TE_169082	0.35714(15/42)	0.94118(16/17)	0.90476(19/21)	0.60000(12/20)
Bo_TE_129193	0.21429(9/42)	0.94118(16/17)	0.22727(5/22)	0.31579(6/19)
Bo_TE_173016	0.37778(17/45)	0.00000(0/18)	0.85714(18/21)	0.20000(4/20)
Bo_TE_124417	0.76316(29/38)	0.83333(15/18)	0.23810(5/21)	0.05882(1/17)
Bo_TE_138839	0.72727(32/44)	0.81250(13/16)	0.55000(11/20)	0.25000(5/20)
Bo_TE_210983	0.10870(5/46)	0.43750(7/16)	0.57895(11/19)	0.61111(11/18)
Bo_TE_215388	0.86667(39/45)	0.43750(7/16)	0.08696(2/23)	0.11765(2/17)
Bo_TE_199044	0.70732(29/41)	0.23529(4/17)	0.00000(0/22)	0.00000(0/20)
Bo_TE_123938	0.25000(11/44)	1.00000(19/19)	0.76190(16/21)	0.94737(18/19)
Bo_TE_23072	0.97727(43/44)	0.47368(9/19)	1.00000(23/23)	0.70000(14/20)
Bo_TE_162868	0.24444(11/45)	0.21053(4/19)	0.35000(7/20)	0.77778(14/18)
Bo_TE_207133	0.89286(25/28)	0.50000(9/18)	0.88889(16/18)	1.00000(18/18)
Bo_TE_177427	0.41463(17/41)	0.76471(13/17)	0.13636(3/22)	0.05000(1/20)
Bo_TE_230817	0.02174(1/46)	0.56250(9/16)	0.42857(9/21)	0.00000(0/20)
Bo_TE_4540	0.97778(44/45)	0.31250(5/16)	0.80952(17/21)	0.55556(10/18)
Bo_TE_83946	0.13636(6/44)	0.00000(0/18)	0.61905(13/21)	0.80000(16/20)
Bo_TE_144752	0.86667(39/45)	0.10526(2/19)	0.15000(3/20)	0.95000(19/20)
Bo_TE_73606	0.70000(28/40)	0.17647(3/17)	0.00000(0/23)	0.05000(1/20)
Bo_TE_104851	0.00000(0/43)	0.05882(1/17)	0.22727(5/22)	0.57895(11/19)
Bo_TE_132959	0.79070(34/43)	0.58824(10/17)	0.15000(3/20)	0.94737(18/19)
Bo_TE_173716	0.95349(41/43)	0.94737(18/19)	0.78261(18/23)	0.40000(8/20)
Bo_TE_138159	0.27907(12/43)	0.00000(0/16)	0.85000(17/20)	0.36842(7/19)
Bo_TE_48545	0.65909(29/44)	0.00000(0/17)	0.00000(0/23)	0.42105(8/19)
Bo_TE_140992	0.89474(34/38)	0.05882(1/17)	0.76471(13/17)	0.05882(1/17)
Bo_TE_207168	0.38095(16/42)	0.61111(11/18)	0.61111(11/18)	0.10000(2/20)
Bo_TE_189384	0.00000(0/44)	0.54545(6/11)	0.25000(5/20)	0.85000(17/20)
Bo_TE_190900	0.02326(1/43)	0.62500(10/16)	0.00000(0/23)	0.10000(2/20)
Bo_TE_93681	0.00000(0/42)	0.00000(0/5)	0.77273(17/22)	0.00000(0/20)
Bo_TE_232709	0.97826(45/46)	1.00000(19/19)	0.61905(13/21)	0.22222(4/18)
Bo_TE_67072	0.82927(34/41)	0.05882(1/17)	0.18182(4/22)	0.73684(14/19)
Bo_TE_236221	0.88636(39/44)	0.12500(2/16)	0.00000(0/23)	0.16667(3/18)
Bo_TE_135857	0.02222(1/45)	0.00000(0/19)	0.45000(9/20)	0.77778(14/18)
Bo_TE_154634	0.72093(31/43)	0.73333(11/15)	0.21053(4/19)	0.68421(13/19)
Bo_TE_162589	0.63636(28/44)	0.22222(4/18)	0.42857(9/21)	0.00000(0/20)
Bo_TE_30193	0.93023(40/43)	0.29412(5/17)	0.20000(4/20)	0.50000(9/18)
Bo_TE_146007	0.86047(37/43)	0.75000(3/4)	0.14286(2/14)	0.05263(1/19)
Bo_TE_171135	0.52381(22/42)	0.75000(12/16)	0.57143(12/21)	0.15789(3/19)
Bo_TE_163363	0.20455(9/44)	0.58824(10/17)	0.00000(0/23)	0.20000(4/20)
Bo_TE_52690	0.86047(37/43)	0.06250(1/16)	0.08696(2/23)	0.05556(1/18)
Bo_TE_189061	0.73171(30/41)	0.40000(6/15)	0.00000(0/22)	0.05263(1/19)
Bo_TE_120868	0.41463(17/41)	0.33333(6/18)	0.66667(14/21)	0.00000(0/20)
Bo_TE_111951	0.60976(25/41)	0.35294(6/17)	0.72222(13/18)	0.10526(2/19)
Bo_TE_206012	0.80000(36/45)	0.68750(11/16)	0.00000(0/23)	0.10000(2/20)
Bo_TE_119998	0.00000(0/43)	0.26316(5/19)	0.66667(14/21)	0.61111(11/18)

Bo_TE_163844	0.04762(2/42)	0.00000(0/18)	0.33333(7/21)	0.78947(15/19)
Bo_TE_50281	0.65909(29/44)	0.17647(3/17)	0.38095(8/21)	0.10000(2/20)
Bo_TE_128449	0.23256(10/43)	0.68750(11/16)	0.63158(12/19)	0.00000(0/20)
Bo_TE_231733	0.80000(32/40)	0.52941(9/17)	0.57143(12/21)	0.20000(4/20)
Bo_TE_18925	0.31707(13/41)	0.75000(12/16)	0.04762(1/21)	0.15789(3/19)
Bo_TE_161676	1.00000(45/45)	0.58824(10/17)	0.40000(8/20)	0.77778(14/18)
Bo_TE_76694	0.51282(20/39)	0.50000(9/18)	0.00000(0/23)	0.00000(0/20)
Bo_TE_134604	0.09524(4/42)	0.12500(2/16)	0.40909(9/22)	0.80000(16/20)
Bo_TE_232695	0.54762(23/42)	0.00000(0/18)	0.33333(7/21)	0.84211(16/19)
Bo_TE_76198	0.52632(20/38)	0.00000(0/18)	0.00000(0/18)	0.00000(0/18)
Bo_TE_90663	0.00000(0/45)	0.00000(0/19)	0.04348(1/23)	0.65000(13/20)
Bo_TE_158395	0.80000(36/45)	0.47059(8/17)	1.00000(22/22)	0.89474(17/19)
Bo_TE_74243	0.52381(22/42)	0.33333(6/18)	0.00000(0/23)	0.00000(0/20)
Bo_TE_53619	0.02326(1/43)	0.00000(0/19)	0.00000(0/21)	0.68750(11/16)
Bo_TE_48980	0.00000(0/44)	0.00000(0/19)	0.63636(14/22)	0.55556(10/18)
Bo_TE_122307	0.95556(43/45)	0.35294(6/17)	1.00000(22/22)	0.68421(13/19)
Bo_TE_106375	0.58140(25/43)	0.93750(15/16)	0.34783(8/23)	0.66667(12/18)
Bo_TE_65375	0.80000(32/40)	0.70588(12/17)	0.15000(3/20)	0.80000(16/20)
Bo_TE_191535	0.26190(11/42)	0.81250(13/16)	1.00000(22/22)	1.00000(20/20)
Bo_TE_66117	0.10870(5/46)	0.21053(4/19)	0.76190(16/21)	0.42105(8/19)
Bo_TE_32705	0.04545(2/44)	0.15789(3/19)	0.65217(15/23)	0.05000(1/20)
Bo_TE_136018	0.67442(29/43)	0.68421(13/19)	0.04348(1/23)	0.00000(0/18)
Bo_TE_61337	0.88636(39/44)	0.63158(12/19)	0.21739(5/23)	0.25000(5/20)
Bo_TE_170831	0.00000(0/43)	0.11111(2/18)	0.50000(10/20)	0.00000(0/20)
Bo_TE_43510	0.85366(35/41)	0.55556(10/18)	0.00000(0/22)	0.47368(9/19)
Bo_TE_91667	0.97778(44/45)	0.38889(7/18)	0.38095(8/21)	0.85000(17/20)
Bo_TE_124589	0.65854(27/41)	0.17647(3/17)	0.15789(3/19)	0.00000(0/19)
Bo_TE_53422	0.24390(10/41)	1.00000(13/13)	0.75000(15/20)	1.00000(20/20)
Bo_TE_18931	0.32500(13/40)	0.73684(14/19)	0.00000(0/20)	0.05000(1/20)
Bo_TE_172922	0.00000(0/45)	0.00000(0/18)	0.56522(13/23)	0.75000(15/20)
Bo_TE_215798	0.65854(27/41)	1.00000(18/18)	0.78261(18/23)	0.33333(6/18)
Bo_TE_78577	0.83333(35/42)	0.68750(11/16)	0.42857(9/21)	0.05000(1/20)
Bo_TE_215949	0.97674(42/43)	0.41176(7/17)	0.06667(1/15)	0.20000(3/15)
Bo_TE_219102	0.75000(30/40)	0.05556(1/18)	0.27273(6/22)	0.15789(3/19)
Bo_TE_103906	0.54762(23/42)	0.22222(4/18)	0.04348(1/23)	0.05000(1/20)
Bo_TE_190882	0.68182(30/44)	0.00000(0/13)	0.00000(0/2)	0.00000(0/16)
Bo_TE_156871	0.17778(8/45)	0.26667(4/15)	1.00000(23/23)	1.00000(19/19)
Bo_TE_112500	0.88889(40/45)	0.88889(16/18)	0.42857(9/21)	0.95000(19/20)
Bo_TE_196749	0.54762(23/42)	0.11765(2/17)	0.04545(1/22)	0.00000(0/20)
Bo_TE_90207	0.75610(31/41)	0.52941(9/17)	0.38095(8/21)	0.10000(2/20)
Bo_TE_145540	0.63415(26/41)	0.29412(5/17)	1.00000(21/21)	1.00000(20/20)
Bo_TE_29683	0.62791(27/43)	0.70588(12/17)	0.95652(22/23)	0.30000(6/20)
Bo_TE_53428	0.25581(11/43)	0.00000(0/19)	0.52381(11/21)	0.21053(4/19)
Bo_TE_146283	0.22500(9/40)	0.38889(7/18)	0.57143(12/21)	0.05263(1/19)
Bo_TE_223443	1.00000(45/45)	0.82353(14/17)	0.26316(5/19)	0.55556(10/18)
Bo_TE_172375	0.52632(20/38)	0.00000(0/18)	0.85714(18/21)	0.31579(6/19)
Bo_TE_216844	0.61538(24/39)	0.00000(0/17)	0.09091(2/22)	0.42105(8/19)
Bo_TE_13969	0.12195(5/41)	0.69231(9/13)	0.58824(10/17)	0.72222(13/18)
Bo_TE_120606	0.58537(24/41)	0.23529(4/17)	0.90909(20/22)	0.47368(9/19)
Bo_TE_195464	0.60976(25/41)	0.41176(7/17)	0.38095(8/21)	0.05000(1/20)
Bo_TE_55541	0.00000(0/44)	0.68750(11/16)	0.00000(0/22)	0.22222(4/18)
Bo_TE_224660	0.39535(17/43)	0.83333(15/18)	0.38095(8/21)	0.89474(17/19)
Bo_TE_162860	0.25581(11/43)	0.17647(3/17)	0.34783(8/23)	0.80000(16/20)
Bo_TE_239690	0.76316(29/38)	0.37500(6/16)	0.00000(0/23)	0.00000(0/18)
Bo_TE_161890	0.40000(16/40)	0.06667(1/15)	0.95000(19/20)	0.66667(12/18)
Bo_TE_91534	0.06818(3/44)	0.44444(8/18)	0.80952(17/21)	0.45000(9/20)

Bo_TE_159053	0.75556(34/45)	0.85714(12/14)	0.15000(3/20)	0.20000(4/20)
Bo_TE_194672	1.00000(45/45)	1.00000(18/18)	1.00000(22/22)	0.27778(5/18)
Bo_TE_75308	0.02273(1/44)	0.23529(4/17)	0.60000(12/20)	0.00000(0/20)
Bo_TE_106921	0.63636(28/44)	0.17647(3/17)	0.13636(3/22)	0.20000(4/20)
Bo_TE_100781	0.81395(35/43)	1.00000(17/17)	0.95000(19/20)	0.15000(3/20)
Bo_TE_198147	0.37209(16/43)	0.62500(10/16)	0.22222(4/18)	0.94444(17/18)
Bo_TE_99639	0.16667(7/42)	0.81818(9/11)	1.00000(21/21)	0.94737(18/19)
Bo_TE_179968	0.63158(24/38)	0.10526(2/19)	0.00000(0/21)	0.10526(2/19)
Bo_TE_91545	0.25000(11/44)	0.41176(7/17)	0.23810(5/21)	0.78947(15/19)
Bo_TE_213046	0.78571(33/42)	0.05556(1/18)	0.13636(3/22)	0.15000(3/20)
Bo_TE_224098	0.66667(30/45)	1.00000(18/18)	0.52381(11/21)	0.37500(6/16)
Bo_TE_138193	0.69767(30/43)	0.25000(4/16)	0.00000(0/19)	0.06250(1/16)
Bo_TE_42100	0.06818(3/44)	0.10526(2/19)	0.04545(1/22)	0.78947(15/19)
Bo_TE_153065	0.00000(0/42)	0.26667(4/15)	0.72222(13/18)	0.50000(8/16)
Bo_TE_97780	0.26829(11/41)	0.88889(16/18)	0.04762(1/21)	0.16667(3/18)
Bo_TE_30963	0.75610(31/41)	0.68750(11/16)	0.21053(4/19)	0.72222(13/18)
Bo_TE_230540	0.08889(4/45)	0.00000(0/18)	0.19048(4/21)	0.50000(9/18)
Bo_TE_113752	0.10256(4/39)	0.17647(3/17)	0.42105(8/19)	0.85000(17/20)
Bo_TE_159145	0.16279(7/43)	0.50000(8/16)	1.00000(22/22)	1.00000(19/19)
Bo_TE_101583	0.93478(43/46)	0.43750(7/16)	0.25000(5/20)	0.70588(12/17)
Bo_TE_93246	0.00000(0/45)	0.00000(0/19)	0.73913(17/23)	0.30000(6/20)
Bo_TE_8527	0.56818(25/44)	0.06250(1/16)	0.28571(6/21)	0.70000(14/20)
Bo_TE_141452	0.18182(8/44)	0.88889(16/18)	0.95455(21/22)	0.76471(13/17)
Bo_TE_195570	0.84615(33/39)	0.60000(9/15)	0.50000(11/22)	0.21053(4/19)
Bo_TE_16671	0.70270(26/37)	0.40000(6/15)	0.33333(7/21)	0.20000(4/20)
Bo_TE_29791	0.68293(28/41)	0.22222(4/18)	0.00000(0/22)	0.05263(1/19)
Bo_TE_127528	0.83333(35/42)	0.37500(6/16)	0.13636(3/22)	0.00000(0/20)
Bo_TE_53557	0.10256(4/39)	0.00000(0/16)	0.15000(3/20)	0.85000(17/20)
Bo_TE_24496	0.16667(7/42)	0.61111(11/18)	0.81818(18/22)	0.44444(8/18)
Bo_TE_138321	0.07317(3/41)	0.68421(13/19)	0.23810(5/21)	0.57895(11/19)
Bo_TE_96288	0.57143(24/42)	0.05556(1/18)	0.00000(0/23)	0.05000(1/20)
Bo_TE_37504	0.87805(36/41)	0.77778(14/18)	0.38095(8/21)	0.10526(2/19)
Bo_TE_226195	0.57895(22/38)	0.11765(2/17)	0.61905(13/21)	0.05263(1/19)
Bo_TE_158925	0.13953(6/43)	0.75000(12/16)	0.04545(1/22)	0.55000(11/20)
Bo_TE_92940	0.02381(1/42)	0.00000(0/19)	0.70000(14/20)	0.84615(11/13)
Bo_TE_6022	0.29545(13/44)	0.11765(2/17)	0.27273(6/22)	1.00000(19/19)
Bo_TE_170134	0.47727(21/44)	0.66667(12/18)	1.00000(22/22)	0.57895(11/19)
Bo_TE_45824	0.08889(4/45)	0.00000(0/18)	0.00000(0/22)	0.65000(13/20)
Bo_TE_241823	0.23077(9/39)	0.93750(15/16)	0.52381(11/21)	0.90000(18/20)
Bo_TE_7026	0.78947(30/38)	0.23529(4/17)	0.13043(3/23)	0.05000(1/20)
Bo_TE_61428	0.46341(19/41)	0.11765(2/17)	0.90476(19/21)	0.84211(16/19)
Bo_TE_144814	0.13953(6/43)	0.93750(15/16)	1.00000(21/21)	0.29412(5/17)
Bo_TE_122707	0.56098(23/41)	0.26316(5/19)	0.00000(0/22)	0.00000(0/18)
Bo_TE_232114	0.56818(25/44)	0.47059(8/17)	0.04348(1/23)	0.30000(6/20)
Bo_TE_49023	0.13514(5/37)	0.00000(0/18)	0.55000(11/20)	0.31579(6/19)
Bo_TE_186400	0.83333(35/42)	0.23529(4/17)	0.50000(10/20)	0.94737(18/19)
Bo_TE_27505	0.00000(0/44)	0.00000(0/19)	0.55000(11/20)	0.00000(0/20)
Bo_TE_173080	0.09756(4/41)	0.52941(9/17)	0.00000(0/22)	0.00000(0/20)
Bo_TE_4963	0.48889(22/45)	0.29412(5/17)	0.95238(20/21)	0.90000(18/20)
Bo_TE_89218	0.48889(22/45)	0.05882(1/17)	0.40000(2/5)	0.88889(16/18)
Bo_TE_101315	0.00000(0/45)	0.00000(0/16)	0.45455(10/22)	0.70000(14/20)
Bo_TE_69834	0.55814(24/43)	0.41176(7/17)	0.36364(8/22)	0.90000(18/20)
Bo_TE_9971	0.61905(26/42)	0.11765(2/17)	0.71429(15/21)	1.00000(19/19)
Bo_TE_227345	0.04444(2/45)	0.58824(10/17)	0.18182(4/22)	0.10000(2/20)
Bo_TE_70512	0.52273(23/44)	0.05556(1/18)	0.00000(0/23)	0.05000(1/20)
Bo_TE_57136	0.00000(0/44)	0.00000(0/19)	0.22727(5/22)	0.52632(10/19)

Bo_TE_57868	0.76923(30/39)	0.43750(7/16)	0.54545(12/22)	0.20000(4/20)
Bo_TE_130335	0.69231(27/39)	0.94118(16/17)	1.00000(22/22)	0.35000(7/20)
Bo_TE_191207	0.69767(30/43)	0.10526(2/19)	0.65000(13/20)	0.00000(0/18)
Bo_TE_74445	0.02326(1/43)	0.00000(0/16)	0.57143(12/21)	0.10000(2/20)
Bo_TE_111870	0.77778(35/45)	0.05882(1/17)	0.76190(16/21)	0.27778(5/18)
Bo_TE_38705	0.02500(1/40)	0.13333(2/15)	0.33333(7/21)	0.70000(14/20)
Bo_TE_92766	0.88095(37/42)	0.42857(6/14)	0.90476(19/21)	0.25000(4/16)
Bo_TE_76178	0.44186(19/43)	0.11765(2/17)	0.85714(18/21)	0.55556(10/18)
Bo_TE_119432	0.15556(7/45)	0.06250(1/16)	0.57143(12/21)	0.47368(9/19)
Bo_TE_119493	0.18182(8/44)	0.00000(0/18)	0.55000(11/20)	0.15000(3/20)
Bo_TE_42269	0.13043(6/46)	0.35294(6/17)	0.78261(18/23)	0.90000(18/20)
Bo_TE_130275	0.32558(14/43)	0.83333(15/18)	0.00000(0/22)	0.16667(3/18)
Bo_TE_197613	0.97674(42/43)	0.22222(4/18)	0.91304(21/23)	0.44444(8/18)
Bo_TE_148626	0.43182(19/44)	0.58824(10/17)	1.00000(22/22)	1.00000(20/20)
Bo_TE_227070	0.75862(22/29)	0.37500(6/16)	1.00000(21/21)	0.80000(16/20)
Bo_TE_173707	0.93333(42/45)	0.88889(16/18)	0.38095(8/21)	0.20000(4/20)
Bo_TE_138535	0.48837(21/43)	0.83333(15/18)	0.04545(1/22)	0.33333(6/18)
Bo_TE_172569	0.02222(1/45)	0.93333(14/15)	0.52381(11/21)	0.36842(7/19)
Bo_TE_57344	0.02222(1/45)	0.00000(0/19)	0.22727(5/22)	0.63158(12/19)
Bo_TE_10274	0.25000(10/40)	1.00000(16/16)	0.52632(10/19)	0.61111(11/18)
Bo_TE_149541	0.85366(35/41)	0.29412(5/17)	0.00000(0/22)	0.00000(0/20)
Bo_TE_132994	0.16667(7/42)	0.17647(3/17)	0.77273(17/22)	0.00000(0/20)
Bo_TE_121088	0.16667(7/42)	0.22222(4/18)	0.14286(3/21)	0.77778(14/18)
Bo_TE_90311	0.20513(8/39)	0.77778(14/18)	0.95455(21/22)	0.95000(19/20)
Bo_TE_236110	0.00000(0/46)	0.00000(0/19)	0.76190(16/21)	0.00000(0/20)
Bo_TE_23297	0.61905(26/42)	0.11765(2/17)	0.61905(13/21)	0.73684(14/19)
Bo_TE_77477	0.20000(8/40)	0.41176(7/17)	0.50000(11/22)	0.89474(17/19)
Bo_TE_158966	0.50000(21/42)	0.12500(2/16)	0.95455(21/22)	0.78947(15/19)
Bo_TE_169494	0.13953(6/43)	0.00000(0/18)	0.80000(16/20)	0.30000(6/20)
Bo_TE_62434	0.00000(0/45)	0.05263(1/19)	0.80952(17/21)	0.00000(0/20)
Bo_TE_109021	0.02273(1/44)	0.06250(1/16)	0.40000(8/20)	0.55000(11/20)
Bo_TE_159292	0.87805(36/41)	0.38889(7/18)	0.61905(13/21)	0.23529(4/17)
Bo_TE_30162	0.02273(1/44)	0.00000(0/19)	0.75000(15/20)	0.00000(0/20)
Bo_TE_201993	0.16279(7/43)	0.50000(9/18)	0.08696(2/23)	0.61111(11/18)
Bo_TE_16303	0.16667(7/42)	0.60000(9/15)	0.90476(19/21)	0.68421(13/19)
Bo_TE_144790	0.13636(6/44)	0.82353(14/17)	1.00000(22/22)	0.42105(8/19)
Bo_TE_153241	0.97436(38/39)	0.31250(5/16)	0.95000(19/20)	0.94737(18/19)
Bo_TE_124968	0.42500(17/40)	0.05882(1/17)	0.55000(11/20)	0.77778(14/18)
Bo_TE_53146	0.73810(31/42)	0.26667(4/15)	0.13043(3/23)	0.94444(17/18)
Bo_TE_37489	0.97826(45/46)	1.00000(16/16)	0.59091(13/22)	0.31579(6/19)
Bo_TE_171145	0.46512(20/43)	0.72222(13/18)	0.60000(12/20)	0.15000(3/20)
Bo_TE_92192	0.09524(4/42)	0.38889(7/18)	0.04348(1/23)	0.63158(12/19)
Bo_TE_191480	0.70455(31/44)	0.00000(0/17)	0.00000(0/23)	0.00000(0/20)
Bo_TE_202257	0.84444(38/45)	0.36364(4/11)	0.09091(2/22)	0.05556(1/18)
Bo_TE_122381	0.36364(16/44)	0.11765(2/17)	0.59091(13/22)	0.63158(12/19)
Bo_TE_95005	0.76744(33/43)	0.16667(3/18)	0.00000(0/23)	0.05263(1/19)
Bo_TE_130675	0.33333(14/42)	0.16667(3/18)	0.95652(22/23)	0.95000(19/20)
Bo_TE_120960	0.69048(29/42)	0.63158(12/19)	0.40000(8/20)	0.90000(18/20)
Bo_TE_199713	0.16279(7/43)	0.29412(5/17)	0.65000(13/20)	0.75000(12/16)
Bo_TE_143122	1.00000(43/43)	0.76471(13/17)	0.30435(7/23)	1.00000(15/15)
Bo_TE_147959	0.88095(37/42)	0.76471(13/17)	0.14286(3/21)	0.00000(0/19)
Bo_TE_105816	0.95556(43/45)	0.38889(7/18)	0.59091(13/22)	0.26316(5/19)
Bo_TE_107845	0.80952(34/42)	0.27778(5/18)	0.00000(0/23)	0.00000(0/18)
Bo_TE_20353	0.84615(22/26)	0.27778(5/18)	0.76471(13/17)	0.82353(14/17)
Bo_TE_5019	0.56098(23/41)	0.00000(0/18)	0.00000(0/23)	0.00000(0/18)
Bo_TE_171201	0.53488(23/43)	0.23529(4/17)	0.42105(8/19)	0.85000(17/20)

Bo_TE_7994	0.04444(2/45)	0.00000(0/17)	0.57895(11/19)	0.57895(11/19)
Bo_TE_45548	0.00000(0/46)	0.11765(2/17)	0.54545(12/22)	0.10000(2/20)
Bo_TE_172677	0.18182(8/44)	0.00000(0/17)	0.14286(3/21)	0.52632(10/19)
Bo_TE_194692	0.60976(25/41)	0.21053(4/19)	0.17391(4/23)	0.05000(1/20)
Bo_TE_128547	0.27273(12/44)	0.72222(13/18)	0.55000(11/20)	0.00000(0/20)
Bo_TE_160708	0.02222(1/45)	0.00000(0/19)	0.71429(15/21)	0.45000(9/20)
Bo_TE_48217	0.55814(24/43)	0.00000(0/18)	0.00000(0/20)	0.00000(0/20)
Bo_TE_125831	0.26829(11/41)	0.11765(2/17)	0.33333(5/15)	0.63158(12/19)
Bo_TE_178800	0.25581(11/43)	0.25000(4/16)	0.47619(10/21)	0.89474(17/19)
Bo_TE_57334	0.00000(0/43)	0.00000(0/19)	0.21739(5/23)	0.68421(13/19)
Bo_TE_70216	0.25641(10/39)	0.47059(8/17)	0.20000(4/20)	0.78947(15/19)
Bo_TE_117497	0.95652(44/46)	0.18750(3/16)	0.56250(9/16)	0.61111(11/18)
Bo_TE_25478	0.64286(27/42)	0.64706(11/17)	0.61905(13/21)	0.10526(2/19)
Bo_TE_226333	0.25641(10/39)	0.83333(15/18)	0.00000(0/21)	0.05556(1/18)
Bo_TE_239681	0.15789(6/38)	0.55556(10/18)	1.00000(20/20)	0.90000(18/20)
Bo_TE_109715	0.71795(28/39)	0.68750(11/16)	0.54545(12/22)	0.00000(0/19)
Bo_TE_148892	0.00000(0/45)	0.00000(0/19)	0.60000(12/20)	0.55000(11/20)
Bo_TE_103205	0.88372(38/43)	0.23529(4/17)	0.57143(12/21)	0.05000(1/20)
Bo_TE_222840	0.58537(24/41)	0.06250(1/16)	0.00000(0/23)	0.00000(0/20)
Bo_TE_180208	0.85366(35/41)	0.00000(0/16)	0.00000(0/19)	0.00000(0/17)
Bo_TE_172104	0.69048(29/42)	0.12500(2/16)	0.85714(18/21)	0.55556(10/18)
Bo_TE_153710	0.64286(27/42)	0.00000(0/16)	0.00000(0/22)	0.00000(0/20)
Bo_TE_180135	0.00000(0/44)	0.50000(9/18)	0.00000(0/21)	0.15000(3/20)
Bo_TE_102909	0.46512(20/43)	0.35294(6/17)	1.00000(20/20)	0.30000(6/20)
Bo_TE_216710	0.04545(2/44)	0.00000(0/18)	0.40909(9/22)	0.55556(10/18)
Bo_TE_173718	0.02222(1/45)	0.00000(0/17)	0.21053(4/19)	0.63158(12/19)
Bo_TE_121413	0.15217(7/46)	0.82353(14/17)	0.04348(1/23)	0.00000(0/19)
Bo_TE_239693	0.83333(35/42)	0.41176(7/17)	0.00000(0/23)	0.00000(0/18)
Bo_TE_89498	0.60465(26/43)	0.66667(12/18)	0.33333(7/21)	0.05556(1/18)
Bo_TE_114306	0.00000(0/46)	0.00000(0/19)	0.04545(1/22)	0.73684(14/19)
Bo_TE_208373	0.23810(10/42)	0.33333(5/15)	0.77273(17/22)	0.05263(1/19)
Bo_TE_107786	0.02174(1/46)	0.68421(13/19)	0.00000(0/22)	0.05000(1/20)
Bo_TE_48364	0.80000(36/45)	0.00000(0/18)	0.00000(0/22)	0.22222(4/18)
Bo_TE_135220	0.15217(7/46)	0.55556(10/18)	0.44444(8/18)	0.00000(0/20)
Bo_TE_69203	1.00000(42/42)	0.38889(7/18)	0.68182(15/22)	0.84211(16/19)
Bo_TE_180179	0.00000(0/43)	0.00000(0/18)	0.72727(16/22)	0.75000(15/20)
Bo_TE_28350	0.02326(1/43)	0.22222(4/18)	0.66667(14/21)	0.47368(9/19)
Bo_TE_148163	0.12195(5/41)	0.82353(14/17)	0.57895(11/19)	0.00000(0/20)
Bo_TE_5684	0.23256(10/43)	0.64706(11/17)	0.91304(21/23)	0.81250(13/16)
Bo_TE_122855	0.00000(0/44)	0.61111(11/18)	0.00000(0/21)	0.05263(1/19)
Bo_TE_156895	0.31111(14/45)	0.00000(0/17)	0.72727(16/22)	0.16667(3/18)
Bo_TE_165663	0.11364(5/44)	0.16667(3/18)	0.55000(11/20)	0.00000(0/20)
Bo_TE_195817	0.93478(43/46)	0.36842(7/19)	0.95455(21/22)	0.25000(5/20)
Bo_TE_223416	0.97561(40/41)	0.38462(5/13)	0.45000(9/20)	0.33333(6/18)
Bo_TE_49929	0.02326(1/43)	0.82353(14/17)	0.86957(20/23)	0.47368(9/19)
Bo_TE_235371	0.69767(30/43)	0.25000(4/16)	0.00000(0/23)	0.10000(2/20)
Bo_TE_165294	0.45238(19/42)	1.00000(16/16)	0.85714(18/21)	0.90000(18/20)
Bo_TE_219262	0.35897(14/39)	1.00000(17/17)	0.63158(12/19)	0.77778(14/18)
Bo_TE_150865	0.00000(0/43)	0.00000(0/19)	0.61905(13/21)	0.10000(2/20)
Bo_TE_108394	0.04762(2/42)	0.00000(0/17)	0.68421(13/19)	0.16667(3/18)
Bo_TE_39821	0.00000(0/44)	0.00000(0/14)	0.70000(14/20)	0.85000(17/20)
Bo_TE_180925	0.58537(24/41)	0.26316(5/19)	0.66667(14/21)	0.06250(1/16)
Bo_TE_134714	0.00000(0/43)	0.56250(9/16)	0.04545(1/22)	0.00000(0/19)
Bo_TE_67940	0.04444(2/45)	0.00000(0/18)	0.36364(8/22)	0.85000(17/20)
Bo_TE_121846	0.40000(16/40)	0.05263(1/19)	0.35000(7/20)	0.78947(15/19)
Bo_TE_137129	0.48837(21/43)	0.83333(15/18)	1.00000(23/23)	0.68421(13/19)

Bo_TE_145597	0.09091(4/44)	0.57895(11/19)	0.30000(6/20)	0.70588(12/17)
Bo_TE_240746	0.83784(31/37)	0.27778(5/18)	0.31818(7/22)	0.15000(3/20)
Bo_TE_216070	0.70000(28/40)	0.62500(10/16)	0.05000(1/20)	0.50000(9/18)
Bo_TE_225029	0.97674(42/43)	0.52632(10/19)	0.72222(13/18)	0.27778(5/18)
Bo_TE_221486	0.85000(34/40)	0.46667(7/15)	0.40909(9/22)	0.10000(2/20)
Bo_TE_39049	0.04348(2/46)	0.00000(0/15)	0.09091(2/22)	0.89474(17/19)
Bo_TE_87270	0.08889(4/45)	0.15789(3/19)	0.23810(5/21)	0.80000(16/20)
Bo_TE_33980	0.31818(14/44)	0.50000(8/16)	0.23810(5/21)	0.85000(17/20)
Bo_TE_20019	0.20930(9/43)	0.88889(16/18)	0.52381(11/21)	0.90000(18/20)
Bo_TE_122053	0.56098(23/41)	0.00000(0/3)	1.00000(23/23)	0.31579(6/19)
Bo_TE_163329	0.00000(0/44)	0.50000(9/18)	0.00000(0/23)	0.00000(0/17)
Bo_TE_71213	0.04545(2/44)	0.52632(10/19)	0.09091(2/22)	0.76471(13/17)
Bo_TE_41153	0.06667(3/45)	0.00000(0/18)	0.04545(1/22)	0.75000(15/20)
Bo_TE_173221	0.05128(2/39)	0.56250(9/16)	0.52632(10/19)	0.56250(9/16)
Bo_TE_131825	0.04545(2/44)	0.22222(4/18)	0.66667(14/21)	0.61111(11/18)
Bo_TE_183018	0.52381(22/42)	0.16667(3/18)	0.60000(12/20)	0.90000(18/20)
Bo_TE_34932	0.24444(11/45)	0.00000(0/17)	0.76190(16/21)	0.47368(9/19)
Bo_TE_144853	0.75000(30/40)	0.05882(1/17)	0.00000(0/20)	0.61111(11/18)
Bo_TE_121503	0.00000(0/45)	0.76471(13/17)	0.00000(0/23)	0.10000(2/20)
Bo_TE_16226	0.58537(24/41)	0.56250(9/16)	0.40000(8/20)	1.00000(18/18)
Bo_TE_43178	0.09302(4/43)	0.64706(11/17)	0.00000(0/23)	0.00000(0/19)
Bo_TE_222229	0.94737(36/38)	0.18750(3/16)	1.00000(22/22)	0.22222(4/18)
Bo_TE_122203	0.56098(23/41)	0.58824(10/17)	0.04545(1/22)	0.78947(15/19)
Bo_TE_179546	0.80488(33/41)	0.87500(14/16)	0.00000(0/22)	0.41176(7/17)
Bo_TE_44355	0.86842(33/38)	0.94118(16/17)	0.42857(9/21)	0.73684(14/19)
Bo_TE_19849	0.93182(41/44)	0.76471(13/17)	0.30000(6/20)	0.52632(10/19)
Bo_TE_45985	0.02174(1/46)	0.10526(2/19)	0.00000(0/21)	0.50000(10/20)
Bo_TE_110947	0.58140(25/43)	0.87500(14/16)	0.33333(7/21)	0.70000(14/20)
Bo_TE_134993	0.07500(3/40)	0.72222(13/18)	0.00000(0/22)	0.00000(0/20)
Bo_TE_210922	0.50000(21/42)	0.05556(1/18)	0.00000(0/23)	0.00000(0/18)
Bo_TE_37228	0.87805(36/41)	0.43750(7/16)	1.00000(21/21)	0.95000(19/20)
Bo_TE_45649	0.62222(28/45)	0.00000(0/18)	0.23810(5/21)	0.00000(0/20)
Bo_TE_1502	0.21951(9/41)	0.81250(13/16)	0.61905(13/21)	1.00000(19/19)
Bo_TE_231729	0.66667(28/42)	0.52941(9/17)	0.66667(14/21)	0.15789(3/19)
Bo_TE_89522	0.59091(26/44)	0.64706(11/17)	0.35000(7/20)	0.05263(1/19)
Bo_TE_178349	0.02273(1/44)	0.00000(0/19)	0.00000(0/23)	0.50000(9/18)
Bo_TE_86283	0.18182(8/44)	0.76471(13/17)	0.71429(15/21)	0.10526(2/19)
Bo_TE_131144	0.25000(11/44)	0.00000(0/17)	0.80952(17/21)	0.47368(9/19)
Bo_TE_122909	0.95349(41/43)	0.35294(6/17)	0.25000(5/20)	0.15789(3/19)
Bo_TE_108196	0.02174(1/46)	0.29412(5/17)	0.70000(14/20)	0.38889(7/18)
Bo_TE_94375	0.00000(0/45)	0.11111(2/18)	0.40000(8/20)	0.75000(15/20)
Bo_TE_194519	0.59524(25/42)	0.29412(5/17)	0.91304(21/23)	0.38889(7/18)
Bo_TE_106101	0.62222(28/45)	0.00000(0/19)	0.09091(2/22)	0.05556(1/18)
Bo_TE_76366	0.32558(14/43)	0.94118(16/17)	0.22727(5/22)	0.55556(10/18)
Bo_TE_217657	0.00000(0/45)	0.00000(0/17)	0.00000(0/23)	0.58824(10/17)
Bo_TE_22184	0.48780(20/41)	0.93750(15/16)	0.36842(7/19)	0.84211(16/19)
Bo_TE_160080	0.48718(19/39)	0.00000(0/18)	0.63158(12/19)	0.78947(15/19)
Bo_TE_216466	0.64286(27/42)	0.05556(1/18)	0.80952(17/21)	0.63158(12/19)
Bo_TE_143483	0.85714(36/42)	0.35294(6/17)	0.75000(15/20)	0.85000(17/20)
Bo_TE_135867	0.02222(1/45)	0.00000(0/19)	0.26316(5/19)	0.65000(13/20)
Bo_TE_62709	0.64286(27/42)	0.17647(3/17)	1.00000(23/23)	0.95000(19/20)
Bo_TE_163014	0.79545(35/44)	0.62500(10/16)	0.31579(6/19)	0.05000(1/20)
Bo_TE_55715	0.04444(2/45)	0.00000(0/18)	0.08696(2/23)	0.85000(17/20)
Bo_TE_91492	0.92683(38/41)	0.55556(10/18)	0.27273(6/22)	0.76923(10/13)
Bo_TE_169868	0.62222(28/45)	0.22222(4/18)	0.00000(0/22)	0.05556(1/18)
Bo_TE_85199	0.32500(13/40)	0.77778(14/18)	0.23810(5/21)	0.10000(2/20)

Bo_TE_160548	0.07692(3/39)	0.06250(1/16)	0.70000(14/20)	0.15000(3/20)
Bo_TE_63581	0.09302(4/43)	0.76471(13/17)	0.22727(5/22)	0.00000(0/20)
Bo_TE_185391	1.00000(44/44)	0.43750(7/16)	0.70588(12/17)	0.89474(17/19)
Bo_TE_48313	0.84783(39/46)	0.21053(4/19)	0.47059(8/17)	0.36842(7/19)
Bo_TE_21147	0.15217(7/46)	0.71429(10/14)	0.52381(11/21)	0.84211(16/19)
Bo_TE_188944	0.34091(15/44)	0.18750(3/16)	0.80952(17/21)	0.95000(19/20)
Bo_TE_78795	0.18605(8/43)	0.00000(0/18)	0.14286(3/21)	0.70000(14/20)
Bo_TE_56785	0.86364(38/44)	0.56250(9/16)	0.30000(6/20)	0.10526(2/19)
Bo_TE_136693	0.40476(17/42)	0.68750(11/16)	1.00000(22/22)	0.89474(17/19)
Bo_TE_96698	0.63636(28/44)	0.33333(6/18)	0.42857(9/21)	0.95000(19/20)
Bo_TE_151675	0.90909(40/44)	0.88235(15/17)	0.47619(10/21)	0.35000(7/20)
Bo_TE_149052	0.30303(10/33)	0.44444(8/18)	1.00000(22/22)	1.00000(19/19)
Bo_TE_177454	0.30000(12/40)	0.43750(7/16)	0.04762(1/21)	0.55000(11/20)
Bo_TE_182242	0.04651(2/43)	0.61111(11/18)	0.00000(0/21)	0.25000(5/20)
Bo_TE_204875	0.33333(13/39)	0.77778(14/18)	0.52381(11/21)	0.05000(1/20)
Bo_TE_154756	0.64865(24/37)	0.18750(3/16)	0.04348(1/23)	0.00000(0/20)
Bo_TE_96025	0.30952(13/42)	0.50000(9/18)	1.00000(20/20)	1.00000(19/19)
Bo_TE_178664	0.26190(11/42)	0.12500(2/16)	0.42105(8/19)	0.94737(18/19)
Bo_TE_214122	0.80000(32/40)	0.25000(4/16)	0.80952(17/21)	0.47368(9/19)
Bo_TE_96778	0.00000(0/45)	0.29412(5/17)	0.80000(16/20)	0.00000(0/20)
Bo_TE_171608	0.59524(25/42)	0.27778(5/18)	0.00000(0/23)	0.15000(3/20)
Bo_TE_142595	0.40000(18/45)	0.77778(14/18)	0.95652(22/23)	0.50000(10/20)
Bo_TE_103321	0.25581(11/43)	0.05556(1/18)	0.68182(15/22)	0.10000(2/20)
Bo_TE_181364	0.28261(13/46)	0.56250(9/16)	1.00000(22/22)	0.57895(11/19)
Bo_TE_8658	0.04545(2/44)	0.00000(0/18)	0.57895(11/19)	0.73684(14/19)
Bo_TE_197349	0.00000(0/45)	0.11111(2/18)	0.00000(0/21)	0.52941(9/17)
Bo_TE_200206	0.20513(8/39)	0.47059(8/17)	0.45455(10/22)	0.75000(15/20)
Bo_TE_80621	0.18182(6/33)	0.17647(3/17)	0.58824(10/17)	0.89474(17/19)
Bo_TE_39672	0.33333(12/36)	0.25000(4/16)	0.22727(5/22)	0.90000(18/20)
Bo_TE_216836	0.46154(18/39)	1.00000(15/15)	1.00000(22/22)	0.94118(16/17)
Bo_TE_234332	0.86842(33/38)	0.47059(8/17)	0.40000(8/20)	0.31579(6/19)
Bo_TE_66952	0.02222(1/45)	0.68750(11/16)	0.45455(10/22)	0.72222(13/18)
Bo_TE_58338	0.21429(9/42)	0.00000(0/19)	0.59091(13/22)	0.16667(3/18)
Bo_TE_121622	0.51220(21/41)	0.82353(14/17)	0.61905(13/21)	0.15789(3/19)
Bo_TE_103845	1.00000(45/45)	0.70588(12/17)	0.42105(8/19)	0.16667(3/18)
Bo_TE_206984	0.63415(26/41)	0.58824(10/17)	0.00000(0/20)	0.21053(4/19)
Bo_TE_155843	0.04651(2/43)	0.50000(9/18)	0.04545(1/22)	0.00000(0/19)
Bo_TE_34922	0.71429(30/42)	1.00000(16/16)	0.25000(5/20)	0.45000(9/20)
Bo_TE_178796	0.57143(24/42)	0.83333(15/18)	0.38095(8/21)	0.05882(1/17)
Bo_TE_131128	0.20930(9/43)	0.00000(0/18)	0.82609(19/23)	0.45000(9/20)
Bo_TE_89401	0.65909(29/44)	0.38889(7/18)	0.09091(2/22)	0.00000(0/20)
Bo_TE_195389	0.78571(33/42)	0.29412(5/17)	0.95455(21/22)	0.44444(8/18)
Bo_TE_83990	0.09524(4/42)	0.00000(0/17)	0.57143(12/21)	0.75000(15/20)
Bo_TE_155090	0.88372(38/43)	0.88889(16/18)	0.72727(16/22)	0.31579(6/19)
Bo_TE_148852	0.74359(29/39)	0.37500(6/16)	0.04762(1/21)	0.00000(0/18)
Bo_TE_184690	0.86047(37/43)	0.35294(6/17)	0.00000(0/22)	0.15000(3/20)
Bo_TE_113317	0.78049(32/41)	0.29412(5/17)	0.71429(15/21)	0.10526(2/19)
Bo_TE_180409	0.04348(2/46)	0.52941(9/17)	0.36364(8/22)	0.00000(0/19)
Bo_TE_115219	0.57143(24/42)	0.81250(13/16)	0.13636(3/22)	0.26316(5/19)
Bo_TE_234666	0.88372(38/43)	0.13333(2/15)	0.38095(8/21)	0.30000(6/20)
Bo_TE_188245	0.88889(40/45)	0.16667(3/18)	0.00000(0/22)	0.00000(0/20)
Bo_TE_47400	0.78049(32/41)	0.16667(3/18)	0.19048(4/21)	0.05000(1/20)
Bo_TE_213074	0.80000(36/45)	0.11765(2/17)	0.36364(8/22)	0.20000(4/20)
Bo_TE_102511	0.58974(23/39)	0.05556(1/18)	0.10000(2/20)	0.47368(9/19)
Bo_TE_13548	0.13636(6/44)	0.18750(3/16)	0.52381(11/21)	0.70000(14/20)
Bo_TE_96245	0.31818(14/44)	0.88889(16/18)	1.00000(23/23)	0.90000(18/20)

Bo_TE_220938	0.70000(28/40)	0.00000(0/19)	0.00000(0/23)	0.10526(2/19)
Bo_TE_88889	0.00000(0/45)	0.00000(0/18)	0.18182(4/22)	0.70000(14/20)
Bo_TE_27993	0.81818(36/44)	0.11765(2/17)	0.52632(10/19)	0.10000(2/20)
Bo_TE_58902	0.11364(5/44)	0.80000(12/15)	0.95000(19/20)	0.68421(13/19)
Bo_TE_210972	0.02381(1/42)	0.37500(6/16)	0.57143(12/21)	0.52941(9/17)
Bo_TE_149406	0.04651(2/43)	0.00000(0/17)	0.80000(16/20)	0.00000(0/20)
Bo_TE_206519	0.04444(2/45)	0.00000(0/19)	0.04545(1/22)	0.61111(11/18)
Bo_TE_158612	0.14286(6/42)	0.06667(1/15)	0.85000(17/20)	0.17647(3/17)
Bo_TE_153300	0.69767(30/43)	0.29412(5/17)	0.13043(3/23)	0.73684(14/19)
Bo_TE_101754	0.02500(1/40)	0.50000(9/18)	0.00000(0/22)	1.00000(12/12)
Bo_TE_222346	0.08824(3/34)	0.05882(1/17)	0.63636(14/22)	0.16667(3/18)
Bo_TE_45970	0.55814(24/43)	0.12500(2/16)	0.00000(0/20)	0.23529(4/17)
Bo_TE_235291	0.39535(17/43)	0.17647(3/17)	1.00000(20/20)	0.84211(16/19)
Bo_TE_179901	0.83721(36/43)	0.44444(8/18)	0.33333(7/21)	0.00000(0/18)
Bo_TE_41932	0.27273(12/44)	0.88889(16/18)	0.90000(18/20)	0.85000(17/20)
Bo_TE_156157	0.33333(14/42)	0.82353(14/17)	1.00000(22/22)	0.85000(17/20)
Bo_TE_84084	0.15556(7/45)	0.87500(14/16)	0.19048(4/21)	0.30000(6/20)
Bo_TE_49017	0.00000(0/44)	0.00000(0/16)	0.57143(12/21)	0.21053(4/19)
Bo_TE_230914	0.84444(38/45)	0.89474(17/19)	0.00000(0/19)	0.00000(0/20)
Bo_TE_171471	0.68293(28/41)	0.55556(10/18)	0.21053(4/19)	0.00000(0/20)
Bo_TE_28532	0.80952(34/42)	0.78947(15/19)	0.00000(0/22)	0.05263(1/19)
Bo_TE_195469	0.23810(10/42)	0.23529(4/17)	0.71429(15/21)	0.75000(15/20)
Bo_TE_169627	0.00000(0/46)	0.00000(0/18)	0.09091(2/22)	0.84211(16/19)
Bo_TE_89386	0.31707(13/41)	0.58824(10/17)	0.10526(2/19)	1.00000(18/18)
Bo_TE_222295	0.07500(3/40)	0.18750(3/16)	0.81818(18/22)	0.68421(13/19)
Bo_TE_117819	0.75610(31/41)	0.17647(3/17)	0.60000(12/20)	0.85000(17/20)
Bo_TE_51949	0.41463(17/41)	0.94118(16/17)	1.00000(22/22)	1.00000(20/20)
Bo_TE_88846	0.64444(29/45)	0.25000(4/16)	0.86364(19/22)	0.50000(10/20)
Bo_TE_43326	0.91111(41/45)	0.35294(6/17)	1.00000(23/23)	1.00000(20/20)
Bo_TE_25458	0.00000(0/44)	0.05882(1/17)	0.38095(8/21)	0.89474(17/19)
Bo_TE_18114	0.33333(14/42)	0.58824(10/17)	0.25000(5/20)	0.85000(17/20)
Bo_TE_207463	0.06818(3/44)	0.35294(6/17)	0.27778(5/18)	0.66667(12/18)
Bo_TE_240438	0.75000(33/44)	0.25000(4/16)	0.35000(7/20)	0.50000(10/20)
Bo_TE_32145	0.06977(3/43)	0.78947(15/19)	0.08696(2/23)	0.05000(1/20)
Bo_TE_202245	0.14634(6/41)	0.35294(6/17)	0.90909(20/22)	0.89474(17/19)
Bo_TE_96881	0.58140(25/43)	0.00000(0/19)	0.00000(0/22)	0.06250(1/16)
Bo_TE_143183	0.91111(41/45)	0.88889(16/18)	0.63636(14/22)	0.15000(3/20)
Bo_TE_163566	0.20455(9/44)	0.05263(1/19)	0.18182(4/22)	0.63158(12/19)
Bo_TE_97971	0.00000(0/44)	0.11765(2/17)	0.61905(13/21)	0.20000(4/20)
Bo_TE_48824	0.00000(0/43)	0.17647(3/17)	0.52381(11/21)	0.35000(7/20)
Bo_TE_50631	0.00000(0/45)	0.00000(0/19)	0.08696(2/23)	0.84211(16/19)
Bo_TE_151837	0.08696(4/46)	0.76471(13/17)	0.16667(3/18)	0.35294(6/17)
Bo_TE_113750	0.88095(37/42)	0.55556(10/18)	0.09524(2/21)	0.16667(3/18)
Bo_TE_238476	0.60000(24/40)	0.05263(1/19)	0.00000(0/22)	0.00000(0/18)
Bo_TE_143524	0.20455(9/44)	0.41176(7/17)	1.00000(22/22)	0.95000(19/20)
Bo_TE_198053	0.51282(20/39)	0.00000(0/18)	0.00000(0/22)	0.00000(0/19)
Bo_TE_15445	0.26829(11/41)	0.93750(15/16)	1.00000(23/23)	0.41176(7/17)
Bo_TE_102694	0.11111(5/45)	0.84211(16/19)	0.38095(8/21)	0.00000(0/20)
Bo_TE_165531	0.02222(1/45)	0.00000(0/17)	0.33333(7/21)	0.60000(12/20)
Bo_TE_71935	0.75610(31/41)	0.56250(9/16)	0.18182(4/22)	0.61111(11/18)
Bo_TE_195402	0.77273(34/44)	0.25000(4/16)	1.00000(21/21)	0.50000(9/18)
Bo_TE_138000	0.00000(0/41)	0.00000(0/18)	0.00000(0/22)	0.55000(11/20)
Bo_TE_230966	0.46512(20/43)	0.70588(12/17)	0.00000(0/23)	0.00000(0/20)
Bo_TE_180027	0.06667(3/45)	0.00000(0/19)	0.10000(2/20)	0.68421(13/19)
Bo_TE_97755	0.25000(10/40)	1.00000(18/18)	1.00000(22/22)	0.95000(19/20)
Bo_TE_41979	0.11905(5/42)	0.36842(7/19)	0.68421(13/19)	0.16667(3/18)

Bo_TE_113759	0.82500(33/40)	0.61111(11/18)	0.05000(1/20)	0.11765(2/17)
Bo_TE_152376	0.80000(36/45)	0.10526(2/19)	0.47619(10/21)	0.10526(2/19)
Bo_TE_6999	0.06522(3/46)	0.00000(0/17)	0.54545(12/22)	0.65000(13/20)
Bo_TE_101805	0.70455(31/44)	0.47059(8/17)	0.21739(5/23)	0.15789(3/19)
Bo_TE_221966	0.35714(15/42)	0.82353(14/17)	0.26667(4/15)	0.05000(1/20)
Bo_TE_39029	0.02273(1/44)	0.00000(0/18)	0.08696(2/23)	0.88889(16/18)
Bo_TE_24153	0.45238(19/42)	0.68750(11/16)	0.60000(12/20)	0.10000(2/20)
Bo_TE_43686	0.16667(7/42)	0.38889(7/18)	1.00000(22/22)	0.57895(11/19)
Bo_TE_33014	0.16279(7/43)	0.81250(13/16)	0.91304(21/23)	0.75000(15/20)
Bo_TE_112244	0.00000(0/44)	0.52632(10/19)	0.00000(0/21)	0.10526(2/19)
Bo_TE_213977	0.35000(14/40)	0.25000(4/16)	0.60000(12/20)	0.94444(17/18)
Bo_TE_174560	0.40476(17/42)	0.60000(9/15)	0.04348(1/23)	0.75000(15/20)
Bo_TE_111070	0.35556(16/45)	0.70588(12/17)	0.00000(0/23)	0.21053(4/19)
Bo_TE_117652	0.64286(27/42)	0.33333(6/18)	0.30435(7/23)	0.05000(1/20)
Bo_TE_19901	0.24390(10/41)	0.81250(13/16)	0.00000(0/22)	0.05263(1/19)
Bo_TE_95172	0.60000(24/40)	0.00000(0/18)	0.00000(0/23)	0.00000(0/20)
Bo_TE_215433	0.35714(15/42)	0.72222(13/18)	0.86364(19/22)	0.00000(0/18)
Bo_TE_124887	0.26667(12/45)	0.50000(8/16)	0.09091(2/22)	0.88235(15/17)
Bo_TE_236603	0.76190(32/42)	1.00000(17/17)	0.95455(21/22)	0.45000(9/20)
Bo_TE_14618	0.72500(29/40)	0.64706(11/17)	0.18182(4/22)	0.05263(1/19)
Bo_TE_127049	0.73810(31/42)	0.64706(11/17)	0.10526(2/19)	0.61111(11/18)
Bo_TE_24702	0.06667(3/45)	0.86667(13/15)	0.26087(6/23)	0.60000(12/20)
Bo_TE_44150	0.86667(26/30)	1.00000(17/17)	0.95652(22/23)	0.50000(10/20)
Bo_TE_108146	0.97674(42/43)	0.41176(7/17)	0.81818(18/22)	1.00000(18/18)
Bo_TE_154311	0.02273(1/44)	0.82353(14/17)	0.00000(0/22)	0.00000(0/18)
Bo_TE_4076	0.81818(36/44)	0.37500(6/16)	0.04348(1/23)	0.11111(2/18)
Bo_TE_201346	0.53659(22/41)	0.37500(6/16)	0.80952(17/21)	0.94737(18/19)
Bo_TE_169467	0.63415(26/41)	0.33333(6/18)	0.00000(0/23)	0.00000(0/20)
Bo_TE_24475	0.36842(14/38)	0.15789(3/19)	1.00000(20/20)	0.88889(16/18)
Bo_TE_123996	0.76744(33/43)	0.94118(16/17)	0.90476(19/21)	0.16667(3/18)
Bo_TE_112743	0.21951(9/41)	0.35294(6/17)	0.75000(15/20)	1.00000(19/19)
Bo_TE_104861	0.62500(25/40)	0.31250(5/16)	0.00000(0/21)	0.00000(0/19)
Bo_TE_162089	0.91111(41/45)	0.94737(18/19)	0.57143(12/21)	0.15789(3/19)
Bo_TE_41862	0.23256(10/43)	0.68421(13/19)	0.95238(20/21)	0.93750(15/16)
Bo_TE_178111	0.31707(13/41)	0.58824(10/17)	1.00000(22/22)	0.27778(5/18)
Bo_TE_108691	0.92683(38/41)	0.17647(3/17)	0.66667(14/21)	0.80000(16/20)
Bo_TE_126170	0.25000(10/40)	0.64706(11/17)	0.35000(7/20)	0.05263(1/19)
Bo_TE_235265	0.00000(0/45)	0.05556(1/18)	0.52381(11/21)	0.10000(2/20)
Bo_TE_95258	0.18421(7/38)	0.75000(12/16)	0.57895(11/19)	0.20000(4/20)
Bo_TE_112758	0.25000(11/44)	0.60000(6/10)	1.00000(16/16)	1.00000(19/19)
Bo_TE_6154	0.97500(39/40)	0.80000(12/15)	0.30000(6/20)	0.58824(10/17)
Bo_TE_87248	0.00000(0/45)	0.00000(0/19)	0.17391(4/23)	0.78947(15/19)
Bo_TE_76236	0.12195(5/41)	0.00000(0/18)	0.10000(2/20)	0.50000(10/20)
Bo_TE_226789	0.62791(27/43)	0.23529(4/17)	0.50000(11/22)	0.78947(15/19)
Bo_TE_238528	0.79070(34/43)	1.00000(18/18)	0.25000(5/20)	0.11111(2/18)
Bo_TE_174367	0.00000(0/41)	0.15789(3/19)	0.18182(4/22)	0.73684(14/19)
Bo_TE_224736	0.00000(0/41)	0.00000(0/18)	0.65000(13/20)	0.15000(3/20)
Bo_TE_191330	1.00000(45/45)	0.47059(8/17)	0.85714(18/21)	0.11111(2/18)
Bo_TE_70282	0.23684(9/38)	0.41176(7/17)	0.09091(2/22)	0.73684(14/19)
Bo_TE_51667	0.26087(12/46)	0.60000(9/15)	0.77273(17/22)	0.63158(12/19)
Bo_TE_135881	0.00000(0/45)	0.00000(0/17)	0.23810(5/21)	0.72222(13/18)
Bo_TE_61263	0.82051(32/39)	0.58824(10/17)	0.19048(4/21)	0.31579(6/19)
Bo_TE_135264	0.84444(38/45)	0.43750(7/16)	0.55000(11/20)	1.00000(20/20)
Bo_TE_10817	0.02564(1/39)	0.18750(3/16)	0.08696(2/23)	0.84211(16/19)
Bo_TE_102717	0.52381(22/42)	0.00000(0/16)	0.04545(1/22)	0.22222(4/18)
Bo_TE_226851	0.48718(19/39)	0.50000(8/16)	0.00000(0/23)	0.35000(7/20)

Bo_TE_85963	0.83721(36/43)	0.75000(12/16)	0.61905(13/21)	0.27778(5/18)
Bo_TE_142646	0.29545(13/44)	0.05882(1/17)	0.66667(14/21)	0.30000(6/20)
Bo_TE_73086	0.09756(4/41)	0.57895(11/19)	0.28571(6/21)	0.60000(12/20)
Bo_TE_103754	0.85714(36/42)	0.35294(6/17)	0.68182(15/22)	0.31579(6/19)
Bo_TE_123402	0.00000(0/46)	0.50000(9/18)	0.80000(16/20)	0.60000(12/20)
Bo_TE_194772	0.11628(5/43)	0.05882(1/17)	0.71429(15/21)	0.23529(4/17)
Bo_TE_150638	0.29268(12/41)	0.61111(11/18)	0.80952(17/21)	0.10000(2/20)
Bo_TE_34819	0.75000(33/44)	1.00000(18/18)	0.38095(8/21)	0.42105(8/19)
Bo_TE_157939	0.75000(30/40)	0.23529(4/17)	0.00000(0/23)	0.05556(1/18)
Bo_TE_66485	0.41860(18/43)	0.31579(6/19)	0.85714(18/21)	0.50000(10/20)
Bo_TE_45737	0.72093(31/43)	0.63158(12/19)	0.00000(0/22)	0.78947(15/19)
Bo_TE_180924	0.59524(25/42)	0.17647(3/17)	0.57143(12/21)	0.05000(1/20)
Bo_TE_236472	0.13953(6/43)	0.76471(13/17)	0.00000(0/23)	0.20000(4/20)
Bo_TE_116974	0.02222(1/45)	0.63158(12/19)	0.04762(1/21)	0.00000(0/19)
Bo_TE_183507	0.00000(0/45)	0.00000(0/19)	0.61905(13/21)	0.00000(0/20)
Bo_TE_174997	0.00000(0/41)	0.00000(0/18)	0.04762(1/21)	0.66667(12/18)
Bo_TE_202656	0.97674(42/43)	0.66667(10/15)	0.73913(17/23)	0.33333(6/18)
Bo_TE_140983	0.00000(0/45)	0.00000(0/18)	0.70000(14/20)	0.00000(0/20)
Bo_TE_42852	1.00000(43/43)	1.00000(16/16)	0.15000(3/20)	0.20000(3/15)
Bo_TE_113661	0.50000(19/38)	0.55556(10/18)	0.04762(1/21)	0.05000(1/20)
Bo_TE_91343	0.76190(32/42)	0.16667(3/18)	0.00000(0/22)	0.00000(0/20)
Bo_TE_45045	0.27907(12/43)	0.35294(6/17)	0.80952(17/21)	0.15000(3/20)
Bo_TE_43453	0.12195(5/41)	0.47059(8/17)	1.00000(21/21)	0.52632(10/19)
Bo_TE_199093	0.13636(6/44)	0.70588(12/17)	0.65000(13/20)	0.00000(0/20)
Bo_TE_220809	1.00000(45/45)	0.40000(2/5)	0.75000(15/20)	0.31579(6/19)
Bo_TE_34724	0.42500(17/40)	0.00000(0/18)	0.86364(19/22)	0.40000(8/20)
Bo_TE_149345	0.04348(2/46)	0.00000(0/17)	0.78947(15/19)	0.00000(0/20)
Bo_TE_211218	0.00000(0/45)	0.05263(1/19)	0.00000(0/21)	0.58824(10/17)
Bo_TE_146901	0.73913(34/46)	0.76471(13/17)	0.15000(3/20)	0.00000(0/19)
Bo_TE_33037	0.04762(2/42)	0.77778(7/9)	0.85714(12/14)	0.80000(16/20)
Bo_TE_41906	0.78261(36/46)	0.33333(6/18)	0.04762(1/21)	0.15000(3/20)
Bo_TE_85774	1.00000(41/41)	0.68421(13/19)	0.86957(20/23)	0.50000(10/20)
Bo_TE_96591	0.31579(12/38)	0.94444(17/18)	0.42857(9/21)	0.63158(12/19)
Bo_TE_162027	0.53659(22/41)	0.21429(3/14)	0.00000(0/21)	0.05556(1/18)
Bo_TE_226680	0.64865(24/37)	0.94118(16/17)	0.34783(8/23)	0.90000(18/20)
Bo_TE_157004	0.34091(15/44)	0.12500(2/16)	0.68421(13/19)	0.31579(6/19)
Bo_TE_220495	0.00000(0/38)	0.10526(2/19)	0.45455(10/22)	0.78947(15/19)
Bo_TE_86821	0.10870(5/46)	0.72222(13/18)	0.77273(17/22)	0.68421(13/19)
Bo_TE_53584	0.97619(41/42)	1.00000(19/19)	0.25000(5/20)	0.10526(2/19)
Bo_TE_25113	0.76190(32/42)	0.33333(6/18)	0.00000(0/22)	0.00000(0/20)
Bo_TE_168803	0.32609(15/46)	0.71429(5/7)	0.95455(21/22)	0.89474(17/19)
Bo_TE_146466	0.80000(32/40)	0.56250(9/16)	0.28571(6/21)	0.63158(12/19)
Bo_TE_239406	0.78947(30/38)	0.35294(6/17)	0.10000(2/20)	0.52632(10/19)
Bo_TE_182527	0.04444(2/45)	0.38889(7/18)	0.63158(12/19)	0.27778(5/18)
Bo_TE_36882	0.40476(17/42)	0.43750(7/16)	0.81818(18/22)	1.00000(20/20)
Bo_TE_156376	0.75000(33/44)	0.05556(1/18)	0.33333(7/21)	0.00000(0/19)
Bo_TE_147773	0.67500(27/40)	0.53333(8/15)	0.00000(0/23)	0.89474(17/19)
Bo_TE_224667	0.41463(17/41)	0.78947(15/19)	0.35000(7/20)	0.88235(15/17)
Bo_TE_93988	0.97561(40/41)	0.47059(8/17)	0.05556(1/18)	0.10526(2/19)
Bo_TE_28982	0.14634(6/41)	0.11111(2/18)	0.78261(18/23)	0.15000(3/20)
Bo_TE_147758	0.81081(30/37)	0.22222(4/18)	0.70000(14/20)	0.95000(19/20)
Bo_TE_122833	0.24390(10/41)	0.76471(13/17)	0.27273(6/22)	0.05882(1/17)
Bo_TE_141612	0.81818(36/44)	0.06250(1/16)	0.04545(1/22)	0.21053(4/19)
Bo_TE_208247	0.67391(31/46)	0.50000(8/16)	0.00000(0/22)	0.10000(2/20)
Bo_TE_141256	0.84444(38/45)	0.05882(1/17)	0.10000(2/20)	0.21053(4/19)
Bo_TE_135125	0.45946(17/37)	0.92857(13/14)	0.10526(2/19)	0.94444(17/18)

Bo_TE_140197	0.11628(5/43)	0.52632(10/19)	0.57143(12/21)	1.00000(20/20)
Bo_TE_158133	0.85366(35/41)	0.12500(2/16)	0.89474(17/19)	0.58824(10/17)
Bo_TE_55919	0.73171(30/41)	0.00000(0/17)	0.00000(0/23)	0.00000(0/19)
Bo_TE_54312	0.00000(0/45)	0.18750(3/16)	0.59091(13/22)	0.65000(13/20)
Bo_TE_199572	0.06818(3/44)	0.00000(0/17)	0.80952(17/21)	0.00000(0/19)
Bo_TE_75302	0.42857(18/42)	0.76471(13/17)	0.00000(0/21)	0.57895(11/19)
Bo_TE_92012	0.16667(6/36)	0.11111(1/9)	1.00000(22/22)	0.36842(7/19)
Bo_TE_50901	0.22222(10/45)	0.11765(2/17)	0.27273(6/22)	0.63158(12/19)
Bo_TE_5133	0.18605(8/43)	0.42857(3/7)	0.89474(17/19)	0.75000(6/8)
Bo_TE_188205	0.33333(14/42)	0.43750(7/16)	0.22727(5/22)	0.83333(15/18)
Bo_TE_199028	0.83333(35/42)	0.27778(5/18)	0.00000(0/22)	0.00000(0/20)
Bo_TE_61149	0.73171(30/41)	0.29412(5/17)	0.04545(1/22)	0.05000(1/20)
Bo_TE_158856	0.57143(24/42)	0.17647(3/17)	0.36364(8/22)	0.05263(1/19)
Bo_TE_78642	0.85714(36/42)	0.82353(14/17)	0.36364(8/22)	0.10000(2/20)
Bo_TE_15459	0.29268(12/41)	0.05882(1/17)	0.00000(0/23)	0.52632(10/19)
Bo_TE_141395	0.81818(36/44)	0.05882(1/17)	0.00000(0/23)	0.05556(1/18)
Bo_TE_32106	0.06667(3/45)	0.82353(14/17)	0.08696(2/23)	0.05000(1/20)
Bo_TE_170131	0.42857(18/42)	0.66667(12/18)	1.00000(23/23)	0.60000(12/20)
Bo_TE_181997	0.86047(37/43)	0.89474(17/19)	0.04545(1/22)	0.41176(7/17)
Bo_TE_6618	0.26190(11/42)	0.06667(1/15)	0.61905(13/21)	0.10000(2/20)
Bo_TE_208505	0.77273(34/44)	0.47059(8/17)	0.42857(9/21)	0.94737(18/19)
Bo_TE_91999	0.65909(29/44)	0.00000(0/18)	0.00000(0/23)	0.63158(12/19)
Bo_TE_69140	0.45455(20/44)	0.00000(0/17)	0.59091(13/22)	0.05556(1/18)
Bo_TE_158458	0.13043(6/46)	0.50000(9/18)	0.00000(0/21)	0.00000(0/19)
Bo_TE_190863	0.02500(1/40)	0.70588(12/17)	0.04762(1/21)	0.26316(5/19)
Bo_TE_158314	0.93478(43/46)	0.87500(14/16)	0.14286(3/21)	0.26316(5/19)
Bo_TE_36551	0.27273(12/44)	1.00000(17/17)	0.65000(13/20)	0.78947(15/19)
Bo_TE_91476	1.00000(45/45)	0.84211(16/19)	0.20000(4/20)	0.55000(11/20)
Bo_TE_24416	0.84783(39/46)	0.05556(1/18)	0.08696(2/23)	0.05263(1/19)
Bo_TE_179118	0.30952(13/42)	0.56250(9/16)	0.70000(14/20)	0.06250(1/16)
Bo_TE_629	0.72727(32/44)	0.06250(1/16)	0.20000(4/20)	0.50000(8/16)
Bo_TE_154259	1.00000(43/43)	0.25000(4/16)	1.00000(21/21)	0.85000(17/20)
Bo_TE_67802	1.00000(40/40)	1.00000(11/11)	0.88235(15/17)	0.22222(4/18)
Bo_TE_33146	0.00000(0/43)	0.41176(7/17)	0.36364(8/22)	0.89474(17/19)
Bo_TE_112940	0.27500(11/40)	0.00000(0/15)	0.82609(19/23)	0.84211(16/19)
Bo_TE_142465	0.92683(38/41)	0.94118(16/17)	0.13636(3/22)	0.36842(7/19)
Bo_TE_69661	0.93333(42/45)	0.68750(11/16)	0.20000(4/20)	0.10526(2/19)
Bo_TE_128731	0.72500(29/40)	0.23529(4/17)	0.42857(9/21)	1.00000(19/19)
Bo_TE_154800	0.88372(38/43)	0.00000(0/17)	0.00000(0/16)	0.05556(1/18)
Bo_TE_8179	0.13514(5/37)	0.37500(6/16)	0.76190(16/21)	0.00000(0/20)
Bo_TE_16170	0.58537(24/41)	0.05556(1/18)	0.82353(14/17)	0.05000(1/20)
Bo_TE_205122	0.18605(8/43)	0.75000(12/16)	0.14286(2/14)	0.33333(1/3)
Bo_TE_76212	0.94872(37/39)	0.64706(11/17)	0.00000(0/18)	0.16667(3/18)
Bo_TE_238289	0.72500(29/40)	0.23529(4/17)	1.00000(23/23)	1.00000(20/20)
Bo_TE_49294	0.11628(5/43)	0.88235(15/17)	0.28571(6/21)	1.00000(18/18)
Bo_TE_21010	0.20455(9/44)	0.88235(15/17)	0.54545(12/22)	0.90000(18/20)
Bo_TE_140253	0.84615(33/39)	0.11765(2/17)	0.27273(6/22)	0.22222(4/18)
Bo_TE_33087	0.23077(9/39)	0.50000(7/14)	0.66667(14/21)	0.00000(0/16)
Bo_TE_180546	0.47727(21/44)	0.58824(10/17)	1.00000(23/23)	1.00000(20/20)
Bo_TE_222336	0.00000(0/46)	0.68421(13/19)	0.00000(0/23)	0.00000(0/20)
Bo_TE_103289	0.72727(32/44)	0.27778(5/18)	0.00000(0/23)	0.22222(4/18)
Bo_TE_101124	0.91111(41/45)	0.56250(9/16)	0.61905(13/21)	0.11111(2/18)
Bo_TE_115810	0.84444(38/45)	0.50000(9/18)	0.18182(4/22)	0.85000(17/20)
Bo_TE_55010	0.86667(39/45)	0.47059(8/17)	0.18182(4/22)	0.52632(10/19)
Bo_TE_94534	1.00000(40/40)	0.82353(14/17)	0.54545(12/22)	0.45000(9/20)
Bo_TE_159196	0.33333(13/39)	0.56250(9/16)	1.00000(22/22)	0.89474(17/19)

Bo_TE_121980	0.58537(24/41)	0.88889(16/18)	0.95652(22/23)	0.22222(4/18)
Bo_TE_78986	1.00000(45/45)	1.00000(19/19)	0.26316(5/19)	0.58824(10/17)
Bo_TE_184581	0.42857(18/42)	0.27778(5/18)	0.68421(13/19)	0.11765(2/17)
Bo_TE_21946	0.61364(27/44)	0.86667(13/15)	0.00000(0/11)	0.00000(0/19)
Bo_TE_109205	0.75862(22/29)	1.00000(17/17)	0.47368(9/19)	0.25000(5/20)
Bo_TE_239608	0.69231(27/39)	0.46667(7/15)	0.00000(0/22)	0.38889(7/18)
Bo_TE_107925	0.00000(0/43)	0.00000(0/19)	0.81818(18/22)	0.75000(15/20)
Bo_TE_129957	0.04545(2/44)	0.00000(0/17)	0.04545(1/22)	0.70588(12/17)
Bo_TE_143026	0.75000(33/44)	0.11765(2/17)	0.27273(6/22)	0.40000(8/20)
Bo_TE_134492	0.35135(13/37)	0.94118(16/17)	1.00000(22/22)	1.00000(20/20)
Bo_TE_196599	0.13333(6/45)	0.58824(10/17)	0.00000(0/23)	0.00000(0/20)
Bo_TE_222671	0.95238(40/42)	1.00000(18/18)	1.00000(23/23)	0.50000(10/20)
Bo_TE_184187	0.00000(0/46)	0.00000(0/19)	0.66667(14/21)	0.00000(0/20)
Bo_TE_216648	0.57500(23/40)	0.11111(2/18)	0.04545(1/22)	0.00000(0/20)
Bo_TE_69415	0.02222(1/45)	0.00000(0/18)	0.05000(1/20)	0.70000(14/20)
Bo_TE_97631	0.80488(33/41)	0.33333(6/18)	0.00000(0/22)	0.20000(4/20)
Bo_TE_126838	0.90909(40/44)	1.00000(19/19)	0.42857(9/21)	0.83333(15/18)
Bo_TE_143515	0.20000(9/45)	0.37500(6/16)	1.00000(23/23)	0.94737(18/19)
Bo_TE_99801	0.58974(23/39)	0.41176(7/17)	0.00000(0/23)	0.00000(0/20)
Bo_TE_24676	0.00000(0/44)	0.05556(1/18)	0.45000(9/20)	0.52632(10/19)
Bo_TE_215138	1.00000(46/46)	0.50000(9/18)	1.00000(23/23)	1.00000(19/19)
Bo_TE_36976	0.13953(6/43)	0.13333(2/15)	0.90000(18/20)	0.84211(16/19)
Bo_TE_200248	0.02273(1/44)	0.56250(9/16)	0.61905(13/21)	0.80000(16/20)
Bo_TE_179552	0.18605(8/43)	0.17647(3/17)	1.00000(20/20)	0.52632(10/19)
Bo_TE_69254	0.02564(1/39)	0.00000(0/16)	0.61905(13/21)	0.42105(8/19)
Bo_TE_94676	0.21429(9/42)	0.50000(9/18)	1.00000(21/21)	0.31579(6/19)
Bo_TE_13616	0.08889(4/45)	0.33333(5/15)	0.85714(18/21)	0.60000(12/20)
Bo_TE_216484	0.78571(33/42)	1.00000(18/18)	0.95238(20/21)	0.20000(4/20)
Bo_TE_39927	0.02326(1/43)	0.41176(7/17)	0.19048(4/21)	0.57895(11/19)
Bo_TE_173230	0.42857(18/42)	0.70588(12/17)	0.70000(14/20)	1.00000(18/18)
Bo_TE_33415	0.09091(4/44)	0.88235(15/17)	0.80952(17/21)	0.88889(16/18)
Bo_TE_151668	0.93333(42/45)	0.84211(16/19)	0.50000(11/22)	0.30000(6/20)
Bo_TE_58669	0.66667(26/39)	0.82353(14/17)	0.13043(3/23)	0.05000(1/20)
Bo_TE_140867	0.71429(30/42)	0.11765(2/17)	0.08696(2/23)	0.20000(4/20)
Bo_TE_142933	0.59091(26/44)	0.53333(8/15)	0.09091(2/22)	0.15000(3/20)
Bo_TE_163941	0.69048(29/42)	0.22222(4/18)	0.00000(0/23)	0.15000(3/20)
Bo_TE_23406	0.26667(12/45)	0.62500(10/16)	0.15000(3/20)	0.68421(13/19)
Bo_TE_130173	0.76316(29/38)	0.33333(5/15)	0.00000(0/23)	0.00000(0/18)
Bo_TE_136387	0.88095(37/42)	0.11111(2/18)	0.28571(6/21)	0.10000(2/20)
Bo_TE_79910	0.06977(3/43)	0.62500(10/16)	0.04545(1/22)	0.26316(5/19)
Bo_TE_146762	0.81395(35/43)	1.00000(19/19)	0.50000(10/20)	0.83333(15/18)
Bo_TE_136017	0.60465(26/43)	0.61111(11/18)	0.00000(0/23)	0.00000(0/19)
Bo_TE_150512	0.88372(38/43)	0.29412(5/17)	0.00000(0/23)	0.30000(6/20)
Bo_TE_39508	0.02381(1/42)	0.31250(5/16)	0.66667(14/21)	0.52632(10/19)
Bo_TE_165567	0.88636(39/44)	0.31250(5/16)	0.52632(10/19)	0.33333(6/18)
Bo_TE_193137	0.70455(31/44)	0.23529(4/17)	0.00000(0/22)	0.83333(15/18)
Bo_TE_239764	0.73171(30/41)	1.00000(16/16)	0.43478(10/23)	0.15789(3/19)
Bo_TE_136347	0.80000(32/40)	0.17647(3/17)	0.95238(20/21)	0.88889(16/18)
Bo_TE_24092	0.51282(20/39)	0.16667(3/18)	0.36842(7/19)	0.80000(16/20)
Bo_TE_128605	0.61905(26/42)	0.10526(2/19)	0.00000(0/21)	0.05000(1/20)
Bo_TE_18060	0.34146(14/41)	0.53333(8/15)	0.23810(5/21)	0.85000(17/20)
Bo_TE_39310	0.50000(22/44)	0.14286(2/14)	0.00000(0/22)	0.05556(1/18)
Bo_TE_204776	0.11905(5/42)	0.10526(2/19)	0.68182(15/22)	0.36842(7/19)
Bo_TE_62827	0.97727(43/44)	0.38889(7/18)	0.57143(12/21)	1.00000(19/19)
Bo_TE_162876	0.00000(0/44)	0.00000(0/18)	0.40000(8/20)	0.66667(12/18)
Bo_TE_141477	0.80952(34/42)	0.05882(1/17)	0.04545(1/22)	0.25000(5/20)

Bo_TE_3863	0.19048(8/42)	0.70588(12/17)	0.23810(5/21)	0.31579(6/19)
Bo_TE_38923	0.97727(43/44)	0.89474(17/19)	0.82609(19/23)	0.45000(9/20)
Bo_TE_116391	0.97727(43/44)	0.41176(7/17)	0.70000(14/20)	0.21053(4/19)
Bo_TE_232226	0.00000(0/45)	0.78947(15/19)	0.38095(8/21)	0.52632(10/19)
Bo_TE_142642	0.09756(4/41)	0.00000(0/18)	0.57143(12/21)	0.31579(6/19)
Bo_TE_80302	0.86364(38/44)	0.72222(13/18)	0.52381(11/21)	0.15789(3/19)
Bo_TE_169888	0.20455(9/44)	0.55556(10/18)	0.89474(17/19)	0.70000(14/20)
Bo_TE_101265	0.92857(39/42)	0.83333(15/18)	0.55000(11/20)	0.33333(6/18)
Bo_TE_204678	0.75000(33/44)	0.35294(6/17)	0.40000(8/20)	0.25000(5/20)
Bo_TE_81486	0.27907(12/43)	0.00000(0/17)	0.04545(1/22)	0.55000(11/20)
Bo_TE_155113	0.09302(4/43)	0.16667(3/18)	0.25000(5/20)	0.70000(14/20)
Bo_TE_89585	0.58140(25/43)	0.11765(2/17)	0.13636(3/22)	0.94737(18/19)
Bo_TE_35991	0.57500(23/40)	0.27778(5/18)	1.00000(22/22)	0.95000(19/20)
Bo_TE_141625	0.79545(35/44)	0.10526(2/19)	0.05263(1/19)	0.21053(4/19)
Bo_TE_18785	0.13514(5/37)	0.55556(10/18)	0.00000(0/21)	0.26316(5/19)
Bo_TE_236390	0.00000(0/43)	0.50000(8/16)	0.00000(0/23)	0.00000(0/19)
Bo_TE_67083	0.71795(28/39)	0.00000(0/19)	0.00000(0/23)	0.00000(0/19)
Bo_TE_89960	0.09524(4/42)	0.50000(9/18)	0.84211(16/19)	0.78947(15/19)
Bo_TE_197926	0.53659(22/41)	0.05263(1/19)	0.50000(10/20)	0.72222(13/18)
Bo_TE_206459	0.04348(2/46)	0.78947(15/19)	0.42857(9/21)	0.36842(7/19)
Bo_TE_39044	0.02500(1/40)	0.00000(0/15)	0.05556(1/18)	0.88235(15/17)
Bo_TE_164966	0.00000(0/45)	0.00000(0/18)	0.43478(10/23)	0.73684(14/19)
Bo_TE_211265	1.00000(37/37)	0.94118(16/17)	1.00000(18/18)	0.18750(3/16)
Bo_TE_8771	0.11905(5/42)	0.17647(3/17)	0.77273(17/22)	0.11111(2/18)
Bo_TE_122150	0.24390(10/41)	0.55556(10/18)	0.95455(21/22)	0.20000(4/20)
Bo_TE_134272	0.41463(17/41)	0.93750(15/16)	0.13636(3/22)	0.35000(7/20)
Bo_TE_145020	0.00000(0/46)	0.27778(5/18)	0.85000(17/20)	0.78947(15/19)
Bo_TE_190899	0.04545(2/44)	0.61111(11/18)	0.00000(0/22)	0.10000(2/20)
Bo_TE_32180	0.00000(0/46)	0.66667(12/18)	0.00000(0/23)	0.05000(1/20)
Bo_TE_179084	0.69767(30/43)	0.05882(1/17)	0.42857(9/21)	0.85000(17/20)
Bo_TE_22698	0.88095(37/42)	0.33333(6/18)	1.00000(23/23)	0.95000(19/20)
Bo_TE_188862	0.72727(32/44)	0.17647(3/17)	0.36364(8/22)	1.00000(20/20)
Bo_TE_45863	0.00000(0/46)	0.52632(10/19)	0.09091(2/22)	0.20000(4/20)
Bo_TE_153290	0.73333(33/45)	0.11111(2/18)	0.77778(14/18)	0.21053(4/19)
Bo_TE_99915	0.53333(24/45)	0.17647(3/17)	0.90476(19/21)	1.00000(19/19)
Bo_TE_235021	0.04444(2/45)	0.35294(6/17)	0.09091(2/22)	0.63158(12/19)
Bo_TE_88445	0.30952(13/42)	0.33333(5/15)	0.86957(20/23)	0.77778(14/18)
Bo_TE_224507	0.69048(29/42)	0.10526(2/19)	0.65000(13/20)	0.10526(2/19)
Bo_TE_206709	0.35000(14/40)	0.94118(16/17)	0.77778(14/18)	0.66667(12/18)
Bo_TE_87451	0.06667(3/45)	0.16667(3/18)	0.71429(15/21)	0.10526(2/19)
Bo_TE_229142	0.43590(17/39)	0.68750(11/16)	0.04348(1/23)	0.00000(0/20)
Bo_TE_121729	0.88889(40/45)	1.00000(19/19)	1.00000(22/22)	0.11111(2/18)
Bo_TE_239727	0.07143(3/42)	0.76471(13/17)	0.52381(11/21)	0.10000(2/20)
Bo_TE_77753	0.64286(27/42)	0.77778(14/18)	0.30000(6/20)	0.00000(0/20)
Bo_TE_73782	1.00000(44/44)	0.22222(4/18)	0.95652(22/23)	0.90000(18/20)
Bo_TE_95115	0.51220(21/41)	0.12500(2/16)	1.00000(22/22)	1.00000(19/19)
Bo_TE_238309	0.53659(22/41)	0.05556(1/18)	0.00000(0/23)	0.00000(0/20)
Bo_TE_100968	0.00000(0/43)	0.73684(14/19)	0.80952(17/21)	0.21053(4/19)
Bo_TE_216171	0.02381(1/42)	0.29412(5/17)	0.09091(2/22)	0.52632(10/19)
Bo_TE_149155	0.95556(43/45)	0.94118(16/17)	0.17391(4/23)	0.55000(11/20)
Bo_TE_178095	0.65854(27/41)	0.35294(6/17)	0.00000(0/23)	0.00000(0/7)
Bo_TE_168660	0.20930(9/43)	1.00000(18/18)	0.33333(7/21)	0.16667(3/18)
Bo_TE_216687	0.06818(3/44)	0.05263(1/19)	0.77778(14/18)	0.78947(15/19)
Bo_TE_25928	0.20930(9/43)	0.88889(16/18)	0.52381(11/21)	0.90000(18/20)
Bo_TE_43250	0.02273(1/44)	0.50000(9/18)	0.00000(0/23)	0.00000(0/19)
Bo_TE_219024	0.72222(26/36)	0.16667(3/18)	0.84211(16/19)	0.85000(17/20)

Bo_TE_101084	0.78049(32/41)	0.46667(7/15)	0.23810(5/21)	0.05556(1/18)
Bo_TE_68859	0.70455(31/44)	0.12500(2/16)	0.00000(0/23)	0.16667(3/18)
Bo_TE_4415	0.38636(17/44)	0.94737(18/19)	0.59091(13/22)	0.94737(18/19)
Bo_TE_121444	0.00000(0/44)	0.00000(0/18)	0.04545(1/22)	0.73684(14/19)
Bo_TE_52512	0.41860(18/43)	0.25000(4/16)	0.26316(5/19)	1.00000(18/18)
Bo_TE_83778	0.07143(3/42)	0.05556(1/18)	0.80000(16/20)	0.38889(7/18)
Bo_TE_87783	0.10870(5/46)	0.55556(10/18)	0.00000(0/23)	0.05000(1/20)
Bo_TE_41180	0.04545(2/44)	0.00000(0/18)	0.28571(6/21)	0.63158(12/19)
Bo_TE_154594	0.00000(0/45)	0.00000(0/18)	0.71429(15/21)	0.57895(11/19)
Bo_TE_2338	0.30769(12/39)	0.11765(2/17)	0.65000(13/20)	0.15000(3/20)
Bo_TE_213804	0.00000(0/44)	0.00000(0/18)	0.31818(7/22)	0.50000(9/18)
Bo_TE_49795	0.75610(31/41)	0.18750(3/16)	0.66667(14/21)	0.55556(10/18)
Bo_TE_177478	0.58140(25/43)	0.52941(9/17)	0.95455(21/22)	0.42105(8/19)
Bo_TE_143560	0.00000(0/44)	0.00000(0/17)	0.00000(0/21)	0.63158(12/19)
Bo_TE_168769	0.73171(30/41)	0.11111(1/9)	0.09091(2/22)	0.15789(3/19)
Bo_TE_65351	0.73810(31/42)	0.18750(3/16)	0.40000(8/20)	0.00000(0/20)
Bo_TE_216157	0.06977(3/43)	0.29412(5/17)	0.18182(4/22)	0.61111(11/18)
Bo_TE_50783	0.37500(15/40)	0.29412(5/17)	0.20000(4/20)	0.84211(16/19)
Bo_TE_43886	0.81395(35/43)	0.22222(4/18)	0.00000(0/23)	0.05000(1/20)
Bo_TE_60929	0.65854(27/41)	0.55556(10/18)	0.04762(1/21)	0.10526(2/19)
Bo_TE_98857	0.00000(0/45)	0.00000(0/19)	0.52174(12/23)	0.25000(5/20)
Bo_TE_120951	0.68421(26/38)	0.66667(12/18)	0.23810(5/21)	0.10000(2/20)
Bo_TE_95720	0.55000(22/40)	0.46667(7/15)	0.00000(0/23)	0.05263(1/19)
Bo_TE_223053	0.85714(36/42)	0.25000(4/16)	0.81818(18/22)	0.36842(7/19)
Bo_TE_25371	0.37500(15/40)	0.50000(9/18)	0.82609(19/23)	1.00000(17/17)
Bo_TE_170710	0.52381(22/42)	0.11111(2/18)	0.00000(0/23)	0.70000(14/20)
Bo_TE_197600	0.02326(1/43)	0.10526(2/19)	0.17391(4/23)	0.61111(11/18)
Bo_TE_75743	0.02326(1/43)	0.11765(2/17)	0.00000(0/22)	0.61111(11/18)
Bo_TE_80204	0.00000(0/44)	0.00000(0/19)	0.04762(1/21)	0.73684(14/19)
Bo_TE_57670	0.46512(20/43)	0.41176(7/17)	0.35000(7/20)	1.00000(20/20)
Bo_TE_119834	0.24390(10/41)	0.88889(16/18)	1.00000(8/8)	0.73684(14/19)
Bo_TE_105061	0.65789(25/38)	1.00000(15/15)	0.00000(0/22)	0.05882(1/17)
Bo_TE_191810	0.62791(27/43)	0.41176(7/17)	0.04348(1/23)	0.56250(9/16)
Bo_TE_61700	0.30952(13/42)	0.38889(7/18)	0.71429(15/21)	0.84211(16/19)
Bo_TE_210038	0.50000(20/40)	0.94444(17/18)	1.00000(23/23)	0.78947(15/19)
Bo_TE_138272	0.00000(0/35)	0.00000(0/9)	0.14286(3/21)	0.89474(17/19)
Bo_TE_192196	0.41860(18/43)	0.28571(4/14)	0.00000(0/23)	0.85000(17/20)
Bo_TE_219251	0.21429(9/42)	0.70588(12/17)	0.13043(3/23)	0.00000(0/20)
Bo_TE_215869	0.00000(0/46)	0.00000(0/19)	0.13043(3/23)	0.50000(10/20)
Bo_TE_10370	0.36364(16/44)	0.88235(15/17)	0.57143(12/21)	0.84211(16/19)
Bo_TE_177450	0.58537(24/41)	0.56250(9/16)	0.95238(20/21)	0.42105(8/19)
Bo_TE_212313	0.97674(42/43)	0.56250(9/16)	0.42857(9/21)	0.90000(18/20)
Bo_TE_123838	0.00000(0/46)	0.72222(13/18)	0.00000(0/22)	0.00000(0/18)
Bo_TE_86047	0.44444(20/45)	0.00000(0/17)	0.76190(16/21)	0.05263(1/19)
Bo_TE_156637	0.61364(27/44)	0.08333(1/12)	0.95455(21/22)	0.10526(2/19)
Bo_TE_198525	0.88636(39/44)	0.68421(13/19)	0.61905(13/21)	0.05263(1/19)
Bo_TE_230434	0.26190(11/42)	0.82353(14/17)	1.00000(23/23)	0.26316(5/19)
Bo_TE_158606	0.37209(16/43)	0.94118(16/17)	0.13043(3/23)	0.64706(11/17)
Bo_TE_186331	0.11364(5/44)	0.76471(13/17)	0.63636(14/22)	0.58824(10/17)
Bo_TE_129199	0.80000(32/40)	0.06250(1/16)	0.77273(17/22)	0.65000(13/20)
Bo_TE_179969	0.62500(25/40)	0.10526(2/19)	0.00000(0/22)	0.10526(2/19)
Bo_TE_185925	0.43478(20/46)	0.50000(8/16)	0.21739(5/23)	0.72222(13/18)
Bo_TE_136893	0.47500(19/40)	0.12500(2/16)	0.71429(15/21)	0.68421(13/19)
Bo_TE_209511	0.90909(40/44)	0.18750(3/16)	0.91304(21/23)	0.73684(14/19)
Bo_TE_57631	0.56818(25/44)	0.56250(9/16)	0.40000(8/20)	0.00000(0/19)
Bo_TE_154822	0.86047(37/43)	0.00000(0/17)	0.00000(0/22)	0.05000(1/20)

Bo_TE_21606	0.47500(19/40)	0.82353(14/17)	0.09091(2/22)	0.50000(10/20)
Bo_TE_227764	0.84091(37/44)	0.88889(16/18)	0.45455(10/22)	0.05000(1/20)
Bo_TE_140295	0.32558(14/43)	0.17647(3/17)	0.63636(14/22)	0.00000(0/19)
Bo_TE_140398	0.73810(31/42)	0.47059(8/17)	0.42857(9/21)	0.00000(0/19)
Bo_TE_209325	0.44444(16/36)	0.62500(10/16)	0.00000(0/23)	0.00000(0/19)
Bo_TE_239561	0.83784(31/37)	0.37500(6/16)	0.00000(0/23)	0.33333(6/18)
Bo_TE_210178	0.15385(6/39)	0.82353(14/17)	0.72727(16/22)	0.63158(12/19)
Bo_TE_33050	0.95349(41/43)	0.18750(3/16)	1.00000(23/23)	0.85000(17/20)
Bo_TE_230239	0.29268(12/41)	0.18750(3/16)	0.95455(21/22)	0.88889(16/18)
Bo_TE_48817	0.80435(37/46)	0.70588(12/17)	0.42105(8/19)	0.26316(5/19)
Bo_TE_57194	1.00000(43/43)	1.00000(19/19)	0.77273(17/22)	0.47368(9/19)
Bo_TE_45436	0.57500(23/40)	0.00000(0/17)	0.18182(4/22)	0.00000(0/20)
Bo_TE_211525	0.00000(0/46)	0.00000(0/18)	0.81818(18/22)	0.00000(0/20)
Bo_TE_160541	0.09091(4/44)	0.12500(2/16)	0.71429(15/21)	0.15000(3/20)
Bo_TE_117697	0.63636(28/44)	0.35294(6/17)	0.33333(7/21)	0.05263(1/19)
Bo_TE_178290	0.73684(28/38)	0.35294(6/17)	0.00000(0/20)	0.10000(2/20)
Bo_TE_138865	0.28889(13/45)	0.16667(3/18)	0.45000(9/20)	0.73684(14/19)
Bo_TE_214142	0.00000(0/45)	0.55556(10/18)	0.15789(3/19)	0.00000(0/20)
Bo_TE_137809	0.47727(21/44)	0.16667(3/18)	0.26316(5/19)	0.95000(19/20)
Bo_TE_223023	0.24390(10/41)	0.70588(12/17)	0.95000(19/20)	0.95000(19/20)
Bo_TE_6714	0.87179(34/39)	1.00000(18/18)	0.60000(12/20)	0.31579(6/19)
Bo_TE_49036	0.58974(23/39)	1.00000(17/17)	0.34783(8/23)	0.40000(8/20)
Bo_TE_170937	0.43182(19/44)	0.25000(4/16)	0.40000(8/20)	0.89474(17/19)
Bo_TE_102171	0.72500(29/40)	0.87500(14/16)	1.00000(22/22)	0.47368(9/19)
Bo_TE_100334	0.02439(1/41)	0.00000(0/19)	0.85000(17/20)	0.84211(16/19)
Bo_TE_169844	0.59524(25/42)	0.10526(2/19)	0.00000(0/22)	0.10526(2/19)
Bo_TE_126645	0.51220(21/41)	0.17647(3/17)	0.00000(0/22)	0.10000(2/20)
Bo_TE_186132	0.20513(8/39)	0.05263(1/19)	0.76190(16/21)	0.00000(0/18)
Bo_TE_89421	0.39535(17/43)	0.76471(13/17)	0.90476(19/21)	1.00000(18/18)
Bo_TE_119486	0.18605(8/43)	0.00000(0/18)	0.61905(13/21)	0.15789(3/19)
Bo_TE_178112	0.73810(31/42)	0.44444(8/18)	0.00000(0/20)	0.00000(0/18)
Bo_TE_95097	0.48718(19/39)	0.87500(14/16)	0.00000(0/22)	0.00000(0/20)
Bo_TE_109404	0.18919(7/37)	0.41176(7/17)	0.90000(18/20)	0.68421(13/19)
Bo_TE_144067	0.46512(20/43)	0.31250(5/16)	0.89474(17/19)	0.88235(15/17)
Bo_TE_74631	0.52500(21/40)	0.06250(1/16)	0.25000(1/4)	0.56250(9/16)
Bo_TE_194525	0.95652(44/46)	0.35294(6/17)	1.00000(23/23)	0.63158(12/19)
Bo_TE_30068	0.90000(36/40)	0.22222(4/18)	0.80000(16/20)	0.11765(2/17)
Bo_TE_97376	0.58537(24/41)	0.41176(7/17)	0.00000(0/21)	0.31579(6/19)
Bo_TE_13080	0.64103(25/39)	1.00000(16/16)	0.47619(10/21)	0.90000(18/20)
Bo_TE_90110	0.65909(29/44)	0.33333(6/18)	0.09091(2/22)	0.00000(0/20)
Bo_TE_178747	0.87805(36/41)	0.15385(2/13)	0.30000(6/20)	0.90000(18/20)
Bo_TE_32057	0.93333(42/45)	0.16667(3/18)	0.90909(20/22)	0.95000(19/20)
Bo_TE_231279	0.97674(42/43)	1.00000(16/16)	0.76190(16/21)	0.11111(2/18)
Bo_TE_18649	0.97727(43/44)	0.40000(6/15)	0.20000(4/20)	0.42105(8/19)
Bo_TE_25001	0.78049(32/41)	1.00000(3/3)	1.00000(22/22)	0.11111(2/18)
Bo_TE_231461	0.33333(14/42)	0.82353(14/17)	0.15000(3/20)	0.50000(7/14)
Bo_TE_8978	0.82927(34/41)	0.53333(8/15)	0.20000(4/20)	0.05000(1/20)
Bo_TE_94501	0.00000(0/44)	0.11765(2/17)	0.42857(9/21)	0.52632(10/19)
Bo_TE_39997	0.71795(28/39)	0.50000(6/12)	0.93333(14/15)	0.05556(1/18)
Bo_TE_227098	0.06977(3/43)	0.64286(9/14)	0.84211(16/19)	0.55556(10/18)
Bo_TE_174790	0.23077(9/39)	0.61111(11/18)	0.50000(11/22)	0.94737(18/19)
Bo_TE_119843	0.71053(27/38)	0.05882(1/17)	0.00000(0/23)	0.14286(2/14)
Bo_TE_121530	0.92857(39/42)	0.23529(4/17)	0.55000(11/20)	0.31579(6/19)
Bo_TE_60114	0.06667(3/45)	0.05556(1/18)	0.45455(10/22)	0.89474(17/19)
Bo_TE_98033	1.00000(46/46)	0.44444(8/18)	0.69565(16/23)	0.68421(13/19)
Bo_TE_4955	0.72093(31/43)	0.25000(4/16)	0.90000(18/20)	0.10526(2/19)

Bo_TE_89866	0.55814(24/43)	0.00000(0/19)	0.00000(0/23)	0.00000(0/20)
Bo_TE_236249	0.00000(0/42)	0.52632(10/19)	0.08696(2/23)	0.15000(3/20)
Bo_TE_143651	0.88636(39/44)	0.41176(7/17)	0.42857(9/21)	0.30000(6/20)
Bo_TE_56774	0.86047(37/43)	0.58824(10/17)	0.22727(5/22)	0.10526(2/19)
Bo_TE_53768	0.06818(3/44)	0.11111(2/18)	0.80000(16/20)	0.85000(17/20)
Bo_TE_5151	1.00000(45/45)	0.81250(13/16)	0.72727(16/22)	0.40000(8/20)
Bo_TE_200113	0.09524(4/42)	0.81250(13/16)	0.44444(8/18)	0.10526(2/19)
Bo_TE_137093	0.30233(13/43)	0.05556(1/18)	0.65000(13/20)	0.05263(1/19)
Bo_TE_44041	0.14286(6/42)	0.76471(13/17)	0.18182(4/22)	0.10526(2/19)
Bo_TE_226368	0.00000(0/45)	0.00000(0/19)	0.04348(1/23)	0.80000(16/20)
Bo_TE_155451	0.31707(13/41)	0.58824(10/17)	0.81818(18/22)	0.25000(5/20)
Bo_TE_178475	0.00000(0/45)	0.00000(0/19)	0.45000(9/20)	0.66667(12/18)
Bo_TE_132982	0.57143(24/42)	0.52941(9/17)	0.14286(3/21)	0.65000(13/20)
Bo_TE_124468	0.18182(8/44)	0.89474(17/19)	0.66667(14/21)	0.63158(12/19)
Bo_TE_92958	0.06667(3/45)	0.00000(0/18)	0.89474(17/19)	0.89474(17/19)
Bo_TE_186250	0.25581(11/43)	0.05556(1/18)	0.00000(0/22)	0.89474(17/19)
Bo_TE_172600	0.59091(26/44)	0.55556(10/18)	0.85000(17/20)	0.00000(0/18)
Bo_TE_169380	0.52381(22/42)	0.11765(2/17)	0.00000(0/22)	0.15789(3/19)
Bo_TE_79600	0.18605(8/43)	0.68750(11/16)	0.38095(8/21)	0.65000(13/20)
Bo_TE_89067	0.06977(3/43)	0.33333(5/15)	0.85714(18/21)	0.70588(12/17)
Bo_TE_69785	0.57778(26/45)	0.43750(7/16)	0.00000(0/23)	0.85000(17/20)
Bo_TE_209777	0.16279(7/43)	0.58824(10/17)	0.00000(0/17)	0.77778(14/18)
Bo_TE_129366	0.00000(0/43)	0.00000(0/19)	0.73684(14/19)	0.15000(3/20)
Bo_TE_44446	1.00000(43/43)	0.88889(16/18)	1.00000(21/21)	0.36842(7/19)
Bo_TE_25713	0.75556(34/45)	0.11765(2/17)	0.40000(8/20)	0.36842(7/19)
Bo_TE_86715	0.02381(1/42)	0.76471(13/17)	0.35000(7/20)	0.22222(4/18)
Bo_TE_42865	0.27027(10/37)	0.52941(9/17)	0.00000(0/22)	0.00000(0/19)
Bo_TE_49964	0.30233(13/43)	0.15789(3/19)	0.00000(0/22)	0.72222(13/18)
Bo_TE_15769	0.09756(4/41)	0.25000(4/16)	0.40000(8/20)	0.83333(15/18)
Bo_TE_107296	0.65854(27/41)	0.23529(4/17)	0.40909(9/22)	0.05000(1/20)
Bo_TE_124884	0.00000(0/43)	0.40000(6/15)	0.09091(2/22)	0.88889(16/18)
Bo_TE_214206	0.05000(2/40)	0.70588(12/17)	0.23810(5/21)	0.00000(0/20)
Bo_TE_227500	0.28571(12/42)	0.10526(2/19)	0.42857(9/21)	0.78947(15/19)
Bo_TE_218967	0.69231(27/39)	0.18750(3/16)	0.85714(18/21)	0.85000(17/20)
Bo_TE_18631	0.00000(0/45)	0.16667(3/18)	0.70000(14/20)	0.20000(4/20)
Bo_TE_65768	0.97826(45/46)	0.89474(17/19)	1.00000(23/23)	0.30000(6/20)
Bo_TE_238548	0.78049(32/41)	1.00000(9/9)	0.11765(2/17)	0.00000(0/18)
Bo_TE_130477	0.27500(11/40)	0.50000(9/18)	0.00000(0/22)	0.00000(0/20)
Bo_TE_198928	0.83333(35/42)	0.31250(5/16)	0.00000(0/22)	0.00000(0/19)
Bo_TE_66925	0.23077(9/39)	0.88235(15/17)	0.95652(22/23)	0.94444(17/18)
Bo_TE_33163	1.00000(44/44)	0.62500(10/16)	0.66667(14/21)	0.11111(2/18)
Bo_TE_63412	0.68293(28/41)	0.05556(1/18)	0.59091(13/22)	0.00000(0/19)
Bo_TE_96884	0.00000(0/46)	0.15789(3/19)	0.71429(15/21)	0.27778(5/18)
Bo_TE_77203	0.77778(35/45)	0.75000(12/16)	0.57143(12/21)	0.15000(3/20)
Bo_TE_104886	0.77778(35/45)	0.68750(11/16)	0.22727(5/22)	0.77778(14/18)
Bo_TE_146793	0.87500(35/40)	1.00000(19/19)	0.47619(10/21)	0.85000(17/20)
Bo_TE_53618	0.97826(45/46)	1.00000(17/17)	0.22727(5/22)	0.10526(2/19)
Bo_TE_38306	0.21429(9/42)	0.55556(10/18)	0.75000(15/20)	0.21053(4/19)
Bo_TE_136340	0.50000(21/42)	0.88889(16/18)	0.76190(16/21)	1.00000(20/20)
Bo_TE_223643	0.86364(38/44)	0.88889(16/18)	0.38095(8/21)	1.00000(19/19)
Bo_TE_61803	0.11628(5/43)	0.61111(11/18)	1.00000(23/23)	0.94444(17/18)
Bo_TE_91614	0.87500(35/40)	0.31579(6/19)	0.00000(0/22)	0.00000(0/20)
Bo_TE_49674	0.36585(15/41)	0.94118(16/17)	0.27273(6/22)	0.36842(7/19)
Bo_TE_80806	0.47059(16/34)	0.50000(9/18)	1.00000(22/22)	0.25000(4/16)
Bo_TE_97586	0.73171(30/41)	0.72222(13/18)	0.19048(4/21)	0.33333(6/18)
Bo_TE_56989	0.51163(22/43)	0.05882(1/17)	0.04545(1/22)	0.00000(0/20)

Bo_TE_239675	0.00000(0/39)	0.56250(9/16)	0.94444(17/18)	0.57895(11/19)
Bo_TE_204546	0.73810(31/42)	0.88889(16/18)	1.00000(20/20)	0.25000(5/20)
Bo_TE_169572	0.55814(24/43)	0.94737(18/19)	0.50000(10/20)	0.26316(5/19)
Bo_TE_124442	0.17778(8/45)	0.88235(15/17)	0.70000(14/20)	1.00000(19/19)
Bo_TE_139106	0.30000(12/40)	0.82353(14/17)	0.40000(8/20)	0.31579(6/19)
Bo_TE_11844	0.61538(24/39)	0.38889(7/18)	0.00000(0/23)	0.00000(0/20)
Bo_TE_83936	0.11628(5/43)	0.00000(0/18)	0.61905(13/21)	0.80000(16/20)
Bo_TE_92339	0.37500(15/40)	0.38889(7/18)	0.05000(1/20)	0.65000(13/20)
Bo_TE_222720	0.76744(33/43)	0.27778(5/18)	0.61905(13/21)	0.00000(0/19)
Bo_TE_38289	0.27273(12/44)	0.22222(4/18)	0.85714(18/21)	0.70000(14/20)
Bo_TE_236703	0.00000(0/43)	0.00000(0/18)	0.50000(11/22)	0.00000(0/19)
Bo_TE_144751	0.80952(34/42)	0.11111(2/18)	0.20000(4/20)	0.95000(19/20)
Bo_TE_97127	0.00000(0/42)	0.10526(2/19)	0.85714(18/21)	0.55556(10/18)
Bo_TE_135341	0.40476(17/42)	0.72222(13/18)	0.60000(12/20)	1.00000(19/19)
Bo_TE_128827	0.69048(29/42)	0.31579(6/19)	0.40909(9/22)	1.00000(20/20)
Bo_TE_45696	0.83333(35/42)	0.00000(0/18)	0.00000(0/22)	0.10000(2/20)
Bo_TE_212054	0.81395(35/43)	0.82353(14/17)	0.22727(5/22)	0.68421(13/19)
Bo_TE_159386	0.34884(15/43)	0.94118(16/17)	0.78261(18/23)	0.73684(14/19)
Bo_TE_86064	0.40000(18/45)	0.82353(14/17)	0.95455(21/22)	0.10526(2/19)
Bo_TE_168863	0.02273(1/44)	0.68750(11/16)	0.09524(2/21)	0.30769(4/13)
Bo_TE_128869	0.36364(16/44)	0.23529(4/17)	0.47826(11/23)	1.00000(20/20)
Bo_TE_178103	0.02273(1/44)	0.00000(0/19)	0.00000(0/22)	0.70000(14/20)
Bo_TE_136242	0.75000(30/40)	0.72222(13/18)	0.76190(16/21)	0.20000(4/20)
Bo_TE_53138	0.53659(22/41)	0.29412(5/17)	0.47826(11/23)	1.00000(20/20)
Bo_TE_142162	0.02703(1/37)	0.00000(0/17)	0.50000(11/22)	0.43750(7/16)
Bo_TE_25415	0.02381(1/42)	0.16667(3/18)	0.33333(7/21)	0.80000(16/20)
Bo_TE_184855	0.34091(15/44)	0.47059(8/17)	0.36364(8/22)	1.00000(18/18)
Bo_TE_111604	0.82222(37/45)	0.77778(14/18)	0.25000(5/20)	0.88889(16/18)
Bo_TE_19725	0.57500(23/40)	0.05882(1/17)	0.20000(4/20)	0.35294(6/17)
Bo_TE_17999	0.00000(0/45)	0.00000(0/18)	0.50000(10/20)	0.05000(1/20)
Bo_TE_5223	0.16279(7/43)	0.63158(12/19)	0.71429(15/21)	0.94737(18/19)
Bo_TE_140919	0.24390(10/41)	0.88235(15/17)	0.90476(19/21)	0.84211(16/19)
Bo_TE_4302	0.90476(38/42)	0.73333(11/15)	0.95238(20/21)	0.15000(3/20)
Bo_TE_165553	0.13953(6/43)	0.70588(12/17)	0.45000(9/20)	0.80000(16/20)
Bo_TE_118537	0.57500(23/40)	0.10526(2/19)	0.00000(0/21)	0.00000(0/20)
Bo_TE_92830	0.02222(1/45)	0.00000(0/17)	0.14286(3/21)	0.65000(13/20)
Bo_TE_33445	0.89130(41/46)	0.16667(3/18)	0.19048(4/21)	0.05263(1/19)
Bo_TE_200214	0.82927(34/41)	0.35294(6/17)	0.39130(9/23)	0.20000(4/20)
Bo_TE_131726	0.52381(22/42)	0.76471(13/17)	0.04762(1/21)	0.38889(7/18)
Bo_TE_225058	0.13636(6/44)	0.37500(6/16)	0.84211(16/19)	0.00000(0/20)
Bo_TE_140329	0.65000(26/40)	0.77778(14/18)	0.36364(8/22)	1.00000(18/18)
Bo_TE_203670	0.65000(26/40)	0.31250(5/16)	0.80000(16/20)	0.94737(18/19)
Bo_TE_149418	0.04444(2/45)	0.00000(0/17)	0.80000(16/20)	0.00000(0/20)
Bo_TE_48221	0.52381(22/42)	0.35294(6/17)	0.00000(0/22)	0.00000(0/19)
Bo_TE_191037	0.70000(28/40)	0.05556(1/18)	0.00000(0/23)	0.10000(2/20)
Bo_TE_15458	0.74419(32/43)	0.05882(1/17)	0.00000(0/21)	0.55556(10/18)
Bo_TE_125315	0.00000(0/45)	0.50000(8/16)	0.45000(9/20)	0.05263(1/19)
Bo_TE_206485	0.00000(0/43)	0.70588(12/17)	0.36842(7/19)	0.00000(0/20)
Bo_TE_226714	0.66667(26/39)	0.82353(14/17)	0.33333(7/21)	0.90000(18/20)
Bo_TE_223668	0.88889(40/45)	0.93750(15/16)	0.40909(9/22)	1.00000(20/20)
Bo_TE_211832	0.11364(5/44)	0.44444(8/18)	0.90000(18/20)	0.31579(6/19)
Bo_TE_36433	0.11364(5/44)	0.77778(14/18)	0.65217(15/23)	0.61111(11/18)
Bo_TE_151482	0.33333(14/42)	0.66667(12/18)	0.95238(20/21)	0.90000(18/20)
Bo_TE_195925	0.62791(27/43)	0.10526(2/19)	0.10000(2/20)	0.00000(0/20)
Bo_TE_63010	0.54762(23/42)	0.33333(6/18)	0.00000(0/22)	0.00000(0/20)
Bo_TE_75725	0.02273(1/44)	0.64706(11/17)	0.20000(4/20)	0.00000(0/19)

Bo_TE_195046	0.37500(15/40)	0.00000(0/18)	0.68182(15/22)	0.16667(3/18)
Bo_TE_48973	0.65854(27/41)	0.87500(14/16)	0.09524(2/21)	0.33333(6/18)
Bo_TE_141588	0.20000(9/45)	0.88235(15/17)	0.95455(21/22)	0.83333(15/18)
Bo_TE_173275	0.19512(8/41)	0.00000(0/19)	0.85000(17/20)	0.00000(0/19)
Bo_TE_77406	0.04762(2/42)	0.46667(7/15)	0.42857(9/21)	0.84211(16/19)
Bo_TE_155063	0.15556(7/45)	0.00000(0/18)	0.27273(6/22)	0.70000(14/20)
Bo_TE_113747	0.23810(10/42)	0.30769(4/13)	0.27273(6/22)	0.80000(16/20)
Bo_TE_145239	0.50000(21/42)	0.05556(1/18)	0.00000(0/22)	0.05263(1/19)
Bo_TE_34896	0.72727(32/44)	1.00000(17/17)	0.23810(5/21)	0.55000(11/20)
Bo_TE_150588	0.53659(22/41)	0.17647(3/17)	0.00000(0/21)	0.15000(3/20)
Bo_TE_54934	0.04545(2/44)	0.64706(11/17)	0.66667(14/21)	0.05263(1/19)
Bo_TE_159836	0.56098(23/41)	0.05556(1/18)	0.70000(14/20)	0.00000(0/20)
Bo_TE_112256	0.00000(0/31)	0.61111(11/18)	0.00000(0/21)	0.10000(2/20)
Bo_TE_116806	0.00000(0/45)	0.00000(0/19)	0.55000(11/20)	0.10000(2/20)
Bo_TE_167873	0.62222(28/45)	0.72222(13/18)	0.00000(0/23)	0.15000(3/20)
Bo_TE_121874	0.51163(22/43)	0.00000(0/16)	0.00000(0/23)	0.05000(1/20)
Bo_TE_71298	0.88889(40/45)	0.05882(1/17)	0.08696(2/23)	0.15000(3/20)
Bo_TE_85793	0.00000(0/45)	0.33333(6/18)	0.09091(2/22)	0.50000(10/20)
Bo_TE_10252	0.67568(25/37)	0.53846(7/13)	0.04545(1/22)	0.21053(4/19)
Bo_TE_143448	0.68293(28/41)	0.00000(0/18)	0.19048(4/21)	0.21053(4/19)
Bo_TE_74303	0.00000(0/43)	0.00000(0/19)	0.22727(5/22)	0.72222(13/18)
Bo_TE_128777	0.28947(11/38)	0.72222(13/18)	0.55000(11/20)	0.00000(0/20)
Bo_TE_140165	0.92683(38/41)	0.64706(11/17)	0.42857(9/21)	0.31579(6/19)
Bo_TE_183956	0.81395(35/43)	0.72222(13/18)	0.04545(1/22)	0.05263(1/19)
Bo_TE_235773	1.00000(44/44)	1.00000(18/18)	0.68182(15/22)	0.26316(5/19)
Bo_TE_7484	0.00000(0/43)	0.00000(0/18)	0.18182(4/22)	0.57895(11/19)
Bo_TE_28605	0.07143(3/42)	0.11765(2/17)	0.47059(8/17)	0.84211(16/19)
Bo_TE_103842	0.88636(39/44)	0.64706(11/17)	0.14286(3/21)	0.10526(2/19)
Bo_TE_212512	0.30000(12/40)	0.73684(14/19)	0.81818(18/22)	0.10000(2/20)
Bo_TE_110896	0.88889(40/45)	0.93750(15/16)	0.94737(18/19)	0.31579(6/19)
Bo_TE_1186	0.54762(23/42)	0.31250(5/16)	1.00000(20/20)	0.73684(14/19)
Bo_TE_130542	0.74419(32/43)	0.12500(2/16)	0.61905(13/21)	0.10526(2/19)
Bo_TE_89151	0.97826(45/46)	0.62500(10/16)	0.14286(3/21)	0.38462(5/13)
Bo_TE_31517	0.10000(4/40)	0.11111(2/18)	0.00000(0/22)	0.63158(12/19)
Bo_TE_123993	0.79545(35/44)	0.94737(18/19)	0.95455(21/22)	0.16667(3/18)
Bo_TE_173783	0.08696(4/46)	0.00000(0/17)	0.58824(10/17)	0.88235(15/17)
Bo_TE_136044	0.52500(21/40)	0.61111(11/18)	0.00000(0/23)	0.00000(0/20)
Bo_TE_154492	0.97778(44/45)	0.13333(2/15)	0.22727(5/22)	0.12500(2/16)
Bo_TE_171220	0.00000(0/43)	0.50000(9/18)	0.00000(0/23)	0.00000(0/20)
Bo_TE_114215	0.02174(1/46)	0.00000(0/19)	0.04545(1/22)	0.77778(14/18)
Bo_TE_117464	0.04545(2/44)	0.81250(13/16)	0.69565(16/23)	0.42105(8/19)
Bo_TE_223005	0.71429(30/42)	0.93333(14/15)	0.85000(17/20)	0.00000(0/19)
Bo_TE_117499	0.04545(2/44)	0.77778(14/18)	0.44444(8/18)	0.42105(8/19)
Bo_TE_154644	0.23256(10/43)	0.28571(4/14)	0.80952(17/21)	0.31579(6/19)
Bo_TE_145911	0.22500(9/40)	0.94118(16/17)	1.00000(21/21)	1.00000(19/19)
Bo_TE_117823	0.78049(32/41)	0.17647(3/17)	0.60000(12/20)	0.85000(17/20)
Bo_TE_136887	0.00000(0/43)	0.64706(11/17)	0.00000(0/21)	0.00000(0/17)
Bo_TE_133086	0.04651(2/43)	0.05263(1/19)	0.81818(18/22)	0.10000(2/20)
Bo_TE_179330	0.54545(24/44)	0.35294(6/17)	0.00000(0/21)	0.10526(2/19)
Bo_TE_102629	0.19048(8/42)	0.05882(1/17)	0.59091(13/22)	0.12500(2/16)
Bo_TE_215390	0.00000(0/45)	0.00000(0/19)	0.31818(7/22)	0.76471(13/17)
Bo_TE_239523	0.21053(8/38)	0.68750(11/16)	0.04348(1/23)	0.31579(6/19)
Bo_TE_142293	0.26829(11/41)	0.77778(14/18)	0.78261(18/23)	0.94737(18/19)
Bo_TE_86793	0.65000(26/40)	0.11765(2/17)	0.18182(4/22)	0.55000(11/20)
Bo_TE_195391	0.18182(8/44)	0.73333(11/15)	0.00000(0/21)	0.52632(10/19)
Bo_TE_28034	0.19512(8/41)	0.88235(15/17)	0.52381(11/21)	0.90000(18/20)

Bo_TE_6644	0.76190(32/42)	0.06250(1/16)	0.63158(12/19)	0.73684(14/19)
Bo_TE_60154	0.59524(25/42)	0.68750(11/16)	0.00000(0/22)	0.00000(0/18)
Bo_TE_73083	0.10000(4/40)	0.61111(11/18)	0.31579(6/19)	0.60000(12/20)
Bo_TE_220816	1.00000(46/46)	0.11765(2/17)	0.71429(15/21)	0.38889(7/18)
Bo_TE_93342	0.63636(28/44)	0.30000(3/10)	0.00000(0/23)	0.44444(8/18)
Bo_TE_117383	0.04348(2/46)	0.83333(15/18)	0.66667(14/21)	0.50000(9/18)
Bo_TE_236946	0.48780(20/41)	0.93750(15/16)	1.00000(23/23)	0.80000(16/20)
Bo_TE_49045	0.02326(1/43)	0.76471(13/17)	0.54545(12/22)	0.23529(4/17)
Bo_TE_150744	0.85714(36/42)	0.31250(5/16)	0.27273(6/22)	0.15000(3/20)
Bo_TE_179561	0.00000(0/43)	0.00000(0/18)	0.80000(16/20)	0.00000(0/20)
Bo_TE_207898	0.50000(22/44)	0.18750(3/16)	0.82609(19/23)	0.63158(12/19)
Bo_TE_67930	0.87179(34/39)	0.05882(1/17)	0.14286(3/21)	0.00000(0/18)
Bo_TE_27370	0.77273(34/44)	0.88235(15/17)	0.45455(10/22)	0.05556(1/18)
Bo_TE_14098	0.54762(23/42)	0.81250(13/16)	0.75000(15/20)	0.15789(3/19)
Bo_TE_178279	0.11628(5/43)	0.83333(15/18)	0.85714(18/21)	0.88889(16/18)
Bo_TE_216233	0.00000(0/43)	0.12500(2/16)	0.57143(12/21)	0.20000(4/20)
Bo_TE_63706	0.39535(17/43)	0.33333(6/18)	0.80952(17/21)	0.11111(2/18)
Bo_TE_194849	0.55000(22/40)	0.23529(4/17)	0.23810(5/21)	0.05000(1/20)
Bo_TE_17967	0.17073(7/41)	0.58824(10/17)	0.70000(14/20)	0.10000(2/20)
Bo_TE_126173	0.37209(16/43)	0.81250(13/16)	0.86364(19/22)	0.10000(2/20)
Bo_TE_42731	0.62500(25/40)	0.62500(10/16)	0.00000(0/22)	0.55000(11/20)
Bo_TE_69857	0.48780(20/41)	0.43750(7/16)	0.35000(7/20)	0.90000(18/20)
Bo_TE_79127	0.41860(18/43)	0.23529(4/17)	0.71429(15/21)	0.80000(16/20)
Bo_TE_214886	0.00000(0/42)	0.05556(1/18)	0.85000(17/20)	0.83333(15/18)
Bo_TE_9575	0.27273(12/44)	0.81250(13/16)	0.57143(12/21)	0.30000(6/20)
Bo_TE_15572	0.00000(0/44)	0.00000(0/18)	0.60000(12/20)	0.00000(0/16)
Bo_TE_199043	0.16667(7/42)	0.75000(12/16)	0.95455(21/22)	0.89474(17/19)
Bo_TE_26361	0.83333(35/42)	0.17647(3/17)	0.47619(10/21)	0.90000(18/20)
Bo_TE_117734	0.64286(27/42)	0.35294(6/17)	0.31818(7/22)	0.05000(1/20)
Bo_TE_183438	0.45000(18/40)	0.05556(1/18)	0.18182(4/22)	0.73684(14/19)
Bo_TE_46477	0.04444(2/45)	0.52941(9/17)	0.57895(11/19)	0.45000(9/20)
Bo_TE_111590	0.84091(37/44)	0.72222(13/18)	0.22727(5/22)	0.85000(17/20)
Bo_TE_99307	0.80952(34/42)	0.07692(1/13)	0.25000(5/20)	0.57895(11/19)
Bo_TE_230981	0.15556(7/45)	0.11111(2/18)	1.00000(22/22)	0.95000(19/20)
Bo_TE_200231	0.00000(0/44)	0.00000(0/18)	0.22727(5/22)	0.68421(13/19)
Bo_TE_96004	0.19512(8/41)	0.29412(5/17)	0.63158(12/19)	0.95000(19/20)
Bo_TE_50578	0.65909(29/44)	0.94444(17/18)	0.91304(21/23)	0.15000(3/20)
Bo_TE_126414	0.84091(37/44)	0.55556(10/18)	0.52381(11/21)	0.17647(3/17)
Bo_TE_48213	0.11628(5/43)	0.58824(10/17)	0.04762(1/21)	0.44444(8/18)
Bo_TE_145118	0.65116(28/43)	0.73333(11/15)	0.90909(20/22)	0.26316(5/19)
Bo_TE_78700	0.67442(29/43)	0.18750(3/16)	0.00000(0/23)	0.00000(0/20)
Bo_TE_130319	0.18605(8/43)	0.00000(0/17)	0.80952(17/21)	0.76471(13/17)
Bo_TE_127386	0.17500(7/40)	0.72222(13/18)	1.00000(23/23)	0.94737(18/19)
Bo_TE_108570	0.26190(11/42)	0.16667(3/18)	0.65217(15/23)	0.95000(19/20)
Bo_TE_135368	0.54762(23/42)	0.16667(3/18)	0.00000(0/23)	0.00000(0/20)
Bo_TE_221293	1.00000(41/41)	0.23529(4/17)	0.95652(22/23)	0.94444(17/18)
Bo_TE_2804	0.78049(32/41)	0.56250(9/16)	0.00000(0/23)	0.00000(0/20)
Bo_TE_174044	0.71429(30/42)	0.76471(13/17)	0.47619(10/21)	0.21053(4/19)
Bo_TE_181632	0.46512(20/43)	0.61111(11/18)	0.04545(1/22)	0.66667(12/18)
Bo_TE_141551	0.15556(7/45)	0.92857(13/14)	0.95000(19/20)	0.78947(15/19)
Bo_TE_223900	0.11364(5/44)	0.58824(10/17)	0.21053(4/19)	0.00000(0/20)
Bo_TE_174319	1.00000(44/44)	1.00000(17/17)	0.86364(19/22)	0.22222(4/18)
Bo_TE_232084	0.59524(25/42)	0.47059(8/17)	0.04545(1/22)	0.31579(6/19)
Bo_TE_28584	0.97778(44/45)	1.00000(19/19)	0.22727(5/22)	0.50000(9/18)
Bo_TE_21583	0.06977(3/43)	0.36842(7/19)	0.50000(10/20)	0.00000(0/19)
Bo_TE_76407	0.00000(0/45)	0.06667(1/15)	0.13636(3/22)	0.52632(10/19)

Bo_TE_179951	0.00000(0/46)	0.05556(1/18)	0.00000(0/21)	0.68421(13/19)
Bo_TE_133520	0.43590(17/39)	0.68750(11/16)	0.57143(12/21)	1.00000(20/20)
Bo_TE_132233	0.28205(11/39)	0.62500(10/16)	0.95455(21/22)	1.00000(20/20)
Bo_TE_38119	0.83333(35/42)	0.50000(8/16)	0.60000(12/20)	0.26316(5/19)
Bo_TE_6155	0.97619(41/42)	0.88235(15/17)	0.31579(6/19)	0.33333(5/15)
Bo_TE_133926	0.55814(24/43)	0.00000(0/15)	0.28571(6/21)	0.05000(1/20)
Bo_TE_125296	0.79545(35/44)	0.27778(5/18)	0.31579(6/19)	0.57895(11/19)
Bo_TE_61418	0.53488(23/43)	0.11765(2/17)	0.91304(21/23)	0.85000(17/20)
Bo_TE_235067	0.30952(13/42)	0.64706(11/17)	0.60000(12/20)	0.84211(16/19)
Bo_TE_103947	1.00000(44/44)	0.89474(17/19)	0.45455(10/22)	0.50000(8/16)
Bo_TE_125104	0.35714(15/42)	0.06250(1/16)	0.42857(9/21)	0.84211(16/19)
Bo_TE_122901	0.95349(41/43)	0.35294(6/17)	1.00000(21/21)	0.70000(14/20)
Bo_TE_46753	0.79070(34/43)	0.70588(12/17)	0.15789(3/19)	0.20000(4/20)
Bo_TE_73014	0.91892(34/37)	0.18750(3/16)	0.52381(11/21)	0.35294(6/17)
Bo_TE_219249	0.38095(16/42)	1.00000(17/17)	0.71429(15/21)	0.80000(16/20)
Bo_TE_101966	0.13636(6/44)	0.61111(11/18)	0.95238(20/21)	0.45000(9/20)
Bo_TE_47892	0.50000(21/42)	0.88235(15/17)	0.00000(0/22)	0.20000(4/20)
Bo_TE_195036	0.37500(15/40)	0.94118(16/17)	0.33333(7/21)	0.95000(19/20)
Bo_TE_179929	0.02381(1/42)	0.05556(1/18)	0.75000(15/20)	0.55000(11/20)
Bo_TE_181759	0.63415(26/41)	0.11111(2/18)	0.60000(12/20)	0.26316(5/19)
Bo_TE_100023	0.24444(11/45)	0.00000(0/17)	0.65000(13/20)	0.85000(17/20)
Bo_TE_217969	0.40909(18/44)	0.94118(16/17)	0.30435(7/23)	0.15000(3/20)
Bo_TE_53551	0.08889(4/45)	0.00000(0/18)	0.09091(2/22)	0.89474(17/19)
Bo_TE_174576	0.35000(14/40)	0.76471(13/17)	1.00000(22/22)	1.00000(20/20)
Bo_TE_204935	0.22500(9/40)	0.82353(14/17)	0.77273(17/22)	1.00000(20/20)
Bo_TE_177942	0.04545(2/44)	0.06250(1/16)	0.85000(17/20)	0.55556(10/18)
Bo_TE_185121	0.22222(10/45)	0.25000(4/16)	0.63636(14/22)	0.93750(15/16)
Bo_TE_15492	0.73171(30/41)	0.11111(2/18)	0.00000(0/22)	0.00000(0/19)
Bo_TE_149654	0.76190(32/42)	0.94444(17/18)	0.42857(9/21)	0.50000(10/20)
Bo_TE_158335	0.09091(4/44)	0.17647(3/17)	0.85000(17/20)	0.77778(14/18)
Bo_TE_214154	0.00000(0/45)	0.56250(9/16)	0.22727(5/22)	0.00000(0/20)
Bo_TE_121560	0.00000(0/42)	0.00000(0/18)	0.40909(9/22)	0.70588(12/17)
Bo_TE_149630	0.02273(1/44)	0.05882(1/17)	0.55000(11/20)	0.42105(8/19)
Bo_TE_136578	0.35000(14/40)	0.16667(3/18)	0.73684(14/19)	0.20000(4/20)
Bo_TE_67646	0.15556(7/45)	0.06250(1/16)	0.76190(16/21)	0.33333(6/18)
Bo_TE_30422	0.00000(0/42)	0.05882(1/17)	0.73684(14/19)	0.55556(10/18)
Bo_TE_44639	0.79070(34/43)	0.27778(5/18)	0.09091(2/22)	0.75000(15/20)
Bo_TE_234880	0.02381(1/42)	0.05556(1/18)	0.00000(0/22)	0.50000(9/18)
Bo_TE_213785	0.61364(27/44)	0.23529(4/17)	0.50000(10/20)	0.90000(18/20)
Bo_TE_236664	0.00000(0/46)	0.25000(4/16)	0.76190(16/21)	0.00000(0/19)
Bo_TE_180993	0.28205(11/39)	0.16667(3/18)	0.80000(16/20)	0.15789(3/19)
Bo_TE_223939	0.09302(4/43)	0.68750(11/16)	0.33333(7/21)	0.00000(0/20)
Bo_TE_151411	0.00000(0/46)	0.00000(0/19)	0.00000(0/23)	0.55556(10/18)
Bo_TE_29010	0.40476(17/42)	0.41176(7/17)	0.85714(18/21)	0.15000(3/20)
Bo_TE_111084	0.34091(15/44)	0.70588(12/17)	0.00000(0/23)	0.20000(4/20)
Bo_TE_95751	0.02174(1/46)	0.00000(0/19)	0.00000(0/21)	0.80000(16/20)
Bo_TE_138451	0.00000(0/45)	0.05263(1/19)	0.18182(4/22)	0.75000(15/20)
Bo_TE_43759	0.80952(34/42)	0.05556(1/18)	0.45455(10/22)	0.68421(13/19)
Bo_TE_5247	0.76744(33/43)	0.18750(3/16)	0.00000(0/23)	0.00000(0/20)
Bo_TE_11807	0.60976(25/41)	0.41176(7/17)	0.00000(0/23)	0.00000(0/20)
Bo_TE_154645	0.27273(12/44)	0.28571(4/14)	0.77273(17/22)	0.29412(5/17)
Bo_TE_138764	0.62500(25/40)	0.11765(2/17)	0.33333(7/21)	0.77778(14/18)
Bo_TE_149056	0.77500(31/40)	0.50000(8/16)	0.00000(0/22)	0.00000(0/20)
Bo_TE_152271	0.81395(35/43)	0.10526(2/19)	0.43478(10/23)	0.10000(2/20)
Bo_TE_196560	0.58537(24/41)	0.00000(0/17)	0.00000(0/23)	0.00000(0/20)
Bo_TE_15469	0.33333(15/45)	0.05556(1/18)	0.00000(0/21)	0.52632(10/19)

Bo_TE_209875	0.97727(43/44)	0.38889(7/18)	0.90000(18/20)	1.00000(20/20)
Bo_TE_28138	0.18182(8/44)	0.88889(16/18)	0.18182(4/22)	0.36842(7/19)
Bo_TE_50459	0.11364(5/44)	0.76471(13/17)	0.39130(9/23)	0.83333(15/18)
Bo_TE_92896	0.22222(10/45)	0.66667(12/18)	0.04348(1/23)	0.15789(3/19)
Bo_TE_15430	0.26667(12/45)	0.75000(12/16)	1.00000(23/23)	0.42105(8/19)
Bo_TE_110116	0.37209(16/43)	0.31250(5/16)	1.00000(23/23)	1.00000(20/20)
Bo_TE_143782	0.17778(8/45)	0.05263(1/19)	0.84211(16/19)	0.22222(4/18)
Bo_TE_9255	0.80000(36/45)	0.35294(6/17)	0.80000(16/20)	1.00000(19/19)
Bo_TE_30287	0.71795(28/39)	0.12500(2/16)	0.75000(15/20)	0.94737(18/19)
Bo_TE_116219	0.97727(43/44)	0.58824(10/17)	0.17391(4/23)	0.42105(8/19)
Bo_TE_13424	0.78571(33/42)	0.23529(4/17)	0.09524(2/21)	0.00000(0/20)
Bo_TE_121726	0.52632(20/38)	0.00000(0/18)	0.80000(16/20)	0.50000(1/2)
Bo_TE_53263	0.02381(1/42)	0.16667(3/18)	0.73913(17/23)	0.00000(0/19)
Bo_TE_220379	0.75610(31/41)	0.11765(2/17)	0.40000(8/20)	0.85000(17/20)
Bo_TE_158103	0.19048(8/42)	0.42857(6/14)	0.57143(12/21)	0.72222(13/18)
Bo_TE_24471	0.59524(25/42)	0.84211(16/19)	0.00000(0/23)	0.11111(2/18)
Bo_TE_14617	0.25581(11/43)	0.37500(6/16)	0.78947(15/19)	0.94737(18/19)
Bo_TE_238594	0.88636(39/44)	0.70588(12/17)	0.42857(9/21)	0.10526(2/19)
Bo_TE_189805	0.58140(25/43)	0.47059(8/17)	0.00000(0/23)	0.00000(0/20)
Bo_TE_111938	0.37778(17/45)	0.68750(11/16)	0.28571(6/21)	0.88889(16/18)
Bo_TE_62734	0.41176(14/34)	0.88889(16/18)	0.00000(0/20)	0.05556(1/18)
Bo_TE_191036	0.27907(12/43)	0.38889(7/18)	0.95652(22/23)	0.78947(15/19)
Bo_TE_174537	0.48780(20/41)	0.41176(7/17)	0.95455(21/22)	0.27778(5/18)
Bo_TE_174581	0.32500(13/40)	0.88235(15/17)	1.00000(20/20)	1.00000(20/20)
Bo_TE_25902	0.79070(34/43)	0.11765(2/17)	0.47619(10/21)	0.10000(2/20)
Bo_TE_141469	0.80000(36/45)	0.05882(1/17)	0.04545(1/22)	0.21053(4/19)
Bo_TE_180997	0.65116(28/43)	0.77778(14/18)	0.23810(5/21)	0.78947(15/19)
Bo_TE_224721	0.00000(0/46)	0.00000(0/18)	0.86364(19/22)	0.63158(12/19)
Bo_TE_56586	0.46667(21/45)	0.52941(9/17)	0.75000(15/20)	0.05556(1/18)
Bo_TE_141496	0.81395(35/43)	0.05882(1/17)	0.04762(1/21)	0.21053(4/19)
Bo_TE_122724	0.48780(20/41)	0.73684(14/19)	1.00000(22/22)	1.00000(20/20)
Bo_TE_182231	0.77778(35/45)	0.23529(4/17)	1.00000(21/21)	0.70000(14/20)
Bo_TE_178752	0.00000(0/46)	0.52941(9/17)	0.00000(0/23)	0.00000(0/20)
Bo_TE_62756	0.02273(1/44)	0.66667(12/18)	0.42857(9/21)	0.00000(0/20)
Bo_TE_137670	0.04348(1/23)	0.11111(2/18)	0.59091(13/22)	0.00000(0/18)
Bo_TE_220591	0.71795(28/39)	0.81250(13/16)	0.63158(12/19)	0.11111(2/18)
Bo_TE_78817	0.54762(23/42)	0.68750(11/16)	0.47368(9/19)	0.16667(3/18)
Bo_TE_79782	0.00000(0/37)	0.23077(3/13)	0.65000(13/20)	0.00000(0/16)
Bo_TE_220909	0.69767(30/43)	0.00000(0/18)	0.00000(0/23)	0.11111(2/18)
Bo_TE_225360	0.26829(11/41)	0.00000(0/17)	0.54545(12/22)	0.00000(0/20)
Bo_TE_92187	0.86364(38/44)	0.66667(10/15)	0.95238(20/21)	0.33333(6/18)
Bo_TE_13368	0.97727(43/44)	0.83333(15/18)	0.42857(9/21)	1.00000(20/20)
Bo_TE_57200	0.00000(0/46)	0.00000(0/19)	0.22727(5/22)	0.61111(11/18)
Bo_TE_235769	0.00000(0/43)	0.07692(1/13)	0.31818(7/22)	0.78947(15/19)
Bo_TE_184270	0.13043(6/46)	0.12500(2/16)	0.61905(13/21)	0.10000(2/20)
Bo_TE_177246	0.00000(0/44)	0.57895(11/19)	0.00000(0/23)	0.00000(0/20)
Bo_TE_211931	0.00000(0/44)	0.00000(0/19)	0.71429(15/21)	0.00000(0/20)
Bo_TE_142086	0.30000(12/40)	0.57895(11/19)	0.00000(0/19)	0.00000(0/16)
Bo_TE_153388	0.46154(18/39)	0.31579(6/19)	0.71429(15/21)	0.83333(15/18)
Bo_TE_27448	0.00000(0/43)	0.05882(1/17)	0.54545(12/22)	0.20000(4/20)
Bo_TE_156988	0.52381(22/42)	0.11765(2/17)	0.95652(22/23)	0.31579(6/19)
Bo_TE_195966	0.58974(23/39)	0.81250(13/16)	0.27273(6/22)	0.35294(6/17)
Bo_TE_37400	0.09302(4/43)	0.15789(3/19)	0.81818(18/22)	0.89474(17/19)
Bo_TE_173508	0.86486(32/37)	0.88235(15/17)	0.00000(0/22)	0.00000(0/20)
Bo_TE_101030	0.18605(8/43)	0.35294(6/17)	0.61905(13/21)	0.78947(15/19)
Bo_TE_12405	0.97778(44/45)	0.37500(6/16)	0.19048(4/21)	0.85000(17/20)

Bo_TE_140346	0.68293(28/41)	0.76471(13/17)	0.38095(8/21)	1.00000(19/19)
Bo_TE_96934	0.00000(0/45)	0.50000(9/18)	0.13636(3/22)	0.00000(0/20)
Bo_TE_84535	0.67500(27/40)	0.00000(0/16)	0.45455(10/22)	0.70588(12/17)
Bo_TE_94665	0.79070(34/43)	0.50000(9/18)	0.00000(0/21)	0.65000(13/20)
Bo_TE_143678	0.02222(1/45)	0.52941(9/17)	0.00000(0/22)	0.45000(9/20)
Bo_TE_215462	0.13636(6/44)	0.00000(0/19)	0.19048(4/21)	0.52632(10/19)
Bo_TE_15790	0.09524(4/42)	0.23529(4/17)	0.38889(7/18)	0.85000(17/20)
Bo_TE_55824	0.93333(42/45)	0.70588(12/17)	0.18182(4/22)	0.85000(17/20)
Bo_TE_141524	0.81395(35/43)	0.05882(1/17)	0.04762(1/21)	0.21053(4/19)
Bo_TE_73167	0.54054(20/37)	0.11111(2/18)	0.39130(9/23)	0.70000(14/20)
Bo_TE_199168	0.00000(0/46)	0.00000(0/18)	0.54545(12/22)	0.00000(0/20)
Bo_TE_45332	0.55814(24/43)	0.44444(8/18)	0.85714(18/21)	0.00000(0/18)
Bo_TE_149633	0.25581(11/43)	0.05882(1/17)	0.57143(12/21)	0.50000(10/20)
Bo_TE_95748	0.60465(26/43)	0.66667(12/18)	0.18182(4/22)	0.00000(0/20)
Bo_TE_199555	0.04651(2/43)	0.00000(0/17)	0.80000(16/20)	0.00000(0/18)
Bo_TE_234709	0.52273(23/44)	0.31250(5/16)	0.00000(0/23)	0.55556(10/18)
Bo_TE_224484	0.00000(0/42)	0.22222(4/18)	0.15000(3/20)	0.75000(15/20)
Bo_TE_95853	0.56098(23/41)	0.68750(11/16)	0.05000(1/20)	0.84211(16/19)
Bo_TE_172486	0.97727(43/44)	1.00000(19/19)	0.95652(22/23)	0.47368(9/19)
Bo_TE_21461	0.57895(22/38)	0.22222(4/18)	0.04545(1/22)	0.05000(1/20)
Bo_TE_91540	0.61905(26/42)	0.06250(1/16)	0.04348(1/23)	0.16667(3/18)
Bo_TE_237285	0.46341(19/41)	0.93750(15/16)	0.40000(8/20)	0.61111(11/18)
Bo_TE_34096	0.68293(28/41)	0.17647(3/17)	0.72727(16/22)	0.15789(3/19)
Bo_TE_34727	0.55000(22/40)	1.00000(18/18)	0.13636(3/22)	0.56250(9/16)
Bo_TE_39573	0.73171(30/41)	0.82353(14/17)	0.26087(6/23)	0.44444(8/18)
Bo_TE_141187	0.09302(4/43)	0.88889(16/18)	0.17391(4/23)	0.82353(14/17)
Bo_TE_114224	0.00000(0/46)	0.05556(1/18)	0.80952(17/21)	0.00000(0/20)
Bo_TE_106120	0.11905(5/42)	0.76471(13/17)	0.00000(0/21)	0.00000(0/17)
Bo_TE_156741	0.44186(19/43)	0.88889(16/18)	0.18182(4/22)	0.44444(8/18)
Bo_TE_146072	0.95455(42/44)	0.94444(17/18)	0.45000(9/20)	0.65000(13/20)
Bo_TE_63987	0.48718(19/39)	0.93750(15/16)	1.00000(23/23)	0.90000(18/20)
Bo_TE_227697	0.85714(36/42)	0.94118(16/17)	0.14286(3/21)	0.15789(3/19)
Bo_TE_101732	0.26829(11/41)	0.58824(10/17)	0.45455(10/22)	1.00000(19/19)
Bo_TE_168449	0.00000(0/46)	0.00000(0/18)	0.65000(13/20)	0.85000(17/20)
Bo_TE_104853	0.15789(6/38)	0.70588(12/17)	0.77273(17/22)	0.47368(9/19)
Bo_TE_136010	0.23810(10/42)	0.15789(3/19)	0.95238(20/21)	1.00000(2/2)
Bo_TE_45162	0.48889(22/45)	0.26667(4/15)	0.63636(14/22)	0.00000(0/20)
Bo_TE_138494	0.26316(10/38)	0.17647(3/17)	0.90000(18/20)	0.68421(13/19)
Bo_TE_10229	0.58140(25/43)	0.44444(8/18)	0.30000(6/20)	0.00000(0/20)
Bo_TE_122473	0.00000(0/45)	0.00000(0/19)	0.86364(19/22)	0.00000(0/19)
Bo_TE_168094	0.10256(4/39)	0.88235(15/17)	0.47368(9/19)	0.40000(8/20)
Bo_TE_97845	0.67442(29/43)	0.00000(0/19)	0.23810(5/21)	0.15000(3/20)
Bo_TE_178379	0.00000(0/44)	0.00000(0/19)	0.00000(0/23)	0.50000(9/18)
Bo_TE_17997	0.02174(1/46)	0.00000(0/18)	0.55000(11/20)	0.05000(1/20)
Bo_TE_74509	0.00000(0/24)	0.00000(0/10)	0.63158(12/19)	0.05556(1/18)
Bo_TE_4291	0.11111(5/45)	0.22222(4/18)	0.09091(2/22)	0.85000(17/20)
Bo_TE_119765	0.32558(14/43)	0.93750(15/16)	1.00000(23/23)	0.68421(13/19)
Bo_TE_140279	0.22500(9/40)	0.52632(10/19)	0.19048(4/21)	0.00000(0/20)
Bo_TE_39315	0.52273(23/44)	0.20000(3/15)	0.00000(0/23)	0.10000(2/20)
Bo_TE_196645	0.00000(0/43)	0.00000(0/19)	0.00000(0/23)	0.83333(15/18)
Bo_TE_163297	0.26190(11/42)	0.16667(3/18)	0.95455(21/22)	0.77778(14/18)
Bo_TE_71934	0.37209(16/43)	0.56250(9/16)	0.00000(0/23)	0.45000(9/20)
Bo_TE_109180	0.39024(16/41)	0.12500(2/16)	0.75000(12/16)	0.85000(17/20)
Bo_TE_182505	0.04545(2/44)	0.00000(0/18)	0.61905(13/21)	0.31579(6/19)
Bo_TE_121306	0.20513(8/39)	0.13333(2/15)	0.60000(12/20)	0.05000(1/20)
Bo_TE_221303	0.00000(0/44)	0.81250(13/16)	0.00000(0/22)	0.05000(1/20)

Bo_TE_4378	0.70000(28/40)	0.56250(9/16)	0.14286(3/21)	0.88235(15/17)
Bo_TE_38929	0.97778(44/45)	0.89474(17/19)	0.86364(19/22)	0.42105(8/19)
Bo_TE_117337	0.97778(44/45)	0.17647(3/17)	0.33333(7/21)	0.50000(8/16)
Bo_TE_111785	0.63158(24/38)	0.93750(15/16)	0.22727(5/22)	1.00000(18/18)
Bo_TE_142647	0.71795(28/39)	0.94444(17/18)	0.36364(8/22)	0.70000(14/20)
Bo_TE_99173	1.00000(44/44)	0.16667(3/18)	0.75000(15/20)	0.72222(13/18)
Bo_TE_213663	0.61905(26/42)	0.62500(10/16)	0.00000(0/23)	0.05000(1/20)
Bo_TE_52294	0.11364(5/44)	0.06250(1/16)	0.57143(12/21)	0.62500(10/16)
Bo_TE_108180	0.88636(39/44)	0.00000(0/18)	0.09091(2/22)	0.52632(10/19)
Bo_TE_196873	0.06818(3/44)	0.12500(2/16)	0.37500(6/16)	0.88889(16/18)
Bo_TE_143434	1.00000(46/46)	1.00000(18/18)	0.22727(5/22)	0.90000(18/20)
Bo_TE_200541	0.16667(7/42)	0.68421(13/19)	0.55000(11/20)	0.40000(8/20)
Bo_TE_173234	0.57895(22/38)	0.33333(5/15)	0.28571(6/21)	0.00000(0/20)
Bo_TE_87424	0.80952(34/42)	0.36842(7/19)	0.23810(5/21)	0.83333(15/18)
Bo_TE_33277	1.00000(38/38)	0.53333(8/15)	0.13043(3/23)	0.72222(13/18)
Bo_TE_8624	0.83333(35/42)	0.11111(2/18)	0.08696(2/23)	0.11111(2/18)
Bo_TE_111134	0.30233(13/43)	0.46667(7/15)	1.00000(22/22)	0.75000(15/20)
Bo_TE_5270	0.23256(10/43)	0.75000(12/16)	0.38095(8/21)	0.70000(14/20)
Bo_TE_87726	0.72093(31/43)	0.11111(2/18)	0.23810(5/21)	0.00000(0/19)
Bo_TE_229946	0.02222(1/45)	0.00000(0/18)	0.00000(0/23)	0.73684(14/19)
Bo_TE_236213	0.11364(5/44)	0.25000(4/16)	1.00000(22/22)	0.73333(11/15)
Bo_TE_168157	0.33333(13/39)	0.05556(1/18)	0.47619(10/21)	0.65000(13/20)
Bo_TE_117656	0.34146(14/41)	0.64706(11/17)	0.68182(15/22)	0.95000(19/20)
Bo_TE_76203	0.46341(19/41)	0.11765(2/17)	0.85714(18/21)	0.60000(12/20)
Bo_TE_95157	0.44737(17/38)	0.63158(12/19)	0.00000(0/23)	0.10526(2/19)
Bo_TE_61095	0.19048(8/42)	0.52941(9/17)	0.05000(1/20)	0.70000(14/20)
Bo_TE_94957	0.55814(24/43)	0.23529(4/17)	0.00000(0/21)	0.00000(0/20)
Bo_TE_33141	1.00000(45/45)	0.58824(10/17)	0.63636(14/22)	0.16667(3/18)
Bo_TE_130280	0.62791(27/43)	0.15789(3/19)	1.00000(21/21)	0.78947(15/19)
Bo_TE_178692	0.52632(20/38)	0.62500(10/16)	0.36364(8/22)	1.00000(20/20)
Bo_TE_12217	0.00000(0/42)	0.00000(0/18)	0.71429(15/21)	0.11111(2/18)
Bo_TE_226889	0.56410(22/39)	0.38889(7/18)	0.00000(0/23)	0.15000(3/20)
Bo_TE_134181	0.02222(1/45)	0.61111(11/18)	0.04348(1/23)	0.00000(0/20)
Bo_TE_227193	0.91111(41/45)	0.64706(11/17)	0.95455(21/22)	0.15789(3/19)
Bo_TE_232703	0.97727(43/44)	1.00000(19/19)	0.63636(14/22)	0.26316(5/19)
Bo_TE_194382	0.00000(0/43)	0.00000(0/19)	0.63158(12/19)	0.33333(6/18)
Bo_TE_223056	0.38095(16/42)	0.16667(3/18)	0.66667(14/21)	0.00000(0/20)
Bo_TE_112167	0.58537(24/41)	0.11765(2/17)	1.00000(20/20)	0.65000(13/20)
Bo_TE_69752	0.42857(18/42)	0.00000(0/18)	0.25000(5/20)	0.55000(11/20)
Bo_TE_17989	0.00000(0/45)	0.00000(0/18)	0.50000(10/20)	0.05000(1/20)
Bo_TE_140235	0.00000(0/43)	0.37500(6/16)	0.30435(7/23)	0.73684(14/19)
Bo_TE_189187	0.25641(10/39)	0.06250(1/16)	0.52632(10/19)	0.77778(14/18)
Bo_TE_148597	0.79545(35/44)	0.52941(9/17)	0.04545(1/22)	0.00000(0/20)
Bo_TE_33082	0.81579(31/38)	0.42857(6/14)	0.35000(7/20)	1.00000(20/20)
Bo_TE_172562	0.84444(38/45)	0.06667(1/15)	0.40909(9/22)	0.17647(3/17)
Bo_TE_169098	0.72093(31/43)	0.42105(8/19)	1.00000(22/22)	0.66667(12/18)
Bo_TE_147642	0.24444(11/45)	0.50000(9/18)	0.80952(17/21)	0.21053(4/19)
Bo_TE_59114	0.00000(0/45)	0.66667(12/18)	0.63158(12/19)	0.88889(16/18)
Bo_TE_144115	0.04545(2/44)	0.50000(9/18)	0.00000(0/22)	0.00000(0/17)
Bo_TE_41928	0.76744(33/43)	0.00000(0/17)	0.21429(3/14)	0.20000(3/15)
Bo_TE_213832	0.85714(36/42)	0.94118(16/17)	0.31818(7/22)	0.44444(8/18)
Bo_TE_151337	0.97619(41/42)	1.00000(18/18)	0.85000(17/20)	0.45000(9/20)
Bo_TE_53642	0.80952(34/42)	0.94444(17/18)	0.85714(18/21)	0.10526(2/19)
Bo_TE_220463	0.66667(28/42)	0.83333(15/18)	0.42857(9/21)	0.22222(4/18)
Bo_TE_98133	0.60465(26/43)	0.70588(12/17)	0.04348(1/23)	0.47368(9/19)
Bo_TE_100753	0.80952(34/42)	0.88889(16/18)	0.91304(21/23)	0.17647(3/17)

Bo_TE_182343	0.93333(42/45)	0.44444(8/18)	0.42857(9/21)	0.55000(11/20)
Bo_TE_141368	0.79070(34/43)	0.05882(1/17)	0.04762(1/21)	0.21053(4/19)
Bo_TE_13430	0.04762(2/42)	0.25000(2/8)	1.00000(14/14)	0.61111(11/18)
Bo_TE_154269	0.91304(42/46)	0.18750(3/16)	1.00000(23/23)	1.00000(20/20)
Bo_TE_149088	0.15000(6/40)	0.42105(8/19)	1.00000(23/23)	1.00000(20/20)
Bo_TE_201404	0.85366(35/41)	0.70588(12/17)	0.83333(15/18)	0.16667(3/18)
Bo_TE_26815	0.52273(23/44)	1.00000(19/19)	1.00000(22/22)	0.31579(6/19)
Bo_TE_64854	0.17949(7/39)	0.50000(8/16)	0.89474(17/19)	0.72222(13/18)
Bo_TE_14610	0.30000(12/40)	0.40000(6/15)	0.80952(17/21)	0.52632(10/19)
Bo_TE_71144	0.65116(28/43)	0.94118(16/17)	0.71429(15/21)	0.42105(8/19)
Bo_TE_69747	0.52500(21/40)	0.00000(0/18)	0.15000(3/20)	0.50000(8/16)
Bo_TE_92276	0.36364(16/44)	0.50000(7/14)	0.95000(19/20)	0.20000(4/20)
Bo_TE_18097	0.55000(22/40)	0.11111(2/18)	0.00000(0/23)	0.10000(2/20)
Bo_TE_136132	0.65909(29/44)	0.35294(6/17)	0.60000(12/20)	0.00000(0/19)
Bo_TE_164749	0.29545(13/44)	1.00000(17/17)	1.00000(23/23)	0.95000(19/20)
Bo_TE_28921	0.75610(31/41)	0.94118(16/17)	0.90476(19/21)	0.27778(5/18)
Bo_TE_162122	0.00000(0/43)	0.00000(0/18)	0.42857(9/21)	0.84211(16/19)
Bo_TE_101302	0.89744(35/39)	0.75000(12/16)	0.45455(10/22)	0.05263(1/19)
Bo_TE_86701	0.04651(2/43)	0.70588(12/17)	0.33333(7/21)	0.20000(4/20)
Bo_TE_109022	0.84091(37/44)	0.73333(11/15)	0.61905(13/21)	0.10000(2/20)
Bo_TE_172543	0.38095(16/42)	0.05882(1/17)	0.76190(16/21)	0.22222(4/18)
Bo_TE_101280	0.04545(2/44)	0.18750(3/16)	0.42857(9/21)	0.73684(14/19)
Bo_TE_19591	0.86842(33/38)	0.20000(3/15)	0.57143(12/21)	0.15789(3/19)
Bo_TE_178764	0.35897(14/39)	0.06250(1/16)	0.38095(8/21)	0.66667(12/18)
Bo_TE_191912	0.85366(35/41)	0.11111(2/18)	1.00000(21/21)	0.90000(18/20)
Bo_TE_52355	0.95556(43/45)	0.35294(6/17)	0.95455(21/22)	0.76471(13/17)
Bo_TE_77638	0.51220(21/41)	0.07143(1/14)	0.04545(1/22)	0.00000(0/19)
Bo_TE_224191	0.39024(16/41)	0.18750(3/16)	0.13636(3/22)	0.65000(13/20)
Bo_TE_227526	0.93182(41/44)	0.33333(6/18)	0.23810(5/21)	0.89474(17/19)
Bo_TE_56732	0.81818(36/44)	0.56250(9/16)	0.25000(5/20)	0.05000(1/20)
Bo_TE_118359	0.54762(23/42)	0.21053(4/19)	0.00000(0/23)	0.05000(1/20)
Bo_TE_121134	0.69048(29/42)	0.62500(10/16)	0.23810(5/21)	0.11111(2/18)
Bo_TE_191394	0.02174(1/46)	0.50000(8/16)	0.00000(0/23)	0.00000(0/19)
Bo_TE_217988	0.42857(18/42)	0.94118(16/17)	0.28571(6/21)	0.15789(3/19)
Bo_TE_170940	0.41860(18/43)	0.29412(5/17)	0.40000(8/20)	0.90000(18/20)
Bo_TE_179336	0.51111(23/45)	0.35294(6/17)	0.00000(0/23)	0.10526(2/19)
Bo_TE_83994	0.90244(37/41)	1.00000(18/18)	0.42857(9/21)	0.25000(5/20)
Bo_TE_146920	0.26667(12/45)	0.56250(9/16)	1.00000(22/22)	1.00000(19/19)
Bo_TE_236849	0.70455(31/44)	0.41176(7/17)	0.27273(6/22)	0.05000(1/20)
Bo_TE_21349	0.54762(23/42)	0.61538(8/13)	0.08696(2/23)	0.60000(12/20)
Bo_TE_229528	0.00000(0/43)	0.10526(2/19)	0.65000(13/20)	0.66667(12/18)
Bo_TE_162211	0.09302(4/43)	0.64706(11/17)	0.00000(0/22)	0.11111(2/18)
Bo_TE_22279	0.60000(24/40)	0.68750(11/16)	0.33333(7/21)	0.16667(3/18)
Bo_TE_5872	0.75000(33/44)	0.68750(11/16)	0.63636(14/22)	0.05882(1/17)
Bo_TE_177949	0.93023(40/43)	0.28571(4/14)	0.17647(3/17)	0.11111(2/18)
Bo_TE_223120	0.51111(23/45)	0.77778(14/18)	0.13636(3/22)	0.11111(2/18)
Bo_TE_41141	0.00000(0/43)	0.00000(0/18)	0.00000(0/20)	0.50000(9/18)
Bo_TE_63426	0.83333(35/42)	0.72222(13/18)	0.57895(11/19)	0.05000(1/20)
Bo_TE_156594	0.86047(37/43)	0.38889(7/18)	1.00000(20/20)	0.75000(15/20)
Bo_TE_69575	0.55000(22/40)	0.81250(13/16)	0.52381(11/21)	0.05000(1/20)
Bo_TE_95244	0.68293(28/41)	0.68750(11/16)	0.35000(7/20)	0.00000(0/20)
Bo_TE_180067	0.00000(0/45)	0.70588(12/17)	0.66667(14/21)	0.00000(0/20)
Bo_TE_114412	0.62500(25/40)	0.33333(6/18)	0.95238(20/21)	0.52632(10/19)
Bo_TE_15437	0.71429(30/42)	0.05556(1/18)	0.00000(0/23)	0.52632(10/19)
Bo_TE_21932	0.02273(1/44)	0.11765(2/17)	0.57143(12/21)	0.00000(0/19)
Bo_TE_138935	0.81395(35/43)	0.88889(16/18)	1.00000(22/22)	0.42105(8/19)

Bo_TE_223736	0.68293(28/41)	0.86667(13/15)	0.09524(2/21)	0.38889(7/18)
Bo_TE_70148	0.73810(31/42)	0.06250(1/16)	0.76190(16/21)	0.35000(7/20)
Bo_TE_200955	0.06667(3/45)	0.77778(14/18)	0.00000(0/23)	0.20000(4/20)
Bo_TE_76697	0.52500(21/40)	0.50000(8/16)	0.00000(0/23)	0.00000(0/20)
Bo_TE_126137	0.12195(5/41)	0.52941(9/17)	0.00000(0/23)	0.00000(0/20)
Bo_TE_55283	0.11364(5/44)	0.53846(7/13)	0.95238(20/21)	0.83333(15/18)
Bo_TE_178106	0.65116(28/43)	0.38889(7/18)	0.00000(0/23)	0.00000(0/19)
Bo_TE_225286	0.26190(11/42)	0.22222(4/18)	0.61905(13/21)	0.90000(18/20)
Bo_TE_224579	0.32500(13/40)	0.93750(15/16)	0.42857(6/14)	0.47059(8/17)
Bo_TE_180036	0.11111(3/27)	0.10000(1/10)	0.00000(0/22)	0.58824(10/17)
Bo_TE_61831	0.77273(34/44)	0.27778(5/18)	0.00000(0/23)	0.42105(8/19)
Bo_TE_132319	0.25581(11/43)	0.11765(2/17)	1.00000(21/21)	1.00000(19/19)
Bo_TE_201642	0.88372(38/43)	0.82353(14/17)	0.04762(1/21)	0.00000(0/20)
Bo_TE_209922	0.00000(0/39)	0.00000(0/16)	0.45455(10/22)	0.85000(17/20)
Bo_TE_92756	0.30952(13/42)	0.75000(12/16)	0.36364(8/22)	0.83333(15/18)
Bo_TE_41171	0.04444(2/45)	0.75000(12/16)	0.00000(0/23)	0.05263(1/19)
Bo_TE_91095	0.65854(27/41)	0.16667(3/18)	0.71429(15/21)	0.84211(16/19)
Bo_TE_121438	1.00000(46/46)	1.00000(19/19)	0.95652(22/23)	0.25000(5/20)
Bo_TE_10725	0.00000(0/45)	0.11111(2/18)	0.85000(17/20)	0.10000(2/20)
Bo_TE_184828	0.32500(13/40)	0.28571(4/14)	0.33333(7/21)	0.84211(16/19)
Bo_TE_84230	0.00000(0/45)	0.00000(0/19)	0.57143(12/21)	0.00000(0/20)
Bo_TE_47905	0.51282(20/39)	0.11765(2/17)	1.00000(21/21)	0.80000(16/20)
Bo_TE_4169	0.44186(19/43)	0.87500(14/16)	0.15000(3/20)	0.00000(0/20)
Bo_TE_39119	0.06818(3/44)	0.06250(1/16)	0.61905(13/21)	0.88889(16/18)
Bo_TE_232067	0.00000(0/44)	0.05263(1/19)	0.34783(8/23)	0.52632(10/19)
Bo_TE_186602	0.85366(35/41)	0.76923(10/13)	0.13636(3/22)	0.83333(15/18)
Bo_TE_14179	0.77500(31/40)	0.87500(14/16)	0.76190(16/21)	0.05263(1/19)
Bo_TE_81886	0.11628(5/43)	0.93750(15/16)	0.86364(19/22)	0.88889(16/18)
Bo_TE_180723	0.04545(2/44)	0.22222(4/18)	0.52381(11/21)	0.78947(15/19)
Bo_TE_129396	0.72500(29/40)	0.00000(0/17)	0.00000(0/23)	0.00000(0/19)
Bo_TE_22800	0.93023(40/43)	0.38889(7/18)	1.00000(22/22)	0.95000(19/20)
Bo_TE_100764	0.17073(7/41)	0.06250(1/16)	0.08696(2/23)	0.84211(16/19)
Bo_TE_16166	0.42857(18/42)	0.93750(15/16)	0.28571(6/21)	0.95000(19/20)
Bo_TE_112336	0.00000(0/45)	0.22222(4/18)	0.73684(14/19)	0.55556(10/18)
Bo_TE_203630	0.62791(27/43)	0.82353(14/17)	0.40000(8/20)	0.10000(2/20)
Bo_TE_138864	0.73333(33/45)	0.83333(15/18)	0.57143(12/21)	0.15000(3/20)
Bo_TE_221770	1.00000(45/45)	0.73333(11/15)	0.95455(21/22)	0.11765(2/17)
Bo_TE_141093	0.09091(4/44)	0.91667(11/12)	0.22222(4/18)	0.81250(13/16)
Bo_TE_100494	0.52632(20/38)	0.46154(6/13)	0.33333(7/21)	0.00000(0/18)
Bo_TE_109723	0.16279(7/43)	0.11111(2/18)	0.30000(6/20)	0.75000(15/20)
Bo_TE_108941	0.70732(29/41)	0.68750(11/16)	0.31818(7/22)	0.15000(3/20)
Bo_TE_148110	0.13636(6/44)	0.88889(16/18)	0.61905(13/21)	0.00000(0/18)
Bo_TE_13035	0.13953(6/43)	0.93750(15/16)	0.80952(17/21)	0.72222(13/18)
Bo_TE_135363	0.51220(21/41)	0.16667(3/18)	0.00000(0/21)	0.00000(0/19)
Bo_TE_230391	0.26829(11/41)	0.27778(5/18)	0.80952(17/21)	0.05000(1/20)
Bo_TE_54368	0.95349(41/43)	0.43750(7/16)	0.66667(14/21)	0.42105(8/19)
Bo_TE_50750	0.09091(4/44)	0.22222(4/18)	0.60000(12/20)	0.33333(6/18)
Bo_TE_162288	0.09091(4/44)	0.64706(11/17)	0.00000(0/21)	0.10526(2/19)
Bo_TE_216156	0.93333(42/45)	0.70588(12/17)	0.82609(19/23)	0.42105(8/19)
Bo_TE_129593	0.87805(36/41)	0.94444(17/18)	1.00000(18/18)	0.36842(7/19)
Bo_TE_193321	0.34884(15/43)	0.58824(10/17)	0.20000(4/20)	0.05000(1/20)
Bo_TE_158081	0.02174(1/46)	0.00000(0/18)	0.50000(11/22)	0.10526(2/19)
Bo_TE_326	0.30952(13/42)	0.75000(12/16)	0.00000(0/22)	0.00000(0/19)
Bo_TE_190934	0.34146(14/41)	0.55556(10/18)	0.85714(18/21)	0.20000(4/20)
Bo_TE_185893	0.84091(37/44)	0.11111(2/18)	0.57895(11/19)	0.11765(2/17)
Bo_TE_72371	0.80435(37/46)	0.60000(9/15)	0.09091(2/22)	0.05263(1/19)

Bo_TE_195383	0.17073(7/41)	0.68750(11/16)	0.00000(0/22)	0.52632(10/19)
Bo_TE_63978	0.52381(22/42)	0.05556(1/18)	0.00000(0/21)	0.10000(2/20)
Bo_TE_145329	0.27500(11/40)	0.00000(0/19)	0.42857(9/21)	0.65000(13/20)
Bo_TE_156087	0.54348(25/46)	0.11111(2/18)	0.95000(19/20)	0.63158(12/19)
Bo_TE_104706	0.67442(29/43)	0.76471(13/17)	0.04762(1/21)	0.25000(5/20)
Bo_TE_36893	0.60976(25/41)	0.50000(8/16)	0.68182(15/22)	1.00000(20/20)
Bo_TE_5034	0.43590(17/39)	1.00000(19/19)	1.00000(23/23)	1.00000(20/20)
Bo_TE_188375	0.68421(26/38)	0.64706(11/17)	0.00000(0/22)	0.15789(3/19)
Bo_TE_230382	0.22727(10/44)	0.27778(5/18)	0.80952(17/21)	0.05000(1/20)
Bo_TE_104989	0.32609(15/46)	1.00000(17/17)	0.66667(14/21)	0.42105(8/19)
Bo_TE_33990	0.34146(14/41)	0.58824(10/17)	0.23810(5/21)	0.85000(17/20)
Bo_TE_135776	0.18605(8/43)	0.16667(3/18)	0.55000(11/20)	0.68421(13/19)
Bo_TE_98754	0.11905(5/42)	0.23529(4/17)	0.42105(8/19)	0.85000(17/20)
Bo_TE_200130	0.84211(32/38)	0.05556(1/18)	0.19048(4/21)	0.15000(3/20)
Bo_TE_95293	0.06818(3/44)	0.68421(13/19)	0.00000(0/22)	0.11111(2/18)
Bo_TE_162888	0.70455(31/44)	0.50000(9/18)	0.68182(15/22)	0.15000(3/20)
Bo_TE_149482	0.09302(4/43)	0.35294(6/17)	0.75000(15/20)	0.00000(0/20)
Bo_TE_27354	0.14286(6/42)	0.06667(1/15)	0.54545(12/22)	0.95000(19/20)
Bo_TE_138529	0.28571(12/42)	0.17647(3/17)	0.91304(21/23)	0.65000(13/20)
Bo_TE_188268	0.02273(1/44)	0.27778(5/18)	0.20000(4/20)	0.72222(13/18)
Bo_TE_224645	0.41026(16/39)	0.83333(15/18)	0.30000(6/20)	0.88889(16/18)
Bo_TE_92904	0.65116(28/43)	0.87500(14/16)	0.11111(2/18)	0.15000(3/20)
Bo_TE_207188	0.38095(16/42)	0.66667(12/18)	0.65000(13/20)	0.05263(1/19)
Bo_TE_158313	0.04545(2/44)	0.18750(3/16)	0.59091(13/22)	0.72222(13/18)
Bo_TE_196327	0.83333(35/42)	0.64706(11/17)	1.00000(21/21)	0.37500(6/16)
Bo_TE_7742	0.58537(24/41)	0.41176(7/17)	0.40000(8/20)	0.05000(1/20)
Bo_TE_34673	0.97674(42/43)	0.73333(11/15)	0.42857(9/21)	0.17647(3/17)
Bo_TE_20238	0.08889(4/45)	0.81250(13/16)	0.14286(3/21)	0.25000(5/20)
Bo_TE_37495	0.00000(0/43)	0.00000(0/19)	0.25000(5/20)	0.72222(13/18)
Bo_TE_200878	0.11111(5/45)	0.00000(0/17)	0.04348(1/23)	0.80000(16/20)
Bo_TE_27792	0.17778(8/45)	0.00000(0/18)	0.52381(11/21)	0.84211(16/19)
Bo_TE_48001	0.00000(0/44)	0.00000(0/18)	0.60870(14/23)	0.70000(14/20)
Bo_TE_115130	0.90476(38/42)	0.22222(4/18)	0.85000(17/20)	0.78947(15/19)
Bo_TE_162095	0.58974(23/39)	0.58824(10/17)	0.42857(9/21)	0.05556(1/18)
Bo_TE_12331	0.60000(18/30)	0.62500(10/16)	0.10526(2/19)	0.62500(10/16)
Bo_TE_41930	0.28261(13/46)	0.94118(16/17)	0.88235(15/17)	0.80000(16/20)
Bo_TE_80202	1.00000(43/43)	0.36842(7/19)	1.00000(22/22)	0.22222(4/18)
Bo_TE_204978	0.36111(13/36)	0.15789(3/19)	0.33333(7/21)	0.94737(18/19)
Bo_TE_159429	0.23810(10/42)	0.72222(13/18)	0.71429(15/21)	0.76471(13/17)
Bo_TE_80745	0.31818(14/44)	0.00000(0/19)	0.52381(11/21)	0.00000(0/18)
Bo_TE_84443	0.20000(9/45)	0.00000(0/17)	0.52381(11/21)	0.35000(7/20)
Bo_TE_225171	0.60000(24/40)	0.05882(1/17)	0.00000(0/22)	0.05263(1/19)
Bo_TE_107850	0.71429(30/42)	0.22222(4/18)	0.00000(0/23)	0.00000(0/20)
Bo_TE_186244	0.15556(7/45)	0.05556(1/18)	0.57895(11/19)	0.05000(1/20)
Bo_TE_6735	0.87805(36/41)	0.06667(1/15)	0.85714(18/21)	0.15000(3/20)
Bo_TE_18037	0.57143(24/42)	0.25000(4/16)	0.00000(0/23)	0.05000(1/20)
Bo_TE_240150	0.02381(1/42)	0.52632(10/19)	0.04348(1/23)	0.05000(1/20)
Bo_TE_89581	0.36585(15/41)	0.88889(16/18)	0.35000(7/20)	0.05263(1/19)
Bo_TE_16873	0.11111(5/45)	0.16667(3/18)	1.00000(23/23)	0.94737(18/19)
Bo_TE_45150	0.40000(16/40)	0.31250(5/16)	0.00000(0/22)	0.61111(11/18)
Bo_TE_144075	0.82927(34/41)	0.56250(9/16)	0.00000(0/21)	0.05000(1/20)
Bo_TE_120400	0.88372(38/43)	0.82353(14/17)	0.58824(10/17)	0.27778(5/18)
Bo_TE_177487	0.34091(15/44)	0.56250(9/16)	0.85714(18/21)	0.27778(5/18)
Bo_TE_116265	0.02273(1/44)	0.47368(9/19)	0.57143(12/21)	0.31579(6/19)
Bo_TE_225208	0.20455(9/44)	0.52941(9/17)	0.80000(16/20)	0.47368(9/19)
Bo_TE_138514	0.28571(12/42)	0.16667(3/18)	0.91304(21/23)	0.63158(12/19)

Bo_TE_76600	0.04444(2/45)	0.50000(9/18)	0.52381(11/21)	0.00000(0/20)
Bo_TE_227282	0.04545(2/44)	0.00000(0/19)	0.66667(14/21)	0.00000(0/19)
Bo_TE_18929	0.45000(18/40)	0.23529(4/17)	0.95652(22/23)	0.25000(5/20)
Bo_TE_160984	0.77778(35/45)	0.50000(8/16)	0.28571(6/21)	0.05556(1/18)
Bo_TE_204541	0.31707(13/41)	0.11765(2/17)	0.00000(0/18)	0.78947(15/19)
Bo_TE_21926	0.37209(16/43)	0.17647(3/17)	0.40000(8/20)	1.00000(20/20)
Bo_TE_211449	0.77273(34/44)	0.66667(12/18)	0.18182(4/22)	1.00000(20/20)
Bo_TE_122141	0.59459(22/37)	0.37500(6/16)	0.00000(0/20)	0.50000(8/16)
Bo_TE_72759	0.62791(27/43)	0.31250(5/16)	0.43478(10/23)	0.94737(18/19)
Bo_TE_177608	1.00000(45/45)	1.00000(18/18)	0.60000(12/20)	0.47059(8/17)
Bo_TE_28995	0.53333(24/45)	0.22222(4/18)	0.09524(2/21)	0.84211(16/19)
Bo_TE_226897	0.00000(0/43)	0.31250(5/16)	0.86364(19/22)	0.29412(5/17)
Bo_TE_156306	1.00000(39/39)	1.00000(19/19)	0.52381(11/21)	0.31579(6/19)
Bo_TE_95697	0.93478(43/46)	1.00000(19/19)	1.00000(23/23)	0.30000(6/20)
Bo_TE_227688	0.85366(35/41)	0.94444(17/18)	0.14286(3/21)	0.15000(3/20)
Bo_TE_18968	0.46512(20/43)	0.75000(12/16)	0.61111(11/18)	0.10000(2/20)
Bo_TE_184841	0.86047(37/43)	0.41176(7/17)	0.30000(6/20)	1.00000(20/20)
Bo_TE_130107	0.02273(1/44)	0.00000(0/18)	0.75000(15/20)	0.65000(13/20)
Bo_TE_8946	0.88372(38/43)	0.37500(6/16)	0.00000(0/23)	0.05263(1/19)
Bo_TE_191076	0.05000(2/40)	0.11111(2/18)	0.17391(4/23)	0.63158(12/19)
Bo_TE_220554	0.40476(17/42)	0.16667(3/18)	0.47619(10/21)	0.73684(14/19)
Bo_TE_210881	0.41667(15/36)	0.83333(15/18)	0.28571(6/21)	0.10000(2/20)
Bo_TE_126088	0.75610(31/41)	0.83333(15/18)	0.00000(0/21)	0.05556(1/18)
Bo_TE_200030	0.89130(41/46)	0.23529(4/17)	0.71429(15/21)	0.20000(4/20)
Bo_TE_240738	0.14634(6/41)	0.73684(14/19)	0.68182(15/22)	0.85000(17/20)
Bo_TE_45699	0.15556(7/45)	0.84211(16/19)	1.00000(20/20)	0.90000(18/20)
Bo_TE_145736	0.06522(3/46)	0.63158(12/19)	0.00000(0/23)	0.00000(0/20)
Bo_TE_148133	0.70000(28/40)	0.00000(0/16)	0.40000(8/20)	0.85000(17/20)
Bo_TE_25629	0.00000(0/43)	0.05556(1/18)	0.04762(1/21)	0.75000(15/20)
Bo_TE_115256	0.57143(24/42)	0.81250(13/16)	0.14286(3/21)	0.26316(5/19)
Bo_TE_102201	0.93182(41/44)	0.73333(11/15)	0.60000(12/20)	0.21053(4/19)
Bo_TE_55681	0.32500(13/40)	0.75000(12/16)	0.91304(21/23)	0.68421(13/19)
Bo_TE_22382	0.02222(1/45)	0.47059(8/17)	0.71429(15/21)	0.00000(0/20)
Bo_TE_39765	0.88636(39/44)	0.05882(1/17)	0.14286(3/21)	0.68421(13/19)
Bo_TE_156238	0.16279(7/43)	0.44444(8/18)	0.55000(11/20)	0.68421(13/19)
Bo_TE_57019	0.50000(21/42)	0.05556(1/18)	0.04545(1/22)	0.00000(0/19)
Bo_TE_81996	0.00000(0/41)	0.00000(0/18)	0.65000(13/20)	0.22222(4/18)
Bo_TE_2523	0.16667(7/42)	0.06250(1/16)	0.04762(1/21)	0.89474(17/19)
Bo_TE_84100	0.70455(31/44)	0.00000(0/17)	0.15000(3/20)	0.00000(0/20)
Bo_TE_17509	0.06667(3/45)	0.76471(13/17)	0.86364(19/22)	0.73684(14/19)
Bo_TE_151193	0.84091(37/44)	0.18750(3/16)	0.13636(3/22)	0.55556(10/18)
Bo_TE_143553	0.69767(30/43)	0.64706(11/17)	1.00000(23/23)	0.36842(7/19)
Bo_TE_99098	0.64286(27/42)	0.52941(9/17)	0.21739(5/23)	0.05263(1/19)
Bo_TE_34772	0.73171(30/41)	1.00000(18/18)	0.54545(12/22)	0.40000(8/20)
Bo_TE_210939	0.51351(19/37)	0.00000(0/16)	0.00000(0/13)	0.00000(0/9)
Bo_TE_107249	0.75000(33/44)	0.80000(12/15)	0.22727(5/22)	0.10000(2/20)
Bo_TE_239825	0.00000(0/46)	0.00000(0/19)	0.76190(16/21)	0.25000(5/20)
Bo_TE_143654	0.93182(41/44)	0.53333(8/15)	0.33333(7/21)	0.45000(9/20)
Bo_TE_223348	0.00000(0/45)	0.00000(0/18)	0.36364(8/22)	0.72222(13/18)
Bo_TE_63587	0.85000(34/40)	0.11765(2/17)	0.52381(11/21)	1.00000(19/19)
Bo_TE_44517	1.00000(42/42)	0.44444(8/18)	0.50000(11/22)	0.85000(17/20)
Bo_TE_53152	0.00000(0/45)	0.64706(11/17)	0.52174(12/23)	0.05000(1/20)
Bo_TE_148543	0.04444(2/45)	0.00000(0/5)	0.00000(0/23)	0.83333(15/18)
Bo_TE_37417	0.00000(0/45)	0.64706(11/17)	0.00000(0/21)	0.05263(1/19)
Bo_TE_123421	0.04545(2/44)	0.16667(3/18)	0.77273(17/22)	0.63158(12/19)
Bo_TE_174505	0.34146(14/41)	0.05556(1/18)	0.00000(0/22)	0.78947(15/19)

Bo_TE_223173	0.15909(7/44)	0.23077(3/13)	0.47368(9/19)	1.00000(18/18)
Bo_TE_99230	0.00000(0/44)	0.27778(5/18)	0.52174(12/23)	0.30000(6/20)
Bo_TE_830	0.00000(0/45)	0.00000(0/18)	0.18182(4/22)	0.77778(14/18)
Bo_TE_150821	1.00000(44/44)	0.72222(13/18)	0.13636(3/22)	0.55000(11/20)
Bo_TE_182201	0.20000(9/45)	0.72222(13/18)	0.00000(0/20)	0.30000(6/20)
Bo_TE_230397	0.02381(1/42)	0.05882(1/17)	0.10000(2/20)	0.52632(10/19)
Bo_TE_214327	0.73171(30/41)	0.18750(3/16)	0.34783(8/23)	0.42105(8/19)
Bo_TE_225377	0.31818(14/44)	0.00000(0/17)	0.52174(12/23)	0.00000(0/20)
Bo_TE_45629	0.54054(20/37)	0.05556(1/18)	0.22727(5/22)	0.00000(0/18)
Bo_TE_191724	0.50000(22/44)	0.58333(7/12)	0.00000(0/22)	0.00000(0/18)
Bo_TE_16431	0.50000(20/40)	0.22222(4/18)	0.21739(5/23)	0.73684(14/19)
Bo_TE_170495	0.00000(0/42)	0.12500(2/16)	0.54545(12/22)	0.45000(9/20)
Bo_TE_94664	0.22857(8/35)	0.47059(8/17)	1.00000(23/23)	0.36842(7/19)
Bo_TE_230064	0.83721(36/43)	0.11765(2/17)	0.42857(9/21)	0.15000(3/20)
Bo_TE_190880	0.00000(0/41)	0.16667(3/18)	0.85714(18/21)	0.00000(0/19)
Bo_TE_129420	0.82500(33/40)	0.00000(0/17)	0.00000(0/23)	0.00000(0/19)
Bo_TE_230833	0.20930(9/43)	0.11111(2/18)	1.00000(23/23)	1.00000(20/20)
Bo_TE_138253	0.23077(9/39)	0.00000(0/19)	0.19048(4/21)	0.89474(17/19)
Bo_TE_77759	0.00000(0/46)	0.56250(9/16)	0.00000(0/23)	0.00000(0/20)
Bo_TE_199145	0.00000(0/46)	0.64706(11/17)	0.09524(2/21)	0.33333(6/18)
Bo_TE_204880	0.33333(13/39)	0.76471(13/17)	0.57143(12/21)	0.05556(1/18)
Bo_TE_145524	0.36957(17/46)	0.70588(12/17)	0.00000(0/22)	0.00000(0/20)
Bo_TE_159268	0.11364(5/44)	0.00000(0/18)	0.15000(3/20)	0.89474(17/19)
Bo_TE_25697	0.20000(9/45)	0.52941(9/17)	0.00000(0/23)	0.55000(11/20)
Bo_TE_231180	0.84091(37/44)	0.33333(6/18)	0.82609(19/23)	0.80000(16/20)
Bo_TE_182405	0.04444(2/45)	0.21053(4/19)	0.57143(12/21)	0.45000(9/20)
Bo_TE_168747	0.72727(32/44)	0.37500(6/16)	0.70000(14/20)	0.11111(2/18)
Bo_TE_83911	0.09756(4/41)	0.00000(0/17)	0.58824(10/17)	0.77778(14/18)
Bo_TE_21700	0.02174(1/46)	0.16667(3/18)	0.21739(5/23)	0.65000(13/20)
Bo_TE_71228	0.45000(18/40)	1.00000(17/17)	0.28571(6/21)	0.80000(16/20)
Bo_TE_138809	0.18182(8/44)	0.68750(11/16)	0.43478(10/23)	0.11765(2/17)
Bo_TE_77083	0.82927(34/41)	0.56250(9/16)	0.64706(11/17)	0.05556(1/18)
Bo_TE_182232	0.21429(9/42)	0.70588(12/17)	0.00000(0/22)	0.29412(5/17)
Bo_TE_8210	0.19512(8/41)	0.82353(14/17)	1.00000(23/23)	1.00000(20/20)
Bo_TE_170978	0.42857(18/42)	0.11111(2/18)	0.33333(7/21)	0.90000(18/20)
Bo_TE_39286	0.70000(28/40)	0.64286(9/14)	0.13636(3/22)	0.00000(0/19)
Bo_TE_234979	0.47500(19/40)	0.80000(12/15)	1.00000(22/22)	0.68421(13/19)
Bo_TE_65533	0.84091(37/44)	0.27778(5/18)	0.80952(17/21)	0.23529(4/17)
Bo_TE_79480	0.70455(31/44)	0.52941(9/17)	0.60000(12/20)	0.15000(3/20)
Bo_TE_154289	0.06977(3/43)	0.68750(11/16)	0.00000(0/23)	0.00000(0/20)
Bo_TE_180400	0.61905(26/42)	0.05882(1/17)	0.00000(0/9)	0.37500(3/8)
Bo_TE_58526	0.00000(0/44)	0.29412(5/17)	0.73684(14/19)	0.00000(0/20)
Bo_TE_224248	0.34091(15/44)	0.25000(4/16)	0.04348(1/23)	0.56250(9/16)
Bo_TE_200705	0.20000(8/40)	0.60000(9/15)	0.85714(18/21)	0.50000(10/20)
Bo_TE_180170	1.00000(43/43)	1.00000(18/18)	0.00000(0/17)	0.27778(5/18)
Bo_TE_168654	0.55814(24/43)	1.00000(18/18)	0.33333(7/21)	0.20000(4/20)
Bo_TE_90847	0.51220(21/41)	0.05263(1/19)	0.00000(0/23)	0.00000(0/20)
Bo_TE_213565	0.79545(35/44)	0.86667(13/15)	0.36364(8/22)	0.90000(18/20)
Bo_TE_193426	0.31818(14/44)	0.57895(11/19)	0.23810(5/21)	0.05000(1/20)
Bo_TE_67708	0.78947(30/38)	0.94444(17/18)	0.21053(4/19)	0.89474(17/19)
Bo_TE_37570	0.78049(32/41)	0.93750(15/16)	0.40000(8/20)	1.00000(20/20)
Bo_TE_49816	0.02174(1/46)	0.00000(0/19)	0.57143(12/21)	0.00000(0/20)
Bo_TE_191370	0.02778(1/36)	0.58824(10/17)	0.23810(5/21)	0.89474(17/19)
Bo_TE_210247	0.00000(0/46)	0.00000(0/19)	0.75000(15/20)	0.10526(2/19)
Bo_TE_65340	0.69048(29/42)	0.12500(2/16)	0.42857(9/21)	0.35000(7/20)
Bo_TE_85701	0.04545(2/44)	0.58824(10/17)	0.86364(19/22)	0.52632(10/19)

Bo_TE_164971	1.00000(43/43)	1.00000(17/17)	0.55000(11/20)	0.25000(5/20)
Bo_TE_132990	0.58537(24/41)	0.61111(11/18)	0.15000(3/20)	0.68421(13/19)
Bo_TE_229861	0.50000(22/44)	0.05882(1/17)	0.40909(9/22)	0.84211(16/19)
Bo_TE_107862	0.26087(12/46)	0.73684(14/19)	1.00000(23/23)	1.00000(20/20)
Bo_TE_119586	0.35714(15/42)	0.93750(15/16)	0.31818(7/22)	0.58824(10/17)
Bo_TE_63932	0.47727(21/44)	0.70588(12/17)	0.25000(5/20)	0.05556(1/18)
Bo_TE_79707	0.08889(4/45)	0.16667(3/18)	0.59091(13/22)	0.15000(3/20)
Bo_TE_224502	0.00000(0/45)	0.16667(3/18)	0.19048(4/21)	0.75000(15/20)
Bo_TE_52609	0.35714(15/42)	0.12500(2/16)	0.28571(6/21)	0.66667(12/18)
Bo_TE_24188	0.43902(18/41)	0.66667(12/18)	0.60000(12/20)	0.10526(2/19)
Bo_TE_172348	0.58140(25/43)	0.88889(16/18)	0.14286(3/21)	0.65000(13/20)
Bo_TE_45644	0.60465(26/43)	0.06667(1/15)	0.22727(5/22)	0.00000(0/19)
Bo_TE_73166	0.52632(20/38)	0.00000(0/17)	0.42105(8/19)	0.27778(5/18)
Bo_TE_5030	0.57143(24/42)	0.00000(0/18)	0.00000(0/23)	0.00000(0/19)
Bo_TE_32661	0.11364(5/44)	0.62500(10/16)	0.26316(5/19)	0.50000(9/18)
Bo_TE_8016	0.04878(2/41)	0.00000(0/17)	0.33333(6/18)	0.56250(9/16)
Bo_TE_190628	0.92857(39/42)	0.11111(2/18)	0.63636(14/22)	0.10526(2/19)
Bo_TE_216172	0.97778(44/45)	0.66667(12/18)	0.90909(20/22)	0.44444(8/18)
Bo_TE_124134	0.79545(35/44)	0.23529(4/17)	0.19048(4/21)	0.15000(3/20)
Bo_TE_219916	0.06977(3/43)	0.27778(5/18)	0.57895(11/19)	0.61111(11/18)
Bo_TE_16832	0.39474(15/38)	0.35294(6/17)	0.84211(16/19)	0.31579(6/19)
Bo_TE_225994	0.07143(3/42)	0.55556(10/18)	0.50000(10/20)	0.89474(17/19)
Bo_TE_100015	0.31707(13/41)	0.00000(0/11)	0.70000(14/20)	0.88889(16/18)
Bo_TE_87275	0.06818(3/44)	0.16667(3/18)	0.23810(5/21)	0.80000(16/20)
Bo_TE_199631	0.84091(37/44)	0.33333(6/18)	1.00000(20/20)	0.44444(8/18)
Bo_TE_227613	0.64286(27/42)	0.12500(2/16)	0.00000(0/22)	0.00000(0/20)
Bo_TE_59776	0.81818(36/44)	0.31250(5/16)	0.77273(17/22)	0.20000(4/20)
Bo_TE_133710	0.93182(41/44)	0.82353(14/17)	0.19048(4/21)	0.20000(4/20)
Bo_TE_12145	0.00000(0/45)	0.00000(0/17)	0.00000(0/22)	0.50000(10/20)
Bo_TE_58042	0.02326(1/43)	0.00000(0/18)	0.09091(2/22)	0.63158(12/19)
Bo_TE_152183	0.66667(28/42)	0.11765(2/17)	0.13636(3/22)	0.00000(0/19)
Bo_TE_152414	0.76744(33/43)	0.05263(1/19)	0.00000(0/23)	0.05000(1/20)
Bo_TE_234870	0.00000(0/44)	0.00000(0/18)	0.04762(1/21)	0.52632(10/19)
Bo_TE_14650	0.16279(7/43)	0.23529(4/17)	0.80952(17/21)	0.94444(17/18)
Bo_TE_52227	0.04444(2/45)	0.31250(5/16)	0.57143(12/21)	0.05263(1/19)
Bo_TE_25652	0.45238(19/42)	0.68750(11/16)	0.00000(0/20)	0.10000(2/20)
Bo_TE_196536	0.30000(12/40)	0.44444(8/18)	1.00000(23/23)	1.00000(20/20)
Bo_TE_49976	0.20455(9/44)	0.00000(0/19)	0.00000(0/20)	0.64706(11/17)
Bo_TE_101535	0.84444(38/45)	0.33333(4/12)	0.47826(11/23)	0.15789(3/19)
Bo_TE_178306	0.09091(4/44)	0.36842(7/19)	0.80000(16/20)	0.10000(2/20)
Bo_TE_121784	0.60000(27/45)	1.00000(18/18)	0.95652(22/23)	0.15789(3/19)
Bo_TE_74617	0.00000(0/45)	0.44444(8/18)	0.57143(12/21)	0.00000(0/20)
Bo_TE_17047	0.76087(35/46)	0.00000(0/12)	0.00000(0/19)	0.16667(3/18)
Bo_TE_205143	0.78571(33/42)	0.31250(5/16)	0.45000(9/20)	0.10000(2/20)
Bo_TE_85284	0.58824(10/17)	0.00000(0/18)	0.60000(6/10)	0.88889(16/18)
Bo_TE_193819	0.39535(17/43)	0.77778(14/18)	0.81818(18/22)	0.15000(3/20)
Bo_TE_216620	0.00000(0/46)	0.05263(1/19)	0.52381(11/21)	0.30000(6/20)
Bo_TE_128635	0.21429(9/42)	0.52941(9/17)	0.00000(0/22)	0.00000(0/20)
Bo_TE_76336	0.66667(28/42)	0.06250(1/16)	0.81818(18/22)	0.42105(8/19)
Bo_TE_191485	0.69048(29/42)	0.00000(0/19)	0.00000(0/22)	0.00000(0/20)
Bo_TE_113885	0.00000(0/46)	0.11111(2/18)	0.08696(2/23)	0.63158(12/19)
Bo_TE_10819	0.97500(39/40)	0.81250(13/16)	0.91304(21/23)	0.17647(3/17)
Bo_TE_132792	0.65909(29/44)	0.23529(4/17)	0.14286(3/21)	0.17647(3/17)
Bo_TE_145104	0.39535(17/43)	0.11111(2/18)	0.90000(18/20)	0.20000(4/20)
Bo_TE_24472	0.66667(28/42)	0.84211(16/19)	0.00000(0/23)	0.15789(3/19)
Bo_TE_207252	0.39535(17/43)	0.68421(13/19)	0.66667(14/21)	0.15000(3/20)

Bo_TE_189804	0.02222(1/45)	0.15789(3/19)	0.60000(12/20)	0.00000(0/20)
Bo_TE_104968	0.65116(28/43)	0.00000(0/16)	0.26316(5/19)	0.60000(12/20)
Bo_TE_133748	0.38095(16/42)	0.11111(2/18)	0.47619(10/21)	0.73684(14/19)
Bo_TE_153297	0.65909(29/44)	0.29412(5/17)	0.09524(2/21)	0.72222(13/18)
Bo_TE_184904	0.00000(0/45)	0.60000(9/15)	0.65000(13/20)	0.00000(0/20)
Bo_TE_227157	0.37209(16/43)	0.11111(2/18)	0.04545(1/22)	0.73684(14/19)
Bo_TE_3175	0.02381(1/42)	0.62500(10/16)	0.04762(1/21)	0.20000(4/20)
Bo_TE_200049	0.04444(2/45)	0.05556(1/18)	0.00000(0/21)	0.65000(13/20)
Bo_TE_209677	0.65000(26/40)	0.83333(15/18)	0.71429(15/21)	0.10000(2/20)
Bo_TE_158610	0.52500(21/40)	0.12500(2/16)	0.94737(18/19)	0.27778(5/18)
Bo_TE_162865	0.00000(0/43)	0.00000(0/18)	0.31818(7/22)	0.65000(13/20)
Bo_TE_104436	0.95652(44/46)	1.00000(17/17)	0.38095(8/21)	1.00000(20/20)
Bo_TE_129852	0.45714(16/35)	0.82353(14/17)	0.25000(3/12)	0.11765(2/17)
Bo_TE_153419	0.48718(19/39)	0.63158(12/19)	0.00000(0/17)	0.00000(0/18)
Bo_TE_83703	0.08889(4/45)	0.05263(1/19)	0.75000(15/20)	0.21053(4/19)
Bo_TE_17058	0.02174(1/46)	0.05556(1/18)	0.23810(5/21)	0.80000(16/20)
Bo_TE_136129	0.32558(14/43)	0.60000(9/15)	0.33333(7/21)	0.85000(17/20)
Bo_TE_102099	0.22500(9/40)	0.94444(17/18)	0.81818(18/22)	1.00000(19/19)
Bo_TE_204261	0.50000(20/40)	0.16667(3/18)	0.00000(0/22)	0.05000(1/20)
Bo_TE_112197	0.20455(9/44)	0.52941(9/17)	0.82609(19/23)	0.58824(10/17)
Bo_TE_45695	0.20000(1/5)	0.00000(0/14)	0.40000(8/20)	0.94118(16/17)
Bo_TE_92776	0.08889(4/45)	0.73333(11/15)	0.04545(1/22)	0.55556(10/18)
Bo_TE_17869	0.74419(32/43)	0.93750(15/16)	0.95652(22/23)	0.42105(8/19)
Bo_TE_235074	0.00000(0/43)	0.11111(2/18)	0.09524(2/21)	0.68421(13/19)
Bo_TE_42795	0.27273(12/44)	0.72222(13/18)	0.09091(2/22)	0.00000(0/20)
Bo_TE_89950	0.87179(34/39)	0.60000(9/15)	0.23810(5/21)	0.21053(4/19)
Bo_TE_156658	0.41860(18/43)	0.26316(5/19)	0.04545(1/22)	0.89474(17/19)
Bo_TE_223399	0.51163(22/43)	0.11111(2/18)	0.00000(0/23)	0.05263(1/19)
Bo_TE_19653	0.38095(16/42)	0.18750(3/16)	0.95652(22/23)	0.77778(14/18)
Bo_TE_156071	0.51163(22/43)	0.11111(2/18)	1.00000(22/22)	0.84211(16/19)
Bo_TE_82148	0.19048(8/42)	0.23529(4/17)	0.61905(13/21)	1.00000(18/18)
Bo_TE_138149	0.67442(29/43)	0.05556(1/18)	0.00000(0/23)	0.00000(0/19)
Bo_TE_43006	0.88636(39/44)	0.64706(11/17)	0.91304(21/23)	0.10526(2/19)
Bo_TE_189010	0.60976(25/41)	0.37500(6/16)	1.00000(22/22)	1.00000(20/20)
Bo_TE_66038	0.57778(26/45)	0.75000(12/16)	0.86364(19/22)	0.31579(6/19)
Bo_TE_232374	1.00000(45/45)	0.70588(12/17)	0.59091(13/22)	0.50000(10/20)
Bo_TE_191118	0.56757(21/37)	0.00000(0/16)	0.00000(0/17)	0.05556(1/18)
Bo_TE_64039	0.53488(23/43)	0.13333(2/15)	0.00000(0/23)	0.10000(2/20)
Bo_TE_74944	0.00000(0/46)	0.57895(11/19)	0.00000(0/23)	0.00000(0/20)
Bo_TE_121656	0.26667(12/45)	0.05882(1/17)	0.04545(1/22)	0.66667(12/18)
Bo_TE_71282	0.11111(5/45)	0.94118(16/17)	0.68421(13/19)	0.68750(11/16)
Bo_TE_149527	0.84615(33/39)	0.29412(5/17)	0.00000(0/23)	0.00000(0/20)
Bo_TE_31548	0.86364(38/44)	0.87500(14/16)	1.00000(22/22)	0.27778(5/18)
Bo_TE_1138	0.54545(24/44)	0.29412(5/17)	0.23810(5/21)	0.94444(17/18)
Bo_TE_92929	0.34091(15/44)	0.17647(3/17)	0.94737(18/19)	0.85000(17/20)
Bo_TE_159304	0.10256(4/39)	0.61111(11/18)	0.42105(8/19)	0.76471(13/17)
Bo_TE_209912	0.02326(1/43)	0.00000(0/19)	0.20000(4/20)	0.75000(15/20)
Bo_TE_41977	0.09302(4/43)	0.27778(5/18)	0.65000(13/20)	0.00000(0/20)
Bo_TE_145497	0.00000(0/45)	0.58824(10/17)	0.00000(0/21)	0.00000(0/20)
Bo_TE_179656	0.71429(30/42)	0.25000(4/16)	0.27778(5/18)	0.00000(0/20)
Bo_TE_138427	0.16667(7/42)	0.05556(1/18)	0.13636(3/22)	0.73684(14/19)
Bo_TE_81939	0.89744(35/39)	1.00000(15/15)	0.76190(16/21)	0.11765(2/17)
Bo_TE_50602	0.41026(16/39)	0.05263(1/19)	0.08696(2/23)	0.80000(16/20)
Bo_TE_211216	0.00000(0/45)	0.00000(0/17)	0.00000(0/22)	0.55000(11/20)
Bo_TE_36914	0.04348(2/46)	0.68750(11/16)	0.72727(8/11)	0.00000(0/15)
Bo_TE_108038	0.00000(0/46)	0.89474(17/19)	0.00000(0/23)	0.00000(0/20)

Bo_TE_93910	0.00000(0/43)	0.52941(9/17)	0.00000(0/23)	0.00000(0/20)
Bo_TE_79666	0.21429(9/42)	0.76471(13/17)	0.57143(8/14)	0.65000(13/20)
Bo_TE_48413	0.23256(10/43)	0.05556(1/18)	0.50000(10/20)	0.00000(0/20)
Bo_TE_92632	0.90476(38/42)	0.11765(2/17)	0.45455(10/22)	0.73684(14/19)
Bo_TE_44453	0.91111(41/45)	1.00000(18/18)	0.95455(21/22)	0.44444(8/18)
Bo_TE_108113	0.95652(44/46)	0.23529(4/17)	0.82609(19/23)	1.00000(19/19)
Bo_TE_13946	0.46341(19/41)	1.00000(17/17)	0.77273(17/22)	0.84211(16/19)
Bo_TE_48996	0.16667(7/42)	0.75000(12/16)	0.90000(18/20)	0.65000(13/20)
Bo_TE_60296	0.42857(18/42)	0.16667(3/18)	0.73913(17/23)	0.21053(4/19)
Bo_TE_150628	0.67442(29/43)	0.33333(6/18)	0.04348(1/23)	0.42105(8/19)
Bo_TE_141532	0.18182(8/44)	0.94444(17/18)	0.95455(21/22)	0.78947(15/19)
Bo_TE_198589	0.11364(5/44)	0.21053(4/19)	0.38095(8/21)	0.94737(18/19)
Bo_TE_183457	0.55000(22/40)	0.94444(17/18)	0.80000(16/20)	0.36842(7/19)
Bo_TE_37462	1.00000(36/36)	0.38889(7/18)	1.00000(19/19)	0.94118(16/17)
Bo_TE_53143	0.46512(20/43)	0.25000(4/16)	0.50000(11/22)	1.00000(19/19)
Bo_TE_240721	0.73684(28/38)	0.15789(3/19)	0.00000(0/23)	0.05263(1/19)
Bo_TE_180758	0.53488(23/43)	0.64706(11/17)	0.00000(0/22)	0.00000(0/19)
Bo_TE_123743	0.02174(1/46)	0.05556(1/18)	0.50000(11/22)	0.00000(0/20)
Bo_TE_5298	0.00000(0/45)	0.31250(5/16)	0.52381(11/21)	0.25000(5/20)
Bo_TE_215870	0.32558(14/43)	0.58824(10/17)	0.00000(0/22)	0.00000(0/20)
Bo_TE_165058	0.95556(43/45)	0.50000(8/16)	0.95455(21/22)	1.00000(20/20)
Bo_TE_14065	0.94595(35/37)	1.00000(17/17)	0.63636(14/22)	0.26316(5/19)
Bo_TE_42782	0.04651(2/43)	0.22222(4/18)	0.22727(5/22)	0.55556(10/18)
Bo_TE_232706	0.97778(44/45)	1.00000(19/19)	0.61905(13/21)	0.26316(5/19)
Bo_TE_231564	1.00000(45/45)	0.64706(11/17)	0.25000(5/20)	0.45000(9/20)
Bo_TE_202843	0.88636(39/44)	0.66667(12/18)	0.00000(0/22)	0.05263(1/19)
Bo_TE_122873	0.61905(26/42)	0.23529(4/17)	1.00000(20/20)	0.65000(13/20)
Bo_TE_159859	0.00000(0/33)	0.00000(0/16)	0.60000(12/20)	0.16667(3/18)
Bo_TE_156690	0.46341(19/41)	0.68750(11/16)	0.04348(1/23)	0.36842(7/19)
Bo_TE_178793	0.46667(21/45)	0.16667(3/18)	0.65217(15/23)	0.88889(16/18)
Bo_TE_81648	0.02222(1/45)	0.81250(13/16)	0.00000(0/22)	0.00000(0/20)
Bo_TE_82141	0.21429(9/42)	0.35294(6/17)	0.59091(13/22)	1.00000(18/18)
Bo_TE_25827	0.67500(27/40)	0.06250(1/16)	0.00000(0/20)	0.00000(0/18)
Bo_TE_220105	0.27273(9/33)	0.68750(11/16)	0.05882(1/17)	0.06250(1/16)
Bo_TE_88362	0.11905(5/42)	0.75000(12/16)	0.08696(2/23)	0.05000(1/20)
Bo_TE_56775	0.94872(37/39)	0.61111(11/18)	0.27778(5/18)	0.11765(2/17)
Bo_TE_163565	0.79070(34/43)	1.00000(17/17)	0.80952(17/21)	0.36842(7/19)
Bo_TE_40002	0.33333(14/42)	0.55556(10/18)	0.63636(14/22)	0.88889(16/18)
Bo_TE_197259	0.00000(0/44)	0.11111(2/18)	0.00000(0/23)	0.52632(10/19)
Bo_TE_42864	0.67442(29/43)	0.47059(8/17)	0.13636(3/22)	0.16667(3/18)
Bo_TE_34059	0.56098(23/41)	0.11111(2/18)	0.00000(0/21)	0.10000(2/20)
Bo_TE_53972	0.21212(7/33)	0.10526(2/19)	0.55000(11/20)	0.93750(15/16)
Bo_TE_29621	0.50000(20/40)	1.00000(17/17)	1.00000(18/18)	1.00000(19/19)
Bo_TE_131313	1.00000(43/43)	1.00000(18/18)	1.00000(23/23)	0.44444(8/18)
Bo_TE_159546	0.48780(20/41)	0.12500(2/16)	0.95455(21/22)	0.50000(9/18)
Bo_TE_234805	0.88636(39/44)	0.16667(3/18)	0.00000(0/23)	0.10000(2/20)
Bo_TE_151660	0.93182(41/44)	0.88235(15/17)	0.50000(11/22)	0.29412(5/17)
Bo_TE_173291	0.19048(8/42)	0.00000(0/18)	0.86364(19/22)	0.00000(0/20)
Bo_TE_192029	0.57143(24/42)	0.16667(3/18)	0.00000(0/22)	0.00000(0/19)
Bo_TE_213016	0.00000(0/27)	0.00000(0/19)	0.59091(13/22)	0.37500(6/16)
Bo_TE_41878	0.77273(34/44)	0.52941(9/17)	0.09524(2/21)	0.17647(3/17)
Bo_TE_190915	0.47619(20/42)	0.58824(10/17)	0.04348(1/23)	0.00000(0/19)
Bo_TE_139805	1.00000(43/43)	0.50000(9/18)	0.68421(13/19)	0.57895(11/19)
Bo_TE_237216	0.81818(36/44)	0.11111(2/18)	0.00000(0/22)	0.10526(2/19)
Bo_TE_208068	0.89130(41/46)	0.47059(8/17)	1.00000(23/23)	0.66667(12/18)
Bo_TE_129792	0.00000(0/43)	0.62500(10/16)	0.09091(2/22)	0.05000(1/20)

Bo_TE_223461	0.00000(0/46)	0.11765(2/17)	0.75000(15/20)	0.50000(10/20)
Bo_TE_41158	0.02381(1/42)	0.00000(0/18)	0.00000(0/21)	0.68750(11/16)
Bo_TE_221277	0.00000(0/44)	0.81250(13/16)	0.04762(1/21)	0.05263(1/19)
Bo_TE_168777	0.50000(20/40)	0.05556(1/18)	0.00000(0/23)	0.21053(4/19)
Bo_TE_15808	0.14286(6/42)	0.25000(4/16)	0.33333(6/18)	0.84211(16/19)
Bo_TE_101188	0.41860(18/43)	0.31250(5/16)	0.85000(17/20)	0.17647(3/17)
Bo_TE_63966	0.52381(22/42)	0.05556(1/18)	0.00000(0/22)	0.10000(2/20)
Bo_TE_161932	0.52500(21/40)	0.33333(6/18)	0.00000(0/23)	0.15000(3/20)
Bo_TE_226830	0.02222(1/45)	0.13333(2/15)	0.14286(3/21)	0.52632(10/19)
Bo_TE_144326	0.40909(18/44)	0.00000(0/17)	0.52381(11/21)	0.38889(7/18)
Bo_TE_235577	0.52500(21/40)	0.41176(7/17)	0.94737(18/19)	0.84211(16/19)
Bo_TE_144750	0.13953(6/43)	0.82353(14/17)	0.77778(14/18)	0.05882(1/17)
Bo_TE_215188	0.08889(4/45)	0.73333(11/15)	0.81818(18/22)	0.52632(10/19)
Bo_TE_117061	0.26190(11/42)	0.58824(10/17)	0.00000(0/23)	0.05000(1/20)
Bo_TE_48842	0.06667(3/45)	0.33333(6/18)	0.13636(3/22)	0.57895(11/19)
Bo_TE_56008	0.15385(6/39)	0.57895(11/19)	0.00000(0/23)	0.10000(2/20)
Bo_TE_124107	0.52381(22/42)	0.06250(1/16)	0.84211(16/19)	0.72222(13/18)
Bo_TE_68795	0.13953(6/43)	0.44444(8/18)	0.47368(9/19)	0.68421(13/19)
Bo_TE_17719	0.04545(2/44)	0.00000(0/16)	0.81818(18/22)	0.36842(7/19)
Bo_TE_93724	0.04545(2/44)	0.50000(9/18)	0.04545(1/22)	0.00000(0/20)
Bo_TE_220606	0.30769(12/39)	0.52632(10/19)	0.52632(10/19)	1.00000(19/19)
Bo_TE_89540	0.61364(27/44)	0.70588(12/17)	0.33333(7/21)	0.05263(1/19)
Bo_TE_177249	0.13636(6/44)	1.00000(18/18)	0.91304(21/23)	0.15789(3/19)
Bo_TE_154639	0.69231(27/39)	0.66667(10/15)	0.19048(4/21)	0.66667(12/18)
Bo_TE_210583	0.14286(6/42)	0.05263(1/19)	0.54545(12/22)	0.70000(14/20)
Bo_TE_213791	0.13636(6/44)	0.57895(11/19)	0.08696(2/23)	0.05000(1/20)
Bo_TE_223502	1.00000(45/45)	0.40000(6/15)	0.28571(6/21)	0.21053(4/19)
Bo_TE_121744	0.00000(0/43)	0.00000(0/19)	0.04762(1/21)	0.70000(14/20)
Bo_TE_66862	0.00000(0/42)	0.00000(0/18)	0.38889(7/18)	0.73684(14/19)
Bo_TE_229875	0.08696(4/46)	0.62500(10/16)	0.09524(2/21)	0.05000(1/20)
Bo_TE_153824	0.77778(21/27)	0.88889(16/18)	0.33333(7/21)	0.05882(1/17)
Bo_TE_92838	0.68889(31/45)	0.88889(16/18)	0.86364(19/22)	0.15000(3/20)
Bo_TE_52707	0.41463(17/41)	0.88235(15/17)	0.95652(22/23)	0.94737(18/19)
Bo_TE_61761	0.70455(31/44)	0.68750(11/16)	0.69565(16/23)	0.15000(3/20)
Bo_TE_185386	0.97778(44/45)	0.41176(7/17)	0.52381(11/21)	0.75000(15/20)
Bo_TE_143742	0.20000(9/45)	0.88235(15/17)	0.76190(16/21)	0.85000(17/20)
Bo_TE_152179	0.67442(29/43)	0.11765(2/17)	0.13636(3/22)	0.00000(0/18)
Bo_TE_144927	0.86667(39/45)	0.23529(4/17)	0.00000(0/22)	0.52941(9/17)
Bo_TE_154365	0.13953(6/43)	0.75000(12/16)	0.10526(2/19)	0.00000(0/20)
Bo_TE_130446	0.75000(33/44)	0.88889(16/18)	0.04545(1/22)	0.75000(15/20)
Bo_TE_235360	0.59524(25/42)	0.41176(7/17)	1.00000(23/23)	0.70000(14/20)
Bo_TE_58070	0.39535(17/43)	0.17647(3/17)	0.78261(18/23)	0.80000(16/20)
Bo_TE_9798	0.86957(40/46)	0.31250(5/16)	0.05263(1/19)	0.36842(7/19)
Bo_TE_200042	0.11111(5/45)	0.56250(9/16)	0.72727(16/22)	0.84211(16/19)
Bo_TE_99330	0.91111(41/45)	1.00000(18/18)	0.42105(8/19)	0.68421(13/19)
Bo_TE_145979	0.79070(34/43)	0.18750(3/16)	0.21739(5/23)	0.00000(0/19)
Bo_TE_21244	0.06977(3/43)	0.00000(0/16)	0.61905(13/21)	0.33333(6/18)
Bo_TE_140013	0.00000(0/45)	0.36842(7/19)	0.59091(13/22)	0.45000(9/20)
Bo_TE_224842	0.62069(18/29)	0.06667(1/15)	0.15789(3/19)	0.84211(16/19)
Bo_TE_52717	0.79545(35/44)	0.94118(16/17)	0.18182(4/22)	0.15789(3/19)
Bo_TE_207173	0.39535(17/43)	0.61111(11/18)	0.63158(12/19)	0.11111(2/18)
Bo_TE_91732	0.74419(32/43)	0.50000(8/16)	1.00000(20/20)	0.89474(17/19)
Bo_TE_209512	0.90698(39/43)	0.11765(2/17)	0.91304(21/23)	0.72222(13/18)
Bo_TE_239536	0.03030(1/33)	0.17647(3/17)	0.90000(18/20)	0.12500(2/16)
Bo_TE_172099	0.70732(29/41)	0.12500(2/16)	0.80952(17/21)	0.52632(10/19)
Bo_TE_63129	0.02326(1/43)	0.76471(13/17)	0.66667(14/21)	0.76471(13/17)

Bo_TE_235668	0.19048(8/42)	0.10526(2/19)	0.90909(20/22)	0.44444(8/18)
Bo_TE_15039	1.00000(44/44)	0.25000(4/16)	0.36842(7/19)	0.11111(2/18)
Bo_TE_63683	0.65116(28/43)	0.43750(7/16)	0.00000(0/22)	0.26316(5/19)
Bo_TE_18025	0.02174(1/46)	0.00000(0/17)	0.50000(11/22)	0.05000(1/20)
Bo_TE_158928	0.61538(24/39)	0.00000(0/15)	0.09524(2/21)	0.47368(9/19)
Bo_TE_58875	0.13333(6/45)	0.61111(11/18)	0.00000(0/21)	0.00000(0/20)
Bo_TE_5519	0.80000(36/45)	0.20000(3/15)	0.19048(4/21)	0.15789(3/19)
Bo_TE_235193	0.36364(16/44)	0.31250(5/16)	0.75000(15/20)	0.83333(15/18)
Bo_TE_153790	0.23810(10/42)	0.88235(15/17)	0.04348(1/23)	0.10526(2/19)
Bo_TE_44222	0.02222(1/45)	0.00000(0/19)	0.43478(10/23)	0.73684(14/19)
Bo_TE_197255	1.00000(45/45)	0.88235(15/17)	1.00000(23/23)	0.47368(9/19)
Bo_TE_138930	0.81818(36/44)	0.94118(16/17)	1.00000(21/21)	0.35000(7/20)
Bo_TE_141536	0.78378(29/37)	0.05556(1/18)	0.08696(2/23)	0.17647(3/17)
Bo_TE_182371	0.93478(43/46)	0.43750(7/16)	0.38095(8/21)	0.61111(11/18)
Bo_TE_215128	0.00000(0/45)	0.56250(9/16)	0.00000(0/22)	0.00000(0/20)
Bo_TE_152405	0.77500(31/40)	0.10526(2/19)	0.42857(9/21)	0.10000(2/20)
Bo_TE_56194	0.48718(19/39)	0.61111(11/18)	0.60000(12/20)	1.00000(20/20)
Bo_TE_146037	0.38095(16/42)	0.75000(12/16)	0.00000(0/22)	0.00000(0/20)
Bo_TE_2337	0.57143(24/42)	0.40000(6/15)	0.09524(2/21)	0.05263(1/19)
Bo_TE_94435	1.00000(43/43)	0.88235(15/17)	0.55000(11/20)	0.47368(9/19)
Bo_TE_94014	0.76744(33/43)	0.68421(13/19)	0.23810(5/21)	0.35294(6/17)
Bo_TE_114558	0.17778(8/45)	0.88235(15/17)	0.52381(11/21)	0.90000(18/20)
Bo_TE_169498	0.58974(23/39)	0.82353(14/17)	0.00000(0/21)	0.00000(0/19)
Bo_TE_162543	0.80000(36/45)	0.94737(18/19)	0.57143(12/21)	0.10526(2/19)
Bo_TE_66362	0.02273(1/44)	0.05556(1/18)	0.42857(9/21)	0.64706(11/17)
Bo_TE_215947	0.55814(24/43)	0.00000(0/18)	0.00000(0/22)	0.05000(1/20)
Bo_TE_170053	0.00000(0/45)	0.56250(9/16)	0.38095(8/21)	0.15000(3/20)
Bo_TE_230822	0.95652(44/46)	0.52941(9/17)	0.50000(10/20)	0.31579(6/19)
Bo_TE_43830	0.16279(7/43)	0.76471(13/17)	0.72727(16/22)	0.78947(15/19)
Bo_TE_158853	0.57143(24/42)	0.26667(4/15)	0.33333(7/21)	0.05000(1/20)
Bo_TE_215511	0.79545(35/44)	0.47059(8/17)	0.47619(10/21)	0.05000(1/20)
Bo_TE_177874	0.04651(2/43)	0.00000(0/18)	0.04348(1/23)	0.68421(13/19)
Bo_TE_109364	0.11364(5/44)	0.87500(14/16)	0.19048(4/21)	0.57895(11/19)
Bo_TE_133094	0.00000(0/46)	0.00000(0/18)	0.76190(16/21)	0.16667(3/18)
Bo_TE_97110	0.55000(22/40)	0.00000(0/19)	0.00000(0/22)	0.15000(3/20)
Bo_TE_69318	0.72093(31/43)	0.50000(8/16)	0.17391(4/23)	0.85000(17/20)
Bo_TE_156636	0.00000(0/41)	0.75000(12/16)	0.04762(1/21)	0.15789(3/19)
Bo_TE_172581	0.00000(0/46)	0.62500(10/16)	0.00000(0/21)	0.00000(0/20)
Bo_TE_95249	0.14634(6/41)	0.58824(10/17)	0.00000(0/23)	0.00000(0/18)
Bo_TE_194449	0.68421(26/38)	0.88889(16/18)	0.84211(16/19)	0.00000(0/20)
Bo_TE_223733	0.74359(29/39)	1.00000(16/16)	0.47619(10/21)	0.85000(17/20)
Bo_TE_47907	0.51220(21/41)	0.11111(2/18)	1.00000(22/22)	0.78947(15/19)
Bo_TE_39588	0.95349(41/43)	1.00000(19/19)	0.50000(11/22)	0.75000(6/8)
Bo_TE_18151	0.85714(36/42)	0.83333(15/18)	0.00000(0/21)	0.80000(16/20)
Bo_TE_190165	0.36585(15/41)	0.15385(2/13)	0.52941(9/17)	1.00000(18/18)
Bo_TE_182378	0.93182(41/44)	0.42105(8/19)	0.40000(8/20)	0.55000(11/20)
Bo_TE_86838	0.88636(39/44)	0.25000(4/16)	0.23810(5/21)	0.36842(7/19)
Bo_TE_178042	0.88636(39/44)	0.05556(1/18)	0.19048(4/21)	0.45000(9/20)
Bo_TE_225192	0.61905(26/42)	0.27778(5/18)	0.95455(21/22)	0.61111(11/18)
Bo_TE_129400	0.00000(0/45)	0.00000(0/19)	0.68421(13/19)	0.05000(1/20)
Bo_TE_47886	0.43590(17/39)	0.75000(12/16)	0.00000(0/23)	0.11111(2/18)
Bo_TE_61731	0.32500(13/40)	0.33333(5/15)	0.68182(15/22)	0.84211(16/19)
Bo_TE_193657	0.34091(15/44)	0.58824(10/17)	0.15789(3/19)	0.05000(1/20)
Bo_TE_108449	0.60000(27/45)	0.22222(4/18)	1.00000(23/23)	0.95000(19/20)
Bo_TE_156996	0.63415(26/41)	0.94118(16/17)	0.31818(7/22)	0.70000(14/20)
Bo_TE_105285	0.38095(16/42)	0.11111(2/18)	0.82609(19/23)	0.50000(10/20)

Bo_TE_197869	1.00000(46/46)	0.47368(9/19)	1.00000(20/20)	0.78947(15/19)
Bo_TE_91654	0.95455(42/44)	0.66667(12/18)	0.36842(7/19)	0.85000(17/20)
Bo_TE_81993	1.00000(46/46)	1.00000(18/18)	0.30000(6/20)	0.77778(14/18)
Bo_TE_35853	0.78378(29/37)	0.88235(15/17)	1.00000(16/16)	0.35000(7/20)
Bo_TE_141345	0.14634(6/41)	0.91667(11/12)	0.95455(21/22)	0.72222(13/18)
Bo_TE_71152	0.82500(33/40)	0.41176(7/17)	0.09091(2/22)	0.21053(4/19)
Bo_TE_224498	0.06522(3/46)	0.64706(11/17)	0.00000(0/22)	0.00000(0/20)
Bo_TE_123504	0.30952(13/42)	0.29412(5/17)	0.80000(16/20)	0.70000(14/20)
Bo_TE_56344	0.21429(9/42)	0.41176(7/17)	0.35000(7/20)	0.85000(17/20)
Bo_TE_81749	0.00000(0/41)	0.36842(7/19)	0.33333(7/21)	0.58824(10/17)
Bo_TE_13507	0.92683(38/41)	0.88235(15/17)	0.00000(0/18)	0.22222(4/18)
Bo_TE_28926	0.77273(34/44)	0.93750(15/16)	0.91304(21/23)	0.36842(7/19)
Bo_TE_19877	0.52381(22/42)	0.76471(13/17)	0.00000(0/23)	0.22222(4/18)
Bo_TE_104986	0.32609(15/46)	1.00000(17/17)	0.66667(14/21)	0.40000(8/20)
Bo_TE_58678	0.46341(19/41)	0.05263(1/19)	0.82609(19/23)	0.00000(0/20)
Bo_TE_204011	0.71429(30/42)	0.18750(3/16)	0.76190(16/21)	0.20000(4/20)
Bo_TE_129648	0.53488(23/43)	0.00000(0/18)	0.00000(0/20)	0.15789(3/19)
Bo_TE_55736	0.04545(2/44)	0.00000(0/19)	0.08696(2/23)	0.80000(16/20)
Bo_TE_81507	0.10256(4/39)	0.70588(12/17)	0.09524(2/21)	0.21429(3/14)
Bo_TE_14636	0.73171(30/41)	0.64706(11/17)	0.20000(4/20)	0.05556(1/18)
Bo_TE_169218	0.06977(3/43)	0.81250(13/16)	0.72727(8/11)	0.18750(3/16)
Bo_TE_134982	0.70732(29/41)	0.35294(6/17)	1.00000(22/22)	0.94737(18/19)
Bo_TE_7009	0.02439(1/41)	0.68750(11/16)	0.33333(7/21)	0.26316(5/19)
Bo_TE_13905	0.73810(31/42)	0.46667(7/15)	0.40909(9/22)	0.00000(0/20)
Bo_TE_79116	0.52632(20/38)	0.22222(4/18)	0.00000(0/22)	0.25000(5/20)
Bo_TE_144612	0.84444(38/45)	0.42105(8/19)	0.00000(0/22)	0.80000(16/20)
Bo_TE_149385	0.06818(3/44)	0.61111(11/18)	0.00000(0/23)	0.47368(9/19)
Bo_TE_91240	0.18182(8/44)	0.00000(0/17)	0.15789(3/19)	0.73684(14/19)
Bo_TE_232743	1.00000(42/42)	1.00000(19/19)	0.63636(14/22)	0.35000(7/20)
Bo_TE_18429	0.04651(2/43)	0.00000(0/18)	0.00000(0/23)	0.55556(10/18)
Bo_TE_101430	0.04651(2/43)	0.00000(0/17)	0.00000(0/23)	0.55556(10/18)
Bo_TE_27468	0.69767(30/43)	0.00000(0/18)	0.00000(0/23)	0.00000(0/20)
Bo_TE_230123	0.67442(29/43)	0.64286(9/14)	0.19048(4/21)	0.15000(3/20)
Bo_TE_67798	0.09091(4/44)	0.43750(7/16)	0.31818(7/22)	0.80000(16/20)
Bo_TE_76440	0.18605(8/43)	0.61111(11/18)	0.00000(0/21)	0.30000(6/20)
Bo_TE_219046	0.19512(8/41)	0.94118(16/17)	0.72222(13/18)	0.77778(14/18)
Bo_TE_139329	0.21951(9/41)	0.05263(1/19)	0.00000(0/23)	0.52632(10/19)
Bo_TE_115545	0.25000(11/44)	0.05882(1/17)	0.04762(1/21)	0.64706(11/17)
Bo_TE_129247	0.82051(32/39)	0.82353(14/17)	0.28571(6/21)	0.26316(5/19)
Bo_TE_124381	0.06977(3/43)	0.56250(9/16)	0.00000(0/23)	0.05000(1/20)
Bo_TE_25626	0.23256(10/43)	0.66667(12/18)	0.20000(4/20)	0.72222(13/18)
Bo_TE_237505	0.46512(20/43)	0.57143(8/14)	1.00000(23/23)	1.00000(20/20)
Bo_TE_230135	0.77778(35/45)	0.00000(0/16)	0.45455(10/22)	0.42105(8/19)
Bo_TE_21911	0.29545(13/44)	0.11111(2/18)	0.47619(10/21)	1.00000(20/20)
Bo_TE_58247	0.53659(22/41)	0.89474(17/19)	0.22727(5/22)	0.52941(9/17)
Bo_TE_89476	0.72500(29/40)	0.68750(11/16)	0.09091(2/22)	0.05263(1/19)
Bo_TE_234996	0.50000(21/42)	0.76471(13/17)	1.00000(23/23)	0.66667(12/18)
Bo_TE_190435	0.02222(1/45)	0.15789(3/19)	0.55000(11/20)	0.00000(0/20)
Bo_TE_231515	0.06522(3/46)	0.60000(9/15)	0.56522(13/23)	0.60000(12/20)
Bo_TE_197601	0.00000(0/45)	0.10526(2/19)	0.00000(0/19)	0.50000(8/16)
Bo_TE_6990	0.78049(32/41)	0.17647(3/17)	0.19048(4/21)	0.00000(0/20)
Bo_TE_75951	0.97561(40/41)	0.47059(8/17)	0.85714(18/21)	0.55000(11/20)
Bo_TE_105706	0.02326(1/43)	0.00000(0/19)	0.33333(7/21)	0.75000(15/20)
Bo_TE_19625	0.00000(0/46)	0.00000(0/18)	0.40909(9/22)	0.50000(10/20)
Bo_TE_153713	0.00000(0/41)	0.87500(14/16)	0.50000(10/20)	0.56250(9/16)
Bo_TE_2519	0.29545(13/44)	0.64706(11/17)	0.00000(0/22)	0.15000(3/20)

Bo_TE_56731	0.00000(0/45)	0.00000(0/18)	0.50000(10/20)	0.15789(3/19)
Bo_TE_167775	0.06667(3/45)	0.16667(3/18)	0.65000(13/20)	0.00000(0/19)
Bo_TE_78938	0.11628(5/43)	0.29412(5/17)	0.66667(14/21)	0.94737(18/19)
Bo_TE_23382	0.72093(31/43)	0.18750(3/16)	0.09091(2/22)	0.47368(9/19)
Bo_TE_229862	0.50000(23/46)	0.05556(1/18)	0.40909(9/22)	0.85000(17/20)
Bo_TE_133284	0.00000(0/45)	0.77778(14/18)	0.00000(0/23)	0.10526(2/19)
Bo_TE_28416	0.59524(25/42)	0.35294(6/17)	1.00000(23/23)	0.83333(15/18)
Bo_TE_93251	0.58537(24/41)	0.17647(3/17)	0.95455(21/22)	0.94444(17/18)
Bo_TE_28353	0.02273(1/44)	0.22222(4/18)	0.66667(14/21)	0.44444(8/18)
Bo_TE_74236	0.09756(4/41)	0.11111(2/18)	0.28571(6/21)	0.68421(13/19)
Bo_TE_37360	0.85714(36/42)	0.10526(2/19)	0.00000(0/21)	0.05263(1/19)
Bo_TE_168303	0.85000(34/40)	0.47368(9/19)	1.00000(20/20)	1.00000(18/18)
Bo_TE_150802	0.97436(38/39)	0.17647(3/17)	0.13636(3/22)	0.35000(7/20)
Bo_TE_81570	0.59524(25/42)	0.17647(3/17)	0.34783(8/23)	0.75000(15/20)
Bo_TE_144818	0.15556(7/45)	0.88235(15/17)	1.00000(22/22)	0.35000(7/20)
Bo_TE_96323	0.97778(44/45)	1.00000(17/17)	0.61905(13/21)	0.16667(3/18)
Bo_TE_10873	0.06667(3/45)	0.29412(5/17)	0.08696(2/23)	0.85000(17/20)
Bo_TE_164959	1.00000(44/44)	1.00000(18/18)	0.45455(10/22)	0.26316(5/19)
Bo_TE_141396	0.83721(36/43)	0.06250(1/16)	0.04762(1/21)	0.22222(4/18)
Bo_TE_23892	0.11364(5/44)	0.75000(12/16)	0.13043(3/23)	0.21053(4/19)
Bo_TE_88348	0.47368(18/38)	0.88235(15/17)	0.91304(21/23)	1.00000(20/20)
Bo_TE_63652	0.41667(15/36)	0.76471(13/17)	0.00000(0/19)	0.11111(1/9)
Bo_TE_79996	0.95455(42/44)	1.00000(17/17)	0.90476(19/21)	0.27778(5/18)
Bo_TE_97175	0.47619(20/42)	0.61111(11/18)	0.18182(4/22)	0.10526(2/19)
Bo_TE_174635	0.97826(45/46)	0.81250(13/16)	0.45000(9/20)	0.95000(19/20)
Bo_TE_240184	0.02500(1/40)	0.52941(9/17)	0.04762(1/21)	0.26316(5/19)
Bo_TE_221206	0.81579(31/38)	0.17647(3/17)	0.95455(21/22)	0.94737(18/19)
Bo_TE_22966	0.00000(0/45)	0.00000(0/18)	0.38095(8/21)	0.73684(14/19)
Bo_TE_28109	0.35556(16/45)	0.00000(0/17)	0.04545(1/22)	0.63158(12/19)
Bo_TE_197252	0.00000(0/45)	0.10526(2/19)	0.00000(0/23)	0.52941(9/17)
Bo_TE_42962	0.95455(42/44)	0.27778(5/18)	0.81818(18/22)	0.94444(17/18)
Bo_TE_57324	1.00000(44/44)	1.00000(19/19)	0.77273(17/22)	0.45000(9/20)
Bo_TE_155096	0.83721(36/43)	0.88889(16/18)	0.69565(16/23)	0.30000(6/20)
Bo_TE_145599	0.09756(4/41)	0.57895(11/19)	0.26316(5/19)	0.66667(12/18)
Bo_TE_212957	0.65909(29/44)	0.00000(0/15)	0.09524(2/21)	0.11111(2/18)
Bo_TE_86787	0.78049(32/41)	0.06250(1/16)	0.17391(4/23)	0.57895(11/19)
Bo_TE_4321	0.09302(4/43)	0.22222(4/18)	0.04762(1/21)	0.75000(12/16)
Bo_TE_197994	0.97619(41/42)	0.52941(9/17)	0.20000(4/20)	0.44444(8/18)
Bo_TE_189783	0.02381(1/42)	0.15789(3/19)	0.54545(12/22)	0.00000(0/19)
Bo_TE_188240	0.90909(40/44)	0.35294(6/17)	0.14286(3/21)	0.38889(7/18)
Bo_TE_211904	0.04545(2/44)	0.06250(1/16)	0.21053(4/19)	0.65000(13/20)
Bo_TE_44870	0.28125(9/32)	0.11111(2/18)	0.00000(0/19)	0.88235(15/17)
Bo_TE_3657	0.54762(23/42)	0.75000(12/16)	0.80000(16/20)	0.00000(0/20)
Bo_TE_173145	0.00000(0/40)	0.05556(1/18)	0.52381(11/21)	0.16667(3/18)
Bo_TE_163852	0.02222(1/45)	0.20000(3/15)	0.35000(7/20)	0.89474(17/19)
Bo_TE_179384	0.77273(34/44)	0.31250(5/16)	0.00000(0/21)	0.05000(1/20)
Bo_TE_33090	0.76190(32/42)	0.33333(6/18)	0.38095(8/21)	0.83333(15/18)
Bo_TE_144832	0.11905(5/42)	0.88235(15/17)	1.00000(22/22)	0.33333(6/18)
Bo_TE_42597	0.22727(10/44)	0.20000(3/15)	0.65000(13/20)	0.84211(16/19)
Bo_TE_222133	0.02273(1/44)	0.00000(0/19)	0.00000(0/23)	0.64706(11/17)
Bo_TE_190831	0.34091(15/44)	0.87500(14/16)	0.76190(16/21)	0.90000(18/20)
Bo_TE_55016	0.88372(38/43)	0.50000(9/18)	0.13636(3/22)	0.55000(11/20)
Bo_TE_77693	0.00000(0/45)	0.56250(9/16)	0.36364(8/22)	0.00000(0/20)
Bo_TE_12980	0.13636(6/44)	0.94118(16/17)	0.78261(18/23)	0.68421(13/19)
Bo_TE_89009	0.22500(9/40)	0.91667(11/12)	0.69231(9/13)	1.00000(17/17)
Bo_TE_112774	0.25000(11/44)	0.76471(13/17)	0.59091(13/22)	0.55000(11/20)

Bo_TE_125595	0.61905(26/42)	0.70588(12/17)	0.04545(1/22)	0.00000(0/20)
Bo_TE_180472	0.56818(25/44)	0.35294(6/17)	1.00000(23/23)	0.90000(18/20)
Bo_TE_129781	0.47368(18/38)	0.77778(14/18)	0.00000(0/23)	0.05263(1/19)
Bo_TE_235639	0.72093(31/43)	0.11765(2/17)	1.00000(23/23)	0.65000(13/20)
Bo_TE_4633	0.16667(7/42)	0.76471(13/17)	0.66667(14/21)	0.43750(7/16)
Bo_TE_55619	0.56098(23/41)	0.83333(15/18)	0.65000(13/20)	0.26316(5/19)
Bo_TE_28324	0.00000(0/45)	0.00000(0/19)	0.65000(13/20)	0.00000(0/20)
Bo_TE_133796	0.92857(39/42)	0.16667(3/18)	1.00000(22/22)	0.80000(16/20)
Bo_TE_69138	0.45455(20/44)	0.00000(0/19)	0.55000(11/20)	0.00000(0/19)
Bo_TE_76460	0.83333(35/42)	0.12500(2/16)	1.00000(20/20)	0.70000(14/20)
Bo_TE_127172	0.06977(3/43)	0.18182(2/11)	0.63636(14/22)	0.65000(13/20)
Bo_TE_180605	0.08889(4/45)	0.00000(0/19)	0.04762(1/21)	0.55000(11/20)
Bo_TE_91082	0.82927(34/41)	0.29412(5/17)	0.90476(19/21)	0.84211(16/19)
Bo_TE_33616	0.28571(12/42)	0.00000(0/17)	0.23810(5/21)	0.72222(13/18)
Bo_TE_169885	0.60465(26/43)	0.22222(4/18)	0.00000(0/23)	0.05000(1/20)
Bo_TE_118291	0.02273(1/44)	0.05882(1/17)	1.00000(18/18)	0.85000(17/20)
Bo_TE_14661	0.17500(7/40)	0.75000(6/8)	0.94737(18/19)	1.00000(18/18)
Bo_TE_59126	0.00000(0/43)	0.64706(11/17)	0.61905(13/21)	0.83333(15/18)
Bo_TE_197614	0.97674(42/43)	0.89474(17/19)	1.00000(23/23)	0.44444(8/18)
Bo_TE_223479	0.02222(1/45)	0.00000(0/17)	0.73684(14/19)	0.73684(14/19)
Bo_TE_83060	0.86842(33/38)	0.46667(7/15)	0.15000(3/20)	0.80000(12/15)
Bo_TE_25046	0.04651(2/43)	0.35294(6/17)	0.61905(13/21)	0.15000(3/20)
Bo_TE_113079	0.76190(32/42)	0.25000(4/16)	0.47619(10/21)	0.52632(10/19)
Bo_TE_123396	0.00000(0/45)	0.47368(9/19)	0.78947(15/19)	0.57895(11/19)
Bo_TE_142274	0.72500(29/40)	0.57895(11/19)	0.00000(0/22)	0.00000(0/20)
Bo_TE_60175	0.06977(3/43)	0.05882(1/17)	0.38889(7/18)	0.88889(16/18)
Bo_TE_65546	0.19512(8/41)	0.21053(4/19)	0.00000(0/22)	0.65000(13/20)
Bo_TE_129185	0.23810(10/42)	0.82353(14/17)	0.08696(2/23)	0.10000(2/20)
Bo_TE_46374	0.70732(29/41)	0.23529(4/17)	0.15000(3/20)	0.80000(16/20)
Bo_TE_10791	0.20455(9/44)	0.80000(12/15)	0.86957(20/23)	0.90000(18/20)
Bo_TE_33695	0.05405(2/37)	0.15385(2/13)	0.26087(6/23)	0.63158(12/19)
Bo_TE_136448	0.86364(38/44)	0.06250(1/16)	0.04348(1/23)	0.00000(0/19)
Bo_TE_79636	0.75556(34/45)	0.23529(4/17)	0.68182(15/22)	0.10000(2/20)
Bo_TE_149085	0.80952(34/42)	0.61111(11/18)	0.00000(0/23)	0.00000(0/20)
Bo_TE_103953	0.02174(1/46)	0.47059(8/17)	0.50000(11/22)	0.70000(14/20)
Bo_TE_227064	0.46154(6/13)	0.37500(6/16)	1.00000(23/23)	0.93750(15/16)
Bo_TE_120804	0.42857(18/42)	0.64706(11/17)	0.95238(20/21)	1.00000(20/20)
Bo_TE_94486	1.00000(44/44)	0.83333(15/18)	0.52381(11/21)	0.47368(9/19)
Bo_TE_88644	0.75610(31/41)	0.40000(6/15)	0.80952(17/21)	0.15789(3/19)
Bo_TE_155785	0.58140(25/43)	0.27778(5/18)	0.04545(1/22)	0.10000(2/20)
Bo_TE_162993	0.62222(28/45)	0.88235(15/17)	0.35000(7/20)	0.25000(5/20)
Bo_TE_239369	0.00000(0/43)	0.50000(9/18)	0.80000(16/20)	0.10000(2/20)
Bo_TE_85589	0.52381(22/42)	0.55556(10/18)	0.26087(6/23)	0.00000(0/20)
Bo_TE_116782	0.93478(43/46)	0.94118(16/17)	0.22727(5/22)	0.17647(3/17)
Bo_TE_41777	0.11111(5/45)	0.00000(0/19)	0.65217(15/23)	0.21053(4/19)
Bo_TE_55374	0.63415(26/41)	0.00000(0/16)	0.00000(0/22)	0.00000(0/20)
Bo_TE_158793	0.00000(0/46)	0.55556(10/18)	0.00000(0/23)	0.00000(0/20)
Bo_TE_86563	0.73171(30/41)	0.06250(1/16)	0.19048(4/21)	0.25000(5/20)
Bo_TE_136478	0.85366(35/41)	0.05882(1/17)	0.68182(15/22)	0.68421(13/19)
Bo_TE_2072	0.06667(3/45)	0.38889(7/18)	0.71429(15/21)	0.42105(8/19)
Bo_TE_168931	1.00000(44/44)	0.33333(6/18)	0.75000(15/20)	0.95000(19/20)
Bo_TE_12966	0.86957(40/46)	0.05882(1/17)	0.18182(4/22)	0.36842(7/19)
Bo_TE_2188	0.09302(4/43)	0.11765(2/17)	0.59091(13/22)	0.00000(0/20)
Bo_TE_6070	0.00000(0/39)	0.00000(0/18)	0.80952(17/21)	0.77778(14/18)
Bo_TE_113341	0.52381(22/42)	0.06250(1/16)	0.40000(8/20)	0.00000(0/19)
Bo_TE_70756	0.09091(4/44)	0.05882(1/17)	0.17391(4/23)	0.57895(11/19)

Bo_TE_90208	0.95122(39/41)	0.52941(9/17)	0.40000(8/20)	0.21053(4/19)
Bo_TE_32642	0.16667(7/42)	0.89474(17/19)	0.47368(9/19)	0.20000(4/20)
Bo_TE_234323	0.02222(1/45)	0.47059(8/17)	0.63636(14/22)	0.68421(13/19)
Bo_TE_224884	0.41860(18/43)	0.83333(15/18)	0.61905(13/21)	0.15000(3/20)

[a] The population frequencies of TE loci were measured as the ratio of the number of accessions carrying TE insertions to the total number of accessions with non-missing calls at the corresponding loci.

Supplementary Table 4 The proportion of each frequency type in different superfamilies from *B. oleracea* and *A. thaliana*.

	B. oleracea				*A. thaliana*			
Superfamily	Low	Median	High	No. of loci	Low	Median	High	No. of loci
Copia	62.1%	24.5%	13.4%	6,327	73.0%	6.0%	21.0%	1,882
Gypsy	57.3%	28.8%	13.8%	1,423	54.0%	7.0%	40.0%	4,876
SINE	61.1%	27.9%	11.0%	1,661	58.0%	3.0%	38.0%	65
LINE	58.5%	18.9%	22.6%	164	74.0%	6.0%	19.0%	1,147
CACTA	63.2%	24.3%	12.5%	4,416	64.0%	5.0%	31.0%	1,031
Tc1/Mariner	55.8%	25.3%	18.9%	2,402	54.0%	7.0%	38.0%	149
hAT	62.3%	24.2%	13.6%	1,275	79.0%	5.0%	16.0%	1,182
Mutator	59.4%	25.1%	15.5%	1,133	73.0%	4.0%	22.0%	4,246
PIF/Harbinger	68.3%	21.2%	10.5%	1,112	74.0%	6.0%	20.0%	360
Helitron	51.8%	29.9%	18.3%	7,092	70.0%	4.0%	26.0%	6,847
Pong	51.0%	29.0%	19.9%	4,447	56.0%	7.0%	37.0%	148
Others	55.9%	28.9%	15.2%	743	56.3%	9.4%	34.3%	1,162
All	57.7%	26.7%	15.6%	32,195	66.9%	5.4%	27.7%	23,095

Supplementary Table 5 Statistics of consistency between the two results from reference assembly and NGS reads in the two references at 1,000 randomly selected loci.

	Positive	Negative	False Positive	False Negative	Heterozygous	Misssing	Accuracy
line 02-12	506	466	23	3	2	0	97.4%
TO1000 DH	458	492	18	5	21	6	97.6%

Note: The accuray was calculated as (Positive + Negative) / (Positive + Negative + False Positive + False Negative);
Positive : the presence of TE insertion in the both results from reference assembly and NGS reads;
Negative: the absence of TE insertion in the both results;
False Positive: the absence of TE insertion in the result from reference assembly, but presence from NGS reads;
False Negative: the presence of TE insertion in the result from reference assembly, but absence from NGS reads;
Heterozygous: the presence and absence of TE insertion were detected simultaneously by NGS reads;
Missing: No result can be obtained by NGS reads due to lacking the corresponding chimeric reads spanning the breakpoint;
